# Understanding Conformational Preferences of Atropisomeric Hydrazides and Its Influence on Excited State Transformations in Crystalline Media

**DOI:** 10.3390/molecules24163001

**Published:** 2019-08-19

**Authors:** Akila Iyer, Angel Ugrinov, J. Sivaguru

**Affiliations:** 1 Center for Photochemical Sciences and Department of Chemistry, Bowling Green State University, Bowling Green, OH 43403, USA; 2 Department of Chemistry and Biochemistry, North Dakota State University, Fargo, ND 58108-6050, USA

**Keywords:** atropselective photoreactions, photochirogenesis, asymmetric photochemistry, chiral photochemistry, photochiral auxiliary, hydrazide based photochromophores

## Abstract

Hydrazides derivatives were evaluated to understand the role of *N*–*N* bond in dictating the outcome of photoreactions in the solid state.

## 1. Introduction

**Scheme 1:**
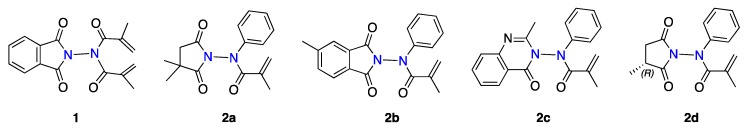
Hydrazides with restricted *N*–*N* bond rotation evaluated towards solid state photoreaction.

Nature employs light as a powerful reagent to perform chemical transformations for many life-sustaining processes. Scientists have tried to mimic the processes in nature to design and develop methodologies for the synthesis of complex structural scaffolds and for building photoresponsive materials and molecular assemblies with unique architectures [1,2]. One of the critical aspects to control chemical transformations from the excited state involves harnessing the reactivity and dynamics of photoexcited species. Supramolecular assemblies, in particular crystalline media, have potential to channel the reactivity of the excited states towards useful chemical pathways [3]. Recently, research from our group established the use of hydrazides as effective photoauxiliary for carrying out various excited state transformations [4,5]. In this report, we have evaluated the role of hydrazides as photoactive units in crystalline media. Gilchrist and coworkers reported the photochemistry of hydrazides [6] in which 1-phthalimidoaziridine underwent photoaddition with alkenes to yield the corresponding aziridine skeleton. The proof of concerted fragmentation of the phthalimidoaziridines to give olefin and corresponding phthalimidonitrene was confirmed by trapping the nitrene. Couture and coworkers [7,8] reported an efficient strategy that utilizes non-oxidative, photoinduced *N*–*N* bond cleavage. Watterson and coworkers [9] reported that irradiation of acyclic hydrazides resulted in *N*–C(CO) (carbonyl) and *N*–*N* bond cleaved products. Based on literature precedence, our group has developed hydrazides as a photochiral auxiliary to carry out excited state transformations [4,5]. In this report, we present our results on the influence of *N*–*N* bond in controlling the excited state reactivity of compounds **1** and **2a**–**d** in the solid state (Scheme 1).

## 2. Results and Discussion

**Figure 1:**
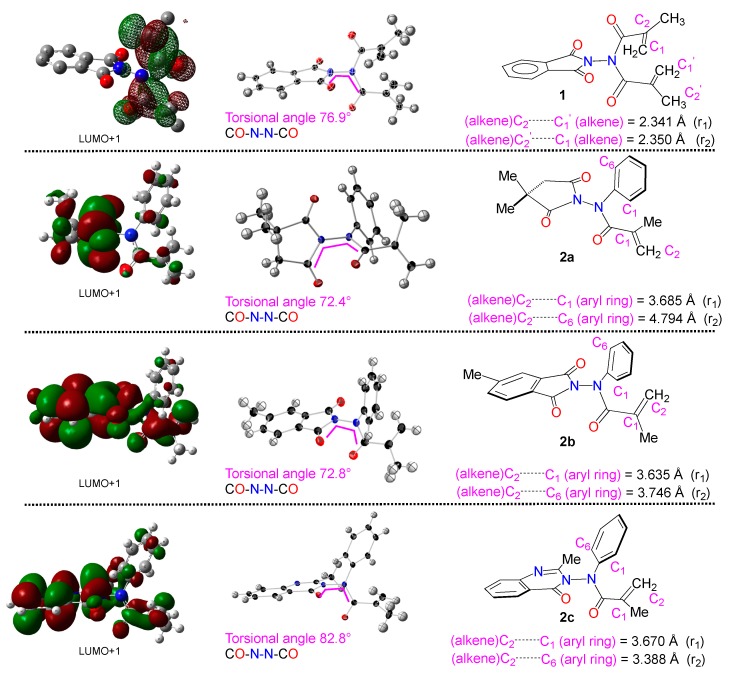
Understanding the solid state photochemical reactivity based on orbital density computed with Gaussian 09 at B3LYP/6-31G* and single crystal XRD analysis of hydrazides **1**, **2a**, **2b**, and **2c**.

An important and unique feature shown by hydrazides in crystalline media relates to the pyramidalization of the nitrogen atom [10,11,12,13,14,15,16,17,18,19,20,21,22]. There have been few reports in the literature on the single crystal XRD analysis of *N*–*N* bond based compounds that shed light on this unusual feature and describe such hydrazides as atypical compounds. Aitken and coworkers attribute the *N*–*N* bond twist in *N-*Acetylamino phthalimide to intermolecular H-bonding interactions [22]. They observed an unusually large torsional angle θ (CO–*N*–*N*–CO) of ~86° that explains the orthogonality between the two planes containing each nitrogen atom. As we were successful in obtaining good quality crystals for single crystal XRD analysis of hydrazides **1** and **2** (Figure 1), we became interested to understand the conformational features and how they impacted reactivity in the excited state [23]. The torsional angles θ (CO–*N*–*N*–CO) for **1**, **2a**, **2b**, and **2c** was ~76.9°, ~72.4°, ~72.8°, and ~82.8°, respectively (Figure 1; Table 1). For quinazolinone based hydrazide **2c** the measured torsional angles θ being ~82.8° confirmed the planes of the groups on the two nitrogen atoms to be nearly perpendicular. This is expected from the repulsive interactions between the lone pair on the two heteroatoms.

**Figure 2:**
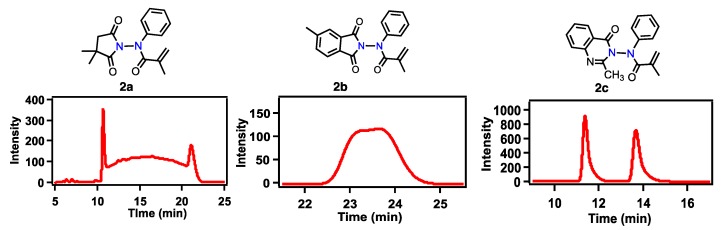
HPLC analysis of hydrazides **2a**–**c** on a chiral stationary phase. HPLC condition: Figure 2. CHIRALPAK^®^ IC, mobile phase hexane:2-propanol 70:30, flow rate: 1.0 mL/min, retention times 10.6 min and 20.9 min; **2b** CHIRALPAK^®^ IC, mobile phase-hexane:2-propanol 70:30, flow rate: 0.8 mL/min, HPLC condition; broad peak from 22.4–25.0 min; **2c** CHIRALPAK^®^ AD-H, mobile phase hexane:2-propanol 70:30, flow rate: 0.8 mL/min, retention times 11.4 min and 13.7 min.

As monoacylated nitrogen on hydrazides exhibit axial chirality (Δ*G*^‡^*_rac_* ~ 23 kcal/mol, *T*_c_ = 188 °C) [15,24], we evaluated hydrazides **2a–c** as they have asymmetrical substitution around the *N*–*N* bond. HPLC analysis of hydrazides (Figure 2) using chiral stationary phase showed partial separation of enantiomers for **2a** and **2b** indicating that the *N*–*N* bond rotation at room temperature is not favorable for resolution of the enantiomers. Verma and coworkers have extensively documented studies [14,15,25] on restricted *N*–*N* bond rotation in camphorimide/other dimidyl based hydrazides and have mentioned moderate to high rotational barrier by NMR spectral analysis [26,27,28,29,30,31,32,33]. In case of succinimide **2a**, peak shape characteristics reflected two resolved peaks albeit with fast rotation around the *N*–*N* bond under our separation conditions [34,35]. We then synthesized chiral phthalimide-derived hydrazide **2b** and the HPLC separation did not show any base-to-base separation at room temperature (Figure 2). The 5,6-fused aromatic ring system in phthalimide **2b** was then altered to a 6,6-fused aromatic ring system of quinazolinone-based acrylanilide **2c** as quinazolinone diacylated hydrazides have been shown to yield stable atropisomers [18,20,21,36,37,38,39,40,41]. 

Based on chiral stationary phase based HPLC analysis, the quinazolinone derived hydrazide **2c** showed well-resolved peaks for the individual *P* and *M* atropisomers (Figure 2). Table 2 reveals the kinetic parameters for single bond rotation for **2c** where appreciable barrier for *N*–*N* bond rotation was observed. The barrier for racemization (Δ*G*^‡^*_rac_*) in ethyl acetate (polar solvent) was found to be ~24.2 kcal/mol at 45 °C with a racemization rate constant (*k*_rac_) of 16.4 × 10^−5^ s^−1^ that corresponded to a half-life of racemization (τ_1/2_) of 1.2 h. Changing the solvent from ethyl acetate to benzene (non-polar solvent) had a minor influence on the racemization kinetic parameters. The activation barrier for racemization (Δ*G*^‡^*_rac_*) at 45 °C was found to be ~23.9 kcal/mol with a racemization rate constant (*k*_rac_) of 2.15 × 10^−4^ s^−1^ that corresponded to a half-life of racemization (τ_1/2_) of 0.89 h. This suggests that the *N*–*N* bond based optically pure atropisomers could be stable atropisomers at room temperature.

**Scheme 2:**
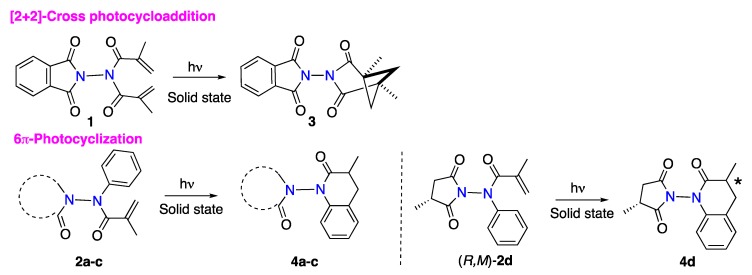
Photoreactivity of hydrazides in the solid state.

Having established the importance of axial chirality centered around *N*–*N* bond, we evaluated hydrazide **1** for [2+2] photocycloaddition and hydrazides **2a**–**c** towards 6π-photocyclization, to understand the effect of conformational preferences in crystalline media and their impact on the excited state reactivity. The photoreactions were performed under the conditions of direct irradiation by exposing the crystals to appropriate wavelength of light. The acrylimide based hydrazide **1** underwent solid state [2+2] photoreaction to afford corresponding cycloadduct **3** (Scheme 2). The photoproduct was characterized by ^1^H-NMR spectroscopy. While [2+2]-photocycloaddition of hydrazide in the solid state occurred as expected, the 6π-photocyclization of hydrazides showed some interesting trends. Hydrazides **2b** and **2c** on irradiation underwent 6π-photocyclization in solid state to the corresponding cyclized product **4b** and **4c**, respectively. However, the hydrazide **2a** on subjecting to two different light sources viz λ~300 nm and λ~254 nm did not afford the corresponding cyclized product. The crystals were stable and no decomposition was observed. The stability of the crystal was monitored until 72 h by single crystal XRD. We postulate the bond distances/the conformation make-up of the hydrazide in the crystalline lattice to be the plausible reason that explains the ability/inability of these substrate to undergo solid state photoreaction. To gain better understanding we performed DFT calculations at B3LYP/6-31G* level of theory using the cartesians obtained from single crystal XRD structures. The LUMO+1 states for succinimide based acrylanilide **2a** showed negligible orbital localization on the C_2_ carbon (C_1_=C_2_) of methacryloyl functionality when compared to other hydrazides (Figure 1). An appreciable orbital density was observed on the terminal alkenyl carbon for the hydrazides that underwent photoreaction in solid state.

A closer examination of single crystal XRD structure suggests that only in the case of succinimide based hydrazide **2a**, the terminal alkene carbon points away from the phenyl group. Further the measured bond distances from the single crystal XRD structures for the distances between C1/C6 carbon atom(s) of the phenyl ring to the terminal alkenyl carbon for **2b** and **2c** were both within the Schmidt distance (for **2b** r1 = 3.635 Å and r2 = 3.746 Å; for **2c** r1 = 3.670 Å and r2 = 3.388 Å). However, for hydrazide **2a** the measured bond distance was slightly larger than the optimal distance for C6 (phenyl)–C2 (C_1_=C_2_) bond (r1 = 3.685 Å and r2 = 4.794 Å).

**Figure 3:**
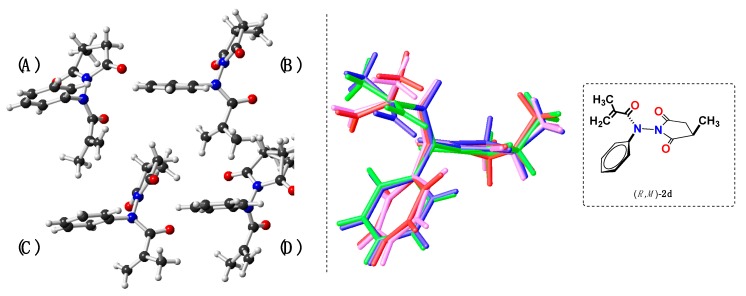
Single crystal XRD analysis performed for (*R*)-methyl succinimide based hydrazide **2d**. A–D represents (R,M)-**2d** in a unit cell. The CCDC Deposition Number 1943951. (Refer to supporting information).

To bias the restricted bond rotation towards one atropisomer, (*R*)-methyl succinimide based hydrazide **2d** (Scheme 2; Figure 3) with a point chiral center was evaluated both in solution and in the solid state. The presence of the chiral center, forced crystallization of the (*R*,*M*)-isomer of **2d** leading to optical resolution of the diastereomers. Inspection of the crystal structures showed four molecules per unit cell (Figure 3, structure A, B, C and D) with both *s-cis* (Figure 3A,D) and *s-trans* (Figure 3B,C) OC–C rotamer in the same unit cell. The structural overlay (Figure 3 right) indicates that the *N*-phenyl ring exhibits different orientation within the individual rotamers. As the cyclization distance was optimal in the solid state, irradiation of the crystals of (*R*,*M*)-**2d** resulted in the photocyclized product **4d** (Scheme 2). Slow rotation of *N–N* bond leading to diasteromerization was observed during the HPLC analysis of the photoproduct **4d**. Nevertheless, the results clearly showcase the ability of restricted bond rotation around *N–N* bond and crystalline confinement can lead to good stereoenrichment in the photoproduct.

## 3. Conclusions

Our study clearly demonstrates the power of *N–N* bond to enforce reactivity in the crystalline state. The conformational aspects of the hydrazides in the present study corroborates with literature precedence. The orthogonality of the planes containing the two nitrogen centers provides a useful handle to control the stereochemistry of photoreactions in the solid state. The research lays down the path for developing chirality around *N–N* bond in hydrazides and channels its excited state energy to afford synthetically useful scaffolds in crystalline media.

## 4. Materials and Methods

The synthesis and characterization of hydrazides **1** and **2a**–**c** as well as their corresponding photoproducts are reported in our previous communications [4,5,23]. All commercially obtained reagents/solvents were used as received; chemicals that were purchased from Alfa Aesar^®^ (Ward Hill, MA, USA) Sigma—Aldrich^®^ (St. Louis, MO, USA), Acros^®^ (Geel, Belgium), TCI^®^ America (Portland, OR, USA), Mallinckrodt^®^ (Petten, The Netherlands), and Oakwood Products^®^ (West Columbia, Anaheim, CA, USA) were used as received without further purification. ^1^H-NMR and ^13^C-NMR spectra were recorded on Varian 400 MHz (100 MHz) and on 500 MHz (125 MHz) spectrometers. HPLC analyses were performed on Waters^®^ HPLC (Milford, MA, USA) equipped with 2525 pump or on Dionex^®^ Ultimate 3000 HPLC (Thermo Fisher Scientific, Bremen, Germany or Thermo Fisher Scientific Inc., Sunnyvale, CA, USA). Waters^®^ 2767 sample manager was used for automated sample injection. Chromeleon 7.0 (Dionex Thermoline Fisher Scientific, Waltham, MA, USA) software was used for analyzing HPLC injections on Dionex ^®^ HPLC. The reactants and photoproducts were purified by flash chromatography using silica gel (by standard technique with solvents as indicated).

High-resolution mass spectrum data in Electrospray Ionization mode were recorded on a Bruker—Daltronics^®^ BioTof mass spectrometer (Billerica, MA, USA) in positive (ESI+) ion mode. Single crystal X-ray diffraction data of the compounds **1**, **2a**–**d** were collected on a Bruker Apex Duo diffractometer with an Apex 2 CCD area detector at T = 100K. ImSCu source radiation was used in all the cases and the structures were processed using Apex 2 v2010.9-1 software package (Bruker-AXS, Madison, WI, USA). Direct method was used to solve the structures after multi-scan absorption corrections.

**Scheme 3:**
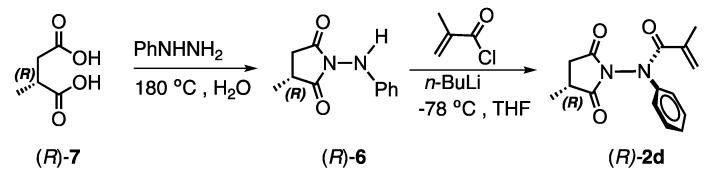
Synthesis of hydrazide derivative *(R*)-**2d.**

Following the modified procedure reported by Kamiński et al., a suspension of *(R)*-(+)-methyl-succinic acid (*R*)-**7** (1 equiv) in water (10 mL per gram of acid), phenyl hydrazine (1 equiv) was added [42]. The mixture was heated in an oil bath at 180 °C with simultaneous removal of water (Scheme 3). After 1 h, the mixture was brought to room temperature and the crude was diluted with ethyl acetate and the organic layer was sequentially washed with 10% HCl, (2 × 10 mL), DI water (2 × 10 mL), saturated NaHCO_3_ (2 × 10 mL), and finally with brine. The organic layer was dried over *anhyd* Na_2_SO_4_, filtered and the solvent was removed under reduced pressure to yield crude product. After concentrating the organic layer, the crude product was purified by combiflash using hexanes and ethyl acetate mixture to get the desired hydrazide (*R*)-**6**. TLC condition—R*_f_*= 0.3 (50% ethyl acetate:hexanes). Crystalline solid (Yield = 40%). ^1^H-NMR (400 MHz, CDCl_3_, δ ppm): 1.41 (d, 3H, *J* 7.2Hz), 2.44 (dd, 1H, *J*_1_ 17.6Hz, *J*_2_ 3.6 Hz), 2.95–3.07 (m, 2H), 6.09 (bs, 1H), 6.76 (d, 2H, *J* 8 Hz), 6.97 (t, 1H, *J* 7.6 Hz), and 7.21–7.25 (m, 2H). ^13^C-NMR (100 MHz, CDCl_3_, δ ppm): 17.2, 34.3, 34.9, 114.9, 123.0, 129.6, 145.3, 174.1, and 179.4.

Hydrazide derivative (*R*)-**6** (1 equiv) was dissolved in anhydrous THF under an inert atmosphere.^23^ The solution was then cooled to −78 °C. To this cold solution *n*-BuLi (2.5 M, 1.1 equiv) was added slowly. The mixture was stirred at this temperature for 1 h. The reaction was quenched with anhydrous methacryloyl chloride (1.3 equiv). The stirring was continued for another 2 h after which the solution was quenched with slow addition of ~10 mL of saturated NH_4_Cl. (Note: The solution was quenched at −78 °C). The solution was further stirred for 35 min and then diluted with diethyl ether and the organic layer was sequentially washed with DI water (2 × 10 mL), saturated NaHCO_3_ (2 × 10 mL), and finally with brine. The organic layer was dried over *anhyd* Na_2_SO_4_, filtered, and the solvent was removed under reduced pressure to yield crude product. After concentrating the organic layer, the crude product was purified by combiflash using hexanes and ethyl acetate mixture to get the desired compound. TLC condition—R*_f_*= 0.6 (50% ethyl acetate:hexanes). Crystalline clear solid (Yield = 75%). ^1^H-NMR (400 MHz, CDCl_3_, δ ppm, rotamer peaks are reported together): 1.29 (d, *J* 6.8 Hz), 1.39–1.43 (m), 1.83 (bs), 2.28–2.45 (m), 2.84–3.05 (m), 5.19 (s), 5.27 (m), 7.29–7.35 (m), and 7.40–7.43 (m). ^13^C-NMR (100 MHz, CDCl_3_, δ ppm, rotamer peaks are reported together): 21.7, 22.0, 22.1, 38.4, 38.5, 39.9, 40.0, 41.9, 131.6, 132.7, 132.9, 133.8, 133.9, 133.9, 134.3, 134.5, 134.6, 177.5, 177.7, 181.6, and 181.7. HRMS-ESI (*m*/*z*) [M + Na]: Chemical Formula: C_16_H_20_N_2_O_3_, calculated: 295.1059, observed: 295.1073, |Δm|: 4.7 ppm.

Condition for crystallization: 

The hydrazide (5 mg) was added to a well-dried pyrex tube and then dissolved in an appropriate solvent (minimum volume). The solution was layered with another solvent (minimum volume) in which the hydrazide was insoluble. The vessel was then sealed with a rubber septum and a needle inserted on the septum to allow slow evaporation of the solvent. Solvent mixture used for crystallization for hydrazides: **1**, **2a**–**d**: Hexane/ethyl acetate. CCDC Deposition Number 1943951.

## Figures and Tables

**Table 1:** Torsional angle (θ) (CO–*N*–*N*–CO) of the evaluated hydrazides ^a^.

Entry	Hydrazide	Torsional Angle (θ)
1	**1**	76.9°
2	**2a**	72.4°
3	**2b**	72.8°
4	**2c**	82.8°

^a^ Torsional angle (θ) were measured from single crystal XRD analysis of hydrazides.

**Table 2:** Kinetic parameters of racemization barrier for hydrazide **2c** ^a^.

Entry	Solvent	Kinetic Parameters		
		τ_1/2_ (h)	*k_rac_* (s^−1^)	Δ*G*^‡^*_rac_* (kcal mol^−1^)
1	Benzene	0.89	2.15 × 10^−4^	23.9
2	Ethyl acetate	1.20	16.4 × 10^−5^	24.2

^a^ Values carry an error of ±5%. The racemization kinetics was followed at 45 °C by HPLC analysis on a chiral stationary phase.

## Supplementary Materials

The following are available: Single crystal XRD (CIF format), and characterization data. Single crystal X-ray data can be obtained from the supplementary materials ofor this paper. These data can also be obtained free of charge via http://www.ccdc.cam.ac.uk/conts/retrieving.html (or from the CCDC, 12 Union Road, Cambridge CB2 1EZ, UK; Fax: +44-1223-336033; E-mail: deposit@ccdc.cam.ac.uk) using CCDC Deposiotn number 1943951.
