# Supplementary material for: Understanding Conformational Preferences of Atropisomeric Hydrazides and Its Influence on Excited State Transformations in Crystalline Media
Source: Molecules. 2019 Aug 19;24(16):3001. doi: 10.3390/molecules24163001 (PMC6719012; doi:10.3390/molecules24163001)
Supplement: Supplementary file 1 [file molecules-24-03001-s001.zip › sp-proof/Molecules_ESI_&XRD_Final.pdf]

Supporting Information (22 Pages)

Understanding conformational preferences of atropisomeric hydrazides and its influence on excited state transformations in crystalline media.

Akila Iyer,[a,b] Angel Ugrinov,[a] and Jayaraman Sivaguru[b*]

[a] North Dakota State University, Department of Chemistry and Biochemistry, Fargo, ND 43403 USA.

[b] Center for Photochemical Science and Department of Chemistry, Bowling Green State University, Bowling Green, Ohio 43403, USA

e-Mail: sivagj@bgsu.edu

Table of Contents

1. General methods 2
1.1. Single crystal XRD analysis 3
2. Chemical structures of hydrazides and corresponding photoproducts 4
3. Synthetic protocol for the synthesis of hydrazide derivative (*R*)-2d 5
3.1. Synthesis of hydrazide derivative (*R*)-**6** 5
3.2. Synthesis of hydrazide derivative (*R*)-**2d** 8
4. General irradiation procedures and characterization of photoproduct(s) 15
4.1. Procedure for photoreaction 15
5. Photoreaction of hydrazide derivative (*R*)-2d in solution 16
5.1. Characterization of photoproduct (*R*)-**4d** 17
6. HPLC analysis for hydrazides 19
6.1. HPLC analysis for hydrazide derivative (R)-**6** 19
6.2. HPLC analysis for hydrazide derivative (*R*)-**2d** 19
6.3. HPLC analysis conditions for hydrazide derivative (*R*)-**4d** by direct irradiation (crude sample was injected) 20
6.4. HPLC analysis conditions for hydrazide derivative (*R*)-**4d** by sensitized irradiation (crude sample was injected) 20
7. Racemization kinetics of hydrazide derivative 2c 21
8. References 22

1. General methods

All commercially obtained reagents/solvents were used as received; chemicals were purchased from Alfa Aesar®, Sigma-Aldrich®, Acros organics®, TCI America®, Mallinckrodt®, and Oakwood® Products, and were used as received without further purification. The synthesis and characterization of hydrazides **1** and **2a-c** as well as their corresponding photoproducts i.e. **3** and **4a-c** are reported in our previous communications.[1] Unless stated otherwise, reactions were conducted in oven-dried glassware under nitrogen atmosphere. The compounds were purified by combiflash equipped with dual wavelength UV-Vis absorbance detector (Teledyne ISCO) using hexanes: ethyl acetate as the mobile phase and Redisep® cartridge filled with silica (Teledyne ISCO) as stationary phase. In some cases, compounds were purified by column chromatography on silica gel (Sorbent Technologies®, silica gel standard grade: porosity 60 Å, particle size: 230 x 400 mesh, surface area: 500 – 600 m2/g, bulk density: 0.4 g/mL, pH range: 6.5 – 7.5). Unless indicated, the Retardation Factor (R_f) values were recorded using a 5-50% hexanes:ethyl acetate as mobile phase and on Sorbent Technologies®, silica Gel TLC plates (200 mm thickness w/UV254).

The residual solvent signal was used as reference. ($CDCl_3$: δ_H = 7.26 ppm, δ_C = 77.2 ppm). Data for ^{1}H NMR spectra are reported as follows: chemical shift (δ ppm), multiplicity, coupling constant (Hz) and integration. The following abbreviations were used to explain the multiplicities: s = singlet, d = doublet, t = triplet, q = quartet, m = multiplet, quin = quintuplet, sext = sextet, sep = septet, b = broad. In some instances, it was not possible to obtain a signal for the carbonyl carbon, despite long relaxation times and concentrated samples. However these signals are reported wherever possible. High-resolution mass spectrum data in Electrospray Ionization mode were recorded on a Bruker – Daltronics® BioTof mass spectrometer in positive (ESI+) ion mode.

HPLC analyses were performed on Waters® HPLC equipped with 2525 pump or on Dionex® Ultimate 3000 HPLC. Waters® 2767 sample manager was used for automated sample injection. All HPLC injections were monitored using a Waters® 2487 dual wavelength absorbance detector at 254 nm and 270 nm. Analytical and semi-preparative injections were performed on chiral stationary phase using various columns indicated below:

i. Regis® PIRKLE COVALENT (R,R) WHELK–01

a) 25 cm x 4.6 mm column for analytical injections

b) 25 cm x 10 mm column for semi-preparative injections.

ii. CHIRACEL® OD-H

a) 0.46 cm x 25 cm column for analytical injections

b) 25 cm x 10 mm column for semi-preparative injections.

iii. CHIRALPAK® IC

a) 0.46 cm x 25 cm column for analytical injections

b) 10 mm x 25 cm column for semi-preparative injections

iv. CHIRALPAK® AD-H

a) 0.46 cm x 25 cm column for analytical injections

b) 25 cm x 10 mm column for semi-preparative injections

Masslynx software version 4.1 was used to analyze/process the HPLC injections. Masslynx software version 4.1 was used for analyzing HPLC injections on Waters®. Chromeleon 7.0 software was used for analyzing HPLC injections on Dionex ® HPLC. Igor Pro® Software version 3.0 was used for processing HPLC chromatograms.

1.1. Single crystal XRD analysis

Single crystal X-ray diffraction data of the compounds **2a** and **2c** were collected on a Bruker Apex Duo diffractometer with a Apex 2 CCD area detector at T = 100 K. ImSCu source radiation was used. All structures were process with Apex 2 v2010.9-1 software package (SAINT v. 7.68A, XSHELL v. 6.3.1). Direct method was used to solve the structures after multi-scan absorption corrections.

2. Chemical structures of hydrazides and corresponding photoproducts

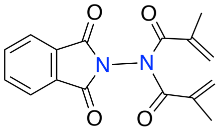

1

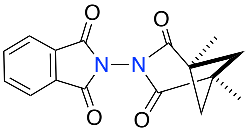

3

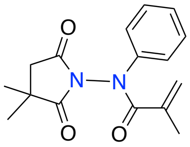

2a

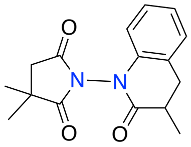

4a

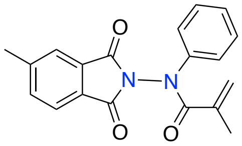

2b

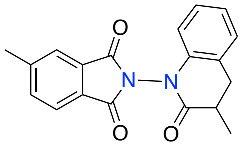

4b

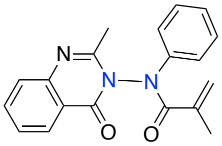

2c

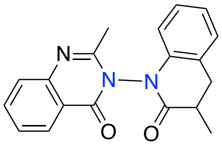

4c

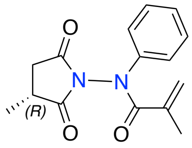

2d

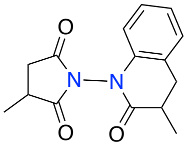

4d

3. Synthetic protocol for the synthesis of hydrazide derivative (*R*)-2d

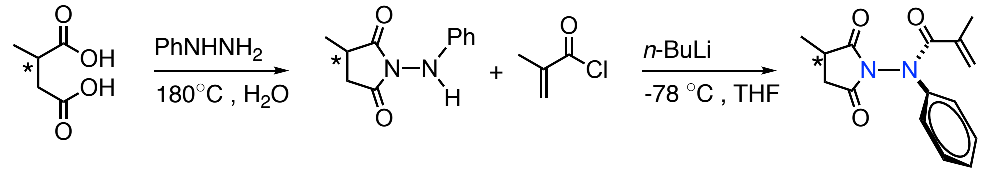

(*R*)-**7** (*R*)-**6** (*R*)-**2d**

Scheme S1: Synthesis of hydrazide derivative (*R*)-**2d**.

3.1. Synthesis of hydrazide derivative (*R*)-6

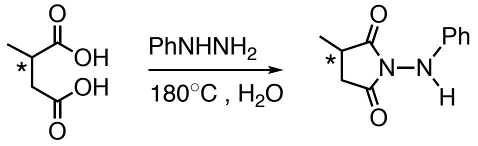

(*R*)-**7** (*R*)-**6**

Scheme S2: Synthesis of hydrazide derivative (*R*)-**6**

Following the modified procedure reported by Kamiński et al., a suspension of *(R)*-(+)-methyl-succinic acid (*R*)-**7** (1 equiv) in water (10 mL per gram of acid), phenyl hydrazine (1 equiv) was added.[2] The mixture was heated in an oil bath at 180 °C with simultaneous removal of water. After 1 h, the mixture was brought to room temperature and the crude was diluted with ethyl acetate and the organic layer was sequentially washed with 10% HCl, (2 × 10 mL), DI water (2 × 10 mL), saturated $NaHCO_3$ (2 × 10 mL) and finally with brine. The organic layer was dried over anhyd Na_2SO_4, filtered and the solvent was removed under reduced pressure to yield crude product. After concentrating the organic layer, the crude product was purified by combiflash using hexanes and ethyl acetate mixture to get the desired compound.

TLC condition - R_f = 0.3 (50% ethyl acetate:hexanes). Crystalline solid (Yield = 40%)

^{1}H-NMR (400 MHz, $CDCl_3$, δ ppm): 1.41 (d, 3H, *J* 7.2Hz), 2.44 (dd, 1H, J_1 17.6Hz, J_2 3.6Hz), 2.95-3.07 (m, 2H), 6.09 (bs, 1H), 6.76 (d, 2H, *J* 8Hz), 6.97 (t, 1H, *J* 7.6Hz) and 7.21-7.25 (m, 2H).

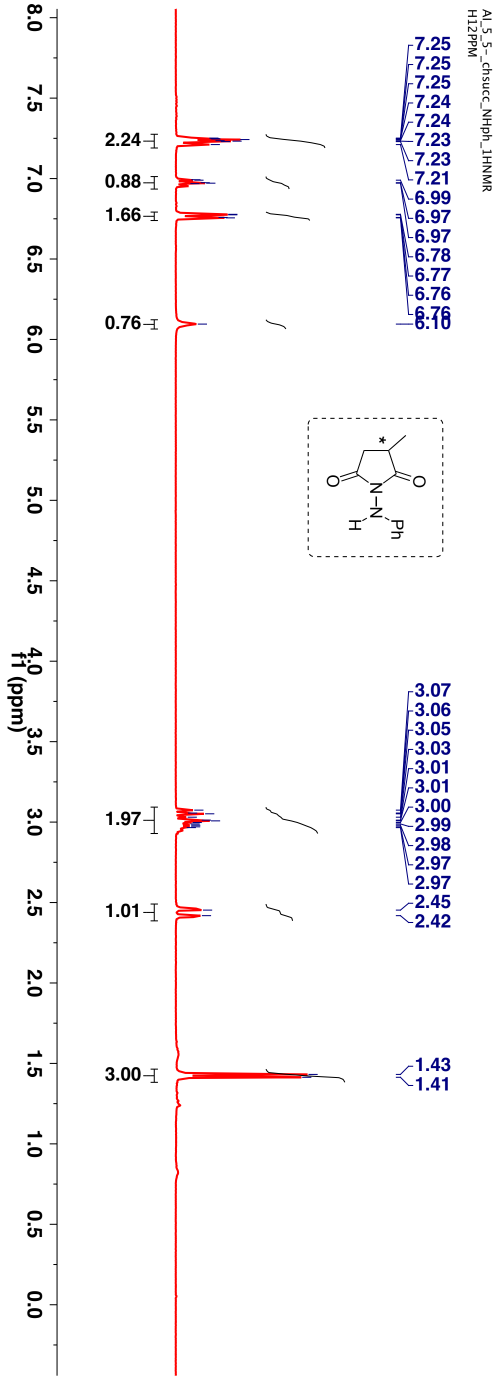

^{13}C-NMR (100 MHz, $CDCl_3$, δ ppm): 17.2, 34.3, 34.9, 114.9, 123.0, 129.6, 145.3, 174.1 and 179.4.

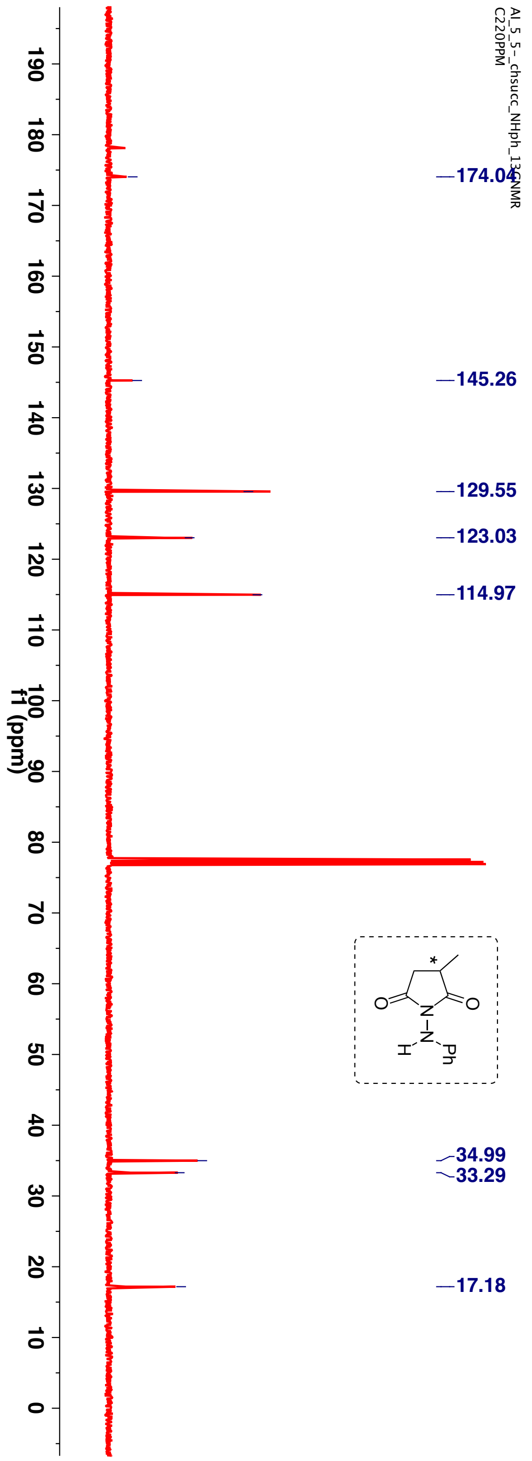

3.2. Synthesis of hydrazide derivative (*R*)-**2d**

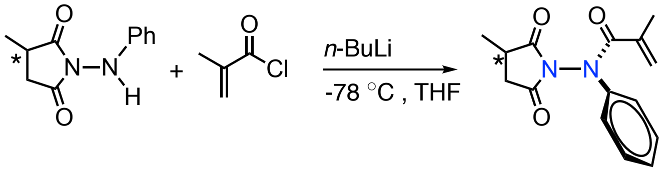

(*R*)-**6** (*R*)-**2d**

Scheme S3: Synthesis of hydrazide derivative (*R*)-**2d**

Hydrazide derivative (*R*)-**6** (1 equiv) was dissolved in anhydrous THF under an inert atmosphere. The solution was then cooled to -78 °C. To this cold solution *n*-BuLi (2.5 M, 1.1 equiv) was added slowly. The mixture was stirred at this temperature for 1 h. The reaction was quenched with anhydrous methacryloyl chloride (1.3 equiv). The stirring was continued for another 2 h after which the solution was quenched with slow addition of ~10 mL of saturated NH_4Cl. (Note: the solution was quenched at -78 °C). The solution was further stirred for 35 min and then diluted with diethyl ether and the organic layer was sequentially washed with DI water (2 × 10 mL), saturated $NaHCO_3$ (2 × 10 mL) and finally with brine. The organic layer was dried over anhyd Na_2SO_4, filtered and the solvent was removed under reduced pressure to yield crude product. After concentrating the organic layer, the crude product was purified by combiflash using hexanes and ethyl acetate mixture to get the desired compound.

TLC condition - R_f = 0.6 (50% ethyl acetate:hexanes).

Crystalline clear solid (Yield = 75%).

Crude NMR

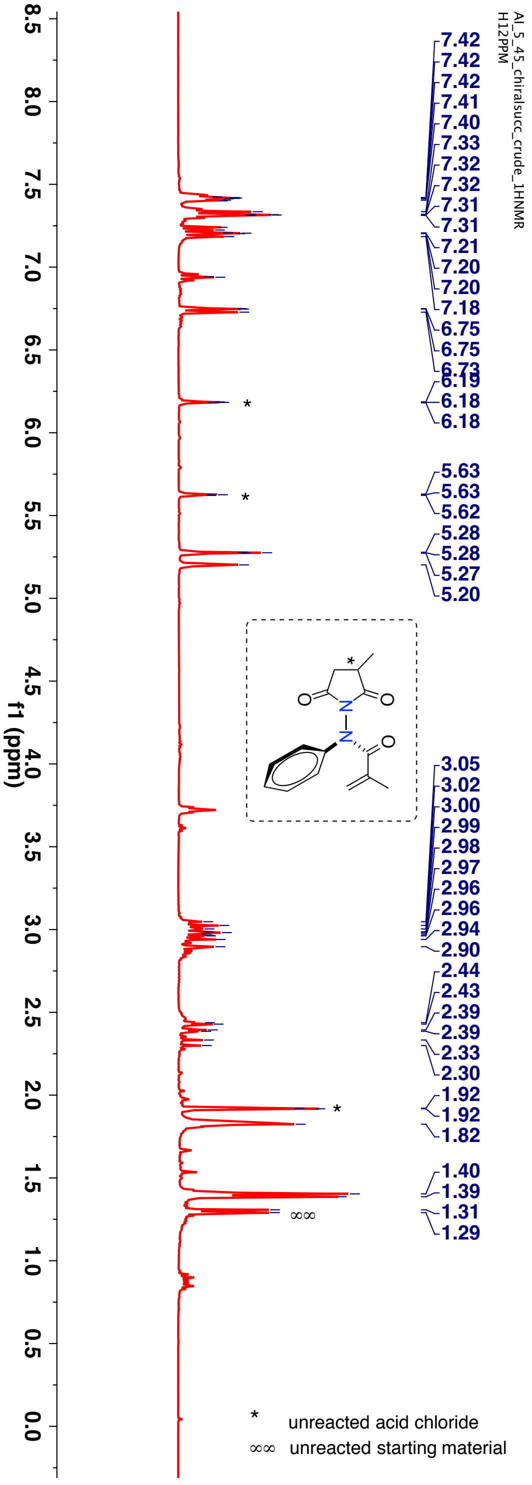

AI_5_45_chiralsucc_crude_1HNMR
H12PPM
7.42
7.42
7.42
7.41
7.40
7.33
7.32
7.32
7.31
7.31
7.21
7.20
7.20
7.18
6.75
6.75
6.73
6.19
6.18
6.18
5.63
5.63
5.62
5.28
5.28
5.27
5.20
3.05
3.02
3.00
2.99
2.98
2.97
2.96
2.96
2.94
2.90
2.44
2.43
2.39
2.39
2.33
2.30
1.92
1.92
1.82
1.40
1.39
1.31
1.29
f1 (ppm)
* unreacted acid chloride
∞∞ unreacted starting material

^{1}H-NMR (400 MHz, $CDCl_3$, δ ppm, rotamer peaks are reported together): 1.29 (d, *J* 6.8 Hz), 1.39-1.43 (m), 1.83 (bs), 2.28-2.45 (m), 2.84-3.05 (m), 5.19 (s), 5.27 (m), 7.29-7.35 (m) and 7.40-7.43 (m).

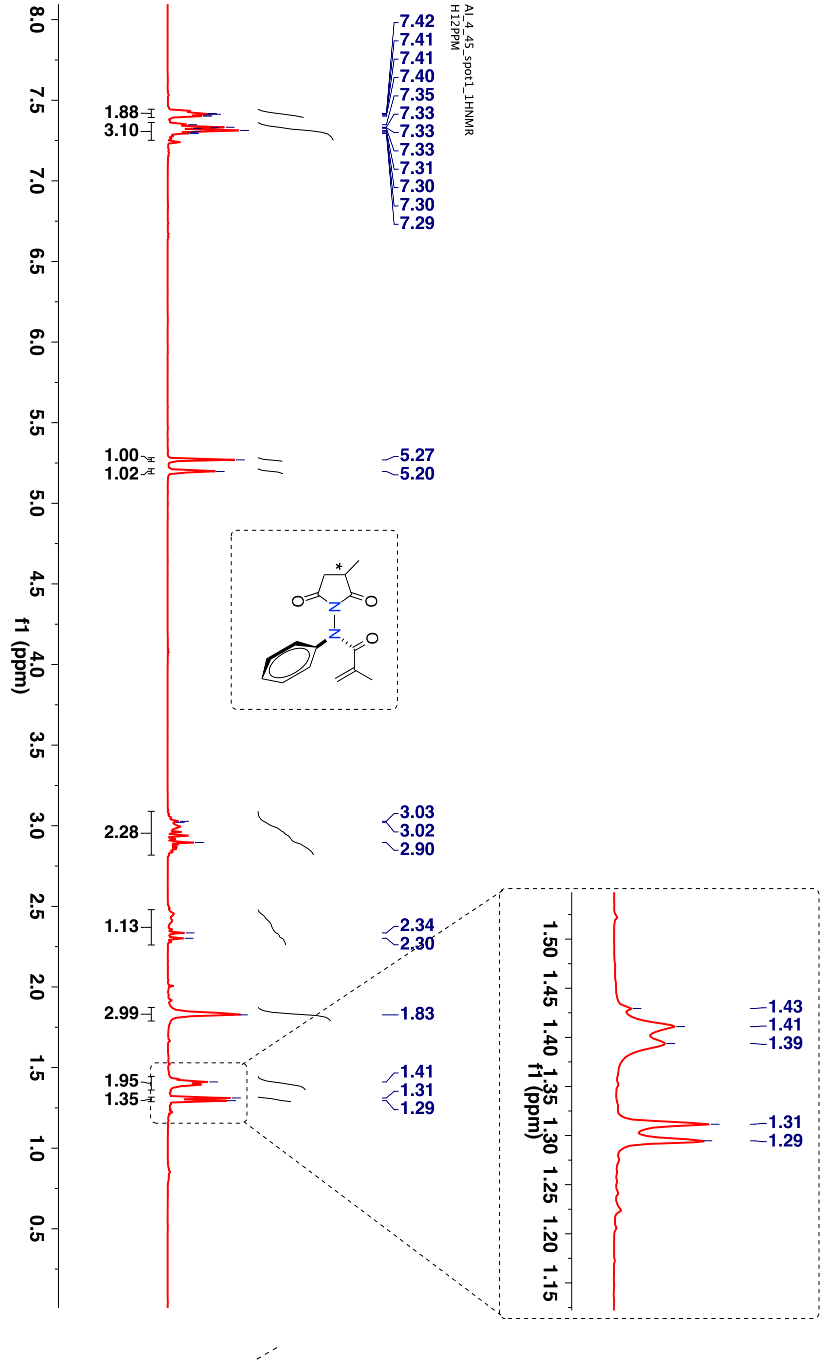

Parameters		
	Parameter	Value (f2, f1)
1	Origin	Bruker BioSpin GmbH
2	Solvent	CDCl3
3	Temperature	298.2
4	Experiment	COSY
5	Probe	5 mm PABBO BB/ 19F-1H/ D Z-GRD Z108618/ 0645
6	Number of Scans	4
7	Receiver Gain	197
8	Relaxation Delay	2.0000
9	Pulse Width	14.7000
10	Acquisition Date	2015-07-29T12:52:02
11	Spectrometer Frequency	(400.13, 400.13)
12	Spectral Width	(5341.9, 5341.9)
13	Lowest Frequency	(-265.3, -265.3)
14	Nucleus	(1H, 1H)
15	Acquired Size	(1024, 128)
16	Spectral Size	(1024, 1024)

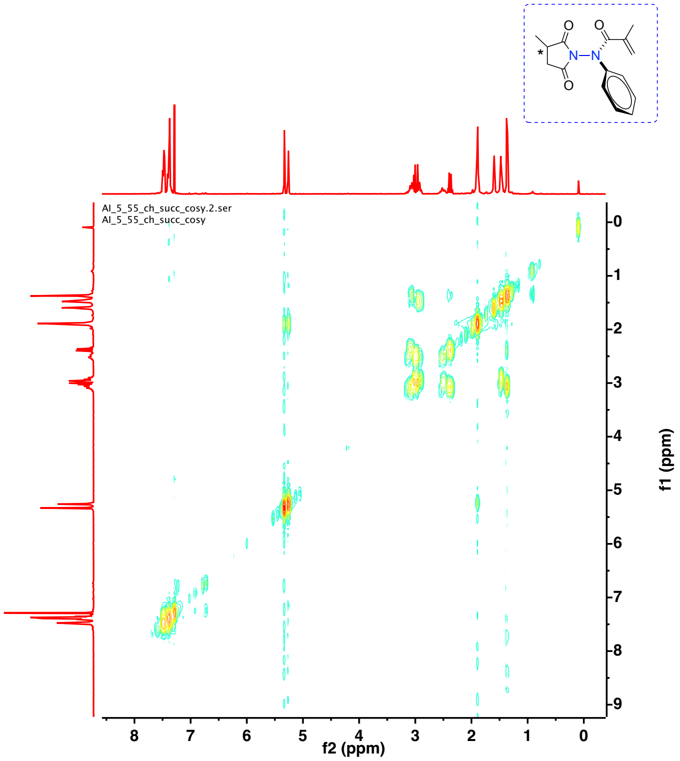

Parameters		
	Parameter	Value (f2, f1)
1	Origin	Bruker BioSpin GmbH
2	Solvent	CDCl3
3	Temperature	298.2
4	Experiment	COSY
5	Probe	5 mm PABBO BB/ 19F-1H/ D Z-GRD Z108618/ 0645
6	Number of Scans	4
7	Receiver Gain	197
8	Relaxation Delay	2.0000
9	Pulse Width	14.7000
10	Acquisition Date	2015-07-29T12:52:02
11	Spectrometer Frequency	(400.13, 400.13)
12	Spectral Width	(5341.9, 5341.9)
13	Lowest Frequency	(-265.3, -265.3)
14	Nucleus	(1H, 1H)
15	Acquired Size	(1024, 128)
16	Spectral Size	(1024, 1024)

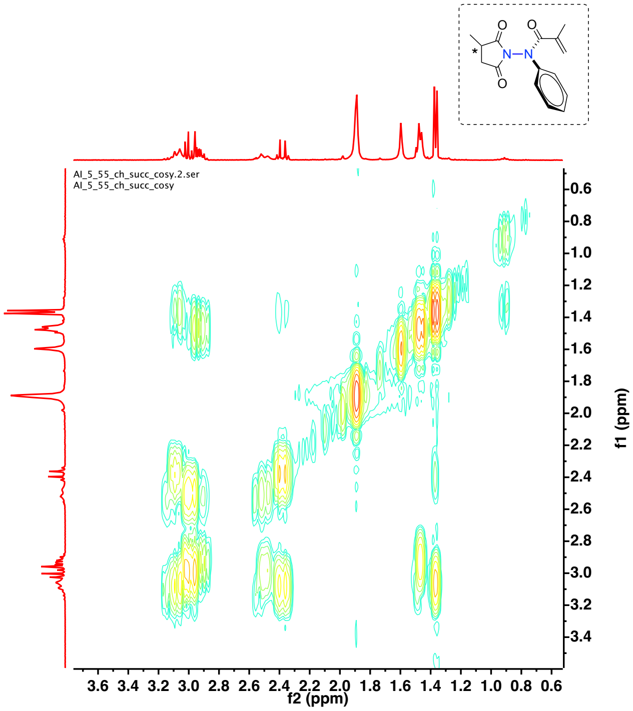

Figure S1: COSY (400 MHz, $CDCl_3$, δ ppm) spectrum of (*R*)-**2d**.

	Parameter	Value (f2, f1)
1	Origin	Bruker BioSpin GmbH
2	Solvent	CDCl3
3	Temperature	298.2
4	Experiment	NOESY
5	Probe	5 mm PABBO BB/ 19F-1H/ D Z-GRD Z108618/ 0645
6	Number of Scans	16
7	Receiver Gain	172
8	Relaxation Delay	2.0000
9	Pulse Width	14.7000
10	Acquisition Date	2015-09-09T14:24:00
11	Spectrometer Frequency	(400.13, 400.13)
12	Spectral Width	(4807.7, 4807.7)
13	Lowest Frequency	(67.0, 67.0)
14	Nucleus	(1H, 1H)
15	Acquired Size	(1024, 256)
16	Spectral Size	(1024, 1024)

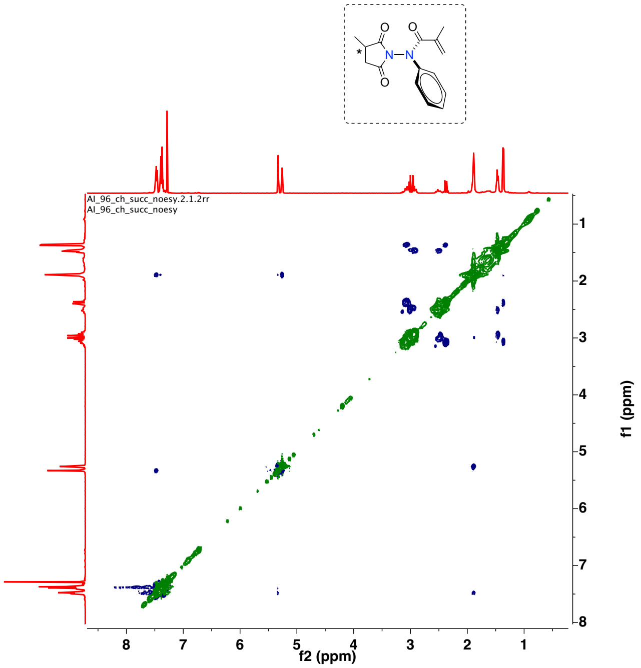

	Parameter	Value (f2, f1)
1	Origin	Bruker BioSpin GmbH
2	Solvent	CDCl3
3	Temperature	298.2
4	Experiment	NOESY
5	Probe	5 mm PABBO BB/ 19F-1H/ D Z-GRD Z108618/ 0645
6	Number of Scans	16
7	Receiver Gain	172
8	Relaxation Delay	2.0000
9	Pulse Width	14.7000
10	Acquisition Date	2015-09-09T14:24:00
11	Spectrometer Frequency	(400.13, 400.13)
12	Spectral Width	(4807.7, 4807.7)
13	Lowest Frequency	(67.0, 67.0)
14	Nucleus	(1H, 1H)
15	Acquired Size	(1024, 256)
16	Spectral Size	(1024, 1024)

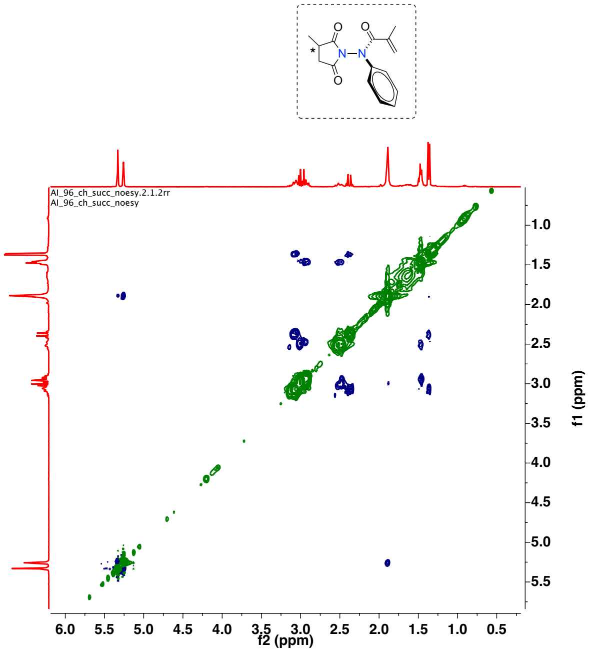

Figure S2: NOESY (400 MHz, $CDCl_3$, δ ppm) spectrum of (*R*)-**2d**.

^{13}C-NMR (100 MHz, $CDCl_3$, δ ppm, rotamer peaks are reported together): 21.7, 22.0, 22.1, 38.4, 38.5, 39.9, 40.0, 41.9, 131.6, 132.7, 132.9, 133.8, 133.9, 133.9, 134.3, 134.5, 134.6, 177.5, 177.7, 181.6 and 181.7.

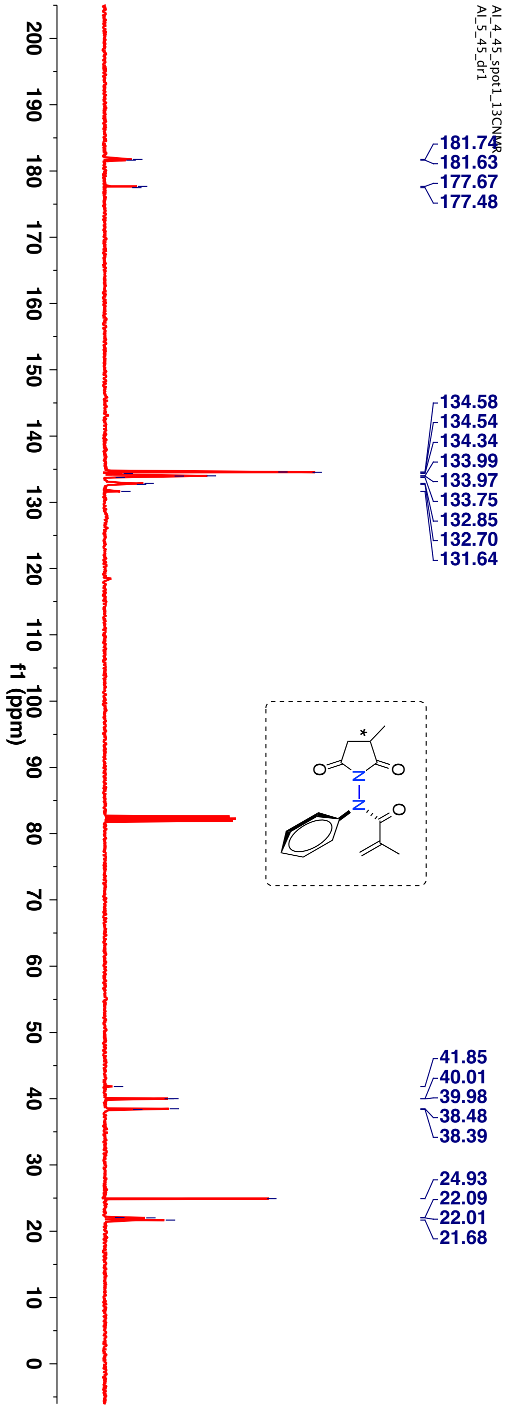

HRMS-ESI (m/z) ([M + Na]):

Chemical Formula: $C_{16}H_{20}N_2O_3$

Calculated : 295.1059

Observed : 295.1073

|Δm| : 4.7 ppm

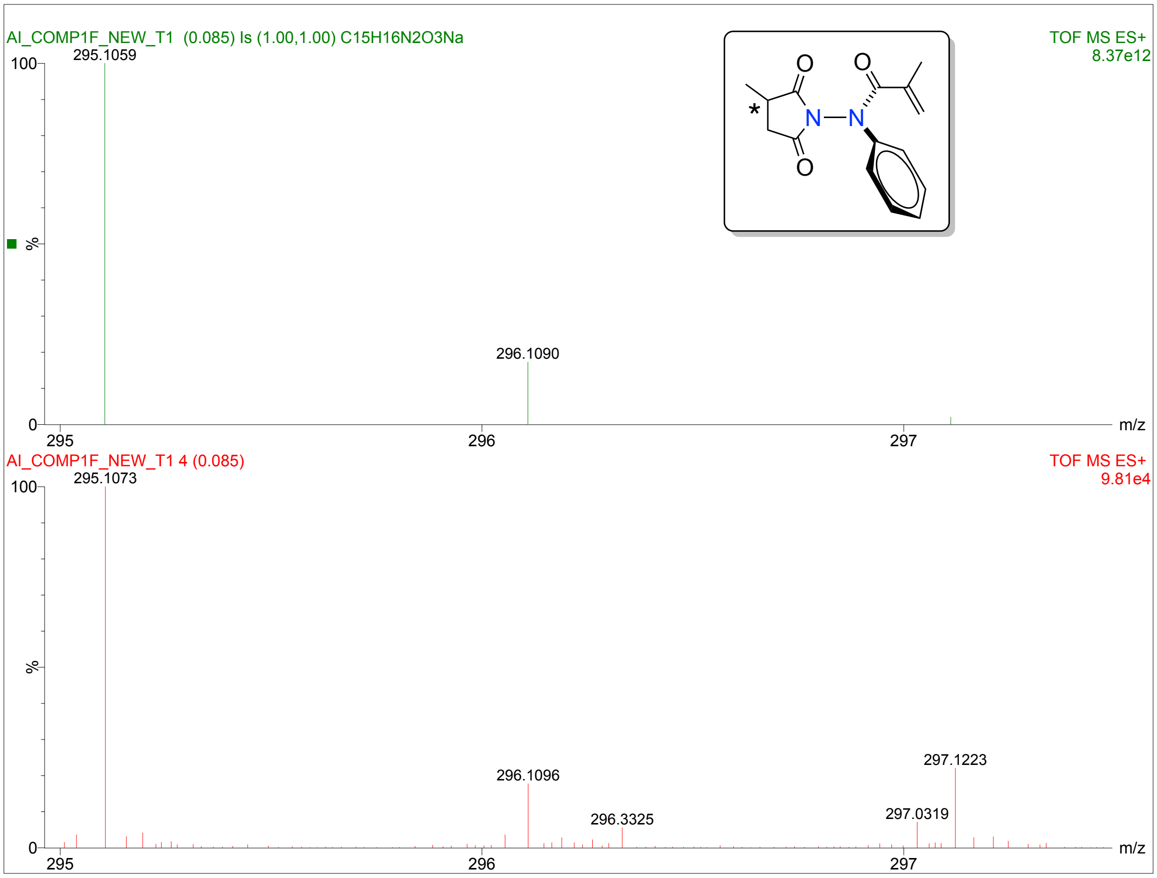

4. General irradiation procedures and characterization of photoproduct(s)

4.1. Procedure for photoreaction

a) Sensitized irradiation

In a pyrex tube, solution of the desired substrate was dissolved in an appropriate solvent. This was followed by addition of thioxanthone and then the solution was deoxygenated by purging N_2. The sample was then irradiated at ambient temperature in a Rayonet reactor equipped with ~420 nm tubes (16 tubes x 14 Watt).

b) Direct irradiation

In a pyrex tube, solution of the desired substrate was dissolved in an appropriate solvent. The solution was then deoxygenated by purging N_2. The sample was then irradiated at ambient temperature in a Rayonet reactor equipped with ~300 nm tubes (16 tubes x 12 Watt).

5. Photoreaction of hydrazide derivative (*R*)-2d in solution

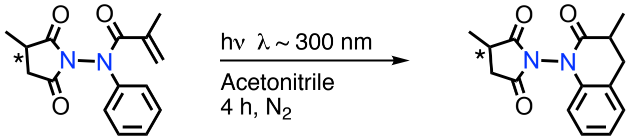

(*R*)-**2d** (*R*)-**4d**

Scheme S4: 6π Photocyclization of hydrazide derivative (*R*)-**2d** by direct irradiation.

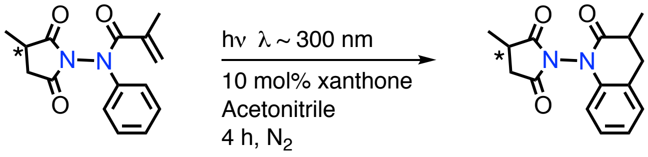

(*R*)-**2d** (*R*)-**4d**

Scheme S5: 6π Photocyclization of hydrazide derivative (*R*)-**2d** under the conditions of sensitized irradiation.

5.1. Characterization of photoproduct (*R*)-**4d**

TLC condition - R_f = 0.4 (50% hexanes:ethyl acetate). Clear solid (Yield = 75%).

^{1}H-NMR (400 MHz, $CDCl_3$, δ ppm): 1.31-1.33 (m), 1.46-1.52 (m), 2.52-2.61(m), 2.90-3.18 (m), 6.53-6.61 (m), 7.01-7.05 (m) and 7.14-7.20 (m).

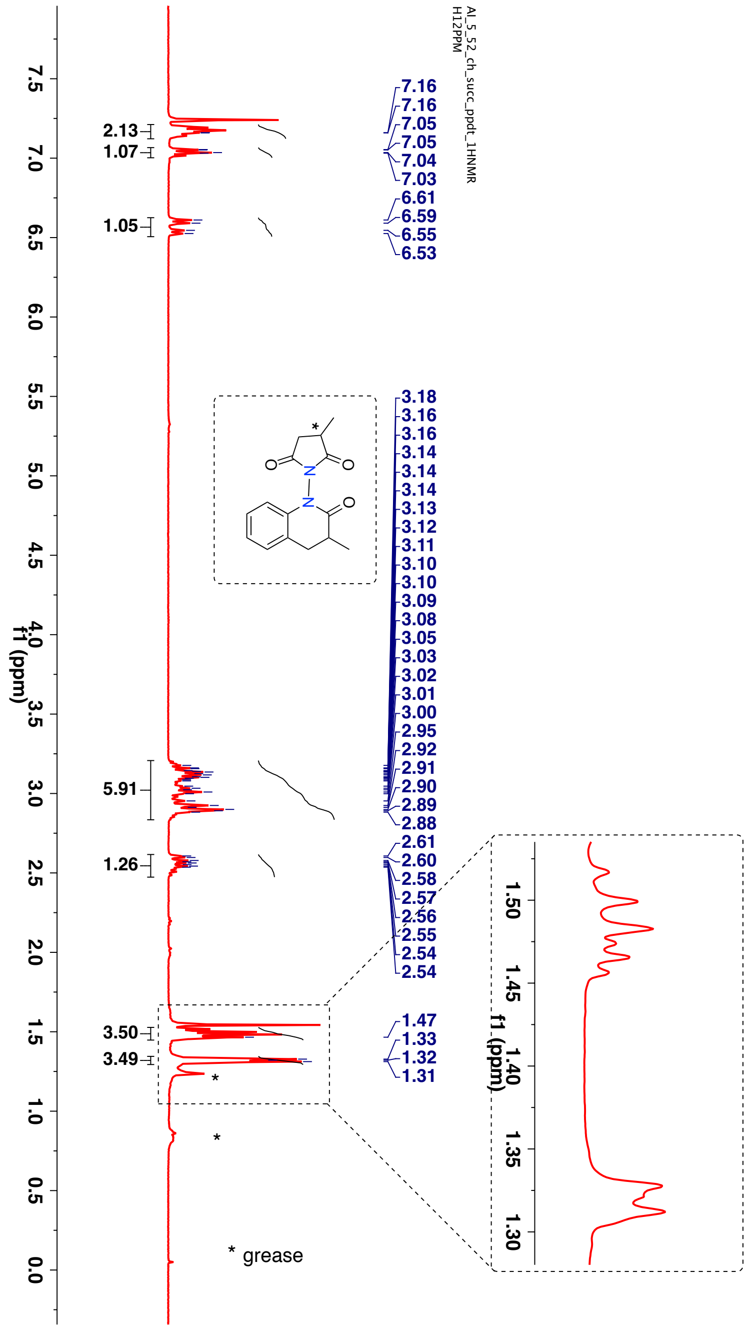

^{13}C-NMR (100 MHz, $CDCl_3$, δ ppm): 15.4, 15.4, 17.1, 17.3, 17.4, 17.4, 33.1, 33.2, 33.6, 33.7, 33.8, 35.1, 35.2, 35.2, 35.3, 35.7, 35.7, 112.6, 112.7, 112.9, 112.9, 124.4, 124.7, 124.7, 127.9, 127.9, 127.9, 128.5, 128.5, 128.6, 138.1, 169.6, 169.6, 172.1, 172.7, 175.9, 176.2 and 176.8.

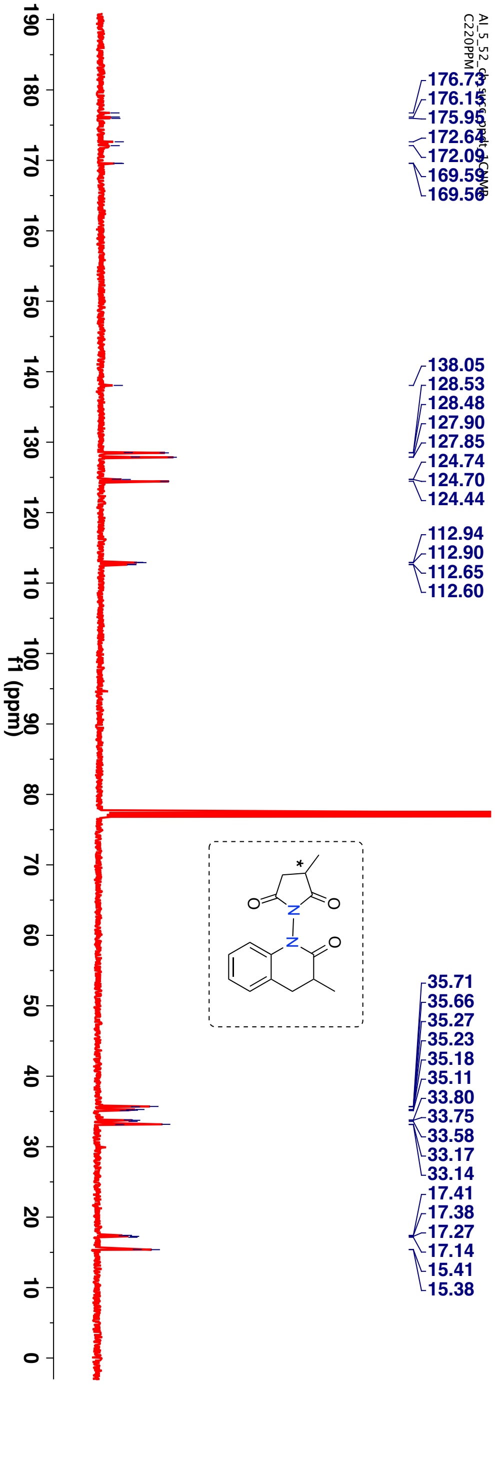

6. HPLC analysis for hydrazides

6.1. HPLC analysis for hydrazide derivative (*R*)-**6**

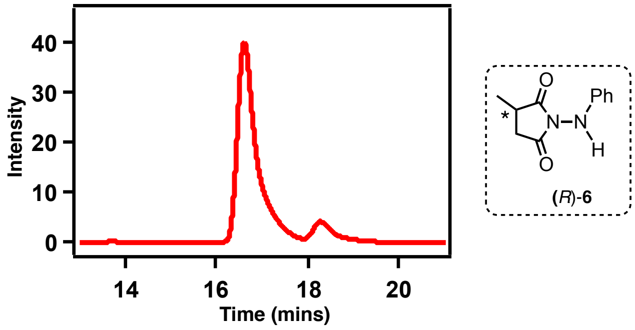

For analytical conditions,

Column	: CHIRALPAK® AD-H
Abs. detector wavelength	: 254 nm and 270 nm
Mobile phase	: Hexanes:2-propanol = 80:20
Flow rate	: 0.8 mL/min
Retention times (min)	: pA 16.6 and pB 18.3
Relative area	: pA ~ 94% and pB ~ 6%

6.2. HPLC analysis for hydrazide derivative (*R*)-**2d**

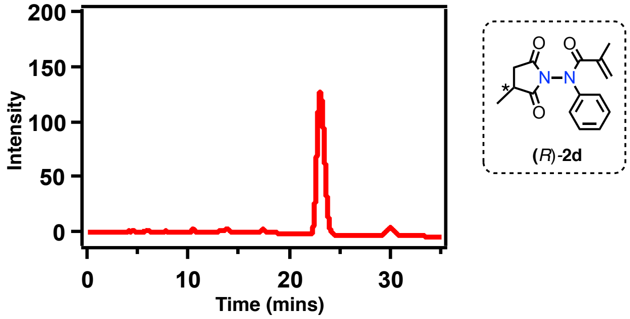

Column	: CHIRALPAK® IC
Abs. detector wavelength	: 254 nm and 270 nm
Mobile phase	: Hexanes:2-propanol = 70:30
Flow rate	: 0.8 mL/min
Retention times (min)	: pA ~23.0

6.3. HPLC analysis conditions for hydrazide derivative (*R*)-**4d** by direct irradiation (crude sample was injected)

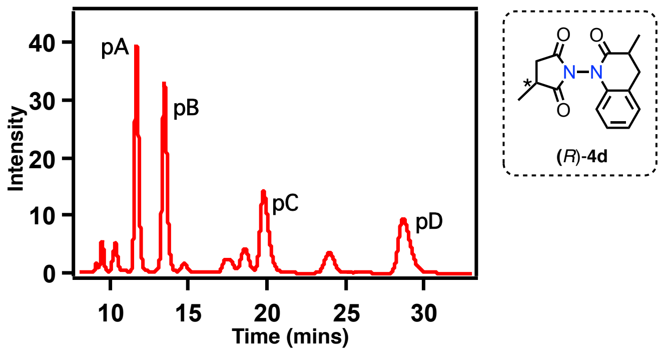

For analytical conditions,

Column	: CHIRALPAK® IC
Abs. detector wavelength	: 254 nm and 270 nm
Mobile phase	: Hexanes:2-propanol = 70:30
Flow rate	: 0.8 mL/min
Retention times (min)	: pA 11.6, pB 13.4, pC 19.7 and 28.6
Relative area	: pA ~ 29%, pB ~ 29%, pC = 21%, pD ~ 21%

6.4. HPLC analysis conditions for hydrazide derivative (*R*)-**4d** by sensitized irradiation (crude sample was injected)

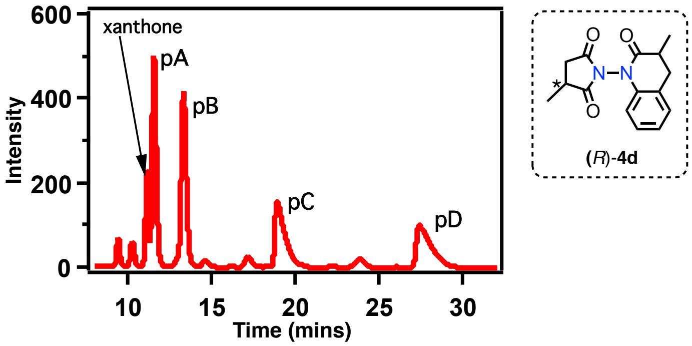

Column	: CHIRALPAK® IC
Abs. detector wavelength	: 254 nm and 270 nm
Mobile phase	: Hexanes:2-propanol = 70:30
Flow rate	: 0.8 mL/min
Retention times (min)	: pA 11.6, pB 13.4, pC 19.7 and 28.6
Relative area	: pA ~ 29%, pB ~ 29%, pC ~ 21%, pD ~ 21%

7. Racemization kinetics of hydrazide derivative 2c

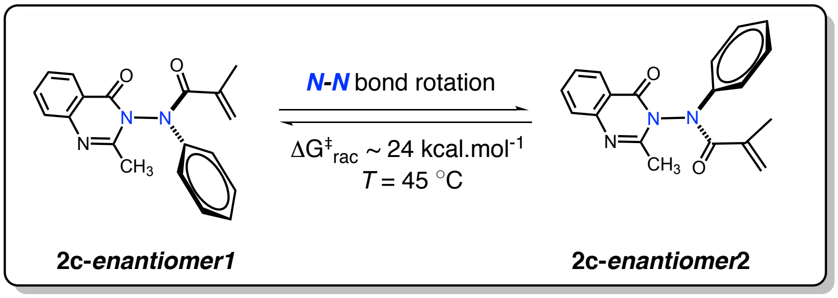

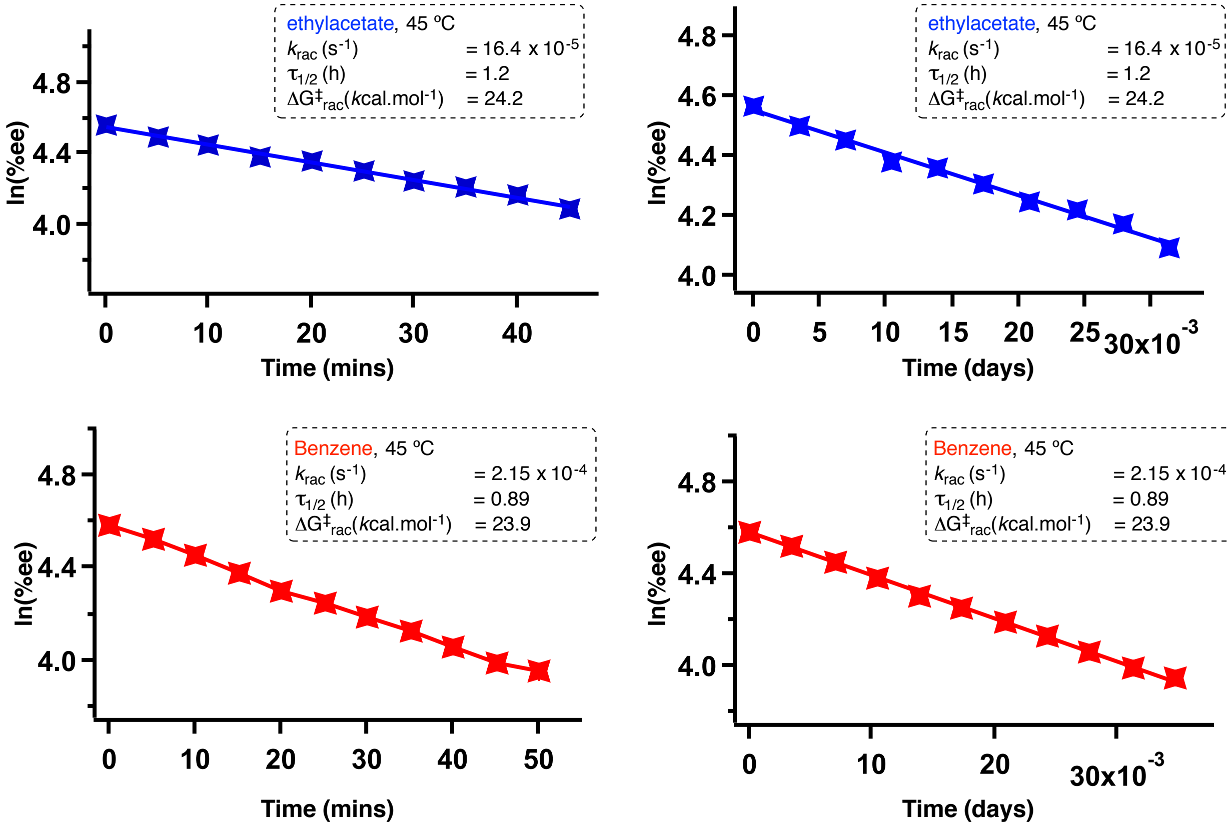

Figure S3: Racemization kinetics for quinazolinone based *N-N* atropisomer **2c** in benzene and ethyl acetate.

8. References

[1] a) A. Iyer, S. Jockusch, J. Sivaguru, *Chem Commun* **2017**, *53(10)*, 1692-1695; b) A. Iyer, S. Ahuja, S. Jockusch, A. Ugrinov, J. Sivaguru, *Chem Commun* **2018**, *54(78)*, 1021-11024; c) A. Iyer, North Dakota State Univeristy **2016**.

[2] K. Kamiński, J. Obniska, *Bioorganic & Medicinal Chemistry* **2008**, *16*, 4921-4931.

```
data_cu_axi20_0m
_audit_creation_date              2015-09-09
_audit_creation_method
;
Olex2 1.2
(compiled 2015.01.26 svn.r3150 for OlexSys, GUI svn.r4998)
;
_publ_contact_author_address      ?
_publ_contact_author_email        ?
_publ_contact_author_name         ''
_publ_contact_author_phone        ?
_publ_section_references
;
Dolomanov, O.V., Bourhis, L.J., Gildea, R.J, Howard, J.A.K. &
Puschmann, H.
 (2009), J. Appl. Cryst. 42, 339-341.

Sheldrick, G.M. (2008). Acta Cryst. A64, 112-122.
;
_chemical_name_common             ?
_chemical_name_systematic         ?
_chemical_formula_moiety          'C15 H16 N2 O3'
_chemical_formula_sum             'C15 H16 N2 O3'
_chemical_formula_weight          272.30
_chemical_absolute_configuration  ad
_chemical_melting_point           ?
loop_
  _atom_type_symbol
  _atom_type_description
  _atom_type_scat_dispersion_real
  _atom_type_scat_dispersion_imag
  _atom_type_scat_source
 'C' 'C' 0.0181 0.0091 'International Tables Vol C Tables 4.2.6.8 and
6.1.1.4'
 'H' 'H' 0.0000 0.0000 'International Tables Vol C Tables 4.2.6.8 and
6.1.1.4'
 'N' 'N' 0.0311 0.0180 'International Tables Vol C Tables 4.2.6.8 and
6.1.1.4'
 'O' 'O' 0.0492 0.0322 'International Tables Vol C Tables 4.2.6.8 and
6.1.1.4'

_shelx_space_group_comment
;
The symmetry employed for this shelxl refinement is uniquely defined
by the following loop, which should always be used as a source of
symmetry information in preference to the above space-group names.
They are only intended as comments.
;
_space_group_crystal_system       'monoclinic'
_space_group_IT_number            4
```

```
_space_group_name_H-M_alt         'P 1 21 1'
_space_group_name_Hall            'P 2yb'
loop_
  _space_group_symop_operation_xyz
 'x, y, z'
 '-x, y+1/2, -z'

_cell_length_a                    8.8088(4)
_cell_length_b                    23.6650(11)
_cell_length_c                    14.1477(7)
_cell_angle_alpha                 90
_cell_angle_beta                  104.096(2)
_cell_angle_gamma                 90
_cell_volume                      2860.4(2)
_cell_formula_units_Z             8
_cell_measurement_reflns_used     9595
_cell_measurement_temperature     100(2)
_cell_measurement_theta_max       66.527
_cell_measurement_theta_min       3.724
_shelx_estimated_absorpt_T_max    0.899
_shelx_estimated_absorpt_T_min    0.844
_exptl_absorpt_coefficient_mu     0.732
_exptl_absorpt_correction_T_max   0.7528
_exptl_absorpt_correction_T_min   0.6701
_exptl_absorpt_correction_type    multi-scan
_exptl_absorpt_process_details
;
SADABS-2014/2 (Bruker,2014/2) was used for absorption correction.
wR2(int) was 0.1064 before and 0.0701 after correction.
The Ratio of minimum to maximum transmission is 0.8901.
The \l/2 correction factor is 0.00150.
;
_exptl_absorpt_special_details    ?
_exptl_crystal_colour             colourless
_exptl_crystal_colour_primary     colourless
_exptl_crystal_density_diffrn     1.265
_exptl_crystal_density_meas       ?
_exptl_crystal_density_method     ?
_exptl_crystal_description        'chunk'
_exptl_crystal_F_000              1152
_exptl_crystal_size_max           0.24
_exptl_crystal_size_mid           0.174
_exptl_crystal_size_min           0.148
_exptl_transmission_factor_max    ?
_exptl_transmission_factor_min    ?
_diffrn_reflns_av_R_equivalents   0.0339
_diffrn_reflns_av_unetI/netI      0.0638
_diffrn_reflns_Laue_measured_fraction_full 0.974
_diffrn_reflns_Laue_measured_fraction_max 0.974
_diffrn_reflns_limit_h_max        10
```

```
_diffrn_reflns_limit_h_min        -10
_diffrn_reflns_limit_k_max        27
_diffrn_reflns_limit_k_min        -28
_diffrn_reflns_limit_l_max        16
_diffrn_reflns_limit_l_min        -16
_diffrn_reflns_number             17719
_diffrn_reflns_point_group_measured_fraction_full 0.871
_diffrn_reflns_point_group_measured_fraction_max 0.870
_diffrn_reflns_theta_full         66.779
_diffrn_reflns_theta_max          66.779
_diffrn_reflns_theta_min          3.723
_diffrn_ambient_temperature       100.01
_diffrn_detector_area_resol_mean  ?
_diffrn_measured_fraction_theta_full 0.974
_diffrn_measured_fraction_theta_max 0.974
_diffrn_measurement_device_type   'Bruker APEX-II CCD'
_diffrn_measurement_method        '\f and \w scans'
_diffrn_radiation_type            CuK\a
_diffrn_radiation_wavelength      1.54178
_diffrn_source                    ?
_diffrn_standards_number          0
_reflns_Friedel_coverage          0.741
_reflns_Friedel_fraction_full     0.761
_reflns_Friedel_fraction_max      0.761
_reflns_number_gt                 7549
_reflns_number_total              8826
_reflns_special_details
;
 Reflections were merged by SHELXL according to the crystal
 class for the calculation of statistics and refinement.

 _reflns_Friedel_fraction is defined as the number of unique
 Friedel pairs measured divided by the number that would be
 possible theoretically, ignoring centric projections and
 systematic absences.
;
_reflns_threshold_expression      'I > 2\s(I)'
_computing_cell_refinement        'SAINT v8.34A (Bruker, 2013)'
_computing_data_collection        ?
_computing_data_reduction         'SAINT v8.34A (Bruker, 2013)'
_computing_molecular_graphics     'Olex2 (Dolomanov et al., 2009)'
_computing_publication_material   'Olex2 (Dolomanov et al., 2009)'
_computing_structure_refinement   'XL (Sheldrick, 2008)'
_computing_structure_solution     ?
_refine_diff_density_max          0.503
_refine_diff_density_min          -0.294
_refine_diff_density_rms          0.052
_refine_ls_abs_structure_details
;
 Flack x determined using 2667 quotients [(I+)-(I-)]/[(I+)+(I-)]
```

```
 (Parsons, Flack and Wagner, Acta Cryst. B69 (2013) 249-259).
;
_refine_ls_abs_structure_Flack    -0.1(3)
_refine_ls_extinction_coef        .
_refine_ls_extinction_method      none
_refine_ls_goodness_of_fit_ref    1.041
_refine_ls_hydrogen_treatment     constr
_refine_ls_matrix_type            full
_refine_ls_number_parameters      749
_refine_ls_number_reflns          8826
_refine_ls_number_restraints      1
_refine_ls_R_factor_all           0.0700
_refine_ls_R_factor_gt            0.0610
_refine_ls_restrained_S_all       1.041
_refine_ls_shift/su_max           0.000
_refine_ls_shift/su_mean          0.000
_refine_ls_structure_factor_coef  Fsqd
_refine_ls_weighting_details
'w=1/[\s^2^(Fo^2^)+(0.0730P)^2^+1.9154P] where P=(Fo^2^+2Fc^2^)/3'
_refine_ls_weighting_scheme       calc
_refine_ls_wR_factor_gt           0.1597
_refine_ls_wR_factor_ref          0.1668
_refine_special_details           ?
_olex2_refinement_description
;
1. Fixed Uiso
 At 1.2 times of:
  All C(H) groups, {H12D,H12A,H12B} of C12D, All C(H,H) groups
 At 1.5 times of:
  {H1CA,H1CB,H1CC} of C1C, {H16A,H16B,H16C} of C16D, {H1BA,H1BB,H1BC}
of C1B,
  {H1DA,H1DB,H1DC} of C1D, {H15G,H15H,H15I} of C15A, {H1AA,H1AB,H1AC}
of C1A,
  {H15D,H15E,H15F} of C15B, {H15A,H15B,H15C} of C15D, {H15J,H15K,H15L}
of C15C
2. Others

Sof(H12D)=Sof(C13D)=Sof(H13A)=Sof(H13B)=Sof(C15D)=Sof(H15A)=Sof(H15B)=
 Sof(H15C)=1-FVAR(1)

Sof(H12A)=Sof(H12B)=Sof(C16D)=Sof(H16A)=Sof(H16B)=Sof(H16C)=Sof(C17D)=
 Sof(H17D)=FVAR(1)
3.a Ternary CH refined with riding coordinates:
 C12D(H12D), C17D(H17D), C13B(H13C), C13A(H13D), C13C(H13E)
3.b Secondary CH2 refined with riding coordinates:
 C12D(H12A,H12B), C13D(H13A,H13B), C12B(H12C,H12E), C12A(H12F,H12G),
C12C(H12H,
 H12I)
3.c Aromatic/amide H refined with riding coordinates:
 C6D(H6D), C7D(H7D), C8D(H8D), C9D(H9D), C10D(H10D), C6B(H6B),
```

```
C7B(H7B),
 C8B(H8B), C9B(H9B), C10B(H10B), C6A(H6A), C7A(H7A), C8A(H8A),
C9A(H9A),
 C10A(H10A), C6C(H6C), C7C(H7C), C8C(H8C), C9C(H9C), C10C(H10C)
3.d X=CH2 refined with riding coordinates:
 C3D(H3DA,H3DB), C3B(H3BA,H3BB), C3A(H3AA,H3AB), C3C(H3CA,H3CB)
3.e Idealised Me refined as rotating group:
 C1D(H1DA,H1DB,H1DC), C15D(H15A,H15B,H15C), C16D(H16A,H16B,H16C),
C1B(H1BA,
 H1BB,H1BC), C15B(H15D,H15E,H15F), C1A(H1AA,H1AB,H1AC),
C15A(H15G,H15H,H15I),
 C1C(H1CA,H1CB,H1CC), C15C(H15J,H15K,H15L)
;
_atom_sites_solution_hydrogens    geom
_atom_sites_solution_primary      ?
_atom_sites_solution_secondary    ?
loop_
  _atom_site_label
  _atom_site_type_symbol
  _atom_site_fract_x
  _atom_site_fract_y
  _atom_site_fract_z
  _atom_site_U_iso_or_equiv
  _atom_site_adp_type
  _atom_site_occupancy
  _atom_site_site_symmetry_order
  _atom_site_calc_flag
  _atom_site_refinement_flags_posn
  _atom_site_refinement_flags_adp
  _atom_site_refinement_flags_occupancy
  _atom_site_disorder_assembly
  _atom_site_disorder_group
 C1D C 2.7361(9) 1.7499(3) 1.0756(5) 0.0486(16) Uani 1 1 d . . . . .
 H1DA H 2.6577 1.7519 1.0133 0.073 Uiso 1 1 calc GR . . . .
 H1DB H 2.8385 1.7609 1.0661 0.073 Uiso 1 1 calc GR . . . .
 H1DC H 2.7066 1.7757 1.1224 0.073 Uiso 1 1 calc GR . . . .
 C2D C 2.7442(6) 1.6905(2) 1.1142(4) 0.0296(11) Uani 1 1 d . . . . .
 C3D C 2.6659(7) 1.6469(3) 1.0646(4) 0.0361(13) Uani 1 1 d . . . . .
 H3DA H 2.6819 1.6098 1.0907 0.043 Uiso 1 1 calc R . . . .
 H3DB H 2.5944 1.6531 1.0034 0.043 Uiso 1 1 calc R . . . .
 C4D C 2.8566(6) 1.6819(2) 1.2100(4) 0.0279(11) Uani 1 1 d . . . . .
 C5D C 2.6442(6) 1.6388(2) 1.2799(3) 0.0256(11) Uani 1 1 d . . . . .
 C6D C 2.5318(6) 1.6789(3) 1.2851(4) 0.0335(12) Uani 1 1 d . . . . .
 H6D H 2.5568 1.7180 1.2904 0.040 Uiso 1 1 calc R . . . .
 C7D C 2.3816(7) 1.6602(3) 1.2822(4) 0.0428(15) Uani 1 1 d . . . . .
 H7D H 2.3025 1.6868 1.2858 0.051 Uiso 1 1 calc R . . . .
 C8D C 2.3455(7) 1.6033(3) 1.2740(4) 0.0470(17) Uani 1 1 d . . . . .
 H8D H 2.2416 1.5910 1.2706 0.056 Uiso 1 1 calc R . . . .
 C9D C 2.4600(7) 1.5644(3) 1.2709(5) 0.0430(15) Uani 1 1 d . . . . .
 H9D H 2.4359 1.5252 1.2677 0.052 Uiso 1 1 calc R . . . .
```

C10D C 2.6103(7) 1.5820(3) 1.2725(4) 0.0332(12) Uani 1 1 d . . . . .
H10D H 2.6889 1.5553 1.2685 0.040 Uiso 1 1 calc R . . . .
C11D C 2.9535(7) 1.6895(3) 1.4414(4) 0.0398(14) Uani 1 1 d . . . . .
C12D C 3.0865(7) 1.6635(3) 1.5187(4) 0.0491(17) Uani 1 1 d . . . . .
H12D H 3.0566 1.6663 1.5823 0.059 Uiso 0.452(17) 1 calc R . P A 2
H12A H 3.0473 1.6483 1.5735 0.059 Uiso 0.548(17) 1 calc R . P A 1
H12B H 3.1688 1.6919 1.5443 0.059 Uiso 0.548(17) 1 calc R . P A 1
C13D C 3.103(2) 1.6047(10) 1.4997(15) 0.061(6) Uani 0.452(17) 1 d . .
P A 2
H13A H 3.0524 1.5807 1.5406 0.074 Uiso 0.452(17) 1 calc R . P A 2
H13B H 3.2146 1.5938 1.5107 0.074 Uiso 0.452(17) 1 calc R . P A 2
C14D C 3.0121(7) 1.6005(4) 1.3844(6) 0.059(2) Uani 1 1 d . . . . .
C15D C 3.2279(19) 1.6955(8) 1.5282(12) 0.064(5) Uani 0.452(17) 1
d . . P A 2
H15A H 3.2530 1.6982 1.4645 0.097 Uiso 0.452(17) 1 calc GR . P A 2
H15B H 3.3139 1.6766 1.5744 0.097 Uiso 0.452(17) 1 calc GR . P A 2
H15C H 3.2134 1.7335 1.5520 0.097 Uiso 0.452(17) 1 calc GR . P A 2
C16D C 3.1995(17) 1.5618(6) 1.5239(10) 0.066(5) Uani 0.548(17) 1
d . . P A 1
H16A H 3.2902 1.5701 1.5778 0.099 Uiso 0.548(17) 1 calc GR . P A 1
H16B H 3.2270 1.5322 1.4826 0.099 Uiso 0.548(17) 1 calc GR . P A 1
H16C H 3.1123 1.5489 1.5502 0.099 Uiso 0.548(17) 1 calc GR . P A 1
C17D C 3.1519(14) 1.6145(6) 1.4642(9) 0.038(3) Uani 0.548(17) 1 d . .
P A 1
H17D H 3.2396 1.6285 1.4369 0.046 Uiso 0.548(17) 1 calc R . P A 1
N1D N 2.8019(5) 1.6566(2) 1.2823(3) 0.0307(10) Uani 1 1 d . . . . .
N2D N 2.9119(5) 1.6475(2) 1.3701(3) 0.0338(11) Uani 1 1 d . . . . .
O1D O 2.9941(4) 1.69657(18) 1.2242(3) 0.0381(9) Uani 1 1 d . . . . .
O2D O 3.0003(5) 1.5614(2) 1.3287(5) 0.0704(17) Uani 1 1 d . . . . .
O3D O 2.8875(6) 1.7336(2) 1.4375(4) 0.0526(12) Uani 1 1 d . . . . .
C1B C 3.2275(8) 1.7749(3) 1.0843(4) 0.0400(14) Uani 1 1 d . . . . .
H1BA H 3.1617 1.7742 1.0176 0.060 Uiso 1 1 calc GR . . . .
H1BB H 3.1844 1.7489 1.1250 0.060 Uiso 1 1 calc GR . . . .
H1BC H 3.3341 1.7632 1.0840 0.060 Uiso 1 1 calc GR . . . .
C2B C 3.2308(6) 1.8335(2) 1.1244(4) 0.0310(12) Uani 1 1 d . . . . .
C3B C 3.1518(7) 1.8768(3) 1.0777(4) 0.0350(13) Uani 1 1 d . . . . .
H3BA H 3.0872 1.8719 1.0138 0.042 Uiso 1 1 calc R . . . .
H3BB H 3.1597 1.9129 1.1079 0.042 Uiso 1 1 calc R . . . .
C4B C 3.3450(6) 1.8397(3) 1.2216(4) 0.0334(12) Uani 1 1 d . . . . .
C5B C 3.1302(6) 1.8799(3) 1.2922(4) 0.0318(12) Uani 1 1 d . . . . .
C6B C 3.0207(7) 1.8389(3) 1.2973(4) 0.0369(13) Uani 1 1 d . . . . .
H6B H 3.0500 1.8002 1.3041 0.044 Uiso 1 1 calc R . . . .
C7B C 2.8676(7) 1.8549(3) 1.2925(5) 0.0450(16) Uani 1 1 d . . . . .
H7B H 2.7910 1.8271 1.2953 0.054 Uiso 1 1 calc R . . . .
C8B C 2.8267(7) 1.9112(3) 1.2838(5) 0.0442(16) Uani 1 1 d . . . . .
H8B H 2.7216 1.9219 1.2803 0.053 Uiso 1 1 calc R . . . .
C9B C 2.9363(7) 1.9521(3) 1.2800(4) 0.0380(13) Uani 1 1 d . . . . .
H9B H 2.9066 1.9907 1.2746 0.046 Uiso 1 1 calc R . . . .
C10B C 3.0921(6) 1.9368(2) 1.2841(4) 0.0318(12) Uani 1 1 d . . . . .
H10B H 3.1688 1.9646 1.2814 0.038 Uiso 1 1 calc R . . . .

C11B C 3.4287(11) 1.8183(5) 1.4447(6) 0.083(3) Uani 1 1 d . . . . .
C12B C 3.5646(14) 1.8318(6) 1.5279(8) 0.119(5) Uani 1 1 d . . . . .
H12C H 3.6567 1.8085 1.5251 0.143 Uiso 1 1 calc R . . . .
H12E H 3.5379 1.8251 1.5910 0.143 Uiso 1 1 calc R . . . .
C13B C 3.5964(10) 1.8937(7) 1.5148(8) 0.112(5) Uani 1 1 d . . . . .
H13C H 3.5621 1.9158 1.5661 0.134 Uiso 1 1 calc R . . . .
C14B C 3.4947(9) 1.9109(5) 1.4142(7) 0.071(3) Uani 1 1 d . . . . .
C15B C 3.7523(16) 1.9046(11) 1.5219(10) 0.201(10) Uani 1 1
d . . . . .
H15D H 3.7660 1.9449 1.5097 0.301 Uiso 1 1 calc GR . . . .
H15E H 3.7884 1.8820 1.4736 0.301 Uiso 1 1 calc GR . . . .
H15F H 3.8135 1.8948 1.5874 0.301 Uiso 1 1 calc GR . . . .
N1B N 3.2894(5) 1.8638(2) 1.2951(3) 0.0381(12) Uani 1 1 d . . . . .
N2B N 3.3956(6) 1.8664(3) 1.3855(4) 0.0508(15) Uani 1 1 d . . . . .
O1B O 3.4801(5) 1.82406(19) 1.2360(3) 0.0441(10) Uani 1 1 d . . . . .
O2B O 3.4936(6) 1.9529(3) 1.3697(5) 0.0776(19) Uani 1 1 d . . . . .
O3B O 3.3596(10) 1.7735(3) 1.4285(5) 0.102(3) Uani 1 1 d . . . . .
C1A C 2.6771(11) 1.5714(4) 1.5403(5) 0.074(3) Uani 1 1 d . . . . .
H1AA H 2.6247 1.5836 1.4742 0.111 Uiso 1 1 calc GR . . . .
H1AB H 2.6096 1.5448 1.5641 0.111 Uiso 1 1 calc GR . . . .
H1AC H 2.7762 1.5528 1.5394 0.111 Uiso 1 1 calc GR . . . .
C2A C 2.7088(7) 1.6218(3) 1.6063(4) 0.0444(16) Uani 1 1 d . . . . .
C3A C 2.6654(10) 1.6735(4) 1.5807(6) 0.068(2) Uani 1 1 d . . . . .
H3AA H 2.6091 1.6813 1.5158 0.082 Uiso 1 1 calc R . . . .
H3AB H 2.6901 1.7032 1.6270 0.082 Uiso 1 1 calc R . . . .
C4A C 2.8133(7) 1.6087(3) 1.7048(4) 0.0353(13) Uani 1 1 d . . . . .
C5A C 2.5925(6) 1.6340(2) 1.7811(3) 0.0270(11) Uani 1 1 d . . . . .
C6A C 2.4844(7) 1.5912(3) 1.7606(4) 0.0388(13) Uani 1 1 d . . . . .
H6A H 2.5145 1.5540 1.7474 0.047 Uiso 1 1 calc R . . . .
C7A C 2.3296(7) 1.6035(3) 1.7595(5) 0.0473(17) Uani 1 1 d . . . . .
H7A H 2.2533 1.5744 1.7451 0.057 Uiso 1 1 calc R . . . .
C8A C 2.2858(7) 1.6562(3) 1.7786(4) 0.0467(17) Uani 1 1 d . . . . .
H8A H 2.1797 1.6636 1.7781 0.056 Uiso 1 1 calc R . . . .
C9A C 2.3936(8) 1.6986(3) 1.7986(5) 0.0536(18) Uani 1 1 d . . . . .
H9A H 2.3621 1.7356 1.8118 0.064 Uiso 1 1 calc R . . . .
C10A C 2.5482(7) 1.6883(3) 1.7998(5) 0.0408(14) Uani 1 1 d . . . . .
H10A H 2.6231 1.7179 1.8133 0.049 Uiso 1 1 calc R . . . .
C11A C 2.8717(7) 1.5604(3) 1.9206(5) 0.0375(13) Uani 1 1 d . . . . .
C12A C 2.9951(8) 1.5662(3) 2.0153(5) 0.0501(17) Uani 1 1 d . . . . .
H12F H 3.0792 1.5379 2.0194 0.060 Uiso 1 1 calc R . . . .
H12G H 2.9486 1.5612 2.0719 0.060 Uiso 1 1 calc R . . . .
C13A C 3.0591(6) 1.6266(3) 2.0126(4) 0.0375(13) Uani 1 1 d . . . . .
H13D H 3.0449 1.6480 2.0708 0.045 Uiso 1 1 calc R . . . .
C14A C 2.9612(6) 1.6540(3) 1.9213(4) 0.0333(13) Uani 1 1 d . . . . .
C15A C 3.2315(7) 1.6270(3) 2.0119(5) 0.0497(15) Uani 1 1 d . . . . .
H15G H 3.2478 1.6030 1.9589 0.075 Uiso 1 1 calc GR . . . .
H15H H 3.2939 1.6127 2.0743 0.075 Uiso 1 1 calc GR . . . .
H15I H 3.2640 1.6658 2.0021 0.075 Uiso 1 1 calc GR . . . .
N1A N 2.7544(5) 1.6229(2) 1.7838(3) 0.0282(10) Uani 1 1 d . . . . .
N2A N 2.8542(5) 1.61352(19) 1.8758(3) 0.0284(10) Uani 1 1 d . . . . .

```
 O1A O 2.9446(5) 1.5887(2) 1.7166(3) 0.0435(10) Uani 1 1 d . . . . .
 O2A O 2.9723(5) 1.70026(18) 1.8903(3) 0.0422(10) Uani 1 1 d . . . . .
 O3A O 2.7970(5) 1.51970(19) 1.8865(3) 0.0427(10) Uani 1 1 d . . . . .
 C1C C 2.8403(9) 1.4418(4) 1.4486(5) 0.065(2) Uani 1 1 d . . . . .
 H1CA H 2.7411 1.4560 1.4591 0.097 Uiso 1 1 calc GR . . . .
 H1CB H 2.8937 1.4722 1.4225 0.097 Uiso 1 1 calc GR . . . .
 H1CC H 2.9068 1.4287 1.5107 0.097 Uiso 1 1 calc GR . . . .
 C2C C 2.8088(7) 1.3943(3) 1.3784(4) 0.0419(15) Uani 1 1 d . . . . .
 C3C C 2.8587(9) 1.3414(4) 1.3999(5) 0.063(2) Uani 1 1 d . . . . .
 H3CA H 2.9183 1.3325 1.4636 0.076 Uiso 1 1 calc R . . . .
 H3CB H 2.8345 1.3128 1.3515 0.076 Uiso 1 1 calc R . . . .
 C4C C 2.7043(7) 1.4075(2) 1.2816(4) 0.0332(12) Uani 1 1 d . . . . .
 C5C C 2.9236(6) 1.3864(2) 1.2010(4) 0.0277(11) Uani 1 1 d . . . . .
 C6C C 2.9707(7) 1.3337(3) 1.1797(4) 0.0385(13) Uani 1 1 d . . . . .
 H6C H 2.8984 1.3032 1.1679 0.046 Uiso 1 1 calc R . . . .
 C7C C 3.1247(7) 1.3253(3) 1.1755(5) 0.0540(18) Uani 1 1 d . . . . .
 H7C H 3.1578 1.2891 1.1595 0.065 Uiso 1 1 calc R . . . .
 C8C C 3.2292(7) 1.3692(3) 1.1943(6) 0.057(2) Uani 1 1 d . . . . .
 H8C H 3.3352 1.3630 1.1931 0.069 Uiso 1 1 calc R . . . .
 C9C C 3.1815(7) 1.4227(3) 1.2152(6) 0.0534(18) Uani 1 1 d . . . . .
 H9C H 3.2539 1.4531 1.2269 0.064 Uiso 1 1 calc R . . . .
 C10C C 3.0251(7) 1.4315(3) 1.2190(5) 0.0383(14) Uani 1 1 d . . . . .
 H10C H 2.9904 1.4677 1.2336 0.046 Uiso 1 1 calc R . . . .
 C11C C 2.5524(7) 1.3642(3) 1.0648(4) 0.0354(13) Uani 1 1 d . . . . .
 C12C C 2.4531(7) 1.3909(3) 0.9750(4) 0.0394(14) Uani 1 1 d . . . . .
 H12H H 2.3419 1.3915 0.9776 0.047 Uiso 1 1 calc R . . . .
 H12I H 2.4622 1.3700 0.9161 0.047 Uiso 1 1 calc R . . . .
 C13C C 2.5167(7) 1.4518(3) 0.9736(4) 0.0370(13) Uani 1 1 d . . . . .
 H13E H 2.5677 1.4556 0.9181 0.044 Uiso 1 1 calc R . . . .
 C14C C 2.6394(6) 1.4578(3) 1.0684(4) 0.0327(12) Uani 1 1 d . . . . .
 C15C C 2.3936(8) 1.4967(3) 0.9652(5) 0.0588(19) Uani 1 1 d . . . . .
 H15J H 2.3454 1.4938 1.0206 0.088 Uiso 1 1 calc GR . . . .
 H15K H 2.3133 1.4915 0.9044 0.088 Uiso 1 1 calc GR . . . .
 H15L H 2.4418 1.5340 0.9653 0.088 Uiso 1 1 calc GR . . . .
 N1C N 2.7621(5) 1.3957(2) 1.2023(3) 0.0293(10) Uani 1 1 d . . . . .
 N2C N 2.6604(5) 1.4050(2) 1.1120(3) 0.0288(10) Uani 1 1 d . . . . .
 O1C O 2.5732(5) 1.4260(2) 1.2735(3) 0.0441(11) Uani 1 1 d . . . . .
 O2C O 2.7117(5) 1.49948(18) 1.1029(3) 0.0397(10) Uani 1 1 d . . . . .
 O3C O 2.5492(5) 1.31684(19) 1.0952(3) 0.0522(12) Uani 1 1 d . . . . .

loop_
  _atom_site_aniso_label
  _atom_site_aniso_U_11
  _atom_site_aniso_U_22
  _atom_site_aniso_U_33
  _atom_site_aniso_U_23
  _atom_site_aniso_U_13
  _atom_site_aniso_U_12
 C1D 0.063(4) 0.034(3) 0.047(4) 0.009(3) 0.009(3) 0.011(3)
 C2D 0.026(2) 0.036(3) 0.031(3) 0.001(2) 0.014(2) 0.001(2)
```

C3D 0.037(3) 0.045(3) 0.026(3) 0.004(2) 0.008(2) -0.001(3)
C4D 0.032(3) 0.025(3) 0.029(3) -0.002(2) 0.012(2) 0.001(2)
C5D 0.021(2) 0.036(3) 0.019(2) -0.001(2) 0.0039(18) -0.003(2)
C6D 0.034(3) 0.042(3) 0.026(3) 0.006(2) 0.009(2) 0.002(3)
C7D 0.025(3) 0.070(5) 0.035(3) 0.006(3) 0.011(2) 0.005(3)
C8D 0.030(3) 0.074(5) 0.037(3) 0.000(3) 0.006(2) -0.011(3)
C9D 0.038(3) 0.053(4) 0.038(3) -0.001(3) 0.008(3) -0.015(3)
C10D 0.034(3) 0.040(3) 0.026(3) 0.001(2) 0.009(2) -0.004(3)
C11D 0.041(3) 0.050(4) 0.031(3) -0.003(3) 0.014(3) -0.008(3)
C12D 0.037(3) 0.079(5) 0.027(3) -0.005(3) -0.001(2) -0.010(3)
C13D 0.038(10) 0.104(16) 0.040(10) 0.002(10) 0.005(8) 0.011(10)
C14D 0.025(3) 0.083(6) 0.058(4) -0.004(4) -0.010(3) 0.006(3)
C15D 0.062(10) 0.073(11) 0.050(9) -0.008(8) -0.002(7) -0.015(8)
C16D 0.068(9) 0.065(9) 0.051(8) 0.002(6) -0.013(6) 0.024(7)
C17D 0.020(6) 0.068(8) 0.024(6) -0.002(5) 0.000(4) 0.005(5)
N1D 0.022(2) 0.045(3) 0.023(2) -0.0017(19) 0.0033(17) -0.0045(19)
N2D 0.028(2) 0.046(3) 0.025(2) -0.003(2) 0.0021(18) 0.000(2)
O1D 0.0279(19) 0.042(2) 0.046(2) 0.0022(19) 0.0117(17) -0.0073(17)
O2D 0.037(2) 0.069(3) 0.095(4) -0.031(3) -0.003(3) 0.010(2)
O3D 0.058(3) 0.055(3) 0.046(3) -0.013(2) 0.015(2) -0.013(2)
C1B 0.052(4) 0.036(3) 0.032(3) -0.003(2) 0.010(3) -0.006(3)
C2B 0.038(3) 0.027(3) 0.033(3) 0.000(2) 0.017(2) -0.010(2)
C3B 0.041(3) 0.036(3) 0.030(3) -0.004(2) 0.012(2) -0.005(3)
C4B 0.028(3) 0.035(3) 0.039(3) -0.005(2) 0.010(2) 0.001(2)
C5B 0.029(3) 0.044(3) 0.022(2) 0.001(2) 0.005(2) 0.004(2)
C6B 0.040(3) 0.036(3) 0.034(3) -0.001(2) 0.009(2) -0.003(3)
C7B 0.040(3) 0.053(4) 0.046(3) -0.012(3) 0.017(3) -0.016(3)
C8B 0.027(3) 0.061(4) 0.044(3) -0.003(3) 0.007(2) 0.005(3)
C9B 0.036(3) 0.045(3) 0.032(3) 0.000(3) 0.005(2) 0.010(3)
C10B 0.032(3) 0.033(3) 0.030(3) 0.001(2) 0.006(2) 0.001(2)
C11B 0.077(6) 0.110(8) 0.046(4) -0.008(5) -0.016(4) 0.065(6)
C12B 0.107(9) 0.134(11) 0.082(7) -0.009(7) -0.045(6) 0.058(8)
C13B 0.040(5) 0.200(14) 0.073(6) -0.061(8) -0.029(4) 0.024(7)
C14B 0.035(4) 0.095(7) 0.077(6) -0.042(6) 0.002(4) 0.010(4)
C15B 0.101(10) 0.40(3) 0.088(9) -0.018(15) 0.000(8) -0.033(14)
N1B 0.029(2) 0.050(3) 0.031(2) -0.010(2) 0.0001(19) 0.009(2)
N2B 0.037(3) 0.066(4) 0.039(3) -0.017(3) -0.009(2) 0.016(3)
O1B 0.030(2) 0.048(3) 0.054(3) -0.013(2) 0.0101(18) 0.0049(19)
O2B 0.037(3) 0.100(5) 0.095(5) -0.040(4) 0.013(3) -0.015(3)
O3B 0.153(7) 0.073(5) 0.064(4) 0.005(3) -0.007(4) 0.059(5)
C1A 0.084(6) 0.096(7) 0.041(4) -0.020(4) 0.014(4) -0.026(5)
C2A 0.039(3) 0.073(5) 0.025(3) 0.002(3) 0.016(2) -0.009(3)
C3A 0.081(5) 0.082(6) 0.045(4) 0.025(4) 0.020(4) 0.000(5)
C4A 0.034(3) 0.041(3) 0.032(3) -0.004(2) 0.011(2) -0.006(3)
C5A 0.024(2) 0.036(3) 0.020(2) 0.000(2) 0.0042(18) -0.004(2)
C6A 0.037(3) 0.040(3) 0.038(3) 0.001(3) 0.007(2) -0.006(3)
C7A 0.028(3) 0.058(4) 0.054(4) 0.015(3) 0.007(3) -0.013(3)
C8A 0.030(3) 0.081(5) 0.029(3) 0.005(3) 0.007(2) 0.007(3)
C9A 0.044(4) 0.055(4) 0.051(4) -0.021(3) -0.008(3) 0.016(3)
C10A 0.037(3) 0.040(3) 0.041(3) -0.009(3) 0.001(2) 0.000(3)

```
 C11A 0.041(3) 0.027(3) 0.043(3) -0.007(3) 0.008(3) 0.004(3)
 C12A 0.045(4) 0.046(4) 0.052(4) 0.013(3) -0.001(3) 0.004(3)
 C13A 0.027(3) 0.043(3) 0.039(3) -0.006(3) 0.000(2) 0.002(3)
 C14A 0.025(3) 0.037(3) 0.036(3) -0.011(3) 0.005(2) 0.001(2)
 C15A 0.039(3) 0.067(4) 0.041(3) 0.001(3) 0.004(2) 0.003(3)
 N1A 0.023(2) 0.036(2) 0.025(2) -0.0015(19) 0.0046(17) -0.0024(19)
 N2A 0.026(2) 0.028(2) 0.030(2) -0.0022(18) 0.0043(18) 0.0006(18)
 O1A 0.033(2) 0.055(3) 0.047(2) -0.004(2) 0.0187(18) 0.0016(19)
 O2A 0.039(2) 0.035(2) 0.050(2) -0.003(2) 0.0064(19) -0.0097(19)
 O3A 0.050(2) 0.034(2) 0.045(2) -0.0036(19) 0.015(2) -0.001(2)
 C1C 0.068(5) 0.094(6) 0.030(3) -0.016(4) 0.008(3) -0.011(4)
 C2C 0.044(3) 0.055(4) 0.030(3) 0.004(3) 0.013(2) -0.012(3)
 C3C 0.075(5) 0.077(6) 0.031(3) 0.018(3) -0.001(3) -0.007(4)
 C4C 0.036(3) 0.034(3) 0.032(3) 0.002(2) 0.011(2) -0.008(2)
 C5C 0.026(3) 0.032(3) 0.023(2) 0.000(2) 0.003(2) 0.003(2)
 C6C 0.028(3) 0.035(3) 0.045(3) -0.004(3) -0.003(2) 0.001(2)
 C7C 0.032(3) 0.053(4) 0.071(5) -0.012(4) 0.000(3) 0.014(3)
 C8C 0.025(3) 0.071(5) 0.073(5) -0.020(4) 0.007(3) 0.004(3)
 C9C 0.031(3) 0.049(4) 0.077(5) -0.008(3) 0.007(3) -0.013(3)
 C10C 0.031(3) 0.032(3) 0.050(3) -0.007(3) 0.006(3) -0.005(2)
 C11C 0.030(3) 0.036(3) 0.037(3) 0.001(3) 0.003(2) 0.000(2)
 C12C 0.033(3) 0.046(4) 0.035(3) 0.000(3) 0.001(2) 0.001(3)
 C13C 0.039(3) 0.038(3) 0.033(3) 0.002(3) 0.007(2) 0.002(3)
 C14C 0.030(3) 0.033(3) 0.035(3) 0.004(3) 0.007(2) 0.003(3)
 C15C 0.060(4) 0.049(4) 0.055(4) 0.003(3) -0.010(3) 0.019(3)
 N1C 0.026(2) 0.035(3) 0.025(2) 0.0045(19) 0.0021(17) -0.0019(19)
 N2C 0.025(2) 0.036(3) 0.023(2) 0.0031(18) 0.0000(17) 0.0000(19)
 O1C 0.036(2) 0.056(3) 0.045(2) -0.003(2) 0.0188(18) 0.002(2)
 O2C 0.047(2) 0.034(2) 0.036(2) -0.0004(18) 0.0072(18) 0.000(2)
 O3C 0.049(3) 0.040(3) 0.055(3) 0.007(2) -0.011(2) -0.009(2)

_geom_special_details
;
 All esds (except the esd in the dihedral angle between two l.s.
planes)
 are estimated using the full covariance matrix.  The cell esds are
taken
 into account individually in the estimation of esds in distances,
angles
 and torsion angles; correlations between esds in cell parameters are
only
 used when they are defined by crystal symmetry.  An approximate
(isotropic)
 treatment of cell esds is used for estimating esds involving l.s.
planes.
;
loop_
  _geom_bond_atom_site_label_1
  _geom_bond_atom_site_label_2
  _geom_bond_distance
```

```
 _geom_bond_site_symmetry_2
 _geom_bond_publ_flag
C1D H1DA 0.9800 . ?
C1D H1DB 0.9800 . ?
C1D H1DC 0.9800 . ?
C1D C2D 1.504(8) . ?
C2D C3D 1.341(8) . ?
C2D C4D 1.484(7) . ?
C3D H3DA 0.9500 . ?
C3D H3DB 0.9500 . ?
C4D N1D 1.371(7) . ?
C4D O1D 1.228(6) . ?
C5D C6D 1.388(8) . ?
C5D C10D 1.374(8) . ?
C5D N1D 1.444(6) . ?
C6D H6D 0.9500 . ?
C6D C7D 1.386(8) . ?
C7D H7D 0.9500 . ?
C7D C8D 1.382(10) . ?
C8D H8D 0.9500 . ?
C8D C9D 1.374(10) . ?
C9D H9D 0.9500 . ?
C9D C10D 1.383(8) . ?
C10D H10D 0.9500 . ?
C11D C12D 1.524(9) . ?
C11D N2D 1.400(8) . ?
C11D O3D 1.188(9) . ?
C12D H12D 1.0000 . ?
C12D H12A 0.9900 . ?
C12D H12B 0.9900 . ?
C12D C13D 1.43(2) . ?
C12D C15D 1.436(17) . ?
C12D C17D 1.577(15) . ?
C13D H13A 0.9900 . ?
C13D H13B 0.9900 . ?
C13D C14D 1.637(19) . ?
C14D C17D 1.491(12) . ?
C14D N2D 1.402(9) . ?
C14D O2D 1.203(10) . ?
C15D H15A 0.9800 . ?
C15D H15B 0.9800 . ?
C15D H15C 0.9800 . ?
C16D H16A 0.9800 . ?
C16D H16B 0.9800 . ?
C16D H16C 0.9800 . ?
C16D C17D 1.508(19) . ?
C17D H17D 1.0000 . ?
N1D N2D 1.393(6) . ?
C1B H1BA 0.9800 . ?
C1B H1BB 0.9800 . ?
```

C1B H1BC 0.9800 . ?
C1B C2B 1.496(8) . ?
C2B C3B 1.323(8) . ?
C2B C4B 1.500(8) . ?
C3B H3BA 0.9500 . ?
C3B H3BB 0.9500 . ?
C4B N1B 1.376(7) . ?
C4B O1B 1.215(7) . ?
C5B C6B 1.382(9) . ?
C5B C10B 1.387(8) . ?
C5B N1B 1.444(7) . ?
C6B H6B 0.9500 . ?
C6B C7B 1.386(9) . ?
C7B H7B 0.9500 . ?
C7B C8B 1.377(10) . ?
C8B H8B 0.9500 . ?
C8B C9B 1.378(10) . ?
C9B H9B 0.9500 . ?
C9B C10B 1.407(8) . ?
C10B H10B 0.9500 . ?
C11B C12B 1.494(13) . ?
C11B N2B 1.402(12) . ?
C11B O3B 1.217(13) . ?
C12B H12C 0.9900 . ?
C12B H12E 0.9900 . ?
C12B C13B 1.512(19) . ?
C13B H13C 1.0000 . ?
C13B C14B 1.540(14) . ?
C13B C15B 1.376(17) . ?
C14B N2B 1.365(11) . ?
C14B O2B 1.174(12) . ?
C15B H15D 0.9800 . ?
C15B H15E 0.9800 . ?
C15B H15F 0.9800 . ?
N1B N2B 1.389(7) . ?
C1A H1AA 0.9800 . ?
C1A H1AB 0.9800 . ?
C1A H1AC 0.9800 . ?
C1A C2A 1.499(11) . ?
C2A C3A 1.306(12) . ?
C2A C4A 1.503(8) . ?
C3A H3AA 0.9500 . ?
C3A H3AB 0.9500 . ?
C4A N1A 1.383(7) . ?
C4A O1A 1.223(7) . ?
C5A C6A 1.373(8) . ?
C5A C10A 1.386(8) . ?
C5A N1A 1.441(7) . ?
C6A H6A 0.9500 . ?
C6A C7A 1.391(9) . ?

C7A H7A 0.9500 . ?
C7A C8A 1.351(10) . ?
C8A H8A 0.9500 . ?
C8A C9A 1.364(11) . ?
C9A H9A 0.9500 . ?
C9A C10A 1.379(9) . ?
C10A H10A 0.9500 . ?
C11A C12A 1.512(9) . ?
C11A N2A 1.400(8) . ?
C11A O3A 1.199(7) . ?
C12A H12F 0.9900 . ?
C12A H12G 0.9900 . ?
C12A C13A 1.542(10) . ?
C13A H13D 1.0000 . ?
C13A C14A 1.513(8) . ?
C13A C15A 1.521(8) . ?
C14A N2A 1.388(7) . ?
C14A O2A 1.191(8) . ?
C15A H15G 0.9800 . ?
C15A H15H 0.9800 . ?
C15A H15I 0.9800 . ?
N1A N2A 1.400(6) . ?
C1C H1CA 0.9800 . ?
C1C H1CB 0.9800 . ?
C1C H1CC 0.9800 . ?
C1C C2C 1.481(10) . ?
C2C C3C 1.336(11) . ?
C2C C4C 1.486(8) . ?
C3C H3CA 0.9500 . ?
C3C H3CB 0.9500 . ?
C4C N1C 1.369(7) . ?
C4C O1C 1.214(7) . ?
C5C C6C 1.372(8) . ?
C5C C10C 1.375(8) . ?
C5C N1C 1.444(7) . ?
C6C H6C 0.9500 . ?
C6C C7C 1.387(9) . ?
C7C H7C 0.9500 . ?
C7C C8C 1.371(11) . ?
C8C H8C 0.9500 . ?
C8C C9C 1.387(10) . ?
C9C H9C 0.9500 . ?
C9C C10C 1.407(9) . ?
C10C H10C 0.9500 . ?
C11C C12C 1.495(8) . ?
C11C N2C 1.404(8) . ?
C11C O3C 1.205(8) . ?
C12C H12H 0.9900 . ?
C12C H12I 0.9900 . ?
C12C C13C 1.547(9) . ?

```
 C13C H13E 1.0000 . ?
 C13C C14C 1.511(7) . ?
 C13C C15C 1.502(9) . ?
 C14C N2C 1.386(7) . ?
 C14C O2C 1.210(7) . ?
 C15C H15J 0.9800 . ?
 C15C H15K 0.9800 . ?
 C15C H15L 0.9800 . ?
 N1C N2C 1.388(6) . ?

loop_
  _geom_angle_atom_site_label_1
  _geom_angle_atom_site_label_2
  _geom_angle_atom_site_label_3
  _geom_angle
  _geom_angle_site_symmetry_1
  _geom_angle_site_symmetry_3
  _geom_angle_publ_flag
 H1DA C1D H1DB 109.5 . . ?
 H1DA C1D H1DC 109.5 . . ?
 H1DB C1D H1DC 109.5 . . ?
 C2D C1D H1DA 109.5 . . ?
 C2D C1D H1DB 109.5 . . ?
 C2D C1D H1DC 109.5 . . ?
 C3D C2D C1D 123.8(5) . . ?
 C3D C2D C4D 121.1(5) . . ?
 C4D C2D C1D 114.9(5) . . ?
 C2D C3D H3DA 120.0 . . ?
 C2D C3D H3DB 120.0 . . ?
 H3DA C3D H3DB 120.0 . . ?
 N1D C4D C2D 117.6(4) . . ?
 O1D C4D C2D 121.5(5) . . ?
 O1D C4D N1D 120.8(5) . . ?
 C6D C5D N1D 119.6(5) . . ?
 C10D C5D C6D 121.9(5) . . ?
 C10D C5D N1D 118.5(5) . . ?
 C5D C6D H6D 121.1 . . ?
 C7D C6D C5D 117.8(6) . . ?
 C7D C6D H6D 121.1 . . ?
 C6D C7D H7D 119.6 . . ?
 C8D C7D C6D 120.9(6) . . ?
 C8D C7D H7D 119.6 . . ?
 C7D C8D H8D 120.0 . . ?
 C9D C8D C7D 120.0(6) . . ?
 C9D C8D H8D 120.0 . . ?
 C8D C9D H9D 119.9 . . ?
 C8D C9D C10D 120.3(6) . . ?
 C10D C9D H9D 119.9 . . ?
 C5D C10D C9D 119.1(6) . . ?
 C5D C10D H10D 120.4 . . ?
```

C9D C10D H10D 120.4 . . ?
N2D C11D C12D 104.5(6) . . ?
O3D C11D C12D 132.0(6) . . ?
O3D C11D N2D 123.4(6) . . ?
C11D C12D H12D 107.1 . . ?
C11D C12D H12A 110.8 . . ?
C11D C12D H12B 110.8 . . ?
C11D C12D C17D 104.7(6) . . ?
H12A C12D H12B 108.9 . . ?
C13D C12D C11D 110.7(9) . . ?
C13D C12D H12D 107.1 . . ?
C13D C12D C15D 113.8(13) . . ?
C15D C12D C11D 110.6(9) . . ?
C15D C12D H12D 107.1 . . ?
C17D C12D H12A 110.8 . . ?
C17D C12D H12B 110.8 . . ?
C12D C13D H13A 111.5 . . ?
C12D C13D H13B 111.5 . . ?
C12D C13D C14D 101.4(13) . . ?
H13A C13D H13B 109.3 . . ?
C14D C13D H13A 111.5 . . ?
C14D C13D H13B 111.5 . . ?
N2D C14D C13D 103.5(10) . . ?
N2D C14D C17D 107.9(8) . . ?
O2D C14D C13D 130.0(11) . . ?
O2D C14D C17D 126.2(8) . . ?
O2D C14D N2D 124.3(6) . . ?
C12D C15D H15A 109.5 . . ?
C12D C15D H15B 109.5 . . ?
C12D C15D H15C 109.5 . . ?
H15A C15D H15B 109.5 . . ?
H15A C15D H15C 109.5 . . ?
H15B C15D H15C 109.5 . . ?
H16A C16D H16B 109.5 . . ?
H16A C16D H16C 109.5 . . ?
H16B C16D H16C 109.5 . . ?
C17D C16D H16A 109.5 . . ?
C17D C16D H16B 109.5 . . ?
C17D C16D H16C 109.5 . . ?
C12D C17D H17D 110.8 . . ?
C14D C17D C12D 101.6(8) . . ?
C14D C17D C16D 107.8(11) . . ?
C14D C17D H17D 110.8 . . ?
C16D C17D C12D 114.7(12) . . ?
C16D C17D H17D 110.8 . . ?
C4D N1D C5D 128.4(4) . . ?
C4D N1D N2D 116.0(4) . . ?
N2D N1D C5D 115.6(4) . . ?
C11D N2D C14D 114.5(5) . . ?
N1D N2D C11D 122.4(5) . . ?

N1D N2D C14D 121.8(5) . . ?
H1BA C1B H1BB 109.5 . . ?
H1BA C1B H1BC 109.5 . . ?
H1BB C1B H1BC 109.5 . . ?
C2B C1B H1BA 109.5 . . ?
C2B C1B H1BB 109.5 . . ?
C2B C1B H1BC 109.5 . . ?
C1B C2B C4B 112.7(5) . . ?
C3B C2B C1B 124.7(5) . . ?
C3B C2B C4B 122.4(5) . . ?
C2B C3B H3BA 120.0 . . ?
C2B C3B H3BB 120.0 . . ?
H3BA C3B H3BB 120.0 . . ?
N1B C4B C2B 116.8(5) . . ?
O1B C4B C2B 122.3(5) . . ?
O1B C4B N1B 120.9(5) . . ?
C6B C5B C10B 121.8(5) . . ?
C6B C5B N1B 120.0(6) . . ?
C10B C5B N1B 118.2(5) . . ?
C5B C6B H6B 120.3 . . ?
C5B C6B C7B 119.3(6) . . ?
C7B C6B H6B 120.3 . . ?
C6B C7B H7B 120.1 . . ?
C8B C7B C6B 119.9(6) . . ?
C8B C7B H7B 120.1 . . ?
C7B C8B H8B 119.6 . . ?
C7B C8B C9B 120.8(6) . . ?
C9B C8B H8B 119.6 . . ?
C8B C9B H9B 119.9 . . ?
C8B C9B C10B 120.3(6) . . ?
C10B C9B H9B 119.9 . . ?
C5B C10B C9B 117.8(6) . . ?
C5B C10B H10B 121.1 . . ?
C9B C10B H10B 121.1 . . ?
N2B C11B C12B 107.9(11) . . ?
O3B C11B C12B 127.0(10) . . ?
O3B C11B N2B 125.0(7) . . ?
C11B C12B H12C 110.9 . . ?
C11B C12B H12E 110.9 . . ?
C11B C12B C13B 104.3(9) . . ?
H12C C12B H12E 108.9 . . ?
C13B C12B H12C 110.9 . . ?
C13B C12B H12E 110.9 . . ?
C12B C13B H13C 108.7 . . ?
C12B C13B C14B 106.6(8) . . ?
C14B C13B H13C 108.7 . . ?
C15B C13B C12B 112.7(15) . . ?
C15B C13B H13C 108.7 . . ?
C15B C13B C14B 111.3(13) . . ?
N2B C14B C13B 104.9(10) . . ?

O2B C14B C13B 130.3(10) . . ?
O2B C14B N2B 124.8(8) . . ?
C13B C15B H15D 109.5 . . ?
C13B C15B H15E 109.5 . . ?
C13B C15B H15F 109.5 . . ?
H15D C15B H15E 109.5 . . ?
H15D C15B H15F 109.5 . . ?
H15E C15B H15F 109.5 . . ?
C4B N1B C5B 127.6(5) . . ?
C4B N1B N2B 115.5(5) . . ?
N2B N1B C5B 116.5(5) . . ?
C14B N2B C11B 115.1(7) . . ?
C14B N2B N1B 123.2(7) . . ?
N1B N2B C11B 120.6(7) . . ?
H1AA C1A H1AB 109.5 . . ?
H1AA C1A H1AC 109.5 . . ?
H1AB C1A H1AC 109.5 . . ?
C2A C1A H1AA 109.5 . . ?
C2A C1A H1AB 109.5 . . ?
C2A C1A H1AC 109.5 . . ?
C1A C2A C4A 113.1(6) . . ?
C3A C2A C1A 125.1(7) . . ?
C3A C2A C4A 121.5(7) . . ?
C2A C3A H3AA 120.0 . . ?
C2A C3A H3AB 120.0 . . ?
H3AA C3A H3AB 120.0 . . ?
N1A C4A C2A 115.7(5) . . ?
O1A C4A C2A 123.4(5) . . ?
O1A C4A N1A 120.9(5) . . ?
C6A C5A C10A 120.7(5) . . ?
C6A C5A N1A 120.2(5) . . ?
C10A C5A N1A 119.2(5) . . ?
C5A C6A H6A 120.8 . . ?
C5A C6A C7A 118.4(6) . . ?
C7A C6A H6A 120.8 . . ?
C6A C7A H7A 119.4 . . ?
C8A C7A C6A 121.2(6) . . ?
C8A C7A H7A 119.4 . . ?
C7A C8A H8A 119.9 . . ?
C7A C8A C9A 120.2(6) . . ?
C9A C8A H8A 119.9 . . ?
C8A C9A H9A 119.8 . . ?
C8A C9A C10A 120.4(7) . . ?
C10A C9A H9A 119.8 . . ?
C5A C10A H10A 120.5 . . ?
C9A C10A C5A 119.1(6) . . ?
C9A C10A H10A 120.5 . . ?
N2A C11A C12A 107.2(5) . . ?
O3A C11A C12A 129.4(6) . . ?
O3A C11A N2A 123.3(6) . . ?

C11A C12A H12F 110.9 . . ?
C11A C12A H12G 110.9 . . ?
C11A C12A C13A 104.5(5) . . ?
H12F C12A H12G 108.9 . . ?
C13A C12A H12F 110.9 . . ?
C13A C12A H12G 110.9 . . ?
C12A C13A H13D 109.1 . . ?
C14A C13A C12A 106.5(5) . . ?
C14A C13A H13D 109.1 . . ?
C14A C13A C15A 110.8(5) . . ?
C15A C13A C12A 112.2(5) . . ?
C15A C13A H13D 109.1 . . ?
N2A C14A C13A 106.4(5) . . ?
O2A C14A C13A 128.8(6) . . ?
O2A C14A N2A 124.8(5) . . ?
C13A C15A H15G 109.5 . . ?
C13A C15A H15H 109.5 . . ?
C13A C15A H15I 109.5 . . ?
H15G C15A H15H 109.5 . . ?
H15G C15A H15I 109.5 . . ?
H15H C15A H15I 109.5 . . ?
C4A N1A C5A 125.9(4) . . ?
C4A N1A N2A 116.0(4) . . ?
N2A N1A C5A 116.6(4) . . ?
C14A N2A C11A 115.0(5) . . ?
C14A N2A N1A 121.6(5) . . ?
N1A N2A C11A 122.6(5) . . ?
H1CA C1C H1CB 109.5 . . ?
H1CA C1C H1CC 109.5 . . ?
H1CB C1C H1CC 109.5 . . ?
C2C C1C H1CA 109.5 . . ?
C2C C1C H1CB 109.5 . . ?
C2C C1C H1CC 109.5 . . ?
C1C C2C C4C 115.6(6) . . ?
C3C C2C C1C 124.1(6) . . ?
C3C C2C C4C 120.1(6) . . ?
C2C C3C H3CA 120.0 . . ?
C2C C3C H3CB 120.0 . . ?
H3CA C3C H3CB 120.0 . . ?
N1C C4C C2C 116.3(5) . . ?
O1C C4C C2C 121.6(6) . . ?
O1C C4C N1C 122.1(5) . . ?
C6C C5C C10C 121.9(5) . . ?
C6C C5C N1C 119.5(5) . . ?
C10C C5C N1C 118.6(5) . . ?
C5C C6C H6C 120.3 . . ?
C5C C6C C7C 119.4(6) . . ?
C7C C6C H6C 120.3 . . ?
C6C C7C H7C 120.0 . . ?
C8C C7C C6C 120.0(7) . . ?

```
 C8C C7C H7C 120.0 . . ?
 C7C C8C H8C 119.7 . . ?
 C7C C8C C9C 120.6(6) . . ?
 C9C C8C H8C 119.7 . . ?
 C8C C9C H9C 120.2 . . ?
 C8C C9C C10C 119.5(6) . . ?
 C10C C9C H9C 120.2 . . ?
 C5C C10C C9C 118.5(6) . . ?
 C5C C10C H10C 120.8 . . ?
 C9C C10C H10C 120.8 . . ?
 N2C C11C C12C 107.6(5) . . ?
 O3C C11C C12C 129.6(6) . . ?
 O3C C11C N2C 122.8(5) . . ?
 C11C C12C H12H 110.7 . . ?
 C11C C12C H12I 110.7 . . ?
 C11C C12C C13C 105.4(5) . . ?
 H12H C12C H12I 108.8 . . ?
 C13C C12C H12H 110.7 . . ?
 C13C C12C H12I 110.7 . . ?
 C12C C13C H13E 109.1 . . ?
 C14C C13C C12C 105.1(5) . . ?
 C14C C13C H13E 109.1 . . ?
 C15C C13C C12C 113.8(5) . . ?
 C15C C13C H13E 109.1 . . ?
 C15C C13C C14C 110.5(5) . . ?
 N2C C14C C13C 107.3(5) . . ?
 O2C C14C C13C 128.9(5) . . ?
 O2C C14C N2C 123.7(5) . . ?
 C13C C15C H15J 109.5 . . ?
 C13C C15C H15K 109.5 . . ?
 C13C C15C H15L 109.5 . . ?
 H15J C15C H15K 109.5 . . ?
 H15J C15C H15L 109.5 . . ?
 H15K C15C H15L 109.5 . . ?
 C4C N1C C5C 127.2(4) . . ?
 C4C N1C N2C 115.9(4) . . ?
 N2C N1C C5C 115.5(4) . . ?
 C14C N2C C11C 114.0(4) . . ?
 C14C N2C N1C 122.6(5) . . ?
 N1C N2C C11C 122.6(5) . . ?

loop_
  _geom_torsion_atom_site_label_1
  _geom_torsion_atom_site_label_2
  _geom_torsion_atom_site_label_3
  _geom_torsion_atom_site_label_4
  _geom_torsion
  _geom_torsion_site_symmetry_1
  _geom_torsion_site_symmetry_2
  _geom_torsion_site_symmetry_3
```

_geom_torsion_site_symmetry_4
_geom_torsion_publ_flag
C1D C2D C4D N1D 127.6(6) . . . . ?
C1D C2D C4D O1D -52.8(7) . . . . ?
C2D C4D N1D C5D -3.1(8) . . . . ?
C2D C4D N1D N2D 177.9(5) . . . . ?
C3D C2D C4D N1D -56.8(7) . . . . ?
C3D C2D C4D O1D 122.7(6) . . . . ?
C4D N1D N2D C11D 83.5(6) . . . . ?
C4D N1D N2D C14D -82.4(7) . . . . ?
C5D C6D C7D C8D -0.3(9) . . . . ?
C5D N1D N2D C11D -95.6(6) . . . . ?
C5D N1D N2D C14D 98.5(7) . . . . ?
C6D C5D C10D C9D -0.6(8) . . . . ?
C6D C5D N1D C4D -74.5(7) . . . . ?
C6D C5D N1D N2D 104.5(6) . . . . ?
C6D C7D C8D C9D 1.5(10) . . . . ?
C7D C8D C9D C10D -2.2(10) . . . . ?
C8D C9D C10D C5D 1.8(9) . . . . ?
C10D C5D C6D C7D -0.2(8) . . . . ?
C10D C5D N1D C4D 105.5(7) . . . . ?
C10D C5D N1D N2D -75.6(6) . . . . ?
C11D C12D C13D C14D 19.3(16) . . . . ?
C11D C12D C17D C14D -25.8(11) . . . . ?
C11D C12D C17D C16D -141.8(10) . . . . ?
C12D C11D N2D C14D -7.6(7) . . . . ?
C12D C11D N2D N1D -174.5(5) . . . . ?
C12D C13D C14D N2D -22.8(16) . . . . ?
C12D C13D C14D O2D 173.7(10) . . . . ?
C13D C14D N2D C11D 19.1(12) . . . . ?
C13D C14D N2D N1D -174.0(10) . . . . ?
C15D C12D C13D C14D -106.1(13) . . . . ?
C17D C14D N2D C11D -9.8(10) . . . . ?
C17D C14D N2D N1D 157.1(8) . . . . ?
N1D C5D C6D C7D 179.8(5) . . . . ?
N1D C5D C10D C9D 179.5(5) . . . . ?
N2D C11D C12D C13D -9.4(13) . . . . ?
N2D C11D C12D C15D 117.8(10) . . . . ?
N2D C11D C12D C17D 20.8(9) . . . . ?
N2D C14D C17D C12D 21.8(12) . . . . ?
N2D C14D C17D C16D 142.7(10) . . . . ?
O1D C4D N1D C5D 177.3(5) . . . . ?
O1D C4D N1D N2D -1.7(8) . . . . ?
O2D C14D C17D C12D -172.2(9) . . . . ?
O2D C14D C17D C16D -51.2(17) . . . . ?
O2D C14D N2D C11D -176.2(8) . . . . ?
O2D C14D N2D N1D -9.2(12) . . . . ?
O3D C11D C12D C13D 167.5(13) . . . . ?
O3D C11D C12D C15D -65.3(12) . . . . ?
O3D C11D C12D C17D -162.4(9) . . . . ?

O3D C11D N2D C14D 175.2(7) . . . . ?
O3D C11D N2D N1D 8.3(9) . . . . ?
C1B C2B C4B N1B −129.0(6) . . . . ?
C1B C2B C4B O1B 50.3(8) . . . . ?
C2B C4B N1B C5B 5.2(9) . . . . ?
C2B C4B N1B N2B 176.9(5) . . . . ?
C3B C2B C4B N1B 55.8(8) . . . . ?
C3B C2B C4B O1B −124.9(7) . . . . ?
C4B N1B N2B C11B −77.9(8) . . . . ?
C4B N1B N2B C14B 89.2(8) . . . . ?
C5B C6B C7B C8B −0.7(9) . . . . ?
C5B N1B N2B C11B 94.8(8) . . . . ?
C5B N1B N2B C14B −98.2(8) . . . . ?
C6B C5B C10B C9B −0.7(8) . . . . ?
C6B C5B N1B C4B 75.4(8) . . . . ?
C6B C5B N1B N2B −96.2(7) . . . . ?
C6B C7B C8B C9B −0.2(10) . . . . ?
C7B C8B C9B C10B 0.6(9) . . . . ?
C8B C9B C10B C5B −0.1(8) . . . . ?
C10B C5B C6B C7B 1.1(9) . . . . ?
C10B C5B N1B C4B −104.5(7) . . . . ?
C10B C5B N1B N2B 83.9(7) . . . . ?
C11B C12B C13B C14B −9.4(12) . . . . ?
C11B C12B C13B C15B −131.8(11) . . . . ?
C12B C11B N2B C14B 2.6(11) . . . . ?
C12B C11B N2B N1B 170.7(7) . . . . ?
C12B C13B C14B N2B 10.9(11) . . . . ?
C12B C13B C14B O2B −171.1(11) . . . . ?
C13B C14B N2B C11B −8.5(9) . . . . ?
C13B C14B N2B N1B −176.2(6) . . . . ?
C15B C13B C14B N2B 134.2(15) . . . . ?
C15B C13B C14B O2B −47.8(18) . . . . ?
N1B C5B C6B C7B −178.7(5) . . . . ?
N1B C5B C10B C9B 179.1(5) . . . . ?
N2B C11B C12B C13B 4.7(12) . . . . ?
O1B C4B N1B C5B −174.1(6) . . . . ?
O1B C4B N1B N2B −2.5(9) . . . . ?
O2B C14B N2B C11B 173.3(9) . . . . ?
O2B C14B N2B N1B 5.6(13) . . . . ?
O3B C11B C12B C13B −176.9(11) . . . . ?
O3B C11B N2B C14B −175.9(10) . . . . ?
O3B C11B N2B N1B −7.8(13) . . . . ?
C1A C2A C4A N1A −125.1(6) . . . . ?
C1A C2A C4A O1A 57.4(9) . . . . ?
C2A C4A N1A C5A 17.9(8) . . . . ?
C2A C4A N1A N2A −176.9(5) . . . . ?
C3A C2A C4A N1A 61.1(9) . . . . ?
C3A C2A C4A O1A −116.4(8) . . . . ?
C4A N1A N2A C11A −81.7(7) . . . . ?
C4A N1A N2A C14A 87.5(6) . . . . ?

C5A C6A C7A C8A −0.3(10) . . . . ?
C5A N1A N2A C11A 85.0(6) . . . . ?
C5A N1A N2A C14A −105.8(6) . . . . ?
C6A C5A C10A C9A 0.7(9) . . . . ?
C6A C5A N1A C4A 67.6(8) . . . . ?
C6A C5A N1A N2A −97.5(6) . . . . ?
C6A C7A C8A C9A 0.6(10) . . . . ?
C7A C8A C9A C10A −0.2(10) . . . . ?
C8A C9A C10A C5A −0.5(10) . . . . ?
C10A C5A C6A C7A −0.3(9) . . . . ?
C10A C5A N1A C4A −112.7(6) . . . . ?
C10A C5A N1A N2A 82.2(6) . . . . ?
C11A C12A C13A C14A 4.6(7) . . . . ?
C11A C12A C13A C15A −116.8(6) . . . . ?
C12A C11A N2A C14A 6.9(7) . . . . ?
C12A C11A N2A N1A 176.7(5) . . . . ?
C12A C13A C14A N2A −0.8(6) . . . . ?
C12A C13A C14A O2A −178.8(6) . . . . ?
C13A C14A N2A C11A −3.8(6) . . . . ?
C13A C14A N2A N1A −173.8(4) . . . . ?
C15A C13A C14A N2A 121.4(5) . . . . ?
C15A C13A C14A O2A −56.6(8) . . . . ?
N1A C5A C6A C7A 179.4(5) . . . . ?
N1A C5A C10A C9A −179.0(5) . . . . ?
N2A C11A C12A C13A −6.7(7) . . . . ?
O1A C4A N1A C5A −164.5(6) . . . . ?
O1A C4A N1A N2A 0.7(8) . . . . ?
O2A C14A N2A C11A 174.3(5) . . . . ?
O2A C14A N2A N1A 4.3(8) . . . . ?
O3A C11A C12A C13A 174.2(6) . . . . ?
O3A C11A N2A C14A −174.0(6) . . . . ?
O3A C11A N2A N1A −4.1(9) . . . . ?
C1C C2C C4C N1C 125.3(6) . . . . ?
C1C C2C C4C O1C −56.9(8) . . . . ?
C2C C4C N1C C5C −17.9(8) . . . . ?
C2C C4C N1C N2C 176.4(5) . . . . ?
C3C C2C C4C N1C −59.5(8) . . . . ?
C3C C2C C4C O1C 118.3(8) . . . . ?
C4C N1C N2C C11C −86.3(6) . . . . ?
C4C N1C N2C C14C 83.1(6) . . . . ?
C5C C6C C7C C8C 1.2(11) . . . . ?
C5C N1C N2C C11C 106.3(6) . . . . ?
C5C N1C N2C C14C −84.3(6) . . . . ?
C6C C5C C10C C9C −0.1(9) . . . . ?
C6C C5C N1C C4C 112.1(6) . . . . ?
C6C C5C N1C N2C −82.1(6) . . . . ?
C6C C7C C8C C9C −1.7(12) . . . . ?
C7C C8C C9C C10C 1.3(12) . . . . ?
C8C C9C C10C C5C −0.4(11) . . . . ?
C10C C5C C6C C7C −0.3(9) . . . . ?

```
 C10C C5C N1C C4C -69.6(7) . . . . ?
 C10C C5C N1C N2C 96.2(6) . . . . ?
 C11C C12C C13C C14C -5.2(6) . . . . ?
 C11C C12C C13C C15C -126.1(6) . . . . ?
 C12C C11C N2C C14C 4.3(7) . . . . ?
 C12C C11C N2C N1C 174.5(5) . . . . ?
 C12C C13C C14C N2C 7.7(6) . . . . ?
 C12C C13C C14C O2C -173.4(6) . . . . ?
 C13C C14C N2C C11C -7.7(6) . . . . ?
 C13C C14C N2C N1C -178.0(5) . . . . ?
 C15C C13C C14C N2C 130.8(6) . . . . ?
 C15C C13C C14C O2C -50.3(8) . . . . ?
 N1C C5C C6C C7C 177.9(5) . . . . ?
 N1C C5C C10C C9C -178.4(6) . . . . ?
 N2C C11C C12C C13C 1.0(6) . . . . ?
 O1C C4C N1C C5C 164.4(6) . . . . ?
 O1C C4C N1C N2C -1.3(8) . . . . ?
 O2C C14C N2C C11C 173.3(5) . . . . ?
 O2C C14C N2C N1C 3.1(9) . . . . ?
 O3C C11C C12C C13C -178.1(7) . . . . ?
 O3C C11C N2C C14C -176.6(6) . . . . ?
 O3C C11C N2C N1C -6.3(9) . . . . ?

_shelx_res_file
;
    cu_axi20_0m.res created by SHELXL-2014/7

TITL cu_axi20_0m in P2(1)
CELL 1.54178 8.8088 23.665 14.1477 90 104.096 90
ZERR 8 0.0004 0.0011 0.0007 0 0.002 0
LATT -1
SYMM -X,0.5+Y,-Z
SFAC C H N O
UNIT 120 128 16 24

L.S. 7
PLAN  -5 0 0
SIZE 0.148 0.174 0.24
TEMP -173.14
HTAB
BOND $H
CONF
fmap 2
acta
REM <olex2.extras>
REM <HklSrc "%.\\cu_axi20_0m.hkl">
REM </olex2.extras>

WGHT    0.073000    1.915400
FVAR       0.49180   0.54849
```

```
C1D   1    2.736052    1.749920    1.075647    11.00000    0.06265
0.03416 =
         0.04661    0.00919    0.00855    0.01095
AFIX 137
H1DA  2    2.657682    1.751940    1.013295    11.00000   -1.50000
H1DB  2    2.838546    1.760882    1.066068    11.00000   -1.50000
H1DC  2    2.706589    1.775663    1.122439    11.00000   -1.50000
AFIX   0
C2D   1    2.744245    1.690486    1.114232    11.00000    0.02607
0.03575 =
         0.03053    0.00112    0.01374    0.00061
C3D   1    2.665895    1.646887    1.064645    11.00000    0.03661
0.04538 =
         0.02621    0.00420    0.00773   -0.00081
AFIX  93
H3DA  2    2.681879    1.609758    1.090704    11.00000   -1.20000
H3DB  2    2.594390    1.653119    1.003447    11.00000   -1.20000
AFIX   0
C4D   1    2.856610    1.681908    1.209981    11.00000    0.03214
0.02511 =
         0.02867   -0.00178    0.01186    0.00096
C5D   1    2.644240    1.638767    1.279943    11.00000    0.02145
0.03563 =
         0.01923   -0.00073    0.00391   -0.00288
C6D   1    2.531756    1.678948    1.285051    11.00000    0.03388
0.04188 =
         0.02578    0.00580    0.00933    0.00233
AFIX  43
H6D   2    2.556826    1.718048    1.290360    11.00000   -1.20000
AFIX   0
C7D   1    2.381642    1.660172    1.282154    11.00000    0.02503
0.06984 =
         0.03521    0.00553    0.01061    0.00543
AFIX  43
H7D   2    2.302505    1.686777    1.285797    11.00000   -1.20000
AFIX   0
C8D   1    2.345540    1.603252    1.274036    11.00000    0.02952
0.07383 =
         0.03651    0.00033    0.00601   -0.01109
AFIX  43
H8D   2    2.241613    1.591028    1.270607    11.00000   -1.20000
AFIX   0
C9D   1    2.460031    1.564377    1.270929    11.00000    0.03760
0.05258 =
         0.03793   -0.00119    0.00773   -0.01538
AFIX  43
H9D   2    2.435901    1.525191    1.267681    11.00000   -1.20000
AFIX   0
C10D  1    2.610270    1.582048    1.272509    11.00000    0.03438
0.04002 =
```

```
         0.02603    0.00084    0.00921   -0.00371
AFIX  43
H10D  2    2.688930    1.555319    1.268508    11.00000   -1.20000
AFIX   0
C11D  1    2.953502    1.689495    1.441393    11.00000    0.04107
0.04997 =
         0.03099   -0.00330    0.01385   -0.00761
C12D  1    3.086494    1.663494    1.518686    11.00000    0.03713
0.07864 =
         0.02712   -0.00469   -0.00094   -0.00969
PART 2
AFIX  13
H12D  2    3.056554    1.666285    1.582317   -21.00000   -1.20000
AFIX  23
PART 0
PART 1
H12A  2    3.047339    1.648307    1.573512    21.00000   -1.20000
H12B  2    3.168801    1.691876    1.544297    21.00000   -1.20000
AFIX   0
PART 0
PART 2
C13D  1    3.103107    1.604677    1.499745   -21.00000    0.03832
0.10398 =
         0.03960    0.00165    0.00538    0.01094
AFIX  23
H13A  2    3.052428    1.580718    1.540578   -21.00000   -1.20000
H13B  2    3.214555    1.593793    1.510667   -21.00000   -1.20000
AFIX   0
PART 0
C14D  1    3.012077    1.600519    1.384392    11.00000    0.02522
0.08347 =
         0.05777   -0.00443   -0.01047    0.00627
PART 2
C15D  1    3.227923    1.695494    1.528168   -21.00000    0.06194
0.07348 =
         0.05005   -0.00817   -0.00174   -0.01468
AFIX 137
H15A  2    3.253005    1.698210    1.464531   -21.00000   -1.50000
H15B  2    3.313876    1.676600    1.574446   -21.00000   -1.50000
H15C  2    3.213407    1.733510    1.551998   -21.00000   -1.50000
AFIX   0
PART 0
PART 1
C16D  1    3.199507    1.561833    1.523906    21.00000    0.06842
0.06513 =
         0.05070    0.00235   -0.01336    0.02390
AFIX 137
H16A  2    3.290185    1.570100    1.577762    21.00000   -1.50000
H16B  2    3.226983    1.532194    1.482630    21.00000   -1.50000
H16C  2    3.112274    1.548946    1.550157    21.00000   -1.50000
```

```
AFIX   0
C17D  1    3.151936    1.614536    1.464171    21.00000    0.02014
0.06817 =
         0.02374   -0.00189   -0.00029    0.00455
AFIX  13
H17D  2    3.239605    1.628508    1.436882    21.00000   -1.20000
AFIX   0
PART 0
N1D   3    2.801946    1.656557    1.282342    11.00000    0.02240
0.04508 =
         0.02349   -0.00170    0.00326   -0.00453
N2D   3    2.911913    1.647451    1.370140    11.00000    0.02823
0.04581 =
         0.02507   -0.00333    0.00207   -0.00042
O1D   4    2.994057    1.696570    1.224232    11.00000    0.02794
0.04158 =
         0.04615    0.00216    0.01170   -0.00727
O2D   4    3.000270    1.561439    1.328655    11.00000    0.03716
0.06884 =
         0.09520   -0.03113   -0.00332    0.01021
O3D   4    2.887519    1.733552    1.437541    11.00000    0.05819
0.05547 =
         0.04558   -0.01291    0.01541   -0.01341

C1B   1    3.227468    1.774883    1.084309    11.00000    0.05211
0.03558 =
         0.03207   -0.00313    0.00954   -0.00614
AFIX 137
H1BA  2    3.161733    1.774197    1.017586    11.00000   -1.50000
H1BB  2    3.184436    1.748882    1.125020    11.00000   -1.50000
H1BC  2    3.334079    1.763188    1.084022    11.00000   -1.50000
AFIX   0
C2B   1    3.230760    1.833461    1.124444    11.00000    0.03771
0.02652 =
         0.03292   -0.00027    0.01653   -0.00954
C3B   1    3.151831    1.876812    1.077675    11.00000    0.04070
0.03634 =
         0.02994   -0.00355    0.01217   -0.00533
AFIX  93
H3BA  2    3.087250    1.871864    1.013849    11.00000   -1.20000
H3BB  2    3.159724    1.912896    1.107910    11.00000   -1.20000
AFIX   0
C4B   1    3.345031    1.839729    1.221608    11.00000    0.02755
0.03485 =
         0.03859   -0.00512    0.00954    0.00066
C5B   1    3.130246    1.879851    1.292239    11.00000    0.02917
0.04375 =
         0.02199    0.00099    0.00510    0.00374
C6B   1    3.020684    1.838917    1.297339    11.00000    0.04003
0.03647 =
```

```
         0.03433   -0.00096    0.00924   -0.00286
AFIX  43
H6B   2    3.049975    1.800216    1.304062    11.00000   -1.20000
AFIX   0
C7B   1    2.867574    1.854889    1.292547    11.00000    0.04029
0.05264 =
         0.04555   -0.01230    0.01697   -0.01586
AFIX  43
H7B   2    2.791012    1.827071    1.295298    11.00000   -1.20000
AFIX   0
C8B   1    2.826677    1.911164    1.283792    11.00000    0.02735
0.06082 =
         0.04369   -0.00334    0.00712    0.00471
AFIX  43
H8B   2    2.721551    1.921909    1.280329    11.00000   -1.20000
AFIX   0
C9B   1    2.936331    1.952065    1.279993    11.00000    0.03567
0.04454 =
         0.03211    0.00035    0.00520    0.01020
AFIX  43
H9B   2    2.906581    1.990741    1.274569    11.00000   -1.20000
AFIX   0
C10B  1    3.092141    1.936809    1.284110    11.00000    0.03191
0.03250 =
         0.03005    0.00133    0.00602    0.00077
AFIX  43
H10B  2    3.168773    1.964609    1.281411    11.00000   -1.20000
AFIX   0
C11B  1    3.428730    1.818291    1.444655    11.00000    0.07708
0.10968 =
         0.04551   -0.00819   -0.01635    0.06504
C12B  1    3.564577    1.831768    1.527864    11.00000    0.10681
0.13421 =
         0.08206   -0.00917   -0.04464    0.05797
AFIX  23
H12C  2    3.656710    1.808460    1.525069    11.00000   -1.20000
H12E  2    3.537932    1.825118    1.591009    11.00000   -1.20000
AFIX   0
C13B  1    3.596449    1.893673    1.514771    11.00000    0.04050
0.20049 =
         0.07305   -0.06076   -0.02857    0.02397
AFIX  13
H13C  2    3.562117    1.915810    1.566091    11.00000   -1.20000
AFIX   0
C14B  1    3.494674    1.910926    1.414224    11.00000    0.03534
0.09536 =
         0.07733   -0.04225    0.00182    0.00974
C15B  1    3.752294    1.904631    1.521895    11.00000    0.10056
0.40120 =
         0.08807   -0.01801   -0.00016   -0.03319
```

```
AFIX 137
H15D  2    3.765980    1.944854    1.509716    11.00000   -1.50000
H15E  2    3.788408    1.882008    1.473607    11.00000   -1.50000
H15F  2    3.813450    1.894833    1.587433    11.00000   -1.50000
AFIX   0
N1B   3    3.289421    1.863757    1.295111    11.00000    0.02921
0.05047 =
         0.03102   -0.01007    0.00015    0.00938
N2B   3    3.395620    1.866421    1.385453    11.00000    0.03732
0.06596 =
         0.03946   -0.01721   -0.00942    0.01585
O1B   4    3.480117    1.824063    1.236003    11.00000    0.03047
0.04814 =
         0.05357   -0.01289    0.01014    0.00494
O2B   4    3.493578    1.952853    1.369672    11.00000    0.03699
0.09952 =
         0.09464   -0.03978    0.01292   -0.01537
O3B   4    3.359614    1.773472    1.428483    11.00000    0.15270
0.07344 =
         0.06390    0.00495   -0.00725    0.05863

C1A   1    2.677077    1.571391    1.540316    11.00000    0.08441
0.09612 =
         0.04059   -0.01974    0.01432   -0.02623
AFIX 137
H1AA  2    2.624730    1.583584    1.474214    11.00000   -1.50000
H1AB  2    2.609586    1.544807    1.564138    11.00000   -1.50000
H1AC  2    2.776161    1.552822    1.539428    11.00000   -1.50000
AFIX   0
C2A   1    2.708821    1.621810    1.606348    11.00000    0.03940
0.07283 =
         0.02530    0.00184    0.01617   -0.00892
C3A   1    2.665417    1.673475    1.580728    11.00000    0.08108
0.08183 =
         0.04455    0.02451    0.01964   -0.00012
AFIX  93
H3AA  2    2.609131    1.681319    1.515789    11.00000   -1.20000
H3AB  2    2.690123    1.703220    1.627028    11.00000   -1.20000
AFIX   0
C4A   1    2.813324    1.608733    1.704843    11.00000    0.03448
0.04050 =
         0.03248   -0.00412    0.01110   -0.00588
C5A   1    2.592546    1.634033    1.781052    11.00000    0.02442
0.03591 =
         0.01995   -0.00010    0.00419   -0.00371
C6A   1    2.484383    1.591204    1.760553    11.00000    0.03730
0.03997 =
         0.03827    0.00081    0.00735   -0.00572
AFIX  43
H6A   2    2.514541    1.554047    1.747400    11.00000   -1.20000
```

```
AFIX   0
C7A   1    2.329602    1.603513    1.759503    11.00000    0.02817
0.05754 =
         0.05444    0.01457    0.00652   -0.01276
AFIX  43
H7A   2    2.253278    1.574369    1.745080    11.00000   -1.20000
AFIX   0
C8A   1    2.285810    1.656169    1.778623    11.00000    0.03001
0.08148 =
         0.02852    0.00459    0.00682    0.00729
AFIX  43
H8A   2    2.179658    1.663640    1.778131    11.00000   -1.20000
AFIX   0
C9A   1    2.393618    1.698631    1.798630    11.00000    0.04447
0.05470 =
         0.05135   -0.02095   -0.00833    0.01595
AFIX  43
H9A   2    2.362062    1.735589    1.811846    11.00000   -1.20000
AFIX   0
C10A  1    2.548164    1.688254    1.799798    11.00000    0.03725
0.03975 =
         0.04093   -0.00890    0.00068    0.00046
AFIX  43
H10A  2    2.623137    1.717874    1.813269    11.00000   -1.20000
AFIX   0
C11A  1    2.871711    1.560383    1.920628    11.00000    0.04104
0.02742 =
         0.04277   -0.00683    0.00802    0.00442
C12A  1    2.995062    1.566168    2.015344    11.00000    0.04533
0.04640 =
         0.05213    0.01341   -0.00052    0.00405
AFIX  23
H12F  2    3.079154    1.537860    2.019388    11.00000   -1.20000
H12G  2    2.948612    1.561224    2.071872    11.00000   -1.20000
AFIX   0
C13A  1    3.059119    1.626639    2.012557    11.00000    0.02690
0.04277 =
         0.03885   -0.00635    0.00048    0.00212
AFIX  13
H13D  2    3.044924    1.647971    2.070814    11.00000   -1.20000
AFIX   0
C14A  1    2.961230    1.654029    1.921307    11.00000    0.02509
0.03712 =
         0.03632   -0.01090    0.00476    0.00135
C15A  1    3.231537    1.627046    2.011890    11.00000    0.03871
0.06675 =
         0.04073    0.00090    0.00391    0.00281
AFIX 137
H15G  2    3.247792    1.602966    1.958861    11.00000   -1.50000
H15H  2    3.293858    1.612661    2.074283    11.00000   -1.50000
```

```
H15I  2    3.264041    1.665772    2.002096    11.00000   -1.50000
AFIX   0
N1A   3    2.754398    1.622929    1.783789    11.00000    0.02332
0.03574 =
         0.02499   -0.00152    0.00464   -0.00239
N2A   3    2.854217    1.613518    1.875783    11.00000    0.02551
0.02798 =
         0.03049   -0.00220    0.00428    0.00058
O1A   4    2.944570    1.588682    1.716560    11.00000    0.03251
0.05521 =
         0.04731   -0.00439    0.01868    0.00160
O2A   4    2.972265    1.700258    1.890345    11.00000    0.03897
0.03472 =
         0.05047   -0.00338    0.00644   -0.00970
O3A   4    2.796976    1.519701    1.886504    11.00000    0.05050
0.03436 =
         0.04495   -0.00360    0.01493   -0.00146

C1C   1    2.840262    1.441798    1.448619    11.00000    0.06827
0.09443 =
         0.02966   -0.01622    0.00820   -0.01118
AFIX 137
H1CA  2    2.741105    1.455972    1.459089    11.00000   -1.50000
H1CB  2    2.893718    1.472203    1.422526    11.00000   -1.50000
H1CC  2    2.906756    1.428660    1.510692    11.00000   -1.50000
AFIX   0
C2C   1    2.808846    1.394293    1.378439    11.00000    0.04355
0.05457 =
         0.02962    0.00365    0.01275   -0.01194
C3C   1    2.858708    1.341441    1.399893    11.00000    0.07482
0.07663 =
         0.03134    0.01826   -0.00148   -0.00663
AFIX  93
H3CA  2    2.918344    1.332471    1.463589    11.00000   -1.20000
H3CB  2    2.834533    1.312824    1.351455    11.00000   -1.20000
AFIX   0
C4C   1    2.704333    1.407475    1.281591    11.00000    0.03558
0.03408 =
         0.03150    0.00249    0.01107   -0.00847
C5C   1    2.923621    1.386390    1.201044    11.00000    0.02627
0.03225 =
         0.02308   -0.00005    0.00331    0.00289
C6C   1    2.970659    1.333655    1.179683    11.00000    0.02841
0.03520 =
         0.04533   -0.00386   -0.00342    0.00148
AFIX  43
H6C   2    2.898387    1.303151    1.167884    11.00000   -1.20000
AFIX   0
C7C   1    3.124657    1.325278    1.175470    11.00000    0.03196
0.05284 =
```

```
         0.07081   -0.01206    0.00026    0.01386
AFIX  43
H7C   2    3.157795    1.289072    1.159501    11.00000   -1.20000
AFIX   0
C8C   1    3.229176    1.369229    1.194341    11.00000    0.02509
0.07066 =
         0.07287   -0.01978    0.00669    0.00361
AFIX  43
H8C   2    3.335217    1.363011    1.193098    11.00000   -1.20000
AFIX   0
C9C   1    3.181478    1.422673    1.215226    11.00000    0.03095
0.04856 =
         0.07725   -0.00825    0.00680   -0.01324
AFIX  43
H9C   2    3.253904    1.453131    1.226920    11.00000   -1.20000
AFIX   0
C10C  1    3.025131    1.431459    1.218969    11.00000    0.03094
0.03192 =
         0.05012   -0.00658    0.00608   -0.00535
AFIX  43
H10C  2    2.990444    1.467708    1.233552    11.00000   -1.20000
AFIX   0
C11C  1    2.552417    1.364250    1.064760    11.00000    0.03036
0.03621 =
         0.03708    0.00079    0.00326    0.00038
C12C  1    2.453135    1.390944    0.974979    11.00000    0.03258
0.04647 =
         0.03528   -0.00048    0.00067    0.00084
AFIX  23
H12H  2    2.341873    1.391495    0.977636    11.00000   -1.20000
H12I  2    2.462172    1.369956    0.916063    11.00000   -1.20000
AFIX   0
C13C  1    2.516674    1.451781    0.973603    11.00000    0.03914
0.03832 =
         0.03278    0.00211    0.00719    0.00213
AFIX  13
H13E  2    2.567696    1.455623    0.918051    11.00000   -1.20000
AFIX   0
C14C  1    2.639410    1.457839    1.068430    11.00000    0.02970
0.03303 =
         0.03511    0.00437    0.00742    0.00328
C15C  1    2.393599    1.496666    0.965246    11.00000    0.06013
0.04918 =
         0.05478    0.00306   -0.00979    0.01854
AFIX 137
H15J  2    2.345413    1.493817    1.020619    11.00000   -1.50000
H15K  2    2.313290    1.491477    0.904376    11.00000   -1.50000
H15L  2    2.441778    1.533996    0.965290    11.00000   -1.50000
AFIX   0
N1C   3    2.762110    1.395656    1.202258    11.00000    0.02601
```

```
0.03490 =
         0.02482    0.00447    0.00206   -0.00191
N2C   3    2.660362    1.405016    1.111961    11.00000    0.02507
0.03557 =
         0.02280    0.00308    0.00004    0.00005
O1C   4    2.573228    1.426003    1.273503    11.00000    0.03605
0.05557 =
         0.04525   -0.00270    0.01884    0.00170
O2C   4    2.711662    1.499485    1.102948    11.00000    0.04728
0.03399 =
         0.03632   -0.00040    0.00718   -0.00039
O3C   4    2.549154    1.316842    1.095215    11.00000    0.04908
0.04031 =
         0.05516    0.00698   -0.01085   -0.00945
HKLF 4

REM  cu_axi20_0m in P2(1)
REM R1 =  0.0610 for    7549 Fo > 4sig(Fo)  and  0.0700 for all
8826 data
REM    749 parameters refined using      1 restraints

END

WGHT      0.0730      1.9153

REM Instructions for potential hydrogen bonds
EQIV $1 x, y, z+1
HTAB C13A O1D_$1
HTAB C10C O2D
EQIV $2 -x+6, y-1/2, -z+2
HTAB C12C O1B_$2
EQIV $3 x, y, z-1
HTAB C13C O3A_$3

REM Highest difference peak  0.503,  deepest hole -0.294,  1-sigma
level  0.052
Q1    1   3.5740  1.8121  1.6046  11.00000  0.05    0.50
Q2    1   3.0554  1.5395  2.0709  11.00000  0.05    0.43
Q3    1   3.0624  1.5727  1.9708  11.00000  0.05    0.36
Q4    1   3.0399  1.7238  1.6067  11.00000  0.05    0.30
Q5    1   3.0272  1.6994  1.5181  11.00000  0.05    0.25
;
_shelx_res_checksum              38044
_shelx_hkl_file
;
  -1   0   0 8.50823 0.99998   5
  -1   0   0 9.64630 0.93628   4
  -1   0   0 9.61772 1.05123   5
  -1   0   0 9.65104 0.86998   4
  -2   0   0 372.460 22.1450   4
```

−2	0	0	350.891	22.0149	5
−2	0	0	361.065	22.1294	4
−2	0	0	366.343	22.8874	2
−2	0	0	366.286	22.0786	3
−2	0	0	344.766	21.9958	5
−3	0	0	13.9800	1.85809	4
−3	0	0	13.5872	1.61828	3
−3	0	0	13.8500	1.76228	4
−3	0	0	11.3315	2.48837	2
−4	0	0	172.308	10.7200	3
−4	0	0	166.411	10.7420	4
−4	0	0	156.745	11.2278	1
−4	0	0	159.866	10.7052	4
−5	0	0	31.3220	2.96691	3
−5	0	0	27.8813	3.02505	4
−6	0	0	87.1173	6.55138	4
−8	0	0	33.1615	3.13468	8
9	0	0	1.31977	3.23207	13
−9	0	0	4.71977	1.33444	8
10	0	0	6.08522	2.78165	13
−1	−1	0	3400.10	214.337	5
−1	−1	0	3502.60	212.789	4
−1	−1	0	3554.59	215.064	5
−1	1	0	3512.91	212.636	3
−1	1	0	3373.92	212.638	3
−1	1	0	3766.77	212.880	4
−1	1	0	3488.97	212.824	4
−2	−1	0	592.201	35.5971	3
−2	−1	0	574.487	35.6510	4
−2	−1	0	589.853	36.3377	2
−2	−1	0	586.380	35.6447	4
−2	−1	0	566.510	35.9582	5
−2	−1	0	606.282	36.1380	5
−2	1	0	599.904	35.5110	4
−2	1	0	573.239	36.2869	2
−2	1	0	569.534	35.5087	4
−2	1	0	572.013	35.4463	3
−3	−1	0	101.871	7.25326	5
−3	−1	0	103.758	8.28152	2
−3	−1	0	102.291	7.26293	4
−3	−1	0	99.5698	7.22996	5
−3	−1	0	103.402	7.26932	4
−3	−1	0	104.632	7.19918	3
−3	1	0	124.904	7.17364	4
−3	1	0	119.963	7.16134	4
−3	1	0	119.866	7.09344	3
−4	−1	0	16.1654	2.34681	4
−4	−1	0	15.6828	3.22674	2
−4	−1	0	15.5517	3.17043	1
−5	−1	0	1.20257	1.71842	1

−5	−1	0	0.57397	1.37805	4
−5	1	0	3.71584	1.29843	3
−5	1	0	2.80866	1.37912	4
−6	−1	0	18.2338	2.91189	4
−6	1	0	22.0778	2.56196	4
−8	−1	0	16.0721	2.44913	8
−8	1	0	23.5257	2.66766	8
8	1	0	18.8342	3.87513	13
−9	−1	0	3.65412	1.50227	8
9	1	0	−2.8929	3.24855	13
10	−1	0	3.47242	2.13337	13
10	1	0	1.76557	2.28696	13
0	2	0	13.4162	1.09505	5
0	2	0	13.2145	0.98290	4
−1	−2	0	1454.31	86.9994	5
−1	2	0	1405.82	86.3582	4
−1	2	0	1422.08	86.4258	4
−2	−2	0	3.92569	0.88360	3
−2	−2	0	3.39989	1.48861	5
−2	−2	0	3.67705	0.91506	4
−2	−2	0	3.83472	1.01498	4
−2	−2	0	3.26013	0.92040	4
−2	−2	0	3.75505	1.51147	5
−2	−2	0	4.52889	1.83945	2
−3	−2	0	2246.21	138.432	3
−3	−2	0	2136.74	138.850	5
−3	−2	0	2356.36	139.106	5
−3	−2	0	2342.51	139.541	2
−3	−2	0	2328.64	138.534	4
−3	2	0	2278.95	138.171	4
−3	2	0	2359.21	138.174	3
−3	2	0	2201.76	138.181	4
−4	−2	0	28.1575	3.04058	4
−4	−2	0	29.8920	4.19484	2
−4	−2	0	27.8819	3.73755	1
−4	−2	0	28.5710	2.81085	3
−4	2	0	23.8026	2.39374	4
−4	2	0	23.4686	2.21688	3
−4	2	0	23.6391	2.35308	4
−5	−2	0	30.2180	3.40611	4
−5	−2	0	33.0581	4.15637	1
−5	2	0	26.8185	2.66588	4
−5	2	0	26.8441	2.62063	4
−5	2	0	30.0472	2.84903	3
−6	−2	0	64.2239	5.24827	4
−6	2	0	61.4483	4.40901	4
−6	2	0	52.7285	4.14124	4
−6	2	0	56.3632	4.87015	3
−7	−2	0	29.7196	3.93449	10
7	2	0	28.8668	5.19295	13

-8	-2	0	10.6367	2.05917	8
-8	2	0	5.73531	1.47342	8
8	2	0	6.44392	3.49821	13
-9	-2	0	22.7525	2.87032	8
9	2	0	16.1582	4.16245	13
10	2	0	11.6252	3.13173	13
0	3	0	2.03040	0.55809	4
0	3	0	2.33513	0.53348	4
-1	-3	0	1418.42	81.2217	5
-1	3	0	1277.59	80.3372	4
-1	3	0	1289.30	80.3701	4
-2	-3	0	21.1420	2.98204	2
-2	-3	0	22.9484	2.84902	5
-2	-3	0	23.3612	3.45126	5
-2	-3	0	22.3472	2.11413	3
-2	-3	0	22.3343	2.29872	4
-2	3	0	22.6516	1.64913	4
-2	3	0	23.1662	1.60452	4
-2	3	0	23.7841	1.55119	3
-3	-3	0	706.153	44.5368	5
-3	-3	0	693.127	44.5478	2
-3	-3	0	686.893	43.6101	4
-3	-3	0	677.224	43.5009	3
-3	3	0	739.012	43.1155	4
-3	3	0	718.045	43.0831	4
-3	3	0	735.658	43.1091	3
-4	-3	0	104.397	8.18361	1
-4	-3	0	99.8897	7.22155	4
-4	-3	0	105.800	8.67729	2
-4	-3	0	106.889	7.49075	5
-4	3	0	92.1995	6.46858	3
-4	3	0	87.0009	6.35837	4
-4	3	0	94.3029	6.39013	4
-5	-3	0	195.850	14.5301	1
-5	-3	0	202.263	15.5728	2
-5	3	0	203.834	12.9436	3
-5	3	0	199.672	12.5934	4
-5	3	0	200.353	12.6038	4
-6	-3	0	164.371	12.6261	1
-6	3	0	181.888	11.6606	3
-7	-3	0	107.251	8.40236	10
7	3	0	99.6723	9.79121	13
-7	3	0	120.070	7.96886	7
-8	-3	0	28.9657	3.65300	8
-8	3	0	28.7460	2.85737	8
8	3	0	34.9274	5.48782	13
-9	-3	0	44.3888	4.08810	8
9	3	0	62.0585	7.04517	13
10	3	0	1.40464	2.39530	13
-1	-4	0	35.9739	4.03294	5

−1	4	0	37.2785	2.86121	4
1	4	0	37.2856	3.31621	4
−1	4	0	39.7591	2.79886	3
−1	4	0	35.7240	2.84530	4
−2	−4	0	45.3675	4.64678	5
−2	−4	0	44.5997	3.76070	4
−2	−4	0	43.2236	4.40157	2
−2	4	0	51.0013	2.96498	3
−3	−4	0	8.86254	1.78143	4
−3	−4	0	9.75708	2.61674	5
−3	−4	0	8.52068	2.51502	2
−3	4	0	15.0620	1.07155	4
−3	4	0	12.8692	1.02761	4
−3	4	0	12.7583	1.20068	3
−4	−4	0	33.8539	4.27911	5
−4	−4	0	37.3221	4.77011	2
−4	−4	0	31.2575	4.87099	1
−4	4	0	36.1099	2.70590	3
−4	4	0	35.8862	2.37537	4
−4	4	0	34.3061	2.37561	4
−5	−4	0	8.29238	3.05403	2
−5	−4	0	7.90091	3.01680	1
−5	4	0	7.97727	1.35890	3
−6	−4	0	7.19030	2.91262	1
−6	4	0	10.6146	1.77444	3
−6	4	0	10.0205	2.09173	7
−6	4	0	9.90640	1.03299	6
−7	−4	0	20.3038	3.53354	10
7	4	0	15.4471	4.77998	13
−7	4	0	28.7426	2.32748	9
−7	4	0	26.2786	3.20808	7
−8	−4	0	4.21070	1.76411	8
−8	−4	0	4.10694	2.31000	10
8	4	0	7.34409	3.78395	13
−9	−4	0	35.4398	3.34108	8
9	4	0	30.4507	4.92136	13
−9	4	0	32.1082	2.49819	9
10	4	0	13.3590	2.87196	13
0	5	0	4.43076	0.93273	4
0	5	0	3.90857	0.94259	4
0	5	0	4.54783	0.80373	3
1	−5	0	41.8089	4.11188	2
−1	−5	0	58.9171	4.55872	2
−1	−5	0	59.0196	4.56820	5
−1	5	0	43.8953	3.12222	4
−1	5	0	45.3689	3.11225	3
−1	5	0	46.9127	3.12506	4
−2	−5	0	248.763	16.6999	5
−2	−5	0	233.342	15.5455	4
−2	−5	0	241.066	16.2120	2

−2	5	0	251.086	14.8064	3
−3	−5	0	10.1579	3.03267	5
−3	5	0	15.1624	1.20626	3
−4	−5	0	249.932	17.0399	5
−4	−5	0	246.437	17.0875	2
−4	−5	0	236.978	17.0512	1
−4	5	0	221.975	14.7948	3
−5	−5	0	261.829	18.8420	2
−5	−5	0	248.399	16.7245	5
−5	−5	0	259.004	18.4576	1
−5	5	0	251.256	16.0693	3
−6	−5	0	4.67573	2.94242	1
−6	5	0	1.48637	1.69258	7
−6	5	0	1.39294	0.57612	6
−6	5	0	1.54434	0.70348	9
−6	5	0	2.24375	1.10782	3
−7	−5	0	214.022	13.2628	10
−7	5	0	168.453	11.6426	9
−7	5	0	170.609	12.4209	7
−7	5	0	178.621	11.5075	6
7	5	0	209.005	14.5270	13
−8	−5	0	68.5184	5.57553	8
−8	−5	0	70.3486	6.09436	10
−8	5	0	58.3886	4.26091	6
8	5	0	71.7575	8.91834	13
−8	5	0	66.1142	5.50200	7
−8	5	0	67.5291	4.49833	9
−9	−5	0	50.3308	4.11286	8
9	5	0	58.6869	6.66293	13
−9	5	0	42.8403	3.24730	9
−9	5	0	45.1127	3.11093	6
0	−6	0	16.4135	2.54108	2
0	6	0	16.2073	1.68323	3
0	6	0	14.7093	1.74436	4
0	6	0	14.5199	1.72817	4
−1	−6	0	407.913	26.1522	5
−1	−6	0	401.981	25.8684	2
1	−6	0	405.779	25.7486	2
1	6	0	400.223	25.2335	3
1	6	0	393.492	25.9007	5
−1	6	0	419.808	24.8930	4
−1	6	0	414.497	24.8874	4
−1	6	0	410.655	24.9255	3
1	6	0	419.784	25.2759	4
−2	−6	0	94.0629	7.54272	2
−2	−6	0	85.1142	8.01434	5
−2	6	0	109.598	6.05951	3
−3	−6	0	15.7598	3.36335	1
−3	−6	0	16.9501	3.30917	2
−3	−6	0	15.2803	3.49577	5

−4	−6	0	55.3450	6.57253	5
−4	−6	0	57.2099	6.19644	2
−4	−6	0	59.7286	6.43130	1
−5	−6	0	99.0487	9.14341	1
−5	−6	0	98.3091	8.26162	5
−5	−6	0	94.8660	9.24151	2
−5	6	0	96.8700	6.56228	3
−6	−6	0	34.3785	5.46219	1
−6	−6	0	38.6829	6.54445	2
−6	6	0	34.5223	3.55995	7
−6	6	0	33.5182	3.07223	3
−6	6	0	33.5076	2.34736	6
−6	6	0	35.1201	2.55154	9
−7	−6	0	134.321	10.7599	10
−7	6	0	152.555	10.2900	7
7	6	0	133.254	11.7623	13
−7	6	0	152.065	9.31806	9
−7	6	0	160.940	9.18720	6
−8	6	0	14.6750	2.41817	7
8	6	0	−0.5473	3.67989	13
−9	−6	0	18.6199	2.77213	8
9	6	0	17.4426	4.01508	13
−9	6	0	23.7433	1.89441	9
−9	6	0	22.6976	1.59828	6
0	−7	0	2.10173	1.81062	2
0	7	0	3.87742	0.95078	4
0	7	0	3.93302	0.89370	3
0	7	0	2.26981	0.87855	4
1	−7	0	128.255	9.29801	2
−1	−7	0	130.449	9.47604	2
−1	7	0	137.087	8.26158	4
−1	7	0	135.517	8.26167	4
1	7	0	125.548	8.65432	4
−1	7	0	138.587	8.33143	3
1	7	0	122.197	8.57740	3
−2	−7	0	88.8690	8.45770	5
−2	−7	0	86.4925	7.74259	2
−2	7	0	113.673	6.38897	3
−2	7	0	110.382	6.42017	3
−3	−7	0	269.204	19.4250	1
−3	−7	0	272.528	19.6454	5
−3	−7	0	279.411	19.1334	2
−3	7	0	294.011	17.1147	3
−4	−7	0	202.162	16.0860	5
−4	−7	0	203.341	15.7870	1
−4	−7	0	205.855	15.6342	2
−4	7	0	233.430	13.2393	3
−5	−7	0	26.3789	5.02637	1
−5	−7	0	32.5367	5.34080	2
−5	−7	0	26.3863	4.39952	5

−5	7	0	22.4749	1.72436	6
−5	7	0	24.0413	2.16250	3
−6	−7	0	8.33562	2.55879	10
−6	−7	0	9.23943	3.83740	2
−6	−7	0	8.38194	4.02947	1
−6	7	0	3.07208	0.61647	6
−6	7	0	3.48866	0.71377	9
−6	7	0	4.17389	1.62917	7
−7	−7	0	73.0813	7.90974	1
−7	−7	0	70.3613	6.86430	10
−7	7	0	81.4410	4.91561	6
−7	7	0	82.4345	5.11592	9
−7	7	0	78.8852	6.26966	7
7	7	0	72.8714	8.30510	13
−8	−7	0	2.75698	1.91652	8
−8	−7	0	6.05482	2.32822	10
8	7	0	7.70060	4.23345	13
−8	7	0	3.75479	0.88177	9
−8	7	0	4.61216	0.67329	6
−8	7	0	4.27216	1.87850	7
−9	−7	0	1.93143	1.54806	8
9	7	0	3.32708	3.26443	13
−9	7	0	0.92963	0.63122	9
−9	7	0	0.39507	0.42246	6
0	−8	0	546.511	35.6335	2
0	8	0	571.406	34.8485	3
0	8	0	577.754	34.8634	4
0	8	0	569.187	34.8514	4
0	8	0	569.201	34.8574	3
1	−8	0	47.4853	4.58566	2
−1	−8	0	60.5965	4.97226	2
−1	8	0	49.6716	3.49994	3
1	8	0	57.3204	3.93986	3
−1	8	0	46.3382	3.48235	4
−1	8	0	48.8284	3.54387	3
−1	8	0	46.8005	3.49025	4
2	−8	0	5031.86	309.161	2
−2	−8	0	5297.46	309.417	2
−2	8	0	4998.55	308.390	3
−2	8	0	5037.45	308.302	3
−3	−8	0	29.3873	4.68511	2
−3	8	0	44.8125	2.89374	3
−3	8	0	44.7267	2.83575	3
−4	−8	0	386.100	26.2275	5
−4	−8	0	386.024	25.8033	1
−4	−8	0	392.699	25.5808	2
−4	8	0	360.367	22.7267	3
−4	8	0	337.253	22.7625	3
−5	−8	0	50.8861	7.24802	1
−5	−8	0	45.8443	6.47916	2

−5	8	0	47.9338	3.48459	3
−5	8	0	45.1585	2.96890	6
−5	8	0	46.1733	3.39078	3
−5	8	0	47.8207	2.98089	6
−6	−8	0	154.184	11.8526	10
−6	−8	0	168.271	14.5205	2
−6	−8	0	168.545	14.2561	1
−6	8	0	157.240	11.0694	7
−6	8	0	168.557	9.98978	6
−6	8	0	160.355	10.1042	9
−7	−8	0	17.2110	4.57193	1
−7	−8	0	18.1441	3.53436	10
−7	8	0	24.6833	1.93257	9
−7	8	0	27.3958	1.73440	6
7	8	0	17.2208	5.14203	13
−7	8	0	26.9648	3.47871	7
−8	−8	0	72.3684	6.49157	10
−8	−8	0	74.9944	6.08616	8
−8	8	0	73.2049	4.83368	9
8	8	0	64.0149	8.33819	13
−8	8	0	76.2961	5.98169	7
−8	8	0	79.7258	4.70746	6
−9	−8	0	2.35502	2.12875	10
−9	−8	0	1.54173	1.53148	8
−9	−8	0	3.79748	1.83262	10
−9	8	0	2.20811	1.07929	7
−9	8	0	2.98587	0.39940	6
−9	8	0	2.76377	0.38816	6
9	8	0	−0.9652	3.21186	13
−9	8	0	2.81347	0.63579	9
−9	8	0	2.16839	0.55605	9
0	−9	0	0.60936	1.59154	2
0	9	0	0.73391	0.77454	3
0	9	0	0.38193	0.95093	4
0	9	0	0.15079	1.01537	4
−1	−9	0	115.246	9.53740	2
1	−9	0	127.281	9.65736	2
1	9	0	127.671	8.82667	4
−1	9	0	136.458	8.36801	4
−1	9	0	135.759	8.41325	3
−1	9	0	138.599	8.40226	4
2	−9	0	462.117	29.9334	2
−2	9	0	463.646	28.4923	3
−2	9	0	469.691	28.4561	3
3	−9	0	306.246	20.4032	2
−3	−9	0	292.832	20.6983	2
−3	−9	0	277.156	20.9403	1
−3	9	0	301.819	18.3728	3
−3	9	0	310.564	18.3279	3
−4	−9	0	60.8486	7.86882	5

-4	-9	0	66.5014	7.07087	2
-4	-9	0	61.3225	7.31747	1
-4	9	0	64.5814	4.32545	3
-4	9	0	65.5053	4.23693	3
-5	-9	0	4.96368	3.53335	2
-5	-9	0	3.12333	3.69761	1
-5	9	0	7.39357	0.66573	6
-5	9	0	8.04569	0.73141	6
-5	9	0	6.63485	1.07060	3
-5	9	0	7.58475	1.78162	7
-5	9	0	7.23130	1.10064	3
-6	-9	0	8.54659	2.81280	10
-6	-9	0	6.06535	3.72975	2
-6	-9	0	5.62323	3.66120	1
6	9	0	10.9788	4.66746	13
-7	-9	0	87.6816	9.59431	1
-7	-9	0	83.4159	7.71671	10
-7	-9	0	78.3596	6.87141	8
-7	9	0	94.3256	6.97509	7
-7	9	0	93.3049	5.54298	6
7	9	0	84.1436	9.16672	13
-7	9	0	92.9620	5.68675	9
-8	-9	0	86.0209	6.71135	8
-8	-9	0	84.3429	7.25099	10
-8	9	0	82.5045	5.31542	6
-8	9	0	88.7621	5.31758	6
-8	9	0	84.8241	6.58578	7
-8	9	0	84.4808	5.45204	9
8	9	0	86.6297	8.94024	13
-9	-9	0	32.3711	3.59552	10
-9	-9	0	31.9772	3.23923	8
-9	-9	0	34.6557	3.51148	10
9	9	0	34.3331	5.40300	13
-9	9	0	41.0448	2.49106	9
-9	9	0	43.7661	3.48655	7
-9	9	0	40.5991	2.49225	9
0	-10	0	7.95365	2.65838	2
0	10	0	7.29134	1.33269	4
0	10	0	7.82383	1.32361	3
1	-10	0	54.0309	5.36416	2
-1	-10	0	53.5515	5.74669	2
-1	10	0	59.7407	4.13507	3
-1	10	0	53.9401	4.09738	4
1	10	0	52.5570	4.72421	4
-1	10	0	56.7257	4.13460	3
-1	10	0	63.2691	4.12844	4
-2	-10	0	55.4608	5.61018	2
2	-10	0	54.2485	5.46550	2
-2	10	0	51.7921	3.79296	3
-2	10	0	53.8765	3.72164	3

-3	-10	0	147.127	11.1700	2
3	-10	0	136.700	10.6421	2
-3	-10	0	132.321	11.7335	1
-3	10	0	129.053	8.56576	3
-3	10	0	134.566	8.51933	3
-4	-10	0	46.1945	6.63660	1
-4	-10	0	45.9594	6.25121	2
4	-10	0	55.7250	6.00406	2
-4	10	0	48.4256	3.44577	3
-4	10	0	51.0469	3.37970	3
5	-10	0	45.3145	6.91141	2
-5	-10	0	54.7805	7.08634	2
-5	-10	0	52.8065	7.40480	1
-5	10	0	45.4720	2.91404	6
-5	10	0	50.4273	4.21237	7
-5	10	0	44.8825	3.22193	3
-5	10	0	43.0568	2.90381	6
-5	10	0	46.3185	3.03070	9
-5	10	0	45.9473	3.30884	3
-6	-10	0	2.15678	3.38419	1
-6	-10	0	-2.3381	2.47167	10
-6	10	0	0.03783	0.52696	9
-6	10	0	-1.7647	1.67382	7
-6	10	0	0.62992	0.40627	6
6	10	0	-2.8070	4.63568	13
-6	10	0	0.70323	0.36277	6
-7	-10	0	19.7925	3.63082	10
7	10	0	15.5241	4.32468	13
-7	10	0	12.2570	1.02261	6
-7	10	0	11.1378	0.98474	6
-7	10	0	11.0713	1.16376	9
-7	10	0	12.0277	2.50206	7
-8	-10	0	12.1542	3.04937	10
-8	-10	0	12.7217	2.56458	8
-8	10	0	18.2319	1.24473	6
-8	10	0	18.2420	1.24444	6
8	10	0	15.1857	4.14269	13
-8	10	0	21.3358	3.05413	7
-8	10	0	18.2841	1.44208	9
-9	-10	0	1.83152	1.62633	10
-9	-10	0	0.99319	1.43190	8
-9	-10	0	0.60883	1.85607	10
9	10	0	0.44893	3.23726	13
0	-11	0	2.91102	2.51751	2
0	11	0	2.46184	1.07737	4
0	11	0	1.92883	1.17825	3
1	-11	0	77.4376	6.78577	2
-1	-11	0	61.1260	6.82491	2
-1	11	0	80.1543	5.21618	4
-1	11	0	77.9764	5.23131	4

-1	11	0	79.8886	5.37632	3
-2	-11	0	59.8174	6.65183	2
2	-11	0	81.2283	7.02771	2
-2	11	0	79.3803	5.04166	3
-3	-11	0	78.0211	7.79795	2
-3	-11	0	74.4455	8.33128	1
3	-11	0	82.6614	7.48175	2
-3	11	0	84.8813	5.44151	3
-3	11	0	81.9793	5.34559	3
4	-11	0	55.2730	6.32584	2
-4	-11	0	49.0834	7.08557	1
-4	-11	0	47.0395	6.51880	2
-4	11	0	55.0084	3.30281	6
-4	11	0	54.3641	3.67933	3
-4	11	0	52.9069	3.60138	3
-5	-11	0	329.491	24.7108	1
-5	-11	0	332.389	24.3368	2
-5	11	0	332.577	21.1559	7
-5	11	0	330.956	20.1337	6
-5	11	0	329.158	20.2077	9
-5	11	0	328.296	20.2298	9
-5	11	0	348.062	20.1296	6
-5	11	0	322.217	20.3964	3
-6	-11	0	4.73040	4.75051	1
-6	-11	0	5.67326	2.76450	10
6	11	0	5.85169	3.70154	13
-6	11	0	0.63560	1.70980	7
-6	11	0	0.48044	0.33276	6
-6	11	0	-2.5391	2.40022	12
-6	11	0	0.31211	0.44723	9
-6	11	0	0.16736	0.31771	6
-7	-11	0	39.8250	5.44472	10
-7	-11	0	35.1193	6.63423	1
-7	11	0	41.9506	2.61770	6
-7	11	0	45.6245	4.42785	7
-7	11	0	42.1750	2.62475	6
-7	11	0	42.1108	2.75339	9
7	11	0	32.0746	5.22849	13
-8	-11	0	3.13145	2.49676	10
-8	-11	0	3.41600	1.97186	8
-8	11	0	1.65600	0.45661	9
-8	11	0	1.81884	0.54882	9
8	11	0	2.73430	3.42019	13
-8	11	0	2.25260	1.72070	7
-9	-11	0	6.68705	1.80338	10
-9	-11	0	6.75269	2.09943	10
9	11	0	5.33938	3.08755	13
0	-12	0	283.462	19.2312	2
0	12	0	274.680	17.8546	3
0	12	0	281.097	17.8000	4

1	−12	0	82.6964	7.44676	2
−1	−12	0	81.2708	7.68987	2
−1	12	0	82.3968	5.73557	4
−1	12	0	83.6040	5.72226	4
−1	12	0	87.7431	5.93339	3
−2	−12	0	13.4324	3.19448	2
2	−12	0	16.7445	3.34227	2
−2	12	0	17.2118	1.89939	3
−2	12	0	16.6353	1.75315	3
−3	−12	0	16.6030	3.76125	2
3	−12	0	15.0490	3.81275	2
−3	12	0	16.5351	1.64218	3
−3	12	0	14.2976	1.52878	3
−4	−12	0	16.6094	4.04108	2
−4	−12	0	16.1757	4.65584	1
−4	12	0	14.5692	1.51676	3
−4	12	0	16.2503	2.50289	7
−4	12	0	15.4183	1.11335	6
−4	12	0	15.8891	1.44049	3
−4	12	0	15.8818	1.08809	6
−5	−12	0	45.7621	5.50132	10
−5	−12	0	35.3374	6.93305	1
−5	−12	0	38.4898	6.98737	2
−5	12	0	30.8034	2.25408	9
−5	12	0	28.3740	3.64896	7
−5	12	0	32.9046	2.10503	6
−5	12	0	31.7814	2.11294	6
−5	12	0	33.9924	2.28612	9
−6	−12	0	52.3741	9.01994	1
−6	−12	0	52.1020	5.32596	10
−6	12	0	47.6575	2.93345	6
6	12	0	42.3081	7.35808	13
−6	12	0	42.6789	5.31679	12
−6	12	0	45.1015	3.09573	9
−6	12	0	46.1942	2.92257	6
−6	12	0	45.7653	3.05694	9
−6	12	0	46.5986	4.60554	7
−7	−12	0	8.31767	4.16452	1
−7	−12	0	2.74139	2.95015	10
−7	12	0	4.76416	0.44556	6
−7	12	0	5.54641	1.78177	7
7	12	0	2.30968	3.73317	13
−7	12	0	5.15847	0.52142	6
−7	12	0	3.42355	0.75215	9
−7	12	0	4.86870	0.61338	9
−8	−12	0	8.99233	2.20535	8
−8	12	0	5.58179	1.92326	7
8	12	0	3.24659	3.81787	13
−9	−12	0	1.66363	1.59710	10
−9	−12	0	2.92833	2.00283	10

9	12	0	3.58094	2.74587	13
0	−13	0	−1.6277	2.38878	2
0	13	0	0.50397	1.19382	4
−1	−13	0	0.18573	1.82554	2
1	−13	0	0.66855	2.43662	2
−1	13	0	0.80517	0.90362	4
−1	13	0	−0.0399	0.75795	4
−1	13	0	0.32317	0.94816	3
−2	−13	0	139.691	11.0463	2
−2	13	0	133.189	8.94965	3
−3	−13	0	7.86893	3.11676	2
−3	13	0	4.43963	1.00084	3
−3	13	0	5.71451	1.02280	3
−4	−13	0	353.756	25.1567	2
−4	−13	0	349.711	25.9520	1
−4	13	0	327.183	21.5098	3
−4	13	0	350.809	22.2287	7
−4	13	0	354.445	21.4959	3
−4	13	0	350.335	21.2208	6
−4	13	0	358.677	21.3486	9
−4	13	0	350.811	21.1988	6
−5	−13	0	1.78816	2.67122	10
−5	−13	0	3.26892	3.67978	2
−5	−13	0	1.62589	3.84525	1
−5	13	0	5.38374	1.89587	7
−5	13	0	5.80168	0.59422	6
−5	13	0	5.81150	0.70945	9
−5	13	0	5.58228	0.71693	9
−5	13	0	5.13556	2.77600	12
−5	13	0	5.78290	0.57883	6
−6	−13	0	39.4655	5.24939	10
−6	−13	0	41.1612	8.62364	1
−6	13	0	25.5018	1.73631	6
−6	13	0	29.1893	4.58359	12
−6	13	0	25.2604	1.87173	9
6	13	0	32.1014	5.66471	13
−6	13	0	26.2041	1.93083	9
−6	13	0	27.4723	1.75544	6
−6	13	0	27.4458	3.70380	7
−7	−13	0	5.28268	3.00381	10
−7	13	0	3.40701	0.43587	9
7	13	0	3.29744	3.18338	13
−7	13	0	4.85959	1.66261	7
−7	13	0	3.55592	0.51593	9
−8	−13	0	24.0909	3.38065	10
8	13	0	31.9320	5.14300	13
0	−14	0	168.404	13.0454	2
0	14	0	169.078	11.2996	4
−1	−14	0	61.6308	7.05101	2
−1	14	0	69.4354	4.92058	4

−1	14	0	72.1764	5.26726	3
−2	−14	0	33.4747	5.31086	2
−2	14	0	32.3499	2.94949	3
−3	−14	0	409.468	26.9757	2
−3	14	0	367.016	23.6939	3
−3	14	0	374.455	23.1885	6
−4	−14	0	159.150	14.9711	1
−4	−14	0	144.893	13.8063	2
−4	14	0	169.505	10.2093	6
−4	14	0	172.272	10.3782	9
−4	14	0	164.178	10.5276	3
−4	14	0	178.470	10.1903	6
−4	14	0	165.958	11.2649	7
−5	−14	0	91.3140	8.45534	10
−5	−14	0	101.266	11.2013	1
−5	14	0	87.3568	5.73533	9
−5	14	0	93.1606	5.61570	6
−5	14	0	87.9994	7.77872	12
−5	14	0	94.3845	7.04052	7
−5	14	0	91.1765	5.61222	6
−5	14	0	89.4648	5.75145	9
−6	−14	0	53.5137	8.80411	1
−6	−14	0	50.3768	6.40235	10
−6	14	0	58.3151	4.94290	7
−6	14	0	60.5797	3.63751	9
−6	14	0	57.7031	3.67599	9
−7	−14	0	42.6229	5.57298	10
−7	14	0	38.9582	3.87842	7
−8	−14	0	15.5262	2.71192	10
0	15	0	0.67708	1.33527	4
−1	−15	0	7.91262	5.09693	2
−1	15	0	8.42529	1.68826	3
−1	15	0	6.61446	1.40197	4
−1	15	0	5.71799	1.32568	4
−2	−15	0	110.065	9.73116	2
−2	15	0	104.504	7.45221	3
−3	−15	0	116.534	11.7903	2
−3	15	0	135.564	8.83825	3
−3	15	0	142.620	8.23634	6
−3	15	0	132.060	8.45475	9
−4	−15	0	24.3238	5.67841	2
−4	−15	0	30.3054	8.31587	1
−4	15	0	28.2694	2.23438	9
4	15	0	22.4647	4.62952	10
−4	15	0	27.8608	3.47236	7
−4	15	0	27.5345	1.96110	6
−4	15	0	38.5970	5.32476	12
−4	15	0	32.0928	1.94539	6
−5	−15	0	12.5625	5.15885	1
−5	15	0	11.5533	1.16235	9

−5	15	0	12.2952	0.89449	6
−5	15	0	10.8824	1.06637	9
−5	15	0	12.2627	3.74192	12
−5	15	0	11.8725	2.43757	7
−5	15	0	11.3315	0.88733	6
−6	−15	0	143.995	13.7652	1
−6	−15	0	138.688	10.7095	10
−6	15	0	122.075	7.82744	9
−6	15	0	122.986	7.82611	9
−6	15	0	127.104	9.31255	7
−7	−15	0	18.1164	3.76920	10
−8	−15	0	21.8267	3.61755	10
0	16	0	520.329	32.6724	4
−1	16	0	4.34009	1.24210	4
−1	16	0	6.26161	1.48366	4
−2	16	0	140.108	8.80271	6
−2	16	0	144.475	9.72294	3
3	16	0	75.3602	8.19774	10
−3	16	0	59.9346	4.27964	9
−3	16	0	59.4679	3.94647	6
−4	−16	0	105.160	8.83586	13
4	16	0	111.071	10.2161	10
−4	16	0	110.294	6.92001	6
−4	16	0	111.401	6.94919	6
−4	16	0	121.033	7.21464	9
−5	−16	0	16.4655	5.62935	1
5	16	0	13.5434	4.55480	10
−5	16	0	15.5140	1.10380	6
−5	16	0	16.2937	1.28956	9
−5	16	0	15.3556	1.36869	9
−5	16	0	16.3753	1.10353	6
−6	−16	0	29.4942	6.87913	1
−6	−16	0	35.9607	4.80094	10
−7	−16	0	210.925	14.9510	10
−8	−16	0	16.4265	2.59333	10
0	17	0	1.80774	1.43121	4
−1	17	0	262.493	16.6411	4
−2	17	0	69.9340	4.83496	9
−2	17	0	68.5915	5.45857	3
−3	−17	0	40.2088	4.64090	13
3	17	0	40.4597	6.32807	10
−3	17	0	40.9351	2.68278	6
−3	17	0	39.2742	3.15269	9
−4	−17	0	76.1980	7.28184	13
−4	17	0	99.9702	5.71003	6
4	17	0	78.9825	9.02727	10
−4	17	0	95.2755	5.74201	6
−4	17	0	98.5366	6.00415	9
−5	−17	0	11.8616	5.22426	1
−5	17	0	8.93925	0.69446	6

-5	17	0	8.52956	0.92044	9
-5	17	0	8.40046	0.67228	6
5	17	0	9.05574	4.25651	10
-5	17	0	7.79460	1.06071	9
-6	-17	0	145.443	11.5467	10
-7	-17	0	4.93684	2.21281	10
-8	-17	0	3.86773	2.01171	10
-8	-17	0	4.67535	2.34806	10
1	18	0	30.7291	4.94284	10
-1	18	0	37.1241	3.52672	11
2	18	0	66.4302	7.03439	10
-2	18	0	57.3808	4.54527	9
-3	-18	0	36.4636	5.36024	13
3	18	0	52.2905	6.93681	10
-3	18	0	41.2498	2.79352	6
-3	18	0	43.5898	3.34662	9
-4	-18	0	5.82541	2.39101	13
-4	18	0	11.4583	0.93737	6
-4	18	0	11.5643	1.45975	9
4	18	0	4.90722	3.70989	10
-4	18	0	9.60553	0.99888	6
-5	-18	0	23.0435	6.09391	1
-5	18	0	16.4203	1.33125	9
5	18	0	26.0192	5.63432	10
-5	18	0	14.0812	1.41900	9
-5	18	0	18.5144	1.12766	6
-5	18	0	16.8993	1.12170	6
-6	-18	0	16.7218	3.16433	10
6	18	0	15.9102	3.73525	10
-7	-18	0	4.33004	2.67908	10
0	19	0	-0.6468	2.11600	10
-1	-19	0	4.97690	2.94753	13
-1	19	0	1.14614	1.34746	11
1	19	0	6.96821	3.40551	10
-2	-19	0	226.953	15.0219	13
-2	19	0	183.784	13.8285	9
2	19	0	242.478	15.9136	10
-3	-19	0	133.966	10.3980	13
-3	19	0	135.088	8.41240	6
3	19	0	139.683	11.4971	10
-3	19	0	135.170	8.84337	9
-4	-19	0	19.7495	3.19153	13
4	19	0	22.5737	4.81536	10
-4	19	0	17.8885	1.87909	9
-4	19	0	20.5781	1.43062	6
-4	19	0	17.5837	1.41673	6
-5	19	0	38.5328	2.62407	9
5	19	0	37.3488	6.54706	10
-5	19	0	41.2806	2.59148	9
6	19	0	56.3471	7.01563	10

-7	-19	0	7.89409	2.67174	10
0	-20	0	5.91716	2.73010	13
-1	-20	0	34.4953	4.84607	13
-1	20	0	29.0577	2.91909	11
1	20	0	32.5828	5.22459	10
-2	-20	0	35.8467	5.12733	13
2	20	0	30.7250	6.16035	10
-3	-20	0	121.290	8.98970	13
-3	20	0	111.093	7.47912	9
-3	20	0	104.534	7.01173	6
3	20	0	117.466	10.0487	10
-4	-20	0	0.23845	2.90947	13
-4	20	0	1.69301	0.82838	9
4	20	0	6.51928	3.93142	10
-4	20	0	1.19781	0.46123	6
-4	20	0	1.22831	0.47192	6
-5	20	0	3.26133	0.41913	9
5	20	0	3.56604	3.70639	10
-5	20	0	3.75375	0.44939	9
6	20	0	28.7206	5.43030	10
0	-21	0	-0.5328	2.03505	13
0	21	0	0.58426	1.87417	11
-1	-21	0	1.04330	2.75670	13
-1	21	0	4.82998	1.13784	11
1	21	0	1.44385	2.69757	10
-1	21	0	5.15206	1.11316	11
-2	-21	0	46.7602	5.34616	13
2	21	0	44.6287	5.78212	10
-3	-21	0	0.99757	2.46968	13
-3	21	0	1.40221	1.14753	9
3	21	0	2.26794	3.29262	10
-3	21	0	2.31426	0.53732	6
-4	-21	0	17.1713	3.09278	13
-4	21	0	14.4354	1.64005	9
4	21	0	9.55709	4.06083	10
-4	21	0	17.6655	1.23460	6
-4	21	0	14.8919	1.22232	6
5	21	0	14.0904	4.21171	10
6	21	0	7.27592	3.33328	10
0	-22	0	118.828	9.18473	13
0	22	0	120.077	8.43562	11
-1	-22	0	276.844	17.2698	13
-1	22	0	247.596	15.6383	11
1	22	0	264.298	17.3369	10
-1	22	0	236.285	15.6361	11
-2	-22	0	18.9031	4.06563	13
2	22	0	25.2690	4.26579	10
-3	-22	0	0.75319	2.23136	13
-3	22	0	1.71359	0.95079	9
-3	22	0	2.33741	0.51879	6

3	22	0	1.05037	2.82345	10
-4	-22	0	1.56880	2.28440	13
-4	22	0	4.79669	0.84591	9
4	22	0	3.66331	2.43779	10
-4	22	0	4.65495	0.51011	6
-4	22	0	4.06514	0.49449	6
5	22	0	44.0276	5.15520	10
6	22	0	23.8341	4.21430	10
0	23	0	1.09423	1.22057	11
-1	-23	0	1.99065	3.10635	13
1	23	0	1.51059	2.18419	10
-2	-23	0	202.046	14.0381	13
2	23	0	202.825	14.5627	10
-3	-23	0	47.5195	5.32705	13
-3	23	0	47.5708	3.61009	9
-3	23	0	50.6317	3.22874	6
3	23	0	49.0094	5.80807	10
-4	-23	0	2.87542	2.08139	13
-4	23	0	3.05436	0.38820	6
-4	23	0	2.54179	0.37825	6
-4	23	0	2.87687	0.72937	9
4	23	0	3.48011	2.17531	10
5	23	0	5.81839	2.24679	10
0	-24	0	69.5334	6.67951	13
0	24	0	58.9148	4.44120	11
0	24	0	67.4249	4.59820	11
-1	-24	0	5.10330	2.42065	13
1	24	0	7.27131	1.80724	11
-2	-24	0	3.95384	2.33246	13
2	24	0	4.30693	2.77265	10
3	24	0	4.45291	2.66442	10
-3	24	0	7.72178	1.15218	9
-3	24	0	7.41109	0.75951	6
-4	24	0	17.9717	1.37667	9
4	24	0	12.9088	3.21850	10
-4	24	0	16.4422	1.25899	9
5	24	0	0.94522	2.27903	10
0	-25	0	-1.3200	2.06842	13
1	-25	0	6.74818	2.16008	13
-1	-25	0	4.94949	2.35418	13
1	25	0	2.58384	1.53356	11
-2	-25	0	5.83449	2.29546	13
2	25	0	6.60526	2.38121	10
-3	-25	0	3.30153	2.02011	13
-3	25	0	1.63256	0.83165	9
3	25	0	3.08620	2.60066	10
-3	25	0	2.47187	0.44317	6
4	25	0	16.8372	3.24470	10
0	-26	0	6.23513	2.18124	13
-1	-26	0	10.0694	2.40668	13

1	−26	0	8.99563	2.25256	13
1	26	0	13.1587	1.57862	11
1	26	0	12.6846	1.51335	11
−2	−26	0	4.82160	1.99806	13
2	26	0	3.12763	1.25251	11
−2	26	0	1.65052	0.95385	9
−3	−26	0	3.55548	2.08442	13
−3	26	0	3.50359	0.95440	9
3	26	0	1.96831	1.76526	10
0	−27	0	−0.3206	2.01451	13
−1	−27	0	4.51021	2.17602	13
1	−27	0	2.36085	1.89815	13
−2	−27	0	−0.5533	1.93689	13
2	27	0	−0.3967	0.73255	11
2	27	0	−0.0844	0.74219	11
0	−28	0	1.58931	1.45032	13
−1	−28	0	19.6635	2.79149	13
10	0	−1	18.6846	3.61157	13
−9	0	1	80.8939	5.73994	8
9	0	−1	77.4446	7.62922	13
8	0	−1	3.44109	3.66899	13
−7	0	1	5.34650	1.44260	11
−6	0	1	36.4507	3.74458	4
−5	0	1	94.9577	7.48327	1
−5	0	1	100.805	7.09098	4
−4	0	1	204.678	12.8154	4
−4	0	1	189.268	13.4940	1
−4	0	1	200.823	12.7626	3
−3	0	1	57.5930	4.06383	4
−3	0	1	53.0364	3.84909	3
−3	0	1	53.5489	3.67717	5
−3	0	1	57.5281	4.05452	4
−3	0	1	52.9503	3.71521	5
−2	0	1	27.1327	2.82626	5
−2	0	1	28.2286	2.55844	5
−2	0	1	28.8457	2.41058	4
−2	0	1	25.9099	3.17163	2
−2	0	1	27.6118	2.21827	3
−2	0	1	27.2549	2.35336	4
−1	0	1	−8.5016	0.43644	4
−1	0	1	−8.7742	0.73502	5
−1	0	−1	−5.7904	0.65089	3
−1	0	−1	−5.1567	0.87270	5
−1	0	−1	−5.1331	0.75533	4
−1	0	−1	−5.8418	0.87935	5
−1	0	−1	−5.1967	0.84308	4
−2	0	−1	245.878	14.8947	4
−2	0	−1	252.669	14.8932	3
−2	0	−1	233.812	14.9587	4
−2	0	−1	240.250	15.7447	2

−2	0	−1	228.282	14.8584	3
−3	0	−1	18.7789	3.57604	2
−3	0	−1	20.8113	2.04872	3
−3	0	−1	20.8752	2.24120	4
−3	0	−1	21.8234	2.07893	4
−4	0	−1	594.036	36.4193	4
−4	0	−1	597.917	36.3963	3
−4	0	−1	550.251	36.6226	1
−4	0	−1	571.589	36.3010	4
−4	0	−1	635.361	38.2591	2
−5	0	−1	2.47654	1.31181	3
−5	0	−1	1.54626	1.27439	4
−8	0	−1	0.99025	1.22409	8
−9	0	−1	1.52359	1.08118	8
9	0	1	1.21523	2.90241	13
10	−1	−1	8.90421	3.10362	13
10	1	−1	9.12912	3.31044	13
9	−1	−1	130.168	10.9446	13
−9	−1	1	141.005	9.14711	8
9	1	−1	131.829	10.7937	13
−9	1	1	136.797	8.90874	8
−8	1	1	233.274	14.7753	7
8	1	−1	185.838	15.1577	13
−7	−1	1	25.0952	3.59330	10
−6	−1	1	94.2591	6.97310	4
−6	1	1	87.0517	6.61474	4
−5	−1	1	304.843	19.4093	1
−5	−1	1	305.085	18.6435	4
−5	1	1	279.950	18.3517	4
−5	1	1	266.248	18.3530	3
−4	−1	1	7.13887	1.63243	4
−4	−1	1	8.04672	1.50220	3
−4	−1	1	5.24365	2.27293	1
−4	1	1	5.35932	1.37963	4
−4	1	1	5.66069	1.32478	4
−4	1	1	5.23383	1.21984	3
−3	−1	1	410.704	26.1103	5
−3	−1	1	382.951	25.5858	4
−3	−1	1	396.150	26.5666	2
−3	−1	1	394.321	25.5342	3
−3	−1	1	406.136	25.6528	4
−3	−1	1	392.588	25.8994	5
−3	1	1	450.210	25.6033	4
−3	1	1	458.364	25.5296	4
−3	1	1	429.826	25.4520	3
−2	−1	1	272.282	16.6930	3
−2	−1	1	252.625	16.7667	4
−2	−1	1	275.281	17.4362	2
−2	−1	1	279.123	16.8021	4
−2	−1	1	255.781	17.1184	5

−2	−1	1	283.847	17.5136	5
−1	−1	1	180.955	13.5424	5
−1	1	1	225.996	13.0593	3
−1	1	1	219.120	13.0297	4
−1	1	1	214.440	13.0876	4
−1	1	1	216.303	13.2714	5
0	−1	−1	203.439	11.8869	5
0	1	−1	177.333	11.6060	4
0	1	1	200.585	11.9880	5
0	1	−1	177.318	11.7139	5
−1	−1	−1	125.301	8.12839	5
−1	−1	−1	125.044	7.83137	4
−1	−1	−1	113.103	8.14116	5
−1	−1	−1	125.912	7.75995	4
−1	−1	−1	117.117	7.71313	3
−1	1	−1	118.413	7.55780	3
−1	1	−1	126.695	7.60647	4
−1	1	−1	126.964	7.57058	3
−1	1	−1	129.949	7.66978	4
−2	−1	−1	24.3515	2.03180	4
−2	−1	−1	24.2234	2.44187	5
−2	−1	−1	25.9041	1.87607	3
−2	−1	−1	25.4155	3.28802	2
−2	−1	−1	24.5124	2.12208	4
−2	−1	−1	26.5221	2.01513	3
−2	1	−1	17.8375	1.82162	4
−2	1	−1	17.9729	1.66524	4
−2	1	−1	17.5065	1.60628	3
−2	1	−1	18.2541	1.62365	3
−2	1	−1	18.5800	2.94196	2
−2	1	−1	16.7613	1.63983	4
−3	−1	−1	710.710	43.4544	2
−3	−1	−1	692.387	42.3707	4
−3	−1	−1	672.051	42.2209	4
−3	−1	−1	689.990	42.2715	3
−3	−1	−1	683.386	42.2537	4
−3	1	−1	688.259	42.1722	4
−3	1	−1	694.551	42.1241	3
−3	1	−1	669.523	42.0857	4
−3	1	−1	705.241	43.3968	2
−4	−1	−1	25.2566	2.84983	4
−4	−1	−1	23.7153	3.62106	1
−4	−1	−1	24.7996	2.51869	3
−4	−1	−1	23.5331	3.98529	2
−4	1	−1	24.8756	2.60562	4
−4	1	−1	24.4311	2.30050	4
−4	1	−1	27.5192	2.47426	3
−5	−1	−1	271.656	16.5881	4
−5	1	−1	249.735	16.0584	4
−5	1	−1	245.762	16.1514	4

−5	1	−1	245.466	16.2764	3
−6	1	−1	83.8760	6.03319	4
−8	−1	−1	8.52999	1.74500	8
−8	1	−1	7.04114	1.63013	8
−9	−1	−1	4.44576	1.38750	8
9	1	1	1.55554	3.11751	13
10	−2	−1	4.26736	2.45473	13
10	2	−1	6.55643	2.70899	13
−9	−2	1	41.2287	3.86456	8
−9	2	1	53.9320	3.58416	8
9	2	−1	36.0009	6.40359	13
8	2	−1	52.9527	7.06020	13
−8	2	1	73.0677	5.43788	7
−7	−2	1	46.7041	4.95000	10
7	2	−1	59.3413	6.83518	13
7	2	−1	46.0681	6.80189	13
−6	−2	1	5.77099	2.52056	1
−5	−2	1	285.894	18.6605	4
−5	−2	1	288.911	19.6673	1
−5	2	1	298.505	18.3709	3
−5	2	1	292.959	18.2473	4
−5	2	1	275.287	18.2639	4
−4	−2	1	589.200	37.0377	5
−4	−2	1	636.116	38.4991	2
−4	−2	1	628.500	37.2464	5
−4	−2	1	625.423	38.1141	1
−4	−2	1	611.478	36.9992	4
−4	2	1	562.668	36.5750	4
−4	2	1	559.254	36.6769	4
−4	2	1	558.829	36.5846	3
−3	−2	1	60.2439	4.50864	4
−3	−2	1	52.8165	4.89112	5
−3	−2	1	61.9588	5.46869	5
−3	−2	1	58.9535	5.57765	2
−3	−2	1	60.4861	4.25691	3
−3	2	1	55.2600	4.15308	4
−3	2	1	58.3184	4.04263	4
−3	2	1	58.3955	4.00806	3
−2	−2	1	956.125	59.5768	2
−2	−2	1	969.126	59.0145	4
−2	−2	1	990.402	59.9064	5
−2	−2	1	870.734	59.4192	5
−2	−2	1	914.131	58.9233	3
−2	2	1	983.960	58.7695	3
−2	2	1	1051.98	58.8182	4
−2	2	1	1012.33	58.8789	4
−1	2	1	38.9124	2.86042	3
−1	2	1	41.4628	3.02062	4
−1	2	1	43.7295	2.92739	4
−1	2	1	41.9770	2.84560	5

0	−2	−1	77.1815	5.74171	5
0	2	−1	85.4410	5.39737	4
0	2	−1	79.0699	5.37279	4
0	2	1	83.5436	5.86500	5
0	2	−1	82.4754	5.39495	5
−1	−2	−1	23.6937	2.65291	5
−1	−2	−1	22.2044	2.03234	4
−1	2	−1	19.5482	1.43333	4
1	2	1	24.3950	2.72174	5
−1	2	−1	20.5162	1.48840	4
−2	−2	−1	507.645	30.4840	5
−2	−2	−1	487.087	30.4480	5
−2	−2	−1	511.833	30.6520	2
−2	−2	−1	488.075	29.8230	3
−2	−2	−1	510.660	29.9690	4
−2	−2	−1	499.722	29.8394	4
−2	2	−1	464.543	29.5105	4
−2	2	−1	461.469	29.5570	4
−2	2	−1	462.687	29.5020	3
−2	2	−1	464.176	29.5093	3
−3	−2	−1	97.2908	6.77018	4
−3	−2	−1	89.6018	6.92108	5
−3	−2	−1	99.3862	6.62378	3
−3	−2	−1	96.7863	7.79021	2
−3	−2	−1	102.089	7.06707	5
−4	−2	−1	141.656	9.73000	1
−4	−2	−1	132.339	8.72124	3
−4	−2	−1	147.266	10.4044	2
−4	−2	−1	141.862	8.92656	4
−4	2	−1	102.868	8.20884	3
−4	2	−1	105.778	8.16605	4
−5	2	−1	9.92613	1.57282	4
−5	2	−1	11.0490	1.73668	3
−5	2	−1	10.7141	1.52311	4
−6	2	−1	21.7371	2.79862	3
7	2	1	8.22807	3.21579	13
−7	2	−1	5.21408	1.41204	8
−8	−2	−1	2.25965	1.56753	8
8	2	1	−5.2011	3.37241	13
−9	−2	−1	17.5984	2.33555	8
9	2	1	16.3965	4.32963	13
10	3	−1	16.8504	3.14915	13
−9	−3	1	47.8492	4.13618	8
9	3	−1	49.3180	6.82872	13
−8	−3	1	29.6409	3.90599	10
8	3	−1	32.6372	5.29871	13
−8	3	1	28.4847	3.50314	7
−7	−3	1	57.7140	5.26779	10
7	3	−1	59.6301	7.30744	13
−7	3	1	47.7088	4.69293	7

−6	−3	1	1.73882	2.28583	1
−6	3	1	0.67724	1.14476	3
−6	3	1	0.54367	0.79298	4
−6	3	1	1.49670	0.89903	4
−5	3	1	127.859	8.33585	4
−5	3	1	124.621	8.35847	4
−5	3	1	126.275	8.47792	3
−4	−3	1	736.201	46.9335	2
−4	−3	1	693.344	45.5397	4
−4	−3	1	700.605	46.6543	1
−4	−3	1	700.778	46.2477	5
−4	3	1	747.649	45.1291	4
−4	3	1	777.931	45.0615	4
−4	3	1	797.841	45.1453	3
−3	−3	1	153.317	9.88068	3
−3	−3	1	160.733	10.9912	2
−3	−3	1	151.676	11.1046	5
−3	−3	1	153.127	10.0159	4
−3	3	1	142.599	9.51841	3
−3	3	1	149.383	9.53724	4
−3	3	1	151.215	9.63944	4
−2	−3	1	3165.21	202.378	5
−2	−3	1	3186.36	201.983	2
−2	−3	1	3226.88	201.469	4
−2	3	1	3490.82	201.818	3
−2	3	1	3439.94	201.862	4
−2	3	1	3448.83	201.253	4
−1	−3	1	458.536	28.2493	5
−1	3	1	447.396	27.4893	4
−1	3	1	443.837	27.4146	4
−1	3	1	452.296	27.4266	3
1	3	−1	467.815	27.6974	4
−1	3	1	428.884	27.3820	3
0	3	−1	947.222	57.8723	4
0	3	1	967.380	58.3086	5
0	3	1	993.334	57.9851	4
0	3	−1	917.913	57.8712	5
0	3	−1	970.302	57.8685	4
0	3	−1	933.475	57.8692	4
−1	−3	−1	524.289	33.9809	2
−1	−3	−1	556.960	34.1072	5
−1	−3	−1	546.910	33.6169	4
1	3	1	563.528	34.1865	5
−2	−3	−1	64.9983	5.33999	5
−2	−3	−1	58.2650	5.12073	2
−2	−3	−1	55.0832	4.18291	4
−2	−3	−1	59.9761	4.20550	3
−2	−3	−1	54.0638	4.91679	5
−2	−3	−1	59.6028	4.40184	4
2	3	1	57.4531	5.19838	5

−3	−3	−1	444.388	28.4584	2
−3	−3	−1	435.211	27.4433	4
−3	−3	−1	437.742	28.1061	5
−3	−3	−1	427.737	27.2428	3
−3	3	−1	423.030	26.8043	3
−3	3	−1	444.285	26.7249	4
−3	3	−1	454.498	26.7074	4
−3	3	−1	436.011	26.7965	3
−4	−3	−1	0.17173	0.93742	5
−4	−3	−1	0.01189	1.92798	1
−4	−3	−1	0.04931	1.25047	4
−4	−3	−1	0.52149	2.36536	2
−4	−3	−1	−0.1965	0.90047	5
−5	−3	−1	3.51121	2.64687	2
−5	−3	−1	5.08062	1.96606	4
−5	−3	−1	4.32997	2.34767	1
−6	3	−1	113.413	7.93096	3
−6	3	−1	110.488	7.16037	9
−7	−3	−1	88.3091	7.13783	10
−7	3	−1	89.4430	6.15059	8
7	3	1	90.0851	8.81292	13
−8	−3	−1	9.76414	1.96967	8
8	3	1	5.51492	3.79517	13
−9	−3	−1	1.19898	1.17942	8
9	3	1	3.90652	3.22474	13
10	4	−1	26.2222	4.48489	13
−9	−4	1	59.4592	4.52842	8
−9	4	1	41.5639	4.94260	7
−8	−4	1	46.3956	4.74097	10
−8	4	1	30.8686	3.91769	7
8	4	−1	44.0847	6.53931	13
−7	−4	1	63.8140	5.72813	10
−7	4	1	46.5386	4.64962	7
7	4	−1	49.8791	6.67124	13
−6	−4	1	32.3452	5.12085	1
−6	4	1	29.4646	3.08749	3
−5	−4	1	18.0417	4.27104	2
−5	−4	1	15.6887	2.62181	5
−5	−4	1	19.3576	3.99937	1
−5	4	1	23.8889	2.26555	3
−5	4	1	20.4271	1.79739	4
−5	4	1	22.0968	1.87875	4
−4	−4	1	24.0105	3.84729	2
−4	−4	1	22.3080	4.45297	1
−4	−4	1	23.2633	3.97418	5
−4	4	1	18.4013	1.78653	4
−4	4	1	16.9364	1.81812	4
−4	4	1	16.1157	1.79278	3
−3	−4	1	626.893	39.6995	2
−3	−4	1	696.270	40.3394	5

−3	−4	1	652.966	38.9943	4
−3	4	1	586.091	38.1776	4
−3	4	1	619.913	38.2601	3
−3	4	1	586.187	38.2488	4
−2	−4	1	106.006	7.64375	2
−2	−4	1	103.334	6.94743	4
−2	−4	1	109.743	8.11119	5
−2	4	1	101.451	6.44056	3
−2	4	1	97.8339	6.42529	4
−2	4	1	98.4034	6.54165	4
1	−4	−1	3515.66	226.748	2
−1	−4	1	3768.54	228.737	5
−1	4	1	3576.23	226.856	4
−1	4	1	3597.99	226.290	3
−1	4	1	3773.73	226.845	4
−1	4	1	3945.18	226.968	3
1	4	−1	3990.20	227.114	4
0	4	−1	427.112	25.9135	3
0	4	1	402.133	25.9834	3
0	4	−1	427.407	25.9375	4
0	4	−1	451.896	25.9452	4
0	4	1	410.598	26.0337	4
−1	−4	−1	591.287	37.4848	2
−1	−4	−1	598.163	37.6246	5
1	4	1	619.685	37.6828	5
−2	−4	−1	164.382	10.9318	3
−2	−4	−1	172.616	11.8257	2
−2	−4	−1	171.464	11.1543	4
−2	−4	−1	170.671	11.9784	5
−3	−4	−1	583.728	37.0835	4
−3	−4	−1	602.945	38.0639	2
−3	−4	−1	631.221	38.0616	5
−3	4	−1	565.566	36.3053	3
−3	4	−1	601.543	36.3033	3
−4	−4	−1	20.3039	3.19698	5
−4	−4	−1	19.3488	4.11665	1
−4	−4	−1	18.6335	3.92278	2
−5	−4	−1	150.808	12.8394	2
−5	−4	−1	150.839	11.8763	1
−5	4	−1	153.970	10.0252	3
−6	−4	−1	11.5454	3.10112	1
−6	4	−1	8.20663	2.03324	7
−6	4	−1	6.75686	1.51015	3
−6	4	−1	7.87262	1.04343	9
−6	4	−1	8.83728	0.92142	6
−7	−4	−1	24.6534	3.81499	10
−7	4	−1	36.7815	2.55689	9
−7	4	−1	37.9865	2.92351	8
7	4	1	23.8231	4.61559	13
−8	−4	−1	73.9253	6.08195	8

8	4	1	67.1754	8.26313	13
−8	4	−1	89.3197	5.32073	9
−9	4	−1	3.62332	0.56819	6
−9	4	−1	4.28685	0.80352	9
−9	4	−1	4.78426	0.90956	9
9	4	1	−0.8911	2.96129	13
10	5	−1	2.17350	2.53045	13
−9	−5	1	32.5726	3.48845	8
−9	5	1	31.7882	2.43333	9
−9	5	1	31.1609	3.41219	7
9	5	−1	28.0641	4.95098	13
−8	−5	1	5.67916	2.21115	10
8	5	−1	3.09035	3.62521	13
−8	5	1	2.47268	1.78921	7
−7	−5	1	163.646	11.8926	10
−7	−5	1	157.448	12.8642	1
−7	5	1	178.369	10.5553	9
−7	5	1	173.622	11.3671	7
7	5	−1	154.710	13.1527	13
−6	−5	1	78.3467	8.03842	1
−6	5	1	72.1214	5.43027	3
−6	5	1	73.9172	4.69227	6
−6	5	1	69.9597	5.82931	7
−5	−5	1	21.3121	3.67885	5
−5	−5	1	22.9913	4.54353	2
−5	−5	1	21.4442	4.38827	1
−4	5	1	46.1887	3.24959	4
−4	5	1	49.2003	3.31659	4
−4	5	1	52.9862	3.58756	3
−3	−5	1	1321.81	77.4670	5
−3	−5	1	1267.17	76.7059	2
−3	5	1	1206.61	75.1574	3
−3	5	1	1187.52	75.0431	4
−3	5	1	1214.55	75.1143	4
−2	−5	1	202.963	15.8311	5
−2	5	1	232.459	14.3368	3
−2	5	1	236.825	14.3814	3
−2	5	1	257.719	14.4321	4
1	−5	−1	802.348	49.4765	2
−1	−5	1	738.028	49.6846	5
−1	5	1	857.005	48.7222	3
−1	5	1	831.843	48.7313	4
−1	5	1	810.995	48.7912	4
−1	5	1	826.277	48.7703	3
1	5	−1	776.171	49.4641	5
1	5	−1	769.532	48.8998	4
0	−5	−1	49.7438	4.58399	2
0	5	1	54.2121	3.76376	3
0	5	1	56.1900	3.93760	4
0	5	1	57.8696	3.86080	4

−1	−5	−1	193.449	12.9473	5
−1	−5	−1	206.056	12.8879	2
−1	5	−1	178.800	11.6112	3
−1	5	−1	183.025	11.5961	4
−1	5	−1	190.829	11.5947	4
−1	5	−1	185.151	11.6141	3
1	5	1	205.335	12.2413	4
−2	−5	−1	13.1553	2.86673	5
−2	−5	−1	13.1451	2.52273	2
−2	−5	−1	14.1820	2.01407	4
−3	−5	−1	23.6204	4.03421	2
−3	−5	−1	24.3141	3.79220	1
−3	−5	−1	24.5485	3.72905	5
−3	5	−1	18.4725	1.45115	3
−3	5	−1	17.5881	1.43085	3
−4	−5	−1	243.780	16.9367	5
−4	−5	−1	235.513	17.1352	1
−4	−5	−1	248.211	17.4844	2
−5	−5	−1	840.477	54.0705	1
−5	−5	−1	868.451	54.7870	2
−5	5	−1	834.184	51.8593	3
−6	−5	−1	54.2442	6.33892	1
−6	−5	−1	53.3909	7.58710	2
−6	5	−1	52.9456	3.61418	9
−6	5	−1	46.9568	4.42335	7
−6	5	−1	55.0238	3.47102	6
−6	5	−1	56.3734	4.69794	3
−7	−5	−1	10.0335	2.37308	8
−7	−5	−1	9.82210	2.48907	10
−7	5	−1	7.23787	0.83098	6
−7	5	−1	5.58174	1.92700	7
−7	5	−1	6.04637	1.05107	9
7	5	1	8.59285	3.72612	13
−8	−5	−1	14.1541	2.43258	8
−8	−5	−1	15.3479	2.81418	10
8	5	1	7.46867	4.96548	13
−8	5	−1	15.7892	1.74478	9
−8	5	−1	18.6162	1.37207	6
−9	−5	−1	8.21381	1.68925	8
−9	5	−1	9.39893	0.87710	6
9	5	1	2.44828	3.28235	13
−9	5	−1	10.2713	1.23244	9
10	6	−1	7.00338	2.63445	13
−9	−6	1	1.26101	1.59928	8
−9	6	1	0.87295	0.66895	9
−9	6	1	2.23598	0.50664	6
9	6	−1	5.55515	3.65272	13
−9	6	1	1.60022	1.53444	7
−8	6	1	19.6422	1.62486	6
−8	6	1	17.5872	1.71190	9

−8	6	1	19.5373	3.22508	7
−7	−6	1	63.6984	6.98989	1
−7	−6	1	64.1825	5.74929	10
7	6	−1	64.8200	8.07371	13
−7	6	1	53.5849	3.64168	6
−7	6	1	50.0739	3.85263	9
−7	6	1	45.9894	4.77205	7
−6	6	1	18.7440	1.49047	6
−6	6	1	17.1080	2.66750	7
−6	6	1	19.1020	2.23163	3
−6	6	1	17.7543	1.56127	9
−5	−6	1	43.7518	5.93287	2
−5	−6	1	39.1078	5.26236	5
−5	−6	1	40.3406	5.82077	1
−5	6	1	45.7770	3.43193	3
−4	−6	1	45.1005	6.13231	5
−4	−6	1	41.9232	5.58138	1
−4	−6	1	43.1582	5.58586	2
−3	−6	1	3.64702	2.27422	2
−3	−6	1	5.56775	2.59334	5
−3	6	1	7.37151	1.03556	3
−3	6	1	7.73928	0.72732	4
−3	6	1	6.94243	0.78778	4
−2	−6	1	1196.49	67.5762	5
−2	−6	1	1125.30	66.7318	2
−2	6	1	1066.46	65.5929	3
−2	6	1	1040.37	65.4700	4
−2	6	1	1013.66	65.5187	3
−2	6	1	1043.76	65.5309	4
−1	−6	1	27.5857	3.72571	5
−1	−6	1	27.6677	3.46103	2
1	−6	−1	39.2876	3.81295	2
1	6	−1	29.9623	3.55666	5
−1	6	1	42.4032	2.77499	3
−1	6	1	38.1546	2.72023	4
−1	6	1	40.1424	2.86649	4
−1	6	1	41.6585	2.65592	3
0	−6	−1	458.561	29.9305	2
0	6	1	473.929	29.2956	4
0	6	−1	477.292	29.1562	3
0	6	1	467.629	29.3261	4
0	6	−1	486.936	29.1669	4
0	6	−1	511.896	29.1834	4
0	6	1	455.034	29.2391	3
1	−6	1	229.706	16.0147	2
−1	−6	−1	253.583	16.6308	2
−1	−6	−1	256.726	16.7515	5
−1	6	−1	243.244	15.2774	4
1	6	1	274.329	15.8963	4
−1	6	−1	248.639	15.2698	4

−2	−6	−1	978.901	60.9412	5
−2	−6	−1	969.130	60.5095	2
−3	−6	−1	28.0879	4.02672	2
−3	−6	−1	28.3195	4.53565	5
−3	−6	−1	29.2963	4.81723	1
−3	6	−1	24.6114	1.73892	3
−4	−6	−1	144.463	11.2684	1
−4	−6	−1	140.490	11.0656	2
−4	−6	−1	139.774	10.9597	5
−4	6	−1	117.007	8.39339	3
−5	−6	−1	8.86397	3.48307	1
−5	−6	−1	9.93054	3.56232	2
−5	−6	−1	7.35703	1.87429	5
−6	−6	−1	14.1608	4.57360	2
−6	−6	−1	18.7280	4.29179	1
−6	−6	−1	15.5840	3.07356	10
−6	6	−1	14.9808	1.49245	9
−6	6	−1	16.6397	2.63297	7
−6	6	−1	16.5806	1.33989	6
−7	−6	−1	22.7882	3.23640	8
−7	−6	−1	23.0936	3.91361	10
−7	6	−1	18.1990	1.86219	9
−7	6	−1	18.1366	3.10276	7
−7	6	−1	21.3820	1.58823	6
7	6	1	24.4006	4.60001	13
−8	−6	−1	12.9297	2.30511	8
−8	−6	−1	10.8017	2.40621	10
8	6	1	10.9409	3.98070	13
−8	6	−1	8.96256	0.95746	6
−8	6	−1	9.73848	1.20642	9
−9	−6	−1	8.17978	1.73445	8
9	6	1	6.72035	3.48032	13
−9	6	−1	12.7779	0.96303	6
−9	6	−1	12.0288	1.40145	9
−9	−7	1	20.9033	2.97284	8
−9	−7	1	21.6040	2.76412	10
−9	7	1	18.9187	2.83293	7
−9	7	1	20.9087	1.81417	9
9	7	−1	20.7224	4.25662	13
−9	7	1	20.8770	1.54409	6
−8	−7	1	5.48941	2.56130	10
8	7	−1	−1.6352	3.92641	13
−8	7	1	2.29879	0.68254	6
−8	7	1	3.99304	1.99889	7
−8	7	1	3.46289	0.75728	9
−7	−7	1	271.410	18.1785	10
−7	−7	1	275.883	19.8328	1
−7	7	1	262.790	16.5620	9
−7	7	1	255.008	17.4338	7
7	7	−1	270.880	18.8098	13

−7	7	1	272.327	16.4035	6
−6	−7	1	49.4097	7.57926	2
−6	−7	1	44.6719	6.92310	1
−6	7	1	33.4096	3.52284	7
−6	7	1	31.9635	2.54114	9
−6	7	1	32.8454	3.03346	3
−6	7	1	35.2192	2.37430	6
−5	−7	1	212.826	16.1790	5
−5	−7	1	232.001	16.6259	1
−5	−7	1	227.325	16.4790	2
−5	7	1	185.861	13.3998	3
−4	−7	1	440.087	31.7167	5
−4	−7	1	431.036	30.9749	2
−4	−7	1	425.710	31.2688	1
−4	7	1	538.362	28.9860	3
−4	7	1	509.577	29.0602	3
−3	−7	1	79.7932	6.76947	2
−3	−7	1	76.4387	7.37295	1
−3	−7	1	85.8019	7.76495	5
−3	7	1	69.7938	4.88086	3
−3	7	1	68.8021	4.96508	3
−2	−7	1	520.935	34.7198	2
2	−7	−1	531.409	34.8259	2
−2	7	1	540.708	33.4681	4
−2	7	1	566.564	33.5830	3
−2	7	1	556.556	33.5199	4
−2	7	1	582.284	33.6615	3
−1	−7	1	45.5921	4.42591	2
1	−7	−1	51.7043	4.67727	2
−1	7	1	50.1864	3.57003	4
−1	7	1	55.1110	3.53110	4
1	7	−1	42.4624	4.26250	5
1	7	−1	46.2721	3.70840	4
−1	7	1	52.3421	3.57104	3
−1	7	1	53.1236	3.46220	3
1	7	−1	44.6177	3.79316	4
0	−7	−1	1408.05	86.1370	2
0	−7	1	1307.55	85.7262	2
0	7	1	1382.84	85.3373	3
0	7	1	1473.26	85.4022	4
0	7	−1	1417.76	85.2545	3
0	7	−1	1396.54	85.2382	4
0	7	−1	1404.11	85.2432	4
0	7	1	1443.18	85.4268	4
−1	−7	−1	56.8356	5.50272	2
1	−7	1	64.1177	5.06735	2
−1	−7	−1	53.5374	5.78565	5
−1	7	−1	68.7549	4.18424	3
−1	7	−1	68.1511	4.07738	4
1	7	1	56.2417	4.65206	4

−1	7	−1	71.9991	4.12296	4
−2	−7	−1	308.248	20.7373	2
−2	−7	−1	317.684	21.3414	5
−3	−7	−1	144.047	11.5998	5
−3	−7	−1	141.167	11.3805	1
−3	−7	−1	145.652	11.1888	2
−4	−7	−1	250.014	18.2544	1
−4	−7	−1	251.866	18.2598	2
−4	−7	−1	246.263	18.1956	5
−4	7	−1	257.215	15.5083	3
−5	−7	−1	25.0478	5.38731	2
−5	−7	−1	27.6132	5.70169	1
−5	7	−1	35.3239	2.17630	6
−5	7	−1	33.0833	2.17352	6
−5	7	−1	34.8061	2.67401	3
−6	−7	−1	13.7026	4.41834	2
−6	−7	−1	12.2985	4.11237	1
−6	7	−1	14.0900	1.32711	9
−6	7	−1	12.5959	1.15162	6
−7	−7	−1	302.092	19.5399	8
−7	−7	−1	315.618	20.2016	10
7	7	1	303.006	20.9483	13
−7	7	−1	307.107	18.4413	6
−7	7	−1	297.656	18.5649	9
−7	7	−1	287.042	19.4338	7
−8	7	−1	10.7142	0.94010	6
−8	7	−1	9.29226	2.50123	7
−8	7	−1	9.83734	1.29450	9
−9	−7	−1	0.10329	0.91796	8
−9	7	−1	0.26941	0.67983	9
9	7	1	1.14820	2.91405	13
−9	7	−1	0.97823	0.33213	6
−9	7	−1	0.78141	0.38529	6
−9	−8	1	4.69162	2.19956	10
−9	−8	1	5.15007	1.85249	8
−9	8	1	7.88503	1.50681	7
−9	8	1	9.27086	0.78324	6
−9	8	1	8.36539	1.08457	9
9	8	−1	4.32163	3.32269	13
−8	−8	1	4.02986	2.64774	10
−8	−8	1	3.87796	1.99460	8
−8	8	1	5.25930	0.65091	6
8	8	−1	14.8810	4.40523	13
−8	8	1	4.99503	0.81253	9
−8	8	1	5.40045	2.10481	7
−7	−8	1	87.6333	9.47781	1
−7	−8	1	84.0213	7.64219	10
−7	8	1	89.5195	6.84688	7
7	8	−1	91.1451	8.92284	13
−7	8	1	83.7696	5.67937	9

−7	8	1	88.4097	5.50124	6
−6	−8	1	8.56061	2.75339	10
−6	−8	1	10.5472	4.51781	1
−6	−8	1	10.0788	4.00360	2
−6	8	1	6.89537	1.83335	7
−6	8	1	4.95156	0.69154	6
−6	8	1	7.35925	0.93754	9
−5	−8	1	32.0483	5.96837	2
−5	−8	1	30.8568	5.32407	5
−5	−8	1	32.4372	6.37452	1
−4	−8	1	61.9360	7.13434	1
−4	−8	1	62.8097	7.38397	5
−4	−8	1	64.3621	6.98398	2
−4	8	1	62.2132	4.45545	3
−4	8	1	64.8721	4.39003	3
−3	−8	1	131.710	10.7645	2
−3	−8	1	131.152	11.4356	1
−3	8	1	150.133	9.18067	3
−3	8	1	153.845	9.09018	3
−2	−8	1	253.708	18.6289	2
2	−8	−1	291.795	19.0088	2
−2	8	1	279.553	17.3549	4
−2	8	1	296.974	17.4891	3
−2	8	1	278.008	17.5339	3
−2	8	1	291.959	17.3279	4
−1	−8	1	175.746	11.8935	2
1	−8	−1	169.778	12.1945	2
−1	8	1	173.155	10.9322	4
−1	8	1	173.794	10.9218	3
1	8	−1	178.992	11.2044	4
1	8	−1	163.957	11.6295	5
−1	8	1	174.932	11.0172	3
−1	8	1	170.578	10.9591	4
0	−8	1	660.977	42.2505	2
0	−8	−1	663.123	42.6124	2
0	8	1	687.830	41.7877	4
0	8	1	664.338	41.7178	3
0	8	−1	705.959	41.6239	4
0	8	−1	680.087	41.6223	4
0	8	1	691.785	41.7704	4
0	8	−1	689.397	41.6339	3
1	−8	1	1432.00	90.0175	2
−1	−8	−1	1435.66	90.4567	2
−1	8	−1	1547.45	89.0768	4
1	8	1	1406.35	89.6019	4
−1	8	−1	1492.26	89.1536	3
−1	8	−1	1504.31	89.0409	4
2	−8	1	35.0655	3.93603	2
−2	8	−1	38.1428	2.47028	3
−3	−8	−1	136.029	11.0745	5

−3	−8	−1	126.910	10.8565	1
−3	−8	−1	131.656	10.6139	2
−4	−8	−1	265.253	18.9930	2
−4	−8	−1	250.862	19.0212	5
−4	−8	−1	264.051	19.0844	1
−4	8	−1	260.041	15.9343	3
−5	−8	−1	8.67247	3.58252	2
−5	−8	−1	7.98204	3.43921	1
−5	8	−1	10.9105	0.83824	6
−5	8	−1	9.97174	1.23271	3
−5	8	−1	13.4904	2.06245	7
−5	8	−1	9.74378	0.79467	6
−6	−8	−1	32.8622	6.48777	1
−6	−8	−1	34.6671	4.60386	10
−6	−8	−1	32.3652	6.41898	2
6	8	1	40.2470	6.38635	13
−6	8	−1	29.4036	3.58241	7
−6	8	−1	33.0889	2.14908	6
−6	8	−1	30.2341	2.10783	6
−6	8	−1	30.1885	2.28814	9
−7	−8	−1	44.5122	4.70434	8
−7	−8	−1	49.4257	5.46524	10
−7	−8	−1	44.2370	6.19066	1
−7	8	−1	49.8292	3.32597	9
−7	8	−1	46.4282	4.58067	7
7	8	1	44.5112	7.22934	13
−7	8	−1	54.4182	3.17338	6
−8	−8	−1	166.812	10.8077	8
−8	−8	−1	155.189	11.0403	10
−8	8	−1	150.659	10.6051	7
−8	8	−1	147.481	9.58678	6
8	8	1	183.310	12.7898	13
−8	8	−1	145.955	9.58079	6
−8	8	−1	157.004	9.73598	9
−9	−8	−1	1.51177	1.41959	8
9	8	1	2.76912	2.88015	13
−9	8	−1	2.14519	0.52502	9
−9	−9	1	−0.2618	1.50018	8
−9	−9	1	1.69389	1.73574	10
−9	9	1	1.06457	0.32121	6
9	9	−1	1.80574	3.13434	13
−9	9	1	0.67852	1.38299	7
−9	9	1	0.17815	0.55317	9
−9	9	1	0.96699	0.32447	6
−8	−9	1	29.2788	3.93241	8
−8	−9	1	29.9923	4.59026	10
−8	9	1	41.8517	3.66909	7
−8	9	1	41.5440	2.58662	9
8	9	−1	25.9667	5.07710	13
−8	9	1	37.4334	2.40107	6

−7	−9	1	55.1623	5.78580	10
−7	−9	1	53.9202	7.46320	1
7	9	−1	57.6730	8.08800	13
−7	9	1	52.7070	4.87462	7
−7	9	1	49.6636	3.35734	6
−7	9	1	49.1866	3.54720	9
−6	−9	1	54.0144	7.36747	2
−6	−9	1	51.7190	5.38415	10
−6	9	1	39.6333	4.06825	7
−6	9	1	39.1142	2.68674	6
−6	9	1	36.6427	2.84596	9
−5	−9	1	491.581	34.8077	2
−5	−9	1	515.902	35.4766	1
−5	9	1	506.086	32.5104	3
−5	9	1	513.438	31.3302	6
−5	9	1	590.454	32.8121	7
−5	9	1	483.962	31.7492	3
−4	−9	1	194.442	14.3447	1
−4	−9	1	198.313	13.7650	2
−4	9	1	161.745	10.9177	3
−4	9	1	151.890	10.9994	3
−3	−9	1	9.78965	2.96285	2
−3	−9	1	6.88465	3.60893	1
3	−9	−1	11.1165	3.21835	2
−3	9	1	10.6180	1.24343	3
−3	9	1	11.3063	1.41322	3
2	−9	−1	333.488	22.2052	2
−2	9	1	346.661	20.6683	3
−2	9	1	343.121	20.4618	4
−2	9	1	318.789	20.6826	3
−2	9	1	325.434	20.4738	4
1	−9	−1	2.92544	2.25954	2
−1	9	1	2.56682	1.05746	3
−1	9	1	2.96999	0.99048	3
0	−9	−1	765.361	47.1744	2
0	−9	1	724.363	46.7219	2
0	9	−1	722.058	45.9542	4
0	9	−1	741.247	45.9857	4
0	9	−1	731.187	46.0997	3
0	9	1	818.304	46.2054	4
0	9	1	760.499	46.1304	3
1	−9	1	47.0850	4.70721	2
−1	−9	−1	56.0758	5.38109	2
−1	9	−1	52.1451	3.49539	4
−1	9	−1	48.9406	3.61561	3
−1	9	−1	55.8007	3.56372	4
−2	−9	−1	431.629	26.7368	2
2	−9	1	376.917	25.9112	2
−3	−9	−1	378.901	25.6930	1
−3	−9	−1	384.386	25.3437	2

3	−9	1	358.256	24.5507	2
−3	9	−1	367.766	22.6137	3
−3	9	−1	372.951	22.6343	3
−4	−9	−1	148.484	13.2018	1
−4	−9	−1	153.695	13.3653	2
−4	−9	−1	143.932	13.2308	5
−4	9	−1	180.471	10.0970	3
−4	9	−1	174.854	10.0474	3
−5	−9	−1	51.5860	7.30607	2
−5	−9	−1	51.0347	7.73927	1
−5	9	−1	54.3337	3.44914	6
−5	9	−1	54.2422	3.53685	9
−5	9	−1	54.0257	4.68161	7
−5	9	−1	58.0293	3.78024	3
−5	9	−1	58.7039	3.46910	6
−5	9	−1	56.4209	3.72269	3
−6	−9	−1	4.88489	2.02969	8
−6	−9	−1	4.62895	3.49175	1
−6	−9	−1	2.23273	2.70353	10
−6	9	−1	8.94783	0.78430	6
−6	9	−1	6.35707	0.76383	9
−6	9	−1	9.10507	2.16839	7
−6	9	−1	3.64133	2.62930	12
−6	9	−1	6.77645	0.70265	6
6	9	1	8.85381	4.25103	13
−7	−9	−1	70.1954	8.07449	1
−7	−9	−1	66.2805	6.70878	10
−7	−9	−1	65.7929	5.98863	8
−7	9	−1	71.9934	4.42904	6
−7	9	−1	71.0987	4.43641	6
7	9	1	67.5879	8.30742	13
−7	9	−1	73.5275	4.59089	9
−7	9	−1	74.5139	5.87295	7
−8	−9	−1	34.8338	3.78094	8
−8	−9	−1	37.4600	4.10768	10
8	9	1	36.8706	6.47321	13
−8	9	−1	24.6002	3.28918	7
−8	9	−1	28.3355	2.09411	9
−8	9	−1	27.8062	1.91387	6
−8	9	−1	27.9246	1.90951	6
−9	−9	−1	1.71892	1.69606	8
9	9	1	0.03257	2.69407	13
−9	−10	1	0.66938	2.09817	10
−9	−10	1	2.69648	1.59942	8
9	10	−1	−0.4600	2.81193	13
−9	10	1	2.09104	0.43766	9
−9	10	1	1.99720	0.53334	9
−9	10	1	2.39089	1.43726	7
−8	10	1	15.1309	1.15691	6
−8	10	1	13.3722	1.29398	9

−8	10	1	12.8853	1.13193	6
−7	−10	1	72.0333	9.05491	1
−7	10	1	48.5248	4.90689	7
7	10	−1	65.0119	8.48664	13
−7	10	1	49.0712	3.52057	9
−7	10	1	49.3859	3.34046	6
−6	−10	1	−1.2148	2.90792	2
−6	−10	1	2.33774	2.59549	10
−6	−10	1	5.11384	3.70706	1
6	−10	−1	9.66907	4.24616	2
−6	10	1	9.03110	0.88047	6
6	10	−1	0.63203	4.21400	13
−6	10	1	8.42122	1.03028	9
−6	10	1	9.70757	2.10663	7
−5	−10	1	86.5722	9.27364	2
−5	−10	1	80.5659	9.40628	1
5	−10	−1	87.0098	8.80048	2
−5	10	1	87.0082	5.23891	6
−5	10	1	85.5570	5.29261	6
−5	10	1	82.2827	6.33259	7
−5	10	1	82.6866	5.39578	9
−5	10	1	82.4474	5.67577	3
−5	10	1	84.4170	5.85424	3
−4	−10	1	34.7036	5.05837	2
4	−10	−1	34.2552	5.12105	2
−4	−10	1	36.1620	5.86176	1
−4	10	1	32.4780	2.59386	3
−4	10	1	32.1003	2.69659	3
3	−10	−1	16.4455	3.71943	2
−3	−10	1	11.2271	3.29584	2
−3	−10	1	12.6497	4.17819	1
−3	10	1	17.8405	1.71142	3
−3	10	1	17.7110	1.72270	3
−2	−10	1	386.785	22.9070	2
2	−10	−1	337.172	22.9470	2
−2	10	1	332.310	20.9488	4
−2	10	1	323.635	21.2370	3
−2	10	1	342.512	21.1985	3
−2	10	1	331.639	20.9657	4
1	−10	−1	95.9405	8.11516	2
−1	−10	1	90.9815	7.64581	2
1	10	−1	86.5265	6.72126	4
−1	10	1	97.6536	6.49648	4
−1	10	1	94.6665	6.40567	3
−1	10	1	101.448	6.56249	4
0	−10	1	79.2020	6.62813	2
0	10	−1	77.0959	5.38206	4
0	10	−1	75.8745	5.34917	4
−1	−10	−1	132.191	11.7350	2
1	−10	1	160.294	11.3989	2

−1	10	−1	171.463	10.1037	4
−1	10	−1	163.444	10.0446	4
−1	10	−1	168.447	10.2527	3
−2	−10	−1	570.715	41.7799	1
−2	−10	−1	637.048	39.7603	2
−2	10	−1	635.891	37.5823	3
−3	−10	−1	227.916	17.0479	2
−3	−10	−1	232.539	17.6160	1
−3	10	−1	228.885	14.2386	3
−3	10	−1	237.323	14.2261	3
−4	−10	−1	30.0559	5.57189	2
−4	−10	−1	38.0324	6.45708	5
−4	−10	−1	27.3691	5.48306	1
−4	10	−1	33.5136	2.04260	6
−5	−10	−1	5.13291	3.39685	1
−5	−10	−1	7.19155	3.55625	2
−5	10	−1	4.24398	0.49979	9
−5	10	−1	3.66095	0.39598	6
−5	10	−1	5.69783	1.98799	7
−5	10	−1	3.56087	0.56518	9
−5	10	−1	4.32554	0.43758	6
−6	−10	−1	14.8513	4.74246	1
−6	−10	−1	15.1939	3.71681	10
−6	10	−1	24.0622	1.56958	6
−6	10	−1	33.4601	4.28878	12
−6	10	−1	23.2443	1.80328	9
−6	10	−1	22.2446	3.31600	7
−6	10	−1	24.4485	1.72372	9
−6	10	−1	26.1784	1.59352	6
6	10	1	17.0703	5.33304	13
−7	−10	−1	18.9954	3.07434	8
−7	−10	−1	20.2644	3.84142	10
−7	−10	−1	22.4560	4.97145	1
7	10	1	21.0010	4.56556	13
−7	10	−1	23.7686	3.36755	7
−7	10	−1	23.2990	1.49965	6
−7	10	−1	22.3351	1.49257	6
−7	10	−1	20.4220	1.64827	9
−8	−10	−1	0.57238	1.28462	8
−8	−10	−1	0.49275	1.70142	10
−8	10	−1	1.91686	0.52893	9
8	10	1	−2.3428	3.36336	13
−8	10	−1	0.45053	1.55990	7
−8	10	−1	1.00788	0.54166	9
9	10	1	7.02178	2.70379	13
−9	−11	1	6.50845	2.03374	10
9	11	−1	3.80213	2.65042	13
−9	11	1	4.02412	1.19540	7
−8	−11	1	7.79405	2.85998	10
−8	−11	1	5.81597	2.15322	8

-8	11	1	5.83818	2.01083	7
-8	11	1	7.73904	0.71925	6
-8	11	1	7.63353	0.74747	6
8	11	-1	6.66555	3.69292	13
-8	11	1	9.28066	0.96844	9
-7	-11	1	152.691	11.4532	10
-7	-11	1	155.569	14.2623	1
7	11	-1	153.581	12.0160	13
-7	11	1	148.047	9.05899	6
-7	11	1	144.980	10.3781	7
-7	11	1	139.514	9.04341	6
-7	11	1	144.817	9.19915	9
-6	-11	1	34.3570	5.34324	10
-6	-11	1	38.0118	8.10740	1
6	11	-1	41.6038	6.22142	13
-6	11	1	49.1693	2.92928	6
-6	11	1	43.2852	4.49508	7
-6	11	1	48.5992	2.93631	6
-6	11	1	47.3136	3.11307	9
-6	11	1	42.3653	5.84886	12
5	-11	-1	11.5231	4.22657	2
-5	-11	1	15.3467	4.02946	2
-5	-11	1	13.3939	5.07790	1
-5	11	1	9.14371	0.82588	6
-5	11	1	9.04485	1.93957	7
-5	11	1	7.85538	1.19616	3
-5	11	1	9.21155	1.14878	9
-5	11	1	7.33348	1.29595	3
-5	11	1	8.65052	0.79130	6
4	-11	-1	98.4608	9.08682	2
-4	-11	1	88.9218	10.1152	1
-4	-11	1	87.2741	8.86643	2
-4	11	1	98.0399	6.46963	3
-4	11	1	99.6937	6.36126	3
3	-11	-1	2.34900	2.31713	2
-3	-11	1	5.22001	2.42619	2
-3	11	1	2.20496	0.87516	3
-3	11	1	2.33215	0.83505	3
-2	-11	1	101.896	8.70988	2
2	-11	-1	106.914	8.95438	2
-2	11	1	101.114	6.72143	4
-2	11	1	105.490	6.71486	4
-2	11	1	108.760	7.04913	3
-1	-11	1	109.083	9.10268	2
1	-11	-1	120.611	9.47186	2
1	11	-1	104.198	8.06076	4
-1	11	1	120.953	7.86737	4
-1	11	1	122.498	7.89607	3
-1	11	1	125.336	7.85562	4
0	-11	1	1214.36	76.0049	2

0	−11	−1	1240.07	76.4504	2
0	11	−1	1208.19	74.9312	4
0	11	1	1221.03	75.1074	3
0	11	1	1260.30	75.1488	4
−1	−11	−1	87.4015	8.04520	2
−1	11	−1	85.0262	5.74832	4
−1	11	−1	93.5785	5.81079	4
−1	11	−1	86.0055	5.99654	3
−2	−11	−1	286.359	19.5670	2
−2	11	−1	265.338	17.1672	3
−2	11	−1	280.753	17.1830	3
−3	−11	−1	116.394	10.4494	1
−3	−11	−1	123.084	10.0422	2
−3	11	−1	103.284	6.92121	3
−3	11	−1	106.309	6.93046	3
−4	−11	−1	63.4191	7.18253	2
−4	11	−1	46.4342	2.99754	6
−4	11	−1	48.6376	3.01187	6
−5	−11	−1	23.0679	5.19238	2
−5	−11	−1	27.4158	6.11182	1
−5	−11	−1	20.3460	3.77756	10
−5	11	−1	17.0623	2.64020	7
−5	11	−1	13.7338	0.92955	6
−5	11	−1	12.0149	1.06179	9
−5	11	−1	12.9385	1.04499	9
−5	11	−1	12.7835	0.92091	6
−6	−11	−1	22.2507	6.14378	1
−6	−11	−1	18.5378	3.58290	10
−6	11	−1	11.2019	1.01154	9
−6	11	−1	10.6646	0.84728	6
6	11	1	18.9678	4.73398	13
−6	11	−1	10.5733	1.10799	9
−6	11	−1	10.1015	2.30675	7
−6	11	−1	10.8664	2.86573	12
−6	11	−1	11.3962	0.85486	6
−7	−11	−1	193.321	13.8769	8
−7	−11	−1	199.124	14.6491	10
−7	−11	−1	174.866	15.8150	1
−7	11	−1	202.359	12.5810	9
−7	11	−1	211.529	12.5692	9
−7	11	−1	216.061	12.4544	6
−7	11	−1	206.755	13.7833	7
−7	11	−1	211.582	12.4579	6
7	11	1	228.456	16.0299	13
−8	−11	−1	16.5583	3.02583	10
−8	−11	−1	13.1544	2.44205	8
8	11	1	12.8660	3.71161	13
−8	11	−1	7.91284	1.68384	7
9	11	1	3.70373	2.30328	13
−9	−12	1	10.9343	2.16848	10

−8	12	1	21.8420	3.25864	7
8	12	−1	26.7727	4.91165	13
−8	12	1	20.0723	1.77825	9
−8	12	1	25.2861	1.66402	9
−7	−12	1	28.6384	4.99782	10
−7	−12	1	26.0552	5.90948	1
7	12	−1	25.0845	4.60315	13
−7	12	1	32.2677	2.27057	9
−7	12	1	36.5362	4.03656	7
−7	12	1	31.8848	2.07937	6
−7	12	1	32.4430	2.06539	6
−6	−12	1	89.8122	8.39382	10
−6	−12	1	108.368	11.4163	1
−6	12	1	95.4493	7.34919	7
6	12	−1	91.8487	9.46601	13
−6	12	1	98.1227	5.96788	6
−6	12	1	96.5890	6.11487	9
−6	12	1	96.3977	5.97316	6
5	−12	−1	12.1315	3.79613	2
−5	−12	1	15.0666	4.21774	2
−5	−12	1	16.7016	5.09275	1
−5	12	1	13.7350	1.06907	6
−5	12	1	15.3601	1.10578	6
−5	12	1	11.6559	1.77702	3
−5	12	1	14.3179	2.37938	7
−5	12	1	13.7844	1.39785	9
−4	−12	1	211.173	16.4335	2
−4	−12	1	202.182	17.2442	1
4	−12	−1	213.466	16.4864	2
−4	12	1	215.567	13.0517	6
−4	12	1	210.121	13.6188	3
−4	12	1	219.389	13.5289	3
−4	12	1	221.546	13.1245	6
−3	−12	1	593.363	36.4921	2
3	−12	−1	518.909	36.0589	2
−3	12	1	540.329	33.8739	3
−2	−12	1	91.4284	7.31987	2
2	−12	−1	72.6292	7.28921	2
−2	12	1	73.8241	4.96115	4
−2	12	1	70.4991	4.93639	4
−2	12	1	73.7717	5.34737	3
1	−12	−1	1087.45	68.1643	2
−1	−12	1	1129.80	67.9338	2
−1	12	1	1043.19	66.4037	4
−1	12	1	1033.65	67.0513	4
1	12	−1	1127.42	66.8427	4
−1	12	1	1104.62	66.5603	3
0	−12	−1	78.7322	7.54111	2
0	12	−1	83.7989	5.70440	4
0	12	1	73.3538	5.94042	4

0	12	−1	82.7811	5.87635	3
−1	−12	−1	511.267	35.3294	2
−1	12	−1	550.050	33.3714	4
−1	12	−1	571.429	33.6822	3
−2	−12	−1	279.088	20.2320	2
−2	12	−1	302.785	18.0590	3
−2	12	−1	284.114	18.0180	3
−3	−12	−1	1.42513	2.83368	1
−3	−12	−1	2.71638	2.50625	2
−3	12	−1	9.69860	1.06440	3
−4	−12	−1	35.8592	6.02974	2
−4	−12	−1	39.1786	7.51308	1
−4	12	−1	36.5452	2.35205	6
−4	12	−1	40.0294	2.47718	9
−4	12	−1	37.0851	2.36609	6
−4	12	−1	38.8190	3.70170	7
−5	−12	−1	5.02847	2.90194	10
−5	−12	−1	5.22558	3.75400	1
−5	−12	−1	7.99545	3.76483	2
−5	12	−1	7.44211	0.68849	9
−5	12	−1	6.02346	2.23395	7
−5	12	−1	6.17237	0.52587	6
−5	12	−1	7.53785	0.73507	9
−5	12	−1	6.25878	0.51195	6
−5	12	−1	1.73004	2.63540	12
−6	−12	−1	155.397	15.0393	1
−6	−12	−1	155.770	12.6259	10
−6	12	−1	174.237	10.3444	9
−6	12	−1	173.229	11.6708	7
6	12	1	149.443	13.2855	13
−6	12	−1	181.267	10.3637	9
−6	12	−1	183.124	12.1916	12
−7	−12	−1	14.2222	3.06387	10
7	12	1	11.9603	4.67798	13
−7	12	−1	13.8328	3.16076	12
−7	12	−1	17.8507	2.75341	7
−8	−12	−1	27.8008	3.95542	10
−8	−12	−1	30.4682	3.60821	8
8	12	1	23.2713	4.06466	13
−9	−13	1	18.2950	2.66683	10
−8	−13	1	12.7408	3.13238	10
−8	13	1	8.07265	2.11180	7
−7	−13	1	38.1572	5.45787	10
−7	−13	1	31.1057	8.35881	1
−7	13	1	34.8445	2.37953	6
−7	13	1	32.5591	2.35118	6
−7	13	1	36.1242	4.23921	7
7	13	−1	30.8643	5.90546	13
−7	13	1	45.0777	2.66519	9
−7	13	1	41.7968	2.56913	9

−6	−13	1	26.5589	4.80172	10
−6	−13	1	28.5832	6.46894	1
−6	13	1	23.6461	3.51979	7
−6	13	1	23.9705	1.69073	6
−6	13	1	26.3638	1.71851	6
−6	13	1	23.9515	1.86023	9
−5	−13	1	194.761	16.8628	1
−5	−13	1	166.153	13.3194	10
−5	−13	1	181.137	15.2161	2
−5	13	1	181.020	11.1245	6
−5	13	1	184.555	13.0971	12
−5	13	1	183.751	11.3082	9
−5	13	1	187.426	12.2908	7
−5	13	1	180.429	11.1350	6
4	−13	−1	5.71998	3.02548	2
−4	−13	1	18.5439	4.10359	2
−4	−13	1	19.8835	5.10429	1
−4	13	1	9.81789	0.84807	6
−4	13	1	6.66325	1.21342	3
−4	13	1	11.0546	2.02213	7
3	−13	−1	262.183	18.9456	2
−3	−13	1	255.056	18.9214	2
−3	13	1	261.317	16.4384	3
2	−13	−1	40.4404	5.48844	2
−2	13	1	40.6634	3.41166	3
−2	13	1	39.7746	2.89028	4
−2	13	1	40.7207	2.88899	4
1	−13	−1	271.788	19.0284	2
−1	13	1	268.970	17.0853	4
−1	13	1	267.961	17.2532	3
0	−13	−1	213.063	16.1109	2
0	13	−1	215.835	14.2182	3
0	13	1	219.972	14.3687	4
0	13	−1	223.409	14.1207	4
−1	−13	−1	82.2888	7.83789	2
−1	13	−1	77.2334	5.42020	4
−1	13	−1	82.7812	5.50558	4
−1	13	−1	87.5211	5.90737	3
−2	−13	−1	53.8129	6.36776	2
−2	13	−1	46.9003	3.60218	3
−2	13	−1	43.7636	3.54542	3
−3	13	−1	5.74020	0.65029	6
−3	13	−1	8.29696	1.12668	3
−3	13	−1	6.04912	0.95963	3
−4	−13	−1	56.3384	7.27185	2
−4	−13	−1	48.3201	7.70985	1
−4	13	−1	47.9668	3.23701	9
−4	13	−1	51.3569	3.27475	9
−4	13	−1	51.5116	3.16109	6
−4	13	−1	53.0693	3.17953	6

−4	13	−1	50.5405	4.45014	7
−5	−13	−1	11.0633	5.08413	2
−5	−13	−1	17.6120	3.87529	10
−5	−13	−1	7.84990	4.31834	1
−5	13	−1	8.49278	3.74727	12
−5	13	−1	13.0578	2.44744	7
−5	13	−1	9.33365	0.97396	9
−5	13	−1	11.8006	0.89887	9
5	13	1	14.7896	4.44758	13
−6	−13	−1	0.49188	4.78116	1
−6	−13	−1	1.64326	2.76453	10
6	13	1	4.77888	3.27630	13
−6	13	−1	6.20911	2.11763	7
−6	13	−1	2.25190	2.67176	12
−7	−13	−1	2.22958	2.91577	10
7	13	1	2.87304	3.38329	13
−7	13	−1	5.92150	1.74298	7
−8	−13	−1	19.9836	2.96632	10
8	13	1	23.4950	4.24313	13
−8	−14	1	1.72176	2.68461	10
−8	14	1	1.27739	1.76413	7
−7	−14	1	7.36397	3.19964	10
−7	14	1	7.14994	2.20965	7
−7	14	1	4.84482	0.59733	9
−7	14	1	4.90062	0.69521	9
−6	−14	1	80.3068	10.4691	1
−6	−14	1	78.2093	7.61077	10
−6	14	1	79.1886	4.83899	6
−6	14	1	78.8210	6.42690	7
−6	14	1	77.8247	4.97541	9
−6	14	1	74.9215	4.81943	6
−5	−14	1	70.9686	9.80974	1
−5	14	1	75.2067	4.93295	9
−5	14	1	76.6915	4.72135	6
−5	14	1	73.6686	4.70704	6
−5	14	1	78.2720	6.44128	7
−4	14	1	110.770	6.72028	6
−4	14	1	105.628	7.25173	3
−4	14	1	110.487	6.95705	9
−4	14	1	106.208	7.75840	7
3	−14	−1	12.4717	3.99716	2
−3	14	1	13.0957	1.79412	3
−3	14	1	12.2624	1.08865	6
2	−14	−1	39.4507	6.04345	2
−2	14	1	35.9060	2.58522	4
−2	14	1	35.5202	2.56762	4
−2	14	1	35.5371	3.27038	3
1	−14	−1	132.623	10.9630	2
−1	14	1	120.684	8.48281	4
−1	14	1	125.151	8.70799	3

0	−14	−1	55.6702	6.38040	2
0	14	−1	43.8850	4.24371	4
0	14	1	64.1215	4.70255	4
−1	−14	−1	120.284	9.92783	2
−1	14	−1	112.627	7.77107	3
−1	14	−1	107.439	7.22829	4
−1	14	−1	109.819	7.26821	4
−2	−14	−1	2.16674	2.82976	2
−2	14	−1	2.04418	0.90465	3
−3	−14	−1	50.3152	6.93948	2
−3	14	−1	40.9457	3.12460	3
−3	14	−1	43.2138	2.70934	6
−3	14	−1	40.9641	3.14233	3
−4	−14	−1	3.17638	3.20315	2
−4	−14	−1	5.37175	4.00656	1
−4	14	−1	4.09550	1.70969	7
−4	14	−1	5.22084	0.52370	6
−4	14	−1	4.61647	0.55674	9
−4	14	−1	4.53299	0.46730	6
−4	14	−1	4.07077	0.66764	9
−5	−14	−1	15.9374	5.33383	1
−5	−14	−1	14.6283	3.77266	10
−5	14	−1	15.6440	3.36188	12
−5	14	−1	19.8224	2.80562	7
−5	14	−1	15.9748	1.11589	9
−5	14	−1	15.0443	1.13001	9
−6	−14	−1	10.9873	4.85659	1
−6	−14	−1	10.0349	2.81846	10
−6	14	−1	4.71676	2.94184	12
−6	14	−1	7.24182	2.29335	7
−7	−14	−1	8.24554	2.58700	10
−8	−14	−1	52.5066	5.01292	10
−8	−14	−1	54.6288	5.13033	10
−8	−15	1	−1.1441	2.30379	10
−7	−15	1	3.25101	2.95096	10
−7	15	1	6.29124	2.18877	7
−6	−15	1	6.18457	2.51619	10
−6	−15	1	8.17936	4.92662	1
−6	15	1	4.53775	0.74419	9
−6	15	1	4.24442	0.66994	9
−6	15	1	4.50831	0.51454	6
−6	15	1	4.74069	0.49190	6
−6	15	1	2.46078	2.08110	7
−5	15	1	63.0141	3.94355	6
−5	15	1	58.5886	5.35029	7
−5	15	1	58.9949	3.93616	6
−5	15	1	68.3668	4.20579	9
−4	15	1	89.9915	5.97807	9
−4	15	1	90.9074	5.68600	6
4	15	−1	98.0084	9.04384	10

−4	15	1	88.0476	6.84152	7
−3	15	1	20.9306	2.78771	7
−3	15	1	16.0925	2.10605	3
−3	15	1	14.4554	1.82445	9
−3	15	1	17.0417	1.30132	6
2	−15	−1	313.204	22.5620	2
−2	15	1	324.073	20.3703	3
1	−15	−1	13.0805	4.03337	2
−1	15	1	13.5764	2.10018	4
0	−15	−1	13.7053	3.59677	2
0	15	1	11.1726	1.99335	4
0	15	−1	9.71663	1.88131	4
−1	−15	−1	1.62405	2.79278	2
−1	15	−1	1.16155	0.80108	4
−1	15	−1	1.09790	0.94517	3
−1	15	−1	1.92198	0.81561	4
−2	−15	−1	24.6268	4.99469	2
−2	15	−1	18.0332	2.11401	3
−2	15	−1	18.6764	1.42972	6
−3	−15	−1	3.12308	2.94628	2
−3	15	−1	5.43722	0.63075	6
−3	15	−1	4.43766	0.88580	9
−3	15	−1	4.90685	0.55463	6
−3	15	−1	5.03380	0.98044	3
−3	15	−1	5.28777	0.98496	3
−4	−15	−1	258.795	20.1513	1
−4	−15	−1	231.387	19.0593	2
−4	15	−1	241.112	14.6290	9
4	15	1	240.668	17.5509	10
−4	15	−1	228.465	14.5452	9
−4	15	−1	235.096	14.4891	6
−4	15	−1	244.634	14.4694	6
−4	15	−1	229.027	16.0787	12
−5	−15	−1	7.55649	4.70783	1
−5	−15	−1	12.8757	3.54415	10
−5	15	−1	5.29705	0.63757	9
−5	15	−1	5.90172	0.57404	9
−5	15	−1	4.60962	2.90772	12
−6	−15	−1	271.321	21.6975	1
−6	−15	−1	278.345	18.9105	10
−6	15	−1	258.277	18.0659	12
−7	−15	−1	60.1432	6.41179	10
−8	−15	−1	9.78467	2.58793	10
−8	−15	−1	7.02762	2.23319	10
−8	−16	1	5.27488	1.96364	10
−7	−16	1	4.63245	2.54604	10
−6	−16	1	122.693	10.7583	10
−6	−16	1	130.003	13.6828	1
−6	16	1	131.272	8.07067	9
−6	16	1	131.427	8.08105	9

−6	16	1	131.821	9.44198	7
−6	16	1	133.507	7.94243	6
−6	16	1	128.386	7.93558	6
−5	−16	1	20.9538	6.80046	1
−5	16	1	7.87980	0.79273	6
5	16	−1	14.8252	4.34302	10
−5	16	1	9.78981	2.34578	7
−5	16	1	8.62154	0.86419	6
−5	16	1	8.91965	1.17522	9
−4	−16	1	6.42923	2.50931	13
−4	16	1	8.01921	2.13805	7
−4	16	1	4.76297	1.08426	9
4	16	−1	8.56691	4.07576	10
−4	16	1	6.82998	0.72377	6
−3	16	1	46.0945	3.00971	6
−3	16	1	44.2371	3.44255	9
−2	16	1	49.4723	4.28544	3
1	−16	−1	91.8845	10.2160	2
−1	16	1	94.2794	6.66074	4
0	−16	−1	269.936	19.2615	2
0	16	1	261.362	17.1144	4
0	16	−1	254.140	16.9108	4
−1	−16	−1	81.9084	8.75592	2
−1	16	−1	82.7293	6.38326	3
−1	16	−1	87.8063	5.64545	4
−1	16	−1	88.6133	5.64765	4
−2	−16	−1	5.46422	3.00538	2
−2	16	−1	3.32027	1.05008	9
−2	16	−1	4.00002	1.24295	3
−2	16	−1	4.19708	0.60879	6
−3	−16	−1	13.5730	4.15217	2
−3	16	−1	4.31582	0.57973	6
−3	16	−1	2.86052	0.97375	9
3	16	1	11.0467	4.22602	10
−4	16	−1	40.4556	2.57027	6
4	16	1	56.9251	7.30272	10
−4	16	−1	40.3582	5.38355	12
−4	16	−1	39.4603	2.61172	6
−4	16	−1	39.1709	2.82529	9
−4	16	−1	40.2404	2.68126	9
−5	−16	−1	4.08577	4.33275	1
−5	16	−1	3.14372	3.52563	12
−6	−16	−1	49.8121	5.51523	10
−7	−16	−1	17.3640	3.51676	10
−8	−16	−1	16.6403	2.84338	10
−8	−16	−1	17.5016	3.03221	10
−8	−17	1	3.43484	2.24409	10
−7	−17	1	−1.7095	2.51742	10
−6	−17	1	41.2793	5.13207	10
6	17	−1	41.5570	5.88267	10

−6	17	1	45.0703	2.84818	9
−6	17	1	44.5439	2.83649	9
−5	−17	1	38.7376	8.17570	1
−5	17	1	24.5435	2.02568	9
5	17	−1	39.0949	6.47426	10
−5	17	1	24.2291	1.76587	6
−5	17	1	24.9921	1.75964	6
−4	−17	1	11.8544	2.67837	13
4	17	−1	17.1633	4.20106	10
−3	−17	1	14.6342	3.59369	13
−3	17	1	25.5566	1.69601	6
3	17	−1	21.5069	4.32925	10
−3	17	1	24.4677	2.44858	9
−2	17	1	197.481	13.2427	3
0	17	1	2.06219	1.37949	4
−1	−17	−1	27.7169	5.36404	2
−1	17	−1	24.2197	3.77740	3
−2	−17	−1	17.1881	4.71362	2
2	17	1	21.8180	4.24387	10
−2	17	−1	18.2192	1.45261	6
−2	17	−1	17.4691	2.13826	9
−3	−17	−1	109.436	11.3820	2
−3	−17	−1	101.710	8.77048	13
3	17	1	109.117	9.90020	10
−3	17	−1	98.0956	8.61020	12
−3	17	−1	111.286	6.66893	6
−3	17	−1	112.721	7.02123	9
−4	−17	−1	44.0288	7.55595	13
−4	17	−1	44.7620	3.16522	9
−4	17	−1	49.8841	3.25232	9
−4	17	−1	46.3069	2.95509	6
4	17	1	39.4176	6.96293	10
−4	17	−1	51.1244	5.69679	12
−4	17	−1	48.0413	2.99917	6
5	17	1	35.8583	6.62255	10
−6	−17	−1	19.9787	3.98632	10
−7	−17	−1	60.3142	5.53759	10
−7	−18	1	11.5549	3.08471	10
6	18	−1	9.44458	4.22425	10
−5	18	1	80.7137	5.14636	6
−5	18	1	80.7923	5.12826	6
−5	18	1	84.7387	5.38931	9
5	18	−1	87.4952	9.02014	10
−4	−18	1	51.2491	5.40469	13
−4	18	1	47.9692	3.61617	9
4	18	−1	47.4107	5.92738	10
−4	18	1	48.3417	3.17870	6
−3	−18	1	12.4468	3.21454	13
−3	18	1	7.24135	1.29684	9
3	18	−1	10.7816	3.89810	10

−3	18	1	6.42568	0.87914	6
−2	18	1	110.573	6.87807	11
−2	18	1	109.819	6.98853	11
2	18	−1	97.1871	9.13476	10
1	18	−1	32.2422	4.70282	10
−1	18	−1	35.6997	3.22657	11
−2	18	−1	39.5027	3.26146	9
−2	18	−1	38.7323	2.63951	6
−3	−18	−1	14.9072	3.40055	13
−3	18	−1	21.8499	2.24579	9
−3	18	−1	21.5411	1.49432	6
3	18	1	16.1114	4.29753	10
−4	18	−1	22.7609	1.58047	6
4	18	1	19.2101	4.70253	10
−4	18	−1	21.8769	1.91982	9
−4	18	−1	23.6945	1.54477	6
5	18	1	18.4052	5.02794	10
−6	−18	−1	4.03829	3.14053	10
−7	−18	−1	2.37941	2.21448	10
−7	−18	−1	4.47610	2.65560	10
−7	−19	1	2.88408	2.45250	10
6	19	−1	3.24322	4.06699	10
−5	19	1	66.4423	4.18122	6
−5	19	1	66.6432	4.20042	6
−5	19	1	68.7098	4.44594	9
−4	−19	1	134.139	11.0573	13
−4	19	1	162.800	9.18511	6
−4	19	1	155.186	9.53246	9
4	19	−1	134.844	12.1578	10
−3	19	1	47.8539	3.73275	9
−3	19	1	49.2031	3.27451	6
−2	−19	1	49.8700	5.48051	13
−2	19	1	48.5432	3.34972	11
−2	19	1	45.6020	3.47465	11
2	19	−1	58.4937	6.39478	10
−1	−19	1	68.4315	7.00787	13
1	19	−1	73.1734	6.82196	10
0	19	1	3.77664	2.61343	10
0	19	−1	3.74340	2.08156	11
−1	−19	−1	144.476	10.0847	13
1	19	1	111.777	10.0582	10
−2	−19	−1	36.4095	5.69305	13
−2	19	−1	51.2253	3.23970	6
2	19	1	50.2702	6.36229	10
−2	19	−1	49.2856	3.72670	9
−3	−19	−1	69.1824	6.50054	13
3	19	1	65.1270	7.48222	10
−3	19	−1	60.5793	4.00843	6
−3	19	−1	60.1402	4.43640	9
−4	−19	−1	46.1476	5.17221	13

-4	19	-1	47.0446	2.93778	6
-4	19	-1	48.3544	3.26500	9
4	19	1	44.0325	6.78783	10
-4	19	-1	44.6401	2.94517	6
5	19	1	8.42006	4.34388	10
-6	-19	-1	18.2082	3.60528	10
6	19	1	15.3126	4.18193	10
-7	-19	-1	2.83740	2.03852	10
-7	-19	-1	5.14909	2.43311	10
6	20	-1	2.94201	3.47162	10
-5	20	1	20.2366	1.31265	6
5	20	-1	9.97261	4.19271	10
-5	20	1	19.2040	1.31686	6
-5	20	1	20.1894	1.63595	9
-4	-20	1	26.0628	5.00385	13
-4	20	1	33.5856	2.78825	9
4	20	-1	23.4036	5.23953	10
-4	20	1	37.0787	2.21175	6
-3	-20	1	164.054	11.8622	13
-3	20	1	161.880	10.6442	9
3	20	-1	162.614	12.9213	10
-3	20	1	171.321	10.2483	6
-2	-20	1	36.0247	4.52137	13
2	20	-1	23.2407	4.39674	10
-1	-20	1	465.348	29.2280	13
1	20	-1	458.039	29.4047	10
-1	20	1	446.305	28.2835	11
0	-20	-1	5.95879	2.81809	13
0	20	1	7.01295	2.67333	10
-1	-20	-1	32.3589	4.66216	13
-1	20	-1	38.2033	2.75406	11
-1	20	-1	37.0165	2.95599	11
1	20	1	30.6336	4.88241	10
-2	-20	-1	13.7476	2.94759	13
-2	20	-1	8.10292	1.47909	9
-2	20	-1	8.27468	0.98417	6
-3	-20	-1	15.6267	2.97091	13
-3	20	-1	9.20615	0.98299	6
-3	20	-1	7.22463	1.36108	9
-4	-20	-1	4.80724	3.38481	13
-4	20	-1	0.50923	0.33075	6
4	20	1	2.99539	3.70835	10
-4	20	-1	1.86250	0.72378	9
-4	20	-1	1.06282	0.35098	6
5	20	1	1.10761	2.61913	10
6	20	1	1.18230	2.34215	10
6	21	-1	7.05874	3.40865	10
-5	21	1	8.11654	1.00196	9
-5	21	1	5.55247	0.75035	9
5	21	-1	9.59483	4.08899	10

-4	-21	1	2.74981	2.40161	13
-4	21	1	3.26412	1.04086	9
4	21	-1	2.62985	3.48932	10
-4	21	1	5.04282	0.62565	6
-3	-21	1	56.9350	6.24914	13
-3	21	1	67.6901	4.70829	9
-3	21	1	68.1323	4.04813	6
3	21	-1	46.7589	6.81594	10
-2	-21	1	291.067	19.5987	13
2	21	-1	296.780	20.0094	10
-1	-21	1	70.9321	6.34844	13
1	21	-1	57.4743	6.00153	10
-1	21	1	46.8221	4.34536	11
0	-21	-1	16.1329	3.34569	13
0	21	1	12.5874	3.01615	10
0	21	-1	13.3689	2.19469	11
-1	-21	-1	27.7665	4.82579	13
-1	21	-1	41.0891	2.70246	11
-1	21	-1	42.6512	2.76470	11
-2	-21	-1	10.1062	3.22978	13
-2	21	-1	14.6439	1.93304	9
2	21	1	9.92246	3.40991	10
-3	-21	-1	15.9553	3.52462	13
-3	21	-1	9.84563	1.04203	6
-3	21	-1	9.87056	1.52457	9
3	21	1	8.52894	3.83305	10
-4	21	-1	3.75680	0.84225	9
4	21	1	-1.7761	3.46292	10
-4	21	-1	4.13501	0.67889	9
5	21	1	10.8599	3.10111	10
6	21	1	12.1338	2.85905	10
6	22	-1	2.35444	2.73237	10
-5	22	1	6.41484	0.70214	9
5	22	-1	4.47157	3.30153	10
-5	22	1	6.14096	0.65073	9
-4	-22	1	40.7497	4.66917	13
4	22	-1	41.3524	5.75172	10
-4	22	1	37.7419	2.48077	6
-4	22	1	34.0360	2.75757	9
-3	-22	1	14.5315	3.11103	13
-3	22	1	10.9974	1.71902	9
3	22	-1	14.3696	3.64804	10
-3	22	1	12.1823	1.30362	6
2	22	-1	10.8192	3.30582	10
-1	-22	1	112.612	9.03936	13
-1	22	1	122.172	7.81492	11
-1	22	1	116.613	7.76666	11
1	22	-1	118.833	9.14819	10
0	-22	-1	8.86979	2.59895	13
0	22	1	10.5039	2.41681	11

-1	-22	-1	2.11215	2.27524	13
1	22	1	2.99678	2.44406	10
-2	-22	-1	27.4565	4.25447	13
2	22	1	33.2056	5.21345	10
-3	-22	-1	-1.5500	2.27582	13
-3	22	-1	-0.7783	0.75064	9
3	22	1	2.42973	3.11484	10
-3	22	-1	0.47636	0.46715	6
4	22	1	20.9203	3.80824	10
-4	22	-1	22.4331	1.59672	9
-4	22	-1	15.9449	1.42499	9
5	22	1	18.0622	3.44863	10
5	23	-1	34.7488	4.93032	10
-4	-23	1	32.7519	4.03926	13
-4	23	1	27.2463	1.90697	6
4	23	-1	32.3921	4.91420	10
-4	23	1	26.3346	1.89528	6
-4	23	1	28.6094	2.30389	9
-3	-23	1	4.56116	2.42125	13
-3	23	1	2.41286	1.24025	9
3	23	-1	3.45289	2.10128	10
-3	23	1	2.09428	0.65249	6
-2	-23	1	24.2852	4.67309	13
2	23	-1	24.6299	4.21111	10
1	-23	-1	22.1057	4.06365	13
-1	-23	1	21.9636	3.56222	13
1	23	-1	20.7431	2.84080	11
-1	23	1	25.7152	1.98072	11
-1	23	1	24.0087	1.99403	11
0	-23	1	20.4042	3.18784	13
0	-23	-1	13.2716	3.44544	13
0	23	-1	22.1291	2.52738	11
0	23	1	12.5465	2.46462	11
0	23	-1	18.3707	2.04735	11
-1	-23	-1	10.3432	2.87697	13
1	23	1	7.41819	3.10690	10
-2	-23	-1	14.2813	3.03376	13
-2	23	-1	12.1848	1.74335	9
2	23	1	16.1183	3.47495	10
-3	-23	-1	8.36221	2.62440	13
-3	23	-1	10.0058	0.90796	6
3	23	1	2.23554	2.95795	10
-3	23	-1	7.96746	1.26488	9
-3	23	-1	8.66636	0.82350	6
4	23	1	12.3128	3.47832	10
-4	23	-1	10.2230	0.87226	9
-4	23	-1	10.0215	0.93386	9
5	23	1	1.36468	1.93423	10
5	24	-1	5.68754	1.96524	10
-4	-24	1	-0.8064	1.71908	13

−4	24	1	0.68575	0.30045	6
−4	24	1	0.44880	0.31722	6
−4	24	1	−0.1682	0.69107	9
−4	24	1	0.06536	0.31261	6
4	24	−1	−0.7533	1.60939	10
−3	−24	1	14.0081	3.01757	13
−3	24	1	6.30358	0.74267	6
3	24	−1	8.24201	2.78563	10
−3	24	1	5.74011	1.18770	9
−2	−24	1	11.4376	2.77763	13
2	24	−1	9.70774	2.88987	10
−1	−24	1	17.2998	3.14769	13
1	−24	−1	21.0336	3.81830	13
1	24	−1	16.1065	2.14817	11
0	−24	−1	17.7562	3.03891	13
0	−24	1	17.0464	3.35608	13
0	24	−1	16.1604	1.50405	11
0	24	−1	13.4292	1.46527	11
0	24	1	16.5014	2.05678	11
−1	−24	−1	0.43142	2.12099	13
1	24	1	0.85859	1.90181	10
1	24	1	1.66327	1.85473	11
−2	−24	−1	7.05735	2.61564	13
2	24	1	4.98528	2.61093	10
−2	24	−1	8.09133	1.34951	9
−3	−24	−1	19.1466	2.94356	13
3	24	1	14.9055	2.77881	10
−3	24	−1	14.1037	1.69363	9
4	24	1	10.6812	3.06860	10
5	24	1	−0.6086	3.21238	10
−4	−25	1	1.59545	2.11469	13
4	25	−1	1.29576	1.94723	10
−4	25	1	2.16311	0.53568	9
−3	−25	1	14.7530	2.47176	13
3	25	−1	11.5112	2.40792	10
−3	25	1	9.52090	1.01763	6
−3	25	1	8.28519	1.33160	9
−2	−25	1	2.23212	2.12895	13
2	25	−1	0.19905	1.42846	11
1	−25	−1	6.61420	2.32624	13
1	25	−1	7.66518	1.45960	11
0	−25	−1	8.15969	2.62187	13
0	−25	1	4.75343	2.40362	13
0	25	1	6.34334	1.15398	11
−1	−25	−1	0.78878	2.12157	13
1	25	1	−2.3295	1.30737	11
−2	−25	−1	1.91885	2.11260	13
−2	25	−1	0.00606	0.93460	9
2	25	1	1.65508	1.93246	10
−2	25	−1	1.04364	0.54350	6

−3	−25	−1	3.61317	1.98784	13
3	25	1	1.89810	2.24560	10
−3	25	−1	4.21427	0.45294	6
−3	25	−1	3.81856	0.77220	9
4	25	1	13.9072	2.70286	10
−3	−26	1	1.40437	1.73567	13
3	26	−1	1.66418	1.25541	11
−2	−26	1	8.28242	2.16331	13
2	26	−1	5.53235	1.40318	11
−1	−26	1	3.60221	2.01481	13
1	−26	−1	5.35428	2.05196	13
0	−26	−1	0.66507	2.05880	13
0	−26	1	−0.4068	1.82286	13
−1	−26	−1	−0.2312	1.88447	13
1	26	1	0.63036	1.12711	11
2	26	1	2.35089	1.31019	11
−2	26	−1	1.43133	0.85602	9
−3	−26	−1	10.4278	2.05987	13
3	26	1	7.82476	1.88096	10
−2	−27	1	7.74014	1.91195	13
1	−27	−1	0.21078	1.49222	13
−1	−27	1	2.92319	1.72294	13
0	−27	−1	30.8000	3.75452	13
0	−27	1	34.9665	3.69601	13
−1	−27	−1	15.0773	2.66687	13
−2	−27	−1	1.13770	1.51571	13
2	27	1	1.71084	0.93129	11
0	−28	1	1.16252	1.45650	13
0	−28	−1	−0.1542	1.67852	13
10	0	−2	1.96962	2.57103	13
9	0	−2	1.93576	2.96907	13
8	0	−2	10.5659	3.71039	13
8	0	−2	5.90507	3.71258	13
−7	0	2	2.57008	1.94412	10
−6	0	2	1.02814	1.59325	4
−5	0	2	131.437	10.0221	1
−5	0	2	139.398	9.30402	4
−4	0	2	887.984	56.8251	1
−4	0	2	946.745	56.0343	4
−4	0	2	906.922	55.9707	3
−3	0	2	5.23576	1.16553	4
−3	0	2	5.64345	1.30065	4
−3	0	2	4.84854	1.60734	5
−3	0	2	4.18253	0.95736	3
−3	0	2	5.27219	1.45692	5
−2	0	2	6242.49	380.113	3
−2	0	2	6362.25	379.257	4
−2	0	2	6273.52	383.634	5
−2	0	2	6168.93	379.362	4
−1	0	2	54.3614	4.28724	4

−1	0	2	45.0868	4.86745	5
0	0	−2	1999.12	118.763	5
0	0	−2	1962.80	117.333	4
0	0	−2	1834.08	117.715	5
−1	0	−2	8.64303	1.53947	5
−1	0	−2	6.77835	1.40163	4
−1	0	−2	7.19366	1.06115	3
−1	0	−2	7.51737	1.23046	4
−1	0	−2	7.86198	1.22150	3
−2	0	−2	7069.21	425.435	2
−2	0	−2	7145.17	424.546	3
−2	0	−2	6861.43	424.219	3
−2	0	−2	7161.21	424.787	4
−2	0	−2	6750.77	425.153	4
−3	0	−2	13.7194	1.85164	4
−3	0	−2	8.17474	2.50970	2
−3	0	−2	10.9189	1.48349	3
−3	0	−2	12.0566	1.64188	4
−4	0	−2	59.3245	4.69796	4
−4	0	−2	62.2116	4.73920	4
−4	0	−2	59.8268	6.39749	2
−4	0	−2	58.4696	4.61848	3
−5	0	−2	0.03633	1.13397	4
−5	0	−2	0.50223	1.06260	3
−6	0	−2	169.128	11.0611	8
−7	0	−2	0.21117	1.43961	8
−8	0	−2	1.17975	1.31978	8
9	0	2	3.24852	2.68209	13
10	−1	−2	0.62668	2.44393	13
10	1	−2	2.11785	2.63830	13
9	−1	−2	8.54762	3.85467	13
−9	1	2	4.09199	1.73398	7
9	1	−2	1.56111	3.30316	13
8	1	−2	3.40106	2.97847	13
−7	−1	2	16.3398	3.22624	10
−7	1	2	12.1710	1.81495	11
−6	−1	2	53.8489	5.43333	1
−6	−1	2	50.0851	4.75421	4
−6	1	2	57.5851	4.71878	4
−5	−1	2	1149.67	68.1848	4
−5	−1	2	1123.48	69.1209	1
−5	1	2	1039.17	68.2321	1
−5	1	2	1125.75	67.9469	4
−4	−1	2	1039.81	61.5991	4
−4	−1	2	1012.63	61.7710	5
−4	−1	2	967.630	62.5134	1
−4	−1	2	1011.87	61.6376	5
−4	1	2	1034.74	61.4248	4
−4	1	2	1010.94	61.4243	3
−4	1	2	953.873	62.0340	1

−3	1	2	80.5578	5.81555	4
−3	1	2	81.9261	5.57763	4
−3	1	2	83.0777	5.55343	3
−3	1	2	77.2304	5.38652	5
−3	1	2	83.4418	5.51236	5
−2	−1	2	7588.45	425.866	4
−2	−1	2	6909.93	426.252	3
−2	−1	2	7435.35	428.156	5
−2	1	2	6858.94	428.071	5
−2	1	2	6736.25	426.350	3
−2	1	2	6686.86	426.513	4
−2	1	2	6977.92	426.619	4
−1	−1	2	1097.81	66.8881	5
−1	1	2	1107.73	66.1049	4
−1	1	2	1061.13	66.6857	5
0	−1	−2	2022.52	126.692	5
0	1	−2	2146.01	128.398	5
0	1	−2	2238.52	128.454	5
0	1	2	1987.24	126.922	5
0	1	−2	2054.70	126.749	4
0	1	−2	2052.62	126.578	4
0	1	−2	2096.80	126.306	3
−1	−1	−2	61.3401	5.30448	5
−1	−1	−2	68.6261	5.18331	5
−1	−1	−2	66.1686	4.75070	3
−1	−1	−2	69.9766	4.76351	4
−1	−1	−2	69.4654	4.89272	4
−1	1	−2	76.1214	4.73997	4
−1	1	−2	79.7561	4.63963	3
−1	1	−2	76.5124	4.58977	3
−2	−1	−2	117.192	8.49682	2
−2	−1	−2	100.427	7.46538	3
−2	−1	−2	126.522	7.62122	4
−2	−1	−2	114.146	7.59646	3
−2	1	−2	122.785	7.42197	3
−2	1	−2	112.887	8.29275	2
−2	1	−2	123.307	7.39481	4
−2	1	−2	108.821	7.36713	3
−3	−1	−2	473.795	30.0927	2
−3	−1	−2	465.717	28.8904	3
−3	−1	−2	479.652	28.8969	4
−3	−1	−2	499.201	29.0778	4
−3	1	−2	447.009	28.6255	4
−3	1	−2	465.344	28.7286	3
−3	1	−2	466.575	29.9642	2
−3	1	−2	434.786	28.6267	4
−3	1	−2	481.677	28.8115	4
−3	1	−2	454.550	28.7229	3
−4	−1	−2	121.970	8.58418	3
−4	−1	−2	129.418	8.76472	4

−4	−1	−2	118.180	9.09587	1
−4	−1	−2	131.772	10.3517	2
−4	1	−2	128.852	8.45703	4
−4	1	−2	120.106	8.21889	4
−4	1	−2	132.154	8.46130	3
−5	1	−2	7.46069	1.56472	4
−5	1	−2	6.52349	1.54432	3
−5	1	−2	11.2852	1.59409	4
−6	−1	−2	29.9270	2.96950	8
−6	1	−2	22.9009	2.65682	8
−7	−1	−2	158.303	10.6497	8
−7	1	−2	159.713	10.4661	8
−8	−1	−2	9.59905	1.70173	8
8	1	2	13.0296	3.72283	13
−8	1	−2	11.6986	1.64317	8
−9	−1	−2	33.9103	2.66559	8
9	1	2	26.8088	5.06736	13
10	−2	−2	−0.7345	2.76879	13
10	2	−2	7.09272	3.38583	13
−9	2	2	7.34376	1.45552	8
−9	2	2	7.40047	1.59105	7
9	2	−2	3.29803	3.46258	13
−8	−2	2	105.631	7.95007	10
−8	2	2	109.552	7.83458	7
8	2	−2	98.3099	9.70914	13
−7	−2	2	3.01634	1.84379	10
−7	2	2	4.39342	1.18872	11
−6	−2	2	92.6614	8.30639	1
−6	2	2	100.044	7.02814	4
−5	−2	2	15.6991	3.34421	1
−5	2	2	10.6138	1.83179	3
−5	2	2	10.6280	1.76057	4
−4	−2	2	145.900	11.9977	1
−4	−2	2	148.803	10.7730	4
−4	−2	2	149.884	11.6067	5
−4	2	2	157.715	11.0042	1
−4	2	2	177.224	10.5830	3
−4	2	2	179.161	10.7810	4
−4	2	2	177.119	10.5802	4
−3	−2	2	1757.25	112.551	4
−3	−2	2	1876.67	113.719	5
−3	−2	2	1751.84	112.464	3
−3	−2	2	1891.42	113.488	2
−3	2	2	1886.40	112.364	4
−3	2	2	1909.45	112.506	4
−3	2	2	1886.58	112.350	3
−2	−2	2	596.739	38.6826	2
−2	−2	2	628.963	38.2529	4
−2	−2	2	620.622	39.2856	5
−2	2	2	632.185	38.2341	4

−2	2	2	617.714	38.0750	4
−2	2	2	633.077	38.0556	3
−2	2	2	630.644	38.2849	5
−1	−2	2	4176.62	257.515	5
−1	2	2	4235.50	257.619	3
1	2	−2	4196.63	257.446	4
−1	2	2	4103.24	259.384	5
−1	2	2	4342.16	257.340	4
−1	2	2	4405.96	257.618	4
0	−2	−2	16.9881	2.19391	5
0	2	−2	12.3653	1.47535	4
0	2	−2	10.6726	1.58321	4
0	2	−2	12.1214	1.62623	5
0	2	−2	12.4877	1.55358	5
0	2	−2	11.8847	1.42707	3
−1	−2	−2	5785.19	359.940	5
−1	−2	−2	5931.85	357.674	4
−1	−2	−2	5992.45	358.818	5
−1	−2	−2	6054.80	357.203	3
−1	−2	−2	5822.76	358.876	2
−1	2	−2	5673.61	356.849	4
1	2	2	5698.52	359.962	5
−1	2	−2	6134.85	356.876	3
−1	2	−2	5856.27	356.509	3
−1	2	−2	5900.55	356.675	4
−2	−2	−2	64.8359	4.89230	3
−2	−2	−2	58.3362	4.98911	4
−2	−2	−2	57.1041	5.31198	5
−2	−2	−2	64.2921	5.33817	5
−2	−2	−2	58.6977	4.70913	4
−2	−2	−2	56.5824	4.65523	3
−2	−2	−2	56.9868	5.47034	2
−2	2	−2	76.2919	5.51101	2
−2	2	−2	79.4924	4.42136	3
−2	2	−2	77.2438	4.51098	4
−2	2	−2	79.4815	4.39810	3
−3	−2	−2	443.362	29.7026	2
−3	−2	−2	428.738	28.4304	5
−3	−2	−2	454.142	28.6927	4
−3	−2	−2	449.257	28.4435	5
−3	−2	−2	448.537	28.5768	3
−3	2	−2	481.434	28.2662	3
−3	2	−2	469.603	28.2546	3
−3	2	−2	483.291	28.1610	4
−3	2	−2	477.845	28.2627	4
−4	−2	−2	129.840	9.92673	1
−4	−2	−2	132.289	9.25412	4
−4	−2	−2	137.332	10.8253	2
−4	2	−2	127.977	8.45376	4
−4	2	−2	131.921	8.57703	4

−4	2	−2	133.920	8.72520	3
−5	−2	−2	632.538	41.3478	1
−5	−2	−2	606.158	41.0404	4
−5	−2	−2	637.704	43.1050	2
−5	2	−2	722.659	40.8578	3
−5	2	−2	657.050	40.1918	4
−5	2	−2	685.962	40.1915	4
−6	−2	−2	63.9103	5.30848	8
−6	2	−2	78.8430	5.21717	8
−6	2	−2	75.6217	5.94815	3
−7	−2	−2	3.85149	1.83983	8
7	2	2	5.11451	3.94439	13
−8	−2	−2	6.96324	1.59304	8
−8	2	−2	4.87152	1.27374	8
−9	−2	−2	1.02657	1.20311	8
9	2	2	2.24008	2.92865	13
10	3	−2	23.8576	4.19125	13
−9	3	2	7.75878	1.60999	7
−9	3	2	9.39165	1.45175	8
9	3	−2	2.73604	3.60156	13
−8	−3	2	220.617	14.3564	10
8	3	−2	216.341	15.7288	13
−8	3	2	200.209	14.0894	7
−7	−3	2	6.11324	2.16838	10
−7	3	2	8.41174	1.22251	11
7	3	−2	2.67487	3.77793	13
7	3	−2	4.69814	3.68129	13
−6	−3	2	345.724	21.9603	1
−6	3	2	284.469	19.7056	4
−5	−3	2	101.185	7.50788	5
−5	−3	2	104.494	9.18393	1
−5	3	2	105.887	7.56524	4
−5	3	2	105.293	7.14885	4
−5	3	2	103.085	7.18207	3
−4	−3	2	60.6778	6.61534	1
−4	−3	2	62.4733	6.58330	5
−4	−3	2	63.9896	6.80666	2
−4	3	2	75.7127	5.23038	4
−4	3	2	77.0836	5.09717	4
−4	3	2	78.8017	5.04711	3
−3	−3	2	9.89626	2.64282	5
−3	−3	2	7.74092	1.55548	4
−3	−3	2	7.52662	2.15547	2
−3	3	2	8.06089	1.25838	4
−3	3	2	7.61966	1.40390	4
−2	−3	2	1852.51	116.770	2
−2	−3	2	1830.11	116.354	4
−2	−3	2	1871.24	117.524	5
−2	3	2	1925.90	116.195	3
−2	3	2	2010.99	116.290	4

−2	3	2	1922.00	116.142	4
−2	3	2	2000.63	116.141	3
1	−3	−2	547.601	35.6585	2
−1	−3	2	551.347	36.0475	5
−1	3	2	603.488	35.1649	4
−1	3	2	576.181	35.2886	4
1	3	−2	578.887	35.2374	4
−1	3	2	573.581	35.1268	3
−1	3	2	597.083	35.5694	5
0	−3	−2	1197.78	67.8254	5
0	3	−2	1100.89	67.1201	4
0	3	−2	1064.71	67.1048	3
0	3	−2	1074.63	67.2375	5
0	3	2	1219.72	67.9725	5
0	3	−2	963.524	67.0360	3
0	3	2	1240.65	67.2913	4
0	3	−2	1073.01	67.0801	4
0	3	−2	1018.47	67.1466	5
−1	−3	−2	105.223	6.52599	4
−1	−3	−2	106.717	7.09432	5
−1	−3	−2	107.252	7.15556	2
−1	3	−2	86.8632	5.86464	3
−1	3	−2	88.0395	5.93659	4
−1	3	−2	83.0492	5.89572	4
−1	3	−2	83.7692	5.89090	3
1	3	2	106.033	7.34247	5
−2	−3	−2	1464.90	82.1739	5
−2	−3	−2	1356.36	81.4775	4
−2	−3	−2	1407.30	82.2139	2
−2	−3	−2	1351.66	82.2757	5
−2	3	−2	1295.70	80.8614	3
−2	3	−2	1271.23	80.8025	4
−2	3	−2	1280.93	80.8644	3
−2	3	−2	1256.59	80.8322	4
−3	−3	−2	16.7537	2.55340	4
−3	−3	−2	15.1195	2.06333	3
−3	−3	−2	20.8768	3.15729	5
−3	−3	−2	16.2283	3.03347	5
−3	−3	−2	15.2122	3.00552	1
−3	−3	−2	15.3663	3.00318	2
−3	3	−2	13.9896	1.51377	3
−3	3	−2	13.9389	1.55873	3
−3	3	−2	18.1459	1.43997	4
−4	−3	−2	120.281	10.4240	2
−4	−3	−2	121.244	9.95441	1
−4	−3	−2	114.771	9.07229	4
−4	3	−2	140.482	8.10840	4
−4	3	−2	143.314	8.57465	3
−4	3	−2	131.805	8.09998	4
−5	−3	−2	209.897	14.5044	4

−5	−3	−2	219.385	15.1421	1
−5	−3	−2	216.066	16.3701	2
−5	3	−2	204.373	13.7628	3
−6	−3	−2	84.7958	5.63558	8
−6	3	−2	66.0607	4.93217	8
−6	3	−2	64.3565	4.70549	9
−7	−3	−2	84.1531	6.80541	10
−7	−3	−2	84.1391	6.35116	8
−7	3	−2	88.4582	5.94775	8
7	3	2	82.9324	8.26834	13
−8	−3	−2	21.3796	2.79350	8
8	3	2	20.8164	4.35776	13
−9	3	−2	19.8284	1.72318	6
9	3	2	29.4877	5.16973	13
−9	3	−2	22.5206	2.01994	9
10	4	−2	20.7883	3.55039	13
−9	4	2	17.9888	2.37783	7
−8	−4	2	77.8885	6.90995	10
8	4	−2	76.7274	9.19954	13
−8	4	2	102.321	6.87025	7
−7	−4	2	−0.9221	2.09811	10
−7	−4	2	2.94915	2.50329	1
7	4	−2	3.47467	3.35921	13
−7	4	2	1.82981	0.80131	11
−6	−4	2	99.4339	10.0024	1
−6	4	2	131.331	8.35435	3
−5	−4	2	56.7923	6.49502	1
−5	−4	2	52.9605	5.30255	5
−5	4	2	45.0452	3.71939	4
−5	4	2	49.1838	3.73969	4
−5	4	2	43.9784	3.78367	3
−4	4	2	22.0891	2.16571	4
−4	4	2	21.5907	2.17826	3
−4	4	2	22.2102	2.22582	4
−3	−4	2	873.121	52.8134	5
−3	−4	2	858.316	52.0103	2
−3	4	2	826.871	50.7478	3
−3	4	2	798.573	50.8377	4
−3	4	2	806.583	50.7112	4
−2	−4	2	1704.07	108.402	5
−2	−4	2	1683.30	107.578	2
−2	−4	2	1833.95	107.381	4
−2	4	2	1802.97	106.992	3
−2	4	2	1705.74	106.888	3
−2	4	2	1833.11	106.917	4
−2	4	2	1781.56	107.046	4
−1	−4	2	387.931	25.1217	5
1	−4	−2	377.676	24.8078	2
−1	4	2	405.621	24.4484	5
1	4	−2	408.193	24.2460	4

−1	4	2	382.752	24.1041	3
−1	4	2	399.879	24.1310	4
1	4	−2	387.420	24.8493	5
−1	4	2	393.071	24.2750	4
0	−4	−2	3017.34	196.977	2
0	−4	−2	2939.10	197.043	5
0	4	2	3177.25	197.124	5
0	4	2	3180.59	196.542	4
0	4	−2	3546.29	197.197	4
0	4	−2	3447.15	197.071	3
0	4	−2	3414.62	196.369	4
−1	4	−2	38.1278	2.53877	3
−1	4	−2	40.4538	2.57663	4
−1	4	−2	38.3495	2.59221	3
−1	4	−2	40.2181	2.58704	4
−2	−4	−2	352.426	22.7103	3
−2	−4	−2	360.277	22.5200	5
−2	−4	−2	365.458	22.4432	2
−2	−4	−2	348.641	21.6647	4
−2	4	−2	325.783	20.8644	3
−2	4	−2	312.178	20.8645	3
−3	−4	−2	307.743	21.0224	2
−3	−4	−2	297.324	20.8953	1
−3	−4	−2	313.339	20.6817	5
−3	−4	−2	294.743	19.9379	4
−3	4	−2	321.808	19.1670	3
−3	4	−2	330.493	19.1590	3
−4	−4	−2	34.1697	3.29729	5
−4	−4	−2	34.7320	4.50690	1
−4	−4	−2	34.8513	4.92427	2
−4	−4	−2	35.2088	3.75445	5
−4	4	−2	34.4294	2.73492	3
−5	−4	−2	790.606	51.5629	1
−5	−4	−2	849.722	52.9263	2
−5	4	−2	801.908	49.9704	3
−6	4	−2	10.1160	1.02923	6
−6	4	−2	9.56883	1.17568	9
−6	4	−2	9.98276	1.58238	8
−7	−4	−2	501.218	28.8461	8
−7	−4	−2	479.222	29.0644	10
−7	4	−2	417.762	28.0364	9
7	4	2	461.738	29.9728	13
−7	4	−2	431.043	27.8982	6
−8	−4	−2	25.4225	2.96346	8
−8	4	−2	19.9778	1.89077	9
−8	4	−2	19.8079	1.76358	6
8	4	2	24.4540	4.50726	13
−9	−4	−2	1.93567	1.08075	8
9	4	2	0.82866	2.84827	13
−9	4	−2	0.54481	0.45549	6

−9	4	−2	1.11370	0.75394	9
10	5	−2	5.18380	2.79930	13
−9	−5	2	16.5884	3.50473	10
−9	−5	2	16.9918	2.82420	8
9	5	−2	13.7912	4.14234	13
−8	−5	2	116.586	8.95310	10
−8	5	2	112.522	8.53794	7
8	5	−2	127.296	11.1201	13
−7	−5	2	18.4699	3.27998	10
−7	−5	2	23.7847	5.03966	1
7	5	−2	25.6578	5.02386	13
−7	5	2	13.4173	2.44425	7
−6	−5	2	373.863	27.1063	1
−6	5	2	415.256	25.0092	3
−5	−5	2	162.924	13.5796	5
−5	−5	2	169.970	14.2163	2
−5	−5	2	161.382	14.2562	1
−5	5	2	207.777	11.7030	4
−5	5	2	196.817	11.7726	4
−5	5	2	198.832	12.0474	3
−4	−5	2	238.333	17.4784	2
−4	−5	2	241.227	18.0365	1
−4	−5	2	242.324	18.1809	5
−4	5	2	258.374	15.8226	4
−4	5	2	263.777	15.6803	4
−4	5	2	259.484	15.8371	3
−3	5	2	64.0591	4.54252	4
−3	5	2	57.7945	4.38731	3
−3	5	2	68.4552	4.36084	4
−3	5	2	61.0160	4.31961	3
−2	−5	2	383.145	23.7332	5
−2	−5	2	381.399	22.6627	2
−2	5	2	337.901	21.7856	3
−2	5	2	333.866	21.7674	4
−2	5	2	334.438	21.9079	4
−2	5	2	357.205	21.8926	3
1	−5	−2	625.989	39.3811	2
−1	−5	2	647.539	40.2531	5
−1	5	2	639.735	38.6189	3
1	5	−2	624.124	38.7538	4
1	5	−2	639.152	38.7257	4
−1	5	2	613.609	38.6985	3
−1	5	2	673.807	38.8081	4
1	5	−2	592.032	39.2437	5
−1	5	2	636.203	38.6353	4
0	−5	−2	37.6579	4.27369	5
0	−5	−2	37.8097	4.51680	2
0	5	2	41.6247	4.11887	5
0	5	−2	52.9019	3.29632	4
0	5	−2	51.7693	3.29493	4

0	5	−2	55.0322	3.28257	4
0	5	2	39.5747	3.33889	3
0	5	2	38.7234	3.45671	4
0	5	−2	53.9655	3.23140	3
−1	−5	−2	482.670	28.4315	5
−1	−5	−2	479.001	28.3086	2
1	5	2	461.344	28.4991	5
−1	5	−2	424.567	26.9960	3
−1	5	−2	425.891	26.9683	4
−1	5	−2	418.569	27.0251	3
−1	5	−2	423.566	26.9651	4
−2	−5	−2	186.453	12.8848	2
−2	−5	−2	196.842	13.2670	5
−2	5	−2	181.064	11.2207	3
−2	5	−2	174.697	11.2248	3
−3	−5	−2	29.9462	4.30830	5
−3	−5	−2	31.1505	4.58668	2
−3	−5	−2	29.1300	4.29951	1
−3	5	−2	29.5367	2.09174	3
−3	5	−2	27.5632	2.09153	3
−4	−5	−2	325.946	22.7043	2
−4	−5	−2	304.871	22.3114	1
−4	−5	−2	317.561	21.6847	5
−4	5	−2	358.708	20.3098	3
−5	−5	−2	143.470	10.9128	1
−5	−5	−2	151.063	11.8109	2
−5	5	−2	113.111	7.81347	6
−5	5	−2	115.488	8.42480	3
−6	−5	−2	10.2104	3.00084	1
−6	−5	−2	9.28236	4.09654	2
−6	−5	−2	10.8543	2.09724	8
−6	−5	−2	9.27881	2.62229	10
−6	5	−2	7.73079	0.89191	6
−6	5	−2	8.29040	1.05152	9
−7	−5	−2	545.050	36.2393	8
−7	−5	−2	547.651	36.6258	10
−7	5	−2	637.317	35.4417	6
7	5	2	575.419	37.7257	13
−7	5	−2	604.465	35.5758	9
−8	−5	−2	4.66127	1.82991	8
−8	5	−2	7.19144	1.08502	9
−8	5	−2	6.04477	0.81549	6
−8	5	−2	6.95866	0.97906	9
9	5	2	23.2768	3.75281	13
−9	5	−2	18.6719	1.61417	9
−9	5	−2	16.2742	1.36062	6
10	6	−2	−0.1381	2.49390	13
−9	−6	2	1.73878	1.75771	8
−9	−6	2	1.45450	1.74263	10
9	6	−2	3.62249	3.63327	13

−9	6	2	1.26768	0.86821	9
−9	6	2	2.65363	1.68451	7
−8	−6	2	272.715	18.5616	10
−8	6	2	286.146	18.1654	7
8	6	−2	275.280	19.5495	13
−7	−6	2	17.5429	4.15975	1
−7	−6	2	20.7610	3.50958	10
−7	6	2	18.1934	1.95625	9
7	6	−2	17.9192	4.23044	13
−7	6	2	19.9473	2.99684	7
−6	6	2	9.40783	1.19209	9
−6	6	2	7.05280	1.46931	3
−6	6	2	9.55268	1.92427	7
−5	−6	2	34.6389	5.38630	5
−5	−6	2	36.7918	5.42088	2
−5	−6	2	38.0080	5.70005	1
−4	−6	2	48.7122	6.46834	5
−4	−6	2	50.1337	6.49292	1
−4	−6	2	52.6776	6.18741	2
−4	6	2	65.2045	4.36733	3
−4	6	2	67.3645	4.11252	4
−4	6	2	65.1902	4.25281	4
−3	−6	2	77.5230	6.67866	2
−3	−6	2	78.0903	8.12960	5
−3	6	2	75.1345	5.20281	4
−3	6	2	79.8546	5.44247	3
−3	6	2	82.9689	5.44434	4
−3	6	2	80.9262	5.33078	3
−2	−6	2	475.931	28.9265	2
−2	−6	2	464.383	29.9058	5
2	−6	−2	444.961	29.1708	2
−2	6	2	418.296	28.0901	4
−2	6	2	458.055	27.9888	4
−2	6	2	469.412	28.0415	3
−2	6	2	465.113	28.1405	3
1	−6	−2	1.76727	1.96188	2
1	6	−2	4.34696	1.52421	5
1	6	−2	5.03455	1.10784	4
−1	6	2	1.55703	0.96952	4
−1	6	2	1.22711	0.69480	3
−1	6	2	2.23176	0.86458	3
1	6	−2	3.74262	1.06718	4
−1	6	2	2.32020	1.01411	4
0	−6	−2	14.6661	2.95277	2
0	−6	−2	13.9022	2.55448	5
0	6	2	17.0302	2.30912	4
0	6	2	14.7853	1.71144	3
0	6	2	15.5668	1.91799	4
1	6	2	12.5283	2.85541	5
−2	−6	−2	54.9570	5.29952	2

−2	6	−2	52.9333	3.31823	3
−2	6	−2	52.6848	3.30195	3
−3	−6	−2	19.3830	3.88049	2
−3	−6	−2	21.1323	3.81333	5
−3	−6	−2	22.0570	4.56593	1
−3	6	−2	25.1169	1.73381	3
−3	6	−2	26.3494	1.73084	3
−4	−6	−2	79.8759	7.88146	2
−4	−6	−2	75.0093	7.23724	5
−4	−6	−2	72.6508	7.50503	1
−4	6	−2	86.7978	5.27160	3
−5	−6	−2	38.9888	6.14046	2
−5	−6	−2	30.7905	5.61817	1
−5	6	−2	39.6932	2.50148	6
−5	6	−2	39.8271	3.14351	3
−6	−6	−2	205.787	13.8729	10
−6	−6	−2	197.291	13.1648	8
−6	−6	−2	201.395	15.1177	1
−6	−6	−2	197.915	16.9723	2
−6	6	−2	194.719	12.2531	9
−6	6	−2	202.058	12.1574	6
−6	6	−2	186.898	13.1406	7
−7	−6	−2	93.5305	7.33330	8
−7	−6	−2	89.4275	7.54445	10
7	6	2	91.7001	9.25480	13
−7	6	−2	101.227	5.98041	6
−7	6	−2	94.6223	6.13361	9
−8	−6	−2	12.7243	2.67111	10
−8	−6	−2	11.7667	2.13152	8
−8	6	−2	6.70496	1.01248	9
−8	6	−2	6.83004	0.94764	9
−8	6	−2	7.41471	0.80162	6
8	6	2	13.6826	4.00014	13
−9	−6	−2	6.28741	1.64417	8
9	6	2	7.76092	3.00149	13
10	7	−2	16.1377	3.01190	13
−9	−7	2	10.9781	2.39453	10
−9	−7	2	12.5820	1.97703	8
9	7	−2	20.0267	4.20421	13
−8	−7	2	106.824	8.57394	10
−8	7	2	94.6191	7.31355	7
8	7	−2	101.057	9.86700	13
−8	7	2	96.1285	6.46272	9
−8	7	2	100.051	6.27748	6
−7	7	2	49.9927	3.51409	9
−7	7	2	51.8358	4.60395	7
−7	7	2	50.1213	3.21894	6
7	7	−2	32.1494	6.49090	13
−6	−7	2	211.853	16.1677	1
−6	7	2	188.060	11.8867	9

−6	7	2	193.643	11.7058	6
−6	7	2	187.477	12.4938	3
−6	7	2	175.833	12.5857	7
−5	−7	2	26.0613	4.81081	2
−5	−7	2	34.5897	5.37144	5
−5	−7	2	27.5876	5.34573	1
−5	7	2	25.8839	2.59542	3
−4	−7	2	108.235	9.90440	5
−4	−7	2	111.225	8.96091	2
−4	−7	2	104.433	9.52432	1
−4	7	2	103.872	6.93300	3
−4	7	2	100.909	6.56097	4
−4	7	2	98.6043	6.51453	4
−3	7	2	99.4278	6.21425	3
−3	7	2	90.9677	6.04815	4
−3	7	2	91.3152	6.29942	3
−3	7	2	90.8513	6.16959	4
−2	−7	2	222.458	16.6646	2
2	−7	−2	263.661	17.2796	2
−2	7	2	252.470	15.8657	4
−2	7	2	264.815	16.0529	3
−2	7	2	262.426	16.0235	4
−2	7	2	265.023	15.9379	3
−1	−7	2	375.729	22.6849	2
1	−7	−2	325.667	23.1989	2
1	7	−2	390.309	22.3553	4
1	7	−2	398.729	22.9064	5
−1	7	2	336.971	22.1915	4
−1	7	2	338.677	22.1788	3
−1	7	2	324.734	22.2770	4
−1	7	2	332.378	22.2489	3
1	7	−2	402.971	22.4344	4
0	−7	−2	92.0932	7.30163	2
0	7	2	95.4675	6.37658	4
0	7	2	94.1666	6.27011	3
0	7	−2	94.1204	6.04874	4
0	7	−2	87.4764	6.01753	4
0	7	−2	89.0888	6.00615	3
−1	−7	−2	347.755	23.9462	2
−1	−7	−2	364.363	24.6326	5
−1	7	−2	387.835	22.4849	4
−1	7	−2	364.794	22.5783	3
−1	7	−2	379.215	22.4906	4
−2	−7	−2	290.840	18.6374	5
−2	−7	−2	288.303	18.2344	2
−2	7	−2	245.235	16.1450	3
−2	7	−2	245.595	16.1246	3
−3	−7	−2	217.510	15.9060	2
−3	−7	−2	213.121	16.0212	1
−3	−7	−2	219.201	16.1529	5

−3	7	−2	222.797	13.4119	3
−3	7	−2	223.862	13.4277	3
−4	−7	−2	162.835	12.4203	5
−4	−7	−2	159.463	12.6652	2
−4	−7	−2	156.328	12.4945	1
−4	7	−2	143.876	9.67372	3
−5	−7	−2	16.6567	2.58558	8
−5	−7	−2	18.8458	4.61626	2
−5	−7	−2	17.7598	4.47634	1
−5	7	−2	16.1886	1.17053	6
−5	7	−2	17.9997	2.74677	7
−5	7	−2	15.2995	1.75357	3
−5	7	−2	14.1933	1.09923	6
−6	−7	−2	111.496	8.89213	10
−6	−7	−2	107.204	11.4024	2
−6	−7	−2	109.451	10.0115	1
−6	−7	−2	112.967	8.12609	8
−6	7	−2	114.023	7.03240	9
−6	7	−2	114.019	6.91083	6
−6	7	−2	106.822	7.94504	7
−7	−7	−2	101.356	8.62894	10
−7	−7	−2	101.230	8.39991	8
−7	7	−2	120.949	7.19516	9
7	7	2	108.092	10.1871	13
−7	7	−2	125.687	7.04156	6
−8	−7	−2	1.91120	1.89768	10
−8	−7	−2	1.98714	2.16297	10
−8	−7	−2	2.43305	1.69562	8
−8	7	−2	0.27012	0.39681	6
8	7	2	5.24866	3.54337	13
−8	7	−2	0.79015	0.73331	9
−9	−7	−2	15.1173	2.09419	8
−9	7	−2	12.3488	1.14871	9
9	7	2	16.2675	3.31462	13
−9	7	−2	12.2021	1.11841	9
−9	−8	2	2.82781	1.72771	8
−9	−8	2	2.30785	1.93178	10
−9	8	2	3.02330	0.55297	6
−9	8	2	3.49380	0.68985	9
−9	8	2	3.13463	1.30176	7
9	8	−2	0.45862	3.46836	13
−8	−8	2	38.4403	4.61627	10
−8	8	2	28.6906	3.74524	7
−8	8	2	25.9094	2.06427	6
−8	8	2	28.7526	2.40181	9
−7	−8	2	7.80186	2.72992	10
−7	−8	2	8.70312	4.40774	1
−7	8	2	4.36440	0.65903	6
−7	8	2	2.94574	0.91110	9
−7	8	2	4.01144	1.93914	7

7	8	−2	11.6873	4.71944	13
−6	−8	2	131.909	11.4437	10
−6	−8	2	154.055	13.5636	1
−6	8	2	152.759	9.54054	9
−6	8	2	157.808	10.3774	7
−6	8	2	157.667	9.35898	6
−5	−8	2	58.9111	7.58398	1
−5	−8	2	68.9274	8.14450	5
−5	−8	2	62.2441	7.48759	2
−5	8	2	62.3119	4.64802	3
−4	−8	2	29.9408	4.45438	2
−4	−8	2	30.0721	5.35589	5
−4	−8	2	29.3766	5.26840	1
−4	8	2	29.3556	2.53619	3
−3	−8	2	211.676	14.8258	2
−3	−8	2	204.282	15.7902	1
3	−8	−2	210.998	15.2481	2
−3	8	2	216.044	13.0893	4
−3	8	2	210.271	13.1814	4
−3	8	2	205.084	13.4286	3
−3	8	2	215.685	13.3099	3
2	−8	−2	1520.27	94.6238	2
−2	−8	2	1555.08	94.3098	2
−2	8	2	1523.98	93.1916	4
−2	8	2	1516.76	93.3797	3
−2	8	2	1517.59	93.2927	4
−2	8	2	1576.44	93.2787	3
1	−8	−2	37.6446	4.33779	2
−1	−8	2	34.4323	3.53094	2
−1	8	2	34.1370	2.88019	4
−1	8	2	32.3579	2.84751	3
−1	8	2	31.6378	3.09591	4
1	8	−2	37.3385	3.04227	4
1	8	−2	39.1333	3.47246	5
0	−8	−2	805.380	53.6523	2
0	8	−2	920.055	52.5375	4
0	8	−2	889.628	52.5042	4
0	8	2	848.475	52.8068	4
0	8	−2	903.583	52.5495	3
0	8	2	798.088	52.7028	3
−1	8	−2	21.7414	1.80350	3
−2	8	−2	453.005	27.5325	3
−3	−8	−2	203.616	15.3185	5
−3	−8	−2	203.762	15.0648	2
−3	−8	−2	201.742	15.3062	1
−4	−8	−2	137.710	11.9257	5
−4	−8	−2	136.249	12.1623	1
−4	−8	−2	140.152	12.2415	2
−4	8	−2	151.560	8.90055	3
−5	−8	−2	150.225	12.9911	2

−5	−8	−2	148.227	9.94625	8
−5	−8	−2	147.905	12.7624	1
−5	8	−2	141.933	9.11487	3
−5	8	−2	138.922	8.74747	6
−5	8	−2	146.273	8.76329	6
−5	8	−2	139.375	8.82031	9
−5	8	−2	139.400	9.72788	7
−6	−8	−2	6.54743	2.40591	8
−6	−8	−2	6.23404	3.25473	1
−6	−8	−2	4.08057	2.51542	10
−6	8	−2	2.54908	1.78456	7
6	8	2	5.00744	3.90862	13
−6	8	−2	0.33582	3.23188	12
−6	8	−2	4.75004	0.68512	9
−6	8	−2	6.45410	0.64468	6
−6	8	−2	5.40465	0.63971	6
−7	−8	−2	125.657	9.26693	10
−7	−8	−2	122.991	8.67926	8
−7	8	−2	117.825	8.45879	7
−7	8	−2	116.321	7.30042	6
7	8	2	119.835	10.5860	13
−7	8	−2	111.659	7.28931	6
−7	8	−2	116.621	7.43196	9
−8	−8	−2	1.30355	1.66888	10
−8	−8	−2	2.80740	2.01958	10
−8	−8	−2	2.98323	1.82837	8
−8	8	−2	4.49407	0.83767	9
−8	8	−2	4.21291	0.49532	6
8	8	2	3.55210	3.62390	13
−8	8	−2	3.59110	0.49933	6
9	8	2	17.3877	3.36583	13
−9	−9	2	6.19855	1.95617	8
−9	−9	2	7.53854	2.27616	10
−9	9	2	9.07134	1.10415	9
−9	9	2	8.27292	0.74374	6
9	9	−2	3.39968	3.31033	13
−9	9	2	6.11985	1.83540	7
−8	−9	2	63.7988	6.46506	10
−8	9	2	67.1452	4.35789	6
−8	9	2	68.5275	4.55905	9
8	9	−2	63.8631	7.93123	13
−8	9	2	76.5428	5.85870	7
−7	−9	2	39.3816	6.75602	1
−7	9	2	29.3560	2.14255	6
7	9	−2	42.7545	6.34433	13
−7	9	2	28.2882	3.68587	7
−7	9	2	26.7244	2.29086	9
−6	−9	2	53.9153	7.71510	2
−6	−9	2	52.7930	8.64168	1
−6	9	2	65.2702	4.11022	6

−6	9	2	64.4836	5.40381	7
−6	9	2	71.8631	4.37332	9
−5	−9	2	101.090	9.83819	2
−5	−9	2	101.086	10.8119	5
−5	−9	2	110.508	10.7405	1
−5	9	2	107.089	6.60628	6
−5	9	2	110.414	7.26651	3
−4	−9	2	27.7534	4.74096	2
−4	−9	2	27.2963	5.32134	1
4	−9	−2	27.2569	4.76420	2
−4	9	2	29.7633	2.50247	3
3	−9	−2	39.0406	5.13700	2
−3	−9	2	31.9738	4.39879	2
−3	−9	2	29.8479	5.84926	1
−3	9	2	40.2296	3.20673	3
−3	9	2	40.2204	2.80028	4
−3	9	2	40.7885	3.05914	3
−3	9	2	39.1526	2.71423	4
−2	−9	2	678.457	42.5304	2
2	−9	−2	655.452	42.9356	2
−2	9	2	679.288	41.3911	4
−2	9	2	715.068	41.3268	4
−2	9	2	647.406	41.3821	3
1	−9	−2	363.056	24.9144	2
1	9	−2	415.832	23.7489	4
−1	9	2	351.561	23.6040	4
1	9	−2	403.450	24.0011	5
−1	9	2	367.072	23.5715	4
−1	9	2	371.799	23.5908	3
0	−9	−2	31.5352	4.53054	2
0	9	−2	41.6414	3.04145	3
0	9	−2	42.7017	3.07316	4
0	9	−2	40.5037	3.00856	4
0	9	2	28.4598	3.20134	4
−1	−9	−2	36.5151	5.29006	2
−1	9	−2	47.5518	3.21125	3
−1	9	−2	45.0943	3.04133	4
−1	9	−2	45.8320	3.04579	4
−2	−9	−2	17.5535	3.63375	2
−2	−9	−2	18.4681	3.74578	1
−2	9	−2	13.9723	1.26132	3
−3	−9	−2	90.1217	8.56823	2
−3	−9	−2	93.4338	9.27533	1
−3	−9	−2	96.2140	8.96444	5
−4	−9	−2	5.69255	3.32891	5
−4	−9	−2	4.79594	3.50152	1
−4	−9	−2	4.55725	2.85907	2
−5	−9	−2	20.5757	3.16431	8
−5	−9	−2	20.2571	5.26719	1
−5	−9	−2	21.4146	5.03419	2

−5	9	−2	26.9332	1.64546	6
−5	9	−2	25.0334	1.78745	3
−5	9	−2	24.7099	3.34667	7
−5	9	−2	25.4248	1.72440	9
−5	9	−2	25.1469	1.62713	6
−5	9	−2	25.9412	1.79749	9
−6	−9	−2	56.9969	4.92196	8
−6	−9	−2	54.7755	7.89851	1
−6	−9	−2	63.2004	5.92590	10
−6	9	−2	47.0656	3.18566	6
−6	9	−2	48.5018	3.30142	9
−6	9	−2	46.2074	4.52674	7
6	9	2	53.5905	8.45527	13
−6	9	−2	51.5883	3.20025	6
−6	9	−2	44.1326	5.30190	12
−7	−9	−2	20.9351	4.08662	10
−7	−9	−2	18.4522	2.88321	8
−7	9	−2	19.2580	1.59070	9
−7	9	−2	20.2982	1.38255	6
7	9	2	14.3335	4.43276	13
−7	9	−2	20.7865	3.05014	7
−7	9	−2	19.9676	1.41068	6
−8	−9	−2	36.8982	3.99035	10
−8	−9	−2	36.9213	3.98665	8
−8	9	−2	38.1308	2.44339	9
−8	9	−2	31.6383	2.42054	9
8	9	2	30.5983	5.06061	13
9	9	2	5.83518	2.68983	13
−9	−10	2	5.73551	2.13490	10
−9	−10	2	4.03740	1.76005	8
−9	10	2	4.00785	0.43723	6
−9	10	2	3.65142	0.66046	9
−9	10	2	3.29517	0.43855	6
−9	10	2	2.61731	1.59429	7
9	10	−2	5.01044	3.01848	13
−8	−10	2	4.60544	2.27196	10
−8	10	2	10.0062	1.11235	9
8	10	−2	1.26457	3.84764	13
−8	10	2	12.9464	2.42498	7
−8	10	2	9.42582	0.97031	6
−7	−10	2	34.7445	5.15394	10
−7	−10	2	37.0065	7.29852	1
7	10	−2	30.6764	5.29645	13
−7	10	2	52.2657	4.55638	7
−7	10	2	45.5794	2.87776	6
−7	10	2	48.4951	3.13438	9
6	−10	−2	15.5022	4.53652	2
−6	−10	2	27.1094	6.37990	1
−6	−10	2	23.4930	5.18416	2
−6	10	2	11.9920	1.16606	6

−6	10	2	14.9301	2.40641	7
−6	10	2	12.8282	1.49126	9
6	10	−2	21.1285	5.44083	13
−5	−10	2	120.562	11.3353	1
5	−10	−2	106.971	10.2621	2
−5	−10	2	118.190	10.2790	2
−5	10	2	96.4768	7.55512	7
−5	10	2	102.399	7.24926	3
−5	10	2	106.194	6.61506	6
−4	−10	2	24.4386	4.77153	2
−4	−10	2	24.3961	5.42107	1
4	−10	−2	35.7581	5.34709	2
−4	10	2	33.5811	2.67052	3
3	−10	−2	44.1170	5.90101	2
−3	−10	2	35.3241	4.70504	2
−3	10	2	42.8481	3.29943	3
−3	10	2	43.6733	2.85929	4
−3	10	2	40.9797	2.89675	4
2	−10	−2	52.5220	6.25682	2
−2	10	2	48.9096	3.82503	3
−2	10	2	52.7137	3.79377	4
−2	10	2	53.4957	3.95301	4
1	−10	−2	869.449	53.6725	2
1	10	−2	802.467	52.2772	4
−1	10	2	895.530	52.2437	4
−1	10	2	890.628	52.2839	3
1	10	−2	802.014	52.2547	5
0	−10	−2	145.073	11.6389	2
0	10	−2	161.942	10.1204	4
0	10	−2	161.277	10.0626	4
0	10	2	152.817	10.4156	4
−1	−10	−2	292.715	18.0011	2
−1	10	−2	232.900	15.8052	3
−1	10	−2	252.104	15.6231	4
−1	10	−2	246.192	15.5957	4
−2	−10	−2	20.1482	4.36624	1
−2	−10	−2	18.5252	3.74833	2
−2	10	−2	29.0915	1.96799	3
−3	−10	−2	210.407	16.3599	1
−3	−10	−2	213.770	16.0300	2
−3	10	−2	211.900	12.9260	3
−4	−10	−2	6.77834	3.18255	5
−4	−10	−2	6.16670	3.77878	2
−4	−10	−2	7.91493	4.04193	1
−5	−10	−2	14.5319	4.38555	2
−5	−10	−2	16.6523	3.32545	10
−5	−10	−2	13.1055	4.34109	1
−5	10	−2	13.8555	0.93372	6
−5	10	−2	12.5204	1.01753	9
−5	10	−2	12.8687	1.09763	9

−5	10	−2	13.3561	0.91630	6
−5	10	−2	14.3282	2.30157	7
−6	−10	−2	49.7968	4.93875	8
−6	−10	−2	56.1730	8.29126	1
−6	−10	−2	50.7303	5.70753	10
−6	10	−2	49.9243	3.19010	9
−6	10	−2	48.8102	4.56557	7
−6	10	−2	48.5005	3.07684	6
−6	10	−2	50.1903	3.07981	6
−6	10	−2	43.3705	5.27269	12
6	10	2	48.2183	6.91290	13
−6	10	−2	48.3867	3.24116	9
−7	−10	−2	25.2962	3.76444	8
−7	−10	−2	25.8739	5.40294	1
−7	−10	−2	25.4803	4.57310	10
−7	10	−2	25.0251	1.88942	9
−7	10	−2	26.5580	1.64606	6
−7	10	−2	25.4372	1.63968	6
−7	10	−2	21.9387	1.86758	9
−7	10	−2	31.5937	3.63003	7
−8	−10	−2	2.78452	1.42427	8
−8	−10	−2	3.63074	1.97407	10
−8	−10	−2	4.68183	2.32713	10
8	10	2	4.20021	3.41464	13
9	10	2	7.11209	2.46856	13
−9	−11	2	26.4760	3.31317	10
−9	−11	2	26.4901	3.70728	8
−9	11	2	20.7667	2.79070	7
−9	11	2	19.8863	1.50791	9
9	11	−2	24.2167	4.02021	13
−9	11	2	19.2777	1.55863	9
−8	−11	2	2.01135	2.62646	10
−8	11	2	8.14535	0.80711	6
−8	11	2	8.90557	2.22911	7
8	11	−2	2.52362	4.09483	13
−8	11	2	7.95290	1.02640	9
−7	−11	2	7.03205	2.93071	10
−7	−11	2	5.92268	4.19827	1
7	11	−2	3.92727	4.40276	13
−7	11	2	7.35149	0.78412	6
−7	11	2	7.68216	0.99421	9
−7	11	2	7.32623	2.26109	7
6	−11	−2	24.0943	7.46196	2
−6	11	2	21.6137	1.87741	9
−6	11	2	21.3518	1.57070	6
−6	11	2	24.9265	3.24186	7
6	11	−2	10.0732	4.50586	13
−5	−11	2	169.187	14.9583	1
5	−11	−2	158.531	13.5654	2
−5	−11	2	164.141	13.6423	2

-5	11	2	155.968	9.97363	9
-5	11	2	158.938	9.76430	6
-5	11	2	152.668	10.7306	7
-5	11	2	158.873	10.3951	3
-4	-11	2	15.2186	3.77113	2
-4	-11	2	13.5428	4.30673	1
-3	-11	2	200.495	14.8774	2
3	-11	-2	210.287	15.3765	2
-3	11	2	205.302	13.1257	3
-3	11	2	202.967	12.5391	4
2	-11	-2	391.726	26.1592	2
-2	11	2	388.371	23.9334	4
-2	11	2	384.058	24.1158	3
-2	11	2	382.082	23.9838	4
1	-11	-2	39.9533	5.23236	2
1	11	-2	47.8994	3.81019	4
-1	11	2	37.5409	3.47868	4
-1	11	2	39.2651	3.41562	3
0	11	-2	97.7725	6.19549	4
0	11	2	70.2326	6.31087	4
0	11	-2	96.3581	6.12133	4
-1	-11	-2	381.128	24.4151	2
-1	11	-2	356.324	21.9749	4
-1	11	-2	357.546	22.0095	4
-1	11	-2	344.836	22.2594	3
-2	-11	-2	10.0272	3.47146	2
-2	11	-2	14.7795	1.37941	3
-3	11	-2	27.5415	1.94891	3
-3	11	-2	28.8904	1.93091	3
-4	-11	-2	27.2433	5.59586	1
-4	-11	-2	23.4499	4.98950	2
-4	11	-2	27.3024	3.05396	7
-5	-11	-2	8.09606	3.98424	2
-5	-11	-2	9.93699	4.02007	1
-5	-11	-2	8.71743	3.49555	10
-5	11	-2	9.10368	2.02489	7
-5	11	-2	7.37335	2.69282	12
-5	11	-2	7.71536	0.75979	9
-6	-11	-2	8.99222	3.16290	10
-6	-11	-2	10.3796	4.53257	1
-6	11	-2	19.0811	3.60584	12
6	11	2	8.84017	4.50494	13
-6	11	-2	15.2826	2.53448	7
-6	11	-2	15.3960	1.19972	9
-6	11	-2	16.6705	1.18254	9
-7	-11	-2	11.1542	3.25878	10
-7	-11	-2	11.0370	2.52979	8
-7	11	-2	2.67685	2.58263	12
-7	11	-2	7.86443	2.14498	7
7	11	2	10.7466	3.79351	13

−8	−11	−2	0.06412	2.15336	10
−8	−11	−2	1.27840	1.74480	8
−8	−11	−2	0.51159	1.81397	10
8	11	2	1.92043	3.08128	13
−9	−12	2	1.59943	1.90106	10
−8	−12	2	1.30608	1.96889	10
−8	12	2	0.89109	0.55027	9
−8	12	2	0.47288	0.40384	6
−8	12	2	0.60258	1.77239	7
8	12	−2	0.57291	3.12899	13
−8	12	2	0.34244	0.33452	6
−7	−12	2	5.02044	4.17573	1
−7	−12	2	5.26739	2.89238	10
−7	12	2	3.27822	0.55834	6
7	12	−2	4.07824	3.44693	13
−7	12	2	3.66111	0.74153	9
−7	12	2	1.99808	1.96660	7
−6	−12	2	69.6591	9.25083	1
−6	−12	2	62.1526	6.37382	10
−6	12	2	53.8965	3.92573	9
−6	12	2	55.8934	5.15968	7
−6	12	2	55.8412	3.72356	6
6	12	−2	66.7709	7.83917	13
5	−12	−2	50.7835	7.15988	2
−5	−12	2	48.8453	8.67020	1
−5	12	2	50.4299	4.52336	7
−5	12	2	48.3409	3.51847	9
−5	12	2	53.0561	3.27235	6
4	−12	−2	83.7933	8.45776	2
−4	−12	2	68.8921	8.91182	1
−4	12	2	80.5009	5.55640	3
−4	12	2	77.5378	4.89743	6
3	−12	−2	188.895	14.5092	2
−3	12	2	184.524	12.1003	3
2	−12	−2	252.408	18.2936	2
−2	12	2	254.599	15.9255	4
−2	12	2	243.056	16.0660	3
−2	12	2	258.159	15.8610	4
1	−12	−2	24.8214	4.62075	2
−1	12	2	24.8012	2.71965	4
1	12	−2	28.0388	2.83348	4
−1	12	2	23.4946	2.72958	3
0	−12	−2	436.986	28.8835	2
0	12	−2	415.977	26.8317	4
0	12	2	443.737	27.3801	4
−1	12	−2	20.1726	1.69841	4
−1	12	−2	20.3897	1.66288	4
−2	−12	−2	88.5220	8.04435	2
−2	12	−2	74.0879	5.24664	3
−3	−12	−2	25.8895	5.12084	2

−3	−12	−2	26.4984	5.48827	1
−3	12	−2	30.0818	2.08648	3
−3	12	−2	28.3724	2.04885	3
−3	12	−2	30.0678	1.83797	6
−4	−12	−2	111.339	10.7323	2
−4	−12	−2	111.182	11.5559	1
−4	12	−2	102.210	7.64763	7
−5	−12	−2	7.17327	4.84163	1
−5	−12	−2	5.96223	3.88384	2
−5	12	−2	17.3632	3.73620	12
−5	12	−2	6.86241	2.12050	7
−6	−12	−2	28.9192	4.78967	10
−6	−12	−2	25.7408	5.84645	1
6	12	2	23.9059	4.98620	13
−6	12	−2	21.7384	3.48150	7
−6	12	−2	24.4269	3.73290	12
−7	−12	−2	82.6309	6.69304	8
−7	−12	−2	82.7186	7.46962	10
−7	12	−2	77.7057	6.98490	12
7	12	2	84.0742	9.19843	13
−8	−12	−2	29.4992	3.76204	10
−8	−12	−2	29.0776	3.39810	8
−8	−12	−2	30.0864	3.61493	10
8	12	2	24.7396	4.03637	13
−9	−13	2	12.3860	2.34995	10
−8	−13	2	10.7839	3.12169	10
−8	13	2	5.54232	0.51709	6
−8	13	2	4.51308	0.51681	6
−8	13	2	6.69573	0.84392	9
−8	13	2	5.53458	0.78404	9
−8	13	2	3.64593	1.91174	7
−7	−13	2	25.4517	4.40130	10
−7	−13	2	26.3313	5.90655	1
−7	13	2	28.0231	2.10549	9
−7	13	2	28.2075	1.89612	6
−7	13	2	29.0190	1.95440	6
−7	13	2	28.8961	3.86019	7
−6	−13	2	1.77760	2.67159	10
−6	−13	2	2.96875	4.38917	1
−6	13	2	3.07444	0.55661	6
−6	13	2	2.39292	1.98416	7
−6	13	2	3.52396	0.78026	9
5	−13	−2	9.09157	3.68764	2
−5	−13	2	14.0386	5.65515	1
−5	13	2	8.28562	1.18040	9
−5	13	2	10.7519	0.97259	6
−5	13	2	12.0719	2.51898	7
4	−13	−2	6.06126	3.23913	2
−4	13	2	5.91408	1.78471	7
−4	13	2	6.44880	0.72802	6

−4	13	2	6.97745	1.29858	3
3	−13	−2	1.29561	2.63108	2
−3	13	2	1.76329	1.02815	3
2	−13	−2	365.361	24.9846	2
−2	13	2	359.149	22.8106	3
−2	13	2	364.918	22.4753	4
−2	13	2	357.296	22.5227	4
1	−13	−2	304.754	22.3234	2
−1	13	2	310.622	20.2666	4
1	13	−2	343.369	20.5623	4
0	−13	−2	83.8801	8.30627	2
0	13	−2	83.8443	5.96941	4
−1	−13	−2	162.324	13.1831	2
−1	13	−2	163.621	10.2895	4
−1	13	−2	168.576	10.2862	4
−2	−13	−2	222.059	17.3765	2
−2	13	−2	231.074	14.5799	3
−2	13	−2	239.775	14.6530	3
−3	−13	−2	120.162	10.6439	2
−3	−13	−2	122.893	11.7515	1
−3	13	−2	105.066	6.58558	6
−3	13	−2	107.686	6.87934	3
−3	13	−2	98.9920	6.81100	3
−4	−13	−2	17.7669	5.19135	1
−4	−13	−2	22.2597	5.16251	2
−4	13	−2	22.0787	2.98152	7
−4	13	−2	20.6325	1.43617	9
−4	13	−2	22.0050	1.32884	6
−4	13	−2	20.7505	1.33669	6
−4	13	−2	21.5857	1.38251	9
−5	−13	−2	11.1411	4.58546	1
−5	−13	−2	9.10740	3.31185	10
5	13	2	13.4778	4.32021	13
−5	13	−2	15.5202	3.18891	12
−6	−13	−2	304.275	23.4005	1
−6	−13	−2	310.288	20.8799	10
6	13	2	293.781	20.6928	13
−6	13	−2	313.904	20.2113	12
−7	−13	−2	6.79410	3.01223	10
7	13	2	5.88649	3.26710	13
−7	13	−2	0.10602	2.45496	12
−8	−13	−2	5.49393	2.07524	10
−8	−13	−2	5.12916	2.41693	10
8	13	2	6.52515	2.66913	13
−8	−14	2	1.45014	1.82609	10
−8	14	2	3.01966	0.39652	9
−8	14	2	1.78950	1.29795	7
−7	−14	2	72.7809	6.29657	10
−7	14	2	61.4833	3.99206	9
−7	14	2	56.8655	3.81712	6

−7	14	2	59.8098	3.81813	6
−7	14	2	58.6848	5.37357	7
−6	−14	2	97.2328	11.6748	1
−6	−14	2	98.1231	8.58389	10
−6	14	2	90.3049	6.01968	9
−6	14	2	92.6951	7.22412	7
−6	14	2	93.4209	5.83820	6
5	−14	−2	9.35800	4.39916	2
−5	−14	2	6.21538	4.30782	1
−5	14	2	4.93238	0.99107	9
5	14	−2	0.84116	3.96449	10
−5	14	2	4.42724	0.64467	6
−5	14	2	3.34850	1.92296	7
4	−14	−2	19.8953	4.61168	2
−4	14	2	20.9542	2.44116	3
−4	14	2	24.0110	2.95980	7
−4	14	2	23.2081	1.64695	6
−4	14	2	21.5595	2.10172	9
3	−14	−2	35.1788	6.41816	2
−3	14	2	33.7442	2.36352	6
−3	14	2	34.8077	3.21590	3
2	−14	−2	21.6836	4.41840	2
−2	14	2	21.1813	2.58913	3
−2	14	2	22.1114	2.31685	4
−2	14	2	22.4256	2.28816	4
1	−14	−2	99.9169	9.51415	2
−1	14	2	92.7999	6.78508	4
0	−14	−2	0.14525	3.12706	2
0	14	−2	3.80167	1.24584	3
0	14	−2	4.11571	1.29776	4
−1	−14	−2	9.68454	3.23852	2
−1	14	−2	11.1991	1.71280	3
−1	14	−2	9.80860	1.07060	4
−1	14	−2	10.7591	1.04419	4
−2	−14	−2	28.1651	5.73419	2
−2	14	−2	32.6662	2.95506	3
−2	14	−2	38.2450	2.31723	6
−3	14	−2	61.3398	4.09361	9
−3	14	−2	67.4896	3.94650	6
−3	14	−2	60.3059	4.23369	3
−3	14	−2	65.1605	4.31847	3
−4	−14	−2	0.18655	4.19587	1
−4	−14	−2	0.76689	3.04423	2
−4	14	−2	5.69026	0.47430	6
−4	14	−2	5.25824	0.44980	6
−4	14	−2	14.9977	3.37119	12
−4	14	−2	5.18598	0.70220	9
−4	14	−2	5.95790	0.58820	9
−5	−14	−2	55.3077	6.36234	10
−5	−14	−2	54.9377	8.50865	1

−5	14	−2	48.7406	5.78343	12
−6	−14	−2	6.88850	5.19474	1
−6	−14	−2	5.58068	3.29142	10
−6	14	−2	2.75848	3.07723	12
−7	−14	−2	30.8665	4.78672	10
−8	−14	−2	5.59616	1.62362	10
−8	−15	2	5.87095	2.13134	10
−7	−15	2	46.6124	5.70157	10
−7	15	2	42.1110	4.35175	7
−7	15	2	38.6582	2.55059	6
−7	15	2	38.5419	2.55766	6
−7	15	2	42.3303	2.72585	9
−6	−15	2	34.1314	5.12813	10
−6	−15	2	39.2127	8.04962	1
−6	15	2	23.2851	1.90385	9
−6	15	2	23.1347	3.55107	7
−6	15	2	20.0141	1.61892	6
−6	15	2	22.0054	1.64824	6
−5	−15	2	8.25523	5.03271	1
5	15	−2	8.43983	3.06591	10
−5	15	2	11.0655	1.08350	6
−5	15	2	8.68276	2.15437	7
−5	15	2	10.3840	1.25829	9
4	−15	−2	8.60551	4.36866	2
−4	15	2	14.6304	1.18589	6
4	15	−2	10.4637	4.22600	10
−4	15	2	15.9473	2.40416	7
3	−15	−2	35.4729	6.60965	2
−3	15	2	30.6212	3.33162	7
−3	15	2	31.9712	3.17784	3
−3	15	2	34.3544	2.31581	6
2	−15	−2	24.9999	5.25358	2
−2	15	2	24.4707	2.35615	4
−2	15	2	26.4695	2.40538	4
−2	15	2	23.9810	2.87709	3
1	−15	−2	15.2346	4.48047	2
−1	15	2	14.7317	2.28873	4
0	−15	−2	24.6887	4.79886	2
0	15	−2	24.4808	2.67009	4
−1	−15	−2	9.34912	3.85920	2
−1	15	−2	15.8865	2.08619	3
−2	−15	−2	498.484	33.4763	2
−2	15	−2	487.104	29.6121	6
−2	15	−2	479.587	30.2724	3
−3	−15	−2	15.8623	4.31884	2
−3	15	−2	6.13974	0.64256	6
−3	15	−2	4.71142	0.84511	3
−3	15	−2	6.24095	0.84955	9
−4	15	−2	27.7162	1.70531	6
−4	15	−2	27.3932	1.92167	9

−4	15	−2	24.6200	1.78626	9
−4	15	−2	26.8198	1.68307	6
−4	15	−2	25.2644	4.46020	12
−5	−15	−2	50.0509	6.17188	10
−5	−15	−2	46.0061	8.34359	1
−5	15	−2	51.6181	6.36916	12
−6	−15	−2	8.49035	3.35780	10
−6	15	−2	18.1113	3.56725	12
−7	−15	−2	9.46678	3.14671	10
−8	−15	−2	7.12081	1.97877	10
−8	−16	2	25.5588	3.97086	10
−7	−16	2	3.89746	3.11311	10
−7	16	2	2.94459	0.49885	9
−7	16	2	1.08827	1.96368	7
−7	16	2	2.74755	0.47286	9
−6	−16	2	90.0003	11.5555	1
−6	−16	2	92.9367	9.75200	10
−6	16	2	98.4002	5.99460	6
−6	16	2	101.737	7.49514	7
−6	16	2	97.3716	5.99606	6
6	16	−2	78.8889	12.7537	10
−6	16	2	100.166	6.19609	9
−5	−16	2	15.5585	5.62417	1
−5	16	2	7.16238	0.79399	6
−5	16	2	6.60713	1.09881	9
−5	16	2	7.12964	2.12787	7
5	16	−2	14.7764	4.20570	10
−4	−16	2	4.87204	2.97223	13
4	16	−2	3.63625	3.64132	10
−4	16	2	10.7670	2.19420	7
−4	16	2	9.32651	1.39114	9
−4	16	2	9.54549	0.93873	6
3	−16	−2	85.2995	9.57682	2
3	16	−2	91.9374	8.19535	10
−3	16	2	74.0923	6.13152	3
−3	16	2	80.6307	5.59181	9
2	−16	−2	88.3934	9.26543	2
−2	16	2	87.1667	5.93649	4
1	−16	−2	28.0655	5.78200	2
−1	16	2	30.6963	3.20268	4
0	−16	−2	36.0470	5.87062	2
0	16	−2	35.9027	3.32334	4
−1	−16	−2	136.885	12.2636	2
−1	16	−2	135.610	9.41362	3
−2	−16	−2	86.5089	9.82675	2
−2	16	−2	108.503	6.92616	9
−2	16	−2	108.761	7.31822	3
−2	16	−2	110.150	6.56419	6
−3	−16	−2	2.80544	3.12046	2
−3	16	−2	2.05662	0.82214	9

3	16	2	3.01393	3.55465	10
−3	16	−2	4.35491	3.26677	12
−3	16	−2	1.95491	0.48697	6
−3	16	−2	2.49406	0.43831	6
−4	−16	−2	9.64683	4.81750	1
−4	16	−2	9.41970	0.76435	6
−4	16	−2	10.5803	1.08084	9
−4	16	−2	10.1019	0.89013	9
−4	16	−2	9.73082	3.07773	12
4	16	2	7.23063	3.98488	10
−5	−16	−2	39.3266	7.81263	1
−5	−16	−2	34.1332	5.67956	10
−5	16	−2	48.1092	6.18433	12
−6	−16	−2	22.1231	4.34536	10
−6	16	−2	20.3210	3.82461	12
−7	−16	−2	12.6296	3.36216	10
−7	−16	−2	12.6236	3.07552	10
−8	−17	2	7.48010	2.50207	10
−7	−17	2	−1.4498	2.59583	10
6	17	−2	10.7051	4.48627	10
−6	17	2	13.9730	1.10429	6
−6	17	2	15.7633	1.12514	6
−6	17	2	15.4957	1.46435	9
−5	17	2	33.9327	2.70687	9
−5	17	2	34.3491	2.33169	6
5	17	−2	35.1149	6.13092	10
−4	−17	2	70.1782	6.69407	13
4	17	−2	75.5982	7.83128	10
−4	17	2	69.1883	4.54591	6
−4	17	2	70.1970	4.89926	9
−3	−17	2	42.1857	6.40875	13
−3	17	2	31.1589	3.20137	9
3	17	−2	46.3881	5.90093	10
2	−17	−2	28.4842	6.01970	2
2	17	−2	27.4476	5.39693	10
1	−17	−2	3.44614	3.83944	2
0	−17	−2	14.4488	4.05193	2
−1	−17	−2	69.1496	8.35940	2
−1	17	−2	60.4122	5.18970	3
−2	−17	−2	17.3081	4.95321	2
−2	17	−2	17.1389	1.40614	6
−2	17	−2	15.1018	1.78567	9
2	17	2	27.8564	5.38599	10
−3	−17	−2	91.0774	7.07107	13
−3	17	−2	83.2162	5.28282	6
−3	17	−2	79.1534	5.52564	9
3	17	2	102.592	9.01746	10
−3	17	−2	76.8109	7.34097	12
−3	17	−2	84.1277	5.34808	6
−4	−17	−2	9.43976	5.00872	1

4	17	2	11.4079	4.11991	10
−4	17	−2	10.0650	0.78338	6
−4	17	−2	9.73025	0.94284	9
−4	17	−2	10.7023	0.75863	6
−4	17	−2	9.51898	1.17234	9
−4	17	−2	9.35916	3.12115	12
−5	−17	−2	0.39245	3.90004	10
−5	−17	−2	7.42169	4.51738	1
−5	17	−2	2.41607	3.23064	12
5	17	2	3.76135	4.47101	10
−6	−17	−2	27.8870	4.90105	10
−7	−17	−2	1.16855	2.54996	10
−7	−17	−2	0.43567	2.14954	10
−7	−18	2	11.3044	3.16782	10
−6	18	2	7.99266	1.03597	9
−6	18	2	9.22480	0.71914	6
6	18	−2	5.36381	4.05206	10
−6	18	2	7.92478	0.73536	6
5	18	−2	1.30483	3.73297	10
−5	18	2	0.35112	0.79409	9
−5	18	2	1.07590	0.48821	6
−4	−18	2	83.3232	7.11719	13
4	18	−2	79.2007	8.39291	10
−4	18	2	88.1843	5.86161	9
−4	18	2	86.8132	5.40788	6
−3	−18	2	15.3821	3.52481	13
3	18	−2	20.0204	4.24569	10
−3	18	2	17.4436	2.11258	9
−2	−18	2	16.4963	4.29691	13
−2	18	2	15.2225	1.92966	11
2	18	−2	18.0772	3.63326	10
−1	18	2	16.3017	3.46868	10
0	−18	−2	68.2610	8.38441	2
0	18	−2	53.2815	5.93212	11
0	18	2	62.4446	6.23872	10
−1	−18	−2	28.9339	5.97785	2
−1	18	−2	41.9351	3.03103	11
−1	18	−2	44.1955	3.15957	11
−2	−18	−2	7.97165	3.79738	2
−2	−18	−2	14.9308	3.41637	13
−2	18	−2	7.81745	0.80421	6
−2	18	−2	5.19775	1.34157	9
2	18	2	3.70013	3.48615	10
−2	18	−2	−3.5607	3.53622	12
−3	−18	−2	2.01375	2.18924	13
−3	18	−2	1.31484	0.55630	6
−3	18	−2	0.78337	2.67903	12
−3	18	−2	2.18058	0.96495	9
−3	18	−2	1.79095	0.42181	6
3	18	2	5.60675	3.52847	10

4	18	2	20.0915	4.76604	10
−4	18	−2	16.3125	3.50412	12
−4	18	−2	16.0186	1.08674	6
−4	18	−2	14.6675	1.26596	9
−4	18	−2	16.5508	1.06768	6
−4	18	−2	14.6466	1.46385	9
5	18	2	21.6900	4.89566	10
−6	−18	−2	27.5760	4.64456	10
−7	−18	−2	36.9734	3.92336	10
−7	−18	−2	38.7904	3.81226	10
−7	−19	2	10.1263	2.86298	10
7	19	−2	11.9930	3.64637	10
6	19	−2	7.10157	4.35741	10
−6	19	2	17.7345	1.37892	9
−6	19	2	16.3391	1.26079	9
−5	19	2	1.59580	0.83600	9
5	19	−2	0.52612	3.78492	10
−5	19	2	1.51041	0.51911	6
−4	−19	2	11.2703	3.25840	13
−4	19	2	10.8419	1.07644	6
4	19	−2	11.3035	3.98973	10
−4	19	2	11.5687	1.49545	9
−3	−19	2	138.432	10.7174	13
3	19	−2	141.978	11.4152	10
−2	−19	2	61.2966	5.82472	13
−2	19	2	44.8161	3.95519	11
−1	−19	2	24.4376	4.02004	13
1	19	−2	30.3601	4.96116	10
0	−19	−2	27.4191	4.63706	13
0	19	2	43.1931	5.08549	10
−1	−19	−2	17.8371	3.57883	13
−1	19	−2	11.8624	1.55241	11
1	19	2	18.9193	3.69560	10
−1	19	−2	10.9315	1.52941	11
−2	−19	−2	24.8237	3.78514	13
2	19	2	19.1917	4.07426	10
−2	19	−2	20.7821	1.67103	6
−2	19	−2	19.9153	2.16184	9
−3	−19	−2	43.9025	5.60800	13
3	19	2	45.5559	6.66576	10
−3	19	−2	46.5600	3.04700	6
−3	19	−2	50.1915	3.49554	9
−4	19	−2	0.19317	0.46479	9
4	19	2	11.0761	4.32384	10
5	19	2	6.00945	2.96075	10
−6	−19	−2	10.0825	2.44700	10
−6	−19	−2	9.22920	2.67613	10
7	20	−2	27.3217	4.48076	10
6	20	−2	−1.8815	3.35041	10
−6	20	2	2.27020	0.34157	9

5	20	-2	-3.2086	3.40082	10
-5	20	2	1.08804	0.40376	6
-5	20	2	1.57644	0.40746	6
-5	20	2	1.40184	0.77889	9
-4	-20	2	11.1492	3.48252	13
-4	20	2	4.34484	0.73146	6
-4	20	2	2.85234	1.28544	9
4	20	-2	0.22730	3.71019	10
-3	-20	2	3.93085	2.94763	13
3	20	-2	4.75548	3.26685	10
-2	-20	2	277.018	18.7957	13
-2	20	2	288.785	17.2204	11
2	20	-2	265.008	19.0464	10
-2	20	2	282.156	17.2741	11
-1	-20	2	109.837	8.96052	13
-1	20	2	125.596	8.07664	11
1	20	-2	112.528	8.91237	10
0	-20	-2	48.1165	5.56799	13
0	20	2	41.2029	5.36944	10
0	20	-2	38.0737	3.92395	11
1	20	2	7.57528	3.00514	10
-2	-20	-2	-0.7491	2.13615	13
-2	20	-2	1.12157	1.24184	9
-2	20	-2	2.97524	0.64844	6
2	20	2	1.08773	2.92534	10
-3	-20	-2	125.724	9.64326	13
-3	20	-2	126.737	7.90883	6
3	20	2	129.222	10.9329	10
-3	20	-2	130.550	8.25002	9
-4	20	-2	19.9684	1.50975	9
-4	20	-2	21.4109	1.63605	9
4	20	2	20.3009	4.73263	10
5	20	2	-4.8423	3.52233	10
-6	-20	-2	0.27901	1.79243	10
-6	-20	-2	0.89387	1.55666	10
6	20	2	1.61909	2.17670	10
6	21	-2	7.62251	2.72618	10
-5	21	2	4.91690	0.55249	6
-5	21	2	4.79332	0.53124	6
5	21	-2	7.85426	4.01429	10
-5	21	2	4.21085	0.89126	9
4	21	-2	3.63974	3.32859	10
-4	21	2	3.24141	0.63132	6
-4	21	2	3.23405	1.14694	9
-3	-21	2	12.7616	3.48012	13
3	21	-2	12.7148	3.83085	10
-2	21	2	74.2815	4.87041	11
-2	21	2	78.2462	4.86507	11
-1	-21	2	-0.1640	2.09575	13
1	-21	-2	3.91271	2.56520	13

−1	21	2	5.49202	1.49590	11
1	21	−2	0.59859	2.23570	10
0	−21	−2	27.0703	4.86523	13
0	21	2	28.6298	4.69582	10
−1	−21	−2	424.936	27.4377	13
1	21	2	430.082	28.0408	10
−2	−21	−2	80.0841	6.94135	13
2	21	2	73.7913	7.70311	10
−2	21	−2	77.8764	4.94227	6
−2	21	−2	76.7684	5.43378	9
−3	−21	−2	78.6468	7.04624	13
−3	21	−2	76.7798	4.92257	6
3	21	2	82.0961	8.22258	10
−3	21	−2	77.9020	5.30861	9
4	21	2	9.32496	3.01341	10
−4	21	−2	3.87542	0.56478	9
−4	21	−2	3.80559	0.52015	9
5	21	2	9.55883	3.83397	10
6	21	2	4.09590	2.11491	10
6	22	−2	22.0170	3.42895	10
−5	22	2	35.6796	2.47562	9
−5	22	2	33.6177	2.18316	6
−5	22	2	33.0287	2.18250	6
5	22	−2	37.7679	4.59706	10
−4	−22	2	4.05566	2.40253	13
4	22	−2	3.77231	3.23396	10
−4	22	2	3.01836	0.94145	9
−4	22	2	3.19808	0.54844	6
−3	−22	2	3.56158	2.51426	13
3	22	−2	2.75556	2.95952	10
−2	−22	2	35.8530	6.17804	13
1	−22	−2	58.6825	6.15599	13
−1	22	2	59.2091	4.48635	11
0	−22	−2	12.1198	3.12203	13
0	22	2	11.0907	2.91163	10
0	22	−2	17.2538	2.11142	11
−1	−22	−2	21.0675	3.76182	13
1	22	2	17.8913	3.54675	10
−2	−22	−2	38.1516	5.12557	13
−2	22	−2	31.0263	2.29650	6
−2	22	−2	31.0504	2.37842	6
2	22	2	35.3076	5.59946	10
−2	22	−2	34.5834	3.22571	9
−3	−22	−2	12.8533	3.03552	13
−3	22	−2	15.7263	1.22754	6
−3	22	−2	16.5363	1.79098	9
3	22	2	11.9432	3.11154	10
−3	22	−2	15.3805	1.26122	6
4	22	2	0.62397	3.28629	10
5	22	2	1.72079	3.04554	10

-5	23	2	4.46563	0.56765	9
5	23	-2	-0.8343	2.62763	10
-5	23	2	5.72493	0.64793	9
-4	-23	2	46.1557	5.37186	13
-4	23	2	42.1040	2.88680	6
-4	23	2	43.1838	3.22720	9
4	23	-2	47.7071	5.54652	10
-3	-23	2	30.2266	4.76855	13
3	23	-2	29.7320	4.69486	10
-2	-23	2	31.1229	4.82019	13
2	23	-2	28.0071	4.71714	10
1	-23	-2	9.91444	2.59557	13
-1	-23	2	12.9837	2.81279	13
-1	23	2	10.6235	1.44985	11
1	23	-2	12.4128	2.24000	11
-1	23	2	9.75209	1.40698	11
0	-23	-2	88.1238	8.25215	13
0	-23	2	90.0507	7.65383	13
0	23	-2	104.005	6.20210	11
0	23	-2	99.9686	6.14992	11
0	23	2	91.1856	7.07717	11
-1	-23	-2	31.7854	4.38543	13
1	23	2	32.0872	4.57765	10
-2	-23	-2	7.36150	2.73863	13
-2	23	-2	4.45184	0.66582	6
2	23	2	9.41932	3.17826	10
-2	23	-2	4.69953	1.27013	9
-3	-23	-2	24.1628	3.75271	13
-3	23	-2	27.6524	2.27123	9
3	23	2	20.6506	4.21420	10
-3	23	-2	27.4183	1.88914	6
-3	23	-2	29.4647	1.84938	6
4	23	2	4.75440	2.27850	10
5	23	2	3.96707	2.74480	10
5	24	-2	3.39379	1.74002	10
-4	-24	2	4.54115	1.91770	13
-4	24	2	1.67689	0.43082	6
-4	24	2	1.83978	0.69949	9
4	24	-2	4.45190	2.44522	10
-3	-24	2	4.40964	2.33047	13
3	24	-2	4.17617	2.29199	10
-3	24	2	4.04597	1.20085	9
-2	-24	2	136.420	9.98946	13
2	24	-2	126.633	9.43805	10
2	24	-2	136.537	9.21481	11
-1	-24	2	4.46381	2.31520	13
1	24	-2	3.70404	1.39429	11
-1	24	2	2.22258	0.31486	11
0	-24	-2	16.8538	3.03242	13
0	-24	2	16.2452	2.90698	13

0	24	2	18.7065	2.68446	11
−1	−24	−2	1.28348	2.25372	13
1	24	2	1.25857	1.93438	10
−2	−24	−2	6.71851	2.77828	13
2	24	2	0.39683	2.51290	10
−2	24	−2	2.80096	1.12811	9
−2	24	−2	1.98031	0.57930	6
−3	−24	−2	1.23775	2.49014	13
−3	24	−2	−0.2457	0.32040	6
3	24	2	1.53842	2.65285	10
−3	24	−2	−0.0362	0.33974	6
−3	24	−2	−0.2044	0.61756	9
4	24	2	11.1420	2.48194	10
−4	−25	2	12.2870	2.30296	13
−4	25	2	12.0323	1.25942	9
4	25	−2	11.5049	2.36256	10
−3	−25	2	13.3875	2.55687	13
−3	25	2	18.1483	1.87938	9
3	25	−2	14.2246	2.65294	10
−2	−25	2	6.89574	2.44545	13
2	25	−2	2.68622	1.68051	11
1	−25	−2	1.72730	2.47192	13
−1	−25	2	0.29147	2.12708	13
1	25	−2	0.25185	0.88383	11
1	25	−2	−0.6592	0.86170	11
0	−25	2	3.18107	2.58197	13
0	−25	−2	5.09745	2.26192	13
0	25	2	4.22800	1.31570	11
−1	−25	−2	22.8134	3.38157	13
1	25	2	23.0452	2.84512	11
−2	−25	−2	3.92213	2.28861	13
−2	25	−2	2.34784	0.94523	9
2	25	2	4.43571	2.17738	10
−2	25	−2	3.28826	0.58203	6
−3	−25	−2	15.2573	2.54915	13
−3	25	−2	13.6688	1.24774	9
3	25	2	13.1645	2.79741	10
−3	25	−2	13.1096	1.19730	9
4	25	2	2.96957	2.15408	10
−3	−26	2	21.3346	3.10763	13
3	26	−2	19.2771	2.31373	11
2	−26	−2	2.73574	1.58670	13
−2	−26	2	4.79933	2.03540	13
2	26	−2	2.57264	1.03442	11
2	26	−2	2.40572	0.99157	11
1	−26	−2	13.1100	2.40178	13
−1	−26	2	13.0162	2.42380	13
0	−26	2	2.89210	1.84509	13
0	−26	−2	0.50328	1.90581	13
−1	−26	−2	−0.3753	2.27129	13

1	26	2	0.53616	1.20828	11
−2	−26	−2	5.03826	1.84225	13
−2	26	−2	2.43493	0.90854	9
2	26	2	3.16873	1.44579	11
2	26	2	3.71625	1.72789	10
3	26	2	−0.1461	1.50480	10
−2	−27	2	7.51559	1.83063	13
2	−27	−2	5.97674	1.67354	13
1	−27	−2	9.96204	2.21456	13
−1	−27	2	9.33832	1.98548	13
0	−27	2	6.54088	2.05619	13
0	−27	−2	7.65569	1.88849	13
−1	−27	−2	2.60776	1.66437	13
−2	−27	−2	4.54243	1.54296	13
10	0	−3	48.8988	5.70903	13
−9	0	3	16.8927	2.77442	7
−8	0	3	26.3045	2.93768	11
8	0	−3	28.0194	4.86830	13
−7	0	3	−1.8057	1.96288	10
−6	0	3	200.122	13.5976	4
−6	0	3	201.101	14.0818	1
−5	0	3	63.0540	6.17592	1
−5	0	3	64.3334	5.10644	4
−4	0	3	42.5368	3.70355	5
−4	0	3	40.7104	5.02336	1
−4	0	3	42.2224	3.91476	5
−4	0	3	41.7232	3.63247	4
−3	0	3	6.29994	1.20970	3
−3	0	3	9.18633	1.51431	4
−3	0	3	7.49017	2.13515	5
−2	0	3	963.375	57.7590	4
−2	0	3	959.920	58.8575	5
−2	0	3	917.981	57.6854	3
−1	0	3	0.33987	1.63108	5
0	0	−3	16.6362	2.25612	4
0	0	−3	17.2041	2.09183	3
0	0	−3	18.4431	3.09954	5
0	0	−3	20.7667	2.84609	5
0	0	3	15.3265	3.44426	5
−1	0	−3	78.9667	5.39345	3
−1	0	−3	80.9083	5.85708	5
−1	0	−3	81.4663	5.68437	4
−1	0	−3	83.0206	5.74460	5
−1	0	−3	84.5806	5.52284	3
−1	0	−3	82.2707	5.47734	4
−2	0	−3	890.662	55.1143	2
−2	0	−3	891.280	54.3206	3
−2	0	−3	876.354	54.2928	4
−2	0	−3	895.029	54.4767	4
−2	0	−3	898.044	54.3908	3

−3	0	−3	62.4696	4.76782	4
−3	0	−3	61.9952	4.58881	3
−3	0	−3	60.8870	4.42388	4
−3	0	−3	61.7169	5.70609	2
−4	0	−3	133.905	10.7191	2
−4	0	−3	136.393	9.25727	3
−4	0	−3	133.642	9.18413	4
−6	0	−3	2.52305	1.20950	8
−7	0	−3	5.74736	1.61062	8
−8	0	−3	92.4126	6.34583	8
9	0	3	6.31915	2.52119	13
10	−1	−3	17.1116	3.28677	13
10	1	−3	16.3318	3.41543	13
9	−1	−3	47.5516	6.16993	13
−9	−1	3	40.8911	3.76821	7
−9	1	3	46.7737	4.15055	7
9	1	−3	39.0637	7.05522	13
−8	−1	3	54.3218	4.79657	10
8	−1	−3	38.5075	6.15433	13
−8	1	3	45.9728	3.68302	11
8	1	−3	50.4191	6.27719	13
8	1	−3	52.4111	7.35291	13
−7	−1	3	3.07270	2.18803	10
−7	1	3	1.24126	1.85794	10
−6	−1	3	255.313	17.1228	1
−6	1	3	232.523	16.0098	4
−5	1	3	250.834	17.1716	1
−5	1	3	258.003	16.4660	4
−4	−1	3	118.240	8.21508	4
−4	−1	3	120.111	9.07257	5
−4	−1	3	119.183	9.59323	1
−4	1	3	117.818	7.99388	3
−4	1	3	122.605	8.15651	4
−4	1	3	113.154	9.01410	1
−3	−1	3	236.068	15.4482	5
−3	−1	3	228.137	14.1512	4
−3	−1	3	226.976	14.0359	3
−3	1	3	210.313	14.6360	5
−3	1	3	215.089	13.9731	3
−3	1	3	209.342	14.0011	4
−2	−1	3	463.098	29.1345	4
−2	−1	3	473.198	30.3839	5
−2	1	3	474.515	29.0581	3
−2	1	3	479.390	29.2851	4
−2	1	3	465.538	29.0732	4
−2	1	3	477.410	30.0456	5
−1	−1	3	458.605	29.0030	5
1	1	−3	438.393	28.0444	4
−1	1	3	477.764	27.9709	4
−1	1	3	444.125	28.8676	5

0	1	−3	25.4703	2.71909	5
0	1	−3	24.3076	2.09192	3
0	1	−3	25.5593	2.26105	4
0	1	−3	23.9435	2.16387	4
0	1	−3	22.4441	2.63184	5
−1	−1	−3	210.487	14.4153	2
−1	−1	−3	205.158	14.6404	5
−1	−1	−3	219.830	14.0387	3
−1	−1	−3	210.195	13.9751	4
−1	−1	−3	211.258	14.3663	5
−1	−1	−3	215.702	14.1408	4
−1	1	−3	240.564	13.9176	4
−1	1	−3	241.818	13.9565	3
−1	1	−3	236.002	13.8885	3
−1	1	−3	236.793	14.0188	4
−2	−1	−3	331.406	20.6841	2
−2	−1	−3	328.133	19.8054	4
−2	−1	−3	332.970	20.0020	4
−2	−1	−3	328.843	19.8607	5
−2	−1	−3	328.222	19.8704	3
−2	−1	−3	325.671	19.9331	5
−2	1	−3	306.856	19.7436	4
−2	1	−3	295.139	19.6161	3
−2	1	−3	297.898	20.3793	2
−2	1	−3	306.724	19.6865	3
−2	1	−3	308.121	19.6091	4
−3	−1	−3	21.7697	2.73812	4
−3	−1	−3	20.4889	2.49203	3
−3	−1	−3	17.5980	3.32995	2
−3	−1	−3	20.8318	3.05612	1
−3	1	−3	24.9896	2.16383	4
−3	1	−3	22.7082	3.63801	2
−3	1	−3	23.4165	2.36173	4
−3	1	−3	25.3840	2.24717	3
−4	−1	−3	5.60822	1.41337	3
−4	−1	−3	5.38962	1.78004	1
−4	−1	−3	5.35914	2.71871	2
−4	−1	−3	4.82342	1.67312	4
−4	1	−3	2.32697	1.19918	4
−4	1	−3	1.61794	1.15867	3
−5	−1	−3	69.8584	5.18007	4
−5	1	−3	51.9911	4.66564	3
−5	1	−3	54.8120	4.11098	4
−5	1	−3	53.7307	4.24736	4
−6	−1	−3	264.649	16.6805	8
−6	1	−3	256.857	16.5051	8
−7	−1	−3	19.0775	2.63645	8
−7	1	−3	28.1314	2.74255	8
−8	−1	−3	45.2743	3.69402	8
−8	1	−3	40.5335	3.34037	8

8	1	3	37.4476	6.03218	13
9	1	3	10.0542	3.27522	13
10	−2	−3	3.97933	3.06705	13
10	2	−3	0.14158	2.77616	13
−9	−2	3	93.5178	6.57579	7
9	−2	−3	78.1833	8.59325	13
−9	2	3	81.1315	6.52625	7
9	2	−3	94.6907	8.49021	13
−8	−2	3	1.76902	2.07432	10
8	2	−3	0.10286	2.95298	13
8	2	−3	1.31874	2.94277	13
−8	2	3	5.20673	1.15807	11
−7	−2	3	3.66952	2.43757	10
−7	2	3	5.73791	1.38174	11
−6	−2	3	127.435	10.8952	1
−6	2	3	141.615	9.44108	4
−5	−2	3	19.3691	2.55643	5
−5	−2	3	19.5063	4.26737	1
−4	2	3	163.958	12.0515	1
−4	2	3	173.996	11.2863	4
−4	2	3	179.635	11.2895	3
−3	−2	3	322.528	20.1833	4
−3	−2	3	321.716	21.5516	5
−3	2	3	324.567	20.2355	4
−3	2	3	321.316	19.9434	3
−3	2	3	314.282	19.9953	4
−3	2	3	316.597	20.1468	5
−2	−2	3	2066.56	130.958	5
−2	−2	3	2104.87	129.734	4
−2	2	3	2167.98	130.380	5
−2	2	3	2151.47	129.641	4
−2	2	3	2177.36	129.594	3
−2	2	3	2155.20	129.821	4
−1	−2	3	555.786	36.8074	5
1	−2	−3	572.904	36.1932	2
−1	2	3	611.422	35.8051	4
1	2	−3	603.312	35.8971	4
−1	2	3	572.887	36.6500	5
0	−2	−3	1403.71	96.2782	4
0	−2	−3	1594.42	96.8041	5
0	2	−3	1614.36	96.1446	3
0	2	−3	1634.89	96.1107	4
0	2	3	1552.15	97.1351	5
0	2	−3	1611.99	96.4968	5
0	2	−3	1583.94	96.7828	5
0	2	−3	1679.58	96.2253	4
−1	−2	−3	13.8246	2.26732	5
−1	−2	−3	13.3312	1.91027	3
−1	−2	−3	14.1815	1.97105	4
−1	−2	−3	13.3416	2.50967	5

−1	−2	−3	13.4114	1.71080	4
−1	−2	−3	13.4578	2.25955	2
−1	2	−3	10.5387	1.39840	3
−1	2	−3	8.95621	1.37488	4
1	2	3	13.8743	3.23946	5
−1	2	−3	11.3852	1.59061	4
−1	2	−3	10.9367	1.26096	3
−2	−2	−3	379.774	24.6278	2
−2	−2	−3	374.264	23.7720	3
−2	−2	−3	373.976	24.3916	5
−2	−2	−3	377.439	24.1750	5
−2	−2	−3	374.540	23.8932	4
−2	2	−3	381.247	23.4263	3
−2	2	−3	392.820	23.4070	4
−2	2	−3	404.702	23.5284	3
−2	2	−3	374.713	23.4770	4
−2	2	−3	375.884	24.2139	2
−3	−2	−3	304.400	20.4088	3
−3	−2	−3	329.624	20.5600	4
−3	−2	−3	326.680	21.5896	2
−3	−2	−3	292.547	21.0851	1
−3	2	−3	321.883	20.0139	4
−3	2	−3	325.763	20.0283	3
−3	2	−3	315.714	19.9021	4
−3	2	−3	338.551	21.3969	2
−3	2	−3	341.050	20.1474	3
−4	−2	−3	61.0701	6.65962	2
−4	−2	−3	56.9155	5.01271	4
−4	−2	−3	64.7595	5.86579	1
−4	2	−3	61.1017	4.68148	3
−4	2	−3	60.3379	4.32342	4
−4	2	−3	62.6192	4.25138	4
−5	−2	−3	425.568	27.7661	4
−5	−2	−3	438.564	29.9088	2
−5	2	−3	431.757	26.5618	4
−5	2	−3	431.655	26.5433	4
−5	2	−3	449.922	27.5294	3
−6	−2	−3	65.3995	5.45090	10
−6	−2	−3	65.6473	5.08513	8
−6	2	−3	66.8083	4.91327	8
−7	−2	−3	459.609	28.5403	8
−7	2	−3	453.798	28.2391	8
7	2	3	457.856	29.8410	13
−8	−2	−3	28.4480	3.15257	8
8	2	3	28.0106	4.50636	13
9	2	3	1.96164	2.52144	13
−9	2	−3	1.40151	0.82176	9
10	−3	−3	5.67861	2.50228	13
10	3	−3	1.70532	2.80615	13
9	3	−3	37.9900	5.57427	13

−9	3	3	29.5349	3.17571	7
−8	−3	3	5.19545	2.09354	10
8	3	−3	5.43852	3.29280	13
−8	3	3	7.96168	2.14508	7
−7	−3	3	12.4795	3.30550	1
−7	−3	3	9.33113	2.45468	10
−7	3	3	6.89939	1.37242	11
7	3	−3	9.10090	3.45579	13
−6	−3	3	20.6131	4.54264	1
−6	3	3	37.0139	3.39856	4
−5	−3	3	286.590	18.4086	5
−5	−3	3	287.719	19.2760	1
−5	3	3	247.248	17.1004	3
−5	3	3	251.579	17.0297	4
−4	−3	3	1588.96	102.677	1
−4	−3	3	1640.30	102.818	5
−4	3	3	1741.88	101.077	3
−4	3	3	1666.45	100.991	4
−3	−3	3	492.934	34.2232	5
−3	3	3	528.965	32.7229	4
−3	3	3	544.222	32.5193	4
−3	3	3	545.109	32.5373	3
−2	−3	3	3416.34	202.879	5
−2	−3	3	3281.32	201.603	4
−2	3	3	3050.46	201.202	3
−2	3	3	3170.33	201.426	4
2	3	−3	3786.48	202.258	4
−2	3	3	3269.09	202.323	5
−2	3	3	3279.13	201.357	3
−2	3	3	3340.85	201.279	4
−1	−3	3	175.183	12.4928	5
1	−3	−3	183.806	11.9023	2
−1	3	3	176.030	11.2660	3
1	3	−3	171.914	11.3743	4
1	3	−3	179.291	12.1949	5
−1	3	3	181.174	11.3280	4
−1	3	3	181.848	12.2324	5
0	−3	−3	245.220	13.7149	2
0	3	−3	217.482	12.9046	4
0	3	−3	193.004	12.8206	5
0	3	−3	199.305	12.8104	3
0	3	−3	197.266	12.9052	5
0	3	−3	192.317	12.7759	4
0	3	−3	194.070	12.7455	3
−1	−3	−3	1334.62	78.1232	5
−1	−3	−3	1315.89	77.4752	3
−1	−3	−3	1334.96	77.6184	4
−1	−3	−3	1303.70	78.0225	2
−1	3	−3	1263.86	77.1329	3
−1	3	−3	1231.04	77.1379	4

−1	3	−3	1204.71	77.0782	4
1	3	3	1259.95	78.5237	5
−1	3	−3	1190.98	77.0488	3
−2	−3	−3	326.086	22.5096	2
−2	−3	−3	332.038	21.7308	3
−2	−3	−3	341.333	21.9011	4
−2	−3	−3	324.495	22.3236	5
−2	3	−3	365.441	21.3590	3
−2	3	−3	363.310	21.3047	3
−2	3	−3	365.620	21.2223	4
−2	3	−3	348.087	21.2761	4
−3	−3	−3	325.353	19.7041	5
−3	−3	−3	314.357	19.6784	4
−3	−3	−3	325.253	20.7367	2
−3	−3	−3	309.368	19.7639	5
−3	−3	−3	300.012	20.3933	1
−3	3	−3	297.260	18.9611	3
−3	3	−3	294.329	18.7467	4
−3	3	−3	290.713	18.8838	3
−3	3	−3	288.918	18.6783	4
−4	−3	−3	374.074	25.0846	1
−4	−3	−3	379.659	24.4240	4
−4	−3	−3	381.040	25.8400	2
−4	3	−3	380.020	23.1438	4
−4	3	−3	385.918	23.7860	3
−5	−3	−3	34.8753	5.59245	2
−5	−3	−3	31.6328	4.01707	4
−5	−3	−3	32.9437	3.71672	8
−5	−3	−3	37.9997	4.56800	1
−6	−3	−3	10.6397	2.85133	10
−6	−3	−3	7.79014	1.81358	8
−6	3	−3	9.26386	1.15765	9
−6	3	−3	10.0055	1.49020	8
−7	−3	−3	22.7573	3.50652	8
7	3	3	23.9773	5.15729	13
−7	3	−3	31.1556	2.60381	9
−7	3	−3	33.4990	2.75991	8
−8	−3	−3	4.40219	1.73121	8
8	3	3	8.76495	4.11990	13
−8	3	−3	6.89190	1.31674	9
9	3	3	48.1452	5.50989	13
−9	3	−3	45.7940	3.26233	9
10	4	−3	−0.5753	3.38301	13
−9	−4	3	0.64015	1.93484	10
−9	4	3	−0.3055	1.53628	7
9	4	−3	−1.2721	3.59497	13
−8	−4	3	13.5126	2.75311	10
−8	4	3	13.8282	2.47430	7
8	4	−3	15.9661	3.96059	13
−6	4	3	45.3697	3.79318	4

−5	−4	3	3.30205	2.29751	5
−5	−4	3	0.76999	2.75193	1
−4	−4	3	134.742	11.0986	1
−4	−4	3	137.459	11.0378	5
−4	4	3	123.331	8.72324	3
−4	4	3	125.950	8.71756	4
−4	4	3	142.709	9.30961	4
−3	−4	3	244.489	20.4131	5
−3	4	3	317.066	18.7593	3
−3	4	3	315.024	18.9997	4
−3	4	3	321.280	18.7686	4
−2	−4	3	131.959	9.68318	2
−2	−4	3	133.444	10.8943	5
−2	4	3	149.997	9.25988	4
−2	4	3	147.201	9.32957	3
−2	4	3	145.752	9.49116	4
−2	4	3	142.448	9.16722	3
−2	4	3	146.531	9.48675	5
1	−4	−3	2411.45	150.430	2
−1	−4	3	2416.47	151.034	5
−1	4	3	2483.22	150.005	4
1	4	−3	2487.41	149.852	4
−1	4	3	2534.15	149.812	4
−1	4	3	2494.07	150.502	5
1	4	−3	2473.70	150.549	5
1	4	−3	2473.89	149.814	4
−1	4	3	2465.15	149.791	3
0	4	−3	258.725	16.0912	4
−1	−4	−3	542.768	31.6604	5
−1	−4	−3	515.458	31.4848	2
−1	−4	−3	499.390	30.9291	4
−1	4	−3	471.570	30.3171	4
1	4	3	525.575	32.0220	5
−1	4	−3	474.367	30.3555	3
−1	4	−3	467.671	30.2870	3
−1	4	−3	477.882	30.2968	4
−2	4	−3	32.6074	2.55971	3
−2	4	−3	34.6477	2.64071	3
−2	4	−3	37.8160	2.47664	4
−2	4	−3	38.3847	2.42386	4
−3	−4	−3	627.560	40.0036	5
−3	−4	−3	641.386	40.6297	2
−3	−4	−3	597.878	40.3834	1
−3	4	−3	667.560	38.8278	3
−4	−4	−3	11.5231	3.24349	2
−4	−4	−3	14.7382	3.30380	1
−5	−4	−3	490.815	31.8622	1
−5	−4	−3	515.413	33.3707	2
−5	−4	−3	482.868	30.3382	8
−5	4	−3	478.901	30.4180	3

−5	4	−3	473.315	29.6307	6
−6	−4	−3	101.030	7.80938	10
−6	−4	−3	103.836	7.08442	8
−6	4	−3	92.9296	6.40823	8
−6	4	−3	95.2604	6.23936	9
−6	4	−3	92.6514	6.11290	6
−7	−4	−3	280.309	18.0371	10
−7	−4	−3	275.132	18.0371	10
−7	−4	−3	286.251	17.8385	8
−7	4	−3	262.526	17.1515	9
7	4	3	288.229	19.6552	13
−7	4	−3	271.750	16.9842	6
−8	−4	−3	132.541	8.87906	8
−8	4	−3	131.984	8.36160	9
8	4	3	123.677	10.6838	13
−8	4	−3	131.869	8.11460	6
−9	4	−3	4.44624	0.79373	9
9	4	3	1.32323	2.64332	13
10	5	−3	7.36106	3.00777	13
−9	−5	3	1.43596	2.07062	10
−9	5	3	5.76293	1.89594	7
9	5	−3	1.11091	3.42037	13
−8	−5	3	112.345	8.70275	10
8	5	−3	111.238	10.8320	13
−7	−5	3	9.24814	2.81336	10
−7	−5	3	8.79439	3.89174	1
7	5	−3	15.2794	4.57108	13
−6	5	3	223.473	14.2095	4
−5	−5	3	345.398	23.4811	1
−5	−5	3	336.657	22.9965	5
−5	5	3	320.509	20.5425	4
−5	5	3	313.637	20.7102	4
−5	5	3	328.202	20.7943	3
−4	−5	3	35.6096	5.42816	1
−4	−5	3	40.3408	5.15152	2
−4	−5	3	35.1695	5.47565	5
−4	5	3	42.1113	3.29112	4
−4	5	3	37.4186	3.52480	4
−4	5	3	39.4271	3.16578	3
−3	5	3	149.428	9.90598	4
−3	5	3	159.204	9.95693	3
−3	5	3	152.542	10.1374	4
−2	−5	3	220.344	18.4515	5
2	−5	−3	265.806	17.9866	2
−2	5	3	285.783	17.1590	3
−2	5	3	282.075	16.7928	5
−2	5	3	272.700	17.1525	4
−2	5	3	284.863	16.9627	4
−2	5	3	281.256	16.9489	3
1	−5	−3	1498.72	92.8803	2

−1	5	3	1513.68	92.6848	5
1	5	−3	1468.84	92.1100	4
−1	5	3	1550.10	92.1095	4
−1	5	3	1602.95	92.1179	3
1	5	−3	1454.52	92.6789	5
−1	5	3	1511.53	92.2937	4
0	−5	−3	676.795	41.3863	2
0	−5	−3	699.100	41.5984	5
0	5	3	717.683	41.6704	5
0	5	−3	629.209	40.3432	3
0	5	3	699.411	40.6786	4
0	5	−3	605.400	40.3625	4
0	5	−3	606.084	40.3396	4
−1	−5	−3	207.758	15.3472	2
−1	−5	−3	213.976	15.5206	5
−1	5	−3	242.724	14.2570	3
−1	5	−3	243.790	14.1719	4
1	5	3	213.169	15.7856	5
−1	5	−3	235.222	14.1760	3
−1	5	−3	245.723	14.1620	4
−2	−5	−3	348.513	21.2057	5
−2	−5	−3	351.623	21.0459	2
−2	5	−3	275.543	19.2524	3
−2	5	−3	285.840	19.2158	3
−3	−5	−3	15.4665	3.18205	2
−3	−5	−3	12.9647	3.31575	1
−3	−5	−3	16.1092	3.45552	5
−3	5	−3	12.7072	1.42917	3
−3	5	−3	12.5753	1.40254	3
−4	−5	−3	137.521	9.04240	5
−4	−5	−3	139.434	11.0721	2
−4	−5	−3	138.566	10.6571	1
−4	5	−3	108.042	8.12360	3
−4	5	−3	113.643	8.19132	3
−5	−5	−3	97.9206	8.94788	1
−5	−5	−3	97.4985	9.78113	2
−5	−5	−3	102.064	7.30351	8
−5	5	−3	99.6398	6.22916	6
−5	5	−3	100.440	6.94676	3
−6	−5	−3	91.3130	9.37004	2
−6	5	−3	59.1698	4.07948	6
−6	5	−3	58.6667	4.22677	9
−7	−5	−3	38.1120	4.52445	10
−7	−5	−3	39.0321	4.49263	8
−7	−5	−3	36.1453	4.36786	10
7	5	3	37.5159	6.03532	13
−7	5	−3	53.3840	3.24319	9
−7	5	−3	48.1765	3.26448	9
−8	−5	−3	38.2817	3.65951	8
−8	5	−3	38.9130	2.86687	9

8	5	3	37.3644	6.16468	13
−8	5	−3	38.2668	2.56169	6
9	5	3	32.3072	4.64695	13
−9	5	−3	22.6037	1.84541	9
10	6	−3	0.90280	2.61499	13
9	6	−3	17.4590	4.38085	13
−8	−6	3	5.93011	2.46362	10
8	6	−3	8.42908	3.89052	13
−8	6	3	8.96935	2.24179	7
−7	−6	3	119.288	9.32333	10
−7	−6	3	118.300	11.2824	1
−7	6	3	118.075	7.28953	11
7	6	−3	98.1186	10.5690	13
−7	6	3	121.911	8.51228	7
−6	−6	3	178.994	16.3517	1
−6	6	3	247.323	14.2849	3
−5	−6	3	72.0399	8.07184	1
−5	−6	3	67.0424	7.58700	5
−5	6	3	73.2725	5.43440	3
−5	6	3	75.5789	5.30603	4
−5	6	3	73.9466	5.11631	4
−4	−6	3	321.166	20.6225	5
−4	−6	3	319.720	20.1294	1
−4	6	3	252.550	17.1190	3
−4	6	3	243.668	16.9213	4
−4	6	3	234.830	17.0942	4
−3	−6	3	55.2011	7.91655	5
−3	−6	3	58.2812	5.68833	2
−3	6	3	66.7345	4.89210	4
−3	6	3	71.2866	4.62395	4
−3	6	3	65.1450	4.56830	3
−2	−6	3	154.033	10.1618	2
2	−6	−3	149.051	10.9069	2
−2	6	3	146.190	9.51063	3
−2	6	3	144.780	9.53719	4
−2	6	3	141.703	9.75205	4
1	−6	−3	93.8176	8.15158	2
1	6	−3	117.200	7.65412	5
−1	6	3	96.2201	7.31411	4
1	6	−3	117.502	7.11776	4
1	6	−3	125.621	7.24271	4
−1	6	3	100.634	7.04613	4
−1	6	3	96.2144	7.56658	5
−1	6	3	96.3108	7.03668	3
0	−6	−3	699.185	49.4159	2
0	6	3	701.385	49.4775	5
0	6	−3	850.569	48.4678	3
0	6	−3	856.908	48.4804	4
0	6	−3	897.598	48.5234	4
0	6	−3	842.734	48.4542	4

0	6	3	709.278	48.6842	4
−1	6	−3	10.4990	1.08282	4
−1	6	−3	11.6851	1.20745	3
−1	6	−3	11.2168	1.09001	4
−1	6	−3	10.4326	1.25450	3
−2	−6	−3	1197.91	77.4535	1
−2	−6	−3	1214.51	77.4748	5
−2	−6	−3	1215.53	77.2585	2
−2	6	−3	1294.34	75.5781	3
−2	6	−3	1302.82	75.5191	3
−3	−6	−3	61.9665	6.88788	2
−3	−6	−3	64.8980	6.74379	5
−3	−6	−3	57.9918	6.86587	1
−3	6	−3	71.5222	4.51585	3
−3	6	−3	71.9761	4.44567	3
−4	−6	−3	106.171	8.80701	5
−4	−6	−3	101.576	9.48461	1
−4	−6	−3	108.646	10.0637	2
−4	6	−3	115.211	7.20145	3
−4	6	−3	115.426	7.26051	3
−5	−6	−3	10.4588	3.54884	1
−5	−6	−3	10.5765	3.83343	2
−5	−6	−3	12.7106	2.34200	8
−5	6	−3	8.96355	0.95335	6
−5	6	−3	10.4924	1.65459	3
−5	6	−3	11.2934	1.07652	9
−6	−6	−3	90.5236	7.16282	10
−6	−6	−3	76.6638	7.85889	1
−6	−6	−3	73.0774	6.24637	8
−6	6	−3	87.3641	5.19602	6
6	6	3	73.9217	8.65170	13
−6	6	−3	84.0418	5.31951	9
−7	−6	−3	400.148	23.7644	8
−7	−6	−3	398.318	24.1370	10
−7	−6	−3	384.534	24.0101	10
−7	6	−3	340.702	22.8356	9
−7	6	−3	325.924	22.8382	9
7	6	3	393.244	25.1204	13
−7	6	−3	372.890	22.7190	6
−8	−6	−3	3.10253	1.53737	8
−8	6	−3	1.48898	0.82658	9
8	6	3	2.20156	3.29871	13
−8	6	−3	1.07076	0.44577	6
9	6	3	6.97307	2.79103	13
10	7	−3	10.6759	3.26698	13
−9	−7	3	15.8666	3.18635	10
−9	7	3	15.9856	2.45284	7
9	7	−3	17.9415	4.27936	13
−9	7	3	16.0818	1.55107	9
−8	−7	3	−3.5550	2.25320	10

8	7	−3	−4.4887	4.68107	13
−8	7	3	1.96425	1.85952	7
−7	−7	3	182.145	12.6211	10
−7	−7	3	186.285	14.9843	1
7	7	−3	174.624	13.1599	13
−7	7	3	146.357	11.5180	7
−6	7	3	16.0341	2.22023	3
−6	7	3	17.3045	2.74354	7
−5	−7	3	69.1066	7.66065	2
−5	−7	3	69.8942	8.27388	1
−5	−7	3	70.6563	8.29909	5
−5	7	3	79.5269	5.20825	4
−5	7	3	80.4197	5.10952	4
−5	7	3	80.2064	5.69972	3
−4	−7	3	216.401	17.6565	5
−4	−7	3	211.531	17.3518	1
−4	−7	3	214.079	16.4571	2
−4	7	3	246.222	14.8477	4
−4	7	3	251.752	14.6611	4
−4	7	3	247.033	14.8918	3
3	−7	−3	22.4819	3.91369	2
−3	7	3	19.8234	2.45646	4
−3	7	3	24.4489	2.16811	3
−3	7	3	21.6601	2.13049	4
2	−7	−3	123.116	9.38584	2
−2	7	3	121.269	8.02569	3
−2	7	3	119.238	7.97105	4
−2	7	3	120.342	8.24097	4
1	−7	−3	114.554	8.59431	2
1	7	−3	96.8263	7.19300	4
1	7	−3	94.6282	7.45324	5
−1	7	3	111.460	7.17459	4
−1	7	3	115.458	7.16455	3
0	−7	−3	4190.57	256.842	2
0	7	3	4189.56	255.931	4
0	7	−3	4210.08	255.583	4
0	7	−3	4390.58	255.646	4
0	7	3	4236.91	256.736	5
0	7	−3	4122.76	255.597	3
−1	−7	−3	226.434	14.5540	2
−1	7	−3	193.592	12.7586	3
1	7	3	216.552	14.7731	5
−1	7	−3	188.503	12.6401	4
−1	7	−3	207.429	12.6718	4
−2	−7	−3	91.9577	8.04904	5
−2	−7	−3	96.6452	8.20312	1
−2	−7	−3	96.2774	7.88368	2
−2	7	−3	87.1144	5.73504	3
−2	7	−3	84.4552	5.77430	3
−3	−7	−3	72.7222	7.05901	5

−3	−7	−3	73.0134	7.23872	1
−3	−7	−3	74.3651	7.15578	2
−3	7	−3	67.9380	4.55651	3
−3	7	−3	69.0436	4.62656	3
−4	−7	−3	44.1878	6.66898	2
−4	−7	−3	43.6985	6.10369	1
−4	−7	−3	47.6330	5.84598	5
−4	7	−3	52.3826	3.47449	3
−4	7	−3	51.6493	3.54099	3
−5	−7	−3	7.41191	2.14806	8
−5	−7	−3	6.76723	3.44706	2
−5	−7	−3	6.30775	3.53886	1
−5	7	−3	4.17942	1.81435	7
−5	7	−3	3.35150	0.60405	9
−5	7	−3	3.57355	0.51802	6
−5	7	−3	3.39101	0.47783	6
−5	7	−3	3.31446	0.92822	3
−6	−7	−3	95.5417	7.17126	8
−6	−7	−3	107.526	8.05790	10
−6	−7	−3	89.0058	8.93759	1
−6	7	−3	90.8592	5.93083	6
−6	7	−3	90.0817	6.04778	9
6	7	3	102.423	10.2098	13
−7	−7	−3	60.9968	5.56593	10
−7	−7	−3	62.1469	5.62555	8
−7	−7	−3	59.0434	5.72366	10
7	7	3	59.6354	7.55249	13
−8	−7	−3	8.31215	2.05907	8
−8	7	−3	11.8420	0.94334	6
8	7	3	7.05585	3.74999	13
−8	7	−3	10.1130	0.91891	6
9	7	3	5.64262	2.64643	13
10	8	−3	26.5635	4.59515	13
−9	−8	3	8.36239	1.66894	8
−9	−8	3	8.95023	2.40873	10
−9	8	3	8.33854	0.87313	6
9	8	−3	7.78161	3.67150	13
−9	8	3	8.51846	1.13175	9
−9	8	3	7.78541	1.64383	7
−8	−8	3	20.1010	3.57335	10
−8	8	3	28.7361	3.60409	7
8	8	−3	10.7460	4.49359	13
−8	8	3	20.9372	1.92703	9
−7	−8	3	51.9328	8.71216	1
−7	8	3	74.0077	4.80028	9
−7	8	3	76.7810	5.89497	7
−6	−8	3	25.6463	5.56911	1
−6	−8	3	22.3712	4.41584	10
−6	8	3	32.8286	3.73656	7
−6	8	3	29.3190	2.44369	9

−6	8	3	30.7908	2.10568	11
−5	−8	3	11.9342	4.66446	5
−5	−8	3	15.1747	3.81460	2
−5	−8	3	15.7329	4.44675	1
−5	8	3	18.8339	2.25293	3
−4	−8	3	77.8945	7.90244	2
−4	−8	3	74.7141	8.69122	1
4	−8	−3	94.8759	8.55258	2
−4	8	3	97.1158	6.00151	4
−4	8	3	93.6554	6.25620	3
−4	8	3	94.0530	6.14413	4
−3	−8	3	154.627	12.5707	1
3	−8	−3	154.360	12.2145	2
−3	−8	3	169.394	11.6524	2
−3	8	3	155.176	10.0951	4
−3	8	3	158.528	10.2402	3
−3	8	3	152.525	10.3005	4
2	−8	−3	103.051	8.67098	2
−2	8	3	108.769	7.51745	4
−2	8	3	110.279	7.24196	3
−2	8	3	104.953	7.26870	4
1	−8	−3	458.003	28.4703	2
1	8	−3	426.710	27.2893	5
−1	8	3	440.711	27.0851	3
−1	8	3	443.536	27.1109	4
1	8	−3	412.249	27.0687	4
0	−8	−3	304.591	21.4810	2
0	8	−3	336.939	20.1814	3
0	8	−3	328.411	20.1373	4
0	8	−3	330.643	20.1435	4
0	8	3	318.983	21.0282	4
−1	−8	−3	477.621	32.7978	2
−1	8	−3	519.154	31.2043	3
−1	8	−3	533.395	31.0501	4
−1	8	−3	506.525	31.0373	4
−2	−8	−3	221.085	16.7694	1
−2	−8	−3	214.171	16.6192	5
−2	−8	−3	222.064	16.3631	2
−2	8	−3	243.586	14.2578	3
−2	8	−3	248.411	14.3195	3
−3	−8	−3	28.0423	5.00800	5
−3	−8	−3	29.9405	5.32855	1
−3	−8	−3	29.0112	5.01307	2
−3	8	−3	23.4058	1.88039	3
−3	8	−3	26.0823	1.86076	3
−4	−8	−3	27.8303	4.86874	5
−4	−8	−3	28.7801	5.36176	1
−4	−8	−3	29.1955	5.36832	2
−4	8	−3	32.7625	2.34904	3
−4	8	−3	33.0158	2.02791	6

−5	−8	−3	9.95499	2.21099	8
−5	−8	−3	7.04056	3.81096	2
−5	−8	−3	7.01031	3.96017	1
−5	8	−3	16.3838	1.01259	6
−5	8	−3	13.5701	1.02146	6
−5	8	−3	12.3824	2.07997	7
−5	8	−3	13.1067	1.11972	9
−6	−8	−3	15.9925	3.26494	10
−6	−8	−3	17.1027	4.50417	1
−6	−8	−3	15.4096	2.62735	8
6	8	3	16.3004	4.91072	13
−6	8	−3	15.7466	2.97953	12
−6	8	−3	19.2222	1.36111	6
−6	8	−3	19.0296	1.55790	9
−6	8	−3	20.4403	1.38566	6
7	8	3	5.38680	3.96786	13
−7	8	−3	7.84536	1.03453	9
−7	8	−3	7.91708	0.76160	6
−7	8	−3	8.08367	0.77617	6
−8	−8	−3	−1.0790	1.66044	10
−8	−8	−3	2.48623	1.65035	8
−8	8	−3	0.99768	0.26055	6
−8	8	−3	0.93585	0.25323	6
−8	8	−3	1.18869	0.59991	9
8	8	3	−1.5624	3.37022	13
−8	8	−3	−1.0564	0.61511	9
9	8	3	8.59873	2.62582	13
−9	9	3	5.81963	0.70220	6
−9	9	3	6.68404	0.92241	9
−9	9	3	5.32742	1.81257	7
−8	9	3	21.1739	1.67442	6
−8	9	3	23.6651	1.96024	9
−8	9	3	20.8027	3.37676	7
−7	−9	3	22.9605	4.52823	10
−7	−9	3	29.6554	6.14771	1
7	9	−3	25.6326	5.39156	13
−7	9	3	34.8917	2.34529	6
−7	9	3	36.8469	2.58419	9
−7	9	3	32.8347	3.84950	7
−6	−9	3	15.7028	3.29529	10
6	−9	−3	8.23860	3.85366	2
−6	−9	3	9.39894	4.20119	1
−6	9	3	3.36981	1.82718	7
−5	−9	3	35.8306	5.36743	2
−5	−9	3	35.9318	6.16884	5
5	−9	−3	29.2094	5.41825	2
−5	−9	3	40.1996	6.51938	1
−5	9	3	27.9776	2.87950	3
−5	9	3	26.7792	2.12498	6
4	−9	−3	26.4461	4.85455	2

−4	−9	3	23.0177	4.31403	2
−4	−9	3	24.7968	5.33273	1
−4	9	3	28.4155	2.66988	3
−4	9	3	28.3168	2.36113	4
−4	9	3	30.1895	2.24896	4
3	−9	−3	103.776	9.41017	2
−3	9	3	106.584	7.21062	3
−3	9	3	102.576	7.00430	4
−3	9	3	109.196	7.23968	4
2	−9	−3	33.3965	4.85254	2
−2	9	3	34.2075	3.06616	3
−2	9	3	34.8785	3.05837	4
1	−9	−3	74.3795	6.86909	2
−1	9	3	70.2635	4.90446	4
1	9	−3	54.5251	4.64437	5
−1	9	3	70.1858	4.79442	3
1	9	−3	55.6941	4.77679	4
0	−9	−3	22.5475	4.25937	2
0	9	−3	33.2812	2.74958	4
0	9	−3	36.9273	2.90928	4
−1	−9	−3	62.6727	6.73736	2
−1	9	−3	75.5519	4.65160	4
−1	9	−3	73.2167	4.86264	3
−1	9	−3	72.4219	4.64482	4
−2	−9	−3	39.0194	5.45272	2
−2	−9	−3	39.4848	5.35023	1
−2	9	−3	36.9936	2.66062	3
−3	−9	−3	7.08985	3.08455	1
−3	−9	−3	7.54756	3.38374	2
−3	−9	−3	7.71545	3.22486	5
−3	9	−3	5.66481	0.72855	3
−4	−9	−3	120.928	10.8977	2
−4	−9	−3	114.696	11.1169	1
−4	9	−3	114.229	6.99439	6
−4	9	−3	116.425	7.00965	6
−4	9	−3	111.176	7.14916	3
−5	−9	−3	38.0238	5.98796	10
−5	−9	−3	40.5481	4.45407	8
−5	−9	−3	43.7959	6.75121	1
−5	−9	−3	47.6445	7.95550	2
−5	9	−3	44.1842	3.10299	9
−5	9	−3	47.7951	2.93284	6
−5	9	−3	50.8153	3.02970	9
−5	9	−3	49.6327	2.94910	6
−5	9	−3	46.3080	4.22964	7
−6	−9	−3	9.58418	3.69905	1
−6	−9	−3	13.7048	3.29607	10
−6	−9	−3	13.5491	2.61017	8
6	9	3	11.6564	4.88471	13
−6	9	−3	8.53029	2.12018	7

−6	9	−3	18.7796	3.11507	12
−6	9	−3	9.92450	1.13613	9
−6	9	−3	11.6757	1.14178	9
−6	9	−3	8.00274	0.83380	6
−6	9	−3	9.44791	0.91891	6
−7	−9	−3	0.57708	1.98444	8
−7	−9	−3	−0.6988	2.49894	10
7	9	3	−2.7206	3.53803	13
−8	−9	−3	23.4645	2.95350	10
−8	−9	−3	20.3506	3.10256	8
8	9	3	23.9889	4.33223	13
−9	−10	3	6.50974	2.34591	10
−9	−10	3	3.80809	1.80455	8
−9	10	3	3.89354	0.54707	6
−9	10	3	4.42855	0.74899	9
−9	10	3	4.62558	1.73462	7
9	10	−3	4.58357	3.06174	13
−8	−10	3	20.5910	3.48244	10
−8	10	3	20.9367	3.45320	7
8	10	−3	21.5543	4.50997	13
−8	10	3	22.5244	1.90186	9
−8	10	3	24.8304	1.71482	6
−7	−10	3	16.1345	5.76050	1
−7	−10	3	13.2475	3.32221	10
7	10	−3	14.2649	4.01118	13
−7	10	3	15.7809	2.64052	7
−7	10	3	16.4656	1.80048	9
−7	10	3	17.5208	1.44412	6
−6	−10	3	29.8140	4.84310	10
6	−10	−3	31.8793	6.20225	2
−6	−10	3	33.3577	6.92985	1
6	10	−3	30.8818	5.74706	13
−6	10	3	30.2114	3.61351	7
−6	10	3	30.4678	2.21578	6
−6	10	3	32.6968	2.47223	9
−5	−10	3	10.0270	4.21570	1
5	−10	−3	8.77694	3.68655	2
−5	10	3	11.7112	1.11699	6
−5	10	3	9.62373	1.67937	3
−5	10	3	15.0062	2.20743	7
4	−10	−3	31.3031	5.19845	2
−4	−10	3	24.5638	5.35851	1
−4	10	3	30.5486	2.08743	4
−4	10	3	28.4735	2.09134	4
−4	10	3	28.3013	2.76722	3
3	−10	−3	19.1626	4.13676	2
−3	10	3	18.9583	2.15507	4
−3	10	3	16.8021	1.85512	4
−3	10	3	19.0653	2.06821	3
2	−10	−3	161.895	12.6315	2

−2	10	3	161.486	10.7569	3
−2	10	3	167.117	10.7005	4
1	−10	−3	23.6470	4.54278	2
−1	10	3	26.2594	2.81269	4
1	10	−3	35.6237	2.90451	4
0	−10	−3	1042.68	63.9330	2
0	10	−3	1017.20	62.0863	4
0	10	−3	993.943	62.0502	4
−1	−10	−3	84.1271	8.15239	2
−1	10	−3	82.1376	5.28125	4
−1	10	−3	83.3250	5.58972	3
−1	10	−3	83.2562	5.27943	4
−2	−10	−3	181.586	13.0703	2
−2	−10	−3	174.089	13.3895	1
−2	10	−3	146.411	10.2629	3
−3	−10	−3	178.872	14.6816	1
−3	−10	−3	179.430	14.4575	2
−3	10	−3	188.821	11.3016	3
−4	−10	−3	14.4029	4.26936	1
−4	10	−3	4.71681	1.88624	7
−4	10	−3	4.97378	0.44136	6
−4	10	−3	4.87240	0.45153	6
−5	−10	−3	106.034	11.1909	1
−5	−10	−3	104.671	11.0427	2
−5	−10	−3	97.8396	8.69058	10
−5	10	−3	110.594	6.44775	6
−5	10	−3	109.382	6.44123	6
−5	10	−3	101.665	8.34465	12
−5	10	−3	102.926	6.50583	9
−5	10	−3	109.691	6.55625	9
−5	10	−3	99.4938	7.59716	7
−6	−10	−3	29.5398	5.93814	1
−6	−10	−3	28.0128	4.51608	10
−6	−10	−3	29.1751	3.79576	8
−6	10	−3	25.0432	1.91923	9
−6	10	−3	25.1268	1.66628	6
−6	10	−3	24.7787	3.38108	7
−6	10	−3	27.6230	4.07006	12
−6	10	−3	23.9844	1.86456	9
6	10	3	27.1777	5.45908	13
−6	10	−3	25.4296	1.65653	6
−7	−10	−3	59.3764	4.95230	8
−7	−10	−3	58.5053	5.38511	10
−7	−10	−3	51.9968	5.53752	10
−7	10	−3	48.8185	3.21159	9
−7	10	−3	41.3999	3.18241	9
−7	10	−3	47.5604	6.81917	12
7	10	3	56.4879	7.17983	13
−8	−10	−3	51.5268	4.60891	8
−8	−10	−3	50.9278	4.50581	10

8	10	3	51.4919	6.43112	13
−9	−11	3	28.2485	3.77481	10
−9	−11	3	29.3014	4.00754	8
−9	11	3	30.7611	1.99790	6
−9	11	3	31.6472	2.15412	9
−9	11	3	31.0807	3.32064	7
9	11	−3	25.0743	4.64306	13
−8	−11	3	1.23357	2.56043	10
−8	11	3	2.11059	0.76843	9
−8	11	3	2.27358	1.90707	7
8	11	−3	2.25755	3.44302	13
−8	11	3	2.75118	0.58145	6
−7	−11	3	77.4565	10.2977	1
−7	−11	3	76.5385	7.11629	10
−7	11	3	67.7263	5.88707	7
7	11	−3	78.7492	8.55415	13
−7	11	3	67.6943	4.77664	9
−7	11	3	67.0481	4.54287	6
−6	−11	3	127.144	12.8309	1
−6	−11	3	104.763	9.12884	10
−6	11	3	104.404	6.86269	9
6	11	−3	102.558	10.0522	13
−6	11	3	104.872	7.83520	7
−6	11	3	102.030	6.61591	6
5	−11	−3	14.3989	4.18989	2
−5	−11	3	9.37942	4.33262	1
−5	11	3	12.5381	1.62807	9
−5	11	3	12.8111	2.22552	7
−5	11	3	12.4910	1.18805	6
−5	11	3	12.5257	1.97787	3
4	−11	−3	174.723	13.8800	2
−4	−11	3	163.246	14.8491	1
−4	11	3	170.581	11.1977	3
3	−11	−3	0.83987	2.87282	2
−3	11	3	0.95489	0.99579	3
−3	11	3	−0.2605	0.88123	4
−3	11	3	0.77815	0.81920	4
2	−11	−3	198.767	15.0977	2
−2	11	3	201.873	12.9949	4
−2	11	3	203.341	13.1465	3
1	−11	−3	646.223	40.2999	2
−1	11	3	616.065	38.5065	4
1	11	−3	603.905	38.4821	4
0	−11	−3	61.4153	7.36825	2
0	11	−3	79.5403	5.41710	4
0	11	−3	84.7369	5.48095	4
−1	−11	−3	15.4858	4.02261	2
−1	11	−3	22.2092	1.63341	4
−1	11	−3	22.6121	1.62960	4
−2	−11	−3	153.460	12.9376	1

−2	−11	−3	161.958	13.0136	2
−2	11	−3	164.329	10.1378	3
−3	−11	−3	66.8977	7.75635	1
−3	−11	−3	72.1512	7.89971	2
−3	11	−3	53.7233	3.57123	6
−3	11	−3	57.0256	3.76028	3
−4	−11	−3	75.5840	8.83837	2
−4	−11	−3	69.9402	9.07589	1
−4	11	−3	65.4435	4.10702	6
−4	11	−3	69.8375	4.14057	9
−4	11	−3	63.7757	4.14595	9
−4	11	−3	69.3488	4.09936	6
−4	11	−3	63.5395	5.29538	7
−5	−11	−3	2.72392	4.21466	1
−5	−11	−3	3.47349	2.86905	10
−5	11	−3	3.66495	0.51415	9
5	11	3	1.84965	4.42066	13
−5	11	−3	0.31972	2.59831	12
−5	11	−3	5.47753	1.86471	7
−6	−11	−3	22.9422	3.89587	10
−6	−11	−3	24.1036	3.43300	8
−6	−11	−3	24.6537	5.81731	1
6	11	3	20.5561	4.25488	13
−6	11	−3	12.9581	1.13403	9
−6	11	−3	17.0828	3.18237	12
−6	11	−3	13.7259	1.18491	9
−7	−11	−3	16.4188	3.20445	10
−7	−11	−3	11.2951	2.62969	8
−7	−11	−3	23.6563	4.25740	10
−7	11	−3	15.0424	3.10584	12
−8	−11	−3	−0.3297	1.53534	10
−8	−11	−3	−0.1006	1.59463	8
8	11	3	0.34432	2.74660	13
−9	−12	3	5.54906	2.30846	10
−9	12	3	7.03019	0.76295	9
−9	12	3	4.75091	1.61722	7
−9	12	3	7.58061	0.91293	9
−8	−12	3	27.9901	4.73612	10
−8	12	3	28.1063	3.19053	7
−8	12	3	31.4037	2.08974	6
8	12	−3	44.4339	8.22172	13
−8	12	3	30.6673	2.27867	9
−7	−12	3	2.33074	3.86624	1
−7	−12	3	3.10648	2.71730	10
−7	12	3	1.82087	0.58410	6
7	12	−3	0.73920	4.06720	13
−7	12	3	0.83605	1.94306	7
−7	12	3	1.63808	0.67292	9
−6	−12	3	119.336	13.5924	1
−6	12	3	141.611	8.95599	9

−6	12	3	146.960	9.92435	7
−6	12	3	144.460	8.71169	6
5	−12	−3	267.183	20.2055	2
−5	−12	3	265.689	21.0698	1
−5	12	3	270.297	16.0434	6
−5	12	3	251.230	16.2652	9
−5	12	3	257.810	16.9484	7
4	−12	−3	218.717	17.2209	2
−4	12	3	225.556	14.4419	3
−4	12	3	222.033	13.6420	6
3	−12	−3	92.5215	9.08340	2
−3	12	3	92.5015	6.29359	4
−3	12	3	92.9590	6.60686	3
−3	12	3	91.5254	6.18887	4
2	−12	−3	131.343	11.1973	2
−2	12	3	132.526	9.14215	3
−2	12	3	134.604	8.92706	4
1	−12	−3	36.5683	5.45721	2
1	12	−3	28.2298	3.29889	4
−1	12	3	44.0049	3.63974	4
0	12	−3	113.902	7.68753	4
−1	−12	−3	221.872	15.6687	2
−1	12	−3	194.182	12.2942	4
−1	12	−3	195.296	12.2948	4
−2	12	−3	38.5585	2.89265	3
−3	−12	−3	243.783	18.6624	1
−3	−12	−3	238.327	18.3284	2
−3	12	−3	216.899	14.2167	6
−3	12	−3	243.190	14.2175	6
−3	12	−3	234.028	14.4048	3
−4	−12	−3	67.5664	8.68013	1
−4	−12	−3	74.0016	8.45053	2
−4	12	−3	59.5234	3.88321	9
−5	−12	−3	65.6729	6.72237	10
−5	−12	−3	67.2013	8.98183	1
5	12	3	62.0348	7.28016	13
−6	−12	−3	7.73351	3.23134	10
−6	−12	−3	9.18421	4.30406	1
−6	12	−3	8.13088	2.80477	12
6	12	3	6.44802	3.58742	13
−7	−12	−3	27.2504	3.64099	8
−7	−12	−3	22.7829	4.20708	10
−7	−12	−3	23.3648	3.96337	10
7	12	3	20.5469	4.26578	13
−7	12	−3	25.3067	3.46854	12
−8	−12	−3	16.5634	3.03100	10
8	12	3	15.1599	3.15880	13
−9	−13	3	2.91591	2.00700	10
−8	−13	3	17.1680	3.57989	10
−8	13	3	19.0814	1.41843	6

−8	13	3	21.5920	3.22791	7
−8	13	3	18.8988	1.39636	6
−8	13	3	20.9894	1.61579	9
−7	−13	3	75.3784	7.38853	10
−7	−13	3	74.9505	9.80036	1
−7	13	3	69.8022	4.55309	6
−7	13	3	69.6043	4.77006	9
−7	13	3	71.8563	6.07406	7
−6	−13	3	31.7061	7.12759	1
−6	−13	3	26.9533	5.13623	10
−6	13	3	42.0522	4.61508	7
−6	13	3	40.8702	2.62269	6
−6	13	3	40.3765	2.88975	9
5	−13	−3	18.5509	4.73986	2
−5	−13	3	21.1342	5.72723	1
−5	13	3	16.4416	1.45570	6
−5	13	3	14.6468	1.89704	9
−5	13	3	19.4199	2.90453	7
5	13	−3	29.5568	5.42712	10
4	−13	−3	49.3597	6.98061	2
−4	13	3	45.1411	4.13077	7
−4	13	3	47.3474	4.00221	3
−4	13	3	47.1901	3.13688	6
3	−13	−3	71.4425	7.88406	2
−3	13	3	67.6759	4.65677	4
−3	13	3	67.0310	5.21314	3
−3	13	3	63.1794	4.56486	4
2	−13	−3	97.3820	9.26193	2
−2	13	3	95.0054	6.72517	4
1	−13	−3	657.532	41.6954	2
−1	13	3	661.435	39.4311	4
1	13	−3	583.114	39.3344	4
0	−13	−3	19.5758	4.49693	2
0	13	−3	30.9583	2.79797	4
−1	−13	−3	96.3064	9.10027	2
−2	−13	−3	29.4969	5.42505	2
−2	13	−3	27.0620	2.28681	3
−3	−13	−3	25.2090	5.21498	2
−3	−13	−3	23.4512	5.48729	1
−3	13	−3	16.4042	1.39906	3
−3	13	−3	18.0104	1.16971	6
−3	13	−3	16.6755	1.44118	3
−3	13	−3	16.0503	1.20442	6
−4	−13	−3	12.7085	3.54456	10
−4	−13	−3	15.5130	4.68917	2
−4	−13	−3	13.0273	4.75740	1
−4	13	−3	17.1461	1.16037	9
−4	13	−3	22.1380	3.88582	12
−4	13	−3	17.0600	1.19673	9
−5	−13	−3	611.163	39.6863	10

−5	−13	−3	637.018	42.8730	1
−5	13	−3	626.731	39.0262	12
5	13	3	605.038	39.5969	13
−6	−13	−3	16.5958	5.68160	1
−6	13	−3	26.4928	3.95688	12
−7	−13	−3	25.5908	4.40005	10
−7	−13	−3	23.2993	4.14368	10
7	13	3	14.7299	3.99937	13
−7	13	−3	27.0120	4.25878	12
−8	−13	−3	4.62908	2.02940	10
8	13	3	7.30580	2.36399	13
−9	−14	3	28.9981	3.40668	10
−8	−14	3	4.90506	2.94370	10
−8	14	3	8.22620	2.10026	7
−8	14	3	9.79295	0.75922	6
−8	14	3	9.02555	1.03545	9
−8	14	3	11.1626	0.78053	6
−7	−14	3	3.07514	2.88915	10
−7	14	3	5.47240	0.65872	6
−7	14	3	4.90344	2.11557	7
−7	14	3	4.79303	0.87255	9
−6	−14	3	24.3718	4.62623	10
−6	−14	3	28.4323	6.58731	1
−6	14	3	18.3859	1.98538	9
−6	14	3	18.6082	1.56712	6
−6	14	3	19.1294	2.88299	7
5	−14	−3	23.7063	5.76910	2
−5	−14	3	23.2840	6.22913	1
−5	14	3	21.5920	3.18648	7
−5	14	3	18.6584	2.13043	9
5	14	−3	30.4290	5.04285	10
−5	14	3	20.8948	1.72147	6
4	−14	−3	135.754	12.3161	2
−4	14	3	127.180	8.97943	7
−4	14	3	129.967	8.48391	9
−4	14	3	129.183	8.97129	3
−4	14	3	131.772	8.13784	6
3	−14	−3	42.6077	6.53631	2
−3	14	3	42.3746	3.73249	3
−3	14	3	35.2372	2.77086	4
−3	14	3	38.0021	2.85048	4
2	−14	−3	1.69952	2.93430	2
−2	14	3	3.33222	1.27232	4
1	−14	−3	109.439	10.3926	2
1	14	−3	86.7418	7.33293	4
−1	14	3	110.649	7.43060	4
0	−14	−3	333.038	24.3404	2
0	14	−3	357.588	21.7913	4
−1	−14	−3	53.9761	6.86033	2
−2	−14	−3	114.053	11.2119	2

−2	14	−3	134.683	7.83588	6
−3	−14	−3	15.3243	4.45587	2
−3	−14	−3	15.7711	5.01467	1
−3	14	−3	12.7124	1.28937	9
−3	14	−3	13.3017	1.06307	6
−3	14	−3	13.2565	1.11620	9
−3	14	−3	14.2086	1.27166	3
−3	14	−3	16.3640	1.81373	3
−3	14	−3	13.1831	0.98525	6
−4	−14	−3	10.5764	4.71246	1
−4	14	−3	14.9605	1.09816	9
−4	14	−3	12.7447	3.80231	12
−4	14	−3	12.5308	0.99493	9
−5	−14	−3	2.43752	3.93391	1
−5	−14	−3	2.10259	2.90807	10
−5	14	−3	−1.1553	2.55174	12
−6	−14	−3	74.1596	7.23002	10
−6	14	−3	68.4924	6.81903	12
−7	−14	−3	11.7957	2.62051	10
−7	−14	−3	12.5756	2.42850	10
−7	14	−3	12.0635	2.78230	12
−8	−14	−3	20.5355	2.64745	10
−8	−15	3	17.7654	2.96907	10
−8	15	3	11.7256	2.21000	7
−8	15	3	10.1544	0.92144	9
−8	15	3	10.7490	0.92181	9
−7	−15	3	3.41996	2.86480	10
−7	15	3	−0.3299	1.93026	7
−7	15	3	2.39951	0.69941	9
−7	15	3	2.10491	0.44194	6
−7	15	3	2.12262	0.51864	6
−6	−15	3	86.1802	10.8160	1
−6	15	3	72.7054	4.95561	9
−6	15	3	69.0240	5.99944	7
−6	15	3	71.7899	4.65466	6
−5	15	3	96.7812	6.08236	6
−5	15	3	94.9321	6.40903	9
−5	15	3	96.9613	7.44391	7
4	−15	−3	13.4030	4.93299	2
−4	15	3	13.5321	1.71076	9
−4	15	3	14.4795	1.36458	6
−4	15	3	13.0405	2.20788	7
4	15	−3	14.2395	3.90007	10
3	−15	−3	24.6276	5.53152	2
−3	15	3	22.3500	2.86806	3
2	−15	−3	367.621	25.8954	2
−2	15	3	353.961	22.6581	4
1	−15	−3	6.73884	3.65591	2
−1	15	3	6.84667	1.74870	4
0	−15	−3	34.1222	5.90808	2

0	15	−3	28.2553	2.96837	4
−1	−15	−3	139.799	12.2279	2
−1	15	−3	136.466	8.51574	6
−2	−15	−3	74.3871	8.15870	2
−2	15	−3	62.5270	4.37697	9
−2	15	−3	63.2852	4.01800	6
−2	15	−3	60.6576	4.66009	3
−3	−15	−3	23.1173	5.32398	2
3	15	3	16.8423	4.62632	10
−3	15	−3	29.9984	1.78282	6
−4	−15	−3	22.4242	6.00580	1
−4	15	−3	22.0154	4.00236	12
−4	15	−3	24.2129	3.51761	14
−4	15	−3	21.8771	1.51763	9
−4	15	−3	22.5508	1.58384	9
−5	−15	−3	15.2156	3.88301	10
−5	−15	−3	19.1477	5.64705	1
−5	15	−3	12.2028	3.29536	12
−6	−15	−3	119.089	10.2563	10
−6	15	−3	156.508	10.4944	12
−7	−15	−3	9.46283	3.15091	10
−7	−15	−3	8.81753	2.76314	10
−8	−16	3	2.74299	2.46596	10
−7	16	3	12.2818	0.96341	6
−7	16	3	12.4265	2.38582	7
−7	16	3	11.0639	0.93733	6
−7	16	3	11.5176	1.25546	9
−6	16	3	97.1340	6.19269	9
6	16	−3	72.2842	9.21465	10
−6	16	3	100.177	7.32022	7
−6	16	3	98.6818	5.91700	6
5	16	−3	215.410	17.5272	10
−5	16	3	248.407	15.2711	9
−5	16	3	255.214	14.9581	6
−5	16	3	251.040	16.1209	7
4	−16	−3	21.7161	5.72348	2
−4	−16	3	26.1799	4.03892	13
−4	16	3	19.3148	2.85367	7
4	16	−3	27.9519	4.86255	10
−4	16	3	19.2384	2.19194	9
3	−16	−3	201.279	16.0694	2
3	16	−3	167.115	14.1538	10
−3	16	3	203.247	12.0479	11
2	−16	−3	52.0061	7.47837	2
1	−16	−3	114.318	11.2084	2
1	16	−3	124.078	8.36145	4
−1	16	3	103.404	8.11431	4
0	−16	−3	28.9464	5.96541	2
0	16	−3	34.4233	3.09815	4
−1	−16	−3	37.3241	6.62518	2

−1	16	−3	33.0689	2.93824	9
−1	16	−3	33.8341	3.42178	3
−2	−16	−3	2.26890	3.10719	2
−2	16	−3	0.91336	0.93124	9
2	16	3	0.90215	2.97501	10
−2	16	−3	1.93539	0.45144	6
−3	−16	−3	13.8610	4.51546	2
−3	16	−3	10.6379	0.92419	6
−4	−16	−3	54.5042	8.95555	1
−4	16	−3	58.7378	3.65160	9
−4	16	−3	57.0904	3.69491	9
4	16	3	52.9209	7.42349	10
−4	16	−3	66.4989	6.21266	12
−5	−16	−3	209.050	15.3995	10
−5	−16	−3	209.361	18.7149	1
−5	16	−3	215.129	14.8418	12
−6	−16	−3	12.8239	3.32180	10
−6	−16	−3	12.3124	3.74731	10
−6	16	−3	10.3333	3.16447	12
−7	−16	−3	0.39145	2.06275	10
−8	−17	3	3.24222	2.32910	10
−7	−17	3	13.5261	3.46793	10
−7	17	3	10.6158	0.83719	6
−7	17	3	12.0304	1.10880	9
−7	17	3	11.6590	0.84594	6
6	17	−3	12.0899	4.29169	10
−6	17	3	21.3595	1.92154	9
−6	17	3	20.6118	3.27275	7
−6	17	3	20.1865	1.50443	6
−5	17	3	22.6619	2.19098	9
−5	17	3	24.7569	1.82778	6
−5	17	3	23.0584	3.38386	7
−4	−17	3	51.3432	5.49619	13
4	17	−3	49.6420	6.69449	10
−4	17	3	59.1618	4.09417	9
3	−17	−3	12.3349	4.40273	2
−3	−17	3	8.90181	2.52299	13
3	17	−3	7.91627	3.23595	10
−3	17	3	14.6419	1.75260	11
2	−17	−3	49.0128	7.82258	2
2	17	−3	67.3707	6.37007	10
1	−17	−3	88.5319	9.95648	2
0	−17	−3	4.52760	3.23719	2
−1	−17	−3	161.095	14.5255	2
1	17	3	188.798	13.3341	10
−1	17	−3	160.267	11.1409	9
−1	17	−3	176.014	10.7356	11
−2	−17	−3	8.59917	3.01408	13
−2	−17	−3	8.81716	3.82523	2
−2	17	−3	10.7217	1.48144	9

2	17	3	9.40992	3.63824	10
−2	17	−3	12.7826	1.07593	6
−2	17	−3	12.5462	3.55410	14
−2	17	−3	10.2524	3.41987	12
−3	−17	−3	18.0653	3.28342	13
−3	17	−3	14.1544	1.24310	6
−3	17	−3	14.2416	1.64231	9
−3	17	−3	15.8885	3.24067	12
−3	17	−3	15.4053	1.16959	6
3	17	3	16.4536	4.32323	10
−4	−17	−3	88.1799	10.5732	1
−4	17	−3	67.0108	4.29853	9
−4	17	−3	64.1620	4.24253	9
4	17	3	75.3667	8.26903	10
−4	17	−3	63.6586	6.50707	12
−5	−17	−3	165.841	13.5235	10
−5	17	−3	201.240	13.2171	12
−6	−17	−3	1.15826	3.10221	10
−6	−17	−3	0.94106	2.61750	10
−7	−17	−3	0.62600	1.94361	10
−7	−18	3	2.22784	1.94561	10
−7	18	3	0.81064	0.40632	9
7	18	−3	1.22610	3.50394	10
−7	18	3	1.03760	0.37899	9
−6	18	3	3.26315	0.84280	9
−6	18	3	3.97510	0.55358	6
6	18	−3	13.4233	4.43446	10
−5	18	3	139.248	9.16752	6
5	18	−3	157.257	12.3462	10
−5	18	3	150.800	9.52340	9
−4	−18	3	62.3732	6.50731	13
−4	18	3	63.0198	4.58794	9
4	18	−3	62.8426	7.22897	10
−3	−18	3	21.6528	4.05349	13
3	18	−3	19.1234	3.94879	10
−3	18	3	16.5693	1.73997	11
−3	18	3	16.8308	1.65046	11
2	−18	−3	201.262	16.9174	2
−2	−18	3	189.732	13.3797	13
2	18	−3	196.383	14.1123	10
−2	18	3	182.575	12.3870	11
1	−18	−3	2.54105	3.43684	2
−1	18	3	2.86565	2.01162	10
0	−18	−3	0.28483	2.92618	2
0	18	3	−1.6581	2.41963	10
0	18	−3	3.16302	1.63765	11
−1	−18	−3	17.6193	4.81106	2
−1	−18	−3	20.5254	3.51079	13
−1	18	−3	19.8020	1.59847	11
−1	18	−3	17.2881	1.53656	11

1	18	3	18.6508	3.84526	10
−2	−18	−3	12.0530	3.21068	13
−2	18	−3	16.8850	3.34849	12
−2	18	−3	17.0739	2.09207	9
2	18	3	8.65740	3.80476	10
−2	18	−3	14.5374	1.26710	6
−3	−18	−3	2.85402	2.75135	13
−3	18	−3	15.2426	3.23143	12
−3	18	−3	7.97449	0.81514	6
−3	18	−3	8.29723	1.17675	9
−3	18	−3	8.35421	0.81141	6
3	18	3	1.35367	3.42839	10
−4	−18	−3	48.6600	9.02757	1
−4	18	−3	56.8541	3.43563	9
4	18	3	39.3394	7.12743	10
−4	18	−3	57.8171	3.48398	9
−4	18	−3	54.0666	6.01438	12
−5	−18	−3	0.03228	2.86663	10
−5	−18	−3	1.27643	2.29422	10
−5	18	−3	−1.0102	3.07887	12
5	18	3	0.55796	2.60770	10
−6	−18	−3	35.7049	4.36706	10
−6	−18	−3	41.3035	4.39211	10
−7	−18	−3	2.06113	1.95776	10
7	19	−3	4.47864	2.47161	10
−6	19	3	20.9040	1.45541	6
−6	19	3	19.6882	1.44386	6
−6	19	3	20.0624	1.74447	9
6	19	−3	21.0627	4.24828	10
5	19	−3	164.838	12.1123	10
−5	19	3	150.109	9.49002	6
−5	19	3	148.036	9.76371	9
−4	−19	3	54.8063	6.09549	13
4	19	−3	53.0332	6.44127	10
−4	19	3	35.2296	3.46456	9
−3	−19	3	24.5055	4.29779	13
3	19	−3	22.3146	4.23249	10
−2	−19	3	26.0064	3.92739	13
−2	19	3	26.6403	3.04134	11
2	19	−3	25.2270	4.14691	10
−1	−19	3	0.98582	2.62026	13
1	−19	−3	2.05296	2.49267	13
−1	19	3	0.64722	2.27253	10
1	19	−3	4.05480	2.76681	10
0	−19	−3	7.75561	2.86034	13
0	19	−3	5.47334	1.82606	11
−1	−19	−3	71.6038	6.67880	13
1	19	3	84.0902	7.73900	10
−2	−19	−3	121.572	9.60432	13
−2	19	−3	123.259	8.27813	9

−2	19	−3	131.931	7.96582	6
−2	19	−3	141.859	9.81370	12
2	19	3	122.424	10.7619	10
−3	−19	−3	89.2606	7.84026	13
−3	19	−3	93.8040	5.66805	6
−3	19	−3	85.5249	5.69330	6
−3	19	−3	95.2032	7.85027	12
3	19	3	98.8041	9.09484	10
−3	19	−3	88.4070	5.94200	9
−4	19	−3	6.19046	3.33430	12
4	19	3	3.83317	4.00757	10
5	19	3	26.7917	4.69254	10
−6	−19	−3	12.4818	2.86298	10
7	20	−3	6.09869	3.05326	10
6	20	−3	1.45542	3.40821	10
−6	20	3	3.41060	0.65325	9
−6	20	3	2.51579	0.35361	6
−6	20	3	2.08493	0.34776	6
−5	20	3	20.4920	2.12425	9
−5	20	3	21.3825	1.60853	6
−4	−20	3	69.0895	7.23070	13
−4	20	3	79.8234	5.06886	9
4	20	−3	54.9443	7.49942	10
−3	−20	3	12.8694	3.42358	13
3	20	−3	14.1823	3.69258	10
−2	−20	3	83.0197	7.51252	13
−2	20	3	90.9734	6.06318	11
2	20	−3	84.8860	7.78890	10
1	−20	−3	54.8732	5.22704	13
−1	−20	3	40.0680	5.78583	13
1	20	−3	45.6768	5.35726	10
−1	20	3	48.2205	5.11916	10
0	−20	−3	63.7149	5.88776	13
0	20	3	61.1288	6.18490	10
0	20	−3	50.8097	4.52347	11
−1	−20	−3	84.1520	7.17293	13
1	20	3	83.6340	7.92531	10
−2	−20	−3	49.2147	5.76555	13
−2	20	−3	59.9169	3.79045	6
2	20	3	48.7693	6.70262	10
−2	20	−3	63.4635	4.33889	9
−2	20	−3	61.4170	3.80083	6
−3	20	−3	52.5306	3.46150	6
−3	20	−3	49.0156	3.39054	6
−3	20	−3	59.6916	3.82349	9
4	20	3	3.10491	2.70335	10
5	20	3	36.2935	6.00408	10
−6	−20	−3	0.40214	1.94898	10
6	21	−3	0.07684	2.23417	10
−6	21	3	0.16787	0.43173	9

-6	21	3	0.70151	0.37871	9
-5	21	3	8.04037	1.19000	9
-5	21	3	7.10960	0.75328	6
-4	-21	3	0.28491	2.55098	13
-4	21	3	-0.8051	1.12682	9
4	21	-3	2.63621	3.39206	10
-3	-21	3	14.6554	3.23724	13
-2	-21	3	0.10382	2.27241	13
2	21	-3	0.58520	2.47981	10
-2	21	3	4.36630	1.19457	11
-2	21	3	4.40440	1.18777	11
1	-21	-3	4.74977	2.65526	13
-1	-21	3	5.86573	2.45565	13
-1	21	3	3.72223	1.65226	11
1	21	-3	4.70496	2.43755	11
0	-21	-3	151.144	11.4547	13
0	21	3	158.310	11.9289	10
0	21	-3	160.446	10.3137	11
0	21	-3	169.751	10.2854	11
-1	-21	-3	246.266	16.5544	13
1	21	3	233.477	16.8983	10
-2	-21	-3	7.17722	2.92279	13
-2	21	-3	5.72816	0.80568	6
-2	21	-3	7.39247	1.52577	9
2	21	3	10.4019	3.72799	10
-3	-21	-3	1.24314	2.97655	13
-3	21	-3	1.19495	0.41470	6
3	21	3	0.10277	3.31001	10
-3	21	-3	0.93964	0.44713	6
-3	21	-3	1.03973	0.80745	9
4	21	3	11.7667	4.21042	10
5	21	3	20.1205	4.05028	10
6	22	-3	10.2513	2.53890	10
5	22	-3	17.4383	3.29462	10
-5	22	3	14.7055	1.21351	6
-5	22	3	18.1947	1.65420	9
-4	-22	3	22.6900	3.46404	13
-4	22	3	14.6801	1.39897	6
4	22	-3	20.2434	3.94190	10
-4	22	3	19.8550	2.21881	9
-3	-22	3	3.68021	2.45620	13
3	22	-3	2.76098	2.84004	10
-2	-22	3	-1.5702	2.35299	13
2	22	-3	1.29935	2.20391	10
-2	22	3	-0.1734	0.66235	11
-2	22	3	0.34643	0.79411	11
1	-22	-3	3.95281	2.47624	13
-1	-22	3	5.19665	2.46601	13
-1	22	3	3.95730	1.89244	11
0	-22	-3	31.6172	4.68851	13

0	22	-3	25.9630	2.24733	11
0	22	-3	21.2411	2.11201	11
-1	-22	-3	8.17550	2.79308	13
1	22	3	6.41644	2.96721	10
-2	-22	-3	9.94554	2.84071	13
-2	22	-3	4.64573	1.21737	9
-2	22	-3	5.36200	0.76798	6
2	22	3	11.1340	2.90507	10
-3	-22	-3	3.38420	2.42623	13
3	22	3	4.80956	3.31182	10
-3	22	-3	5.63237	0.96931	9
-3	22	-3	6.34683	0.59972	6
-3	22	-3	5.70721	0.63116	6
4	22	3	-2.3587	2.26182	10
5	22	3	3.92204	2.95271	10
6	23	-3	0.10693	2.44141	10
5	23	-3	2.27581	2.68821	10
-4	-23	3	20.4537	3.84989	13
-4	23	3	16.4440	1.40837	6
-4	23	3	15.6847	1.87142	9
4	23	-3	15.7882	2.95247	10
-3	-23	3	46.6847	5.34210	13
3	23	-3	44.4695	5.20971	10
2	-23	-3	5.37572	2.52168	13
1	-23	-3	6.90504	2.43683	13
-1	23	3	6.55164	1.50513	11
1	23	-3	3.82302	1.56452	11
0	-23	-3	19.5604	3.36152	13
0	23	3	21.8183	3.29676	10
-1	-23	-3	21.2105	3.54346	13
-2	-23	-3	20.1772	3.28337	13
2	23	3	12.8286	3.42072	10
-2	23	-3	14.5368	1.98156	9
-2	23	-3	15.0673	1.31873	6
-3	-23	-3	15.9382	2.97581	13
-3	23	-3	7.13677	1.05647	9
-3	23	-3	9.51078	0.79724	6
-3	23	-3	10.8913	0.81351	6
3	23	3	13.9950	3.60283	10
4	23	3	23.6193	4.60862	10
5	23	3	1.81770	2.64334	10
5	24	-3	11.6122	2.55653	10
-4	-24	3	4.50669	2.02999	13
-4	24	3	5.95202	1.12734	9
4	24	-3	6.26208	2.37865	10
-3	-24	3	7.36358	2.51749	13
3	24	-3	6.38108	2.57148	10
1	-24	-3	16.2070	3.37368	13
-1	-24	3	18.9058	3.01513	13
1	24	-3	20.0907	2.21029	11

-1	24	3	15.8990	1.88897	11
-1	24	3	14.2021	2.03715	11
1	24	-3	22.2652	2.21449	11
0	-24	-3	1.75693	2.42315	13
0	-24	3	0.38436	2.34900	13
0	24	3	1.52642	1.59251	11
-1	-24	-3	4.29379	2.57957	13
1	24	3	0.98763	2.05193	10
-2	-24	-3	13.0868	2.78584	13
2	24	3	12.6052	3.12233	10
-2	24	-3	16.3014	1.82358	9
-2	24	-3	15.1050	1.25063	6
-3	-24	-3	23.6979	3.78318	13
-3	24	-3	27.7789	1.90994	9
3	24	3	25.2579	4.38682	10
-3	24	-3	28.5902	1.98418	9
4	24	3	3.41415	2.48535	10
-4	-25	3	16.6823	2.72507	13
4	25	-3	14.4450	2.55029	10
-3	-25	3	3.36850	2.03363	13
3	25	-3	2.27683	1.45842	11
2	-25	-3	32.9577	4.16022	13
-2	-25	3	21.5310	4.09791	13
2	25	-3	23.0666	2.69184	11
1	-25	-3	1.27821	1.97651	13
-1	-25	3	2.17722	2.09574	13
0	-25	-3	-2.0380	2.49171	13
0	-25	3	0.61036	1.81053	13
0	25	3	-0.8351	1.23288	11
-1	-25	-3	0.13556	2.06668	13
1	25	3	0.70028	1.54253	10
1	25	3	1.06644	1.23328	11
-2	-25	-3	22.4528	3.14204	13
-2	25	-3	18.5219	1.39571	6
2	25	3	20.7779	3.35848	10
-2	25	-3	16.7065	1.88782	9
3	25	3	6.82358	2.37114	10
-3	-26	3	7.86247	1.89445	13
3	26	-3	6.10347	1.27888	11
2	-26	-3	24.2227	3.13169	13
-2	-26	3	30.5390	3.76666	13
-1	-26	3	3.45985	1.97512	13
1	-26	-3	7.05627	1.98960	13
0	-26	3	7.18856	2.35446	13
0	-26	-3	7.53675	2.12582	13
0	26	3	7.62476	1.13460	11
0	26	3	7.86633	1.15729	11
-1	-26	-3	0.47591	1.77263	13
1	26	3	0.28956	1.08994	11
-1	26	-3	-0.0114	1.31775	9

-2	-26	-3	0.68770	1.48015	13
2	26	3	-0.1272	1.58519	10
2	-27	-3	2.62566	1.35961	13
-2	-27	3	0.98426	1.52054	13
-1	-27	3	6.57850	1.84288	13
1	-27	-3	9.97193	1.83743	13
0	-27	-3	0.97731	1.51478	13
0	-27	3	1.76360	1.75257	13
-1	-27	-3	5.44171	1.69207	13
1	27	3	5.54516	0.76242	11
1	27	3	7.10468	1.00372	11
10	0	-4	4.61674	2.87899	13
-10	0	4	1.26376	1.26544	8
9	0	-4	4.01960	3.17254	13
-9	0	4	0.00126	1.52482	7
-8	0	4	14.7769	2.67125	10
8	0	-4	15.6751	4.38889	13
-7	0	4	35.7627	4.15553	10
-6	0	4	7.00196	2.90754	1
-5	0	4	22.1333	4.16942	1
-4	0	4	281.003	19.4268	1
-4	0	4	288.227	19.1473	5
-3	0	4	78.6002	6.12603	4
-3	0	4	85.4945	7.81917	5
-2	0	4	8172.91	492.424	5
-2	0	4	7930.68	488.461	4
1	0	-4	1256.64	77.8074	4
-1	0	4	1294.06	79.0615	5
0	0	-4	3402.40	199.942	5
0	0	-4	3219.51	197.837	4
0	0	4	3246.70	199.160	5
0	0	-4	3316.19	197.986	4
0	0	-4	3047.63	198.689	5
0	0	-4	3362.47	197.927	3
-1	0	-4	7.97961	1.74166	2
-1	0	-4	7.19968	1.77964	5
-1	0	-4	8.25483	1.43517	3
-1	0	-4	7.48778	1.56328	4
-1	0	-4	5.92450	1.20008	4
-1	0	-4	6.45487	1.40198	5
-2	0	-4	1774.85	110.338	4
-2	0	-4	1719.89	110.474	4
-2	0	-4	1847.75	111.165	2
-2	0	-4	1920.80	110.512	3
-3	0	-4	0.73902	0.98222	3
-3	0	-4	1.13633	1.63180	2
-3	0	-4	1.38353	1.25825	4
-3	0	-4	0.91649	1.38450	1
-4	0	-4	357.715	23.0769	3
-4	0	-4	364.197	22.9710	4

-4	0	-4	365.149	24.6449	2
-5	0	-4	4.17326	1.44168	8
-6	0	-4	116.178	7.89530	8
-7	0	-4	3.60099	1.51110	8
-8	0	-4	59.7513	4.26807	8
9	0	4	4.05456	2.59297	13
10	-1	-4	11.8685	3.05510	13
-10	-1	4	9.18482	1.56927	8
10	1	-4	11.2049	3.14978	13
9	-1	-4	4.70874	3.11705	13
9	-1	-4	4.23949	3.20413	13
-9	1	4	6.65435	1.57787	7
9	1	-4	9.50906	3.55077	13
8	-1	-4	7.89782	3.33797	13
-8	1	4	10.6746	1.72243	11
-7	-1	4	33.6103	4.41453	10
-7	1	4	26.0318	3.61035	10
-6	-1	4	26.4760	4.51490	1
-6	1	4	20.6635	3.71628	1
-6	1	4	22.5767	3.85144	4
-5	-1	4	8.83799	2.20028	5
-5	-1	4	9.52839	3.26670	1
-5	1	4	12.4160	2.22769	4
-5	1	4	13.4441	2.99548	1
-4	-1	4	366.792	25.3136	1
-4	-1	4	374.591	25.1986	5
-4	1	4	390.261	24.1930	5
-4	1	4	372.815	24.8354	1
-4	1	4	381.953	23.8003	4
-3	-1	4	994.395	63.2802	5
-3	1	4	1028.26	61.6925	4
-3	1	4	1000.39	62.7501	5
-2	-1	4	1397.88	83.5844	5
-2	1	4	1332.71	81.9397	4
-2	1	4	1242.95	82.0803	3
-2	1	4	1407.68	83.2914	5
1	-1	-4	9462.66	564.000	2
-1	-1	4	8485.76	565.236	5
-1	1	4	9999.99	568.688	5
1	1	-4	9115.10	560.345	4
0	-1	-4	3027.43	192.205	4
0	-1	-4	2899.56	192.110	3
0	-1	-4	3061.87	192.686	5
0	-1	-4	2957.68	192.321	2
0	1	-4	3414.27	192.141	3
0	1	-4	3401.86	194.485	5
0	1	-4	3410.67	192.196	4
0	1	4	2899.84	193.294	5
0	1	-4	3302.01	192.902	5
0	1	-4	3319.92	192.074	4

−1	−1	−4	211.874	12.7612	5
−1	−1	−4	207.265	13.1467	5
−1	−1	−4	217.271	12.4271	3
−1	−1	−4	203.249	12.7548	2
−1	−1	−4	210.610	12.5158	4
−1	1	−4	168.902	12.2062	4
−1	1	−4	185.081	11.9558	5
−1	1	−4	180.039	12.1866	3
−1	1	−4	172.234	12.0936	4
−1	1	−4	179.840	11.8879	5
−1	1	−4	170.711	12.0402	3
−2	−1	−4	243.389	15.7597	3
−2	−1	−4	248.918	16.5178	2
−2	−1	−4	254.715	15.4879	5
−2	−1	−4	268.551	15.6213	5
−2	−1	−4	260.715	15.8922	4
−2	1	−4	238.966	15.5979	3
−2	1	−4	240.713	15.6088	4
−2	1	−4	240.952	16.2379	2
−2	1	−4	217.706	15.4308	4
−3	−1	−4	531.452	33.0946	2
−3	−1	−4	493.599	32.3778	1
−3	−1	−4	510.993	31.8683	3
−3	−1	−4	532.821	31.9898	4
−3	1	−4	531.190	31.6714	4
−3	1	−4	508.846	32.7615	2
−3	1	−4	474.884	31.4133	4
−3	1	−4	500.800	31.6781	3
−4	−1	−4	505.743	32.2120	2
−4	−1	−4	508.491	30.6316	4
−4	1	−4	480.781	30.0150	4
−4	1	−4	469.529	30.1356	4
−4	1	−4	464.553	30.3821	3
−5	−1	−4	13.4983	1.98223	8
−5	−1	−4	11.0515	2.11747	4
−5	1	−4	12.5540	1.51402	4
−5	1	−4	12.3364	1.80191	8
−6	−1	−4	93.3134	6.84338	8
−6	1	−4	97.5692	6.67022	8
−7	−1	−4	9.34612	1.86488	8
−7	1	−4	4.39938	1.40075	8
−8	−1	−4	5.00720	1.36099	8
8	1	4	4.90678	3.05469	13
9	1	4	7.80879	2.82216	13
10	−2	−4	14.2655	3.09988	13
−10	−2	4	12.3211	1.87115	8
10	2	−4	11.3120	3.27398	13
9	−2	−4	3.68937	2.95648	13
9	−2	−4	1.35800	2.93609	13
9	2	−4	−0.9183	3.15570	13

−9	2	4	0.33885	1.10755	7
−8	−2	4	92.5276	7.41942	10
8	−2	−4	90.2148	8.80757	13
−8	2	4	85.4512	6.02982	11
8	2	−4	90.5581	9.02860	13
8	2	−4	86.3897	9.37489	13
−7	−2	4	37.0508	4.44819	10
−7	−2	4	36.5830	5.36834	1
−7	2	4	37.5400	4.14612	10
7	2	−4	31.2107	5.88700	13
−6	−2	4	314.937	21.7904	1
−6	2	4	302.657	19.9539	4
−5	−2	4	166.284	11.9997	5
−5	−2	4	170.370	12.9336	1
−5	2	4	152.515	11.7882	1
−5	2	4	165.706	11.0876	4
−4	−2	4	401.314	24.7575	1
−4	−2	4	423.329	25.1605	5
−4	2	4	323.720	22.8035	4
−4	2	4	311.456	23.6640	1
−3	−2	4	375.074	25.9868	5
−3	2	4	395.659	25.0748	5
−3	2	4	393.231	24.2177	4
−3	2	4	387.139	24.1723	3
−2	−2	4	141.688	10.0109	5
−2	2	4	112.405	9.13491	5
−2	2	4	115.363	7.93074	3
−2	2	4	116.968	8.09280	4
1	−2	−4	182.639	12.4826	2
−1	2	4	194.747	12.1052	4
−1	2	4	189.015	13.3986	5
0	−2	−4	846.623	50.6430	5
0	−2	−4	881.100	50.0352	4
0	−2	−4	884.944	50.2887	2
0	2	−4	795.637	49.9860	5
0	2	−4	739.352	49.6542	4
0	2	−4	806.672	49.7474	3
0	2	−4	747.453	49.6216	3
0	2	4	873.649	51.2872	5
0	2	−4	776.882	50.1857	5
0	2	−4	789.467	49.8082	4
−1	−2	−4	636.746	42.3693	2
−1	−2	−4	642.620	41.9287	3
−1	−2	−4	666.132	42.4672	5
−1	−2	−4	611.618	42.0158	4
−1	2	−4	713.857	41.7194	4
−1	2	−4	735.778	41.8282	4
−1	2	−4	721.070	41.8030	3
−1	2	−4	723.294	41.7151	3
−2	−2	−4	45.1691	4.43104	5

−2	−2	−4	39.1989	4.62291	1
−2	−2	−4	43.9356	4.31108	4
−2	−2	−4	42.6726	3.89060	3
−2	−2	−4	45.3138	4.69569	2
−2	−2	−4	48.2037	4.28251	5
−2	2	−4	60.2839	3.84859	4
−2	2	−4	61.5662	3.80869	3
−3	−2	−4	374.485	24.9887	2
−3	−2	−4	350.964	24.5590	1
−3	−2	−4	363.628	23.9500	4
−3	2	−4	401.482	23.6252	3
−3	2	−4	379.974	23.2926	4
−3	2	−4	387.885	23.4449	4
−4	−2	−4	89.0382	7.56010	4
−4	−2	−4	89.4587	7.82421	1
−4	−2	−4	91.1167	8.75075	2
−4	2	−4	120.929	7.37983	3
−4	2	−4	101.683	6.65375	4
−4	2	−4	112.539	6.86123	4
−5	−2	−4	7.26703	1.48255	8
−5	−2	−4	9.66800	3.60080	2
−5	−2	−4	5.08651	1.85053	4
−5	2	−4	3.16034	1.22204	8
−5	2	−4	2.74299	1.25837	3
−6	−2	−4	2.72233	1.37751	8
−6	2	−4	1.14797	1.12785	8
−7	−2	−4	177.309	11.1335	8
−7	2	−4	159.236	10.7477	8
7	2	4	167.110	13.2332	13
−8	−2	−4	20.2256	2.52087	8
8	2	4	19.5763	4.47364	13
9	2	4	−0.1148	2.14872	13
10	−3	−4	−0.8379	2.31565	13
−10	−3	4	1.51374	1.42931	8
10	3	−4	0.73745	2.86608	13
9	−3	−4	9.96783	3.83391	13
−9	−3	4	7.87258	1.97902	10
9	−3	−4	13.0014	3.29577	13
−9	3	4	13.5436	2.00052	7
9	3	−4	9.40365	3.71467	13
−8	−3	4	87.6502	7.39319	10
8	3	−4	84.2642	9.00665	13
8	3	−4	82.7167	9.42930	13
−8	3	4	87.0127	5.80648	11
−7	−3	4	16.3489	3.91487	1
−7	−3	4	19.1190	3.32715	10
7	3	−4	26.6266	4.89926	13
−7	3	4	18.8689	2.35316	11
−6	−3	4	367.398	24.3240	1
−6	3	4	317.771	21.7466	4

−5	−3	4	8.48745	2.85610	5
−5	−3	4	10.9670	3.63968	1
−4	−3	4	226.020	15.4497	1
−4	−3	4	241.401	16.0605	5
−4	3	4	178.092	14.0731	1
−4	3	4	186.415	13.3526	4
−3	−3	4	74.1244	7.69394	5
−3	3	4	89.4007	7.56450	1
−3	3	4	84.4592	5.77768	3
−3	3	4	81.4593	6.25527	5
−3	3	4	75.2009	5.69951	4
2	−3	−4	138.155	9.93863	2
−2	−3	4	140.802	10.9772	5
−2	3	4	138.396	9.09755	3
−2	3	4	137.729	9.15106	4
−2	3	4	141.316	10.2776	5
1	−3	−4	5267.21	323.859	2
1	3	−4	5542.86	322.542	4
−1	3	4	5209.68	322.454	4
−1	3	4	5287.63	323.573	5
0	−3	−4	2355.16	147.538	4
0	−3	−4	2669.45	148.062	5
0	−3	−4	2585.22	147.650	2
0	3	−4	2317.16	147.083	5
0	3	−4	2400.48	146.885	4
0	3	−4	2279.98	146.940	4
0	3	−4	2299.41	146.829	3
0	3	−4	2311.83	146.930	3
0	3	−4	2477.90	147.975	5
0	3	4	2534.13	148.397	5
−1	−3	−4	1972.83	118.644	5
−1	−3	−4	1833.46	118.035	4
−1	−3	−4	1815.40	118.425	2
−1	3	−4	1995.59	117.666	4
−1	3	−4	1936.74	117.577	4
−1	3	−4	2039.67	117.678	3
−1	3	−4	1972.33	117.573	3
−2	−3	−4	64.5322	5.89565	5
−2	−3	−4	70.0944	6.24706	2
−2	−3	−4	65.8167	5.44510	4
−2	−3	−4	64.4240	6.30368	1
−2	3	−4	73.0279	4.73627	4
−2	3	−4	65.8268	4.77905	3
−2	3	−4	73.7658	4.66405	4
−2	3	−4	63.6485	4.66632	3
−3	−3	−4	19.8632	3.72495	1
−3	−3	−4	20.5993	2.31698	5
−3	−3	−4	18.8785	2.83960	4
−3	−3	−4	19.2195	2.41157	5
−3	−3	−4	21.3908	3.66983	2

−3	3	−4	27.4284	2.34231	3
−4	−3	−4	8.52919	3.27435	2
−4	−3	−4	6.76088	2.60473	1
−4	3	−4	6.19934	1.40043	3
−5	−3	−4	4.44469	3.38436	2
−5	−3	−4	4.04305	2.24521	4
−5	−3	−4	5.77406	1.73618	8
−5	3	−4	7.18351	1.56479	3
−5	3	−4	9.48129	1.16509	9
−5	3	−4	7.39586	1.39179	8
−6	−3	−4	103.009	7.41605	10
−6	−3	−4	101.056	7.22421	8
−6	3	−4	97.4358	6.49423	9
−6	3	−4	96.6328	6.67894	8
−6	3	−4	91.8282	6.45831	9
6	3	4	104.891	9.00702	13
−7	−3	−4	103.197	6.99184	8
7	3	4	100.410	9.68126	13
−7	3	−4	86.7644	6.53852	9
−8	−3	−4	16.6722	2.61491	8
8	3	4	18.8777	4.54579	13
9	3	4	3.27848	2.72350	13
10	−4	−4	17.0819	3.01039	13
10	4	−4	18.1661	3.46830	13
−9	−4	4	7.60403	2.40150	10
9	−4	−4	10.6095	3.25565	13
9	4	−4	7.98958	3.75937	13
−9	4	4	10.2036	2.13316	7
−8	4	4	23.9647	3.45043	7
−8	4	4	25.6002	2.09310	11
8	4	−4	14.3960	4.06507	13
−7	−4	4	15.8178	3.35500	10
−7	−4	4	12.7011	3.67918	1
7	4	−4	19.0852	4.88020	13
−7	4	4	19.9952	2.03139	11
−6	−4	4	117.388	11.0762	1
−6	4	4	131.448	8.74907	4
−5	−4	4	123.626	10.7336	5
−5	−4	4	123.206	10.7586	1
−5	4	4	123.800	8.54417	4
−4	−4	4	725.706	48.7736	1
−4	−4	4	732.105	49.2161	5
−4	4	4	786.021	46.8967	3
−4	4	4	798.542	46.8782	4
−3	−4	4	0.55724	3.01088	5
−3	4	4	5.24615	1.33544	5
−3	4	4	5.95549	1.18510	3
−3	4	4	4.78455	1.46108	4
2	−4	−4	57.8771	5.30759	2
−2	4	4	60.0211	4.35484	4

−2	4	4	57.2854	4.30111	3
−2	4	4	57.8378	5.07601	5
1	−4	−4	573.213	35.6694	2
1	4	−4	554.450	34.9707	4
−1	4	4	570.968	35.9849	5
−1	4	4	579.265	34.9557	4
1	4	−4	556.242	35.7040	5
0	−4	−4	1911.43	129.003	2
0	−4	−4	2010.33	129.360	5
0	4	−4	2118.56	128.014	5
0	4	−4	2183.50	128.273	4
0	4	−4	2328.00	128.392	4
0	4	−4	2214.33	128.090	5
0	4	−4	2175.81	128.362	3
0	4	−4	2136.68	128.259	3
0	4	4	1952.37	129.581	5
−1	−4	−4	260.421	17.5767	2
−1	−4	−4	268.300	17.7423	5
−1	4	−4	269.500	16.4053	3
−1	4	−4	266.524	16.5005	3
−1	4	−4	266.799	16.4058	4
−1	4	−4	250.541	16.4144	4
−2	−4	−4	485.979	32.8456	2
−2	−4	−4	502.112	32.8248	5
−2	−4	−4	444.793	32.8145	1
−2	4	−4	541.682	31.5752	3
−2	4	−4	515.841	31.3023	4
−2	4	−4	556.464	31.3937	4
−2	4	−4	533.380	31.4864	3
−3	−4	−4	275.342	19.1995	5
−3	−4	−4	277.706	19.9350	1
−3	−4	−4	282.702	20.1088	2
−3	4	−4	297.770	18.3998	3
−3	4	−4	301.043	18.3248	3
−3	4	−4	294.819	17.8291	4
−3	4	−4	315.631	17.8644	4
−4	−4	−4	83.1550	8.03122	2
−4	−4	−4	80.7268	7.30774	1
−4	4	−4	64.2170	5.33497	3
−5	−4	−4	89.8092	7.51391	8
−5	−4	−4	91.7330	8.65126	1
−5	−4	−4	94.1540	9.90361	2
−5	4	−4	117.093	7.03365	8
−5	4	−4	116.350	6.71138	6
−5	4	−4	112.980	6.83272	9
−5	4	−4	116.712	7.72734	3
−6	−4	−4	37.8474	4.00372	8
−6	−4	−4	37.6300	5.01099	10
−6	−4	−4	37.8909	4.34296	10
−6	4	−4	55.3146	3.38815	9

−6	4	−4	55.8849	3.13121	6
−7	−4	−4	198.774	12.7336	8
−7	4	−4	192.992	11.8725	6
−7	4	−4	184.348	12.0678	9
7	4	4	192.647	14.3763	13
−8	−4	−4	38.5171	3.80691	8
−8	4	−4	43.6880	2.85653	6
8	4	4	38.2170	6.20088	13
−8	4	−4	46.2092	3.28987	9
9	4	4	0.16711	2.71814	13
10	5	−4	10.2369	3.09972	13
−9	−5	4	1.66700	1.88261	10
9	5	−4	2.34392	3.52048	13
−9	5	4	4.45604	1.80828	7
−8	−5	4	100.519	7.79642	10
8	5	−4	99.3909	9.19564	13
−8	5	4	79.4866	7.00446	7
−7	−5	4	51.8328	7.42425	1
−7	−5	4	53.4949	5.59535	10
7	5	−4	47.9778	7.38741	13
−7	5	4	51.3916	3.71716	11
7	5	−4	44.9071	6.43313	13
−6	−5	4	119.779	11.5118	1
−5	−5	4	243.679	17.8090	1
−5	−5	4	235.454	17.7213	5
−5	5	4	226.031	15.0925	4
−4	−5	4	2067.92	132.174	5
−4	−5	4	2055.17	131.668	1
−4	5	4	2263.79	129.598	3
−3	5	4	111.316	7.46633	4
−3	5	4	106.547	7.35894	3
2	−5	−4	468.922	30.6763	2
−2	5	4	479.211	29.6681	4
−2	5	4	477.698	30.2077	5
−2	5	4	490.312	29.6696	3
1	−5	−4	92.6618	7.42778	2
−1	5	4	101.394	6.59858	4
−1	5	4	89.8775	7.55758	5
0	−5	−4	631.394	39.8115	5
0	−5	−4	617.331	39.5301	2
0	5	−4	629.909	38.4697	4
0	5	4	636.918	40.0347	5
0	5	−4	612.177	38.4664	3
0	5	−4	614.421	38.5175	4
0	5	−4	651.117	38.5675	3
−1	−5	−4	35.3307	4.51397	2
−1	−5	−4	34.8463	4.51112	5
−1	5	−4	39.7232	2.84039	3
−1	5	−4	35.0812	2.88216	3
−1	5	−4	43.7397	2.92558	4

−1	5	−4	34.1384	2.79284	4
−2	−5	−4	237.359	15.9175	5
−2	−5	−4	234.038	15.8558	2
−2	−5	−4	217.416	15.9624	1
−2	5	−4	213.912	14.0520	3
−2	5	−4	228.867	13.8390	4
−2	5	−4	227.150	14.1492	3
−2	5	−4	217.859	13.7994	4
−3	−5	−4	349.645	23.7769	1
−3	−5	−4	361.101	23.3531	5
−3	−5	−4	356.155	23.9229	2
−3	5	−4	354.626	21.8640	3
−3	5	−4	335.166	21.7562	3
−4	−5	−4	90.2361	8.82914	2
−4	−5	−4	95.0860	8.74565	1
−4	5	−4	97.6069	6.60213	3
−5	−5	−4	146.000	12.6487	2
−5	−5	−4	145.733	11.6698	1
−5	−5	−4	143.133	9.86353	8
−5	5	−4	142.165	8.88619	6
−5	5	−4	145.633	9.72146	3
−5	5	−4	139.823	8.97698	9
−6	−5	−4	3.35721	2.28191	10
−6	−5	−4	2.52510	1.87098	10
−6	−5	−4	2.55407	1.64258	8
−6	5	−4	5.22452	0.89415	9
−6	5	−4	2.48045	0.58414	6
6	5	4	3.42730	3.34511	13
−6	5	−4	3.47925	0.93580	9
−7	−5	−4	40.4890	4.69001	10
−7	−5	−4	41.1877	4.38545	8
−7	5	−4	50.1900	3.17315	6
−7	5	−4	49.1818	3.55719	9
7	5	4	63.8766	7.44315	13
−8	−5	−4	10.4827	1.87121	8
8	5	4	13.5005	3.71459	13
−8	5	−4	10.3646	1.03423	6
−8	5	−4	11.1654	1.36196	9
9	5	4	7.76342	2.51245	13
10	6	−4	6.10055	2.80404	13
−9	6	4	10.9188	2.00858	7
−8	−6	4	286.867	19.7340	10
−8	6	4	296.378	19.1010	7
8	6	−4	300.412	20.4406	13
−7	−6	4	14.0231	4.92046	1
−7	−6	4	18.9317	3.90478	10
−7	6	4	17.6715	1.88197	11
7	6	−4	14.2358	4.70149	13
−6	−6	4	259.299	19.7810	1
−5	−6	4	212.245	15.7885	5

−5	−6	4	191.300	15.1503	1
−5	6	4	170.248	12.8688	3
−5	6	4	178.081	12.1803	4
−4	−6	4	165.869	13.0400	1
−4	−6	4	161.942	13.7404	5
−4	6	4	152.983	10.3485	4
−4	6	4	150.807	10.4203	3
3	−6	−4	91.3594	7.81787	2
−3	−6	4	83.2181	8.33465	1
−3	6	4	89.8100	6.23731	4
−3	6	4	88.0737	6.20675	3
2	−6	−4	239.029	16.4555	2
−2	6	4	242.725	15.4992	5
−2	6	4	238.587	15.1989	3
−2	6	4	233.554	15.1861	4
1	−6	−4	277.207	19.5854	2
1	6	−4	310.514	18.5990	4
−1	6	4	293.672	18.5992	4
−1	6	4	293.779	19.5262	5
1	6	−4	296.144	19.0039	5
0	−6	−4	601.706	36.3510	2
0	6	4	591.643	36.6257	5
0	6	−4	562.676	35.0588	4
0	6	−4	563.913	35.0843	3
0	6	−4	536.675	34.9956	4
−1	−6	−4	454.197	28.0644	2
−1	−6	−4	435.215	28.2368	5
−1	6	−4	418.501	26.5517	3
−1	6	−4	413.571	26.4627	3
−1	6	−4	420.462	26.3863	4
−1	6	−4	443.758	26.4189	4
−2	−6	−4	30.0492	4.26692	5
−2	−6	−4	33.8297	4.79062	2
−2	−6	−4	33.0445	4.64536	1
−2	6	−4	32.8681	2.57323	3
−2	6	−4	32.7743	2.48211	3
−3	−6	−4	185.087	14.0707	2
−3	−6	−4	182.577	13.6779	5
−3	−6	−4	184.320	14.0584	1
−3	6	−4	177.686	11.6718	3
−3	6	−4	195.558	11.8099	3
−4	−6	−4	130.719	9.92188	5
−4	−6	−4	135.900	11.8823	2
−4	−6	−4	136.328	11.5651	1
−4	6	−4	150.952	8.74954	6
−4	6	−4	149.586	9.34281	3
−5	−6	−4	5.40433	1.69231	8
−5	−6	−4	7.00987	3.45922	2
−5	−6	−4	2.76987	2.91976	10
−5	−6	−4	5.66377	3.03202	1

−5	6	−4	0.72407	0.46735	6
−5	6	−4	0.99433	0.65779	9
−5	6	−4	1.26719	0.66224	9
−6	−6	−4	5.66180	2.15166	8
−6	−6	−4	5.04845	2.09349	10
−6	−6	−4	4.56202	2.43183	10
−6	6	−4	1.90654	0.82728	9
6	6	4	4.41643	3.53592	13
−7	−6	−4	106.043	7.98323	8
−7	−6	−4	100.216	8.08322	10
7	6	4	99.5338	9.93668	13
−7	6	−4	122.515	7.19274	9
−7	6	−4	119.019	6.93309	6
−8	−6	−4	36.1880	3.77235	8
−8	6	−4	37.9773	2.73055	9
−8	6	−4	38.7046	2.44288	6
−8	6	−4	36.4006	2.44693	6
8	6	4	36.3005	5.83626	13
9	6	4	−0.0152	2.16307	13
10	7	−4	31.3730	4.52868	13
−9	−7	4	11.3164	2.61466	10
9	7	−4	12.8018	3.94226	13
−9	7	4	19.2823	2.99213	7
−8	−7	4	35.6803	5.52431	1
−8	−7	4	36.1970	5.07515	10
8	7	−4	45.8319	8.23894	13
−7	−7	4	0.05700	2.27044	10
−7	−7	4	1.24179	4.14351	1
7	7	−4	0.89298	4.15003	13
−7	7	4	0.91740	0.66461	11
−7	7	4	−0.6173	1.62347	7
−6	−7	4	42.4262	6.86519	1
−5	−7	4	88.7766	9.53035	1
−5	−7	4	85.1438	9.87010	5
−5	7	4	95.5680	6.45042	4
−5	7	4	96.9001	6.76786	3
4	−7	−4	33.1018	5.07047	2
−4	−7	4	46.8896	6.11469	1
−4	7	4	35.4730	3.13612	4
−4	7	4	32.2656	3.09531	3
3	−7	−4	209.160	14.4886	2
−3	−7	4	167.840	14.6061	1
−3	7	4	202.971	12.6995	3
−3	7	4	199.607	12.6406	4
2	−7	−4	536.473	34.2705	2
−2	7	4	521.338	32.9276	4
−2	7	4	536.819	32.9348	3
1	7	−4	12.0340	1.80958	4
1	7	−4	11.7162	1.68002	4
1	7	−4	11.4876	2.12265	5

0	−7	−4	16.4724	3.05421	2
0	7	−4	10.0357	1.43506	4
0	7	−4	9.74690	1.42118	4
0	7	−4	8.14592	1.36005	4
−1	−7	−4	472.065	32.1240	2
−1	7	−4	496.790	30.3838	4
−1	7	−4	515.519	30.5315	3
−1	7	−4	495.928	30.5697	3
−1	7	−4	501.821	30.3749	4
−2	−7	−4	65.5216	6.78125	5
−2	−7	−4	69.5243	6.77749	2
−2	−7	−4	63.7698	7.30931	1
−2	7	−4	76.4315	4.88262	3
−2	7	−4	75.2332	4.79452	3
−3	−7	−4	30.3285	4.71012	2
−3	−7	−4	26.7350	5.10979	1
−3	−7	−4	28.9049	4.66759	5
−3	7	−4	25.1891	2.16690	3
−3	7	−4	29.7342	2.33946	3
−4	−7	−4	29.5273	5.38264	2
−4	−7	−4	26.6387	5.05930	1
−4	7	−4	31.6145	2.57964	3
−4	7	−4	28.9461	2.00999	6
−4	7	−4	31.3490	2.02856	6
−5	−7	−4	8.03769	2.12889	8
−5	−7	−4	6.06785	3.49091	1
−5	−7	−4	6.55093	4.11689	2
−5	7	−4	6.11128	0.72052	6
−6	−7	−4	153.434	10.8100	8
−6	−7	−4	151.443	11.2824	10
−6	−7	−4	156.067	11.4421	10
6	7	4	166.838	13.1073	13
−6	7	−4	160.289	9.70980	6
−6	7	−4	160.069	9.86761	9
−6	7	−4	154.211	11.1365	12
−7	−7	−4	84.2483	7.08617	10
−7	−7	−4	83.1239	6.62272	8
7	7	4	73.2890	8.80742	13
−7	7	−4	90.7492	5.66019	9
−7	7	−4	92.5484	5.43513	6
−8	−7	−4	4.70569	1.59875	8
−8	7	−4	2.11147	0.34424	6
−8	7	−4	1.26757	0.66458	9
−8	7	−4	2.24264	0.33997	6
10	8	−4	27.8411	4.59671	13
−9	−8	4	0.29679	2.03240	10
9	8	−4	2.19420	3.34476	13
−9	8	4	3.54973	1.75724	7
−8	−8	4	1.61076	2.50289	10
−8	8	4	1.28442	1.84790	7

8	8	−4	−7.9939	4.40231	13
−7	−8	4	1.22071	4.33791	1
−7	−8	4	1.38747	2.51188	10
−7	8	4	1.45004	1.70191	7
7	8	−4	−2.1985	4.00841	13
6	−8	−4	116.270	11.6197	2
−6	−8	4	139.054	9.89582	10
−6	−8	4	132.900	12.0644	1
−6	8	4	107.253	8.30971	7
−6	8	4	104.141	7.44303	11
−5	−8	4	49.0829	7.70860	5
−5	−8	4	48.3763	7.12441	1
5	−8	−4	46.9747	6.65686	2
−5	8	4	48.9267	3.64486	4
−5	8	4	45.3509	3.73640	4
−5	8	4	43.1775	3.93479	3
4	−8	−4	22.4593	4.31346	2
−4	−8	4	29.6318	5.41651	1
−4	8	4	21.5978	2.36958	3
−4	8	4	20.2164	2.44100	4
−3	−8	4	140.698	12.7706	1
3	−8	−4	110.672	9.80369	2
−3	8	4	118.204	8.05818	4
−3	8	4	114.372	8.06668	3
2	−8	−4	27.3217	4.45106	2
−2	8	4	31.4519	2.86578	4
−2	8	4	29.4872	2.79418	3
1	−8	−4	34.4009	4.83085	2
−1	8	4	33.7863	3.53813	4
1	8	−4	43.9901	3.52636	5
1	8	−4	43.9036	3.56040	4
−1	8	4	44.9654	4.59154	5
0	−8	−4	363.312	22.9901	2
0	8	4	350.430	22.8171	5
0	8	−4	316.716	21.1865	4
0	8	−4	337.640	21.2590	4
−1	8	−4	66.6985	4.72726	3
−1	8	−4	69.7971	4.55749	4
−1	8	−4	70.5856	4.56220	4
−2	−8	−4	94.1478	8.23770	5
−2	−8	−4	89.8115	8.28748	1
−2	−8	−4	90.0565	7.95473	2
−2	8	−4	79.4956	5.69669	3
−2	8	−4	81.5931	5.63000	3
−3	−8	−4	10.4964	3.25555	2
−3	−8	−4	8.81213	2.98216	5
−3	−8	−4	9.08454	3.20930	1
−3	8	−4	13.4376	1.33679	3
−3	8	−4	12.9456	1.39897	3
−4	−8	−4	9.46723	3.46460	1

−4	−8	−4	10.3714	4.18871	2
−4	8	−4	10.2824	0.92902	6
−4	8	−4	10.6799	1.34736	3
−4	8	−4	10.9798	0.88503	6
−5	−8	−4	1.66293	3.18745	2
−5	−8	−4	8.48398	2.80906	10
−5	−8	−4	5.52168	1.94224	8
−5	−8	−4	4.02558	3.60430	1
−5	8	−4	5.68758	0.83242	9
−5	8	−4	5.15809	0.62374	6
−5	8	−4	5.75929	3.23565	12
−5	8	−4	7.01600	0.71828	6
−6	−8	−4	7.35258	3.35868	1
−6	−8	−4	5.82464	2.50666	10
−6	−8	−4	7.43578	2.87020	10
−6	−8	−4	5.61886	2.07440	8
6	8	4	6.62500	3.63010	13
−7	−8	−4	230.309	14.9560	8
−7	−8	−4	221.908	15.1858	10
−7	8	−4	225.816	13.8083	6
7	8	4	237.636	16.9661	13
−7	8	−4	217.387	13.8058	6
−7	8	−4	230.263	13.9832	9
−8	−8	−4	32.3226	3.23285	8
−8	8	−4	26.6140	1.85477	9
−8	8	−4	26.8056	1.87780	9
−9	−9	4	6.91502	2.50311	10
−9	9	4	5.80209	0.89817	9
−9	9	4	7.92214	2.01784	7
9	9	−4	8.56417	4.11457	13
−9	9	4	5.94165	0.72548	6
−8	−9	4	11.0132	2.54265	10
8	9	−4	13.9716	4.77554	13
−8	9	4	8.38593	2.26570	7
−7	−9	4	13.8555	4.79961	1
−7	−9	4	15.6150	3.44535	10
−7	9	4	7.90661	2.22345	7
7	9	−4	12.5862	3.86306	13
6	−9	−4	29.4685	5.93963	2
−6	−9	4	20.8231	3.82993	10
−6	−9	4	25.5636	6.36359	1
−6	9	4	26.2579	2.00215	11
−6	9	4	26.1861	3.23098	7
5	−9	−4	26.0417	5.57225	2
−5	9	4	22.1917	2.04890	4
−5	9	4	22.8375	2.11619	4
−5	9	4	23.6929	2.79151	3
−5	9	4	27.8557	3.36940	7
−4	−9	4	23.6349	5.02958	1
4	−9	−4	25.5912	4.88070	2

−4	9	4	23.0990	2.46419	3
−4	9	4	20.3395	2.36715	4
3	−9	−4	66.0220	7.27899	2
−3	9	4	63.8053	4.85772	4
−3	9	4	65.4535	4.92159	3
2	−9	−4	369.586	24.9252	2
−2	9	4	363.076	23.0303	4
1	−9	−4	52.0075	5.97165	2
−1	9	4	54.6539	5.27123	4
0	−9	−4	597.652	35.2592	2
0	9	−4	517.560	33.1606	4
0	9	−4	506.769	33.1418	4
−1	−9	−4	484.075	29.6599	2
−1	−9	−4	426.911	29.5285	1
−1	9	−4	427.738	27.3129	3
−1	9	−4	432.833	27.0480	4
−1	9	−4	446.999	27.0547	4
−2	−9	−4	95.6039	9.21703	1
−2	−9	−4	93.4662	8.84974	2
−2	9	−4	109.868	6.62207	3
−3	−9	−4	80.1625	7.97355	5
−3	−9	−4	80.9314	8.11983	1
−3	−9	−4	85.0742	8.14735	2
−3	9	−4	66.0472	4.79858	3
−3	9	−4	66.9389	4.83271	3
−4	−9	−4	81.8514	8.86627	2
−4	9	−4	55.3183	4.74175	7
−4	9	−4	56.9505	3.71223	6
−4	9	−4	60.4565	4.02500	3
−4	9	−4	58.8449	3.80031	9
−4	9	−4	58.7640	3.69241	6
−5	−9	−4	130.897	9.56009	8
−5	−9	−4	138.390	10.4462	10
−5	−9	−4	136.705	12.2877	1
−5	−9	−4	136.262	12.8157	2
−5	9	−4	134.073	10.0353	12
−5	9	−4	132.251	8.37478	9
5	9	4	152.193	11.9018	13
−5	9	−4	132.210	8.27534	6
−5	9	−4	129.609	8.24953	6
−6	−9	−4	0.30644	1.84026	8
−6	−9	−4	−0.5535	2.48234	10
−6	−9	−4	0.11187	3.04837	1
−6	9	−4	1.44857	0.35855	6
−6	9	−4	1.20900	0.39349	6
−6	9	−4	0.85113	0.63731	9
−6	9	−4	2.26414	2.31204	12
6	9	4	1.03036	3.45886	13
−7	−9	−4	82.3029	7.01171	10
−7	−9	−4	83.2831	6.77539	8

−7	9	−4	88.0888	5.28760	6
−7	9	−4	92.9761	5.29230	6
7	9	4	77.0496	8.81086	13
−8	−9	−4	−0.3003	1.40143	8
8	9	4	−0.6425	2.86774	13
−9	−10	4	3.73589	1.73884	8
−9	−10	4	3.05732	2.33139	10
−9	10	4	4.39808	1.73894	7
−9	10	4	3.56684	0.77749	9
9	10	−4	2.30159	2.87071	13
−9	10	4	3.77593	0.54493	6
−8	−10	4	38.5499	5.08581	10
8	10	−4	49.3678	6.68832	13
−8	10	4	36.6492	2.84050	9
−8	10	4	31.0554	2.77727	6
−8	10	4	39.4432	4.15963	7
−7	−10	4	5.87422	2.30935	10
−7	−10	4	4.42231	4.04586	1
7	10	−4	3.81036	4.08840	13
−7	10	4	4.10024	2.02722	7
−7	10	4	4.32704	0.99945	9
6	−10	−4	74.2784	9.25041	2
−6	−10	4	87.0626	10.7527	1
−6	−10	4	77.2062	7.42956	10
6	10	−4	76.4940	8.30511	13
−6	10	4	72.1036	4.72420	11
−6	10	4	75.1893	6.05294	7
−6	10	4	76.9872	4.75383	11
5	−10	−4	23.2752	5.12009	2
−5	10	4	26.0373	3.03280	7
−5	10	4	25.5408	2.93576	3
−4	−10	4	3.28311	3.20408	1
4	−10	−4	5.95071	3.02586	2
−4	10	4	2.84516	1.27885	3
−4	10	4	2.44071	1.12542	4
−4	10	4	4.06687	1.37604	4
3	−10	−4	31.5139	5.20274	2
−3	10	4	34.0051	3.10294	3
−3	10	4	32.9517	3.03119	4
2	−10	−4	99.8567	8.84121	2
−2	10	4	90.9170	6.69011	4
1	−10	−4	235.628	16.2517	2
1	10	−4	209.520	14.3104	4
0	10	−4	32.7985	2.82528	4
0	10	−4	32.2309	2.76942	4
−1	10	−4	104.717	6.65920	4
−1	10	−4	106.283	6.65781	4
−2	−10	−4	62.7655	7.57957	1
−2	−10	−4	68.7429	7.50159	2
−2	10	−4	84.0768	5.06129	3

−3	−10	−4	25.1310	5.10610	5
−3	−10	−4	24.5996	4.98755	2
−3	−10	−4	23.8479	5.23569	1
−3	10	−4	31.4743	2.19341	3
−4	−10	−4	37.8651	6.97790	2
−4	−10	−4	38.4702	6.58756	1
−4	10	−4	42.7380	2.95297	9
−4	10	−4	45.1262	2.79701	6
−4	10	−4	46.7647	2.90920	9
−4	10	−4	47.2075	2.82201	6
−5	−10	−4	26.1788	6.19593	1
−5	−10	−4	31.5618	4.93332	10
−5	−10	−4	34.8844	3.93912	8
5	10	4	40.3711	7.22613	13
−6	−10	−4	150.560	10.7019	8
−6	−10	−4	151.826	11.3300	10
−6	−10	−4	153.769	13.2054	1
−6	−10	−4	156.385	11.5132	10
−6	10	−4	159.255	9.26069	6
−6	10	−4	153.908	11.3882	12
6	10	4	141.802	12.2390	13
−6	10	−4	153.098	9.39030	9
−6	10	−4	152.181	9.40065	9
−6	10	−4	145.752	9.24635	6
−7	−10	−4	4.68624	2.20147	10
−7	−10	−4	3.70421	2.01952	8
−7	10	−4	2.58353	2.38733	12
7	10	4	4.10872	3.68066	13
−8	−10	−4	25.5370	2.99908	8
−8	−10	−4	23.1891	2.56729	10
8	10	4	26.5130	4.77180	13
−9	−11	4	0.72925	2.21496	10
−9	11	4	−0.0108	1.50334	7
−9	11	4	0.37689	0.55248	9
−8	−11	4	10.9240	3.13207	10
8	11	−4	9.99689	3.64479	13
−8	11	4	12.9907	1.45936	9
−8	11	4	15.1796	2.55109	7
−8	11	4	13.3961	1.30143	6
−7	−11	4	13.3931	4.93095	1
−7	−11	4	13.4729	3.50087	10
7	11	−4	15.4814	4.60024	13
−7	11	4	18.7440	1.83828	9
−7	11	4	20.9304	3.25132	7
−6	−11	4	174.110	13.2744	10
−6	−11	4	203.157	17.0876	1
−6	11	4	171.229	11.1775	9
−6	11	4	164.364	12.0079	7
−5	−11	4	77.2216	10.1219	1
5	−11	−4	97.7214	9.94110	2

−5	11	4	93.7700	6.72889	3
−5	11	4	87.2299	6.61159	7
4	−11	−4	62.7688	7.63271	2
−4	11	4	61.1295	4.65345	4
−4	11	4	66.4316	5.10944	3
3	−11	−4	2.80274	3.28364	2
−3	11	4	3.38482	1.23701	4
−3	11	4	5.74365	1.38061	3
2	−11	−4	201.001	15.1682	2
−2	11	4	195.191	12.9912	4
1	−11	−4	859.672	51.9981	2
1	11	−4	761.055	49.7794	4
0	−11	−4	167.266	13.9057	2
0	11	−4	183.526	11.6445	4
0	11	−4	187.999	11.8715	4
−1	−11	−4	21.4964	4.62755	2
−1	11	−4	22.6830	1.56253	4
−2	−11	−4	82.5116	8.83371	1
−2	−11	−4	86.0722	8.67138	2
−2	11	−4	93.4315	5.94656	3
−3	−11	−4	529.766	34.9512	1
−3	−11	−4	527.173	34.7205	2
−3	11	−4	481.050	30.6396	6
−3	11	−4	516.388	30.6513	6
−3	11	−4	483.884	30.9241	3
−4	−11	−4	15.3707	4.41516	2
−4	−11	−4	14.8825	3.42559	10
−4	−11	−4	16.8837	4.86681	1
−4	11	−4	16.0971	1.21945	9
−4	11	−4	14.3763	1.28183	9
−4	11	−4	15.7036	1.09908	6
−4	11	−4	16.1726	1.06406	6
−5	−11	−4	280.384	20.7367	1
−5	−11	−4	277.662	17.7943	10
−5	11	−4	240.922	15.5305	9
−5	11	−4	249.344	17.2948	12
−5	11	−4	246.975	15.4224	6
−5	11	−4	234.760	15.4993	9
5	11	4	273.729	18.0052	13
−5	11	−4	250.900	15.4295	6
−6	−11	−4	20.4682	4.62276	1
6	11	4	17.7643	4.11695	13
−6	11	−4	10.0154	2.96693	12
−6	11	−4	10.7840	0.98549	9
−6	11	−4	11.3464	0.96329	9
−7	−11	−4	7.57040	1.77870	8
−7	−11	−4	7.29352	1.94161	10
−7	11	−4	6.93371	2.43466	12
7	11	4	6.59889	3.55573	13
−8	−11	−4	10.0325	1.88471	10

8	11	4	11.8340	2.97408	13
−9	−12	4	0.24621	1.46666	10
−9	12	4	1.08842	0.47027	9
−9	12	4	1.49676	1.48784	7
−8	−12	4	5.44329	2.85613	10
−8	12	4	8.12871	1.05403	9
−8	12	4	9.85634	1.93328	7
−8	12	4	9.12016	0.93296	6
−7	−12	4	15.9313	5.31914	1
−7	12	4	22.3430	3.46451	7
−7	12	4	20.0741	1.61676	6
−7	12	4	20.5384	1.94039	9
−6	−12	4	134.544	10.8693	10
−6	−12	4	152.514	14.2848	1
−6	12	4	125.053	8.70199	9
−6	12	4	137.257	9.55604	7
5	−12	−4	95.5457	10.1672	2
−5	12	4	92.8363	6.82883	7
−5	12	4	88.5359	5.79874	11
4	−12	−4	50.2986	7.76704	2
−4	12	4	50.0579	3.55142	4
−4	12	4	49.3798	3.59229	4
−4	12	4	48.5821	4.28805	3
3	−12	−4	3.66768	3.05950	2
2	−12	−4	78.8712	7.97358	2
−2	12	4	68.4631	5.44517	4
1	−12	−4	407.201	27.0856	2
1	12	−4	380.597	24.7734	4
0	−12	−4	63.7450	8.10631	2
0	12	−4	74.4991	5.18330	4
−1	−12	−4	2.30861	3.13209	2
−2	−12	−4	197.223	15.4715	1
−2	−12	−4	191.356	15.2456	2
−2	12	−4	180.896	11.9785	3
−3	−12	−4	31.1477	6.41590	1
−3	−12	−4	24.8239	5.38802	2
−3	12	−4	41.2895	2.42868	9
−3	12	−4	34.8139	2.56003	3
−3	12	−4	37.2562	2.26411	6
−3	12	−4	32.8332	2.25844	6
−4	−12	−4	73.4995	9.81693	1
−4	−12	−4	73.8692	8.99756	2
−4	−12	−4	63.9480	6.90047	10
−4	12	−4	70.3228	4.37254	6
−4	12	−4	82.9825	7.22756	12
−4	12	−4	69.7830	4.49387	9
−4	12	−4	73.2516	4.45343	9
−4	12	−4	70.0210	4.38553	6
−5	−12	−4	3.35170	3.90727	1
−5	−12	−4	5.44081	3.13175	10

5	12	4	9.41867	4.41354	13
−5	12	−4	13.4095	3.18499	12
−5	12	−4	11.4355	0.88660	9
−6	−12	−4	2.36965	3.16329	10
−6	−12	−4	1.28578	2.68888	10
−6	−12	−4	4.66201	4.11621	1
6	12	4	8.97848	4.55782	13
−7	−12	−4	21.4842	3.76829	10
−7	−12	−4	22.3244	3.61442	8
−7	12	−4	21.9103	3.26749	12
7	12	4	33.8709	5.56270	13
−8	−12	−4	8.80182	1.79455	10
8	12	4	10.2772	2.45671	13
−9	−13	4	0.98666	2.00786	10
−8	−13	4	−0.3034	2.49554	10
−8	13	4	0.37761	0.49302	6
−8	13	4	0.01533	1.72029	7
−8	13	4	−0.0145	0.57286	9
−7	−13	4	11.8581	3.50385	10
−7	−13	4	11.0515	4.93508	1
−7	13	4	21.4691	1.96094	9
−7	13	4	20.1029	2.91185	7
−7	13	4	18.8107	1.48753	6
−6	−13	4	63.5768	9.47427	1
−6	−13	4	53.5293	6.25807	10
−6	13	4	49.5737	3.50507	6
−6	13	4	53.2808	3.86742	9
−6	13	4	53.0923	4.93622	7
5	−13	−4	38.3739	7.00612	2
−5	−13	4	35.7708	7.73019	1
−5	13	4	34.6710	3.74490	7
−5	13	4	34.9320	3.06857	9
5	13	−4	41.0425	6.17898	10
4	−13	−4	10.5228	3.95898	2
−4	13	4	8.61655	1.81766	3
3	−13	−4	44.0724	7.29894	2
−3	13	4	41.3847	3.60169	4
2	−13	−4	110.840	10.4957	2
−2	13	4	113.554	8.00622	4
1	13	−4	8.61470	1.89691	4
0	−13	−4	169.463	13.5144	2
0	13	−4	157.233	10.5995	4
−1	−13	−4	27.1927	5.23729	2
−2	13	−4	37.0453	2.41416	6
−3	−13	−4	8.47421	3.78558	2
−3	−13	−4	10.0017	4.30609	1
−3	13	−4	7.47971	0.87736	9
−3	13	−4	6.36620	0.67344	6
−3	13	−4	7.04760	0.74865	6
−3	13	−4	7.97488	0.93700	3

−3	13	−4	6.38671	0.81510	9
−4	−13	−4	3.98008	3.84506	1
−4	−13	−4	−2.7049	2.72204	10
−4	13	−4	2.69560	0.32526	6
−4	13	−4	2.98943	0.53988	9
−5	−13	−4	6.68809	3.17952	10
−5	−13	−4	10.0639	5.14501	1
5	13	4	9.62441	4.05108	13
−5	13	−4	0.35986	2.55172	12
−6	−13	−4	6.57390	2.86529	10
−6	−13	−4	10.7223	3.51417	10
−6	13	−4	11.9158	2.98378	12
6	13	4	9.33601	4.18583	13
−7	−13	−4	11.5960	2.41374	8
−7	−13	−4	13.5433	2.76939	10
7	13	4	16.5027	3.47191	13
−7	13	−4	10.2622	2.61944	12
−9	−14	4	6.02894	2.20268	10
−8	−14	4	4.41481	2.09181	10
−8	14	4	1.86822	0.44894	6
−8	14	4	2.38786	1.34172	7
−8	14	4	1.60273	0.66421	9
−7	−14	4	10.4302	3.38653	10
−7	14	4	8.96127	0.94689	6
−7	14	4	11.0185	1.34147	9
−7	14	4	11.7764	2.41629	7
−6	−14	4	87.1367	11.8526	1
−6	14	4	106.854	7.03263	9
−6	14	4	112.010	7.93562	7
−6	14	4	108.487	6.66665	6
−5	−14	4	333.085	26.9666	1
5	14	−4	282.029	21.2868	10
−5	14	4	310.994	19.2294	9
−5	14	4	313.918	19.8944	7
4	−14	−4	2.65174	3.65218	2
−4	14	4	4.02594	1.05328	11
3	−14	−4	7.44086	3.73949	2
−3	14	4	7.85948	1.58416	4
2	−14	−4	16.7767	4.60112	2
−2	14	4	21.5093	2.81589	4
1	−14	−4	128.508	11.5149	2
1	14	−4	119.408	8.74826	4
0	−14	−4	28.2670	5.55938	2
0	14	−4	41.6392	3.24146	4
−1	−14	−4	64.3749	7.94225	2
−1	14	−4	74.0424	4.52601	6
−2	14	−4	15.9289	1.20968	6
−2	14	−4	14.9804	1.58021	9
−3	−14	−4	5.92763	3.62186	2
−3	−14	−4	6.40397	4.93528	1

−3	14	−4	6.46332	0.65201	6
−3	14	−4	6.85611	0.94777	9
−3	14	−4	7.31327	0.75505	6
3	14	4	2.04417	3.68427	10
−4	−14	−4	26.5467	4.56786	10
−4	−14	−4	21.4029	5.92999	1
−4	14	−4	23.4512	3.74779	12
−4	14	−4	27.5191	1.82703	9
−4	14	−4	25.3518	3.64902	14
−4	14	−4	27.3816	1.83775	9
−5	−14	−4	8.92364	5.19363	1
−5	−14	−4	7.44012	3.36393	10
−5	14	−4	13.0494	3.15506	12
−6	−14	−4	74.2014	7.31514	10
−6	−14	−4	76.3753	7.06403	10
−6	14	−4	84.1130	7.14892	12
6	14	4	62.2658	8.81440	13
−7	−14	−4	7.03909	2.37474	10
−7	14	−4	7.64753	2.39139	12
−8	−15	4	32.8259	4.43355	10
−8	15	4	21.8346	1.46266	6
−8	15	4	21.7569	1.49229	6
−8	15	4	19.9662	2.53426	7
−8	15	4	21.5993	1.67903	9
−7	−15	4	4.78941	3.04186	10
−7	15	4	4.25898	0.66141	6
−7	15	4	3.38846	0.90812	9
−7	15	4	4.52900	1.99852	7
−6	−15	4	63.0576	10.0768	1
6	15	−4	61.5712	7.91049	10
−6	15	4	77.9856	5.08762	9
−6	15	4	71.4503	5.93954	7
−6	15	4	76.6521	4.70059	6
−5	−15	4	1.23603	2.46282	13
−5	15	4	1.98391	1.18332	9
−5	15	4	4.56231	1.87175	7
5	15	−4	1.05847	3.27863	10
−4	−15	4	24.5549	4.34148	13
4	−15	−4	15.4453	4.97868	2
−4	15	4	30.7580	2.19641	11
−4	15	4	24.9738	2.20062	11
4	15	−4	19.1722	4.27420	10
3	−15	−4	9.13798	4.06660	2
2	−15	−4	1.63359	3.31490	2
−2	15	4	2.95908	1.51704	4
1	−15	−4	46.9621	8.00199	2
1	15	−4	45.5391	4.13918	4
0	−15	−4	57.0361	7.46399	2
0	15	−4	47.1536	3.86844	4
−1	−15	−4	14.7373	4.87105	2

-1	15	-4	12.5481	1.20303	6
-2	-15	-4	77.7945	9.23052	2
-2	15	-4	86.1223	5.53100	9
-2	15	-4	86.3189	5.31306	6
-3	-15	-4	12.8605	4.80076	2
-3	-15	-4	17.8219	5.39437	1
3	15	4	12.2691	4.32321	10
-3	15	-4	12.1845	3.05402	14
-3	15	-4	12.5130	3.14688	12
-3	15	-4	13.6769	1.12662	6
-3	15	-4	15.1430	1.07336	6
-4	-15	-4	81.7427	7.75199	10
-4	-15	-4	83.0148	10.3991	1
-4	15	-4	80.8972	7.01778	12
-4	15	-4	77.4588	6.97552	14
-4	15	-4	69.5266	4.70314	9
-4	15	-4	73.9194	4.71422	9
-5	-15	-4	189.559	16.9432	1
-5	-15	-4	196.804	14.4353	10
-5	-15	-4	195.144	14.5670	10
-5	15	-4	204.295	13.9910	12
-6	-15	-4	25.9944	3.85671	10
-6	-15	-4	27.4493	3.74556	10
-6	15	-4	21.7711	3.69846	12
-7	-15	-4	12.7911	2.30020	10
-8	-16	4	32.6409	3.93972	10
-8	16	4	38.0557	2.33019	9
-8	16	4	33.6482	2.29465	9
-7	-16	4	5.06349	3.21813	10
-7	16	4	8.34798	2.15619	7
-7	16	4	7.63500	0.94531	9
-7	16	4	6.67080	0.74561	6
-6	-16	4	13.2972	5.60726	1
6	16	-4	13.2553	4.48602	10
-6	16	4	23.4915	1.70786	6
-6	16	4	22.0807	3.35340	7
-5	-16	4	140.802	11.0911	13
5	16	-4	145.309	12.2552	10
-5	16	4	160.125	9.85645	9
-5	16	4	156.150	10.4673	7
-4	16	4	20.3313	1.72010	11
-4	16	4	19.3503	1.71049	11
3	-16	-4	5.77283	4.01478	2
-3	16	4	7.76687	1.63070	11
2	-16	-4	340.095	25.0847	2
1	-16	-4	59.7438	8.39859	2
0	-16	-4	9.45986	4.12307	2
-1	16	-4	43.9621	3.50376	9
-2	-16	-4	-0.2220	2.94137	2
-2	16	-4	9.98471	3.74693	12

2	16	4	2.55256	3.37648	10
−3	−16	−4	34.3734	7.95566	1
−3	16	−4	36.9511	5.78465	14
−3	16	−4	38.2352	2.38016	6
−3	16	−4	34.3502	2.66206	9
−3	16	−4	36.4385	4.77702	12
3	16	4	34.6217	6.28970	10
−4	−16	−4	6.89528	4.87367	1
−4	16	−4	6.12816	0.78667	9
−4	16	−4	5.11318	0.57621	9
−4	16	−4	3.86056	2.73561	14
−4	16	−4	5.70374	3.36716	12
−5	−16	−4	231.185	16.5668	10
−5	−16	−4	236.396	16.7519	10
−5	16	−4	234.512	16.1002	12
−6	−16	−4	8.71286	2.95859	10
−6	16	−4	11.3899	2.43841	12
−7	−16	−4	7.80924	2.27243	10
−8	−17	4	38.1561	3.95867	10
−7	−17	4	0.03605	2.65557	10
−7	17	4	−0.0867	0.60582	9
−7	17	4	0.69752	0.40250	6
−7	17	4	0.92109	0.38018	6
7	17	−4	−0.1653	3.56200	10
−6	17	4	4.24563	1.07761	9
6	17	−4	5.78394	3.89337	10
−6	17	4	2.58613	1.88375	7
−6	17	4	3.07912	0.67972	6
−5	−17	4	63.8224	6.26741	13
5	17	−4	62.3844	7.69590	10
−5	17	4	73.0105	4.86063	9
−4	−17	4	118.562	9.19097	13
4	17	−4	117.156	10.1888	10
−3	−17	4	152.282	10.8449	13
3	−17	−4	160.336	17.0285	2
3	17	−4	138.273	11.5112	10
−3	17	4	123.503	9.09337	11
2	−17	−4	187.659	15.8592	2
2	17	−4	194.382	13.3042	10
−2	17	4	159.152	12.2349	10
1	−17	−4	57.2611	8.25690	2
−1	17	4	51.1259	6.08306	10
0	−17	−4	38.0376	7.40248	2
0	17	−4	55.8109	4.60290	11
0	17	4	57.7533	6.87772	10
−1	−17	−4	1.66659	3.09616	2
−1	17	−4	8.15255	1.54550	9
1	17	4	4.91447	3.11414	10
−2	−17	−4	1.98568	2.26011	13
−2	17	−4	4.95410	2.74609	14

−2	17	−4	4.10725	1.11177	9
2	17	4	2.18770	3.30115	10
−2	17	−4	4.49454	0.62388	6
−3	17	−4	7.24546	0.77176	6
−3	17	−4	11.9869	3.05118	14
−3	17	−4	7.28497	1.08920	9
3	17	4	9.66593	4.00306	10
−3	17	−4	11.1408	2.94732	12
−4	−17	−4	148.146	14.5420	1
4	17	4	159.290	12.4640	10
−4	17	−4	125.624	10.5429	12
−5	−17	−4	26.3547	4.40919	10
−5	−17	−4	26.0180	4.67557	10
−5	17	−4	25.7599	4.07603	12
−6	−17	−4	10.3772	2.90370	10
−6	17	−4	4.81099	2.72267	12
−7	−17	−4	1.49028	1.32431	10
−7	18	4	23.6776	1.75788	9
−7	18	4	22.4274	1.49956	6
−7	18	4	21.9245	1.48976	6
7	18	−4	17.6552	4.43112	10
−6	18	4	20.8839	1.58804	6
6	18	−4	18.7393	4.41864	10
−6	18	4	22.3339	1.97235	9
−5	−18	4	9.40641	3.38710	13
5	18	−4	12.2383	4.16441	10
−4	−18	4	21.0444	3.83145	13
4	18	−4	19.3878	4.24080	10
−3	−18	4	3.71063	2.31333	13
−3	18	4	4.63660	1.17059	11
3	18	−4	7.39163	3.74317	10
−2	−18	4	75.5407	6.86068	13
2	18	−4	70.4396	7.12302	10
−2	18	4	86.2337	5.94254	11
1	−18	−4	18.3472	3.49651	13
1	−18	−4	18.1193	5.33916	2
−1	−18	4	16.5468	4.60923	13
−1	18	4	17.2996	3.32469	10
1	18	−4	24.0001	4.20711	10
0	−18	−4	1.56462	2.87719	13
0	−18	−4	0.55306	3.14075	2
0	18	−4	3.07495	1.60751	11
0	18	4	−3.0091	3.20660	10
−1	−18	−4	83.1637	6.96478	13
−1	18	−4	76.3145	7.05062	14
1	18	4	74.4927	7.94516	10
−1	18	−4	77.7167	5.64009	9
−2	−18	−4	−1.4692	2.12972	13
−2	18	−4	1.15939	1.09102	9
−2	18	−4	1.55422	3.18926	14

−2	18	−4	0.91748	0.41558	6
−2	18	−4	1.13122	2.44419	12
2	18	4	−0.0692	3.35577	10
−3	18	−4	10.3155	0.96139	6
−3	18	−4	10.9637	1.38024	9
−3	18	−4	10.1301	3.01200	12
−3	18	−4	10.1414	0.87222	6
3	18	4	15.1485	4.33471	10
4	18	4	79.4759	8.76204	10
−4	18	−4	67.8863	6.98709	12
−5	−18	−4	3.82474	2.94065	10
−5	18	−4	2.54215	2.85244	12
−6	−18	−4	8.41509	2.69846	10
−7	19	4	1.37199	0.41968	9
7	19	−4	0.20055	3.10920	10
−7	19	4	1.58940	0.46060	9
6	19	−4	49.3468	5.67546	10
−6	19	4	39.6597	3.05181	9
−6	19	4	38.8297	2.71388	6
−5	−19	4	40.2860	5.65075	13
−5	19	4	44.2726	2.99152	6
−5	19	4	52.8136	3.66140	9
5	19	−4	34.4582	6.19674	10
−4	−19	4	43.6546	6.00680	13
4	19	−4	43.9923	6.19524	10
−3	19	4	25.5034	2.25094	11
−3	19	4	24.6989	2.21915	11
−2	−19	4	30.4457	5.24988	13
2	19	−4	29.5514	4.60934	10
−2	19	4	36.9393	3.75394	11
−1	−19	4	42.7420	5.42024	13
1	−19	−4	38.1143	4.69155	13
−1	19	4	37.8480	4.85806	10
1	19	−4	27.8225	5.44111	10
0	−19	−4	81.1934	6.86967	13
0	19	4	82.2684	7.50301	10
0	19	−4	72.0774	5.73382	11
−1	−19	−4	167.909	12.1838	13
1	19	4	171.485	13.1364	10
−2	−19	−4	160.420	11.2883	13
−2	19	−4	148.207	9.87710	9
2	19	4	149.418	12.4524	10
−2	19	−4	161.559	9.61035	6
3	19	4	121.036	10.4717	10
−3	19	−4	126.138	10.0607	12
−3	19	−4	128.771	7.99675	9
−3	19	−4	127.434	7.72931	6
−3	19	−4	123.222	7.75570	6
4	19	4	4.74603	4.11137	10
−4	19	−4	8.11382	3.16522	12

−5	−19	−4	28.6175	4.61665	10
5	19	4	26.1839	4.90732	10
−6	−19	−4	0.70786	1.51273	10
7	20	−4	3.42145	2.86238	10
−6	20	4	7.75862	0.78741	6
−6	20	4	7.82471	1.00752	9
6	20	−4	5.97957	3.68849	10
−5	−20	4	23.1816	4.03891	13
−5	20	4	30.4661	2.71473	9
−5	20	4	29.2693	2.11120	6
−4	−20	4	28.0819	4.87260	13
4	20	−4	21.1415	4.35306	10
−3	−20	4	18.4545	3.34500	13
−3	20	4	13.6053	1.41841	11
−3	20	4	13.3796	1.38392	11
3	20	−4	15.3648	3.53936	10
−2	−20	4	229.703	15.3105	13
2	−20	−4	201.082	14.9815	13
2	20	−4	251.980	15.4640	10
−2	20	4	194.684	13.8987	11
−1	−20	4	16.0961	2.92767	13
1	−20	−4	9.39096	2.59441	13
−1	20	4	8.56005	2.83714	10
0	−20	−4	42.0157	5.50409	13
0	20	4	46.4554	6.29020	10
0	20	−4	59.4917	4.01523	11
0	20	−4	59.3970	4.14594	11
−1	−20	−4	28.9237	5.26381	13
1	20	4	34.8264	6.18532	10
−2	−20	−4	3.15265	3.01792	13
2	20	4	0.84326	3.15346	10
−2	20	−4	1.52138	0.98796	9
−2	20	−4	0.18495	0.51644	6
−3	20	−4	7.07114	0.73283	6
3	20	4	7.24476	3.81850	10
−3	20	−4	7.65442	1.11740	9
−3	20	−4	8.71799	0.78064	6
4	20	4	29.2778	5.74081	10
5	20	4	5.95016	2.65415	10
−6	21	4	11.8812	0.93152	6
−6	21	4	10.8694	1.24725	9
6	21	−4	11.9254	3.60262	10
−5	−21	4	−0.2603	2.38537	13
−5	21	4	1.45425	0.85460	9
−5	21	4	1.70576	0.48927	6
5	21	−4	−0.1735	3.23203	10
−4	−21	4	45.6450	5.38796	13
4	21	−4	35.3867	5.80450	10
−3	−21	4	2.55140	3.26821	13
3	21	−4	−0.4970	2.92034	10

2	-21	-4	11.2550	2.71285	13
2	21	-4	19.7014	3.86590	10
-2	21	4	11.4198	1.77747	11
1	-21	-4	45.7133	5.33959	13
-1	-21	4	48.9795	5.93804	13
1	21	-4	50.7400	4.90695	11
-1	21	4	43.9155	4.67266	10
0	-21	-4	23.3538	3.43621	13
0	21	-4	16.1566	1.79969	11
0	21	4	21.0130	4.07166	10
0	21	-4	17.1871	1.94847	11
-1	-21	-4	20.3734	3.48903	13
1	21	4	18.3667	4.00572	10
-2	-21	-4	17.9080	3.29900	13
2	21	4	13.0593	3.97707	10
-2	21	-4	15.1006	1.27531	6
-2	21	-4	16.0210	1.94719	9
-3	21	-4	26.7444	1.68459	6
-3	21	-4	25.0565	2.14689	9
3	21	4	21.2419	4.32625	10
-3	21	-4	24.6527	1.70150	6
4	21	4	0.31660	2.33412	10
5	21	4	3.37909	2.29633	10
6	22	-4	29.3088	4.34379	10
-5	22	4	5.37384	0.62539	6
-5	22	4	3.12742	1.02793	9
5	22	-4	-0.5444	3.04619	10
-4	-22	4	0.94357	2.50939	13
-3	-22	4	168.066	11.5930	13
3	22	-4	147.745	11.6615	10
2	-22	-4	13.1110	3.15230	13
-2	-22	4	7.81994	2.81699	13
-2	22	4	11.7191	1.46014	11
2	22	-4	9.48967	2.52334	10
-2	22	4	14.4779	2.04337	11
-1	-22	4	8.97803	2.75659	13
1	-22	-4	9.65893	2.87552	13
1	22	-4	4.46419	2.00697	11
-1	22	4	7.53444	2.25054	11
0	-22	-4	20.2661	3.50666	13
0	22	4	16.8422	3.22054	10
-1	-22	-4	86.4472	7.44181	13
1	22	4	76.7996	7.57213	10
2	22	4	6.71830	3.37903	10
-2	22	-4	8.78540	1.33935	9
-2	22	-4	9.34300	0.90875	6
3	22	4	8.96537	2.83894	10
-3	22	-4	6.40265	0.58579	6
-3	22	-4	5.89580	0.84501	9
-3	22	-4	7.09058	0.55543	6

4	22	4	4.31509	2.44214	10
5	22	4	-1.2827	1.87763	10
-5	23	4	5.40535	0.82868	9
5	23	-4	3.58184	2.61340	10
-4	-23	4	5.56369	2.47995	13
4	23	-4	4.36443	2.69327	10
-3	-23	4	45.8018	5.17554	13
3	23	-4	38.5693	4.71183	10
2	-23	-4	13.8950	2.73180	13
-2	-23	4	14.2940	2.93615	13
2	23	-4	9.93103	2.32295	11
-1	-23	4	15.2607	3.08635	13
1	-23	-4	16.8701	3.56618	13
1	23	-4	17.4737	2.56508	11
-1	23	4	18.0913	2.54199	11
0	23	4	46.8964	5.24256	10
-1	-23	-4	29.4490	4.54270	13
1	23	4	26.2801	4.36229	10
-2	-23	-4	1.67840	2.56195	13
-2	23	-4	2.67813	1.22455	9
2	23	4	0.41056	3.09324	10
-2	23	-4	4.75416	0.56835	6
3	23	4	12.4256	3.68414	10
-3	23	-4	17.8636	1.36885	9
-3	23	-4	19.5597	1.48853	9
4	23	4	3.20323	2.72652	10
5	24	-4	5.76825	2.09239	10
-4	-24	4	9.90083	2.61252	13
4	24	-4	8.36680	2.68892	10
-4	24	4	12.0024	1.37673	9
-3	-24	4	7.91078	2.42180	13
3	24	-4	7.47457	2.29926	10
-2	-24	4	29.7055	5.18630	13
2	-24	-4	24.0989	3.88849	13
2	24	-4	32.0770	3.25896	11
1	-24	-4	18.8910	3.65489	13
-1	-24	4	29.3669	4.99887	13
0	-24	-4	13.2235	3.01550	13
-1	-24	-4	25.1867	4.35744	13
1	24	4	28.3276	3.94128	10
-2	-24	-4	2.13652	2.09875	13
-2	24	-4	0.95640	0.81113	9
-2	24	-4	1.70230	0.49913	6
2	24	4	1.71049	2.52935	10
3	24	4	18.6450	3.41312	10
4	24	4	3.90179	3.38513	10
-4	-25	4	9.16077	2.11132	13
-3	-25	4	3.05411	2.00904	13
3	-25	-4	-0.0352	1.44161	13
3	25	-4	0.97435	1.42032	11

−2	−25	4	68.3749	5.92162	13
2	−25	−4	70.8102	5.53312	13
2	25	−4	63.7491	4.47426	11
2	25	−4	62.5483	4.52056	11
−1	−25	4	9.84442	2.39538	13
0	−25	−4	−1.1466	2.02011	13
0	−25	4	2.07351	1.81379	13
0	25	4	−1.2630	1.11429	11
−1	−25	−4	1.39544	1.96645	13
1	25	4	0.39486	1.57751	10
−1	25	−4	0.04968	1.23349	9
−2	−25	−4	22.3825	3.16267	13
−2	25	−4	25.8687	2.10985	9
2	25	4	23.2928	3.45211	10
3	25	4	0.97062	1.92844	10
3	−26	−4	7.19852	1.84056	13
−3	−26	4	8.69656	1.89862	13
3	26	−4	7.62791	0.99079	11
3	26	−4	9.36445	1.34597	11
2	−26	−4	4.88079	1.69659	13
−2	−26	4	7.88265	2.41917	13
−1	−26	4	6.27429	1.96076	13
1	−26	−4	5.73312	1.84009	13
0	−26	−4	2.57010	1.77031	13
0	−26	4	2.22758	1.94196	13
0	26	4	2.21636	0.79407	11
0	26	4	2.12642	0.78166	11
−1	−26	−4	0.77305	1.65793	13
1	26	4	0.34095	1.10099	11
−2	−26	−4	7.51862	1.84537	13
2	−27	−4	0.12759	1.56168	13
−2	−27	4	4.49552	1.66904	13
1	−27	−4	−0.2849	1.72538	13
−1	−27	4	−0.1749	1.42346	13
0	−27	−4	0.60524	1.50909	13
0	−27	4	0.33767	1.78117	13
10	0	−5	9.73482	3.14087	13
9	0	−5	−0.3085	3.18640	13
9	0	−5	−0.6177	3.01025	13
−9	0	5	0.32723	1.16614	11
−8	0	5	24.1165	3.44615	10
8	0	−5	25.0142	4.40773	13
−7	0	5	5.77613	2.76078	10
−6	0	5	10.5210	3.35653	1
−5	0	5	6.74607	1.72036	5
−5	0	5	6.71133	2.97617	1
−4	0	5	211.220	15.7772	1
−4	0	5	231.153	16.0718	5
−3	0	5	7.61437	3.02942	1
−3	0	5	13.9244	3.92894	5

−2	0	5	245.123	17.4763	5
1	0	−5	330.429	21.3208	2
−1	0	5	345.225	23.0080	5
0	0	−5	803.701	48.6494	5
0	0	−5	800.554	48.0765	3
0	0	−5	734.664	48.0606	4
−1	0	−5	1.00250	1.36641	4
−1	0	−5	0.80074	0.93940	3
−1	0	−5	1.13324	1.20151	5
−1	0	−5	1.33398	1.44348	5
−2	0	−5	481.971	29.2965	4
−2	0	−5	454.456	29.8094	1
−3	0	−5	−0.5350	0.86685	3
−3	0	−5	0.34612	1.32722	1
−3	0	−5	0.23073	1.18375	4
−4	0	−5	20.3211	2.62799	4
−5	0	−5	2.87440	1.39266	8
−6	0	−5	13.2347	2.08845	8
−7	0	−5	4.84107	1.56732	8
10	−1	−5	26.0042	3.71245	13
10	1	−5	16.9810	3.54155	13
9	−1	−5	1.36985	3.00258	13
−9	−1	5	1.09004	1.16926	11
9	−1	−5	0.84503	3.00013	13
−9	1	5	1.12852	1.01783	11
9	1	−5	1.91522	3.16807	13
−8	−1	5	6.09023	2.57013	10
8	−1	−5	8.05964	3.32114	13
−8	1	5	9.59849	2.38513	10
8	1	−5	7.89268	4.22115	13
−7	−1	5	47.0048	5.48353	10
7	−1	−5	47.9339	6.92963	13
−7	−1	5	52.4310	5.66689	1
−7	1	5	37.8121	4.64324	10
7	1	−5	47.1934	7.03801	13
−6	−1	5	2.23823	2.92560	1
−6	1	5	7.43405	2.75383	1
−5	−1	5	119.768	8.92715	5
−5	−1	5	115.952	9.65342	1
−5	1	5	89.6062	8.84398	1
−4	1	5	99.9345	8.92342	1
−4	1	5	104.042	8.44745	5
−3	−1	5	12.0847	3.19397	1
−3	−1	5	16.5808	3.83864	5
−3	1	5	15.3160	3.52947	1
2	−1	−5	28.7836	3.46634	2
−2	−1	5	31.9649	4.83630	5
−2	1	5	27.9798	4.47098	5
1	−1	−5	1040.84	61.5904	2
−1	1	5	1096.72	63.1797	5

1	1	−5	866.229	61.3060	4
0	−1	−5	523.681	34.0636	4
0	−1	−5	558.936	34.6998	5
0	−1	−5	530.534	34.1487	2
0	1	−5	548.923	33.9271	3
0	1	−5	556.504	34.0232	4
0	1	−5	570.432	34.4042	5
−1	−1	−5	145.600	9.49120	2
−1	−1	−5	150.037	9.22847	4
−1	−1	−5	111.837	9.40410	3
−1	−1	−5	138.490	9.42936	5
−1	1	−5	128.386	9.02713	4
−1	1	−5	132.563	8.52792	5
−1	1	−5	129.069	8.92555	3
−1	1	−5	131.426	8.60319	5
−2	−1	−5	28.6502	3.27684	4
−2	−1	−5	30.4727	4.11372	1
−2	−1	−5	31.3531	3.97085	2
−2	1	−5	43.6814	3.77777	1
−3	−1	−5	18.7043	3.09742	1
−3	−1	−5	20.6925	2.71269	4
−3	1	−5	14.2983	2.20285	4
−3	1	−5	13.4388	2.14214	3
−4	1	−5	27.8260	2.81007	4
−4	1	−5	21.9023	3.36309	3
−5	−1	−5	43.5889	3.76010	8
−5	1	−5	39.4327	3.52542	8
−6	−1	−5	318.397	19.9841	8
−6	1	−5	312.571	19.8153	8
−7	−1	−5	47.7799	3.83964	8
7	1	5	48.9365	8.98930	13
−7	1	−5	42.6028	3.67383	8
−8	−1	−5	29.8850	2.54670	8
8	1	5	26.8239	4.04599	13
10	−2	−5	4.06028	2.64201	13
10	2	−5	6.92418	2.96098	13
−10	2	5	2.77155	1.90111	7
−9	−2	5	0.95062	1.77535	10
9	−2	−5	−0.0522	2.92274	13
−9	−2	5	1.16838	1.24338	11
9	2	−5	0.36181	3.18597	13
−9	2	5	0.63657	1.69425	7
−8	−2	5	7.33475	2.40970	10
8	−2	−5	5.81963	3.13513	13
−8	2	5	8.77789	1.66538	11
8	2	−5	8.30295	3.64415	13
−7	−2	5	116.698	8.80401	10
7	−2	−5	108.269	9.99624	13
−7	−2	5	89.4439	9.43287	1
−7	2	5	113.668	8.30960	10

7	2	−5	114.962	10.3146	13
−6	−2	5	96.7431	9.42315	1
−5	−2	5	14.6719	3.37862	5
−5	−2	5	16.9476	4.30457	1
−4	−2	5	651.346	46.2862	1
−4	−2	5	640.627	46.6003	5
−4	2	5	789.181	46.0686	1
−4	2	5	804.119	45.2614	5
−3	−2	5	318.745	21.2427	1
−3	−2	5	322.914	21.7938	5
−3	2	5	281.234	20.5026	1
−3	2	5	304.532	20.8343	5
2	−2	−5	655.692	41.1893	2
−2	−2	5	664.910	42.5929	5
−2	2	5	651.840	42.1523	5
1	−2	−5	50.3003	4.70450	2
−1	2	5	56.2828	6.24858	5
0	−2	−5	385.620	25.4798	5
0	−2	−5	365.068	24.9837	2
0	2	−5	395.259	24.5755	3
0	2	−5	391.589	24.6747	4
0	2	−5	423.675	25.2005	5
0	2	−5	397.189	24.8265	5
−1	−2	−5	7.10908	1.84006	4
−1	−2	−5	6.52746	2.02015	5
−1	−2	−5	7.30499	1.96146	2
−1	2	−5	0.61614	1.11744	4
−1	2	−5	0.38599	1.05161	4
−1	2	−5	0.46482	1.03273	3
−2	−2	−5	92.2070	7.70509	1
−2	−2	−5	93.6108	6.92717	4
−2	−2	−5	91.7379	6.80712	5
−2	−2	−5	90.3543	7.53567	2
−2	2	−5	83.5687	6.52524	1
−2	2	−5	87.8532	6.33891	4
−2	2	−5	96.3347	6.44217	3
−3	−2	−5	80.2850	7.09772	2
−3	−2	−5	75.6899	5.89849	4
−3	−2	−5	70.8299	6.51407	1
−3	2	−5	77.0522	5.36561	4
−3	2	−5	70.1513	5.44709	3
−3	2	−5	68.6801	5.07814	4
−4	−2	−5	130.895	11.3401	2
−4	−2	−5	130.415	8.96666	1
−4	2	−5	100.710	7.39498	4
−4	2	−5	111.869	7.57770	4
−4	2	−5	104.911	8.03639	3
−5	−2	−5	74.3514	5.72183	8
−5	2	−5	81.8336	5.67847	8
−6	−2	−5	28.9375	3.04809	8

−6	2	−5	26.6089	2.79924	8
6	2	5	30.4135	5.07379	13
−7	−2	−5	259.960	16.1109	8
7	2	5	241.849	17.5102	13
−7	2	−5	252.399	15.7521	8
−8	−2	−5	8.30652	1.42755	8
8	2	5	7.43572	3.10095	13
10	−3	−5	1.07985	2.45982	13
10	3	−5	5.31645	2.93962	13
9	−3	−5	5.00758	3.12045	13
−9	−3	5	10.5822	2.16691	10
−9	3	5	6.67475	2.00102	7
9	3	−5	9.59100	3.65640	13
−8	−3	5	−1.2208	1.99200	10
8	−3	−5	2.66130	3.50936	13
8	3	−5	−0.0442	3.29120	13
8	3	−5	−0.6990	3.11748	13
−8	3	5	3.85480	1.27577	11
−7	−3	5	47.9906	6.95730	1
7	−3	−5	59.9628	6.90270	13
−7	−3	5	58.2652	6.08890	10
7	3	−5	49.3660	6.84642	13
−7	3	5	51.9943	5.17553	10
−6	−3	5	73.9819	8.31305	1
−6	3	5	61.1019	5.36544	4
−5	−3	5	22.7616	4.99435	1
−5	−3	5	18.6293	4.50598	5
−4	−3	5	7.43324	3.62696	5
−4	3	5	14.2849	1.79006	5
−4	3	5	14.6825	1.92990	5
−4	3	5	15.2713	2.89429	4
−3	−3	5	516.216	32.6675	5
−3	−3	5	470.612	31.8046	1
−3	3	5	463.832	31.0028	5
2	−3	−5	951.125	59.3553	2
−2	3	5	960.720	60.0070	5
1	−3	−5	149.554	10.2636	2
−1	3	5	143.723	11.1070	5
1	3	−5	136.411	9.57541	4
0	−3	−5	757.003	54.4940	2
0	−3	−5	785.035	54.8752	5
0	3	−5	931.649	54.1919	5
0	3	−5	933.869	54.0325	5
0	3	−5	935.878	54.0497	3
0	3	−5	940.337	54.0681	4
−1	−3	−5	21.1390	3.34457	2
−1	−3	−5	26.2728	3.34136	5
−1	3	−5	18.7554	2.37029	4
−1	3	−5	19.4297	2.07514	4
−1	3	−5	19.6916	2.15215	3

−2	−3	−5	119.920	9.23474	5
−2	−3	−5	118.769	9.48465	2
−2	−3	−5	122.070	9.88369	1
−2	3	−5	132.210	8.28842	4
−2	3	−5	126.560	8.36914	3
−3	3	−5	35.2540	2.68313	4
−3	3	−5	33.7293	2.87716	4
−3	3	−5	33.1467	3.05533	3
−4	−3	−5	89.8445	7.81921	1
−4	−3	−5	85.8639	8.56477	2
−5	−3	−5	172.396	11.2699	8
−5	−3	−5	177.527	11.8579	10
−5	3	−5	160.155	10.8898	8
−6	−3	−5	62.3140	4.94181	8
−6	−3	−5	63.4144	5.33363	10
6	3	5	61.1467	7.77302	13
−6	3	−5	56.8923	4.41449	8
−7	−3	−5	61.9788	4.98731	8
7	3	5	65.8592	7.19511	13
−7	3	−5	51.3260	4.25119	9
−8	−3	−5	5.09707	1.41931	8
8	3	5	4.16799	3.03434	13
−8	3	−5	3.53407	0.61188	6
−8	3	−5	6.07103	1.19342	9
10	−4	−5	4.39057	2.52191	13
10	4	−5	5.22622	2.93568	13
9	−4	−5	−2.1511	2.78250	13
−9	−4	5	2.46716	1.83112	10
−9	4	5	1.58855	1.26308	7
9	4	−5	1.28772	3.43033	13
−8	−4	5	0.59981	2.14150	10
8	4	−5	0.16784	3.86891	13
8	4	−5	−0.2814	4.04703	13
−7	−4	5	93.9445	8.11884	10
−7	−4	5	99.7532	9.89151	1
7	4	−5	94.7325	8.97114	13
−6	−4	5	64.8387	7.90265	1
−5	−4	5	26.1992	5.34715	1
−5	−4	5	20.9910	5.14200	5
−4	−4	5	894.141	55.6701	1
−4	−4	5	887.700	56.2584	5
−4	4	5	819.110	53.3278	4
−3	−4	5	649.448	47.3881	1
3	−4	−5	788.171	47.1938	2
−3	4	5	789.375	46.5591	5
−3	4	5	745.120	45.8359	4
2	−4	−5	61.4919	5.87796	2
−2	4	5	63.7314	6.32022	5
1	−4	−5	282.398	18.4387	2
−1	4	5	282.051	19.1685	5

1	4	−5	270.065	17.7091	4
0	−4	−5	223.438	13.7455	5
0	4	−5	179.764	12.1066	4
0	4	−5	182.115	11.5439	5
0	4	−5	179.531	11.5518	5
0	4	−5	176.074	12.0815	3
−1	−4	−5	75.0220	6.79362	5
−1	−4	−5	72.3438	6.72246	2
−1	4	−5	88.5989	5.56942	4
−1	4	−5	76.5358	5.52356	3
−1	4	−5	74.7805	5.45140	4
−2	−4	−5	359.181	25.3873	1
−2	−4	−5	382.367	25.1544	5
−2	−4	−5	369.401	25.3498	2
−2	4	−5	404.612	23.7496	4
−2	4	−5	369.184	23.6300	4
−2	4	−5	411.949	23.9928	3
−3	−4	−5	18.8320	3.51166	2
−3	−4	−5	19.1527	2.52745	5
−3	−4	−5	19.7591	4.05401	1
−3	4	−5	14.1097	1.42011	4
−3	4	−5	13.2727	1.36300	4
−3	4	−5	13.5470	1.95629	3
−4	−4	−5	305.204	21.8370	2
−4	−4	−5	304.741	21.0980	1
−4	4	−5	306.899	18.7032	6
−5	−4	−5	19.3510	3.26919	10
−5	−4	−5	13.4659	3.93491	2
−5	−4	−5	14.6223	2.51854	8
−5	4	−5	16.1753	1.74159	9
−5	4	−5	18.4455	1.53666	6
−5	4	−5	16.4731	2.14605	8
−6	−4	−5	199.180	13.1941	10
−6	−4	−5	201.749	12.9866	8
−6	4	−5	181.861	12.2645	9
6	4	5	194.920	14.3239	13
−6	4	−5	196.648	12.0657	6
−7	−4	−5	64.0659	5.16425	8
−7	4	−5	57.0494	3.94542	6
7	4	5	60.4544	7.24995	13
−7	4	−5	60.6775	4.23564	9
−8	−4	−5	−0.2339	1.36950	8
−8	4	−5	−0.5625	0.91143	9
8	4	5	2.42505	3.09225	13
−8	4	−5	0.21585	0.46683	6
10	−5	−5	10.6989	2.98844	13
10	5	−5	10.6639	3.48534	13
9	−5	−5	3.69388	3.28675	13
−9	−5	5	17.3574	3.07639	10
−9	5	5	10.2794	2.23106	7

9	5	−5	10.7892	3.84263	13
−8	5	5	20.7451	2.96556	7
8	5	−5	11.1907	3.96623	13
−8	5	5	18.1015	1.81436	11
−7	−5	5	46.5054	7.47557	1
−7	−5	5	44.0703	5.39626	10
7	5	−5	47.5806	6.47670	13
−7	5	5	41.6090	3.52877	11
−6	−5	5	115.763	11.1650	1
−5	−5	5	221.802	18.7568	5
−5	−5	5	243.141	18.6386	1
−5	5	5	271.319	16.1462	4
−4	−5	5	359.825	23.4609	1
−4	−5	5	351.011	23.9051	5
−4	5	5	273.164	20.5701	4
−3	−5	5	388.545	23.5582	1
3	−5	−5	322.561	22.4908	2
−3	5	5	318.001	21.2386	5
−3	5	5	309.742	21.0512	4
2	−5	−5	129.234	9.93681	2
−2	5	5	128.144	10.0702	5
1	−5	−5	114.952	8.94489	2
−1	5	5	114.120	9.49388	5
0	−5	−5	103.657	8.00264	2
0	−5	−5	99.3932	8.21128	5
0	5	−5	96.6428	6.78285	4
0	5	−5	92.3332	6.72808	3
0	5	−5	101.516	7.07548	4
0	5	5	99.2994	8.69952	5
−1	−5	−5	279.945	20.2414	1
−1	−5	−5	290.061	19.9725	2
−1	−5	−5	299.903	20.1729	5
−1	5	−5	303.975	18.5638	4
−1	5	−5	300.411	18.6657	3
−1	5	−5	303.990	18.5408	4
−2	−5	−5	3.26154	2.25786	5
−2	−5	−5	3.75091	2.10766	2
−2	−5	−5	3.52748	2.28085	1
−2	5	−5	3.98190	0.83200	4
−2	5	−5	3.58646	0.82571	4
−3	−5	−5	150.065	11.6572	1
−3	−5	−5	157.745	12.0358	2
−3	−5	−5	151.490	10.9014	5
−3	5	−5	134.639	9.73891	3
−4	−5	−5	11.6558	1.97418	8
−4	−5	−5	12.9474	3.53374	1
−4	−5	−5	11.2151	3.35004	2
−4	5	−5	11.5832	1.15095	6
−4	5	−5	10.5250	1.77068	3
−5	−5	−5	47.9275	6.57452	2

−5	−5	−5	41.7143	4.83240	10
−5	−5	−5	42.2987	5.44107	1
−5	−5	−5	41.9917	5.32422	10
−5	−5	−5	39.7975	4.17523	8
−5	5	−5	42.2035	3.10355	9
−5	5	−5	39.9546	2.87369	6
−6	−5	−5	360.906	24.4259	8
−6	−5	−5	387.184	24.8624	10
−6	5	−5	406.375	23.6426	6
6	5	5	360.375	25.7946	13
−6	5	−5	417.200	23.8489	9
−7	−5	−5	105.511	7.17216	8
−7	5	−5	97.0663	6.38614	9
−7	5	−5	92.3084	6.13712	6
7	5	5	97.0695	9.13714	13
−8	−5	−5	2.13329	1.51740	8
−8	5	−5	2.09077	0.44079	6
8	5	5	0.10586	2.98551	13
−8	5	−5	2.11724	0.80622	9
10	−6	−5	14.5705	2.69221	13
10	6	−5	14.2820	3.13378	13
−9	−6	5	7.49754	2.53038	10
−9	6	5	7.36204	1.73860	7
−8	−6	5	18.3088	4.12919	1
−8	−6	5	12.4652	3.05987	10
−8	6	5	11.3058	1.30536	11
8	6	−5	19.4374	4.96179	13
−8	6	5	9.61518	2.36304	7
−7	−6	5	45.0191	5.36427	10
−7	−6	5	48.9190	7.98065	1
7	6	−5	37.5175	6.05800	13
−7	6	5	41.5048	3.30338	11
7	6	−5	40.0585	6.18566	13
−6	−6	5	71.6193	9.30145	1
−5	−6	5	21.7359	5.17017	5
−5	−6	5	18.4350	5.05915	1
−5	6	5	14.8319	2.27468	4
4	−6	−5	761.150	44.8215	2
−4	−6	5	613.432	44.6795	1
−3	−6	5	170.784	12.5423	1
3	−6	−5	136.757	11.3388	2
−3	6	5	138.497	9.94622	4
2	−6	−5	78.2732	7.22064	2
−2	6	5	75.8269	6.58028	5
1	−6	−5	14.1917	2.84854	2
−1	6	5	13.6295	3.67836	5
0	−6	−5	38.8553	5.03684	2
0	6	5	50.0920	5.82427	5
0	6	−5	52.3811	4.02360	4
0	6	−5	47.2618	3.67930	4

−1	−6	−5	5.67499	2.90882	1
−1	−6	−5	6.53643	2.75253	5
−1	−6	−5	7.73176	2.50388	2
−1	6	−5	12.2047	1.44385	4
−1	6	−5	12.4633	1.62160	4
−1	6	−5	13.0986	1.57226	3
−2	−6	−5	26.6531	5.08051	1
−2	−6	−5	28.2663	4.24060	2
−2	−6	−5	26.1282	4.50453	5
−2	6	−5	29.0919	2.60751	3
−2	6	−5	32.2219	2.11892	4
−2	6	−5	29.5120	2.09454	4
−3	−6	−5	32.8393	5.28826	2
−3	−6	−5	35.2769	4.51753	5
−3	−6	−5	30.0557	4.89682	1
−4	−6	−5	371.683	25.0292	2
−4	−6	−5	369.951	24.4914	1
−4	−6	−5	349.561	22.1263	8
−4	6	−5	340.548	21.3099	6
−4	6	−5	322.677	21.9987	3
−5	−6	−5	268.069	17.8866	1
−5	−6	−5	251.979	16.5111	10
−5	−6	−5	245.810	19.0377	2
−5	−6	−5	253.961	16.4974	10
−5	−6	−5	246.307	15.8087	8
−5	6	−5	224.220	14.9833	9
5	6	5	235.339	17.1720	13
−5	6	−5	230.307	16.2003	12
−5	6	−5	230.205	14.8560	6
−6	−6	−5	4.26456	2.06809	10
−6	−6	−5	5.03811	1.86230	8
6	6	5	7.33679	4.32518	13
−6	6	−5	5.19586	1.04090	9
−6	6	−5	5.22570	0.76090	6
−6	6	−5	5.00939	2.28389	12
−7	−6	−5	53.1598	4.53156	10
−7	−6	−5	53.7908	4.63680	8
−7	6	−5	51.9483	3.34990	6
−7	6	−5	48.6613	3.56722	9
7	6	5	42.0523	8.14140	13
−8	−6	−5	13.6486	2.12015	8
−8	6	−5	11.9032	0.92531	6
−8	6	−5	13.5403	1.32845	9
8	6	5	9.01285	3.21802	13
10	7	−5	5.19725	2.47442	13
−9	−7	5	2.07547	2.19161	10
−9	7	5	3.43908	1.85670	7
−8	−7	5	7.59139	2.98285	10
−8	−7	5	6.07855	3.05257	1
−8	7	5	5.30791	2.11039	7

8	7	−5	10.0130	4.92815	13
−7	−7	5	166.540	14.7759	1
−7	−7	5	163.991	12.6311	10
7	7	−5	177.421	13.4711	13
−7	7	5	167.816	10.6902	11
−7	7	5	169.442	11.7423	7
−6	−7	5	63.5858	8.34212	1
6	−7	−5	74.3889	9.37485	2
−6	−7	5	54.2407	6.27218	10
5	−7	−5	11.1763	4.34400	2
−5	7	5	10.1621	1.83796	4
−4	−7	5	40.7932	7.07986	1
4	−7	−5	56.9119	6.50895	2
−4	7	5	56.0201	4.29079	4
3	−7	−5	651.605	41.5548	2
−3	7	5	636.078	39.8699	4
2	−7	−5	695.272	44.9699	2
−2	7	5	714.487	44.1320	5
1	−7	−5	106.252	8.37790	2
1	7	−5	89.5852	6.86784	5
1	7	−5	91.5236	6.85760	4
−1	7	5	110.179	8.56463	5
0	−7	−5	33.7271	5.11151	2
0	7	5	30.6924	4.78054	5
−1	−7	−5	32.8792	4.38085	2
−1	−7	−5	28.8892	4.56820	1
−1	7	−5	28.1613	2.39196	4
−1	7	−5	26.0401	2.42569	3
−1	7	−5	25.5301	2.29402	4
−2	−7	−5	124.382	10.0055	2
−2	−7	−5	111.908	9.95738	1
−2	−7	−5	113.264	9.92559	5
−2	7	−5	114.160	7.70371	3
−3	−7	−5	405.475	27.5328	1
−3	−7	−5	427.653	27.3141	5
−3	−7	−5	410.009	27.7884	2
−3	7	−5	368.685	24.9860	3
−4	−7	−5	95.2710	6.90575	8
−4	−7	−5	107.096	9.76251	1
−4	−7	−5	100.245	9.64390	2
−4	7	−5	89.7425	6.49207	3
−4	7	−5	87.3814	5.98151	9
−4	7	−5	91.8087	5.85697	6
−5	−7	−5	2.72697	1.92656	8
−5	−7	−5	9.88536	3.61784	10
−5	−7	−5	7.75166	3.13827	10
−5	−7	−5	4.84873	3.26570	1
−5	−7	−5	1.35118	3.07379	2
−6	−7	−5	31.0688	3.95806	10
−6	−7	−5	28.5681	4.00830	8

−6	7	−5	25.8714	2.26562	9
−6	7	−5	25.6789	1.97263	6
6	7	5	31.8047	5.59835	13
−6	7	−5	26.6437	4.07125	12
−7	−7	−5	3.30811	1.75837	10
−7	−7	−5	3.48412	1.82661	8
−7	7	−5	1.08573	0.48450	6
7	7	5	8.69717	3.83162	13
−7	7	−5	2.87466	0.80727	9
−7	7	−5	1.26813	0.52654	6
−8	7	−5	9.72792	1.03127	9
−8	7	−5	11.9418	1.08085	9
−9	−8	5	2.22819	2.15076	10
−9	8	5	−1.0155	1.58252	7
9	8	−5	1.51076	3.80999	13
−8	−8	5	1.66491	2.51177	10
−8	8	5	7.88897	2.19559	7
8	8	−5	−1.2412	4.34541	13
−7	−8	5	106.245	10.6512	1
7	8	−5	100.094	9.31917	13
−7	8	5	69.1620	5.24066	11
−7	8	5	76.3323	6.40035	7
−6	−8	5	154.085	11.9269	10
−6	−8	5	155.749	14.4423	1
6	−8	−5	156.601	14.0432	2
−6	8	5	142.064	9.65236	11
5	−8	−5	35.9970	6.29807	2
−5	8	5	37.2566	3.37542	4
4	−8	−5	49.9499	6.34196	2
−4	8	5	43.6804	3.92835	4
3	−8	−5	50.4355	6.47004	2
−3	8	5	52.0569	4.25018	4
2	−8	−5	269.964	18.9876	2
1	−8	−5	194.110	14.0132	2
1	8	−5	181.892	12.1981	4
1	8	−5	168.493	12.1933	4
−1	8	5	203.854	13.5559	5
0	−8	−5	62.9301	6.52378	2
0	8	5	56.4840	6.71027	5
0	8	−5	62.8243	4.48491	4
0	8	−5	59.9133	4.48189	4
−1	−8	−5	−0.1875	2.02834	1
−1	−8	−5	0.89672	2.07887	2
−1	8	−5	1.30261	0.73280	4
−1	8	−5	1.22641	0.73488	4
−2	−8	−5	24.6197	4.46254	2
−2	−8	−5	23.6089	4.55472	5
−2	−8	−5	21.3197	4.53202	1
−2	8	−5	20.1671	2.05331	3
−3	−8	−5	93.3686	9.45913	1

−3	−8	−5	93.4442	9.23333	5
−3	−8	−5	91.3642	9.45722	2
−3	8	−5	105.117	6.84174	3
−3	8	−5	109.505	6.37543	6
−3	8	−5	109.834	6.93755	3
−4	−8	−5	5.33825	3.32427	2
−4	−8	−5	6.15124	3.39162	1
−4	−8	−5	6.84237	2.18097	8
−4	8	−5	8.15294	1.29443	3
−4	8	−5	8.53413	0.98165	9
−4	8	−5	7.96478	0.84842	6
−5	−8	−5	27.5817	5.33773	1
−5	−8	−5	21.7513	4.04792	10
−5	−8	−5	23.0650	4.35770	10
−5	−8	−5	23.6377	3.35614	8
−5	8	−5	17.2464	1.80503	9
−5	8	−5	19.6125	3.51131	12
−5	8	−5	16.9590	1.46176	6
−6	−8	−5	461.515	29.7309	10
−6	−8	−5	475.253	29.2425	8
−6	8	−5	455.040	29.5196	12
−6	8	−5	472.124	28.1260	6
−6	8	−5	448.050	28.1045	6
6	8	5	477.190	30.4598	13
−6	8	−5	453.115	28.2737	9
−7	−8	−5	58.8207	4.81163	8
−7	−8	−5	57.7375	4.82379	10
−7	8	−5	50.5132	3.30198	6
7	8	5	60.4881	7.17899	13
−7	8	−5	50.2425	3.50420	9
−7	8	−5	48.8947	3.28466	6
−8	−8	−5	18.9099	2.48559	8
8	8	5	20.7970	3.52173	13
−9	−9	5	8.48242	2.12328	10
9	9	−5	7.19911	3.30551	13
−9	9	5	3.67435	1.84660	7
−8	−9	5	2.10646	2.62282	10
−8	9	5	5.05853	2.06508	7
8	9	−5	1.73281	3.42121	13
−7	−9	5	61.8722	6.63693	10
−7	−9	5	61.1691	9.55903	1
7	9	−5	60.2301	8.01185	13
−7	9	5	65.1089	5.46020	7
−6	−9	5	13.9457	3.28737	10
6	−9	−5	9.72496	4.31885	2
−6	−9	5	7.57450	4.23840	1
−6	9	5	10.8195	1.39689	11
−6	9	5	12.4335	2.54576	7
5	−9	−5	14.9117	4.43915	2
−5	−9	5	13.3716	5.35165	1

−5	9	5	17.1286	2.57542	7
−4	−9	5	9.09969	4.53810	1
4	−9	−5	18.7185	4.32589	2
−4	9	5	17.0168	2.41325	4
3	−9	−5	21.7268	4.32194	2
−3	9	5	20.3200	2.48417	4
2	−9	−5	258.386	18.5156	2
1	−9	−5	171.673	13.1655	2
−1	9	5	164.040	12.1623	5
0	−9	−5	1.24218	2.59559	2
0	9	5	1.05171	2.08177	5
−1	−9	−5	315.648	21.8430	2
−1	−9	−5	304.172	22.0669	1
−1	9	−5	321.499	19.3788	4
−1	9	−5	312.775	19.3465	4
−2	−9	−5	212.879	16.3668	1
−2	−9	−5	213.565	16.0355	2
−3	−9	−5	47.6145	7.11383	1
−3	−9	−5	44.3850	6.04968	5
−3	−9	−5	39.8176	6.32606	2
−3	9	−5	51.6716	3.66348	3
−3	9	−5	50.1986	3.09776	6
−3	9	−5	50.5424	3.59373	3
−4	−9	−5	5.38922	3.40128	1
−4	−9	−5	5.35389	2.68179	10
−4	−9	−5	5.74906	2.25358	8
−4	−9	−5	7.27336	4.21455	2
−4	9	−5	2.99465	0.65647	9
−4	9	−5	1.10797	0.39880	6
−4	9	−5	1.63220	0.46284	6
−4	9	−5	1.43533	0.72245	3
−5	−9	−5	142.952	10.4947	10
−5	−9	−5	144.693	10.7197	10
−5	−9	−5	144.959	9.83561	8
−5	−9	−5	156.347	12.6674	1
−5	9	−5	128.548	10.4331	12
−5	9	−5	130.829	8.57312	9
−5	9	−5	128.075	8.42817	6
−5	9	−5	128.609	8.45592	6
5	9	5	146.272	12.0373	13
−6	−9	−5	15.8682	2.72604	8
−6	−9	−5	12.9754	2.94476	10
−6	9	−5	16.8025	1.24158	6
6	9	5	16.7617	5.07091	13
−6	9	−5	18.4968	1.27341	6
−6	9	−5	15.4381	3.09083	12
−6	9	−5	17.9978	1.57028	9
−7	−9	−5	46.7080	4.54925	8
−7	−9	−5	46.7624	4.49203	10
7	9	5	53.1961	6.85787	13

−7	9	−5	47.1444	3.11391	9
−7	9	−5	48.5055	3.08522	9
−7	9	−5	41.7706	4.97607	12
8	9	5	16.8093	3.21032	13
−9	−10	5	5.67320	2.42591	10
9	10	−5	3.55560	3.46135	13
−9	10	5	4.64505	0.80777	9
−9	10	5	2.74505	1.67861	7
−8	−10	5	10.7656	3.31759	10
8	10	−5	10.5509	4.40020	13
−7	−10	5	26.0657	6.19970	1
−7	−10	5	22.9404	4.53069	10
7	10	−5	23.2490	4.21432	13
−7	10	5	26.4578	3.53084	7
−6	−10	5	5.15579	4.11756	1
−6	−10	5	2.95139	2.68113	10
6	10	−5	2.58652	3.67807	13
5	−10	−5	18.4063	5.28118	2
−5	−10	5	14.1522	4.89516	1
−5	10	5	14.2628	2.20435	7
4	−10	−5	4.19601	3.07338	2
−4	10	5	3.57301	1.63439	4
3	−10	−5	117.843	10.4015	2
−3	10	5	109.056	7.94937	4
2	−10	−5	62.5493	7.54911	2
1	10	−5	104.687	7.43094	4
0	−10	−5	48.6998	6.26166	2
0	10	−5	44.7142	3.61564	4
−1	−10	−5	111.362	9.56260	2
−1	−10	−5	114.107	9.87331	1
−1	10	−5	98.4835	6.54736	4
−1	10	−5	98.1082	6.56449	4
−2	−10	−5	35.0139	5.48780	2
−2	−10	−5	38.5482	6.64378	1
−3	−10	−5	46.8099	6.96648	2
−3	−10	−5	44.4099	7.21655	1
−3	10	−5	34.2457	2.36011	6
−3	10	−5	36.9633	2.84318	3
−3	10	−5	34.5706	2.34631	6
−4	−10	−5	45.4517	7.67511	1
−4	−10	−5	40.7074	5.36827	10
−4	−10	−5	44.5465	7.09994	2
−4	10	−5	43.4441	2.89913	9
−4	10	−5	40.6647	2.72160	6
−4	10	−5	41.4229	2.68209	6
−4	10	−5	42.3109	6.37763	12
−5	−10	−5	194.448	13.5713	10
−5	−10	−5	198.852	16.1308	1
−5	−10	−5	183.289	12.8616	8
−5	−10	−5	198.529	13.8095	10

−5	10	−5	175.616	11.4466	6
5	10	5	197.810	14.2501	13
−5	10	−5	179.519	11.5696	9
−5	10	−5	185.169	13.1941	12
−6	−10	−5	37.5569	5.70601	1
−6	−10	−5	31.9164	4.56388	10
−6	−10	−5	36.4951	4.15515	8
6	10	5	42.1476	6.97596	13
−6	10	−5	29.6667	1.98032	6
−6	10	−5	30.9368	2.29734	9
−6	10	−5	29.4710	2.19711	9
−6	10	−5	29.1968	1.95728	6
−7	−10	−5	6.06142	2.01900	8
−7	−10	−5	4.89785	2.06094	10
−7	10	−5	2.19096	2.27955	12
8	10	5	19.7196	3.09755	13
−9	−11	5	11.9717	2.79346	10
−9	11	5	14.4401	1.34842	9
−9	11	5	14.2656	2.07411	7
−8	−11	5	16.2487	3.40956	10
−8	11	5	11.9258	2.40713	7
−7	−11	5	53.4435	9.29316	1
−7	−11	5	58.1243	6.36162	10
−7	11	5	49.1079	4.90623	7
−6	−11	5	71.1477	9.48565	1
−6	−11	5	66.2481	6.51211	10
−6	11	5	52.6438	3.62835	11
−6	11	5	52.3207	3.69142	11
−6	11	5	47.0760	4.78406	7
−5	−11	5	13.8579	5.66934	1
5	−11	−5	9.30250	4.03054	2
−5	11	5	11.2601	2.07543	7
4	−11	−5	19.1076	4.58141	2
−4	11	5	15.1500	2.20511	4
3	−11	−5	107.256	10.0066	2
−3	11	5	98.9055	7.36360	4
2	−11	−5	85.5476	8.99761	2
1	−11	−5	158.317	13.4536	2
1	11	−5	181.599	11.4995	4
0	−11	−5	184.321	15.7686	2
0	11	−5	223.055	13.2923	4
−1	−11	−5	324.311	23.0883	2
−2	−11	−5	183.042	14.6768	2
−2	−11	−5	179.135	15.0222	1
−2	11	−5	186.166	11.2264	6
−3	−11	−5	103.947	10.4339	1
−3	−11	−5	103.379	10.2668	2
−3	11	−5	100.093	6.11318	6
−3	11	−5	99.3613	6.22885	9
−3	11	−5	95.0893	6.09244	6

−3	11	−5	96.5289	6.52457	3
−4	−11	−5	15.5700	4.97124	2
−4	−11	−5	18.0610	4.98112	1
−4	−11	−5	18.9347	3.71532	10
−4	−11	−5	17.2036	3.90716	10
−4	11	−5	31.7009	4.22049	12
−4	11	−5	24.8610	1.61387	6
−4	11	−5	25.0013	1.66565	6
−4	11	−5	26.7180	2.03080	9
−4	11	−5	26.1217	1.86032	9
−5	−11	−5	76.9195	7.64076	10
−5	−11	−5	77.5030	7.95141	10
−5	−11	−5	81.7804	10.3140	1
5	11	5	81.1979	8.66801	13
−5	11	−5	96.0139	5.46637	6
−5	11	−5	91.4330	5.44377	6
−5	11	−5	94.9433	5.58292	9
−5	11	−5	94.9194	7.67108	12
−6	−11	−5	91.1282	7.58852	8
−6	−11	−5	93.3773	8.09157	10
−6	11	−5	98.1661	6.08944	9
−6	11	−5	104.998	6.08519	9
−6	11	−5	104.799	8.13550	12
−7	−11	−5	10.6004	2.25378	8
−7	−11	−5	8.88388	2.29027	10
7	11	5	7.78956	3.37927	13
−7	11	−5	7.37190	2.84506	12
8	11	5	5.39068	2.55725	13
−9	−12	5	8.19747	2.49665	10
−9	12	5	8.33364	1.09774	9
−8	−12	5	−1.6110	2.61700	10
−8	12	5	−0.2298	1.22326	7
−8	12	5	−0.2774	0.70199	9
−8	12	5	0.60047	0.75585	6
−7	−12	5	87.7060	10.5665	1
−7	−12	5	88.1322	8.33321	10
−7	12	5	88.9579	6.91230	7
−6	−12	5	352.415	27.2785	1
−6	−12	5	342.492	23.7309	10
−6	12	5	355.001	22.4017	7
−5	−12	5	22.7332	5.83359	1
5	−12	−5	16.0048	4.94697	2
5	12	−5	25.7467	4.45834	10
−5	12	5	21.9585	2.96369	7
−5	12	5	17.8247	2.17874	11
4	−12	−5	10.0983	4.15030	2
3	−12	−5	5.27696	3.31878	2
−3	12	5	6.82851	1.86729	4
2	−12	−5	17.1276	4.34828	2
1	−12	−5	120.160	10.0621	2

1	12	−5	86.3654	7.05368	4
0	−12	−5	234.216	17.0664	2
0	12	−5	210.647	14.0804	4
−1	−12	−5	263.280	19.7090	2
−2	−12	−5	124.285	11.2959	1
−2	−12	−5	129.420	11.0070	2
−2	12	−5	99.1228	6.95285	6
−3	−12	−5	242.000	19.6105	1
−3	12	−5	252.271	15.5531	3
−3	12	−5	242.340	15.1470	6
−3	12	−5	253.250	15.2565	9
−3	12	−5	252.156	15.1616	6
−4	−12	−5	173.743	12.9691	10
−4	−12	−5	177.228	13.1878	10
−4	−12	−5	182.156	15.7284	1
−4	12	−5	166.860	10.7162	6
−4	12	−5	175.593	10.8974	9
4	12	5	155.857	13.0865	13
−4	12	−5	185.198	10.7486	6
−4	12	−5	181.651	12.5944	12
−4	12	−5	183.810	10.8286	9
−5	−12	−5	49.6178	7.66465	1
−5	−12	−5	44.2832	5.50885	10
−5	−12	−5	46.8760	5.88086	10
−5	12	−5	42.3948	5.33249	12
−5	12	−5	42.6343	2.83984	9
5	12	5	45.8269	7.38349	13
−5	12	−5	43.7697	2.89390	9
−6	−12	−5	110.345	8.70623	10
−6	−12	−5	98.4473	7.79907	8
−6	12	−5	124.925	8.74916	12
6	12	5	94.7691	9.49115	13
−7	−12	−5	15.8217	3.15663	10
−7	−12	−5	14.7100	2.21705	8
−7	12	−5	18.1336	2.90465	12
7	12	5	16.5812	4.05907	13
−9	−13	5	30.6152	3.83543	10
−8	13	5	4.72721	0.89576	9
−8	13	5	4.48782	0.67858	6
−8	13	5	4.87388	1.95862	7
−7	−13	5	−1.2044	2.68528	10
−7	13	5	5.09734	1.08179	9
−7	13	5	3.37814	2.00673	7
−6	−13	5	46.9377	8.52061	1
−6	13	5	32.1607	3.93298	7
−5	−13	5	63.6759	10.5349	1
−5	−13	5	55.3106	6.23614	13
−5	13	5	53.1471	3.91312	11
5	13	−5	56.1282	7.05635	10
−5	13	5	61.7166	5.45177	7

4	−13	−5	40.5802	7.08427	2
3	−13	−5	7.62618	4.42194	2
−3	13	5	9.26698	1.83638	4
2	−13	−5	12.1495	4.08293	2
1	−13	−5	226.920	17.7177	2
1	13	−5	232.407	15.0768	4
0	−13	−5	88.1201	9.45820	2
0	13	−5	76.4568	5.64152	4
−1	−13	−5	13.3359	4.47885	2
−2	−13	−5	95.0701	9.67783	2
−2	−13	−5	94.3132	9.65292	1
−2	13	−5	71.8461	4.89410	6
−2	13	−5	72.6970	5.14188	9
−3	−13	−5	290.776	20.1101	10
−3	−13	−5	290.056	20.2682	10
−3	−13	−5	302.472	22.6958	2
−3	−13	−5	304.591	23.0739	1
−3	13	−5	284.688	17.9964	6
−3	13	−5	291.209	18.1069	9
−3	13	−5	286.629	17.9924	6
−3	13	−5	324.982	19.8344	12
−4	−13	−5	69.4287	9.15385	1
−4	−13	−5	64.2322	6.89588	10
−4	−13	−5	65.5134	6.63951	10
4	13	5	62.4723	6.92736	13
−4	13	−5	63.8053	3.86557	6
−4	13	−5	60.7000	3.86975	6
−4	13	−5	56.1771	5.99853	14
−4	13	−5	62.9074	4.00505	9
−4	13	−5	62.0625	6.18237	12
−4	13	−5	59.4172	4.04444	9
−5	−13	−5	191.557	13.9227	10
−5	−13	−5	185.369	13.6976	10
−5	−13	−5	199.189	16.3784	1
5	13	5	177.322	14.2890	13
−5	13	−5	188.187	13.3886	12
−6	−13	−5	5.38674	2.57910	10
6	13	5	2.61507	3.79599	13
−6	13	−5	2.75959	2.54310	12
−7	−13	−5	23.5480	3.33293	10
−7	−13	−5	22.0824	2.64454	8
−7	13	−5	18.4756	2.83549	12
7	13	5	24.0405	3.80077	13
−8	−14	5	37.2745	4.90015	10
−8	14	5	35.6546	3.74167	7
−8	14	5	34.9738	2.43066	6
−8	14	5	38.3456	2.64690	9
−7	−14	5	18.4139	3.48850	10
−7	14	5	21.4484	2.96732	7
−6	−14	5	10.0186	5.17001	1

−6	14	5	18.4351	2.94172	7
6	14	−5	12.7272	4.26181	10
−5	−14	5	58.0438	6.22784	13
5	14	−5	56.7867	7.18873	10
−5	14	5	66.2416	4.16648	11
−5	14	5	63.8004	4.17439	11
4	−14	−5	71.9698	9.06588	2
−4	14	5	65.1480	4.95454	11
4	14	−5	72.1772	7.31673	10
3	−14	−5	2.94170	4.34359	2
2	−14	−5	60.1046	8.02702	2
1	−14	−5	65.3559	8.37577	2
1	14	−5	63.8040	5.15511	4
0	−14	−5	147.088	12.7479	2
0	14	−5	135.216	9.21231	4
−1	−14	−5	24.6719	5.84577	2
−1	14	−5	33.3337	2.23373	6
−2	−14	−5	43.1567	7.39123	2
−2	14	−5	59.8933	4.14519	9
−2	14	−5	68.2632	3.89098	6
−3	−14	−5	0.86244	3.75155	1
−3	14	−5	−2.5413	2.55645	12
−3	14	−5	−1.0395	0.64762	9
−3	14	−5	−0.4047	0.42894	6
−3	14	−5	−0.1838	0.36384	6
−3	14	−5	2.49023	3.10184	14
−4	14	−5	27.7134	4.60991	12
−4	14	−5	25.6648	1.93735	9
−4	14	−5	27.2779	1.98266	9
−4	14	−5	24.9175	3.69633	14
−4	14	−5	28.1587	1.72785	6
−4	14	−5	25.7489	1.71136	6
−5	−14	−5	8.40027	3.34749	10
−5	−14	−5	4.54765	4.72813	1
−5	−14	−5	12.1277	4.01528	10
−5	14	−5	9.20205	3.19503	12
−6	−14	−5	−0.4835	2.61915	10
−6	14	−5	−4.9571	2.65166	12
−7	−14	−5	4.99191	2.07728	10
−7	14	−5	3.89218	1.99715	12
−8	−15	5	7.82863	2.96722	10
−8	15	5	15.4458	1.42144	9
−8	15	5	14.9701	2.28976	7
−8	15	5	15.2814	1.14882	6
−7	15	5	18.1891	2.96028	7
−7	15	5	15.7584	1.40506	6
−7	15	5	16.6144	1.78723	9
−6	−15	5	57.4561	9.62430	1
6	15	−5	67.8062	7.78830	10
−5	−15	5	133.733	10.3267	13

5	15	−5	138.667	11.4557	10
−4	−15	5	−3.3015	2.34872	13
4	−15	−5	−0.0428	3.50622	2
4	15	−5	−1.8561	3.23431	10
−4	15	5	−1.2547	1.11035	11
3	−15	−5	6.96680	4.02525	2
3	15	−5	7.89574	3.00721	10
2	−15	−5	122.452	12.4844	2
1	−15	−5	219.012	17.8660	2
0	−15	−5	72.0235	8.84886	2
−1	−15	−5	31.5502	6.26844	2
−1	15	−5	29.6414	2.21151	6
−1	15	−5	28.2087	2.68202	9
−2	−15	−5	168.015	14.3954	2
−2	15	−5	150.297	12.0085	12
−2	15	−5	159.035	10.0849	9
−2	15	−5	161.215	9.86722	6
2	15	5	163.332	12.9024	10
−3	−15	−5	106.961	12.1354	1
−3	15	−5	107.068	6.88988	6
3	15	5	99.1641	10.1332	10
−3	15	−5	114.795	8.88866	14
−3	15	−5	115.698	7.10077	9
−3	15	−5	115.905	6.89632	6
−3	15	−5	117.089	8.90791	12
−4	−15	−5	106.851	9.10839	10
−4	−15	−5	106.829	8.84880	10
−4	−15	−5	116.084	11.9438	1
−4	15	−5	91.2887	6.04215	9
−4	15	−5	89.0574	6.06652	9
−4	15	−5	90.6962	8.19051	14
−4	15	−5	100.970	8.67588	12
−5	−15	−5	0.27415	2.78211	10
−5	15	−5	0.50770	2.63921	12
−6	−15	−5	24.4921	4.13418	10
−6	15	−5	27.1073	4.44065	12
−7	−15	−5	4.92419	1.94237	10
−8	−16	5	8.68222	2.26103	10
−8	16	5	10.9128	1.13632	9
−7	−16	5	38.0370	5.72200	10
−7	16	5	47.1113	3.34841	9
−7	16	5	53.0233	4.46261	7
−7	16	5	47.3350	3.07739	6
7	16	−5	41.6859	6.87814	10
6	16	−5	68.9075	7.98099	10
−6	16	5	62.5086	6.30535	7
−6	16	5	67.2683	4.83117	9
−5	−16	5	72.2292	6.65845	13
5	16	−5	78.8326	8.02974	10
−4	−16	5	16.5249	3.54185	13

4	16	−5	15.4357	3.85540	10
−4	16	5	13.9732	1.80339	11
3	−16	−5	7.52959	4.18117	2
−3	−16	5	4.39590	2.39342	13
−3	16	5	6.10591	1.87226	11
3	16	−5	7.66875	2.98505	10
2	−16	−5	31.0419	6.42591	2
2	16	−5	28.1904	4.67637	10
1	−16	−5	36.9537	7.18767	2
−1	16	5	33.3056	5.39935	10
0	−16	−5	39.0658	7.07467	2
0	16	5	35.8371	5.34486	10
−1	−16	−5	29.0784	6.43146	2
1	16	5	35.3379	5.76688	10
−2	−16	−5	3.19686	2.69465	13
−2	16	−5	5.74964	2.65502	12
−2	16	−5	5.46510	0.67825	6
−2	16	−5	4.67977	1.07913	9
−2	16	−5	7.82078	2.84001	14
2	16	5	7.54212	3.73394	10
−3	16	−5	142.043	8.74087	9
−3	16	−5	141.051	10.4868	14
−3	16	−5	141.358	8.54657	6
−3	16	−5	132.795	10.6938	12
3	16	5	134.251	11.9081	10
−4	−16	−5	5.47044	3.66916	10
−4	−16	−5	4.73670	3.14947	10
−4	−16	−5	3.46884	4.24757	1
−4	16	−5	3.21867	0.54409	9
−4	16	−5	4.01932	0.58006	9
−4	16	−5	3.92733	2.74309	14
−4	16	−5	2.29047	2.73636	12
−5	−16	−5	91.5173	7.69900	10
−5	16	−5	69.1582	7.20278	12
−6	−16	−5	23.2056	3.41407	10
−6	16	−5	23.2995	3.41928	12
−8	−17	5	24.4185	3.76676	10
7	17	−5	36.1356	5.78174	10
−7	17	5	27.8549	2.00189	6
−7	17	5	26.9009	2.19930	9
−5	−17	5	2.69092	2.90989	13
5	17	−5	5.23590	3.62275	10
−4	−17	5	27.0085	4.91333	13
4	17	−5	33.3310	5.53784	10
−4	17	5	28.4740	2.39313	11
−4	17	5	28.9456	2.42102	11
−3	−17	5	54.8264	6.45086	13
3	17	−5	54.0872	6.98101	10
−3	17	5	71.5876	5.05680	11
2	−17	−5	17.1523	3.18671	13

−2	−17	5	26.3334	4.02520	13
2	−17	−5	18.1372	5.46842	2
−2	17	5	18.9500	3.57019	10
1	−17	−5	78.2248	8.40285	13
1	−17	−5	98.7763	10.6340	2
1	17	−5	87.3398	7.11600	10
−1	17	5	79.2737	7.46486	10
0	−17	−5	12.2632	2.73810	13
0	17	5	12.9547	3.47271	10
0	17	−5	14.7537	1.99010	11
0	17	−5	16.2672	3.57262	14
−1	−17	−5	4.73848	2.37557	13
−1	17	−5	3.71871	1.36984	9
1	17	5	5.40639	3.33550	10
−1	17	−5	3.39397	2.65785	14
−2	−17	−5	53.6435	5.78860	13
−2	17	−5	48.1666	5.31139	12
−2	17	−5	53.5522	3.47608	6
2	17	5	60.2838	7.19931	10
−2	17	−5	48.3672	5.85071	14
−2	17	−5	55.2719	3.91291	9
−3	−17	−5	6.49969	4.75745	1
−3	17	−5	3.70368	0.93423	9
−3	17	−5	4.00652	3.33563	14
−3	17	−5	4.41467	2.61849	12
−3	17	−5	4.46785	0.53796	6
3	17	5	4.68398	3.72259	10
−4	−17	−5	50.9554	6.41625	10
−4	−17	−5	58.7730	10.7496	1
−4	17	−5	48.7394	5.93084	12
4	17	5	49.0144	7.29215	10
−5	−17	−5	38.1763	4.67456	10
−5	17	−5	43.1161	5.23937	12
−6	−17	−5	30.9858	3.58035	10
−6	17	−5	32.3223	4.22412	12
−7	18	5	5.90826	0.63200	6
−7	18	5	5.86700	0.89253	9
7	18	−5	9.42571	3.66016	10
6	18	−5	7.40105	3.94056	10
−6	18	5	14.5635	1.64985	9
−6	18	5	13.6246	1.24321	6
−5	−18	5	5.27614	2.77879	13
5	18	−5	5.22297	3.84049	10
−4	−18	5	3.95316	3.07602	13
4	18	−5	2.36766	3.21777	10
−3	−18	5	50.7195	5.62472	13
3	18	−5	54.8274	6.20438	10
2	−18	−5	59.8765	5.93242	13
−2	−18	5	73.4430	6.84293	13
2	18	−5	73.0201	6.71744	10

−2	18	5	53.4715	5.80879	10
−1	−18	5	11.1323	4.44611	13
1	−18	−5	0.63617	2.35878	13
−1	18	5	4.47642	2.65114	10
0	−18	−5	34.3544	5.19957	13
0	18	−5	29.4032	4.10908	14
0	18	−5	32.9083	3.32274	11
0	18	5	27.4614	5.15659	10
−1	−18	−5	35.9448	4.54341	13
−1	18	−5	32.6818	4.84172	14
−1	18	−5	28.3826	2.79140	9
1	18	5	34.6307	6.61479	10
−2	−18	−5	31.7492	5.13893	13
−2	18	−5	40.1372	3.08664	9
−2	18	−5	39.3899	5.25796	14
−2	18	−5	40.1156	2.58504	6
−2	18	−5	36.4002	5.04198	12
2	18	5	32.2307	6.21193	10
−3	18	−5	11.5449	0.95449	6
−3	18	−5	11.3538	1.03912	6
−3	18	−5	10.6983	3.18762	14
3	18	5	9.63749	4.29451	10
−3	18	−5	10.4104	3.08799	12
−3	18	−5	13.1476	1.49953	9
4	18	5	10.7356	4.62311	10
−4	18	−5	21.6033	3.66686	12
−5	−18	−5	2.82008	2.05793	10
−5	18	−5	7.98782	2.75561	12
−6	−18	−5	1.69850	1.53566	10
−7	19	5	15.9532	1.41414	9
7	19	−5	14.9089	3.18174	10
−6	19	5	5.69381	0.71536	6
6	19	−5	4.21134	3.63006	10
−6	19	5	7.58084	1.20468	9
−5	−19	5	18.0794	3.38131	13
5	19	−5	19.4842	4.42418	10
−4	−19	5	2.30965	3.25817	13
4	19	−5	3.93017	3.48474	10
−3	−19	5	27.1195	4.71197	13
3	19	−5	32.2781	4.87882	10
−3	19	5	19.5292	2.53371	11
2	−19	−5	58.9796	6.09529	13
−2	−19	5	51.6686	6.23844	13
2	19	−5	55.6790	6.13464	10
−2	19	5	56.4603	5.19282	10
1	−19	−5	4.95029	2.96014	13
−1	−19	5	10.8946	2.60592	13
1	19	−5	5.32580	1.91274	11
−1	19	5	8.34900	3.06640	10
0	−19	−5	81.4134	6.78909	13

0	19	−5	70.6265	5.26927	11
0	19	5	80.4762	7.47545	10
0	19	−5	67.3387	5.16502	11
−1	−19	−5	34.1787	5.33548	13
−1	19	−5	42.4834	3.74412	9
−1	19	−5	44.6305	5.24376	14
1	19	5	34.2945	5.93479	10
−2	−19	−5	29.5265	4.54496	13
−2	19	−5	29.6119	2.13793	6
−2	19	−5	30.6639	2.78821	9
2	19	5	31.1191	6.02498	10
−3	19	−5	41.9596	2.73086	6
3	19	5	31.7491	5.85458	10
−3	19	−5	40.9623	2.75794	6
−3	19	−5	50.2395	3.14536	9
−3	19	−5	43.8396	5.24876	12
4	19	5	−0.3777	3.63114	10
−4	19	−5	0.65705	2.62626	12
−5	−19	−5	16.5331	3.09996	10
7	20	−5	7.98433	2.42415	10
−6	20	5	32.1741	2.33656	9
−6	20	5	29.1391	2.04382	6
6	20	−5	24.3678	4.26579	10
−5	−20	5	6.95844	2.95824	13
5	20	−5	6.27690	3.38696	10
−4	−20	5	6.76348	2.71824	13
4	20	−5	7.33207	3.26447	10
3	−20	−5	47.3547	5.55999	13
−3	−20	5	47.3983	5.70934	13
−3	20	5	43.3160	3.48140	11
3	20	−5	47.0521	5.80151	10
−3	20	5	49.2946	3.55336	11
−2	−20	5	24.0598	3.97512	13
2	−20	−5	29.4334	4.97807	13
−2	20	5	28.3125	3.76742	11
2	20	−5	24.6972	4.14169	10
−1	−20	5	85.7253	6.84016	13
1	−20	−5	67.4870	6.62898	13
−1	20	5	63.0425	6.52303	10
1	20	−5	85.3244	5.86660	11
0	−20	−5	163.529	11.8176	13
0	20	−5	159.633	9.83712	11
0	20	5	150.997	12.2579	10
0	20	−5	156.555	9.83711	11
−1	−20	−5	3.54543	2.49699	13
−1	20	−5	1.57352	1.42597	9
1	20	5	0.03249	2.93505	10
−2	−20	−5	0.24783	3.04689	13
2	20	5	0.80673	3.60189	10
−2	20	−5	−0.0388	0.48064	6

−2	20	−5	−1.5172	1.10660	9
3	20	5	62.2615	7.76927	10
−3	20	−5	65.0972	6.31948	12
−3	20	−5	70.5255	4.21271	6
−3	20	−5	69.1431	4.23462	6
−3	20	−5	63.7949	4.55831	9
−3	20	−5	71.2671	4.53255	9
4	20	5	5.84776	3.84226	10
−5	−20	−5	1.84124	1.58184	10
5	20	5	1.34515	2.16736	10
−6	21	5	11.1678	1.22573	9
6	21	−5	14.1482	2.89563	10
−5	−21	5	25.3135	3.60891	13
5	21	−5	17.0042	3.85379	10
−4	−21	5	84.5296	7.50104	13
4	21	−5	92.2412	8.04815	10
−3	−21	5	59.1132	6.13590	13
3	−21	−5	49.4901	5.74554	13
3	21	−5	60.3228	5.99276	10
−3	21	5	54.5523	3.63098	11
−3	21	5	55.4280	3.63934	11
2	−21	−5	69.5569	7.05832	13
−2	−21	5	92.5684	7.68900	13
2	21	−5	88.6997	7.06165	10
−2	21	5	81.9493	5.99862	11
−1	−21	5	144.301	12.0912	13
1	−21	−5	187.292	12.1120	13
−1	21	5	179.987	12.1273	10
1	21	−5	154.515	11.0397	11
0	−21	−5	−2.7282	3.01506	13
0	21	5	4.71159	2.83638	10
−1	−21	−5	24.6154	4.71948	13
−1	21	−5	25.1723	2.90095	9
1	21	5	28.5242	5.35200	10
2	21	5	94.2518	8.56190	10
−2	21	−5	101.447	6.61054	9
−2	21	−5	99.2688	6.19072	6
−3	21	−5	3.88273	0.86603	9
3	21	5	10.5314	3.11961	10
−3	21	−5	5.60761	0.82042	9
4	21	5	14.9892	3.32154	10
6	22	−5	3.40079	2.54154	10
5	22	−5	29.0308	4.01816	10
−5	22	5	40.3927	2.78961	9
−4	−22	5	37.5635	5.46704	13
4	22	−5	37.5220	5.04538	10
3	−22	−5	112.064	8.36376	13
−3	−22	5	113.237	8.84720	13
3	22	−5	105.404	8.60637	10
−2	−22	5	71.2104	6.62809	13

-2	22	5	64.5235	4.82322	11
2	22	-5	59.1441	5.48497	11
1	-22	-5	12.0801	2.85264	13
-1	-22	5	9.64813	3.00998	13
-1	22	5	12.2643	2.71748	10
1	22	-5	10.7317	1.69396	11
1	22	-5	11.6475	1.73899	11
0	-22	-5	10.9476	2.93603	13
0	22	5	12.5864	3.10316	10
-1	-22	-5	17.5866	3.66892	13
1	22	5	13.4028	3.49713	10
-2	-22	-5	28.9368	4.11872	13
-2	22	-5	21.7923	2.25747	9
-2	22	-5	19.1174	1.60255	6
2	22	5	23.1731	4.04445	10
-3	22	-5	3.02838	0.58612	9
-3	22	-5	3.84971	0.55229	9
3	22	5	4.85667	2.45508	10
4	22	5	1.30186	2.05729	10
5	23	-5	2.05037	2.20332	10
-4	-23	5	14.6431	2.79601	13
4	23	-5	13.9392	2.90195	10
-3	-23	5	14.9970	3.03728	13
3	-23	-5	15.2124	3.06629	13
3	23	-5	16.7263	3.01474	10
2	-23	-5	4.68119	2.12974	13
-2	-23	5	4.53958	2.41532	13
-2	23	5	4.13046	0.95730	11
2	23	-5	3.14076	1.60399	11
-2	23	5	4.63515	0.91479	11
1	-23	-5	7.20133	2.47788	13
-1	-23	5	12.6600	3.07201	13
-1	23	5	6.36246	1.63628	11
0	-23	-5	4.25372	2.44419	13
0	23	5	5.14264	2.22230	10
-1	-23	-5	1.85842	2.57377	13
-1	23	-5	4.33189	1.37283	9
1	23	5	7.68480	2.97904	10
-2	-23	-5	18.3632	2.78620	13
-2	23	-5	14.4245	1.24044	6
2	23	5	18.1010	3.61580	10
-2	23	-5	13.3716	1.81771	9
3	23	5	0.54110	2.71590	10
4	23	5	4.92439	2.64146	10
-4	-24	5	2.21760	2.21439	13
4	24	-5	0.31420	1.60277	10
-3	-24	5	7.69794	2.36563	13
3	-24	-5	8.17845	2.46968	13
3	24	-5	7.81324	1.78302	11
-2	-24	5	-1.3567	2.47385	13

2	−24	−5	1.08128	1.94425	13
2	24	−5	1.28260	1.25565	11
2	24	−5	−0.6919	1.17203	11
−1	−24	5	21.7501	3.86741	13
1	−24	−5	24.2891	3.93387	13
−1	24	5	22.2981	2.30943	11
0	−24	−5	19.4017	3.04293	13
0	24	5	19.1915	3.26784	10
−1	−24	−5	−1.4243	1.96521	13
1	24	5	−0.4662	2.10892	10
−1	24	−5	1.84230	0.51351	6
−1	24	−5	0.06651	1.00842	9
−2	−24	−5	30.5144	3.89634	13
−2	24	−5	31.0959	2.35461	9
−2	24	−5	29.0634	1.95380	6
3	24	5	7.09691	2.57543	10
−4	−25	5	3.75530	1.55511	13
3	−25	−5	9.83412	1.94934	13
−3	−25	5	9.92290	2.20501	13
3	25	−5	8.93650	1.34730	11
2	−25	−5	0.03163	1.70894	13
−2	−25	5	−0.6344	1.79476	13
−1	−25	5	21.5649	3.28554	13
1	−25	−5	15.3444	2.55518	13
−1	25	5	16.5320	1.65874	11
−1	25	5	15.6279	1.52145	11
0	−25	−5	3.02636	2.39498	13
0	25	5	2.87145	1.20029	11
−1	−25	−5	5.69088	2.33334	13
−1	25	−5	6.17643	1.12547	9
1	25	5	5.37670	1.77867	10
2	25	5	2.00155	2.22541	10
3	−26	−5	7.72338	1.89105	13
−3	−26	5	4.42998	1.72174	13
2	−26	−5	13.6593	2.33029	13
−2	−26	5	11.0855	2.12865	13
−1	−26	5	2.40090	1.61496	13
1	−26	−5	5.10886	1.81678	13
0	−26	5	5.67084	1.65026	13
0	−26	−5	7.90623	1.92249	13
0	26	5	10.6015	1.42451	11
0	26	5	7.01677	0.99456	11
−1	−26	−5	1.12441	1.76436	13
10	0	−6	2.01066	2.70437	13
9	0	−6	1.24300	3.08971	13
−9	0	6	2.76782	1.39449	11
9	0	−6	1.72834	3.33551	13
8	0	−6	0.17038	3.17341	13
−8	0	6	0.36229	1.87342	10
−7	0	6	−0.9972	2.35278	10

7	0	−6	1.58285	3.48223	13
−6	0	6	230.483	17.2324	1
−5	0	6	97.5784	8.98429	1
−5	0	6	91.1764	7.92747	5
−4	0	6	5.39159	3.03366	5
−4	0	6	8.16853	3.34991	1
−3	0	6	110.857	10.0886	5
−3	0	6	102.884	9.20320	1
−2	0	6	32.0432	5.53021	5
0	0	−6	3804.48	231.486	5
−1	0	−6	747.683	46.9868	5
−1	0	−6	756.126	47.1355	4
−1	0	−6	780.132	47.8049	5
−2	0	−6	140.319	9.79604	4
−2	0	−6	140.352	10.3537	1
−3	0	−6	2.23532	1.67265	1
−3	0	−6	2.57670	1.50001	4
−4	0	−6	19.6521	2.95229	4
−4	0	−6	22.9803	2.74055	8
−5	0	−6	7.78783	1.82941	8
−6	0	−6	66.2642	5.19302	8
−7	0	−6	2.91428	1.25255	8
8	0	6	6.09619	2.53147	13
10	−1	−6	1.79689	3.22976	13
10	1	−6	2.03136	2.74712	13
−9	−1	6	6.50058	1.43764	11
9	−1	−6	5.65368	3.36236	13
−9	−1	6	6.34960	2.00095	10
9	1	−6	6.14205	3.66903	13
−9	1	6	6.63107	1.36048	11
9	1	−6	4.49199	3.31184	13
8	−1	−6	60.0784	7.35877	13
−8	−1	6	74.4999	6.31363	10
−8	1	6	66.2325	6.11794	10
8	1	−6	75.1786	7.92745	13
7	−1	−6	2.01369	2.99608	13
−7	−1	6	16.1421	3.26730	10
−7	−1	6	11.7424	3.70855	1
−7	1	6	7.26276	2.33678	10
7	1	−6	11.9318	3.51485	13
−6	−1	6	1.10964	3.33553	1
−5	−1	6	124.592	9.82534	5
−5	−1	6	121.290	10.4295	1
−5	1	6	101.411	9.45496	1
−5	1	6	96.2207	7.70971	5
−4	−1	6	366.035	24.0647	5
−4	−1	6	358.624	23.4724	1
−4	1	6	309.609	23.0838	1
−4	1	6	313.552	22.9640	5
−3	−1	6	258.277	19.0198	5

−3	−1	6	242.602	18.1306	1
−3	1	6	260.989	18.7870	5
−3	1	6	265.771	18.2614	1
2	−1	−6	2.41526	1.63432	2
−2	1	6	4.25585	2.79359	5
0	−1	−6	627.394	42.2490	5
0	1	−6	683.273	41.9978	5
0	1	−6	702.556	41.6682	4
−1	−1	−6	6577.12	396.995	5
−1	1	−6	6461.19	394.845	4
−2	−1	−6	1027.65	66.0548	1
−2	−1	−6	1031.19	65.3810	4
−2	1	−6	1137.78	65.2627	4
−2	1	−6	1048.28	65.5729	1
−3	−1	−6	13.4220	2.78221	1
−3	−1	−6	13.0400	2.39642	4
−3	1	−6	14.4392	2.15194	4
−4	−1	−6	172.096	12.1224	4
−4	−1	−6	172.858	11.5177	8
−4	1	−6	181.873	11.4687	8
−5	1	−6	17.2728	2.60115	8
−6	−1	−6	154.023	10.6348	8
−6	1	−6	163.726	10.4856	8
−7	−1	−6	13.7477	2.35215	8
−7	1	−6	18.8938	2.10970	8
7	1	6	14.5653	3.69466	13
8	1	6	10.1189	3.20839	13
10	−2	−6	3.39674	2.65728	13
10	2	−6	5.02783	2.78934	13
9	−2	−6	0.39186	3.09895	13
−9	−2	6	3.14509	1.74432	10
9	2	−6	4.19393	3.44400	13
−9	2	6	2.88920	1.02323	11
8	−2	−6	165.255	12.0331	13
−8	−2	6	142.979	11.3325	10
8	2	−6	150.208	12.4556	13
−8	2	6	167.000	10.9967	10
7	−2	−6	80.8027	7.83288	13
−7	−2	6	81.1647	8.69988	1
−7	−2	6	77.9531	7.10015	10
7	2	−6	68.3938	7.94519	13
−7	2	6	89.4903	6.85877	10
−6	−2	6	7.24713	3.91105	1
−5	−2	6	566.338	36.8149	1
−5	2	6	535.523	36.0295	1
−4	−2	6	278.557	21.4157	5
−4	−2	6	276.786	20.8131	1
−4	2	6	310.304	20.1745	5
−4	2	6	305.844	20.4814	1
3	−2	−6	813.177	49.5979	2

−3	−2	6	711.014	50.1375	1
−3	−2	6	737.788	51.0184	5
−3	2	6	808.429	50.1032	1
−3	2	6	859.736	50.6088	5
2	−2	−6	32.7030	4.03750	2
−2	2	6	36.1720	5.33942	5
1	−2	−6	46.9315	5.49566	5
1	−2	−6	34.3518	4.05198	2
0	−2	−6	106.798	8.02742	5
0	2	−6	83.0590	6.64952	4
0	2	−6	86.0383	6.79936	5
−1	2	−6	4.83993	1.39534	3
−1	2	−6	7.92685	1.64044	4
−2	−2	−6	114.145	7.84164	5
−2	−2	−6	113.708	9.29129	1
−2	2	−6	117.911	7.98022	4
−2	2	−6	110.819	8.17105	1
−3	2	−6	26.5590	2.71975	4
−4	−2	−6	10.3530	2.13279	4
−4	−2	−6	10.9187	1.80385	8
−4	2	−6	14.9915	1.90026	8
−4	2	−6	9.77361	1.40606	4
−5	−2	−6	271.813	17.4333	10
−5	−2	−6	274.127	17.0651	8
5	2	6	312.279	18.8813	13
−5	2	−6	227.294	17.4902	12
−5	2	−6	246.696	16.7650	8
−6	−2	−6	3.45801	1.54470	8
−6	2	−6	1.27119	1.18687	8
6	2	6	4.24598	3.86580	13
−7	−2	−6	28.7134	2.87201	8
7	2	6	26.0054	4.32912	13
−8	2	−6	16.4643	1.89165	9
8	2	6	19.2401	3.70604	13
10	−3	−6	2.44044	2.55226	13
10	3	−6	2.12096	2.62354	13
−9	−3	6	3.19880	1.91115	10
−9	3	6	3.92158	1.48169	7
9	3	−6	2.28272	3.35956	13
−9	3	6	4.36735	0.92908	11
8	−3	−6	71.3144	7.39189	13
−8	−3	6	54.2435	5.82134	10
8	3	−6	53.9349	7.40066	13
−8	3	6	59.3772	4.46665	11
7	−3	−6	22.8367	3.73356	13
−7	−3	6	11.3715	3.06081	10
−7	−3	6	17.4842	4.84729	1
7	3	−6	13.4934	4.46063	13
−7	3	6	19.8325	3.25014	10
−6	−3	6	40.9782	6.96402	1

−5	−3	6	126.778	11.4752	1
−5	−3	6	109.418	10.5277	5
−4	−3	6	84.5813	8.71857	1
−4	−3	6	84.9131	9.12704	5
−4	3	6	94.1614	7.29327	5
−3	−3	6	368.598	23.5265	1
3	−3	−6	314.516	22.3129	2
−3	3	6	320.618	22.6962	5
2	−3	−6	50.7138	5.22813	2
−2	3	6	50.2117	6.37389	5
1	−3	−6	212.661	15.1879	2
1	−3	−6	227.708	16.7082	5
−1	3	6	234.822	17.0050	5
0	3	−6	38.5236	3.39107	5
0	3	−6	42.6704	3.60751	5
0	3	−6	34.0516	3.61795	4
−1	−3	−6	1753.15	120.424	2
−1	−3	−6	1795.88	120.558	5
−1	3	−6	2162.99	119.813	4
−1	3	−6	2156.14	119.871	3
−2	−3	−6	301.277	19.6123	1
−2	−3	−6	294.348	18.6089	5
−2	3	−6	265.580	17.9666	3
−2	3	−6	259.286	17.7822	4
−3	−3	−6	49.4651	5.75779	1
−3	3	−6	53.3229	4.43977	3
−3	3	−6	56.3307	4.10179	4
−3	3	−6	52.4608	3.88903	4
−4	−3	−6	2.00228	1.31809	8
−4	−3	−6	1.00451	1.84226	1
−4	3	−6	1.94520	1.15966	8
−4	3	−6	3.76535	1.50297	3
−5	−3	−6	6.48295	2.39756	10
−5	−3	−6	0.18370	1.78562	8
−5	3	−6	8.54732	2.95894	12
−5	3	−6	4.41953	1.34088	8
5	3	6	9.27417	4.25332	13
−5	3	−6	4.24848	0.75347	11
−6	−3	−6	22.0755	3.43105	8
6	3	6	18.0786	4.15837	13
−6	3	−6	28.5105	2.56314	8
−7	−3	−6	45.1971	4.00191	8
7	3	6	36.5150	6.23991	13
−8	3	−6	0.03733	0.69267	9
8	3	6	0.63337	2.49941	13
10	−4	−6	2.71229	2.41438	13
10	4	−6	7.52764	2.83012	13
9	−4	−6	4.31090	3.22643	13
−9	−4	6	3.17226	2.17692	10
−9	4	6	2.31464	1.84949	7

9	4	−6	−1.1312	4.18669	13
−8	−4	6	7.42266	3.18569	10
8	4	−6	7.45096	3.70265	13
8	4	−6	4.96339	3.36786	13
7	−4	−6	40.3764	5.73178	13
−7	−4	6	66.6896	7.71807	1
−7	−4	6	67.7883	6.51532	10
−7	4	6	44.7779	5.06632	10
7	4	−6	68.8043	7.81396	13
−6	−4	6	25.3614	5.69111	1
−5	−4	6	24.5989	5.63895	1
−5	−4	6	30.1594	6.11608	5
4	−4	−6	83.6654	7.71279	2
−4	4	6	74.0946	5.70715	5
3	−4	−6	443.864	28.3706	2
−3	−4	6	403.067	28.9685	1
−3	4	6	437.809	28.1828	5
2	−4	−6	663.085	41.5343	2
−2	4	6	644.296	42.0564	5
1	−4	−6	358.427	24.0613	2
−1	4	6	380.508	25.3890	5
0	−4	−6	46.2583	5.15316	5
0	−4	−6	45.1941	4.77945	2
0	4	−6	43.4677	3.88200	4
0	4	−6	41.8678	3.67151	3
−1	−4	−6	2024.64	121.061	5
−1	−4	−6	2015.37	120.884	2
−1	4	−6	1899.20	119.615	4
−1	4	−6	1928.10	119.739	3
−2	−4	−6	92.1578	7.78957	5
−2	−4	−6	90.7769	8.17403	2
−2	−4	−6	86.5153	8.52050	1
−2	4	−6	98.3048	6.81466	3
−2	4	−6	103.487	6.56080	4
−3	−4	−6	234.790	17.7368	1
−3	−4	−6	250.535	18.1621	2
−3	4	−6	253.729	15.6186	4
−3	4	−6	254.808	15.5761	4
−3	4	−6	257.930	16.3491	3
−4	−4	−6	52.1199	4.68094	8
−4	−4	−6	52.4265	6.16586	1
−4	4	−6	64.2115	4.35062	8
−4	4	−6	60.7669	4.97072	3
−4	4	−6	62.7265	4.09816	9
−5	−4	−6	170.420	11.4758	8
−5	−4	−6	166.901	11.8967	10
−5	4	−6	173.596	10.9794	8
−5	4	−6	170.793	12.0172	12
−5	4	−6	181.097	10.9134	9
5	4	6	162.930	13.5347	13

−6	−4	−6	339.896	21.3230	8
−6	−4	−6	332.309	21.4651	10
−6	4	−6	318.265	20.6651	9
6	4	6	348.212	22.9893	13
−7	−4	−6	152.580	10.1835	8
7	4	6	144.929	12.5360	13
−7	4	−6	157.062	9.72369	9
−7	4	−6	151.984	9.44605	6
−8	4	−6	0.35413	0.60766	9
8	4	6	1.89521	2.51402	13
10	−5	−6	4.82212	2.40117	13
10	5	−6	6.51002	2.68005	13
−9	−5	6	9.34262	2.78337	10
−9	5	6	12.6162	2.07779	7
9	5	−6	23.0399	4.37435	13
−8	−5	6	59.1788	6.26453	10
−8	−5	6	57.2602	6.93880	1
8	−5	−6	60.5961	6.72567	13
8	5	−6	62.9134	8.78374	13
−8	5	6	53.9799	4.17717	11
−7	−5	6	17.7370	4.75053	1
−7	−5	6	20.9871	4.15148	10
7	5	−6	23.0418	4.78323	13
−6	−5	6	97.9748	8.65758	10
−6	−5	6	104.463	11.1930	1
−5	−5	6	75.6302	9.31051	1
−5	−5	6	74.8917	8.87936	5
5	−5	−6	73.6208	8.21546	2
−4	−5	6	681.211	45.8907	1
4	−5	−6	718.265	45.4012	2
3	−5	−6	302.211	21.1843	2
−3	−5	6	322.277	22.0960	1
−3	5	6	302.085	20.5597	5
2	−5	−6	494.768	32.3346	2
−2	5	6	500.948	32.5921	5
1	−5	−6	352.115	22.9190	2
1	5	−6	347.564	22.2763	5
1	5	−6	328.065	21.9702	4
−1	5	6	354.525	23.9505	5
0	−5	−6	499.734	33.7448	2
0	−5	−6	481.803	34.1264	5
0	5	−6	558.430	32.7769	3
0	5	−6	562.433	32.7240	4
−1	−5	−6	86.1371	7.14482	2
−1	−5	−6	82.7473	7.49823	5
−1	−5	−6	82.8524	7.88606	1
−1	5	−6	68.0785	5.48967	4
−1	5	−6	74.2817	5.69839	3
−2	−5	−6	20.4756	4.31405	1
−2	−5	−6	21.4160	3.89092	2

−2	−5	−6	23.0299	3.98854	5
−2	5	−6	33.5472	2.44566	4
−2	5	−6	26.6020	2.38418	4
−2	5	−6	30.0143	2.82765	3
−3	−5	−6	103.450	9.47983	1
−3	−5	−6	105.613	7.70336	5
−3	−5	−6	102.373	9.47173	2
−3	5	−6	105.375	7.46620	3
−4	−5	−6	18.8030	2.54139	8
−4	5	−6	10.9112	1.95727	3
−4	5	−6	10.2748	1.38815	9
−4	5	−6	11.1559	1.64026	8
−4	5	−6	10.9639	1.17286	6
−5	−5	−6	13.0700	2.22112	8
−5	−5	−6	14.0645	2.73819	10
5	5	6	11.0930	3.86923	13
−5	5	−6	7.46906	1.04932	6
−5	5	−6	6.13135	2.52388	12
−5	5	−6	9.07943	1.36602	9
−6	−5	−6	29.8159	3.39786	8
−6	−5	−6	25.7548	3.79407	10
−6	5	−6	25.4536	1.95993	6
6	5	6	25.0912	5.27250	13
−6	5	−6	22.5614	3.72054	12
−6	5	−6	25.7470	2.29799	9
−7	−5	−6	25.1930	3.10646	8
−7	5	−6	26.6674	2.11610	9
−7	5	−6	23.8177	1.85506	6
7	5	6	21.6873	4.34313	13
−8	5	−6	6.80761	0.85576	9
8	5	6	7.60617	2.72363	13
10	−6	−6	2.46650	2.55909	13
10	6	−6	3.15694	2.92434	13
−9	−6	6	7.59048	2.32205	10
9	−6	−6	4.94342	2.85904	13
9	6	−6	3.41187	3.48267	13
−9	6	6	1.98523	1.79283	7
8	−6	−6	73.6486	7.47718	13
−8	−6	6	65.6271	7.95897	1
−8	−6	6	64.8501	6.73682	10
−8	6	6	67.5940	5.89137	7
8	6	−6	78.2733	8.17015	13
−8	6	6	66.9776	4.74277	11
−7	−6	6	18.7836	3.73313	10
−7	−6	6	24.2908	5.45761	1
−7	6	6	21.0790	2.55637	11
7	6	−6	24.2136	4.83755	13
7	6	−6	24.8072	4.27918	13
−6	−6	6	41.4160	5.32514	10
6	−6	−6	34.4386	6.85689	2

−6	−6	6	46.2206	7.37088	1
−5	−6	6	84.5853	9.01091	1
5	−6	−6	62.0593	7.90515	2
−5	−6	6	82.9273	9.55959	5
−5	6	6	58.3857	5.46211	4
−4	−6	6	37.0010	5.97752	1
3	−6	−6	285.155	19.6822	2
−3	6	6	276.892	18.4798	5
2	−6	−6	16.2267	3.39672	2
−2	6	6	19.4217	4.20986	5
1	−6	−6	301.359	21.7524	2
1	6	−6	341.466	20.8159	4
1	6	−6	364.465	20.9041	5
−1	6	6	291.901	22.2665	5
0	6	−6	56.2370	4.44376	4
−1	6	−6	42.5263	3.47223	4
−1	6	−6	43.5228	3.47611	4
−1	6	−6	46.6460	3.76098	3
−2	6	−6	35.5197	3.11514	3
−2	6	−6	32.2601	2.53789	4
−2	6	−6	34.5506	2.57631	4
−3	−6	−6	79.5053	8.42486	2
−3	−6	−6	91.2664	8.74251	1
−3	6	−6	83.2073	6.16365	3
−4	−6	−6	40.9236	5.70046	1
−4	−6	−6	49.5426	5.69131	10
−4	−6	−6	41.2758	4.02452	8
−4	−6	−6	47.5033	6.35710	2
−4	6	−6	41.8147	3.23608	8
−4	6	−6	43.5313	3.14558	9
−4	6	−6	41.7017	2.94846	6
−5	−6	−6	285.244	21.7359	1
−5	−6	−6	281.841	18.1716	10
−5	−6	−6	270.714	17.4708	8
−5	6	−6	266.332	16.5500	6
−5	6	−6	253.953	16.6963	9
5	6	6	265.917	19.2454	13
−5	6	−6	268.530	18.0102	12
−6	−6	−6	2.12034	1.84463	10
−6	−6	−6	1.54967	1.75007	8
−6	6	−6	1.48943	0.78940	9
6	6	6	1.40690	3.49383	13
−6	6	−6	1.52347	0.53790	6
−6	6	−6	−1.5165	2.49574	12
−7	−6	−6	6.57340	1.99658	8
−7	6	−6	7.99033	0.79069	6
−7	6	−6	6.88553	1.14088	9
−8	6	−6	58.6998	3.71793	9
8	6	6	57.8596	5.77450	13
10	7	−6	−1.2305	2.23453	13

−9	−7	6	11.9438	2.67325	10
9	−7	−6	8.40451	3.47544	13
9	7	−6	7.75914	3.43130	13
−9	7	6	11.9537	2.21763	7
−8	−7	6	16.5582	3.28584	10
8	7	−6	13.2540	4.20244	13
−7	−7	6	116.364	11.8942	1
−7	−7	6	121.576	9.75249	10
7	7	−6	110.961	9.97873	13
−6	−7	6	16.4645	3.69499	10
6	−7	−6	3.04202	3.77011	2
−6	−7	6	9.79523	4.38810	1
−5	−7	6	119.807	12.5587	1
5	−7	−6	161.724	13.0353	2
−5	7	6	146.623	9.98469	4
−4	−7	6	17.5696	4.64596	1
4	−7	−6	17.0396	4.01700	2
3	−7	−6	195.514	14.4806	2
−3	7	6	185.598	12.3352	5
2	−7	−6	15.7519	3.44155	2
−2	7	6	15.6053	3.64471	5
1	−7	−6	115.612	8.98908	2
1	7	−6	92.2448	7.28023	4
1	7	−6	101.100	6.87149	5
−1	7	6	108.110	9.10299	5
1	7	−6	104.813	6.92522	5
0	−7	−6	10.5362	3.36038	2
0	7	−6	5.17679	1.28404	4
−1	−7	−6	6.14622	3.15410	1
−1	−7	−6	9.07731	3.06692	2
−1	−7	−6	9.38582	2.75495	5
−1	7	−6	5.40010	1.20957	4
−1	7	−6	4.64451	1.15247	4
−1	7	−6	5.72278	1.25252	3
−2	−7	−6	76.9027	8.17735	1
−2	−7	−6	76.5159	7.44771	2
−2	−7	−6	69.5241	7.47791	5
−2	7	−6	74.5123	5.46030	3
−3	−7	−6	307.954	22.0346	2
−3	−7	−6	286.895	21.7137	1
−3	7	−6	317.845	18.6586	6
−3	7	−6	306.280	19.4185	3
−4	−7	−6	27.7957	5.41943	2
−4	−7	−6	31.4537	5.99643	1
−4	−7	−6	25.3343	3.38160	8
−4	−7	−6	25.5198	4.20297	10
−4	7	−6	24.9331	2.18948	9
−5	−7	−6	120.257	10.2594	1
−5	−7	−6	117.265	8.99535	10
−5	−7	−6	119.852	8.36493	8

5	7	6	127.392	11.0370	13
−5	7	−6	107.971	7.37756	9
−5	7	−6	111.993	7.22143	6
−5	7	−6	110.022	8.73203	12
−6	−7	−6	64.8156	5.62042	10
−6	7	−6	50.8771	3.82261	9
−6	7	−6	53.1103	6.13469	12
−6	7	−6	51.9509	3.65223	6
6	7	6	76.6510	8.17495	13
−7	−7	−6	12.6915	2.26075	8
−7	7	−6	14.7377	1.14925	6
−7	7	−6	18.1274	1.65833	9
−7	7	−6	14.3970	1.16897	6
7	7	6	7.28557	3.82033	13
8	7	6	12.9504	2.74796	13
−9	−8	6	−0.5116	1.46397	10
−9	8	6	0.74017	1.67085	7
9	8	−6	−2.2371	3.60319	13
−8	−8	6	40.0847	5.15977	10
8	8	−6	44.6742	7.21896	13
−8	8	6	31.3141	4.00725	7
−7	−8	6	201.428	15.2051	10
−7	−8	6	219.586	17.8555	1
−7	8	6	214.304	14.1421	7
−7	8	6	213.595	13.2222	11
7	8	−6	205.107	15.5133	13
−6	−8	6	0.68357	3.70766	1
6	−8	−6	1.12515	3.40083	2
−6	−8	6	2.32436	2.55704	10
−6	8	6	3.14335	1.32308	11
5	−8	−6	13.4867	4.29982	2
−5	8	6	12.5402	2.17880	4
4	−8	−6	143.573	12.2506	2
−4	−8	6	165.593	13.3708	1
−4	8	6	126.906	9.85576	4
3	−8	−6	79.8958	8.05058	2
2	−8	−6	80.7317	7.77035	2
−2	8	6	77.3468	6.76373	5
1	−8	−6	128.458	10.7939	2
−1	8	6	127.635	10.6085	5
1	8	−6	128.845	8.86184	4
0	−8	−6	12.6408	3.42432	2
0	8	−6	15.2862	2.03011	4
0	8	6	12.9038	3.52356	5
−1	−8	−6	6.54342	2.63138	2
−1	−8	−6	3.28005	2.56835	1
−1	8	−6	2.52688	0.88800	4
−1	8	−6	3.03337	0.94854	4
−1	8	−6	3.13073	0.92417	4
−2	−8	−6	330.545	23.4681	1

−2	−8	−6	334.017	23.0696	2
−2	−8	−6	316.257	23.1631	5
−2	8	−6	335.204	20.8033	3
−3	−8	−6	15.5438	4.45518	1
−3	−8	−6	15.2171	4.38576	2
−3	8	−6	10.8068	1.09119	6
−3	8	−6	12.5286	1.91783	3
−4	−8	−6	54.6939	5.40689	8
−4	−8	−6	53.1622	6.21472	10
−4	−8	−6	54.8903	7.41922	1
−4	−8	−6	53.6002	7.58950	2
−4	8	−6	69.0109	3.98271	6
−4	8	−6	72.5739	6.02792	12
−4	8	−6	62.0815	4.09436	9
−5	−8	−6	1.59102	1.83998	8
−5	−8	−6	0.95357	2.60087	1
−5	−8	−6	1.87350	2.27347	10
−5	8	−6	2.25305	0.62267	6
−5	8	−6	3.06354	0.80876	9
−5	8	−6	2.84793	2.36139	12
5	8	6	0.33281	4.25843	13
−6	−8	−6	65.8699	6.36318	10
−6	−8	−6	66.0690	6.04760	8
−6	8	−6	77.3453	6.59992	12
−6	8	−6	82.1558	4.91485	9
−6	8	−6	76.9484	4.71495	6
−6	8	−6	78.5020	4.71483	6
6	8	6	64.0904	8.22993	13
−7	−8	−6	56.6824	4.86001	8
−7	−8	−6	49.5292	4.71405	10
7	8	6	65.7635	6.99784	13
−7	8	−6	57.1556	3.49863	6
−7	8	−6	56.5939	3.49846	6
−7	8	−6	58.5463	3.70991	9
8	8	6	12.2887	2.61867	13
−9	−9	6	5.65967	2.42336	10
−9	9	6	8.70143	1.94676	7
9	9	−6	0.66106	2.81711	13
−8	−9	6	33.8784	5.16855	10
−8	9	6	35.4667	4.09861	7
8	9	−6	44.3493	6.35406	13
−7	−9	6	75.0186	7.31012	10
−7	−9	6	76.6133	10.3832	1
7	9	−6	72.0971	8.26966	13
−6	−9	6	41.4692	8.02096	1
−6	−9	6	51.3070	6.16923	10
6	9	−6	54.3923	6.69683	13
5	−9	−6	20.4402	4.98227	2
−5	−9	6	13.5745	4.82435	1
−5	9	6	16.6813	2.54607	7

4	−9	−6	29.1104	5.68037	2
−4	9	6	23.9952	3.18806	4
3	−9	−6	267.168	19.8171	2
2	−9	−6	40.9800	6.21503	2
1	−9	−6	270.942	18.3275	2
−1	9	6	260.391	17.8066	5
1	9	−6	223.377	16.1700	4
0	−9	−6	72.5060	7.69996	2
0	9	6	67.3565	7.40264	5
0	9	−6	59.3986	4.82908	4
−1	−9	−6	14.1523	3.95585	1
−1	−9	−6	18.0088	3.92805	2
−1	9	−6	15.5754	1.66536	4
−1	9	−6	14.9797	1.64751	4
−2	−9	−6	24.7825	4.82516	2
−2	−9	−6	20.9751	4.65209	1
−2	−9	−6	20.3814	4.64149	5
−3	−9	−6	408.844	27.3420	1
−3	−9	−6	415.828	27.5245	2
−3	9	−6	367.430	23.3070	6
−3	9	−6	352.529	23.3938	9
−4	−9	−6	50.9817	7.38800	1
−4	−9	−6	49.2574	5.58467	10
−4	−9	−6	47.2513	7.89277	2
−4	−9	−6	51.9624	4.75824	8
4	9	6	50.2281	6.64552	13
−5	−9	−6	63.9672	6.85718	10
−5	−9	−6	64.6083	6.28940	8
−5	−9	−6	68.5746	8.71778	1
−5	9	−6	87.3158	4.96284	6
5	9	6	62.3704	8.13337	13
−5	9	−6	88.3542	5.16631	9
−5	9	−6	90.7330	7.51688	12
−5	9	−6	88.7063	4.99643	6
−6	−9	−6	100.522	7.86284	10
−6	−9	−6	98.2567	7.49178	8
−6	9	−6	96.0307	6.24024	9
−6	9	−6	99.1516	6.09418	6
−6	9	−6	95.6166	6.07470	6
6	9	6	106.605	10.3668	13
−6	9	−6	99.7030	7.88004	12
−7	−9	−6	25.8521	2.90930	8
−7	−9	−6	27.1220	3.32247	10
−7	9	−6	29.4130	1.82188	9
−7	9	−6	24.2631	3.79031	12
7	9	6	25.1237	4.31634	13
−7	9	−6	27.0547	1.80300	9
8	9	6	16.4465	2.73595	13
−9	−10	6	7.64717	2.50383	10
−9	10	6	3.67932	1.72881	7

−8	−10	6	4.73753	2.86823	10
−8	10	6	19.0513	3.03596	7
−7	−10	6	6.19194	4.50933	1
−7	−10	6	2.31789	2.80483	10
−7	10	6	9.44816	2.25758	7
−6	−10	6	208.720	19.3552	1
−6	−10	6	196.776	15.9799	10
−6	10	6	247.802	14.9555	7
−6	10	6	240.659	14.1882	11
5	−10	−6	12.8380	4.59536	2
4	−10	−6	9.89818	3.94303	2
−4	10	6	8.15149	1.75493	4
3	−10	−6	361.713	25.5655	2
2	−10	−6	523.023	34.8951	2
1	10	−6	247.217	16.1316	4
0	−10	−6	3.01386	3.34817	2
0	10	−6	1.15864	1.02544	4
0	10	6	4.14269	2.61552	5
−1	−10	−6	10.7217	3.82122	1
−1	−10	−6	14.2258	3.79104	2
−1	10	−6	19.5032	1.61106	4
−1	10	−6	19.2204	1.62556	4
−2	−10	−6	16.9706	4.01087	2
−2	−10	−6	18.8732	4.61679	1
−2	10	−6	11.1290	1.11021	6
−3	−10	−6	6.21799	3.87563	2
−4	−10	−6	383.567	24.9290	10
−4	−10	−6	419.593	27.9293	1
4	10	6	397.404	25.2862	13
−4	10	−6	397.979	24.5737	12
−4	10	−6	346.701	23.1224	9
−4	10	−6	354.509	23.0031	6
−4	10	−6	365.706	23.0420	6
−5	−10	−6	30.1620	6.42522	1
−5	−10	−6	26.1322	4.28390	10
−5	10	−6	12.7622	3.06198	12
−5	10	−6	16.0585	1.30183	6
−5	10	−6	15.9894	1.23690	6
−5	10	−6	15.4657	1.50708	9
−6	−10	−6	18.6437	3.19444	10
−6	−10	−6	16.7696	2.77299	8
−6	10	−6	11.6828	1.35901	9
−6	10	−6	11.3959	1.22310	9
−6	10	−6	6.07908	2.78501	12
−6	10	−6	10.4928	0.84919	6
−7	−10	−6	2.15555	1.65620	10
−7	−10	−6	1.54483	1.82679	8
−7	10	−6	2.32290	2.03087	12
7	10	6	2.40358	2.98068	13
−9	−11	6	1.65454	1.59690	10

−9	11	6	0.67349	1.48083	7
−8	−11	6	3.53221	2.70806	10
−8	11	6	2.55576	1.82426	7
−7	−11	6	37.9955	5.57487	10
−7	−11	6	43.7652	7.79448	1
−7	11	6	29.2111	3.90555	7
−6	−11	6	138.748	13.6919	1
−6	−11	6	127.079	10.4568	10
−6	11	6	118.818	8.09806	11
−6	11	6	127.081	9.01689	7
5	−11	−6	73.4831	9.76827	2
−5	−11	6	56.5920	8.90090	1
5	11	−6	57.4365	6.92239	10
−5	11	6	65.3673	4.69746	11
4	−11	−6	38.0007	6.62934	2
−4	11	6	29.1528	3.49803	4
3	−11	−6	117.140	11.0582	2
2	−11	−6	32.9477	5.87955	2
1	−11	−6	511.438	36.6516	2
1	11	−6	591.593	34.6272	4
0	−11	−6	35.0331	6.57837	2
0	11	−6	44.0133	3.48302	4
−1	−11	−6	137.410	12.8327	1
−1	−11	−6	143.441	12.2369	2
−2	−11	−6	32.6840	6.21814	1
−2	−11	−6	32.0095	5.66289	2
−2	11	−6	24.9620	1.94999	6
−2	11	−6	29.8061	2.41403	9
−3	−11	−6	32.7587	5.37779	10
−3	−11	−6	34.9152	6.63402	1
−3	−11	−6	33.7376	6.19012	2
−3	11	−6	18.2837	1.62961	6
−3	11	−6	21.9794	1.95019	9
−4	−11	−6	6.49658	4.02406	1
−4	−11	−6	3.63261	2.68762	10
4	11	6	4.95220	4.04978	13
−4	11	−6	5.53336	0.69489	6
−4	11	−6	6.25642	0.63849	6
−4	11	−6	9.74612	2.78414	12
−4	11	−6	7.14368	0.93690	9
−5	−11	−6	240.467	18.2954	8
−5	−11	−6	257.575	18.0299	10
−5	−11	−6	276.269	20.4438	1
−5	11	−6	260.207	16.0006	6
−5	11	−6	271.431	17.7077	12
−5	11	−6	270.241	16.0245	6
−5	11	−6	267.218	16.1261	9
5	11	6	261.390	18.8630	13
−6	−11	−6	10.3972	2.76492	10
−6	−11	−6	12.7757	2.52785	8

6	11	6	11.9493	3.74146	13
−6	11	−6	9.17776	2.82234	12
−7	−11	−6	0.33072	1.67094	8
−7	−11	−6	1.61228	1.53465	10
−7	11	−6	0.28543	1.81577	12
7	11	6	5.58806	2.76670	13
−9	−12	6	0.72693	2.05311	10
−7	−12	6	88.5871	8.19279	10
−7	−12	6	83.7588	10.4762	1
−7	12	6	85.4600	6.70255	7
−6	−12	6	34.1221	7.41785	1
−6	12	6	41.6000	3.11246	11
−6	12	6	44.4489	4.25413	7
−6	12	6	41.7978	3.05512	11
−5	−12	6	37.7499	8.67708	1
−5	−12	6	29.5945	4.81471	13
5	−12	−6	27.0296	6.66081	2
5	12	−6	36.6352	5.92031	10
−5	12	6	19.5519	2.58601	11
4	−12	−6	99.5442	10.7119	2
3	−12	−6	101.855	10.4191	2
2	−12	−6	10.3865	3.98571	2
1	−12	−6	154.415	13.7525	2
1	12	−6	175.586	11.3225	4
0	−12	−6	165.072	12.8263	2
0	12	−6	127.835	9.37307	4
−1	−12	−6	71.5819	8.28359	2
−2	−12	−6	6.12034	4.19181	1
−2	−12	−6	3.76536	3.19805	2
−2	12	−6	5.86191	0.68455	6
−2	12	−6	5.91852	0.96017	9
−3	−12	−6	52.0800	7.95232	1
−3	−12	−6	47.8741	5.76518	10
−3	12	−6	50.0569	6.16294	12
−3	12	−6	50.6650	3.44203	9
−3	12	−6	48.4147	3.20224	6
−4	−12	−6	32.9189	4.82952	10
−4	−12	−6	35.4908	7.03048	1
−4	12	−6	33.6420	5.41654	12
4	12	6	26.3869	4.29497	13
−4	12	−6	23.3910	1.71592	6
−4	12	−6	24.1750	1.93540	9
−4	12	−6	21.4025	1.66150	6
−5	−12	−6	302.617	20.9817	10
−5	−12	−6	312.624	23.1157	1
−5	12	−6	312.298	20.6009	12
−5	12	−6	323.316	18.9548	9
−5	12	−6	316.862	18.9911	9
−5	12	−6	320.129	18.8334	6
−5	12	−6	304.129	18.8287	6

5	12	6	292.324	21.1587	13
−6	−12	−6	4.07543	2.17001	8
−6	−12	−6	7.86560	2.45943	10
6	12	6	8.03260	3.28917	13
−6	12	−6	5.33473	2.57979	12
−7	−12	−6	2.73199	1.59645	10
−7	12	−6	1.61174	1.84955	12
7	12	6	4.58254	2.35834	13
−9	−13	6	13.0304	2.62288	10
−8	−13	6	18.6147	3.54235	10
−8	13	6	15.0062	2.43045	7
−6	13	6	15.3464	1.34752	11
−6	13	6	19.3973	3.16066	7
−6	13	6	15.9293	1.40463	11
−5	−13	6	55.3561	5.38019	13
5	13	−6	53.8920	6.55730	10
−5	13	6	44.6460	3.77381	11
4	−13	−6	17.7353	5.14503	2
−4	13	6	18.5409	3.27127	10
4	13	−6	13.7155	3.65520	10
3	−13	−6	70.8573	8.84395	2
2	−13	−6	57.6708	7.91459	2
1	−13	−6	47.5482	7.65644	2
1	13	−6	62.3918	4.88283	4
0	−13	−6	54.4782	7.43616	2
0	13	−6	54.9462	4.21094	4
−1	−13	−6	4.92974	3.39812	2
−1	13	−6	17.2892	1.90753	9
−2	−13	−6	4.23087	4.18145	1
−2	−13	−6	4.39086	3.98995	2
−2	13	−6	6.05890	0.69091	6
2	13	6	0.02560	4.08466	10
−3	−13	−6	0.99411	2.80212	10
−3	−13	−6	1.80239	3.75372	1
−3	13	−6	3.40016	0.54261	6
−3	13	−6	5.79708	2.70965	12
−3	13	−6	1.43288	2.89017	14
−3	13	−6	2.38897	0.86525	9
−4	−13	−6	104.808	11.0170	1
−4	−13	−6	103.404	8.38888	10
−4	13	−6	90.1792	7.74192	14
−4	13	−6	87.9554	7.74612	12
−4	13	−6	90.2769	5.82162	9
−4	13	−6	91.6128	5.92225	9
−4	13	−6	88.4031	5.67940	6
4	13	6	96.0889	9.04416	13
−4	13	−6	86.9131	5.66666	6
−5	−13	−6	56.9387	5.71667	10
5	13	6	56.8170	7.60597	13
−5	13	−6	58.8902	6.41604	12

−6	−13	−6	2.03360	1.73200	10
−6	13	−6	2.64309	2.46514	12
6	13	6	0.11526	2.80673	13
−7	−13	−6	1.30885	1.47383	10
−7	13	−6	0.96535	1.69329	12
−8	−14	6	23.7780	3.59595	10
−8	14	6	24.7794	1.84922	6
−8	14	6	25.7823	2.07360	9
−8	14	6	25.4758	3.09307	7
−7	−14	6	8.47444	3.29462	10
−7	14	6	5.41391	1.96460	7
6	14	−6	75.0262	8.30616	10
−5	−14	6	102.961	8.26697	13
−5	14	6	103.131	6.96311	11
5	14	−6	113.441	9.68537	10
−4	−14	6	52.8741	5.50033	13
4	−14	−6	53.5406	8.73421	2
4	14	−6	52.8237	6.44940	10
−4	14	6	45.4998	4.09084	11
3	−14	−6	4.96381	3.90555	2
3	14	−6	8.26726	2.85254	10
2	−14	−6	20.9386	5.34275	2
0	−14	−6	22.3211	5.37482	2
−1	−14	−6	151.371	13.1278	2
−1	14	−6	139.730	8.75980	11
1	14	6	142.276	14.4372	10
−1	14	−6	128.789	8.85082	9
−2	−14	−6	123.065	11.9276	1
−2	−14	−6	126.537	11.6198	2
−2	14	−6	102.798	7.10651	9
−2	14	−6	102.993	8.84219	14
−2	14	−6	106.306	6.86077	6
−2	14	−6	99.6468	8.33340	12
2	14	6	128.588	10.9566	10
−3	−14	−6	33.3327	5.11873	10
−3	−14	−6	27.1066	6.39285	1
−3	14	−6	30.7065	4.79330	14
−3	14	−6	27.7404	4.49697	12
−3	14	−6	29.6101	2.09488	6
−3	14	−6	30.7782	2.10045	6
−3	14	−6	32.8817	2.41485	9
−4	−14	−6	120.787	12.7051	1
−4	−14	−6	130.792	10.2866	10
−4	14	−6	122.524	10.0980	14
−4	14	−6	118.845	7.61077	6
−4	14	−6	124.548	7.82103	9
−4	14	−6	124.445	7.62583	6
−4	14	−6	124.802	9.72841	12
−4	14	−6	129.355	7.79582	9
−5	−14	−6	51.3758	5.65977	10

−5	14	−6	70.6569	6.36686	12
−6	−14	−6	3.00094	1.75090	10
−6	14	−6	4.18003	2.42199	12
−7	−14	−6	1.17658	1.46484	10
−7	14	−6	0.91757	1.61572	12
−8	−15	6	17.5240	3.34804	10
−8	15	6	19.9638	1.69043	9
−8	15	6	20.0809	2.78813	7
−7	−15	6	63.7156	6.32186	10
7	15	−6	70.5862	6.99452	10
−7	15	6	74.9558	5.98520	7
6	15	−6	73.0363	8.32104	10
−5	−15	6	124.416	10.1394	13
−5	15	6	138.596	8.31692	11
−5	15	6	135.395	8.31030	11
5	15	−6	117.488	10.8581	10
−4	−15	6	6.73569	2.66721	13
4	15	−6	7.45002	3.45196	10
−3	−15	6	29.7358	4.54816	13
3	−15	−6	38.9272	7.29333	2
3	15	−6	36.5281	5.71919	10
−3	15	6	36.7143	4.25290	10
2	−15	−6	31.3009	6.39510	2
−2	15	6	22.8765	4.30914	10
2	15	−6	25.0627	4.33566	10
1	−15	−6	23.7141	5.88427	2
−1	15	6	17.4143	3.52730	10
0	−15	−6	8.82224	4.36036	2
0	15	−6	12.6787	2.08644	11
0	15	6	8.19182	3.50095	10
−1	−15	−6	12.1267	4.34988	2
1	15	6	11.2241	3.98244	10
−1	15	−6	7.59401	1.29023	9
−2	−15	−6	6.65012	4.98636	1
−2	15	−6	8.26012	1.26545	9
−2	15	−6	4.60154	0.68222	6
−2	15	−6	11.8664	2.83845	12
−2	15	−6	15.1360	3.14965	14
−3	15	−6	129.496	7.96017	6
−3	15	−6	125.125	10.1001	12
−3	15	−6	123.683	7.95695	6
−3	15	−6	136.277	8.20099	9
−3	15	−6	129.152	9.85533	14
−4	−15	−6	17.7373	4.11038	10
−4	−15	−6	11.8573	4.96566	1
−4	15	−6	16.5148	3.43475	14
−4	15	−6	12.3129	1.25248	9
−4	15	−6	10.5961	1.20468	9
−4	15	−6	15.2741	3.94973	12
−5	−15	−6	33.2048	4.36900	10

−5	15	−6	43.0308	5.17200	12
−6	−15	−6	75.6594	6.17369	10
−6	15	−6	61.8952	6.00178	12
−8	−16	6	−0.0019	1.57442	10
7	16	−6	61.4702	6.62886	10
−7	16	6	68.2573	4.54244	9
6	16	−6	60.6011	7.40685	10
−5	−16	6	260.153	18.0280	13
5	16	−6	273.536	19.2150	10
−5	16	6	268.234	16.5199	11
−5	16	6	278.932	16.5277	11
−4	−16	6	3.63460	2.53960	13
4	16	−6	4.88090	3.30745	10
−4	16	6	6.18606	1.48053	11
3	−16	−6	20.6324	3.43393	13
−3	−16	6	11.0529	3.02886	13
3	16	−6	14.8255	3.56812	10
−3	16	6	20.8627	3.28930	10
−2	−16	6	23.4004	3.67969	13
2	−16	−6	29.1176	6.32974	2
2	−16	−6	29.1504	4.77505	13
2	16	−6	26.9029	4.33045	10
−2	16	6	32.6084	4.79029	10
1	−16	−6	62.3627	9.54001	2
1	16	−6	43.0592	4.85003	10
−1	16	6	60.6314	6.20758	10
0	−16	−6	6.70375	2.83320	13
0	−16	−6	0.25350	3.42683	2
0	16	−6	−0.2301	3.30812	14
0	16	−6	2.10620	1.31742	11
0	16	6	4.75362	3.00359	10
−1	−16	−6	20.7221	3.69635	13
1	16	6	21.8838	4.73560	10
−1	16	−6	26.0438	3.63244	14
−1	16	−6	28.5621	2.46351	9
−2	−16	−6	27.2749	4.97754	13
−2	16	−6	41.5050	5.85794	14
−2	16	−6	39.7203	2.62141	6
−2	16	−6	40.6052	3.03715	9
2	16	6	42.0820	6.59082	10
−2	16	−6	38.7632	4.75385	12
−3	−16	−6	42.9665	8.00842	1
−3	16	−6	30.3882	4.66641	12
3	16	6	42.6953	6.83506	10
−3	16	−6	29.0569	2.46185	9
−3	16	−6	31.9117	4.20457	14
−3	16	−6	30.8869	2.14839	6
−3	16	−6	30.4445	2.09002	6
−4	−16	−6	5.82015	3.29612	10
−4	16	−6	3.34382	2.86373	12

−4	16	−6	2.15429	0.52189	9
−4	16	−6	6.46223	2.99815	14
−4	16	−6	1.97518	0.56060	9
−5	−16	−6	57.2811	5.97619	10
−5	16	−6	41.3292	4.78676	12
−6	−16	−6	5.39917	2.37008	10
−6	16	−6	6.24389	2.31873	12
−7	17	6	1.94770	0.55630	6
7	17	−6	−0.5198	2.46816	10
−7	17	6	1.55248	0.90783	9
6	17	−6	41.7019	5.30080	10
−5	−17	6	84.1887	7.62217	13
5	17	−6	89.6032	8.86209	10
−4	−17	6	30.4383	4.89616	13
4	17	−6	32.6096	5.45866	10
3	−17	−6	21.9878	3.52155	13
−3	−17	6	19.7292	3.84249	13
3	17	−6	24.7110	4.81418	10
−3	17	6	20.9257	3.61248	10
2	−17	−6	41.8318	5.25272	13
−2	−17	6	34.1870	5.00092	13
−2	17	6	33.1627	4.60144	10
2	17	−6	39.8948	4.79630	10
1	17	−6	11.9322	2.95767	14
−1	17	6	11.3868	3.31448	10
1	17	−6	15.0375	2.70544	11
0	−17	−6	6.87502	2.64605	13
0	17	−6	9.15153	3.01884	14
0	17	−6	10.4126	1.74178	11
0	17	6	13.3968	4.16151	10
−1	−17	−6	216.184	15.6869	13
−1	17	−6	237.250	16.0329	14
1	17	6	236.340	17.0383	10
−1	17	−6	237.728	14.5860	9
−2	17	−6	27.4008	4.36629	12
−2	17	−6	30.5523	2.67495	9
−2	17	−6	31.3695	4.98740	14
−2	17	−6	31.8840	2.14312	6
−3	−17	−6	1.28009	4.32794	1
−3	17	−6	3.87254	2.95021	14
−3	17	−6	6.53906	1.05276	9
−3	17	−6	5.93790	2.69776	12
3	17	6	−1.5996	3.79237	10
−3	17	−6	4.22466	0.61228	6
−3	17	−6	5.64042	0.59805	6
−4	−17	−6	66.6404	7.27476	10
−4	17	−6	80.1443	6.89263	12
−5	−17	−6	3.42995	2.09831	10
−5	17	−6	3.57875	2.61692	12
−6	−17	−6	32.3803	3.86648	10

−6	17	−6	34.0815	3.31752	12
−7	18	6	9.71117	1.19175	9
7	18	−6	9.49871	3.47350	10
6	18	−6	0.46017	3.69130	10
−5	−18	6	60.3502	6.03345	13
5	18	−6	64.1274	7.18770	10
−4	−18	6	278.062	19.3468	13
4	18	−6	292.860	20.0433	10
−4	18	6	284.431	17.9343	11
−4	18	6	304.801	18.0803	11
3	−18	−6	23.2870	3.56266	13
−3	−18	6	16.4984	3.28852	13
−3	18	6	25.6813	2.80696	11
3	18	−6	20.2463	3.89325	10
−2	−18	6	51.5234	5.87671	13
2	−18	−6	62.7321	6.25342	13
2	18	−6	52.6869	6.07473	10
−2	18	6	62.5687	5.74159	10
−1	−18	6	54.9184	5.74771	13
1	18	−6	51.3410	5.55834	14
1	18	−6	48.0949	4.95577	11
0	−18	−6	57.0397	5.77982	13
0	18	−6	54.3204	4.20209	11
0	18	6	63.1468	6.85040	10
0	18	−6	60.8744	6.09299	14
0	18	−6	54.6215	4.24332	11
−1	−18	−6	105.456	8.27186	13
1	18	6	110.342	9.43131	10
−1	18	−6	90.1204	6.53756	9
−1	18	−6	79.4509	7.85315	14
−2	−18	−6	111.959	7.89458	13
−2	18	−6	91.9869	6.18039	6
−2	18	−6	95.9866	8.34248	14
−2	18	−6	96.0185	6.55021	9
3	18	6	25.8691	5.25750	10
−3	18	−6	21.8633	4.41336	14
−3	18	−6	21.5531	1.61068	6
−3	18	−6	26.0289	2.08502	9
−3	18	−6	20.6729	3.59181	12
−4	−18	−6	9.21493	2.66916	10
−4	18	−6	8.36341	2.83811	12
4	18	6	9.09317	3.25355	10
−5	−18	−6	8.05057	2.25172	10
−5	18	−6	12.6230	2.68654	12
7	19	−6	2.36530	2.12529	10
6	19	−6	17.2879	4.01934	10
−5	−19	6	54.3020	5.70896	13
5	19	−6	58.2504	6.67535	10
−4	−19	6	54.5496	6.11477	13
−4	19	6	53.2075	3.50464	11

−4	19	6	53.9502	3.51451	11
4	19	−6	47.7096	6.51612	10
−3	−19	6	43.2269	5.31639	13
3	−19	−6	43.2294	4.85017	13
−3	19	6	31.9485	3.37770	11
3	19	−6	42.3164	5.27930	10
2	−19	−6	18.6063	3.52722	13
−2	−19	6	25.4662	4.44334	13
−2	19	6	15.8722	3.01745	10
2	19	−6	24.3615	3.90478	10
1	−19	−6	53.1876	5.78183	13
−1	−19	6	60.2587	5.91370	13
−1	19	6	53.0549	6.03601	10
1	19	−6	66.0085	5.17654	11
0	−19	−6	16.1196	3.47215	13
0	19	6	11.8297	3.50365	10
0	19	−6	13.7729	1.69280	11
0	19	−6	12.1513	3.07016	14
0	19	−6	13.7218	1.70329	11
−1	−19	−6	52.5873	6.44566	13
−1	19	−6	68.3203	4.49866	9
1	19	6	47.4568	6.98405	10
−1	19	−6	65.8616	6.40276	14
−2	−19	−6	52.6873	6.51573	13
−2	19	−6	67.8854	4.16553	6
−2	19	−6	66.8895	4.64366	9
2	19	6	59.0090	7.68006	10
−2	19	−6	73.5427	7.02268	14
−3	19	−6	28.4044	1.87950	6
3	19	6	21.2556	4.98635	10
−3	19	−6	29.2234	1.86806	6
−4	19	−6	−0.2002	2.51158	12
4	19	6	4.98398	2.78718	10
−5	−19	−6	5.48806	2.34041	10
−6	20	6	19.3822	1.92670	9
6	20	−6	31.3331	4.82895	10
−5	−20	6	10.5308	3.24559	13
5	20	−6	7.37763	3.50921	10
−4	−20	6	−0.3950	2.59229	13
4	20	−6	−1.1190	2.87225	10
3	−20	−6	79.2549	7.56949	13
−3	−20	6	83.4006	7.58027	13
3	20	−6	78.6037	7.43228	10
−3	20	6	84.6685	5.80941	11
2	−20	−6	66.9089	6.35505	13
−2	−20	6	66.8899	7.15870	13
2	20	−6	64.3448	5.95092	10
−2	20	6	68.1716	5.87226	10
−1	−20	6	47.9239	6.15214	13
1	−20	−6	63.6811	5.92572	13

-1	20	6	63.6168	6.03484	10
1	20	-6	54.2040	4.83391	11
0	-20	-6	11.7531	2.94576	13
0	20	6	18.4266	3.83611	10
-1	-20	-6	25.6845	4.58426	13
-1	20	-6	20.2343	2.47122	9
1	20	6	29.3845	4.90620	10
-2	20	-6	36.5684	2.92278	9
-2	20	-6	31.0751	2.24750	6
-3	20	-6	13.7254	2.96297	12
-3	20	-6	13.1372	1.30791	9
3	20	6	9.82348	3.35556	10
4	20	6	11.2464	3.66827	10
6	21	-6	13.9791	3.30425	10
-5	-21	6	2.17452	2.56502	13
5	21	-6	1.72675	2.03324	10
-4	-21	6	13.3342	3.05767	13
4	-21	-6	13.9713	3.17947	13
4	21	-6	8.43154	3.13340	10
-3	-21	6	-1.3504	2.56634	13
3	-21	-6	0.31534	2.28234	13
-3	21	6	0.30351	0.89236	11
-3	21	6	0.86209	0.89925	11
-2	-21	6	14.9659	3.10568	13
2	-21	-6	13.1001	2.82599	13
-2	21	6	13.4600	2.00453	11
2	21	-6	15.8742	2.79039	11
1	-21	-6	18.4927	3.27907	13
-1	-21	6	11.5391	3.33343	13
-1	21	6	20.4766	3.63762	10
1	21	-6	18.6127	2.37343	11
1	21	-6	28.4764	2.96723	11
0	-21	-6	33.3302	4.84701	13
0	21	6	31.5393	5.03829	10
-1	-21	-6	2.93607	2.60312	13
1	21	6	-2.6511	2.93718	10
-1	21	-6	0.15022	1.15553	9
2	21	6	9.22493	2.86838	10
-2	21	-6	7.44642	1.14821	9
-2	21	-6	6.55663	0.74319	6
3	21	6	3.56765	3.33657	10
4	21	6	7.87601	2.51623	10
6	22	-6	3.58801	2.34901	10
-5	-22	6	7.39408	2.45191	13
5	22	-6	7.75114	2.76008	10
-4	-22	6	10.4292	2.60107	13
4	-22	-6	5.77185	2.27216	13
4	22	-6	5.89774	2.61491	10
-3	-22	6	2.64010	2.60141	13
3	-22	-6	3.26150	2.11790	13

2	−22	−6	1.80225	2.24640	13
−2	−22	6	1.79312	2.75931	13
2	22	−6	0.39121	1.57890	11
−2	22	6	2.08670	1.17179	11
−1	−22	6	134.373	9.47205	13
1	−22	−6	120.034	9.38291	13
−1	22	6	105.719	9.01427	10
1	22	−6	132.144	7.75081	11
1	22	−6	129.603	7.74390	11
0	−22	−6	42.5924	4.97080	13
0	22	6	36.3715	4.87531	10
−1	−22	−6	15.6401	2.93620	13
−1	22	−6	10.8955	1.66161	9
1	22	6	15.0789	3.49303	10
−2	22	−6	8.48009	0.80774	6
−2	22	−6	6.89365	1.17188	9
2	22	6	3.97938	3.21859	10
3	22	6	9.08646	2.71513	10
4	22	6	4.79770	2.05387	10
5	23	−6	21.1278	3.22212	10
−4	−23	6	1.92461	1.97761	13
4	−23	−6	2.87150	2.20992	13
4	23	−6	0.91396	2.20510	10
3	−23	−6	−1.7053	1.87490	13
−3	−23	6	0.04516	2.08372	13
3	23	−6	−1.4032	1.58724	11
−2	−23	6	13.0707	2.92517	13
2	−23	−6	20.1126	3.33220	13
2	23	−6	12.2054	1.82147	11
−2	23	6	17.3544	1.93259	11
−1	−23	6	19.6575	3.32095	13
1	−23	−6	28.2447	4.47817	13
−1	23	6	27.0382	3.16240	11
0	−23	−6	30.5029	4.68605	13
0	23	6	30.5297	4.03248	10
−1	−23	−6	1.69475	2.10592	13
−1	23	−6	1.87978	0.61011	6
1	23	6	1.12650	1.79032	10
−1	23	−6	0.51446	1.06734	9
−2	23	−6	11.9821	1.28443	6
−2	23	−6	17.0275	1.60256	9
−2	23	−6	16.3711	1.16500	6
2	23	6	16.5515	3.12777	10
3	23	6	0.72948	2.46744	10
−4	−24	6	3.10892	1.77142	13
4	−24	−6	4.59554	1.67081	13
4	24	−6	2.26500	1.70643	11
−3	−24	6	12.8120	2.50224	13
3	−24	−6	16.3453	3.00425	13
3	24	−6	9.48298	1.52600	11

2	-24	-6	5.44756	2.15955	13
-1	-24	6	20.1349	3.25515	13
1	-24	-6	18.8185	3.24507	13
-1	24	6	19.5877	2.58757	11
0	-24	-6	8.58974	2.41375	13
0	24	6	9.94510	2.44564	10
-1	-24	-6	5.67380	1.94823	13
1	24	6	5.00028	2.23703	10
-1	24	-6	3.42804	1.18717	9
2	24	6	29.2238	3.93136	10
-2	24	-6	28.9117	2.11905	9
-3	-25	6	7.63005	1.91726	13
3	-25	-6	5.40547	2.03921	13
2	-25	-6	28.1036	3.47074	13
-2	-25	6	27.7970	3.53288	13
-1	-25	6	2.87958	1.70569	13
1	-25	-6	-0.7091	1.69065	13
-1	25	6	0.18827	0.72745	11
-1	25	6	0.39297	0.72591	11
0	-25	-6	2.74921	2.15832	13
0	25	6	4.27842	1.17089	11
-1	-25	-6	6.91648	2.03585	13
1	25	6	6.46773	2.02017	10
2	-26	-6	5.55636	1.68450	13
-2	-26	6	1.70171	1.50363	13
1	-26	-6	10.6411	2.02274	13
-1	-26	6	13.1061	2.21127	13
0	-26	-6	2.25538	1.52420	13
0	26	6	2.15604	0.88308	11
10	0	-7	3.13088	2.68363	13
-9	0	7	49.9874	3.88686	11
9	0	-7	55.4944	6.73677	13
9	0	-7	38.8352	6.94082	13
-8	0	7	59.7908	6.07785	10
8	0	-7	79.3503	7.86687	13
-7	0	7	10.3001	3.18203	10
7	0	-7	5.53134	3.16686	13
-6	0	7	54.9883	6.54232	10
-6	0	7	48.1421	7.20811	1
-5	0	7	29.0398	5.49751	1
-4	0	7	96.4738	9.50320	5
-4	0	7	99.8283	9.39424	1
-3	0	7	77.6785	8.12847	1
-3	0	7	83.5024	8.73233	5
-2	0	7	132.315	11.4178	1
1	0	-7	69.0150	6.67683	5
0	0	-7	63.7976	5.90008	5
-1	0	-7	1.30423	1.03276	5
-2	0	-7	68.4611	6.30788	1
-2	0	-7	71.3325	5.70254	4

−3	0	−7	15.2298	2.16502	8
−3	0	−7	15.3161	2.53255	4
−4	0	−7	95.8203	6.92596	8
−5	0	−7	1.41840	1.57625	8
−6	0	−7	136.977	9.22483	8
7	0	7	0.55229	3.23352	13
−7	0	−7	3.09539	1.06866	8
8	0	7	2.87049	2.20073	13
10	−1	−7	30.4551	4.62033	13
10	1	−7	24.0942	4.52822	13
−9	−1	7	28.8690	3.81121	10
9	−1	−7	40.5140	6.34244	13
−9	1	7	29.8042	2.81809	11
9	1	−7	32.9325	5.04838	13
9	1	−7	27.1320	4.64888	13
8	−1	−7	149.516	11.6115	13
−8	−1	7	137.416	10.6327	10
−8	1	7	151.154	10.6640	10
8	1	−7	134.643	11.5677	13
7	−1	−7	118.308	10.7653	13
−7	−1	7	121.894	9.64447	10
7	1	−7	130.662	10.6153	13
−7	1	7	120.819	9.73546	10
−6	−1	7	72.7748	8.44326	1
−6	−1	7	76.8296	7.37387	10
−5	1	7	46.7136	6.83788	1
−4	−1	7	114.773	10.7015	5
−4	−1	7	119.768	10.4833	1
−4	1	7	110.437	10.3385	1
−4	1	7	109.918	10.3241	5
−3	−1	7	114.314	11.0671	1
−3	1	7	147.874	12.2848	5
−3	1	7	143.206	11.6689	1
−2	−1	7	172.770	13.2781	1
−2	1	7	160.735	13.0879	1
1	−1	−7	313.745	20.9749	5
1	1	−7	300.621	20.7121	5
0	−1	−7	35.4385	4.24683	5
0	1	−7	32.7772	3.80562	5
−1	−1	−7	78.6076	5.85327	5
−1	1	−7	56.1464	5.50595	4
−2	−1	−7	14.9310	3.15731	1
−2	1	−7	11.7460	2.42865	1
−2	1	−7	10.4098	2.12486	4
−3	−1	−7	4.06943	2.12960	4
−3	−1	−7	4.87371	2.17636	1
−3	−1	−7	5.90358	1.70833	8
−3	1	−7	4.68237	1.63431	4
−3	1	−7	4.70916	1.50218	8
−4	1	−7	13.9550	2.27461	8

−5	−1	−7	9.03857	1.85332	8
−5	1	−7	8.66485	1.80282	8
−5	1	−7	10.9104	2.99190	12
5	1	7	6.86726	4.83120	13
−6	−1	−7	39.5455	3.82462	8
−6	−1	−7	34.3762	2.99458	11
−6	1	−7	34.5042	3.34149	8
−7	−1	−7	7.17097	1.43488	8
7	1	7	6.70235	2.96971	13
8	1	7	−1.0117	2.23961	13
10	−2	−7	62.4841	6.21514	13
10	2	−7	69.2149	6.24708	13
9	2	−7	7.85903	3.74390	13
−9	2	7	5.51376	1.39747	11
−8	−2	7	49.9175	5.58911	10
8	−2	−7	44.6618	6.46765	13
8	2	−7	54.6139	7.45631	13
−8	2	7	49.4632	4.89460	10
7	−2	−7	255.876	19.7882	13
−7	−2	7	315.974	21.6426	1
−7	−2	7	308.670	19.7892	10
−7	2	7	261.030	19.2398	10
7	2	−7	313.785	19.9494	13
−6	−2	7	35.2767	7.12058	1
−6	−2	7	35.4438	4.77630	10
−6	2	7	41.5017	4.59355	10
−5	−2	7	229.727	17.6910	1
−4	−2	7	231.322	15.7528	1
4	−2	−7	180.046	14.3881	2
−4	2	7	175.620	14.4758	5
−3	−2	7	31.9848	5.32817	1
−3	2	7	36.6740	5.46470	5
−2	−2	7	6.96082	3.53756	1
1	−2	−7	208.519	14.6522	5
1	2	−7	190.459	13.9570	5
0	−2	−7	51.6784	5.47568	5
0	2	−7	40.5433	4.04680	5
−1	−2	−7	23.8273	3.35971	5
−1	2	−7	20.5741	2.69216	4
−2	−2	−7	40.2722	5.35528	1
−2	2	−7	42.9682	4.16648	1
−2	2	−7	44.9381	4.03733	4
−3	−2	−7	0.28198	2.13772	1
−3	−2	−7	0.89425	1.30004	8
−3	2	−7	1.53843	1.16565	4
−3	2	−7	1.27002	1.29434	8
−4	−2	−7	36.3453	3.82491	8
−4	−2	−7	48.9357	4.91435	10
−4	2	−7	42.9567	3.76709	8
−5	−2	−7	62.2711	5.23374	8

−5	−2	−7	62.2889	5.56330	10
5	2	7	57.7673	8.03842	13
−5	2	−7	69.1666	4.45372	11
−5	2	−7	72.7963	6.40997	12
−5	2	−7	69.2414	5.02363	8
−6	−2	−7	17.6395	2.59925	8
−6	2	−7	18.3884	2.28517	8
6	2	7	11.3167	4.52345	13
−7	−2	−7	78.9671	5.51834	8
7	2	7	78.8151	7.50794	13
8	2	7	2.25655	2.42687	13
10	−3	−7	29.6702	5.13839	13
10	3	−7	28.8595	4.51713	13
9	−3	−7	35.4297	6.26825	13
−9	−3	7	39.6439	4.71320	10
9	3	−7	49.6942	6.54348	13
−9	3	7	40.1689	3.21861	11
−8	−3	7	7.96177	3.10848	10
8	3	−7	18.2612	5.11525	13
7	−3	−7	85.6332	7.73038	13
−7	−3	7	62.0590	7.04671	10
−7	−3	7	66.8224	8.33924	1
7	3	−7	63.2495	7.32864	13
−7	3	7	81.9001	6.79237	10
−6	−3	7	139.635	12.0254	1
−6	−3	7	126.223	9.82037	10
−6	3	7	109.822	9.53538	10
5	−3	−7	73.3462	7.99317	2
−5	−3	7	73.4897	8.94953	1
4	−3	−7	89.0731	8.35005	2
−4	−3	7	76.5995	8.68217	1
−4	3	7	87.8578	7.71422	5
3	−3	−7	2.64543	1.97481	2
−3	−3	7	5.66220	2.99274	1
−3	3	7	3.41804	2.52430	5
2	−3	−7	8.43009	2.59533	2
−2	3	7	9.84244	3.46792	5
1	3	−7	10.5007	2.58116	5
0	−3	−7	112.242	10.1226	5
0	3	−7	136.985	9.05815	4
0	3	−7	131.143	8.40419	5
0	3	−7	131.842	8.55099	5
−1	−3	−7	25.8251	3.74904	5
−1	3	−7	20.6318	2.83388	4
−2	−3	−7	14.5224	2.36650	5
−2	−3	−7	15.8853	3.43371	1
−2	3	−7	10.8035	1.96673	4
−3	−3	−7	91.6195	6.41478	8
−3	−3	−7	94.7148	8.06325	1
−3	3	−7	77.1646	5.97576	4

−3	3	−7	80.5642	5.98448	8
−4	−3	−7	25.6280	4.03485	10
−4	−3	−7	20.5251	2.79582	8
−4	3	−7	16.0625	2.48667	8
−5	−3	−7	0.11770	1.96635	10
−5	−3	−7	0.65243	1.97878	8
−5	3	−7	−0.1124	2.60002	12
−5	3	−7	0.05001	1.16865	8
5	3	7	−0.8581	3.28438	13
−6	−3	−7	1.43939	1.61724	8
−6	3	−7	0.20965	1.07726	8
6	3	7	1.93503	3.30423	13
7	3	7	13.0351	3.44505	13
8	3	7	−0.0412	2.40633	13
10	−4	−7	1.99417	2.37712	13
10	4	−7	1.43907	2.44702	13
9	−4	−7	18.4863	3.81333	13
−9	−4	7	20.0648	3.38437	10
−9	4	7	17.9981	1.72537	11
−9	4	7	18.8068	3.06750	7
9	4	−7	21.1064	4.19874	13
−8	−4	7	49.8654	5.71343	10
8	−4	−7	49.6563	7.34098	13
−8	4	7	47.7705	4.19047	11
8	4	−7	53.2205	7.42166	13
8	4	−7	47.3986	6.85319	13
−7	−4	7	24.2715	4.35407	10
−7	−4	7	27.2198	6.15952	1
7	4	−7	20.6447	4.24694	13
−6	−4	7	169.093	12.8519	10
6	−4	−7	165.070	14.5496	2
−6	−4	7	179.949	14.7401	1
−6	4	7	157.815	12.0894	10
−5	−4	7	13.3374	4.98151	1
4	−4	−7	89.8039	8.57597	2
−4	4	7	91.9780	7.59560	5
3	−4	−7	353.153	23.3419	2
−3	−4	7	327.762	24.1456	1
−3	4	7	345.753	23.6165	5
2	−4	−7	17.5863	3.37458	2
−2	4	7	17.0532	4.28332	5
1	−4	−7	122.402	9.45821	2
1	4	−7	115.661	8.87945	5
0	−4	−7	42.0258	4.97071	5
0	4	−7	30.4164	3.34604	4
−1	−4	−7	103.959	8.81017	1
−1	−4	−7	92.4224	7.76475	5
−1	4	−7	76.2243	6.43373	4
−2	−4	−7	52.8198	6.47106	1
−2	−4	−7	54.5758	5.07991	5

−2	4	−7	42.2370	4.12037	3
−2	4	−7	45.0184	3.96233	4
−3	−4	−7	5.95833	1.93169	8
−3	−4	−7	3.03636	2.67512	1
−3	4	−7	3.66369	0.86378	4
−3	4	−7	4.23436	0.97921	4
−3	4	−7	4.50693	1.57045	3
−3	4	−7	3.72691	1.30652	8
−4	−4	−7	30.4885	3.19622	8
−4	−4	−7	30.3023	3.88735	10
−4	−4	−7	32.0012	4.40958	1
−4	4	−7	22.4055	2.32556	11
−4	4	−7	27.1303	3.94446	12
−4	4	−7	23.7902	2.66183	8
−5	−4	−7	82.3038	6.54756	10
−5	−4	−7	81.8410	6.25213	8
−5	4	−7	65.8333	6.37802	12
−5	4	−7	69.1415	5.28702	8
5	4	7	84.0827	8.16281	13
−6	−4	−7	9.27720	2.12204	8
−6	4	−7	10.8889	1.36466	9
−6	4	−7	9.60569	3.05681	12
6	4	7	6.06322	3.78168	13
−7	−4	−7	13.2190	2.16673	8
7	4	7	12.3465	3.44323	13
−7	4	−7	13.9603	1.60668	9
−7	4	−7	12.8975	1.17901	6
8	4	7	7.54054	2.23605	13
10	−5	−7	13.5509	2.68840	13
10	5	−7	10.6293	3.11365	13
−9	−5	7	31.5869	3.96746	10
9	−5	−7	29.4158	5.29373	13
−9	5	7	34.4065	3.63841	7
9	5	−7	28.0144	5.42166	13
8	−5	−7	76.6523	7.89411	13
−8	−5	7	84.8697	7.48063	10
−8	5	7	72.2467	5.60532	11
8	5	−7	85.6437	8.39364	13
−7	−5	7	155.352	12.0569	10
−7	−5	7	170.241	14.7670	1
7	−5	−7	162.965	11.7861	13
−7	5	7	162.864	11.3838	10
7	5	−7	145.049	12.1979	13
−6	−5	7	146.080	13.5213	1
6	−5	−7	169.449	13.8902	2
−6	−5	7	130.204	11.1920	10
−6	5	7	145.905	10.6011	10
−5	−5	7	185.931	14.9980	1
5	−5	−7	150.565	13.7356	2
−4	−5	7	156.955	13.3626	1

4	−5	−7	153.127	12.3854	2
−4	5	7	145.002	10.2252	5
3	−5	−7	3.43397	2.71365	2
−3	5	7	2.40156	2.41935	5
2	−5	−7	121.447	9.76800	2
−2	5	7	121.919	10.6957	5
1	−5	−7	70.9236	6.79807	2
1	5	−7	68.4923	5.61805	5
0	−5	−7	51.4249	5.59314	2
0	−5	−7	53.8028	5.84972	5
0	5	−7	40.0428	4.06577	4
−1	−5	−7	38.4228	5.38335	1
−1	−5	−7	38.3286	5.01724	5
−1	5	−7	35.3342	3.40883	4
−1	5	−7	50.7034	5.45110	3
−2	−5	−7	66.4803	7.04417	1
−2	−5	−7	59.4730	6.10033	5
−2	5	−7	61.4490	4.50432	4
−2	5	−7	59.0906	4.51567	4
−2	5	−7	63.3767	5.05625	3
−3	−5	−7	27.2964	3.17403	8
−3	−5	−7	30.5356	5.26709	1
−3	5	−7	30.0610	3.04386	3
−3	5	−7	25.1138	2.65958	8
−4	−5	−7	25.1554	3.06905	8
−4	−5	−7	25.5591	4.79570	1
−4	−5	−7	19.0755	3.81342	10
4	5	7	25.1440	5.48289	13
−4	5	−7	21.2593	2.33347	8
−4	5	−7	27.4714	4.11086	12
−4	5	−7	23.0008	1.58114	11
−4	5	−7	21.6162	2.01400	9
−5	−5	−7	3.83781	2.09784	10
−5	−5	−7	4.28647	1.88937	8
−5	5	−7	4.75398	2.35665	12
5	5	7	7.20723	3.61374	13
−5	5	−7	7.71731	1.23524	9
−6	−5	−7	78.5999	5.77749	8
−6	−5	−7	67.6746	5.54180	10
−6	5	−7	69.3429	4.51485	6
−6	5	−7	68.9703	6.49558	12
−6	5	−7	65.1050	4.82537	9
6	5	7	70.9724	7.93735	13
−7	−5	−7	4.56849	1.50738	8
−7	5	−7	2.48533	0.81790	9
7	5	7	5.87564	3.20323	13
−7	5	−7	2.98667	0.51223	6
10	−6	−7	3.13894	2.57523	13
10	−6	−7	3.21329	1.91275	13
9	−6	−7	4.92889	2.82439	13

−9	−6	7	2.84725	2.22328	10
9	6	−7	2.76216	3.10724	13
−9	6	7	5.22323	1.51100	7
8	−6	−7	77.5303	7.81282	13
−8	−6	7	70.8163	7.12601	10
8	6	−7	73.0820	8.19489	13
−8	6	7	75.2258	6.22804	7
−8	6	7	78.1922	5.24624	11
−7	−6	7	33.4882	5.10026	10
−7	−6	7	37.2634	7.03260	1
7	−6	−7	36.7417	5.66725	13
7	6	−7	35.5838	6.80729	13
−7	6	7	32.0301	3.73861	10
7	6	−7	34.0515	6.31437	13
−6	−6	7	120.425	10.3625	10
6	−6	−7	150.624	13.1478	2
−6	−6	7	132.251	12.9601	1
−6	6	7	128.048	9.72872	10
−5	−6	7	403.704	29.4724	1
5	−6	−7	408.024	28.6500	2
4	−6	−7	220.549	16.4505	2
−4	−6	7	209.415	17.5733	1
3	−6	−7	142.894	11.5899	2
−3	6	7	131.811	10.4613	5
2	−6	−7	38.8485	5.49655	2
−2	6	7	42.9841	5.91965	5
1	−6	−7	54.4258	5.90767	2
−1	6	7	56.0394	6.73702	5
0	−6	−7	168.515	12.9947	2
0	6	−7	174.809	11.5169	4
−1	−6	−7	45.6969	5.62228	2
−1	−6	−7	42.7467	5.59864	5
−1	−6	−7	46.6750	6.37436	1
−1	6	−7	45.8143	3.98640	3
−1	6	−7	45.1254	3.70650	4
−2	−6	−7	285.976	21.1233	2
−2	−6	−7	302.326	20.8328	5
−2	−6	−7	293.608	21.3919	1
−2	6	−7	304.455	18.5196	4
−2	6	−7	313.703	19.2485	3
−2	6	−7	283.161	18.4516	4
−3	−6	−7	138.724	9.26514	8
−3	−6	−7	145.546	11.5218	1
−3	6	−7	122.897	8.27092	9
−3	6	−7	126.826	9.14083	3
−3	6	−7	124.130	8.51329	8
−4	−6	−7	77.9470	8.51774	1
−4	−6	−7	71.6453	6.44772	8
−4	−6	−7	82.8663	7.57335	10
−4	6	−7	88.1565	5.68189	9

−4	6	−7	90.4223	5.81258	8
4	6	7	68.9090	8.26680	13
−4	6	−7	98.4224	7.36515	12
−4	6	−7	94.3632	5.52003	6
−5	−6	−7	17.2826	3.36683	10
−5	−6	−7	17.5739	2.79811	8
−5	6	−7	21.2513	2.15362	9
−5	6	−7	26.5622	3.79477	12
−5	6	−7	22.7440	1.71616	6
5	6	7	19.8678	4.91142	13
−6	−6	−7	55.1982	4.92338	8
−6	−6	−7	54.1290	4.83057	10
−6	6	−7	54.1295	3.85861	9
6	6	7	51.8766	7.10331	13
−6	6	−7	54.2059	3.58356	6
−7	−6	−7	12.7161	2.08509	8
−7	6	−7	19.4678	1.69149	9
7	6	7	15.1857	3.65674	13
−7	6	−7	17.5122	1.27018	6
−9	−7	7	6.26362	2.48330	10
9	−7	−7	5.11296	2.72902	13
−9	7	7	4.91962	1.44572	7
9	7	−7	5.52141	3.12440	13
8	−7	−7	48.0048	7.11075	13
−8	7	7	47.1134	4.28707	7
−8	7	7	49.8537	3.54123	11
7	−7	−7	2.09657	2.91738	13
−7	−7	7	3.39351	3.84219	1
−7	−7	7	0.68196	2.46895	10
−7	7	7	1.87725	1.13194	11
7	7	−7	3.73240	3.20309	13
−6	−7	7	88.2491	8.06516	10
−6	−7	7	101.255	10.8272	1
6	7	−7	72.8628	8.43487	13
6	7	−7	79.6042	8.19749	13
−5	−7	7	148.517	14.6270	1
5	−7	−7	183.988	14.8439	2
4	−7	−7	172.380	13.1344	2
−4	−7	7	130.112	13.5760	1
3	−7	−7	365.911	24.7445	2
−3	7	7	345.468	23.2359	5
2	−7	−7	221.047	16.2797	2
−2	7	7	224.725	16.3313	5
1	−7	−7	430.263	28.7782	2
−1	7	7	430.406	29.4046	5
0	−7	−7	66.4758	7.01430	2
0	7	−7	63.0867	5.02211	4
−1	−7	−7	18.2780	4.42493	1
−1	−7	−7	16.8677	3.74600	2
−1	−7	−7	18.6772	3.77449	5

−1	7	−7	14.0252	2.05215	3
−1	7	−7	12.6657	1.81897	4
−2	−7	−7	350.475	23.8149	5
−2	−7	−7	324.877	22.7972	2
−2	−7	−7	337.487	23.2120	1
−2	7	−7	290.642	19.5320	4
−2	7	−7	320.382	19.5824	4
−2	7	−7	306.475	20.4852	3
−3	−7	−7	310.911	21.8318	1
−3	−7	−7	299.501	19.0761	8
−3	7	−7	288.428	18.2069	9
−3	7	−7	293.427	18.0933	6
−3	7	−7	293.581	18.3894	8
−3	7	−7	289.080	19.0477	3
−4	−7	−7	14.5491	4.46478	1
−4	−7	−7	13.6570	3.38974	10
−4	−7	−7	10.2136	2.33608	8
−4	7	−7	17.9989	1.48493	6
−4	7	−7	14.3673	1.73296	9
4	7	7	9.56315	4.49729	13
−4	7	−7	19.0971	3.18800	12
−5	−7	−7	74.7939	6.71379	8
−5	−7	−7	79.3534	6.95496	10
5	7	7	77.9472	8.51193	13
−5	7	−7	86.7666	6.98939	12
−5	7	−7	85.1349	5.22345	6
−5	7	−7	82.5904	5.40447	9
−6	7	−7	21.0561	1.91578	9
−7	−7	−7	5.27907	1.63913	8
−7	7	−7	4.42723	0.49276	6
7	7	7	4.50530	2.99985	13
−7	7	−7	6.72926	0.91217	9
−7	7	−7	5.47960	0.53041	6
9	−8	−7	9.98980	2.77137	13
−9	−8	7	7.09974	2.00335	10
9	8	−7	3.19798	3.41450	13
−9	8	7	7.43873	1.63355	7
−8	8	7	35.3582	2.34029	11
−8	8	7	29.1217	3.82593	7
−7	−8	7	50.3540	7.91416	1
7	−8	−7	33.8279	4.64702	13
7	8	−7	51.2848	7.36185	13
−7	8	7	30.3380	2.89029	11
−7	8	7	32.6176	3.91009	7
−6	−8	7	384.432	25.9083	10
6	−8	−7	377.216	25.1019	13
−6	−8	7	419.153	29.2017	1
−6	8	7	406.851	24.9887	10
6	8	−7	355.111	26.5671	13
5	−8	−7	0.45602	3.16847	2

−5	−8	7	5.30322	4.04225	1
4	−8	−7	23.0518	5.27093	2
3	−8	−7	85.4855	8.97719	2
2	−8	−7	97.5885	8.98867	2
−2	8	7	97.8479	8.60695	5
1	−8	−7	716.863	46.2846	2
0	−8	−7	37.9019	5.77395	2
0	8	−7	40.6539	3.58425	4
−1	−8	−7	619.991	38.6471	2
−1	−8	−7	628.351	39.1719	1
−1	8	−7	571.856	35.8718	4
−1	8	−7	543.104	35.8610	4
−1	8	−7	560.327	35.8756	4
−2	−8	−7	55.4929	7.25921	1
−2	−8	−7	54.5428	6.66665	2
−3	−8	−7	9.09302	2.22419	8
−3	−8	−7	13.0174	3.95184	1
−3	−8	−7	11.3307	3.71184	2
−3	−8	−7	12.6059	3.55081	10
−4	−8	−7	120.892	8.81437	8
−4	−8	−7	127.817	9.55895	10
−4	−8	−7	123.498	11.3158	1
−4	8	−7	118.447	7.63431	6
4	8	7	122.054	11.0814	13
−4	8	−7	118.766	7.78445	9
−4	8	−7	122.201	9.26874	12
−5	−8	−7	5.96104	2.59793	10
−5	−8	−7	4.37919	2.17011	8
−5	8	−7	10.4941	1.34148	9
−5	8	−7	9.41164	0.94131	6
5	8	7	8.28082	3.88012	13
−5	8	−7	10.7323	2.82766	12
−6	−8	−7	27.1468	3.73930	10
−6	−8	−7	30.3648	3.85734	8
6	8	7	29.4374	5.00574	13
−6	8	−7	34.3865	2.16566	6
−6	8	−7	32.8508	4.32216	12
−6	8	−7	33.2838	2.46180	9
−7	−8	−7	1.24080	1.45655	8
−7	8	−7	2.60837	0.52310	9
−7	8	−7	1.74422	0.58176	9
7	8	7	0.72641	2.72150	13
−7	8	−7	0.34160	1.77091	12
9	−9	−7	8.36799	2.88332	13
−9	−9	7	5.43595	2.38435	10
−9	9	7	9.06363	1.91592	7
8	−9	−7	4.21177	2.73189	13
−8	−9	7	3.72945	2.69251	10
−8	9	7	5.46764	1.98284	7
7	−9	−7	10.5506	3.32768	13

−7	−9	7	12.5278	5.03642	1
−7	−9	7	8.17391	3.30291	10
−7	9	7	14.2792	2.49316	7
−7	9	7	11.8829	1.43658	11
−6	−9	7	177.858	16.2961	1
6	−9	−7	162.583	12.3172	13
−6	−9	7	162.162	13.0933	10
−6	9	7	177.154	11.8233	7
−6	9	7	191.867	11.5526	11
5	−9	−7	316.003	23.7821	2
−5	−9	7	333.950	25.0002	1
5	9	−7	324.415	22.0835	10
−5	9	7	296.364	20.7080	10
4	−9	−7	6.27467	3.51243	2
3	−9	−7	17.2193	4.38198	2
2	−9	−7	126.918	11.0702	2
−2	9	7	128.505	10.0024	5
1	−9	−7	25.3631	5.01542	2
−1	9	7	21.2532	4.76963	5
0	−9	−7	1.65132	2.52436	2
0	9	−7	2.46827	1.21167	4
0	9	−7	1.74307	1.12664	4
−1	−9	−7	33.5990	6.40370	1
−1	−9	−7	33.8729	5.13673	2
−1	9	−7	21.0606	2.24244	4
−1	9	−7	21.5022	2.19408	4
−2	−9	−7	30.2835	5.74571	2
−2	−9	−7	27.3304	6.13640	1
−2	9	−7	42.1353	2.55948	6
−3	−9	−7	322.035	24.1329	1
−3	−9	−7	324.454	21.8286	10
−3	9	−7	319.634	20.1870	9
−3	9	−7	347.183	21.6127	12
−3	9	−7	328.174	20.0635	6
−4	−9	−7	64.2216	6.50960	10
−4	−9	−7	69.4824	5.88652	8
−4	−9	−7	70.7834	8.43505	1
4	9	7	70.3906	7.65866	13
−4	9	−7	66.8889	4.56741	9
−4	9	−7	69.6919	4.43802	6
−4	9	−7	70.5469	6.31977	12
−5	9	−7	43.5197	5.28258	12
−5	9	−7	48.2348	3.25486	9
−5	9	−7	43.6879	3.01379	6
−6	−9	−7	13.6152	2.50366	8
−6	−9	−7	11.4609	2.50518	10
−6	9	−7	10.4958	2.69728	12
−6	9	−7	12.8393	1.46110	9
6	9	7	12.7697	3.83883	13
−6	9	−7	12.7362	0.97557	6

−6	9	−7	12.9892	1.03547	6
−7	−9	−7	6.86889	1.74133	8
−7	9	−7	6.69271	1.96170	12
7	9	7	8.98981	2.92637	13
9	−10	−7	17.3868	3.04433	13
−9	−10	7	14.2230	2.83888	10
−9	10	7	15.8869	2.56269	7
−8	−10	7	15.2837	3.47144	10
8	−10	−7	15.2478	3.09412	13
−8	10	7	13.7115	2.47962	7
−7	−10	7	3.11348	4.06956	1
−7	−10	7	0.13910	2.75189	10
−7	10	7	0.61958	1.75702	7
−7	10	7	3.25074	1.13144	11
−6	−10	7	14.3253	6.23745	1
6	−10	−7	2.88438	3.74249	13
−6	10	7	2.59725	1.18129	11
−6	10	7	3.85260	2.15298	7
5	−10	−7	77.0973	9.43280	2
−5	−10	7	83.1291	11.0275	1
5	10	−7	77.6695	7.56191	10
−5	10	7	64.6491	6.14760	10
4	−10	−7	19.0119	4.97700	2
3	−10	−7	−0.1081	2.89137	2
2	−10	−7	48.2631	7.19880	2
1	−10	−7	20.7855	4.68370	2
1	10	−7	31.8507	3.16296	4
−1	10	7	29.7600	5.27558	5
0	−10	−7	21.1142	5.06348	2
0	10	−7	33.3753	3.12717	4
−1	−10	−7	133.513	12.1652	2
−1	−10	−7	137.354	13.1069	1
−1	10	−7	152.578	9.15898	4
−1	10	−7	147.002	9.14744	4
−2	−10	−7	36.9783	5.85322	2
−2	−10	−7	36.9702	6.45228	1
−2	10	−7	28.4911	2.44515	9
−2	10	−7	26.7376	2.13100	6
−3	−10	−7	292.137	19.1028	10
−3	−10	−7	290.615	21.5032	2
−3	−10	−7	296.107	21.5763	1
−3	10	−7	272.984	17.0949	6
3	10	7	280.108	19.4811	13
−3	10	−7	260.728	18.3754	12
−3	10	−7	268.620	17.2205	9
−4	−10	−7	2.49151	2.60841	10
−4	−10	−7	1.61479	4.10769	1
−4	10	−7	2.33193	2.38186	12
−4	10	−7	3.03989	0.86895	9
4	10	7	2.60381	3.84346	13

−4	10	−7	3.11071	0.63508	6
−5	−10	−7	2.40132	3.33066	1
−5	−10	−7	2.81080	2.95868	10
−5	−10	−7	2.99419	2.31599	8
−5	10	−7	5.54285	0.92851	9
−5	10	−7	9.15894	3.05670	12
5	10	7	7.72819	4.61415	13
−5	10	−7	2.91711	0.60581	6
−5	10	−7	3.79391	0.56937	6
−6	−10	−7	8.88941	1.97626	8
−6	−10	−7	7.50699	2.05107	10
6	10	7	10.0882	3.82298	13
−6	10	−7	10.8848	1.07588	9
−6	10	−7	6.16991	0.92088	9
7	10	7	−0.5990	2.27582	13
−7	10	−7	3.04962	1.75267	12
−9	−11	7	1.83199	2.14181	10
−8	−11	7	10.0509	2.65095	10
−8	11	7	3.73578	1.51586	7
−7	−11	7	11.7851	3.64285	10
−7	−11	7	11.8737	5.06880	1
−7	11	7	10.2314	1.23187	11
−7	11	7	9.31122	1.31276	11
−7	11	7	10.5921	2.29149	7
−6	11	7	90.0545	6.19124	11
5	−11	−7	6.00160	2.70588	13
−5	−11	7	11.2301	3.34413	13
−5	−11	7	13.4778	5.76717	1
5	11	−7	9.41910	3.70787	10
4	−11	−7	62.3410	9.25513	2
4	11	−7	74.7310	7.96091	10
3	−11	−7	1.05334	3.25879	2
2	−11	−7	10.6308	3.99197	2
1	−11	−7	121.134	10.8753	2
1	11	−7	104.268	7.90469	4
0	−11	−7	229.951	17.0049	2
0	11	−7	205.147	13.8987	4
−1	−11	−7	29.5469	5.82807	2
−1	−11	−7	30.4858	6.32367	1
−2	−11	−7	79.8320	10.2030	1
−2	−11	−7	77.9796	9.58301	2
−2	11	−7	113.154	6.55075	9
−2	11	−7	111.773	6.33253	6
−3	−11	−7	242.018	16.0902	10
−3	−11	−7	235.780	18.5700	1
−3	11	−7	218.776	15.3413	14
3	11	7	242.388	16.5352	13
−3	11	−7	203.358	13.9888	9
−3	11	−7	221.774	13.8911	6
−3	11	−7	227.707	15.3467	12

−4	−11	−7	1.38504	3.53940	1
−4	−11	−7	1.13295	2.56499	10
−4	11	−7	1.28511	0.71650	9
−4	11	−7	1.29195	2.28643	14
−4	11	−7	1.35505	0.58339	6
−4	11	−7	−1.2996	2.38217	12
4	11	7	−0.1967	2.96125	13
−5	−11	−7	168.409	11.4576	8
−5	−11	−7	152.956	11.9127	10
−5	11	−7	158.768	9.94984	6
−5	11	−7	158.214	10.0904	9
−5	11	−7	165.710	9.97488	6
5	11	7	174.810	13.1583	13
−5	11	−7	166.597	11.8373	12
−6	−11	−7	−0.0305	1.34615	8
−6	−11	−7	0.48483	2.07047	10
−6	11	−7	1.01642	1.63397	12
6	11	7	5.64974	3.12840	13
−7	11	−7	22.3885	2.89516	12
7	11	7	17.3043	3.18275	13
−9	−12	7	2.41059	1.53143	10
−8	−12	7	43.7373	5.32593	10
−8	12	7	42.7423	4.07811	7
−7	−12	7	5.73364	3.06509	10
−7	12	7	5.74754	1.94013	7
−6	12	7	169.443	10.7686	11
5	−12	−7	34.6677	5.80934	13
−5	12	7	32.6216	3.48048	11
4	−12	−7	18.3280	5.08848	2
−4	12	7	14.7479	3.17670	10
4	12	−7	15.6996	3.47866	10
3	−12	−7	10.1105	4.21590	2
2	−12	−7	8.51042	3.92590	2
1	−12	−7	1.50843	3.45096	2
1	12	−7	2.15183	1.44913	4
0	−12	−7	140.986	12.6055	2
−1	−12	−7	174.944	14.6508	2
−1	−12	−7	169.002	15.0941	1
−2	−12	−7	42.8777	7.40061	1
−2	−12	−7	43.7196	6.84716	2
−2	−12	−7	40.9779	5.53661	10
−2	12	−7	43.8979	3.28769	9
−2	12	−7	53.6202	4.99336	12
−2	12	−7	41.9279	2.97310	6
−3	−12	−7	−0.5647	2.55541	10
−3	−12	−7	3.54420	3.85720	1
−3	12	−7	1.22462	0.77092	9
−3	12	−7	1.99061	0.55691	6
3	12	7	0.87563	3.46163	13
−3	12	−7	0.06576	2.27963	14

−3	12	−7	−0.2939	2.44152	12
−4	−12	−7	5.38658	3.89898	1
−4	−12	−7	3.68629	2.14409	10
−4	12	−7	10.7126	1.32415	9
−4	12	−7	7.23043	0.78231	6
−4	12	−7	7.81260	0.78474	6
4	12	7	2.80166	3.81010	13
−4	12	−7	8.15419	2.78287	12
−4	12	−7	6.85582	3.08896	14
−5	−12	−7	122.744	9.17885	10
−5	12	−7	108.703	6.95977	9
5	12	7	114.485	10.0960	13
−5	12	−7	107.673	6.74805	6
−5	12	−7	105.646	8.84822	12
−5	12	−7	107.576	6.74945	6
−5	12	−7	113.348	6.92103	9
−6	−12	−7	12.9463	2.44142	8
−6	−12	−7	9.21210	2.08601	10
−6	12	−7	5.92549	2.48839	12
6	12	7	13.2366	3.56685	13
−8	−13	7	30.8365	4.07173	10
−8	13	7	37.0797	3.54669	7
−7	−13	7	3.23797	3.00310	10
−6	13	7	18.6996	2.03450	11
−6	13	7	15.9166	2.18094	11
6	13	−7	19.8938	4.44158	10
5	−13	−7	120.270	9.90557	13
−5	−13	7	94.5186	8.79333	13
−5	13	7	117.280	7.50005	11
5	13	−7	103.006	9.54528	10
4	−13	−7	48.0793	7.73517	2
−4	−13	7	32.1947	5.05515	13
−4	13	7	37.2201	4.48156	10
4	13	−7	37.0774	5.75974	10
2	−13	−7	14.9016	4.75519	2
1	−13	−7	10.5440	4.51497	2
0	−13	−7	31.2428	6.42495	2
−1	−13	−7	296.221	20.6320	2
−1	13	−7	236.078	16.0059	11
−2	−13	−7	120.728	12.0104	1
−2	13	−7	106.442	8.69430	12
−2	13	−7	110.763	7.37972	9
−2	13	−7	121.285	7.13064	6
−2	13	−7	107.201	9.26823	14
−3	13	−7	37.3973	5.61322	14
−3	13	−7	40.2935	5.56008	12
−3	13	−7	36.4899	2.78256	9
−3	13	−7	34.9194	2.48739	6
−4	−13	−7	66.7355	6.53858	10
−4	−13	−7	68.3950	8.81409	1

−4	13	−7	61.0676	4.00149	9
−4	13	−7	55.9032	6.03616	12
4	13	7	59.4464	6.47336	13
−4	13	−7	52.1640	6.77702	14
−4	13	−7	55.7000	3.71755	6
−4	13	−7	57.2723	3.74407	6
−5	−13	−7	14.2085	3.24167	10
5	13	7	15.9929	3.44171	13
−5	13	−7	10.4700	3.09899	12
−5	13	−7	11.0445	1.00534	9
−6	−13	−7	0.88729	2.08553	10
−6	13	−7	2.73008	2.19553	12
6	13	7	1.05253	2.92284	13
−8	−14	7	19.9222	3.09708	10
−7	−14	7	0.89132	1.94374	10
7	14	−7	2.01302	2.53874	10
−6	−14	7	14.3768	5.63334	1
6	14	−7	15.1842	4.61998	10
−6	14	7	17.9215	1.68734	11
−6	14	7	17.7104	1.65907	11
−5	−14	7	57.3217	5.66654	13
5	−14	−7	43.2626	6.06330	13
−5	14	7	46.0054	3.89698	11
5	14	−7	59.7695	6.72370	10
−4	−14	7	22.5822	4.39567	13
4	−14	−7	29.6803	4.03084	13
4	14	−7	26.2475	5.04312	10
−4	14	7	29.0623	3.75516	10
3	−14	−7	12.0857	4.97505	2
−3	−14	7	16.6108	3.58194	13
3	14	−7	15.8910	3.36003	10
−3	14	7	13.8266	3.00672	10
2	−14	−7	13.3715	4.98166	2
2	14	−7	11.9136	2.98988	10
−2	14	7	12.7287	3.39405	10
1	−14	−7	0.69232	4.55558	2
−1	14	7	1.13699	2.71689	10
0	−14	−7	18.2728	5.00907	2
0	14	−7	10.3015	2.04124	11
−1	−14	−7	21.3308	5.30729	2
1	14	7	25.9733	5.03569	10
−1	14	−7	21.5981	1.72127	11
−1	14	−7	12.3830	3.08653	14
−2	−14	−7	1.14309	3.57263	1
−2	14	−7	−1.5352	2.26253	14
2	14	7	1.88027	3.71307	10
−2	14	−7	−0.0605	0.52198	6
−2	14	−7	0.77431	1.05257	9
−2	14	−7	0.40629	2.19429	12
−3	−14	−7	16.1523	6.03119	1

-3	-14	-7	11.3549	3.48662	10
-3	14	-7	14.0277	3.04545	14
-3	14	-7	15.7169	3.10732	12
-3	14	-7	15.1322	1.28138	6
-3	14	-7	17.5177	1.86190	9
-4	-14	-7	38.0686	5.60075	10
-4	-14	-7	43.4808	8.40852	1
-4	14	-7	35.7509	2.44380	6
-4	14	-7	38.9353	2.80440	9
-4	14	-7	38.0693	2.46205	6
-4	14	-7	39.7909	2.80590	9
-5	-14	-7	5.53733	2.89607	10
-6	-14	-7	8.06714	2.28246	10
-6	14	-7	11.2655	2.50810	12
-8	-15	7	3.51436	1.80896	10
7	15	-7	40.9422	5.23294	10
6	15	-7	63.9481	7.62378	10
-5	-15	7	1.19746	2.77182	13
5	-15	-7	3.48587	2.79592	13
5	15	-7	0.67613	3.09059	10
-5	15	7	2.84106	1.10313	11
4	-15	-7	-2.0156	2.01410	13
-4	-15	7	-0.5005	2.36112	13
4	15	-7	4.61201	3.72895	10
-4	15	7	-0.9874	2.02850	10
-3	-15	7	20.1511	3.67323	13
3	-15	-7	34.8790	4.89327	13
3	15	-7	24.6462	4.58095	10
2	-15	-7	-0.7266	2.18627	13
-2	-15	7	2.10865	2.40633	13
2	-15	-7	2.44213	5.16187	2
2	15	-7	0.10746	2.26391	10
-2	15	7	4.23470	2.52327	10
1	-15	-7	10.8328	3.06446	13
1	-15	-7	10.1285	5.58569	2
1	15	-7	9.40180	2.67777	10
-1	15	7	12.9938	3.46957	10
0	-15	-7	13.1049	4.80370	2
0	15	-7	6.82301	2.88205	14
-1	-15	-7	34.9445	4.94385	13
1	15	7	36.7880	5.78532	10
-1	15	-7	31.8612	4.98280	14
-2	15	-7	66.4310	6.26262	14
-2	15	-7	63.4636	6.05130	12
-2	15	-7	64.1749	4.60954	9
-2	15	-7	64.7551	4.17365	6
-3	-15	-7	23.4703	4.39353	10
-3	-15	-7	24.4747	6.31802	1
-3	15	-7	27.4904	2.34306	9
-3	15	-7	28.4615	1.91620	6

−3	15	−7	24.3160	3.77483	12
−3	15	−7	28.8231	3.92860	14
−4	−15	−7	32.9747	5.39782	10
−4	15	−7	30.5176	2.30882	9
−4	15	−7	33.7716	2.31555	9
−4	15	−7	34.4225	5.24899	14
−4	15	−7	29.6742	4.89089	12
−5	−15	−7	15.8307	3.33436	10
−5	15	−7	19.1378	3.21329	12
−6	−15	−7	2.75936	1.97085	10
−6	15	−7	1.18152	1.44135	12
−8	−16	7	5.65770	1.88851	10
7	16	−7	43.3578	5.16264	10
6	16	−7	32.1099	6.05397	10
5	−16	−7	7.60244	2.95264	13
−5	16	7	6.19924	1.09591	11
5	16	−7	15.2744	4.40194	10
−5	16	7	7.35554	1.28782	11
−4	−16	7	9.25066	2.81187	13
4	−16	−7	6.64832	2.52251	13
4	16	−7	12.8326	3.64474	10
−4	16	7	8.80193	1.86929	11
3	−16	−7	29.2733	4.21862	13
−3	−16	7	39.3658	5.52121	13
−3	16	7	25.6385	4.45017	10
2	−16	−7	4.77829	2.36150	13
−2	−16	7	8.43083	2.64005	13
−2	16	7	5.17144	2.71072	10
1	−16	−7	1.76221	2.88969	13
−1	16	7	8.01743	3.34529	10
1	16	−7	2.18241	2.54753	14
1	16	−7	5.13078	2.10069	11
0	−16	−7	52.8511	5.46401	13
0	16	−7	58.4169	4.28251	11
0	16	7	54.5132	6.86631	10
0	16	−7	59.7672	5.89752	14
−1	−16	−7	16.7479	3.39036	13
1	16	7	19.0048	4.63857	10
−1	16	−7	18.7191	3.80902	14
−2	−16	−7	5.55783	4.90378	1
−2	16	−7	5.18999	2.48560	12
−2	16	−7	4.64343	0.72872	6
2	16	7	1.71024	3.67311	10
−2	16	−7	5.78192	2.72278	14
−2	16	−7	4.18338	1.20672	9
−3	−16	−7	34.3876	6.58945	10
−3	−16	−7	40.5886	7.76189	1
3	16	7	42.9351	7.75534	10
−3	16	−7	37.5859	2.89898	9
−3	16	−7	40.3189	2.60418	6

−3	16	−7	36.1058	5.73555	14
−3	16	−7	38.2466	2.55147	6
−4	−16	−7	0.45186	2.90015	10
−4	16	−7	2.08026	0.50698	9
−4	16	−7	−1.3247	3.25376	14
−4	16	−7	1.02906	0.46937	9
−4	16	−7	2.41163	2.68388	12
−5	−16	−7	87.7086	6.99069	10
−5	16	−7	83.2965	7.09032	12
−6	−16	−7	3.63726	1.90571	10
−6	16	−7	4.85577	1.94864	12
7	17	−7	4.16885	3.28551	10
6	17	−7	6.31221	3.82369	10
−5	−17	7	16.0224	3.27507	13
−5	17	7	19.0853	1.61482	11
5	17	−7	17.5625	4.37163	10
−5	17	7	20.5654	1.64358	11
−4	−17	7	7.25328	2.81217	13
−4	17	7	8.26924	1.50554	11
4	17	−7	14.5848	3.81186	10
3	−17	−7	24.8165	4.02115	13
−3	−17	7	20.5331	3.52073	13
3	17	−7	23.6592	4.03480	10
−3	17	7	28.3035	4.16834	10
2	−17	−7	16.4947	3.29834	13
−2	−17	7	15.0994	3.16223	13
−2	17	7	13.2486	3.17483	10
2	17	−7	13.9286	3.44359	10
−1	−17	7	18.1016	3.53042	13
1	−17	−7	20.5134	3.24845	13
1	17	−7	19.6941	3.35193	14
−1	17	7	24.6231	4.30355	10
1	17	−7	21.5783	3.16595	11
0	17	−7	10.1450	1.51039	11
0	17	−7	9.23241	3.48269	14
0	17	−7	11.9391	1.60730	11
−1	−17	−7	240.285	16.3363	13
−1	17	−7	235.767	16.5386	14
1	17	7	258.913	17.9419	10
−2	17	−7	34.5910	2.30543	6
−2	17	−7	32.4857	2.69110	9
−2	17	−7	35.3075	5.22680	14
2	17	7	29.1042	5.39006	10
−3	17	−7	3.63984	0.54460	6
−3	17	−7	4.97752	2.89106	14
−3	17	−7	2.95185	0.86442	9
−3	17	−7	4.37905	3.15321	12
3	17	7	0.63661	2.74026	10
−3	17	−7	2.79808	0.51272	6
−4	−17	−7	16.7055	3.90182	10

−4	17	−7	16.2651	2.80693	12
−4	17	−7	15.4596	3.44002	14
−5	−17	−7	41.0154	4.77431	10
−5	17	−7	44.1927	4.66370	12
7	18	−7	22.3227	3.58300	10
6	18	−7	27.0689	4.22331	10
−5	−18	7	5.31273	2.69465	13
5	18	−7	5.10355	3.60875	10
−4	−18	7	68.6411	6.82895	13
4	−18	−7	50.6361	6.22152	13
4	18	−7	76.7066	7.22609	10
−4	18	7	63.7551	4.72717	11
3	−18	−7	14.0203	2.88580	13
−3	−18	7	15.7442	3.64850	13
3	18	−7	12.9238	3.20248	10
−3	18	7	13.2141	2.55428	10
−2	−18	7	45.3772	6.15444	13
2	−18	−7	50.1102	5.86397	13
−2	18	7	47.5736	5.35539	10
2	18	−7	48.6595	5.47374	10
1	−18	−7	21.7679	3.33614	13
−1	−18	7	21.7020	3.72640	13
−1	18	7	25.8833	4.17054	10
1	18	−7	17.0518	3.37723	14
1	18	−7	20.1016	2.58605	11
0	−18	−7	37.3989	5.03086	13
0	18	7	42.1869	6.09016	10
0	18	−7	42.5510	3.09760	11
0	18	−7	41.1780	5.26955	14
0	18	−7	44.4811	3.07985	11
−1	−18	−7	26.8575	4.39697	13
−1	18	−7	32.5389	3.94036	9
1	18	7	27.4708	4.69971	10
−1	18	−7	29.3161	4.03867	14
−2	18	−7	55.2214	3.71530	6
−2	18	−7	58.9199	4.23441	9
2	18	7	63.1880	7.59868	10
−2	18	−7	51.1008	6.57917	14
−3	18	−7	1.43647	0.41363	6
−3	18	−7	1.16804	0.39683	6
−3	18	−7	−0.5116	2.69459	14
3	18	7	2.28411	3.64599	10
−3	18	−7	1.18668	2.41223	12
−3	18	−7	2.12386	0.85941	9
−4	−18	−7	3.73203	2.13973	10
−4	18	−7	3.54692	2.44719	12
−5	−18	−7	18.4212	3.26635	10
−5	18	−7	15.1019	2.61825	12
7	19	−7	21.9729	3.34610	10
6	19	−7	20.4401	3.73065	10

5	−19	−7	5.66229	3.16238	13
−5	−19	7	2.98626	3.18011	13
5	19	−7	7.36763	3.42714	10
−4	−19	7	38.1492	5.36493	13
4	−19	−7	42.8538	5.25766	13
−4	19	7	44.3902	3.19507	11
−4	19	7	42.7856	3.26798	11
−3	−19	7	15.7669	3.91928	13
3	−19	−7	11.0228	2.83777	13
−3	19	7	11.2661	1.93147	11
3	19	−7	14.0298	3.29019	10
2	−19	−7	13.3403	3.28394	13
−2	−19	7	8.33647	2.75704	13
2	19	−7	11.1907	2.95025	10
−2	19	7	10.8854	2.77768	10
−1	−19	7	6.12332	2.88117	13
1	−19	−7	1.82812	2.38577	13
−1	19	7	2.73710	2.75077	10
1	19	−7	3.74395	2.59437	14
1	19	−7	2.12983	1.60587	11
0	−19	−7	14.8174	3.05195	13
0	19	−7	14.0136	3.26028	14
0	19	7	12.4037	3.73516	10
−1	−19	−7	10.8707	2.78668	13
−1	19	−7	7.17166	1.49745	9
1	19	7	10.2277	3.98848	10
−1	19	−7	7.88748	3.20017	14
−2	19	−7	3.68643	1.24056	9
2	19	7	5.19698	3.86438	10
−2	19	−7	14.5789	3.46472	14
−2	19	−7	3.85943	0.65908	6
−3	19	−7	28.7458	2.25746	9
3	19	7	25.6669	5.20718	10
−3	19	−7	28.4564	1.85064	6
−3	19	−7	31.6588	2.25052	9
−4	−19	−7	26.5907	3.61872	10
−4	19	−7	22.9256	3.73470	12
4	19	7	29.5922	4.31174	10
6	20	−7	20.1351	3.38405	10
−5	−20	7	4.59692	2.21503	13
5	−20	−7	1.68834	2.23242	13
5	20	−7	4.03549	2.85950	10
4	−20	−7	13.2067	2.96829	13
−4	−20	7	5.81990	2.72781	13
4	20	−7	6.20428	2.86115	10
−3	−20	7	74.7183	8.30722	13
3	−20	−7	76.6466	7.65610	13
3	20	−7	86.3552	7.17374	10
−3	20	7	87.4165	6.01605	11
2	−20	−7	22.5616	3.43426	13

−2	−20	7	30.9913	4.72908	13
2	20	−7	21.0090	2.82777	11
−2	20	7	21.3736	3.72839	10
−1	−20	7	18.7723	3.69337	13
1	−20	−7	21.8460	3.95443	13
1	20	−7	20.1160	2.50575	11
−1	20	7	22.8619	4.39104	10
1	20	−7	19.2761	2.26289	11
0	−20	−7	9.55792	2.82562	13
0	20	7	11.5760	3.47798	10
−1	−20	−7	2.42942	2.92148	13
1	20	7	7.67789	2.95576	10
−1	20	−7	1.04902	1.48719	9
−2	20	−7	4.10163	0.60764	6
2	20	7	5.00213	3.41974	10
−2	20	−7	4.55094	1.01643	9
−3	20	−7	3.54553	2.30404	12
3	20	7	2.47311	3.39783	10
−3	20	−7	1.33750	0.49242	9
−3	20	−7	0.35590	0.50966	9
4	20	7	3.54586	2.96155	10
6	21	−7	8.31824	2.68080	10
−5	−21	7	−0.9524	2.12806	13
5	−21	−7	0.76734	1.83987	13
5	21	−7	2.16525	2.44509	10
−4	−21	7	8.64990	3.11192	13
4	−21	−7	3.90484	2.92919	13
4	21	−7	5.00772	2.60419	10
−3	−21	7	41.9305	5.26348	13
3	−21	−7	42.2499	4.95430	13
−3	21	7	43.8341	3.41262	11
3	21	−7	42.2693	4.53549	10
−2	−21	7	8.87427	2.62317	13
−2	21	7	4.66030	1.89521	10
−2	21	7	6.14979	1.78865	11
2	21	−7	11.2538	2.21873	11
1	−21	−7	6.88324	2.66621	13
−1	−21	7	9.18441	3.03774	13
−1	21	7	5.77490	2.61010	10
0	21	7	60.9923	5.90005	10
−1	−21	−7	3.48971	2.29139	13
−1	21	−7	6.42546	0.86256	6
1	21	7	2.05805	3.02927	10
−1	21	−7	5.25340	1.49648	9
−2	21	−7	17.4274	1.83626	9
−2	21	−7	15.5081	1.23037	6
2	21	7	10.6441	3.06652	10
3	21	7	17.7774	3.36192	10
5	−22	−7	2.23465	1.65929	13
−5	−22	7	3.62623	1.66179	13

5	22	−7	3.86038	2.17144	10
4	−22	−7	13.8706	2.70205	13
−4	−22	7	17.0195	2.77591	13
4	22	−7	14.7936	2.74239	10
3	−22	−7	45.3663	4.74316	13
3	22	−7	45.6999	4.24908	11
−3	22	7	39.1492	2.73851	11
−3	22	7	37.2798	2.75890	11
−2	−22	7	12.1009	3.32001	13
2	−22	−7	15.7561	3.09621	13
−2	22	7	13.5412	2.08495	11
−1	−22	7	32.7461	4.61837	13
1	−22	−7	32.6575	4.58249	13
−1	22	7	31.8007	4.57454	10
0	−22	−7	27.7911	5.29638	13
−1	−22	−7	2.59876	2.45699	13
1	22	7	0.74970	2.73821	10
−1	22	−7	0.32291	1.02846	9
−1	22	−7	2.10317	0.51821	6
2	22	7	11.5017	2.75014	10
−2	22	−7	9.22510	1.20603	9
−2	22	−7	10.6255	0.92325	6
−2	22	−7	10.4852	1.05686	6
3	22	7	21.3419	3.35136	10
4	−23	−7	17.0362	2.99752	13
−4	−23	7	18.7140	2.78186	13
4	23	−7	12.4290	2.02314	11
4	23	−7	14.3270	2.62810	10
3	−23	−7	8.59367	2.28229	13
−3	−23	7	6.29068	2.25857	13
3	23	−7	3.90930	1.55937	11
−2	−23	7	2.04273	2.67392	13
2	−23	−7	4.87116	2.22648	13
−2	23	7	4.75339	1.20312	11
1	−23	−7	15.3769	2.71267	13
−1	−23	7	15.2755	3.01253	13
−1	23	7	20.0538	2.62302	11
−1	23	7	13.9944	2.56723	10
0	−23	−7	2.63036	2.14385	13
0	23	7	4.08338	2.68626	10
−1	−23	−7	2.96766	2.16641	13
−1	23	−7	2.80706	1.15943	9
1	23	7	2.63339	2.45904	10
2	23	7	21.6593	3.68430	10
−2	23	−7	12.8500	1.36264	9
4	−24	−7	4.28752	1.68718	13
−4	−24	7	2.99512	1.59084	13
3	−24	−7	9.38897	2.11704	13
−3	−24	7	10.9089	2.20387	13
3	24	−7	9.54971	1.36848	11

3	24	−7	9.42930	1.33514	11
2	−24	−7	19.8459	3.29440	13
−2	−24	7	15.1805	2.58086	13
−1	−24	7	20.0101	3.36013	13
1	−24	−7	22.3059	4.06157	13
−1	24	7	19.7149	2.27776	11
0	−24	−7	5.52494	1.96659	13
0	24	7	7.02700	2.16671	10
1	24	7	2.63298	1.94380	10
2	24	7	4.46950	2.26654	10
−3	−25	7	30.4173	3.36658	13
3	−25	−7	28.9877	3.61371	13
2	−25	−7	12.6626	2.26293	13
−2	−25	7	19.6129	2.82227	13
−1	−25	7	25.6185	3.42683	13
1	−25	−7	20.2853	3.14987	13
−1	25	7	23.0015	1.79265	11
−1	25	7	21.3901	1.86117	11
0	−25	−7	2.57157	1.59021	13
10	0	−8	−0.4968	2.31054	13
9	0	−8	2.49271	3.26550	13
−9	0	8	2.81513	1.98466	10
8	0	−8	3.54701	3.62397	13
−8	0	8	4.86598	2.50702	10
7	0	−8	64.1035	7.94849	13
−7	0	8	65.8751	6.84589	10
−6	0	8	43.8391	5.25390	10
−5	0	8	1.16818	2.98298	1
−4	0	8	1062.09	65.9772	1
−4	0	8	993.576	66.3704	5
−3	0	8	40.0155	6.06643	1
1	0	−8	63.5654	6.35063	5
0	0	−8	18.0648	3.26098	5
−2	0	−8	0.30344	1.80310	1
−3	0	−8	0.40012	1.47937	8
−4	0	−8	75.7460	6.81855	12
−4	0	−8	74.0013	5.79586	8
−5	0	−8	0.14515	1.98604	12
−5	0	−8	−0.1853	1.30410	8
5	0	8	1.66186	5.26586	13
6	0	8	33.0853	5.11612	13
−6	0	−8	28.3782	2.82054	8
7	0	8	−0.2193	2.58173	13
10	−1	−8	−1.6323	2.27624	13
10	1	−8	3.76179	2.44947	13
9	−1	−8	−3.9342	3.00843	13
−9	−1	8	3.64225	1.90797	10
−9	1	8	1.43293	1.12642	11
9	1	−8	4.98884	3.08662	13
9	1	−8	2.75876	3.39486	13

−8	−1	8	46.9393	5.41682	10
8	−1	−8	47.7864	6.96997	13
8	1	−8	44.9941	7.12698	13
−8	1	8	51.7878	5.25382	10
7	−1	−8	56.2749	7.28680	13
−7	1	8	62.0004	6.77131	10
7	1	−8	87.6075	7.61094	13
−6	−1	8	13.7910	4.29342	1
−6	−1	8	13.6777	3.09624	10
−6	1	8	12.5921	3.09788	10
6	1	−8	15.8917	4.46086	13
−5	−1	8	47.6513	7.16847	1
−4	−1	8	59.0559	7.61507	1
−4	−1	8	58.7542	7.80570	5
−3	−1	8	39.3728	6.17356	1
1	−1	−8	449.285	32.6706	5
1	1	−8	550.140	32.7920	5
0	−1	−8	690.178	45.7536	5
0	1	−8	751.475	45.1806	5
−1	−1	−8	110.723	7.86664	5
−2	−1	−8	86.4118	7.69109	1
−2	1	−8	81.6932	6.68271	4
−2	1	−8	80.9657	6.97144	1
−3	−1	−8	23.0100	2.77979	8
−3	1	−8	23.8022	2.96003	8
−4	−1	−8	3.51458	2.19194	10
−4	−1	−8	2.82772	1.57727	8
−4	1	−8	5.92222	2.39519	12
−4	1	−8	1.50215	1.38063	8
4	1	8	−3.7277	3.67259	13
−5	−1	−8	35.7423	3.56828	8
−5	1	−8	39.3768	3.08629	11
5	1	8	30.8566	5.62557	13
−5	1	−8	36.7848	4.89106	12
−5	1	−8	34.4414	3.31340	8
−6	−1	−8	21.3036	2.55283	8
6	1	8	21.5651	4.40019	13
−6	1	−8	22.5414	2.50110	8
−7	−1	−8	2.64467	1.02183	8
7	1	8	4.24572	2.42253	13
10	−2	−8	4.91949	2.51644	13
10	2	−8	7.11517	2.53243	13
−9	−2	8	7.33363	2.20982	10
9	−2	−8	4.33571	3.29460	13
9	2	−8	7.03434	3.31808	13
−9	2	8	4.91633	1.16292	11
−8	−2	8	88.0453	8.28007	10
8	−2	−8	114.055	9.21817	13
−8	2	8	114.247	8.05699	10
8	2	−8	78.0741	9.35737	13

−7	−2	8	119.509	9.47550	10
7	−2	−8	107.208	9.64853	13
−7	2	8	112.115	9.27262	10
7	2	−8	119.435	10.4307	13
−6	−2	8	29.6884	4.62870	10
−6	−2	8	32.4054	6.50369	1
6	−2	−8	21.1522	3.88679	13
−6	2	8	28.7913	4.30590	10
6	2	−8	25.1592	5.39732	13
−5	−2	8	20.8762	5.31151	1
−4	2	8	35.0173	5.82835	5
−3	−2	8	28.6561	5.60031	1
−3	2	8	15.9111	4.76912	5
1	2	−8	613.672	39.2327	5
0	−2	−8	460.567	31.4554	5
0	2	−8	498.098	29.9741	5
−1	−2	−8	56.7956	5.09696	5
−1	−2	−8	54.6017	6.40821	1
−1	2	−8	44.0994	5.15030	1
−2	−2	−8	4.19293	2.70993	1
−2	2	−8	7.36076	1.95056	8
−2	2	−8	7.25262	1.96333	4
−3	−2	−8	5.36454	2.33368	1
−3	−2	−8	3.55259	1.55804	8
−3	2	−8	5.57177	1.65626	8
−4	−2	−8	2.64917	1.61222	8
−4	−2	−8	3.93468	2.28423	10
4	2	8	−3.1252	3.78587	13
−4	2	−8	2.83821	1.64117	8
−5	−2	−8	22.0570	2.96610	8
−5	2	−8	21.3815	1.74812	11
−5	2	−8	29.6675	4.43935	12
−5	2	−8	23.7424	2.57012	8
5	2	8	20.0884	4.09358	13
−6	−2	−8	2.89498	1.04410	11
−6	−2	−8	3.29485	1.49660	8
−6	2	−8	6.37243	1.20019	8
−7	−2	−8	6.01714	1.33470	8
7	2	8	7.81750	3.13784	13
10	−3	−8	12.9192	2.78279	13
10	3	−8	5.88919	2.51124	13
−9	−3	8	20.5349	3.28367	10
9	−3	−8	16.0784	3.63832	13
−9	3	8	13.2954	2.27354	7
−9	3	8	12.8392	1.58116	11
−8	−3	8	117.591	8.76521	10
8	−3	−8	93.7704	8.90357	13
8	3	−8	113.276	10.4143	13
−8	3	8	88.9642	7.69057	10
−7	−3	8	305.053	23.4714	1

−7	−3	8	294.492	21.3401	10
7	−3	−8	342.245	21.3572	13
7	3	−8	290.129	21.4809	13
−7	3	8	348.149	21.1699	10
−6	−3	8	16.0029	4.83547	1
6	−3	−8	22.2994	3.53897	13
−6	−3	8	18.4223	3.60695	10
−6	3	8	22.3402	3.84869	10
6	3	−8	17.8122	4.13426	13
−5	−3	8	36.7282	7.54517	1
−4	3	8	22.1146	4.81163	5
−3	−3	8	0.68831	2.74400	1
−3	3	8	8.06229	3.68875	5
1	3	−8	55.9540	5.38237	5
0	−3	−8	1169.31	73.3964	5
−1	−3	−8	4.56705	2.17702	5
−1	−3	−8	5.28124	3.04481	1
−1	3	−8	4.27085	2.05169	1
−1	3	−8	3.51400	1.44275	4
−2	−3	−8	135.790	11.8480	1
−2	3	−8	157.936	10.3467	4
−2	3	−8	154.678	9.92134	8
−3	−3	−8	31.3883	4.67678	10
−3	−3	−8	34.7803	5.07670	1
−3	−3	−8	39.0368	3.76263	8
−3	3	−8	46.3104	3.68640	8
−4	−3	−8	10.0739	2.72987	10
−4	−3	−8	11.9506	2.43606	8
−4	3	−8	16.5707	2.46488	8
4	3	8	9.81785	3.68898	13
−4	3	−8	18.4137	3.47313	12
−5	−3	−8	45.8154	4.52063	8
−5	−3	−8	43.6928	4.56517	10
5	3	8	49.9011	7.31126	13
−5	3	−8	43.2978	3.75546	8
−5	3	−8	46.7612	5.39114	12
−6	−3	−8	20.4814	3.00804	8
−6	3	−8	17.2148	2.81223	12
6	3	8	21.2330	4.47588	13
−7	−3	−8	1.02766	1.14833	8
7	3	8	−0.0395	2.39422	13
−7	3	−8	0.44122	0.81674	9
10	−4	−8	5.60696	2.32665	13
10	4	−8	0.31535	2.66158	13
−9	−4	8	24.5246	3.54894	10
9	−4	−8	16.7128	3.75798	13
9	4	−8	25.7155	4.09636	13
−9	4	8	17.9584	2.95438	7
−9	4	8	17.9083	1.90617	11
−8	−4	8	5.73504	2.83181	10

8	−4	−8	12.3773	4.19512	13
−8	4	8	11.2575	2.24636	10
8	4	−8	4.93346	3.29308	13
8	4	−8	7.99053	3.70494	13
7	−4	−8	48.7426	6.53277	13
−7	−4	8	34.1433	5.19251	10
−7	−4	8	39.9854	8.02652	1
7	4	−8	34.3349	5.59911	13
−7	4	8	50.3633	5.28155	10
−6	−4	8	46.9398	7.70778	1
−6	−4	8	39.0210	5.63559	10
6	4	−8	54.5378	6.41187	13
5	−4	−8	153.151	12.8788	2
−5	−4	8	132.024	12.9504	1
−4	−4	8	98.5506	10.6267	1
−3	4	8	26.6163	4.86916	5
1	4	−8	195.576	13.3927	5
0	−4	−8	72.0388	7.21563	5
−1	−4	−8	2.26190	2.94607	1
−1	4	−8	9.31040	1.91469	4
−1	4	−8	7.41365	2.09408	1
−2	−4	−8	11.2413	2.04436	8
−2	4	−8	5.57682	1.55601	4
−2	4	−8	5.29762	1.53000	8
−3	−4	−8	47.6102	4.46088	8
−3	−4	−8	53.4732	6.37778	1
−3	−4	−8	55.4600	5.28228	10
−3	4	−8	43.1512	4.01625	8
−4	−4	−8	263.286	16.8263	8
−4	−4	−8	272.014	17.2487	10
−4	4	−8	241.717	17.0898	12
4	4	8	273.143	18.4802	13
−4	4	−8	249.311	15.8713	11
−4	4	−8	248.222	16.2949	8
−5	−4	−8	51.1596	4.88843	10
−5	−4	−8	50.1436	4.74989	8
5	4	8	57.1501	7.06421	13
−5	4	−8	55.8601	4.14350	8
−5	4	−8	57.6014	5.79810	12
−6	−4	−8	11.1487	1.98566	8
6	4	8	7.84929	3.40490	13
−6	4	−8	10.1307	2.45613	12
−7	−4	−8	107.204	7.00979	8
−7	4	−8	102.813	6.73966	9
7	4	8	104.010	8.33335	13
−9	−5	8	0.66832	2.06712	10
9	−5	−8	1.73812	2.83094	13
−9	5	8	1.91527	1.68575	7
9	5	−8	−0.9637	3.57968	13
−8	−5	8	43.8701	5.54791	10

8	−5	−8	45.6975	6.17635	13
−8	5	8	45.1072	3.71296	11
8	5	−8	45.3442	6.74238	13
7	−5	−8	43.6877	5.84841	13
−7	−5	8	65.0271	8.58611	1
−7	−5	8	59.9842	6.18563	10
−7	5	8	47.6552	5.35920	10
6	−5	−8	365.903	24.6574	13
−6	−5	8	411.302	28.0063	1
−6	−5	8	380.526	25.2693	10
−6	5	8	349.189	24.5184	10
6	5	−8	386.335	25.1508	13
−5	−5	8	140.393	12.9213	1
5	−5	−8	122.059	12.1449	2
−4	−5	8	41.7328	6.80844	1
4	−5	−8	32.6045	5.96915	2
3	−5	−8	483.980	32.3019	2
−3	5	8	486.927	32.6480	5
−2	5	8	13.1566	3.76351	5
1	5	−8	291.603	18.6592	5
0	−5	−8	33.9151	5.05422	5
−1	−5	−8	4.83840	2.12292	5
−1	−5	−8	5.58473	3.05121	1
−1	5	−8	4.99775	1.54264	4
−2	−5	−8	37.7379	6.06724	1
−2	−5	−8	33.0923	3.46888	8
−2	5	−8	27.6380	2.91582	8
−2	5	−8	22.5838	2.61283	4
−3	−5	−8	34.5061	3.72898	8
−3	−5	−8	35.5193	4.50017	10
−3	−5	−8	34.2473	5.25314	1
−3	5	−8	26.2955	3.01327	8
−4	−5	−8	11.6634	2.33334	8
−4	−5	−8	15.4433	2.94326	10
−4	5	−8	13.6143	2.02210	8
4	5	8	13.1509	4.47985	13
−4	5	−8	14.5366	2.95614	12
−5	−5	−8	21.8007	3.84805	10
−5	−5	−8	24.4516	3.85101	8
−5	5	−8	21.8215	3.10811	9
−5	5	−8	26.7828	4.03297	12
5	5	8	22.3428	4.47374	13
−6	−5	−8	2.50627	1.65968	8
−6	5	−8	1.62266	0.92969	9
6	5	8	4.47935	3.34350	13
−6	5	−8	2.20909	2.10193	12
−6	5	−8	1.70105	0.69348	6
−7	−5	−8	5.58257	1.46656	8
7	5	8	5.45397	2.63200	13
9	−6	−8	8.42092	3.07148	13

9	6	−8	17.2268	3.91024	13
−9	6	8	9.53135	2.08887	7
−8	−6	8	127.027	10.3421	10
8	−6	−8	136.781	10.7596	13
−8	6	8	128.607	9.35477	7
−8	6	8	122.958	8.51958	11
8	6	−8	145.676	11.0552	13
−7	−6	8	132.466	12.1529	1
−7	−6	8	122.553	9.52369	10
7	−6	−8	91.0466	9.20197	13
7	6	−8	118.213	9.51582	13
−7	6	8	89.1265	8.05913	10
7	6	−8	114.867	9.21905	13
−6	−6	8	191.157	16.8792	1
6	−6	−8	226.381	13.5537	13
−6	−6	8	170.082	13.9241	10
6	6	−8	165.260	13.9889	13
5	−6	−8	288.459	21.1085	2
−5	−6	8	267.580	21.5952	1
−5	6	8	274.948	18.9413	10
−4	−6	8	21.5419	5.41298	1
4	−6	−8	17.8836	4.31628	2
3	−6	−8	169.936	13.6406	2
−3	6	8	167.431	13.1198	5
2	−6	−8	289.501	19.9050	2
2	6	−8	261.105	18.8934	5
−2	6	8	292.423	20.7980	5
0	6	−8	104.583	7.60327	4
−1	−6	−8	37.0199	5.51180	5
−1	−6	−8	33.8833	6.19425	1
−2	−6	−8	63.3961	5.07068	8
−2	−6	−8	67.6392	7.90048	1
−2	6	−8	57.6694	4.53884	8
−2	6	−8	54.8228	4.19968	4
−3	−6	−8	68.0040	7.43675	1
−3	−6	−8	70.9258	5.67898	8
−3	−6	−8	68.3581	6.13807	10
−3	6	−8	58.1171	4.37816	11
−3	6	−8	59.7905	4.69994	8
−3	6	−8	60.5526	5.86471	12
−4	−6	−8	4.79714	2.93092	1
−4	−6	−8	4.43052	1.99198	8
−4	−6	−8	5.17147	2.37214	10
−4	6	−8	6.24375	1.38111	8
4	6	8	0.76525	4.05854	13
−4	6	−8	6.53014	1.31794	9
−4	6	−8	5.51490	3.00171	12
−5	−6	−8	60.1331	5.54143	8
−5	−6	−8	63.3023	6.18685	10
−5	6	−8	64.5732	6.10292	12

5	6	8	64.7468	7.73494	13
−5	6	−8	71.2704	4.32348	6
−5	6	−8	63.5759	4.63618	9
−6	−6	−8	0.97753	1.69317	8
−6	−6	−8	1.65284	1.72906	10
−6	6	−8	3.63837	2.17694	12
−6	6	−8	3.28238	1.08940	9
−6	6	−8	4.29273	0.61406	6
6	6	8	1.51642	3.26820	13
−7	6	−8	7.51887	0.91010	9
7	6	8	5.97948	2.57653	13
9	−7	−8	54.1657	6.27211	13
−9	−7	8	59.0561	4.98253	10
9	7	−8	56.6871	5.65908	13
−9	7	8	50.1056	4.58253	7
−8	−7	8	47.7057	5.69145	10
8	−7	−8	42.1717	6.42700	13
8	7	−8	62.3022	7.26993	13
−8	7	8	47.2293	4.63350	7
−8	7	8	51.7899	3.72720	11
7	−7	−8	169.714	11.6308	13
−7	−7	8	135.855	13.6220	1
−7	−7	8	139.210	11.4475	10
7	7	−8	134.272	11.9445	13
6	−7	−8	33.6422	5.32182	13
−6	7	8	31.8636	4.09768	10
−5	−7	8	2.10248	3.69022	1
5	−7	−8	2.86794	3.34382	2
4	−7	−8	37.1405	6.12208	2
3	−7	−8	88.5831	9.42134	2
−3	7	8	103.555	9.37112	5
2	−7	−8	49.6433	6.57982	2
2	7	−8	45.1877	4.92715	5
−2	7	8	62.9980	7.31252	5
1	−7	−8	330.160	23.0924	2
0	7	−8	24.4277	2.88192	4
−1	−7	−8	27.5464	4.60106	5
−1	−7	−8	28.2148	5.40694	1
−1	7	−8	23.2207	3.01140	8
−1	7	−8	19.0022	2.49275	4
−2	−7	−8	24.6383	5.38400	1
−2	7	−8	21.8861	2.54406	8
−3	−7	−8	11.6030	2.29254	8
−3	−7	−8	14.9275	4.07757	1
−3	−7	−8	12.1763	2.90035	10
−3	7	−8	6.88575	1.38322	8
−3	7	−8	5.20089	0.78173	11
−3	7	−8	8.97965	1.16878	11
3	7	8	15.4060	3.57767	13
−4	−7	−8	11.0454	2.40662	8

−4	−7	−8	10.2017	3.75145	1
−4	−7	−8	13.3288	3.01328	10
4	7	8	11.1754	3.73006	13
−5	−7	−8	10.6874	2.61531	10
−5	−7	−8	11.2606	2.49673	8
5	7	8	10.8327	4.74654	13
−5	7	−8	15.3662	1.30797	6
−5	7	−8	15.0228	3.00354	12
−5	7	−8	14.0735	1.68751	9
−6	−7	−8	88.1479	6.61170	8
−6	−7	−8	87.9843	6.67382	10
6	7	8	97.5226	8.58783	13
−6	7	−8	87.6560	6.98156	12
−6	7	−8	83.3080	5.42097	6
−6	7	−8	80.2596	5.62283	9
−7	7	−8	7.40526	0.85666	9
7	7	8	6.24901	2.51475	13
−9	−8	8	30.3234	4.02553	10
9	−8	−8	32.3933	4.56463	13
−9	8	8	34.8858	3.42552	7
8	−8	−8	20.7996	3.73524	13
−8	8	8	22.4892	3.22145	7
−8	8	8	22.0647	2.08468	11
7	−8	−8	51.5817	6.69839	13
−7	8	8	50.4053	4.05264	11
−7	8	8	52.0937	4.75811	7
6	−8	−8	28.8603	5.37375	13
−6	8	8	37.3845	4.44587	10
5	−8	−8	54.7416	8.53301	2
−5	−8	8	61.8455	8.96754	1
−5	8	8	52.1723	5.96158	10
5	8	−8	49.6642	6.24488	10
4	−8	−8	5.22229	3.41175	2
3	−8	−8	2.78291	3.40975	2
2	−8	−8	12.9805	4.10828	2
−2	8	8	13.1635	4.10109	5
2	8	−8	16.4538	2.45775	5
1	−8	−8	22.5175	4.41997	2
−1	8	8	20.4682	4.96689	5
0	−8	−8	3.83925	3.07642	2
0	8	−8	1.95400	1.34330	4
−1	−8	−8	59.3333	8.31269	1
−1	8	−8	76.8671	5.52623	4
−1	8	−8	84.3436	5.54459	4
−1	8	−8	81.3148	5.64678	8
−2	−8	−8	3.62494	3.92368	1
−2	8	−8	9.04030	1.53713	8
−3	−8	−8	46.7834	5.65556	10
−3	−8	−8	38.9862	4.70154	8
−3	−8	−8	35.1798	7.35760	1

3	8	8	52.8585	7.48493	13
−3	8	−8	58.6911	5.67162	12
−3	8	−8	56.7501	3.75371	9
−3	8	−8	50.4322	3.89504	8
−3	8	−8	66.7614	6.35056	14
−4	−8	−8	283.531	19.4555	8
−4	−8	−8	293.949	20.0448	10
−4	−8	−8	290.092	21.8119	1
4	8	8	288.756	20.5596	13
−4	8	−8	303.370	18.5681	9
−4	8	−8	321.292	19.8656	12
−4	8	−8	317.462	18.4377	6
−5	−8	−8	19.0682	3.63280	10
−5	−8	−8	16.4278	2.75914	8
−5	8	−8	15.1416	1.71373	9
5	8	8	15.2687	4.28673	13
−5	8	−8	13.9827	1.31417	6
−5	8	−8	14.1007	3.07119	12
−6	−8	−8	4.63710	1.80533	10
−6	−8	−8	6.03461	1.94057	8
−6	8	−8	2.32562	0.44293	6
−6	8	−8	1.05921	2.07413	12
6	8	8	4.89967	3.26137	13
−6	8	−8	3.05741	0.87433	9
−6	8	−8	2.17939	0.52201	6
7	8	8	30.5391	4.18074	13
9	−9	−8	7.68191	2.40479	13
−9	−9	8	3.04546	2.18122	10
8	−9	−8	52.4639	6.45365	13
−8	−9	8	59.1120	6.38105	10
−8	9	8	63.8095	5.14196	7
7	−9	−8	63.3438	7.23592	13
−7	−9	8	84.2820	7.90947	10
−7	−9	8	82.4570	9.95947	1
−7	9	8	77.7801	6.13521	7
−7	9	8	77.9595	5.40035	11
−6	−9	8	112.738	11.8904	1
6	−9	−8	76.7838	8.10313	13
−6	−9	8	99.2200	8.49782	10
−6	9	8	78.6498	6.86069	10
5	−9	−8	1.31072	3.56600	2
−5	−9	8	7.94397	4.51005	1
4	−9	−8	34.4003	6.51837	2
3	−9	−8	77.0422	8.46160	2
2	−9	−8	8.55908	3.43532	2
1	−9	−8	93.0991	9.36595	2
−1	9	8	95.1907	9.72302	5
0	−9	−8	101.936	10.8614	1
0	−9	−8	106.183	10.4919	2
0	9	−8	117.848	8.07928	4

0	9	−8	126.286	8.13333	4
−1	−9	−8	68.5425	8.44351	1
−1	−9	−8	72.9540	8.06463	2
−1	9	−8	61.1921	5.40093	4
−2	−9	−8	47.2830	7.49757	1
−2	−9	−8	58.1479	6.26353	10
−2	9	−8	58.9193	3.92471	11
−3	−9	−8	10.5601	3.20836	10
−3	−9	−8	9.84623	4.09012	1
−3	9	−8	17.4826	1.54252	6
−3	9	−8	21.9533	3.70908	12
−3	9	−8	19.2141	3.09690	14
−3	9	−8	18.3518	1.89889	9
3	9	8	9.85254	4.15866	13
−4	9	−8	11.7993	2.83148	12
4	9	8	20.9749	4.08813	13
−4	9	−8	14.9775	1.82044	9
−4	9	−8	12.4335	1.39724	6
−5	−9	−8	19.0468	2.91313	8
−5	−9	−8	16.3796	3.10609	10
5	9	8	14.2573	4.76108	13
−5	9	−8	21.5546	1.79163	9
−5	9	−8	20.2300	3.32550	12
−5	9	−8	18.9864	1.51876	6
−6	−9	−8	−0.3712	1.67786	8
−6	−9	−8	0.71625	1.62817	10
−6	9	−8	0.18013	2.05670	12
−6	9	−8	1.25362	0.34728	6
−6	9	−8	1.13071	0.32501	6
6	9	8	3.62499	2.92108	13
−6	9	−8	1.00811	0.69419	9
7	9	8	35.2570	4.16041	13
−9	−10	8	36.5090	4.09528	10
8	−10	−8	21.0661	3.63980	13
−8	10	8	22.8204	2.66293	7
−7	−10	8	51.8720	9.93994	1
−7	−10	8	43.9298	6.28719	10
−7	10	8	63.4162	4.17764	11
6	−10	−8	173.825	12.0873	13
−6	−10	8	132.535	15.0772	1
−6	10	8	176.847	11.4536	10
5	−10	−8	4.48210	3.16778	13
−5	−10	8	4.92440	4.68878	1
5	10	−8	0.83457	2.99291	10
−5	10	8	2.13220	2.16332	10
4	−10	−8	22.4412	5.26383	2
−4	10	8	23.7897	4.29510	10
3	−10	−8	100.263	10.5989	2
2	−10	−8	21.6587	4.87130	2
1	−10	−8	197.429	15.8286	2

0	−10	−8	320.579	22.2943	2
0	−10	−8	316.131	22.9422	1
0	10	−8	278.732	19.6619	4
−1	−10	−8	45.7604	7.28779	1
−1	−10	−8	42.4883	6.34022	2
−2	−10	−8	69.1700	8.88467	2
−2	−10	−8	70.5791	9.10856	1
−2	−10	−8	63.6355	7.03635	10
−2	10	−8	79.7694	4.74623	11
−2	10	−8	80.9673	6.55267	12
−2	10	−8	81.3468	5.15236	9
−3	−10	−8	34.6361	5.16620	10
−3	−10	−8	42.1683	7.08732	1
−3	10	−8	38.4111	4.84812	14
−3	10	−8	38.0405	4.68793	12
−3	10	−8	37.5436	2.70124	6
−3	10	−8	39.6907	3.03498	9
3	10	8	35.0994	5.84086	13
−4	−10	−8	9.96313	2.99210	10
−4	−10	−8	15.9732	4.93757	1
−4	−10	−8	11.1869	3.04924	8
−4	10	−8	7.53678	3.14207	14
−4	10	−8	9.38734	1.00692	6
−4	10	−8	8.44577	1.25366	9
4	10	8	11.9601	3.66368	13
−4	10	−8	6.78093	2.66827	12
−5	−10	−8	9.77095	2.45703	8
−5	10	−8	4.79944	2.67623	12
−5	10	−8	6.75187	0.68137	6
−5	10	−8	6.53511	0.74043	6
5	10	8	4.41337	3.55860	13
−5	10	−8	8.38429	1.06447	9
−6	−10	−8	12.6155	2.31240	8
−6	−10	−8	13.1818	2.40130	10
−6	10	−8	11.3240	1.12775	9
6	10	8	10.9799	3.33826	13
−6	10	−8	11.6765	2.64336	12
−6	10	−8	13.1522	1.19908	9
−9	−11	8	50.0236	4.44854	10
9	−11	−8	36.3604	4.21916	13
8	−11	−8	2.16510	2.84910	13
−8	−11	8	2.52483	1.98617	10
−8	11	8	0.28653	1.34192	7
−7	−11	8	36.4279	5.17141	10
7	−11	−8	25.8494	4.13628	13
−7	11	8	22.2033	2.32955	11
−7	11	8	24.4059	2.56379	11
6	−11	−8	78.2186	7.77636	13
−6	11	8	77.8912	5.58417	11
5	−11	−8	57.3160	7.15819	13

-5	-11	8	80.2272	7.09641	13
-5	11	8	70.1175	6.56679	10
5	11	-8	80.4366	7.58779	10
4	-11	-8	17.6625	5.56761	2
-4	-11	8	16.1815	4.26461	13
-4	11	8	10.2624	2.88391	10
4	11	-8	12.0107	3.39626	10
3	-11	-8	9.94491	4.88673	2
3	11	-8	2.62833	2.78176	10
-3	11	8	8.22473	2.72694	10
2	-11	-8	28.6723	6.11780	2
1	-11	-8	53.7393	7.53448	2
0	-11	-8	23.8210	5.53540	2
-1	-11	-8	301.881	23.0644	2
-1	-11	-8	313.595	23.9481	1
-1	11	-8	326.494	19.5580	11
-2	-11	-8	72.5693	9.79064	2
-2	-11	-8	60.3979	6.78795	10
-2	-11	-8	69.2628	8.88410	1
2	11	8	65.4541	7.77583	13
-3	-11	-8	23.4382	5.43843	1
-3	-11	-8	19.3663	3.66414	10
-3	11	-8	22.9350	3.43091	14
-3	11	-8	20.1898	3.43788	12
-3	11	-8	22.2484	2.18552	9
3	11	8	19.9676	4.02292	13
-3	11	-8	22.3523	1.69737	6
-4	-11	-8	4.56650	3.49775	1
-4	-11	-8	9.14768	3.13046	10
4	11	8	7.75407	3.36273	13
-4	11	-8	2.50406	2.61357	14
-4	11	-8	4.17888	2.72577	12
-4	11	-8	4.96927	0.76791	6
-4	11	-8	6.76038	1.08036	9
-5	-11	-8	0.21347	2.19226	10
-5	-11	-8	0.31273	1.92568	8
-5	11	-8	0.17498	0.33176	6
-5	11	-8	1.18890	1.67733	12
-5	11	-8	-0.1101	0.71357	9
5	11	8	0.64829	3.68368	13
-5	11	-8	0.04948	0.38407	6
-6	-11	-8	5.05161	1.89079	10
-6	-11	-8	4.74406	1.86373	8
-6	11	-8	4.19989	2.08164	12
6	11	8	4.55940	2.58145	13
8	-12	-8	41.8418	5.08027	13
-8	-12	8	49.1863	5.45273	10
7	-12	-8	32.1053	5.09128	13
6	-12	-8	7.63442	3.33622	13
-6	-12	8	19.6649	6.25869	1

−6	12	8	6.06491	1.47979	11
5	−12	−8	38.8247	5.22055	13
−5	−12	8	30.1057	5.40598	13
5	12	−8	40.1669	5.82510	10
−5	12	8	42.4469	4.82817	10
−4	−12	8	19.1239	3.49239	13
4	−12	−8	21.4423	3.83182	13
−4	12	8	20.5733	3.78419	10
4	12	−8	24.1829	4.21033	10
3	−12	−8	22.0566	5.46872	2
−3	12	8	17.8245	3.43545	10
2	−12	−8	38.2233	6.89073	2
−2	12	8	28.1929	4.84690	10
2	12	−8	25.8521	4.09202	10
1	−12	−8	119.390	11.7253	2
0	−12	−8	20.8085	4.96991	2
0	12	−8	14.0628	2.48219	11
−1	−12	−8	22.9022	5.05642	2
−1	−12	−8	24.4283	5.76310	1
−1	12	−8	14.6510	3.08416	14
−1	12	−8	20.0842	2.16649	11
1	12	8	28.2789	4.91814	10
−2	−12	−8	30.9715	6.22055	1
−2	12	−8	14.6156	3.29407	12
−2	12	−8	19.3759	1.51774	6
−2	12	−8	13.3076	1.84690	9
−2	12	−8	18.8651	3.67382	14
−3	−12	−8	1.90599	4.51682	1
−3	−12	−8	2.13590	2.74474	10
−3	12	−8	1.39727	0.96191	9
3	12	8	0.51438	2.83911	13
−3	12	−8	0.21146	2.24781	14
−3	12	−8	0.92191	2.46177	12
−3	12	−8	1.49874	0.61406	6
−4	−12	−8	66.0319	8.23560	1
−4	−12	−8	65.8454	6.47409	10
−4	12	−8	57.9565	3.79639	6
−4	12	−8	51.8226	7.04172	14
4	12	8	55.4685	6.95708	13
−4	12	−8	55.6625	4.00797	9
−4	12	−8	61.5865	6.19250	12
−5	−12	−8	7.69837	2.81605	10
−5	12	−8	13.1611	1.35525	9
−5	12	−8	13.8251	1.46425	9
−5	12	−8	11.1762	2.38143	12
5	12	8	4.62566	3.32305	13
−6	−12	−8	0.13649	1.62833	10
6	12	8	0.77774	2.07130	13
−6	12	−8	0.92950	1.96930	12
8	−13	−8	0.98006	2.35602	13

−8	−13	8	3.17573	2.50476	10
7	−13	−8	21.6329	3.96311	13
−7	−13	8	22.8565	4.57880	10
7	13	−8	23.0805	4.57046	10
6	13	−8	37.1491	6.32771	10
−6	13	8	32.0881	2.81175	11
5	−13	−8	44.9597	5.63063	13
−5	−13	8	61.8390	5.76637	13
5	13	−8	61.1986	6.70987	10
−5	13	8	41.7908	4.68006	10
−4	−13	8	11.6275	3.43326	13
4	−13	−8	11.0690	3.23915	13
−4	13	8	10.6677	2.68460	10
4	13	−8	6.77447	3.08127	10
−3	−13	8	12.8325	3.34042	13
3	−13	−8	13.5178	5.04449	2
3	13	−8	24.1196	4.41702	10
−3	13	8	21.5259	3.76357	10
2	−13	−8	2.59947	3.93847	2
2	13	−8	1.83858	2.22382	10
−2	13	8	3.14221	2.72718	10
1	−13	−8	80.3121	9.47291	2
−1	13	8	80.9369	7.67996	10
1	13	−8	65.4112	6.17105	10
0	13	−8	147.684	12.0375	14
0	13	−8	155.476	10.2941	11
0	13	8	166.324	12.6511	10
−1	−13	−8	4.34186	4.51721	1
1	13	8	−0.3981	3.38289	10
−1	13	−8	0.57254	2.49043	14
−1	13	−8	3.05669	1.02658	11
−1	13	−8	2.98021	0.91146	11
−2	−13	−8	0.05359	3.59556	1
−2	−13	−8	1.68726	2.82034	10
−2	13	−8	3.40989	2.91181	12
−2	13	−8	2.74066	0.60667	6
−2	13	−8	1.21006	2.47747	14
−2	13	−8	2.37284	1.07353	9
−3	−13	−8	11.0118	4.72739	1
−3	−13	−8	10.9140	3.45818	10
−3	13	−8	8.68255	2.81697	12
−3	13	−8	8.64263	0.95182	6
−3	13	−8	10.0908	3.35610	14
−3	13	−8	8.07711	1.31344	9
−4	−13	−8	17.3886	3.71534	10
−4	13	−8	16.2747	1.35704	6
−4	13	−8	21.0704	1.80346	9
−4	13	−8	21.0887	3.41550	14
−4	13	−8	18.3938	3.31567	12
−4	13	−8	15.0789	1.28210	6

−5	−13	−8	2.34691	1.76764	10
−5	13	−8	−0.8151	1.75667	12
−6	−13	−8	1.93572	1.65498	10
−6	13	−8	2.57400	1.39292	12
8	−14	−8	1.55112	1.72311	13
−8	−14	8	1.11195	2.20099	10
7	−14	−8	12.0729	3.03185	13
7	14	−8	0.36191	3.50168	10
6	−14	−8	107.580	9.54339	13
6	14	−8	97.7846	9.67001	10
−6	14	8	115.588	7.24193	11
−6	14	8	115.288	7.16481	11
−5	−14	8	41.9716	5.62226	13
5	−14	−8	30.3930	5.81074	13
5	14	−8	41.2942	5.79098	10
−5	14	8	27.9154	3.43030	11
−4	−14	8	8.86827	3.39721	13
4	−14	−8	11.8071	2.86118	13
−4	14	8	12.1363	2.72344	10
4	14	−8	9.51138	3.22581	10
3	−14	−8	5.31368	2.97749	13
−3	−14	8	7.28475	3.24960	13
−3	14	8	6.06835	2.63223	10
3	14	−8	7.43499	3.28561	10
−2	−14	8	23.0931	4.16996	13
2	−14	−8	18.5033	3.63120	13
2	14	−8	23.0277	3.84990	10
−2	14	8	19.3468	3.87154	10
−1	−14	8	16.5353	3.49290	13
1	14	−8	18.7893	3.80765	14
1	14	−8	19.9804	3.39438	10
0	−14	−8	12.2997	3.20410	13
0	14	−8	12.1147	1.89504	11
0	14	8	19.0482	4.41883	10
0	14	−8	12.3013	2.89063	14
−1	−14	−8	150.446	16.0941	1
1	14	8	149.739	12.5248	10
−1	14	−8	160.306	11.5061	14
−2	−14	−8	36.2740	7.49135	1
−2	14	−8	38.8755	2.70467	6
−2	14	−8	39.4837	3.29887	9
−2	14	−8	36.8268	5.08681	14
−2	14	−8	38.5845	5.11155	12
−3	−14	−8	26.7799	6.43962	1
−3	−14	−8	28.6875	5.31465	10
−3	14	−8	34.1752	4.63221	12
−3	14	−8	31.4319	2.22345	6
−3	14	−8	32.5556	2.76359	9
−4	−14	−8	−0.4108	1.95355	10
−4	14	−8	0.72208	0.37078	6

−4	14	−8	1.45387	0.77063	9
−4	14	−8	1.84090	0.42454	6
−4	14	−8	−0.1257	2.56926	12
−4	14	−8	−1.4677	2.58079	14
−5	−14	−8	18.8822	3.73271	10
−5	14	−8	14.4355	2.96007	12
−6	−14	−8	18.7046	2.62186	10
−6	14	−8	20.0377	3.08967	12
8	−15	−8	1.62570	1.61705	13
−8	−15	8	2.53367	2.19500	10
7	−15	−8	21.9763	3.52723	13
7	15	−8	20.4655	4.24364	10
6	−15	−8	47.4747	5.91541	13
6	15	−8	49.9217	5.80716	10
−5	−15	8	137.985	11.7954	13
5	−15	−8	183.734	12.7115	13
−5	15	8	193.367	10.7867	11
5	15	−8	143.981	12.7461	10
4	−15	−8	18.8300	3.71054	13
−4	−15	8	32.4727	5.19744	13
−4	15	8	20.9452	3.54328	10
3	−15	−8	−0.2087	2.79005	13
−3	−15	8	1.07188	2.43612	13
3	15	−8	7.00919	2.81994	10
−3	15	8	−0.6160	2.20575	10
−2	−15	8	23.2937	3.60521	13
−2	15	8	16.3750	4.03557	10
2	15	−8	19.7077	3.66787	10
−1	−15	8	3.04845	2.80762	13
1	−15	−8	3.07004	2.75800	13
−1	15	8	5.57658	3.16096	10
1	15	−8	6.79740	2.99937	14
1	15	−8	3.38333	2.45245	10
0	−15	−8	6.78049	2.37917	13
0	15	8	6.13032	3.58993	10
−1	15	−8	18.7478	3.34482	14
−2	−15	−8	49.2531	8.48180	1
−2	15	−8	58.7465	5.53516	12
−2	15	−8	54.7587	3.95430	9
2	15	8	41.2635	7.13154	10
−2	15	−8	60.3082	6.09365	14
−2	15	−8	54.7144	3.59723	6
−3	−15	−8	14.1899	3.81803	10
−3	15	−8	16.8780	2.04759	9
−3	15	−8	19.6746	3.57960	14
−3	15	−8	19.8188	1.46272	6
−3	15	−8	18.2416	3.41550	12
−4	−15	−8	23.8204	3.85960	10
−4	15	−8	30.5237	2.07450	9
−4	15	−8	29.2010	2.17166	9

−4	15	−8	30.3722	4.82860	14
−5	−15	−8	6.18569	2.48038	10
−5	15	−8	5.39354	2.26221	12
7	−16	−8	6.32536	2.40743	13
7	16	−8	7.69665	3.21361	10
6	16	−8	17.1440	4.19232	10
−5	−16	8	8.33228	2.65588	13
5	−16	−8	3.32462	3.41075	13
−5	16	8	6.74433	1.34704	11
5	16	−8	7.10719	3.65737	10
−4	−16	8	24.1670	4.20305	13
4	−16	−8	28.5323	4.65830	13
4	16	−8	20.9056	4.09317	10
−4	16	8	27.2483	4.06881	10
3	−16	−8	33.0401	5.27221	13
−3	−16	8	41.4590	5.78670	13
3	16	−8	40.4946	5.13226	10
−3	16	8	35.7808	4.70931	10
2	−16	−8	39.4466	5.01038	13
−2	−16	8	37.9721	5.25654	13
−2	16	8	48.7228	6.20656	10
2	16	−8	45.2788	5.27639	10
−1	−16	8	33.6423	5.36876	13
1	−16	−8	38.3548	5.18307	13
1	16	−8	33.3407	3.63897	11
1	16	−8	36.1307	4.51937	14
−1	16	8	26.5821	5.38433	10
0	16	−8	29.9195	4.60891	14
0	16	−8	22.0449	2.50263	11
0	16	−8	21.9089	2.59497	11
−1	16	−8	24.2236	3.65578	14
1	16	8	19.4334	4.50450	10
−2	−16	−8	36.6592	7.69403	1
−2	16	−8	36.1005	3.17591	9
−2	16	−8	35.2216	2.44288	6
−2	16	−8	31.1994	4.65151	12
−2	16	−8	46.3648	5.73677	14
2	16	8	28.9800	5.53590	10
−3	−16	−8	58.0784	6.88658	10
−3	16	−8	66.9739	4.09824	6
−4	−16	−8	36.2286	4.35099	10
−4	16	−8	34.8878	4.69613	12
−4	16	−8	44.1158	5.06138	14
−5	−16	−8	1.00437	1.59705	10
−5	16	−8	2.60813	2.02202	12
7	−17	−8	3.30628	2.07487	13
7	17	−8	0.22454	2.03917	10
6	−17	−8	1.15096	2.37764	13
6	17	−8	2.48670	2.46664	10
−5	−17	8	7.07753	2.73472	13

5	−17	−8	6.51473	2.89083	13
5	17	−8	7.52055	3.66796	10
−4	−17	8	4.57622	2.89412	13
4	−17	−8	11.7836	3.01464	13
−4	17	8	9.75390	1.71424	11
3	−17	−8	72.4180	6.47040	13
−3	−17	8	61.4377	6.60778	13
−3	17	8	70.6576	6.26585	10
3	17	−8	63.9009	7.05065	10
−2	−17	8	16.1606	3.72737	13
2	−17	−8	25.8321	4.82286	13
−2	17	8	24.7779	4.54519	10
2	17	−8	18.4738	3.68428	10
−1	−17	8	0.00027	2.14888	13
1	−17	−8	2.94794	2.31596	13
−1	17	8	3.69138	2.94295	10
1	17	−8	−1.0003	2.99122	14
1	17	−8	−0.5806	1.70284	11
0	−17	−8	47.3380	5.59950	13
0	17	8	51.3322	6.48744	10
0	17	−8	41.0524	3.15945	11
0	17	−8	40.5288	3.05164	11
0	17	−8	35.3353	5.96634	14
−1	17	−8	37.1952	4.90879	14
−2	17	−8	18.1129	2.24981	9
−2	17	−8	26.5935	3.95056	14
2	17	8	18.7346	3.96231	10
−2	17	−8	21.1655	1.60524	6
−3	−17	−8	1.61460	2.07783	10
−3	17	−8	1.70756	2.30137	12
−3	17	−8	1.92765	0.48331	6
−3	17	−8	1.38909	0.43122	6
−3	17	−8	3.50470	2.79187	14
−3	17	−8	2.04332	0.91476	9
3	17	8	3.68679	2.62465	10
−4	−17	−8	8.47294	3.01777	10
−4	17	−8	6.70728	2.50360	12
−5	−17	−8	12.3248	2.27480	10
−5	17	−8	11.4266	2.23340	12
7	−18	−8	3.62978	1.84620	13
7	18	−8	6.87882	2.21215	10
6	−18	−8	10.3406	2.90432	13
6	18	−8	6.48009	2.60774	10
−5	−18	8	40.9289	4.91226	13
5	−18	−8	39.8182	5.30940	13
5	18	−8	39.4884	5.59996	10
−4	−18	8	8.47494	2.95262	13
4	−18	−8	5.36359	2.74168	13
4	18	−8	6.86652	3.22488	10
−4	18	8	2.81098	1.23730	11

−3	−18	8	6.43094	2.85506	13
3	−18	−8	7.00313	3.28107	13
−3	18	8	9.20449	2.85018	10
3	18	−8	9.81919	3.49398	10
2	−18	−8	21.3137	3.85459	13
−2	−18	8	30.3969	5.00403	13
−2	18	8	21.6749	4.38761	10
2	18	−8	26.7729	4.27832	10
1	−18	−8	6.30228	2.99468	13
−1	−18	8	3.69704	2.43982	13
1	18	−8	5.18773	3.41709	14
1	18	−8	5.41288	1.70725	11
−1	18	8	4.20348	3.01497	10
0	−18	−8	−2.0790	3.02488	13
0	18	8	−4.3283	3.27027	10
−1	−18	−8	58.5429	5.98022	13
−1	18	−8	66.8589	6.77537	14
1	18	8	62.2880	7.49465	10
−2	18	−8	22.1015	2.36158	9
−2	18	−8	19.3716	1.58794	6
2	18	8	26.0205	4.86311	10
−3	18	−8	11.4310	1.33060	9
−3	18	−8	9.85088	2.67425	12
3	18	8	5.64083	3.70723	10
−3	18	−8	11.2437	3.07236	14
−3	18	−8	9.51076	0.86094	6
−4	−18	−8	1.27253	2.32402	10
−4	18	−8	1.77920	2.07741	12
6	−19	−8	10.7079	2.29792	13
6	19	−8	12.3180	2.70109	10
−5	−19	8	19.8589	3.74877	13
5	−19	−8	22.2372	4.20810	13
5	19	−8	18.3468	3.48123	10
−4	−19	8	27.6310	4.60281	13
4	−19	−8	17.6765	3.47266	13
−4	19	8	19.0556	2.27019	11
4	19	−8	27.7419	4.47903	10
−3	−19	8	2.21515	2.68496	13
3	−19	−8	0.71164	2.99584	13
3	19	−8	3.41163	2.49428	10
−3	19	8	0.11250	2.04063	10
−3	19	8	−0.8718	1.53227	11
2	−19	−8	5.20495	2.58846	13
−2	19	8	6.66907	2.79554	10
1	−19	−8	34.2221	4.81614	13
−1	−19	8	31.4314	5.02168	13
1	19	−8	32.3764	3.39260	11
−1	19	8	33.9318	5.35543	10
1	19	−8	37.6594	5.01025	14
1	19	−8	33.9834	3.59253	11

0	−19	−8	3.14339	2.88279	13
0	19	−8	13.6766	3.25306	14
0	19	8	4.81718	3.27347	10
−1	−19	−8	33.0507	5.38910	13
1	19	8	27.8613	4.85347	10
−1	19	−8	42.1420	3.46652	9
−1	19	−8	49.0323	5.60953	14
−2	19	−8	15.4379	1.39723	6
−2	19	−8	17.4706	3.53128	14
2	19	8	18.1820	4.55231	10
−2	19	−8	20.2309	1.89269	9
−3	19	−8	1.51630	0.61827	9
−3	19	−8	2.03948	0.62313	9
3	19	8	2.98874	3.41720	10
−3	19	−8	0.82698	1.98856	12
−4	−19	−8	4.02918	2.17997	10
−4	19	−8	2.60305	1.81326	12
6	−20	−8	16.2959	2.74077	13
6	20	−8	12.6561	2.87720	10
5	−20	−8	6.97173	2.77214	13
−5	−20	8	6.72367	2.58599	13
5	20	−8	4.89419	2.01419	10
4	−20	−8	18.3992	3.42833	13
−4	−20	8	22.7345	3.42312	13
−4	20	8	20.0805	1.77962	11
−4	20	8	18.7424	1.75844	11
4	20	−8	23.8567	3.95617	10
−3	−20	8	17.5367	3.22864	13
3	−20	−8	17.7933	3.18170	13
3	20	−8	17.5115	3.35192	10
−3	20	8	17.5202	2.47508	11
2	−20	−8	56.9154	5.62163	13
−2	−20	8	53.0686	5.80559	13
−2	20	8	54.1956	5.30232	10
2	20	−8	45.8814	4.83187	11
1	−20	−8	30.2308	4.96349	13
−1	20	8	42.9762	5.19746	10
0	−20	−8	4.58846	2.37663	13
0	20	8	3.88445	2.25108	10
1	20	8	14.6015	3.31036	10
−1	20	−8	17.7791	1.86703	9
−1	20	−8	13.5345	3.30095	14
−2	20	−8	183.536	11.4185	9
−2	20	−8	182.046	11.1867	6
2	20	8	181.627	13.5923	10
3	20	8	19.1517	3.87340	10
−5	−21	8	1.27255	1.57242	13
5	−21	−8	0.71694	1.73346	13
5	21	−8	1.40701	2.09261	10
4	−21	−8	−0.5373	2.09981	13

-4	-21	8	-0.2404	2.27809	13
4	21	-8	1.25265	2.02932	10
3	-21	-8	3.16804	2.75522	13
-3	-21	8	3.55852	2.42024	13
3	21	-8	0.04069	1.89083	11
-3	21	8	0.98011	1.20001	11
-2	-21	8	15.9207	3.48444	13
2	-21	-8	13.4393	3.02760	13
2	21	-8	20.9543	2.79454	11
-2	21	8	9.60103	2.45555	10
-1	-21	8	9.36475	3.07881	13
-1	21	8	18.0418	3.41998	10
0	-21	-8	59.6450	5.75827	13
0	21	8	53.8857	6.10661	10
1	21	8	19.3405	3.83075	10
-1	21	-8	14.9261	2.01531	9
-1	21	-8	16.0238	1.42925	6
-2	21	-8	4.74475	0.91898	9
-2	21	-8	5.93226	0.61356	6
2	21	8	8.11388	3.24345	10
3	21	8	4.69625	2.11229	10
5	-22	-8	3.86801	2.00652	13
5	22	-8	9.51478	3.59646	10
-4	-22	8	11.9847	2.38924	13
4	-22	-8	9.81839	2.71915	13
4	22	-8	11.2457	2.09640	11
4	22	-8	9.90166	1.95195	10
-3	-22	8	5.96218	2.32007	13
3	-22	-8	2.90033	2.22030	13
3	22	-8	3.19665	1.78434	11
-3	22	8	3.41755	0.81806	11
-3	22	8	4.37695	0.86596	11
2	-22	-8	10.4046	2.60612	13
-2	-22	8	10.8647	2.93336	13
-2	22	8	9.91324	1.67579	11
2	22	-8	10.6797	1.15850	11
2	22	-8	9.08963	1.11039	11
-1	-22	8	5.10440	2.16026	13
1	-22	-8	14.1954	2.70246	13
-1	22	8	9.74563	2.56049	10
0	-22	-8	9.55661	2.42823	13
0	22	8	4.94224	2.63739	10
-1	22	-8	0.25599	0.88485	9
1	22	8	0.91603	2.50234	10
2	22	8	0.31334	2.46612	10
-2	22	-8	-0.1379	0.55804	9
4	-23	-8	12.9244	2.25634	13
-4	-23	8	13.3428	2.11971	13
4	23	-8	12.0724	1.67134	11
-3	-23	8	34.3810	4.22263	13

3	−23	−8	41.9837	4.27336	13
2	−23	−8	3.15533	2.04254	13
−2	−23	8	1.28493	1.92647	13
−2	23	8	1.11695	1.04635	11
1	−23	−8	27.1909	3.88506	13
−1	−23	8	32.9617	3.75778	13
−1	23	8	27.0359	3.39452	10
0	−23	−8	4.38879	2.33002	13
0	23	8	3.93274	2.10824	10
1	23	8	21.3124	3.48739	10
−3	−24	8	9.33821	2.21275	13
3	−24	−8	9.13642	2.37914	13
−2	−24	8	18.3377	3.51894	13
2	−24	−8	24.1081	3.35854	13
−1	−24	8	8.45458	1.89847	13
1	−24	−8	8.09900	2.37874	13
−1	24	8	7.29865	1.40040	11
0	−24	−8	1.34516	1.56385	13
9	0	−9	36.2821	5.78374	13
9	0	−9	39.6272	5.52289	13
−9	0	9	34.8986	4.03252	10
8	0	−9	24.1595	4.50380	13
−8	0	9	20.0107	4.01256	10
−7	0	9	6.53032	2.79795	10
7	0	−9	6.56104	3.21382	13
6	0	−9	−5.5969	3.25118	13
−6	0	9	1.37475	2.53104	10
−5	0	9	13.6492	3.41498	10
2	0	−9	68.9198	7.35344	5
1	0	−9	30.2043	4.67136	5
0	0	−9	0.73113	1.57212	5
−1	0	−9	44.1752	4.16491	8
−1	0	−9	42.6031	5.61544	1
−2	0	−9	3.29201	1.81944	8
−2	0	−9	2.80638	2.10389	1
4	0	9	222.127	16.4114	13
−4	0	−9	235.261	15.7275	12
−4	0	−9	234.751	15.0232	8
6	0	9	4.55209	3.18340	13
−6	0	−9	6.16995	1.35925	8
7	0	9	7.24412	2.10566	13
9	−1	−9	7.12649	3.38976	13
−9	−1	9	12.6285	2.76106	10
−9	1	9	8.61440	2.14789	10
9	1	−9	11.5737	3.53159	13
−9	1	9	10.4796	1.55894	11
9	1	−9	9.27501	3.27821	13
8	−1	−9	34.6815	6.35762	13
−8	1	9	40.1415	4.69306	10
−7	−1	9	60.0751	6.54252	10

7	−1	−9	72.3409	8.22341	13
−7	1	9	71.7791	6.64875	10
7	1	−9	62.1970	7.08575	13
−6	−1	9	27.6353	4.69720	10
6	−1	−9	22.8568	4.44639	13
−6	1	9	29.0239	4.61502	10
6	1	−9	31.6247	5.32476	13
−5	−1	9	32.5491	6.57509	1
−5	−1	9	25.9217	4.69720	10
−5	1	9	29.4280	4.60520	10
−4	−1	9	84.6850	9.52989	1
−3	−1	9	58.2547	7.78212	1
1	−1	−9	237.410	15.8535	5
1	1	−9	182.120	14.7958	5
0	1	−9	14.2348	2.11725	5
−1	−1	−9	43.9506	5.78859	1
−1	−1	−9	32.7243	3.83904	8
−1	1	−9	37.2508	5.02620	1
−2	−1	−9	4.36922	2.57790	1
−2	−1	−9	3.12793	1.57058	8
−2	1	−9	5.87413	1.64941	8
3	−1	9	16.9594	3.72462	13
3	1	9	25.7311	4.83316	13
−3	1	−9	14.0000	2.85951	10
−3	1	−9	16.1649	2.45236	8
−4	−1	−9	7.63381	2.22864	10
4	−1	9	17.9096	5.38638	13
−4	−1	−9	5.98424	2.16285	8
−4	−1	−9	8.87920	2.87840	12
4	1	9	6.32142	3.14551	13
−4	1	−9	7.15119	2.81646	12
−4	1	−9	8.27431	1.80690	8
5	−1	9	6.48467	3.58168	13
−5	−1	−9	6.24378	2.12629	8
−5	−1	−9	4.17636	2.40390	12
5	1	9	4.20394	3.15209	13
−5	1	−9	9.59447	1.89978	8
−5	1	−9	9.50750	1.29910	11
−6	−1	−9	6.86084	1.56691	8
6	−1	9	4.61356	3.02140	13
6	1	9	6.59331	2.77275	13
−6	1	−9	5.52104	1.29179	8
7	−1	9	18.1687	2.85656	13
7	1	9	17.5829	3.36060	13
−9	−2	9	0.24251	1.92395	10
9	−2	−9	−2.6243	3.02055	13
−9	2	9	−0.2942	0.97557	11
9	2	−9	4.04277	3.06075	13
8	−2	−9	46.7109	6.77812	13
−8	−2	9	52.7055	5.85467	10

8	2	−9	54.3320	7.24538	13
−8	2	9	53.5729	5.28352	10
−7	−2	9	257.133	17.7033	10
7	−2	−9	249.295	17.8592	13
7	2	−9	258.015	17.6077	13
−7	2	9	244.528	17.3270	10
6	−2	−9	71.5768	7.96962	13
−6	−2	9	93.0049	7.91460	10
−6	2	9	71.9728	7.30214	10
6	2	−9	97.4446	7.91142	13
−5	−2	9	38.2235	5.42200	10
−5	−2	9	43.6533	8.25203	1
−5	2	9	55.5754	5.72798	10
−4	−2	9	46.3554	7.42602	1
−3	−2	9	153.882	13.6403	1
2	2	−9	174.587	13.5916	5
1	−2	−9	68.1184	6.97648	5
1	2	−9	59.7867	6.02383	5
0	−2	−9	173.544	13.0996	5
−1	−2	−9	151.439	11.8341	1
−1	−2	−9	123.720	9.95478	8
−1	2	−9	144.002	9.53631	8
−1	2	−9	147.437	11.0365	1
−2	−2	−9	32.4220	3.51804	8
−2	−2	−9	35.1212	5.34628	1
−2	2	−9	34.1529	3.33548	8
−3	−2	−9	8.14884	2.04293	8
−3	2	−9	8.32327	2.12270	8
−4	−2	−9	15.4463	2.70646	8
−4	−2	−9	15.3716	2.93134	10
−4	2	−9	21.1285	2.88360	8
4	2	9	14.2800	3.68244	13
−4	2	−9	20.1702	3.07284	12
−5	−2	−9	63.9251	5.14207	8
5	2	9	52.9620	6.83304	13
−5	2	−9	65.3134	4.84149	8
−5	2	−9	67.0965	5.86951	12
−6	−2	−9	12.1660	1.94815	8
6	2	9	9.78480	3.56858	13
7	2	9	51.0044	5.34460	13
9	−3	−9	6.00415	3.05778	13
−9	3	9	3.77343	1.85270	7
−9	3	9	5.84085	1.21590	11
9	3	−9	10.4372	3.83369	13
−8	−3	9	0.08521	2.57897	10
8	−3	−9	2.49052	3.15402	13
−8	3	9	0.95579	1.95103	10
7	−3	−9	54.3065	6.59308	13
−7	−3	9	49.0962	6.09018	10
−7	3	9	53.6093	5.66115	10

7	3	−9	55.1688	7.37822	13
−6	−3	9	122.047	12.1459	1
−6	−3	9	108.316	10.0394	10
6	−3	−9	128.866	9.50454	13
−6	3	9	131.366	9.83605	10
6	3	−9	99.0383	9.92233	13
−5	−3	9	5.37267	2.89951	10
−5	−3	9	5.37237	4.19516	1
−4	−3	9	39.9787	7.09747	1
−3	3	9	234.673	18.4715	5
2	3	−9	89.6293	8.03133	5
1	−3	−9	40.9749	5.77676	5
1	3	−9	52.9684	4.82660	5
0	−3	−9	109.616	9.67175	5
−1	−3	−9	17.8560	4.56107	1
−1	−3	−9	18.9742	2.88998	8
−1	3	−9	23.8458	3.79352	1
−1	3	−9	25.6263	2.99890	8
−2	−3	−9	47.5572	5.98756	1
−2	−3	−9	45.0727	4.23134	8
−2	−3	−9	55.8530	5.87637	10
−2	3	−9	38.4695	4.02283	8
−3	−3	−9	0.01375	2.02106	10
−3	−3	−9	2.90817	2.66430	1
−3	−3	−9	2.33631	1.62235	8
−3	3	−9	6.28074	2.90514	14
3	3	9	1.72785	3.63719	13
−3	3	−9	3.92928	1.65602	8
−3	3	−9	0.90026	2.27995	12
−4	−3	−9	94.6585	7.13515	8
−4	−3	−9	93.9616	7.69134	10
4	3	9	97.8192	8.78521	13
−4	3	−9	99.0044	6.87424	8
−4	3	−9	96.3151	7.78601	12
−4	3	−9	97.8334	6.43179	11
−5	−3	−9	2.74557	2.07856	8
5	3	9	3.85545	3.03418	13
−5	3	−9	3.41721	2.25796	12
−5	3	−9	5.41262	1.37302	8
−6	−3	−9	−0.7786	1.52115	8
−6	3	−9	2.41571	1.84333	12
6	3	9	−3.8013	3.14534	13
7	3	9	27.9985	4.45494	13
9	−4	−9	−1.8987	2.79830	13
−9	−4	9	2.14508	2.05781	10
−9	4	9	1.72876	1.58715	7
9	4	−9	1.40255	2.75034	13
−9	4	9	1.86973	0.81890	11
−8	−4	9	29.3953	4.75419	10
8	−4	−9	35.7526	5.79990	13

8	4	−9	30.3674	4.52984	13
8	4	−9	29.0941	4.66404	13
−8	4	9	31.9232	4.14111	10
−7	−4	9	85.9444	7.66076	10
−7	−4	9	88.5253	10.4346	1
7	−4	−9	53.4709	7.20628	13
7	4	−9	85.5607	8.87699	13
−6	−4	9	7.84243	4.55522	1
−6	−4	9	8.18860	3.09912	10
6	4	−9	10.3824	3.63916	13
−5	−4	9	4.66821	4.18641	1
−5	−4	9	8.58692	3.04287	10
−4	−4	9	22.6234	5.51610	1
−3	4	9	11.6278	3.73403	5
2	4	−9	6.24112	2.49345	5
1	4	−9	386.978	24.5089	5
0	−4	−9	375.077	25.8276	5
−1	−4	−9	162.980	10.5565	8
−1	−4	−9	169.579	13.2048	1
−1	4	−9	130.213	10.4180	4
−1	4	−9	144.703	10.1356	8
−2	−4	−9	3.81015	2.30365	10
−2	−4	−9	2.88831	2.73512	1
−2	4	−9	4.43385	1.59508	8
−3	−4	−9	85.2603	6.60602	8
−3	−4	−9	83.1317	7.03119	10
−3	−4	−9	84.6363	8.17525	1
−3	4	−9	79.2590	6.96685	12
−3	4	−9	82.2473	7.33908	14
−3	4	−9	79.7582	6.01664	8
3	4	9	79.2978	7.93509	13
−4	−4	−9	54.2072	5.02964	8
−4	−4	−9	54.4811	5.45402	10
−4	4	−9	59.9545	3.78832	11
−4	4	−9	61.8742	6.26705	12
4	4	9	54.3758	7.60674	13
−4	4	−9	62.1179	4.56527	8
−5	−4	−9	38.7511	4.04121	10
−5	−4	−9	36.1802	3.83887	8
−5	4	−9	31.4593	2.96397	8
−5	4	−9	33.8938	4.39289	12
5	4	9	38.8896	5.97210	13
−6	−4	−9	43.4413	3.83021	8
−6	−4	−9	46.8251	3.38921	11
−6	4	−9	43.4448	4.80610	12
6	4	9	43.9102	5.68308	13
7	4	9	48.4286	4.74849	13
9	−5	−9	12.5344	3.17405	13
−9	5	9	12.2991	2.12181	7
−9	5	9	13.4113	1.28300	11

8	−5	−9	32.1529	5.72664	13
−8	−5	9	46.9916	5.29131	10
−8	5	9	34.7537	3.23189	11
−8	5	9	32.1490	3.96609	10
8	5	−9	40.9546	6.16682	13
7	−5	−9	63.0197	8.26110	13
−7	−5	9	94.6969	10.3915	1
−7	−5	9	95.1499	8.15559	10
7	5	−9	87.0445	8.10920	13
7	5	−9	88.1649	7.87038	13
6	−5	−9	27.4960	4.41654	13
−6	−5	9	34.8564	7.35991	1
−6	−5	9	39.1234	5.15843	10
6	5	−9	37.1411	5.11995	13
−6	5	9	24.1641	4.69928	10
−5	−5	9	79.8563	9.95992	1
−5	−5	9	69.4015	7.15104	10
−5	5	9	74.9551	7.51213	10
−4	−5	9	42.6353	7.37672	1
0	−5	−9	41.8018	5.61102	5
−1	−5	−9	237.256	19.0446	1
−1	5	−9	251.830	16.5958	4
−1	5	−9	265.633	16.2065	8
−2	−5	−9	13.7235	2.54273	8
−2	−5	−9	11.4043	3.02195	10
−2	−5	−9	14.0849	3.94532	1
−2	5	−9	14.8835	2.22356	8
−3	−5	−9	1.96550	2.15769	10
−3	−5	−9	2.96030	3.10968	1
−3	−5	−9	3.13331	1.84424	8
3	5	9	1.67189	3.67662	13
−3	5	−9	0.18109	1.36300	8
−3	5	−9	−0.8587	2.08572	12
−3	5	−9	−0.2247	2.06769	14
−4	−5	−9	26.2745	4.45606	10
−4	−5	−9	27.9467	3.64836	8
−4	5	−9	30.3589	4.37039	12
4	5	9	24.9064	4.39750	13
−4	5	−9	31.9107	2.88642	8
−5	−5	−9	30.1451	3.98834	10
−5	−5	−9	27.9042	3.61914	8
5	5	9	26.6099	4.50967	13
−5	5	−9	31.4561	4.31134	12
−6	−5	−9	35.7061	3.23069	8
−6	5	−9	29.2610	2.54390	9
−6	5	−9	29.9224	3.79703	12
−6	5	−9	29.5580	2.16835	6
6	5	9	33.2016	5.44350	13
7	5	9	6.30480	2.06075	13
−9	−6	9	9.47810	2.56366	10

−9	6	9	8.51963	1.86544	7
8	−6	−9	25.8344	4.54369	13
−8	−6	9	26.0223	3.81540	10
−8	6	9	26.2141	3.30077	7
8	6	−9	26.0609	4.28550	13
−8	6	9	20.4987	2.49836	11
−7	−6	9	159.242	11.9996	10
−7	−6	9	162.163	14.3160	1
7	−6	−9	147.997	12.0261	13
−7	6	9	139.768	10.9364	10
7	6	−9	162.663	12.2693	13
7	6	−9	149.275	12.5027	13
6	−6	−9	66.6212	6.70048	13
−6	6	9	67.7347	6.37070	10
5	−6	−9	13.5887	3.36949	13
−5	−6	9	16.0473	5.31542	1
−5	6	9	15.2531	3.80340	10
−2	6	9	31.1314	6.36641	5
0	6	−9	68.5650	5.22506	8
−1	−6	−9	151.297	13.8553	1
−1	6	−9	180.405	11.1682	8
−2	−6	−9	29.5728	5.75503	1
−2	−6	−9	29.2051	3.55613	8
−2	−6	−9	26.6356	4.40274	10
−2	6	−9	33.2244	4.22334	12
−2	6	−9	29.2987	3.12437	8
−3	−6	−9	91.5949	6.48526	8
−3	−6	−9	94.0592	9.20325	1
−3	−6	−9	86.1903	7.13891	10
−3	6	−9	77.8929	5.33484	11
−3	6	−9	73.8627	5.24110	11
−3	6	−9	75.9383	6.86140	12
−3	6	−9	72.5667	6.70029	14
3	6	9	87.2674	8.44546	13
−3	6	−9	78.3031	5.77118	8
−4	−6	−9	149.281	10.1133	10
−4	−6	−9	138.488	9.54794	8
4	6	9	132.792	10.9015	13
−4	6	−9	127.644	9.94760	12
−4	6	−9	122.254	8.74140	8
−5	−6	−9	3.83781	2.14782	10
−5	−6	−9	4.35508	2.02421	8
−5	6	−9	6.65433	2.45926	12
5	6	9	−0.9840	3.46269	13
−5	6	−9	5.92280	1.22472	9
−6	−6	−9	2.40108	1.56509	8
−6	6	−9	5.56538	0.70410	6
−6	6	−9	6.11534	1.20050	9
−6	6	−9	4.14862	2.12127	12
6	6	9	4.59629	3.02886	13

9	−7	−9	22.3243	4.16646	13
−9	−7	9	17.7410	3.35300	10
−8	−7	9	48.7275	5.56212	10
8	−7	−9	48.6772	6.19706	13
−8	7	9	42.9328	3.56578	11
−8	7	9	41.2509	4.21353	7
−7	−7	9	37.3092	5.67736	10
7	−7	−9	39.3521	5.38493	13
−7	7	9	38.1167	4.39250	10
−6	−7	9	235.456	17.5466	10
−6	−7	9	249.210	20.4429	1
6	−7	−9	250.770	17.0912	13
−6	7	9	253.851	17.0518	10
5	−7	−9	31.1995	4.81500	13
−5	−7	9	29.1446	6.36010	1
−5	7	9	23.7250	4.51138	10
5	7	−9	24.8557	4.72007	10
4	7	−9	41.0118	6.31817	10
−4	7	9	41.6821	5.32833	10
0	7	−9	60.5659	5.15073	4
−1	−7	−9	118.814	11.5305	1
−1	7	−9	122.964	8.33663	8
−2	−7	−9	11.2259	2.93033	10
−2	−7	−9	16.1401	4.91642	1
−2	7	−9	12.1614	1.54912	11
−2	7	−9	11.3891	3.07986	12
−2	7	−9	13.2432	3.60086	14
−2	7	−9	12.5867	1.81827	8
2	7	9	12.1688	3.87329	13
−3	−7	−9	4.72358	3.79088	1
−3	−7	−9	2.08030	2.34739	10
−3	−7	−9	3.68190	2.01180	8
−3	7	−9	1.85134	2.30347	12
3	7	9	3.71760	3.07832	13
−3	7	−9	2.23783	2.18362	14
−3	7	−9	2.72405	1.26400	8
−4	−7	−9	58.8496	5.69353	10
−4	−7	−9	55.2172	5.20193	8
4	7	9	59.6476	7.77566	13
−4	7	−9	57.2077	5.63630	12
−4	7	−9	55.0423	4.29404	9
−5	−7	−9	21.4235	3.87280	10
−5	−7	−9	19.6656	3.34555	8
−5	7	−9	21.0686	3.32620	12
−5	7	−9	25.4705	1.89718	6
5	7	9	20.6095	4.79554	13
−5	7	−9	25.8437	2.21499	9
−6	−7	−9	0.66546	1.47246	8
6	7	9	0.31371	2.76342	13
−6	7	−9	0.98354	1.81711	12

−6	7	−9	2.02956	0.51367	6
−6	7	−9	2.20563	0.83974	9
9	−8	−9	14.8385	2.86334	13
−9	−8	9	16.9645	2.60927	10
8	−8	−9	1.14011	3.81814	13
−8	8	9	11.9944	1.63036	11
−8	8	9	6.74949	1.97480	7
−7	−8	9	89.2591	8.14604	10
7	−8	−9	74.6398	7.73324	13
−6	−8	9	88.0546	10.4134	1
−6	−8	9	74.2162	7.54558	10
6	−8	−9	69.1148	7.33034	13
−6	8	9	66.6009	6.62993	10
−5	−8	9	29.3662	6.79814	1
5	−8	−9	36.4703	5.04904	13
−5	8	9	29.2817	4.71433	10
5	8	−9	33.6304	5.45895	10
4	−8	−9	0.42345	3.73345	2
3	−8	−9	31.8406	6.95440	2
0	−8	−9	35.5469	6.88013	1
−1	−8	−9	69.6459	6.73987	10
−1	−8	−9	66.9345	8.59215	1
−2	−8	−9	76.7182	6.94904	10
−2	−8	−9	85.4058	9.62393	1
−2	8	−9	64.3337	6.34453	12
−2	8	−9	68.9157	6.51762	14
2	8	9	74.1308	7.97345	13
−2	8	−9	62.2172	4.79536	11
−3	−8	−9	208.788	16.2313	1
−3	−8	−9	202.513	14.0806	10
−3	−8	−9	203.785	13.4579	8
−3	8	−9	184.666	13.6423	12
3	8	9	209.391	14.9328	13
−3	8	−9	180.620	13.8243	14
−4	−8	−9	31.4937	4.62668	10
−4	−8	−9	34.0492	5.54173	1
−4	−8	−9	27.6912	4.14107	8
−4	8	−9	30.1809	2.92059	9
−4	8	−9	33.0454	2.41723	6
4	8	9	31.2297	4.90820	13
−4	8	−9	44.1026	4.84120	12
−5	−8	−9	31.3707	3.55584	8
−5	−8	−9	26.6087	3.57495	10
−5	8	−9	17.7679	1.69045	6
−5	8	−9	23.8951	3.29839	12
5	8	9	23.6842	4.25473	13
−5	8	−9	21.9225	2.22444	9
−6	−8	−9	50.3478	4.50919	8
−6	8	−9	60.9811	4.98777	12
6	8	9	57.5193	5.85958	13

−6	8	−9	54.1124	3.64936	9
9	−9	−9	12.7852	2.50116	13
−9	−9	9	6.27971	1.84863	10
8	−9	−9	2.86408	2.76156	13
−8	−9	9	3.53693	2.13268	10
−8	9	9	3.49524	0.75247	11
−8	9	9	3.35453	0.74603	11
7	−9	−9	105.521	9.01763	13
−7	−9	9	114.485	9.48545	10
−7	9	9	108.532	7.39397	11
6	−9	−9	40.3991	5.39467	13
−6	9	9	37.5129	4.41944	10
5	−9	−9	14.9568	3.15881	13
−5	9	9	13.8998	3.15952	10
−4	−9	9	100.006	9.00845	13
4	−9	−9	92.7918	8.74618	13
4	−9	−9	92.1294	10.5489	2
4	9	−9	95.9614	8.46694	10
−4	9	9	96.3741	8.28503	10
3	−9	−9	71.4472	8.31579	2
−3	9	9	68.1726	6.64312	10
3	9	−9	58.9351	6.41091	10
2	−9	−9	3.86585	3.27141	2
0	−9	−9	20.9236	5.19287	1
−1	−9	−9	21.1429	3.85047	10
−1	−9	−9	26.6123	5.98098	1
−1	9	−9	19.1156	2.56446	11
−2	−9	−9	81.0033	9.20299	1
−2	−9	−9	73.4681	6.83888	10
2	9	9	74.0952	7.86159	13
−2	9	−9	67.2343	6.78390	14
−2	9	−9	60.9874	4.46619	11
−2	9	−9	61.2730	6.05997	12
−2	9	−9	65.4574	4.62950	11
−3	−9	−9	50.2126	5.68594	10
−3	−9	−9	50.0849	8.07227	1
−3	9	−9	50.7887	5.54431	14
−3	9	−9	49.4759	3.83638	9
−3	9	−9	47.6615	5.21729	12
3	9	9	61.3215	7.71008	13
−4	−9	−9	100.188	8.50872	10
−4	−9	−9	105.998	10.7900	1
−4	−9	−9	100.129	7.93830	8
−4	9	−9	101.158	6.78958	9
−4	9	−9	103.401	6.61522	6
−4	9	−9	113.398	8.42019	12
4	9	9	97.5125	9.57016	13
−4	9	−9	121.189	9.75125	14
−5	−9	−9	3.82388	1.62607	8
−5	−9	−9	4.26962	2.29689	10

−5	9	−9	2.91919	1.05012	9
−5	9	−9	5.18559	0.68250	6
−5	9	−9	2.99192	2.52437	12
−6	−9	−9	2.31749	1.58212	8
−6	−9	−9	2.78745	1.49447	10
−6	9	−9	1.95251	1.87237	12
−6	9	−9	0.84048	0.65847	9
−6	9	−9	2.30424	0.58422	9
6	9	9	2.73017	2.37876	13
−9	−10	9	3.55329	2.00116	10
9	−10	−9	4.19197	2.44501	13
−8	−10	9	38.4516	4.95545	10
8	−10	−9	33.0758	4.61272	13
7	−10	−9	53.3566	6.28793	13
−7	−10	9	42.2198	6.07782	10
−7	10	9	57.7266	4.03183	11
6	−10	−9	3.99287	2.78619	13
−6	10	9	6.90789	2.13798	10
5	−10	−9	29.1210	4.96598	13
5	10	−9	28.2526	5.43557	10
−5	10	9	38.9345	4.81564	10
4	−10	−9	45.2187	6.02543	13
4	−10	−9	53.4414	9.40407	2
−4	−10	9	62.7782	6.69879	13
−4	10	9	51.6934	6.04081	10
4	10	−9	57.9608	6.36915	10
3	−10	−9	97.5705	10.2026	2
3	10	−9	92.1285	8.00202	10
−3	10	9	90.9519	8.18465	10
2	−10	−9	69.6177	8.32580	2
2	10	−9	64.8012	6.55014	10
−2	10	9	65.5337	7.02213	10
1	−10	−9	4.53741	3.40296	2
0	−10	−9	290.651	22.1108	1
0	10	−9	287.413	18.9518	10
0	10	9	273.529	20.0540	10
−1	−10	−9	111.182	12.0807	1
−1	−10	−9	107.275	9.63585	10
−1	10	−9	128.138	7.95031	11
−1	10	−9	125.036	8.94345	12
−1	10	−9	125.119	9.53443	14
−2	−10	−9	2.31208	2.62105	10
−2	−10	−9	4.08510	3.81918	1
−2	10	−9	8.72330	2.89641	12
2	10	9	5.99335	3.65903	13
−2	10	−9	5.06385	2.41773	14
−3	−10	−9	40.0450	5.51479	10
−3	−10	−9	42.2041	8.08323	1
−3	10	−9	53.3845	5.23842	12
−3	10	−9	50.9341	3.36697	6

-3	10	-9	51.8382	3.57477	9
3	10	9	39.4122	6.09628	13
-3	10	-9	53.3965	5.69443	14
-4	-10	-9	21.9353	3.24389	8
-4	-10	-9	23.5432	4.21186	10
4	10	9	24.4410	4.96392	13
-4	10	-9	24.8034	2.33715	9
-4	10	-9	22.1204	1.96515	6
-4	10	-9	34.1724	4.85862	14
-4	10	-9	37.0202	4.62314	12
-5	-10	-9	11.8786	2.38330	8
-5	-10	-9	13.3343	2.66401	10
-5	10	-9	10.3692	1.21691	9
-5	10	-9	8.77310	2.68695	12
-5	10	-9	11.0119	0.97958	6
5	10	9	10.1165	3.99582	13
-5	10	-9	10.1233	0.91695	6
-6	-10	-9	6.55541	1.70814	10
-6	10	-9	5.71675	1.97514	12
6	10	9	9.80650	2.52855	13
-8	-11	9	12.4436	2.67527	10
8	-11	-9	9.56143	2.66647	13
7	-11	-9	8.31289	2.85377	13
-7	11	9	8.12887	1.29286	11
-6	11	9	27.9273	3.52996	10
5	-11	-9	1.52647	2.51854	13
-5	-11	9	-0.7220	2.16725	13
-5	11	9	1.81859	2.21841	10
5	11	-9	1.59022	2.92297	10
-4	-11	9	7.91348	2.82345	13
4	-11	-9	14.3857	3.50635	13
-4	11	9	4.65184	2.70477	10
4	11	-9	7.97351	3.10265	10
3	-11	-9	9.40446	4.40282	2
3	-11	-9	9.58427	2.79266	13
-3	-11	9	10.9181	3.68181	13
-3	11	9	14.2584	3.73610	10
3	11	-9	14.2292	3.22917	10
2	-11	-9	12.9562	4.72739	2
2	11	-9	16.0355	3.04385	10
-2	11	9	12.8333	3.60341	10
1	-11	-9	49.5919	7.41246	2
1	11	-9	49.2625	5.26099	10
-1	11	9	59.4569	6.76217	10
0	-11	-9	105.799	11.0327	1
0	11	9	95.5973	9.12176	10
0	11	-9	86.7034	6.56583	11
0	11	-9	85.9507	7.39519	14
-1	-11	-9	242.831	18.8008	1
-1	-11	-9	234.668	15.6150	10

−1	11	−9	192.962	14.6853	14
−1	11	−9	204.001	13.5507	11
−2	−11	−9	56.8622	6.32420	10
−2	−11	−9	60.8480	8.47304	1
−3	−11	−9	29.5347	6.88735	1
−3	11	−9	47.4436	3.33849	9
3	11	9	30.3331	5.25905	13
−3	11	−9	43.5795	5.38171	14
−3	11	−9	45.9333	2.95109	6
−3	11	−9	50.2456	5.05631	12
−4	−11	−9	13.0124	3.28185	10
−4	11	−9	11.6231	1.17911	6
−4	11	−9	7.72918	3.06623	14
4	11	9	9.99556	3.49583	13
−4	11	−9	6.90349	2.88029	12
−4	11	−9	9.25680	1.38630	9
−5	−11	−9	14.6237	2.52524	8
−5	−11	−9	13.5797	2.57678	10
−5	11	−9	12.4361	2.74510	12
−5	11	−9	13.0596	1.01386	6
5	11	9	14.0151	3.70267	13
−5	11	−9	13.9026	1.51385	9
−5	11	−9	13.8012	1.04365	6
−6	−11	−9	20.8279	3.05114	10
6	11	9	25.1371	4.03339	13
−6	11	−9	30.3138	3.29656	12
8	−12	−9	13.1517	2.71852	13
−8	−12	9	16.9142	3.23062	10
7	−12	−9	14.3391	3.21307	13
−7	12	9	19.8298	1.78083	11
−6	12	9	18.8963	2.54093	11
6	12	−9	23.9329	4.51440	10
−5	−12	9	78.0322	6.97786	13
5	−12	−9	78.0947	7.92098	13
−5	12	9	73.7832	6.79035	10
5	12	−9	80.9456	7.80749	10
4	−12	−9	17.9272	3.41541	13
−4	−12	9	16.4025	3.88372	13
−4	12	9	17.5021	3.89120	10
4	12	−9	18.3064	4.18677	10
3	−12	−9	127.393	10.4007	13
−3	−12	9	123.143	10.5544	13
3	12	−9	142.036	10.4294	10
−3	12	9	140.430	11.0473	10
−2	−12	9	8.22660	3.12851	13
2	−12	−9	13.9258	3.55023	13
2	−12	−9	12.9458	4.93576	2
−2	12	9	19.3306	4.62631	10
1	−12	−9	19.2303	3.75500	13
1	12	−9	28.8456	4.44964	14

0	−12	−9	18.4815	3.53978	13
0	12	−9	15.0943	3.02725	14
0	12	−9	17.4046	2.35457	11
0	12	9	26.1862	4.89253	10
−1	−12	−9	188.345	15.6132	1
−1	12	−9	155.210	10.0294	11
−1	12	−9	150.200	11.3346	14
−1	12	−9	149.190	9.90380	11
−2	−12	−9	215.035	17.6323	1
−2	−12	−9	208.576	14.4135	10
−2	12	−9	173.941	13.3376	12
−2	12	−9	193.478	13.7108	14
−3	−12	−9	89.6820	10.2022	1
−3	−12	−9	78.3045	8.15649	10
−3	12	−9	95.2763	6.21302	9
−3	12	−9	98.5998	8.15469	14
−3	12	−9	97.2889	5.90919	6
−4	−12	−9	1.87823	2.53759	10
−4	12	−9	2.02294	0.99055	9
−4	12	−9	2.55770	2.49635	12
−4	12	−9	3.04732	3.10089	14
−4	12	−9	3.00219	0.58383	6
4	12	9	2.61373	2.89786	13
−5	−12	−9	89.8113	6.98696	10
−5	12	−9	86.5688	7.00588	12
5	12	9	84.6816	7.23424	13
−6	12	−9	62.5947	5.02302	12
−8	−13	9	2.44918	2.25380	10
8	−13	−9	7.31285	2.14624	13
7	−13	−9	8.79229	2.70634	13
7	13	−9	12.7146	3.81315	10
6	−13	−9	32.7604	6.26949	13
6	13	−9	34.3124	5.85489	10
−6	13	9	33.2295	2.94216	11
−5	−13	9	151.146	11.2322	13
5	−13	−9	156.837	11.5663	13
5	13	−9	153.461	11.6758	10
−5	13	9	153.679	11.0735	10
4	−13	−9	9.13361	3.40168	13
−4	−13	9	5.32094	2.79929	13
−4	13	9	7.76050	2.70626	10
−3	−13	9	15.7061	3.33855	13
3	−13	−9	16.3938	3.24053	13
3	13	−9	19.4027	3.49896	10
−3	13	9	17.2742	3.62455	10
−2	−13	9	18.3811	3.87054	13
2	−13	−9	9.38731	3.17651	13
−2	13	9	9.68358	3.42069	10
−1	−13	9	8.69343	3.44246	13
1	−13	−9	11.1567	3.28347	13

−1	13	9	11.6684	3.86451	10
1	13	−9	16.7548	3.04517	14
1	13	−9	12.1759	2.76868	10
0	−13	−9	11.8502	3.06518	13
0	13	9	11.2632	4.18805	10
0	13	−9	20.7544	2.82307	11
−1	−13	−9	1.93019	3.92425	1
1	13	9	4.20287	3.59340	10
−2	−13	−9	147.328	11.0594	10
−2	−13	−9	151.882	14.1697	1
−2	13	−9	125.727	9.92836	12
−2	13	−9	134.563	8.94858	9
−2	13	−9	130.895	10.6728	14
−3	−13	−9	25.9341	4.17752	10
−3	13	−9	33.0696	5.21804	14
−3	13	−9	30.0062	2.17126	6
−3	13	−9	33.3922	2.83777	9
−3	13	−9	30.2826	4.52004	12
−4	−13	−9	21.9583	3.57341	10
−4	13	−9	24.6528	1.68529	6
−4	13	−9	22.0729	1.65585	6
−4	13	−9	22.6679	3.59782	12
−4	13	−9	24.4664	2.16603	9
−4	13	−9	26.5135	3.63688	14
−5	−13	−9	42.9806	4.44979	10
−5	13	−9	42.3248	4.55675	12
8	−14	−9	24.6947	3.97466	13
−8	−14	9	25.2757	3.79678	10
7	−14	−9	34.1354	4.62897	13
7	14	−9	43.6979	5.04549	10
6	−14	−9	1.77604	2.70092	13
−6	14	9	2.28526	1.09501	11
6	14	−9	3.65589	3.44920	10
−5	−14	9	28.2221	4.69023	13
−5	14	9	26.6170	3.62970	10
4	−14	−9	16.4041	3.29473	13
−4	−14	9	15.3492	3.25397	13
4	14	−9	17.0641	3.62344	10
−4	14	9	14.7455	3.10822	10
3	−14	−9	76.7839	7.01064	13
−3	−14	9	74.6232	7.18249	13
3	14	−9	82.0468	7.26478	10
−3	14	9	81.9262	7.45919	10
2	−14	−9	109.347	8.34348	13
−2	−14	9	98.3775	9.05288	13
2	14	−9	93.8560	8.30383	10
−2	14	9	112.265	9.30611	10
1	−14	−9	18.8637	3.34822	13
−1	−14	9	22.7622	4.27044	13
1	14	−9	24.8169	3.27830	10

1	14	−9	28.1003	4.47759	14
−1	14	9	24.5028	4.44595	10
0	−14	−9	193.889	13.0575	13
0	14	−9	176.883	13.1281	14
0	14	−9	190.162	12.0485	11
0	14	9	188.515	14.5001	10
−1	−14	−9	95.8028	11.9298	1
−1	14	−9	107.170	8.37920	14
1	14	9	75.8843	8.99831	10
−2	−14	−9	2.50530	3.09863	10
−2	−14	−9	0.72236	4.48993	1
−2	14	−9	5.30313	1.27821	9
−2	14	−9	8.95885	2.86372	14
−2	14	−9	5.11700	0.82457	6
−2	14	−9	4.55729	2.44872	12
−3	−14	−9	63.7205	7.16326	10
−3	14	−9	82.4782	7.19112	14
−3	14	−9	77.3619	6.57444	12
−4	−14	−9	8.87807	2.87103	10
−4	14	−9	4.79719	2.65775	14
−4	14	−9	9.26666	0.77615	6
−4	14	−9	5.64696	2.62341	12
−4	14	−9	7.12182	1.01657	9
−4	14	−9	7.25292	0.68298	6
−5	−14	−9	37.4861	4.09368	10
−5	14	−9	38.8802	3.64865	12
7	−15	−9	26.0620	3.94038	13
7	15	−9	36.4172	5.07722	10
−6	15	9	18.6247	1.92721	11
6	15	−9	19.6627	3.69509	10
−5	−15	9	38.7750	5.61905	13
5	−15	−9	29.6513	4.20527	13
5	15	−9	36.4141	5.51989	10
−5	15	9	23.8342	2.88974	11
4	−15	−9	52.9536	7.10271	13
−4	−15	9	65.3763	6.60660	13
−4	15	9	61.1503	6.32207	10
4	15	−9	64.9163	6.57748	10
3	−15	−9	74.5254	7.04555	13
−3	−15	9	82.2491	7.75160	13
3	15	−9	79.7381	7.51401	10
−3	15	9	78.1240	7.35670	10
2	−15	−9	28.4143	5.00597	13
−2	−15	9	15.4010	3.78708	13
2	15	−9	16.5332	3.04141	10
−2	15	9	28.5326	4.98664	10
−1	−15	9	21.8858	3.81462	13
1	−15	−9	13.9785	3.42067	13
−1	15	9	11.4522	3.78447	10
1	15	−9	20.5225	3.29689	14

1	15	-9	26.2010	3.69235	11
0	-15	-9	75.8733	6.74286	13
0	15	-9	73.9192	5.47875	11
0	15	-9	71.1813	5.23730	11
0	15	9	76.7389	8.12890	10
0	15	-9	76.1239	7.38895	14
-1	-15	-9	110.252	12.1414	1
1	15	9	102.340	9.49205	10
-1	15	-9	116.184	8.94794	14
-2	-15	-9	4.14311	3.35191	10
-2	15	-9	5.75012	1.29768	9
-2	15	-9	4.49447	2.98390	14
2	15	9	5.74344	4.02632	10
-2	15	-9	8.70712	0.93684	6
-3	-15	-9	5.93880	3.06648	10
-3	15	-9	3.37056	1.02155	9
-3	15	-9	4.05147	2.44463	12
-3	15	-9	4.01948	0.64812	6
-3	15	-9	4.71505	2.71354	14
-4	-15	-9	-0.3077	2.46515	10
-4	15	-9	2.27359	2.31393	12
-4	15	-9	3.20109	2.47328	14
-4	15	-9	0.88920	0.56320	9
-4	15	-9	1.92732	0.60865	9
-5	-15	-9	53.3473	4.74111	10
-5	15	-9	51.5415	4.68359	12
7	-16	-9	18.3843	3.19639	13
7	16	-9	17.3292	3.32921	10
6	-16	-9	5.64826	3.12917	13
6	16	-9	0.45356	3.13792	10
-5	-16	9	135.665	10.1998	13
5	-16	-9	148.153	10.8392	13
5	16	-9	124.626	10.1820	10
-5	16	9	126.392	8.64082	11
4	-16	-9	108.756	9.22352	13
-4	-16	9	105.794	9.02923	13
4	16	-9	114.419	9.30861	10
-4	16	9	107.983	8.74158	10
3	-16	-9	70.5574	6.52607	13
-3	-16	9	59.9630	6.51549	13
-3	16	9	69.7096	6.49805	10
3	16	-9	56.0799	6.19436	10
2	-16	-9	12.3409	3.30543	13
-2	-16	9	5.87517	3.18267	13
2	16	-9	4.04048	2.63696	10
-2	16	9	9.53138	3.36146	10
1	-16	-9	75.9324	7.07052	13
-1	-16	9	60.0775	6.49620	13
1	16	-9	69.7666	6.70042	14
1	16	-9	67.1891	5.37502	11

−1	16	9	81.0604	7.73117	10
0	−16	−9	91.2971	7.81006	13
0	16	−9	90.4108	8.05996	14
0	16	−9	100.086	6.24564	11
0	16	9	104.240	9.30393	10
0	16	−9	90.7493	6.13184	11
−1	16	−9	134.435	11.0972	14
1	16	9	160.059	11.9299	10
2	16	9	46.1424	6.96210	10
−2	16	−9	44.8501	3.06360	6
−3	−16	−9	0.87894	3.10905	10
−3	16	−9	3.72470	3.03355	14
−3	16	−9	0.85688	0.53123	6
−3	16	−9	1.77669	0.86699	9
−3	16	−9	0.57050	2.40258	12
−3	16	−9	1.35546	0.43788	6
−4	−16	−9	13.8749	2.75576	10
−4	16	−9	14.6548	2.58253	12
−4	16	−9	15.2221	2.70738	14
−5	16	−9	2.27903	1.75442	12
7	−17	−9	4.44579	2.06483	13
7	17	−9	0.12019	2.55209	10
6	−17	−9	2.32697	2.31841	13
6	17	−9	−0.3649	3.02899	10
−5	−17	9	56.9763	6.41417	13
5	−17	−9	84.8283	7.05543	13
5	17	−9	65.4342	7.28886	10
−5	17	9	81.0783	5.08910	11
4	−17	−9	16.5068	3.49890	13
−4	−17	9	15.3815	3.45406	13
4	17	−9	18.4313	3.94690	10
−4	17	9	14.9324	2.72862	10
−3	−17	9	7.83757	2.98611	13
3	−17	−9	1.71182	3.22520	13
3	17	−9	1.31830	2.60697	10
−3	17	9	0.83777	2.96771	10
−2	−17	9	46.4272	6.77416	13
2	17	−9	57.8944	5.70057	10
−2	17	9	50.0011	6.45194	10
−1	−17	9	31.2590	5.16781	13
1	−17	−9	37.7277	5.20319	13
−1	17	9	35.8465	6.02039	10
1	17	−9	35.3774	5.40207	14
1	17	−9	39.3414	3.82987	11
0	−17	−9	6.80034	3.23106	13
0	17	−9	5.31763	3.04843	14
0	17	9	1.30315	3.58415	10
1	17	9	50.0580	7.30307	10
−1	17	−9	71.6260	6.66923	14
−2	17	−9	6.51618	0.85247	6

−2	17	−9	5.38327	1.30801	9
−2	17	−9	8.34997	2.98854	14
2	17	9	8.37033	3.96587	10
−3	−17	−9	2.48000	1.95771	10
−3	17	−9	2.44300	2.11224	12
−3	17	−9	2.36248	0.40719	6
−3	17	−9	3.17380	0.88218	9
−3	17	−9	1.88638	2.64741	14
−3	17	−9	2.80008	0.43816	6
−4	−17	−9	−0.9470	2.01549	10
−4	17	−9	0.29179	1.77678	12
6	−18	−9	8.34743	2.57964	13
6	18	−9	6.21429	2.86669	10
5	−18	−9	1.54525	2.38468	13
−5	−18	9	0.33831	2.09888	13
−5	18	9	0.26906	0.60126	11
−5	18	9	−0.0408	0.59107	11
5	18	−9	0.06749	2.83283	10
−4	−18	9	16.9309	3.16477	13
4	18	−9	10.3688	3.14239	10
3	−18	−9	2.07972	2.83302	13
−3	−18	9	−0.0435	2.54908	13
−3	18	9	0.26251	2.18300	10
3	18	−9	−2.5665	2.39532	10
2	−18	−9	16.4766	3.12843	13
−2	−18	9	12.2953	3.01089	13
−2	18	9	10.7322	3.38618	10
2	18	−9	11.6286	2.47991	11
−1	−18	9	108.117	9.21521	13
1	−18	−9	113.168	8.78645	13
−1	18	9	110.788	9.49276	10
1	18	−9	105.019	7.53630	11
1	18	−9	111.525	8.81111	14
1	18	−9	113.383	7.41166	11
0	−18	−9	34.3580	4.96158	13
0	18	9	36.7461	5.60163	10
0	18	−9	38.4488	5.50312	14
1	18	9	9.62647	3.87066	10
−1	18	−9	11.8233	3.23520	14
−2	18	−9	77.5508	6.44865	14
2	18	9	73.8983	7.90978	10
−2	18	−9	77.0427	5.22749	9
−2	18	−9	75.5124	4.85628	6
−3	−18	−9	18.2252	2.94380	10
−3	18	−9	12.4665	1.28742	9
−3	18	−9	13.7372	2.82052	14
3	18	9	13.8667	3.05789	10
−3	18	−9	11.2741	2.70717	12
−3	18	−9	11.5672	1.33749	9
−4	18	−9	31.6736	3.76011	12

6	−19	−9	10.1067	2.09092	13
6	19	−9	13.6612	2.92293	10
−5	−19	9	24.4953	3.59919	13
5	19	−9	20.7068	4.00249	10
4	−19	−9	8.21522	3.04622	13
−4	19	9	11.0872	1.39954	11
3	−19	−9	12.9801	3.18774	13
−3	−19	9	14.7705	3.07269	13
3	19	−9	15.6226	3.09038	10
−3	19	9	16.1098	2.99680	10
2	−19	−9	9.81613	2.77040	13
−2	−19	9	4.65210	3.05374	13
−2	19	9	9.99287	2.90079	10
2	19	−9	6.62601	1.73932	11
1	−19	−9	20.7347	3.42102	13
−1	−19	9	20.8462	3.76372	13
1	19	−9	23.4674	1.83298	11
1	19	−9	23.0407	3.51404	14
−1	19	9	22.3451	4.11008	10
1	19	−9	21.7013	1.80474	11
0	−19	−9	6.95842	2.55278	13
0	19	−9	7.22256	2.91105	14
0	19	9	8.25551	3.40758	10
−1	19	−9	15.6025	1.96669	9
−1	19	−9	14.3068	3.42415	14
−2	19	−9	44.3885	2.93480	6
−2	19	−9	41.1349	4.52023	14
−2	19	−9	44.8390	3.27863	9
2	19	9	50.2150	5.35070	10
3	19	9	6.81099	2.40876	10
6	−20	−9	1.31926	1.77428	13
5	−20	−9	6.10819	2.18377	13
−5	−20	9	7.13876	2.20321	13
5	20	−9	8.52842	2.40448	10
4	−20	−9	27.4850	4.52672	13
−4	−20	9	20.2158	3.33055	13
−4	20	9	27.1841	2.10369	11
4	20	−9	20.4177	3.51995	10
−4	20	9	28.1740	2.07534	11
3	−20	−9	18.1486	3.70614	13
−3	−20	9	24.0657	4.29670	13
3	20	−9	16.1211	2.49684	11
−3	20	9	21.4563	2.62916	11
−2	−20	9	0.66421	2.43752	13
2	−20	−9	1.03881	2.58235	13
−2	20	9	2.72081	2.42560	10
2	20	−9	2.33526	1.44535	11
1	−20	−9	2.78874	2.64144	13
−1	20	9	3.65095	3.07249	10
0	−20	−9	9.87319	2.63511	13

0	20	9	6.92021	2.53804	10
−1	20	−9	27.8459	1.94778	6
−1	20	−9	28.7016	4.54039	14
−1	20	−9	23.9171	2.42546	9
1	20	9	27.9613	4.86862	10
2	20	9	8.84401	2.67307	10
−2	20	−9	13.7854	1.45830	9
5	−21	−9	10.2878	2.06147	13
5	21	−9	5.20504	2.20074	10
4	−21	−9	0.97793	2.04859	13
−4	−21	9	1.96349	1.91225	13
4	21	−9	0.30375	1.68385	11
4	21	−9	1.91516	1.53482	10
3	−21	−9	13.2044	2.79089	13
−3	−21	9	11.1936	2.58962	13
−3	21	9	13.8029	1.68469	11
3	21	−9	11.4032	2.03966	11
2	−21	−9	13.6802	2.85296	13
−2	−21	9	11.7924	2.59994	13
−2	21	9	11.7354	2.38090	10
1	−21	−9	11.2518	2.66143	13
−1	−21	9	14.3761	2.59548	13
−1	21	9	11.4322	2.89995	10
0	−21	−9	4.98659	2.26184	13
0	21	9	4.30721	2.83161	10
−1	21	−9	10.9021	1.32570	9
1	21	9	9.20111	2.52799	10
2	21	9	5.94835	2.13294	10
4	−22	−9	13.8672	2.35179	13
−4	−22	9	14.7361	2.79216	13
4	22	−9	12.7576	1.71838	11
−3	−22	9	1.91119	1.90926	13
3	−22	−9	2.47926	2.53545	13
−3	22	9	2.25766	0.71516	11
−3	22	9	2.44556	0.66985	11
2	−22	−9	4.83797	2.21121	13
−2	−22	9	3.80333	2.04709	13
−2	22	9	3.00282	1.26198	11
1	−22	−9	5.96340	2.22416	13
−1	−22	9	5.80970	1.99178	13
−1	22	9	5.94674	2.03397	10
0	22	9	2.69775	2.26221	10
1	22	9	8.35966	2.58671	10
−3	−23	9	15.3269	2.35536	13
3	−23	−9	17.5424	3.13162	13
2	−23	−9	4.54201	2.01763	13
−2	−23	9	3.53100	1.81241	13
−2	23	9	4.77123	1.00909	11
−1	−23	9	−0.2062	1.44541	13
1	−23	−9	2.34825	1.75962	13

-1	23	9	2.11086	2.11456	10
0	23	9	3.88074	1.75277	10
-9	0	10	18.1355	2.76242	10
9	0	-10	16.6929	3.34874	13
9	0	-10	19.9557	3.49277	13
-8	0	10	24.7507	4.06813	10
8	0	-10	25.6693	4.38144	13
7	0	-10	33.9154	5.71618	13
-7	0	10	28.4821	4.82831	10
6	0	-10	7.59852	3.04761	13
-6	0	10	8.84127	3.03162	10
-5	0	10	34.1345	5.21449	10
5	0	-10	31.4036	5.70713	13
0	0	-10	1107.35	67.9296	8
-1	0	-10	7.64316	1.90816	8
-1	0	-10	7.05646	2.48319	10
-1	0	-10	6.79757	3.10081	1
2	0	10	29.1243	4.88008	13
-2	0	-10	32.0865	3.87131	8
-2	0	-10	25.2214	4.54826	10
3	0	10	3.62055	2.76012	13
-3	0	-10	4.26770	1.75588	8
-3	0	-10	5.19957	2.25864	10
-4	0	-10	45.1347	5.28101	12
-4	0	-10	54.9705	4.83881	8
4	0	10	51.3243	6.24035	13
5	0	10	1.37168	3.17659	13
-5	0	-10	2.30459	2.04821	12
-5	0	-10	1.23262	1.24114	8
6	0	10	66.1342	6.12327	13
9	-1	-10	25.4050	4.29344	13
-9	-1	10	24.5769	3.25752	10
9	1	-10	27.2906	4.67168	13
9	1	-10	24.6001	4.42281	13
-9	1	10	23.8980	3.09308	10
-8	-1	10	1.45201	2.38581	10
8	-1	-10	-1.1258	2.98344	13
-8	1	10	0.67261	2.11554	10
8	1	-10	-0.3279	3.13259	13
-7	-1	10	57.2643	6.01152	10
7	-1	-10	44.7148	6.51310	13
-7	1	10	39.3163	5.52995	10
-6	-1	10	28.3718	4.77269	10
6	-1	-10	23.5465	4.34267	13
-6	1	10	24.2181	4.40483	10
6	1	-10	22.8895	3.83090	13
-5	-1	10	2.71667	2.65780	10
-5	1	10	0.52604	2.58351	10
5	1	-10	0.60567	3.19861	13
1	1	-10	1096.46	67.3285	8

0	−1	−10	150.467	9.22530	8
0	1	−10	110.564	8.94153	8
−1	−1	−10	143.543	12.1134	1
−1	−1	−10	141.647	10.3834	8
−1	−1	−10	146.077	10.9201	10
−1	1	−10	157.035	11.1047	10
−1	1	−10	160.559	11.9200	1
−1	1	−10	150.742	10.2099	8
−2	−1	−10	116.348	7.74294	8
−2	−1	−10	109.615	9.02915	1
−2	−1	−10	110.915	8.35700	10
2	−1	10	105.646	9.47367	13
−2	1	−10	97.6713	7.47259	8
−2	1	−10	89.3389	8.12480	10
2	1	10	96.1078	9.40983	13
3	−1	10	16.6199	3.44755	13
−3	−1	−10	16.3108	2.54005	8
−3	−1	−10	15.9251	2.78929	10
3	1	10	15.2665	4.00006	13
−3	1	−10	19.5613	2.75075	8
−3	1	−10	18.7252	3.11999	10
−4	−1	−10	111.532	8.85246	8
4	−1	10	127.829	10.1152	13
−4	−1	−10	107.077	9.29819	12
−4	1	−10	143.728	9.60703	12
−4	1	−10	145.448	8.96789	8
−5	−1	−10	9.22911	1.28814	11
−5	−1	−10	9.08361	2.31892	12
−5	−1	−10	10.0999	1.75946	8
5	−1	10	9.57830	2.84281	13
−5	1	−10	9.27710	1.71857	8
−5	1	−10	8.08419	2.36773	12
5	1	10	7.72640	2.98800	13
6	−1	10	2.20641	2.69878	13
−6	−1	−10	6.96012	1.57163	8
6	1	10	8.47494	3.07942	13
−9	−2	10	17.3110	3.15636	10
9	−2	−10	25.2047	3.75729	13
−9	2	10	22.8636	2.84730	7
9	2	−10	16.5962	3.71422	13
−9	2	10	23.6695	2.36045	11
8	−2	−10	37.4967	5.85520	13
8	2	−10	32.1058	5.79793	13
−8	2	10	38.0900	4.33904	10
7	−2	−10	68.0154	8.09849	13
−7	−2	10	89.2216	7.96730	10
−7	2	10	81.9545	7.53526	10
7	2	−10	90.5915	8.26400	13
6	−2	−10	8.80576	3.01514	13
−6	−2	10	5.20419	2.68765	10

6	2	−10	6.11181	2.81291	13
−6	2	10	5.55741	2.54641	10
−5	−2	10	34.1777	7.33964	1
−4	−2	10	6.76901	4.18685	1
−4	2	10	1.07666	2.91609	10
1	2	−10	8.68086	2.53128	8
0	−2	−10	25.2232	3.32292	8
−1	2	−10	37.7321	3.98806	8
−1	2	−10	40.1248	5.20082	1
−1	2	−10	52.0928	5.11786	10
−2	−2	−10	25.5275	4.31743	10
2	−2	10	33.5684	5.42318	13
−2	−2	−10	31.0549	4.10809	8
−2	−2	−10	23.2590	5.23385	1
−2	2	−10	34.9169	3.87450	10
−2	2	−10	39.8028	3.55246	8
−3	−2	−10	13.8231	2.92167	10
3	−2	10	17.8467	3.84410	13
−3	−2	−10	12.1159	2.33240	8
−3	2	−10	12.7913	2.77382	14
−3	2	−10	11.2744	2.03838	8
−3	2	−10	9.67347	2.71986	12
3	2	10	11.9654	3.37653	13
4	−2	10	29.6769	4.66717	13
−4	−2	−10	26.4078	3.59017	8
−4	−2	−10	21.4430	3.48662	12
−4	−2	−10	22.5774	3.71150	10
4	2	10	36.2082	5.90867	13
−5	−2	−10	30.5832	4.01625	12
−5	−2	−10	29.7224	3.33325	8
5	−2	10	31.4847	4.90809	13
−5	2	−10	37.9952	3.05086	8
−5	2	−10	34.8826	4.18640	12
5	2	10	32.1381	6.16254	13
6	−2	10	8.41333	2.72482	13
−6	−2	−10	5.52316	1.28422	8
6	2	10	5.90582	2.54665	13
9	−3	−10	1.20944	2.71896	13
−9	−3	10	0.18491	1.87003	10
−9	3	10	2.37527	1.03575	11
9	3	−10	−2.0789	2.30019	13
−9	3	10	0.18759	1.41742	7
−8	−3	10	12.7355	2.67913	10
8	3	−10	11.1141	3.46411	13
−8	3	10	13.3417	2.66264	10
8	3	−10	12.7450	3.33529	13
−7	−3	10	43.6336	5.80632	10
7	3	−10	34.8521	5.28922	13
6	−3	−10	21.4690	3.75124	13
−6	−3	10	19.4094	3.81524	10

−6	−3	10	24.8576	6.69442	1
6	3	−10	17.9921	3.54359	13
−6	3	10	25.1221	4.21383	10
−5	−3	10	7.92246	3.01884	10
−5	−3	10	10.1552	4.65472	1
−4	−3	10	13.0800	4.86717	1
−4	3	10	20.6683	4.05109	10
0	3	−10	322.497	20.5320	8
−1	−3	−10	12.2007	3.60239	10
−1	3	−10	15.3010	2.41309	8
−1	3	−10	19.4910	3.43277	10
2	−3	10	1.61631	3.35338	13
−2	−3	−10	7.49963	2.50165	10
−2	−3	−10	9.15399	3.50323	1
−2	−3	−10	7.70871	2.10823	8
−2	3	−10	3.73750	1.63763	10
2	3	10	8.04531	3.60812	13
−3	−3	−10	3.21950	2.60038	10
−3	−3	−10	1.15001	2.12994	8
−3	3	−10	1.11893	1.68853	8
−3	3	−10	0.84835	2.19664	14
−3	3	−10	1.61809	2.71213	12
3	3	10	−0.3837	2.76521	13
−4	−3	−10	198.287	12.7227	8
−4	−3	−10	187.001	13.0033	12
−4	−3	−10	185.253	12.8309	10
4	3	10	223.147	14.2048	13
−4	3	−10	173.404	12.1898	8
−4	3	−10	186.622	13.1675	12
−4	3	−10	174.044	11.6143	11
−5	3	−10	5.13181	2.24018	12
−5	3	−10	2.90678	1.25681	8
5	3	10	4.70866	3.76023	13
−6	−3	−10	8.76571	1.55168	8
6	3	10	9.79723	2.71612	13
−6	3	−10	11.9621	2.01325	12
−9	−4	10	19.2701	3.13728	10
9	−4	−10	14.1983	3.23388	13
−9	4	10	16.1538	1.65937	11
9	4	−10	19.2790	4.14881	13
8	−4	−10	−1.2226	2.93140	13
8	4	−10	8.42842	3.34882	13
−8	4	10	0.22048	1.87858	10
7	−4	−10	38.8346	5.68426	13
−7	−4	10	40.6597	5.78280	10
−7	4	10	39.1988	4.94763	10
−6	−4	10	6.36687	4.22983	1
6	−4	−10	3.41460	2.76963	13
−6	−4	10	3.19118	2.69818	10
−6	4	10	4.36041	2.46757	10

−5	−4	10	3.63003	2.85941	10
5	−4	−10	2.93079	3.19485	13
−5	−4	10	3.97092	4.19986	1
−5	4	10	5.83010	2.90688	10
−4	−4	10	57.2730	8.37450	1
4	4	−10	38.5064	5.73666	10
−4	4	10	45.2057	6.67931	10
1	4	−10	154.945	10.5361	8
0	4	−10	406.131	25.5717	8
−1	−4	−10	102.104	8.49965	8
−1	−4	−10	119.565	9.43661	10
−1	−4	−10	112.849	10.9568	1
−1	4	−10	122.803	8.65770	10
−1	4	−10	133.789	8.53364	8
−2	−4	−10	44.9291	5.13679	10
−2	−4	−10	37.7156	4.29334	8
−2	−4	−10	43.5129	6.64571	1
2	4	10	54.6938	6.58099	13
−2	4	−10	37.0642	3.77316	8
−2	4	−10	41.6581	5.69832	14
−2	4	−10	40.5151	4.83192	12
−3	−4	−10	12.1502	2.40781	8
−3	−4	−10	11.7319	2.73595	10
3	4	10	13.8856	3.98276	13
−3	4	−10	10.6885	1.85032	8
−3	4	−10	8.44279	2.54937	14
−3	4	−10	9.04283	2.59414	12
−4	−4	−10	0.76140	1.84736	8
−4	−4	−10	0.62345	1.91416	10
−4	−4	−10	−2.7897	2.07427	12
4	4	10	0.83833	3.21982	13
−4	4	−10	1.22141	3.01860	12
−4	4	−10	2.16643	1.18784	8
−5	−4	−10	−1.1487	1.68060	8
5	4	10	−4.5925	3.15335	13
−5	4	−10	2.31081	2.28563	12
−6	−4	−10	11.9023	2.07292	8
−6	4	−10	12.4240	2.31952	12
6	4	10	9.47466	2.72893	13
9	−5	−10	10.5849	3.36940	13
−9	−5	10	7.52298	2.31522	10
−9	5	10	9.93971	1.08162	11
8	−5	−10	38.3025	5.76145	13
−8	5	10	27.8791	3.47396	7
−8	5	10	26.1639	3.76940	10
−7	−5	10	51.2713	6.37518	10
7	−5	−10	54.7481	6.75288	13
−7	−5	10	51.7646	9.34630	1
−7	5	10	59.5545	5.76468	10
−6	−5	10	78.8425	9.83575	1

−6	−5	10	71.8512	6.95686	10
6	−5	−10	56.3133	7.25706	13
−6	5	10	54.9722	6.18631	10
−5	−5	10	5.59126	4.47825	1
5	−5	−10	11.7668	3.47551	13
−5	5	10	7.18471	2.93964	10
−4	−5	10	73.4851	10.9782	1
4	5	−10	54.3463	6.34449	10
−4	5	10	58.5053	6.64329	10
0	5	−10	17.2651	2.70552	8
−1	−5	−10	40.9136	6.31650	1
−1	5	−10	25.9347	3.41636	10
−1	5	−10	26.2809	3.44828	8
−2	−5	−10	47.9950	5.49271	10
−2	−5	−10	48.5914	4.81440	8
−2	−5	−10	45.1862	7.31097	1
2	5	10	46.3582	6.05482	13
−2	5	−10	56.6060	4.50432	8
−2	5	−10	55.2550	5.35029	12
−3	−5	−10	5.76266	2.92430	1
−3	−5	−10	6.54399	2.12137	8
−3	−5	−10	4.65797	2.40536	10
−3	5	−10	0.44381	2.18171	12
−3	5	−10	1.04999	0.85014	11
−3	5	−10	0.38505	1.00469	11
−3	5	−10	0.96599	2.18648	14
3	5	10	2.71332	3.00925	13
−3	5	−10	0.54514	1.45243	8
−4	−5	−10	123.854	8.86194	8
−4	−5	−10	122.727	9.40349	10
−4	5	−10	119.372	8.16749	8
4	5	10	121.813	10.4748	13
−4	5	−10	126.573	9.38998	12
−5	−5	−10	3.37073	1.85885	8
−5	5	−10	8.04631	1.93052	12
5	5	10	6.48148	3.44992	13
6	5	10	2.47135	2.36768	13
−6	5	−10	5.18138	1.72565	12
9	−6	−10	14.3229	3.00355	13
−9	−6	10	17.0738	3.07968	10
−8	−6	10	105.771	9.15350	10
8	−6	−10	127.535	9.80777	13
−8	6	10	130.683	8.06365	11
−7	−6	10	69.2895	7.73312	10
7	−6	−10	89.5587	8.19662	13
−7	6	10	94.6328	7.27572	10
6	−6	−10	26.1733	3.97409	13
−6	−6	10	22.5949	4.24387	10
−6	−6	10	22.0773	6.18463	1
−6	6	10	22.6682	4.17118	10

-5	-6	10	-0.8895	3.76631	1
5	-6	-10	0.93186	2.38982	13
5	6	-10	1.65159	2.87473	10
-5	6	10	1.03826	2.46193	10
4	-6	-10	15.7841	3.24256	13
4	6	-10	18.9166	3.70912	10
-4	6	10	16.1810	3.79350	10
3	6	-10	110.398	8.71423	10
-3	6	10	93.7148	9.05226	10
0	-6	-10	97.5566	9.41180	10
0	6	-10	132.456	9.12064	10
0	6	-10	126.808	8.55070	8
-1	-6	-10	81.5886	8.67085	1
-1	-6	-10	80.0110	6.82179	10
-1	6	-10	62.7521	6.59042	14
-1	6	-10	58.7953	5.31693	10
-1	6	-10	59.9213	5.10687	8
-1	6	-10	57.1706	6.18660	12
1	6	10	72.8331	6.95260	13
-2	-6	-10	18.7531	4.60342	1
-2	-6	-10	17.1284	2.86539	8
-2	-6	-10	20.4381	3.60660	10
-2	6	-10	21.0722	2.36843	11
-2	6	-10	24.9960	2.70043	8
-2	6	-10	19.7231	3.20437	14
2	6	10	20.9026	3.85576	13
-2	6	-10	19.7514	3.14247	12
3	6	10	56.8353	6.33804	13
-3	6	-10	42.0796	2.95497	11
-3	6	-10	39.9301	3.57089	8
-3	6	-10	42.6668	2.97526	11
-3	6	-10	41.8671	5.05408	12
-3	6	-10	41.1040	5.16751	14
-4	-6	-10	18.5779	3.58857	10
-4	-6	-10	16.8819	2.79534	8
-4	6	-10	24.0626	3.75974	12
4	6	10	20.3827	4.20897	13
-5	-6	-10	7.98501	1.67988	8
-5	-6	-10	5.73765	1.73574	10
5	6	10	5.84241	3.27868	13
-5	6	-10	2.52449	2.27798	12
-5	6	-10	4.99500	1.67913	9
6	6	10	3.53505	2.30514	13
-6	6	-10	3.41541	1.66343	12
-9	-7	10	47.6313	4.57206	10
9	-7	-10	45.2923	5.06040	13
8	-7	-10	61.1678	6.28756	13
-8	-7	10	46.1436	5.15986	10
-8	7	10	57.8334	4.14516	11
7	-7	-10	20.5909	3.82890	13

−7	7	10	20.2326	3.44636	10
−6	−7	10	4.58176	4.43174	1
6	−7	−10	7.98139	3.04581	13
−6	−7	10	4.01376	3.04587	10
−6	7	10	8.32854	2.71591	10
−5	−7	10	65.1434	9.64397	1
5	−7	−10	52.6324	6.57006	13
5	7	−10	68.2425	6.94174	10
−5	7	10	50.1744	6.17449	10
−4	−7	10	35.3055	5.40382	13
4	−7	−10	29.2287	4.88856	13
4	7	−10	45.2727	5.85785	10
−3	7	10	30.0340	4.71068	10
3	7	−10	22.2326	3.84656	10
−2	7	10	11.8031	5.55254	10
2	7	−10	7.82655	2.55252	10
1	7	−10	45.3771	4.82479	10
0	−7	−10	276.011	18.6532	10
0	−7	−10	274.384	21.2771	1
0	7	−10	264.369	17.8644	10
−1	−7	−10	200.027	16.0017	1
−1	−7	−10	197.004	13.6435	10
−1	7	−10	194.602	12.1727	11
1	7	10	177.304	13.5330	13
−1	7	−10	174.075	12.8384	14
−1	7	−10	179.653	12.2558	8
−1	7	−10	176.771	12.7317	12
−2	−7	−10	33.8510	6.67067	1
−2	7	−10	44.2225	3.42487	11
−2	7	−10	50.8214	5.15640	14
−2	7	−10	40.9324	3.90553	8
−2	7	−10	46.8368	5.45040	12
−3	−7	−10	3.31997	3.48168	1
−3	−7	−10	2.91141	2.47694	10
−3	−7	−10	5.09924	2.12088	8
−3	7	−10	3.84191	2.46737	14
3	7	10	3.06121	3.13501	13
−3	7	−10	3.45611	2.45900	12
−4	−7	−10	2.71661	2.07554	8
−4	−7	−10	0.90928	2.19718	10
−4	7	−10	1.23154	2.35710	12
4	7	10	1.61019	3.35775	13
−5	−7	−10	−1.1164	1.52735	10
−5	−7	−10	0.53487	1.71309	8
−5	7	−10	1.49163	0.93256	9
−5	7	−10	0.68574	0.52332	6
−5	7	−10	−1.4382	2.11449	12
5	7	10	1.84226	2.92463	13
−6	7	−10	20.2785	3.30436	12
6	7	10	24.6253	4.46256	13

−9	−8	10	35.9768	3.83259	10
9	−8	−10	31.9543	4.30189	13
8	−8	−10	3.43292	2.76097	13
−8	−8	10	5.75932	2.12734	10
−8	8	10	4.23959	1.08271	11
7	−8	−10	34.7570	6.48135	13
−7	−8	10	24.9682	4.28841	10
−7	8	10	25.1861	3.63297	10
−6	−8	10	13.7916	5.45638	1
−6	−8	10	10.7025	3.76730	10
6	−8	−10	8.49367	3.08422	13
−6	8	10	7.87229	2.59053	10
5	−8	−10	31.8063	5.59180	13
−5	−8	10	18.5507	5.90499	1
5	8	−10	19.1548	4.01088	10
4	−8	−10	34.1000	5.88661	13
−4	−8	10	21.5373	3.78467	13
−4	8	10	27.2062	4.98673	10
4	8	−10	21.9075	4.13343	10
3	−8	−10	20.0458	3.41904	13
−3	−8	10	20.3648	4.14410	13
−3	8	10	19.9442	4.14243	10
3	8	−10	19.4177	3.57711	10
2	8	−10	27.9390	4.39287	10
−2	8	10	29.3846	5.70481	10
−1	8	10	5.13404	3.68657	10
1	8	−10	2.78317	2.50697	10
0	−8	−10	79.9704	9.89149	1
0	−8	−10	68.0757	7.03458	10
0	8	−10	69.7622	6.06478	10
0	8	−10	67.3437	6.53879	14
−1	−8	−10	100.339	11.1413	1
−1	−8	−10	88.5460	8.83590	10
−1	8	−10	118.752	8.35133	12
−1	8	−10	124.556	8.90140	14
1	8	10	84.9598	9.64431	13
−1	8	−10	124.276	7.42651	11
−2	−8	−10	216.063	17.7832	1
−2	−8	−10	206.772	15.0593	10
−2	8	−10	211.116	14.8474	14
−2	8	−10	211.946	14.5988	12
−2	8	−10	218.112	13.4482	11
−2	8	−10	219.108	13.3560	11
2	8	10	213.596	15.2960	13
−3	−8	−10	35.4921	4.23689	8
−3	−8	−10	35.1613	5.96110	1
−3	−8	−10	32.9376	4.70919	10
3	8	10	35.9352	5.43103	13
−3	8	−10	32.9477	4.44224	12
−3	8	−10	33.3691	4.99055	14

−4	−8	−10	0.94907	2.29489	10
−4	−8	−10	3.39757	2.03202	8
−4	8	−10	−0.9984	1.21475	9
−4	8	−10	5.98918	2.45957	12
4	8	10	0.27091	3.16553	13
−5	−8	−10	1.17694	2.07087	10
−5	−8	−10	3.23559	1.88760	8
5	8	10	5.20962	3.01923	13
−5	8	−10	3.69408	0.59057	6
−5	8	−10	8.47711	2.36918	12
−5	8	−10	4.47394	0.95507	9
−6	8	−10	−1.1857	1.48311	12
6	8	10	0.40063	2.34681	13
−9	−9	10	0.86933	2.33249	10
9	−9	−10	3.25470	2.03459	13
−8	−9	10	9.27479	2.48504	10
8	−9	−10	5.85537	2.75702	13
−8	9	10	4.31958	0.85070	11
−8	9	10	3.73546	0.85163	11
7	−9	−10	28.7601	5.09160	13
−7	9	10	29.2934	2.95544	11
−7	9	10	28.8395	3.62419	10
6	−9	−10	38.6785	5.22928	13
−6	−9	10	35.3596	7.46193	1
−6	9	10	32.6256	4.44935	10
6	9	−10	42.0840	6.13956	10
5	−9	−10	10.1918	3.50489	13
5	9	−10	4.31371	3.05308	10
−5	9	10	9.51842	3.07699	10
−4	−9	10	19.6230	3.56073	13
4	−9	−10	20.0667	3.94349	13
4	9	−10	20.7497	3.90967	10
−4	9	10	17.4896	3.79713	10
−3	−9	10	27.3088	4.33622	13
3	9	−10	34.7364	5.14886	10
−2	−9	10	23.5152	4.39300	13
−2	9	10	16.6783	4.25153	10
2	9	−10	24.5089	4.53907	10
−1	−9	10	32.1328	5.39821	13
1	9	−10	39.0155	5.05750	14
1	9	−10	44.2767	4.49699	10
0	−9	10	151.397	10.7963	13
0	−9	−10	130.595	11.0037	10
0	−9	−10	123.114	12.9777	1
0	9	−10	143.057	10.2505	14
0	9	−10	140.909	9.93176	10
−1	−9	−10	250.365	19.1692	1
−1	−9	−10	222.914	15.6378	10
−1	9	−10	207.367	14.8963	14
−1	9	−10	212.734	13.9227	11

1	9	10	233.777	15.8950	13
−1	9	−10	202.857	14.7071	12
−2	−9	−10	29.2512	4.81129	10
−2	−9	−10	30.0784	6.13868	1
−2	9	−10	33.2063	4.66679	14
−2	9	−10	33.7753	2.36475	11
2	9	10	25.5912	4.37236	13
−2	9	−10	33.2906	4.32175	12
−2	9	−10	35.0376	2.39043	11
−3	−9	−10	9.73014	3.18390	10
−3	−9	−10	10.1780	3.80974	1
−3	9	−10	7.67419	2.97086	14
3	9	10	8.20082	4.31805	13
−3	9	−10	4.91228	2.66830	12
−4	−9	−10	2.44081	2.04685	8
−4	−9	−10	2.96190	2.38528	10
−4	9	−10	6.05695	2.45574	12
4	9	10	1.33659	3.10464	13
−4	9	−10	3.21004	2.47974	14
−4	9	−10	3.93313	0.81211	6
−4	9	−10	3.85198	1.06061	9
−5	−9	−10	0.84561	1.74499	8
−5	−9	−10	−0.7890	1.69712	10
−5	9	−10	1.27892	0.50808	6
−5	9	−10	0.74997	0.71554	9
5	9	10	0.13731	3.21697	13
−5	9	−10	1.57711	2.17455	12
−8	−10	10	14.5222	3.19237	10
8	−10	−10	15.4294	3.01160	13
−7	−10	10	1.76891	1.97125	10
7	−10	−10	5.33438	2.91707	13
−7	10	10	7.22251	1.31900	11
6	10	−10	30.0035	5.70499	10
−6	10	10	28.0777	3.91220	10
5	−10	−10	65.0065	6.75373	13
5	10	−10	65.6684	7.08051	10
−5	10	10	68.1777	6.68952	10
−4	−10	10	9.53170	3.05592	13
4	−10	−10	7.51796	3.44184	13
−4	10	10	3.75502	2.93986	10
4	10	−10	9.42729	3.22979	10
3	−10	−10	16.7389	3.55595	13
−3	−10	10	13.2598	4.01767	13
3	10	−10	23.0314	3.77585	10
−3	10	10	14.5499	3.84087	10
−2	−10	10	5.27356	3.69670	13
2	10	−10	9.91510	2.73731	10
−2	10	10	10.8905	3.65492	10
1	−10	−10	107.264	9.51110	13
−1	−10	10	128.954	10.0660	13

-1	10	10	110.819	10.4986	10
1	10	-10	124.173	8.98491	10
1	10	-10	121.860	9.02535	14
0	-10	10	55.7058	7.19700	13
0	10	-10	43.8805	5.23565	14
-1	-10	-10	162.747	14.5179	1
-1	-10	-10	165.111	11.7855	10
-1	10	-10	146.574	9.72692	11
-1	10	-10	145.646	10.9205	14
-1	10	-10	138.424	10.6314	12
-2	-10	-10	49.0996	7.40558	1
-2	-10	-10	49.2863	5.57562	10
-2	10	-10	33.9348	4.50487	12
-2	10	-10	34.7393	4.98592	14
2	10	10	45.4209	5.99576	13
-3	10	-10	18.8040	3.80183	14
3	10	10	15.8030	3.83457	13
-3	10	-10	10.6785	2.91594	12
-3	10	-10	21.6022	2.19443	9
-4	-10	-10	41.8007	4.13927	8
-4	-10	-10	35.4923	4.33953	10
4	10	10	39.2741	5.92559	13
-4	10	-10	58.5991	5.50801	14
-4	10	-10	46.2720	3.30488	9
-4	10	-10	47.8024	3.08058	6
-4	10	-10	52.0369	5.26250	12
-5	-10	-10	6.22500	1.56975	8
-5	-10	-10	5.07964	1.92895	10
-5	10	-10	3.48314	0.78447	9
-5	10	-10	3.44651	2.10992	12
5	10	10	2.89984	2.53457	13
-5	10	-10	3.26866	0.55005	6
-8	-11	10	16.7069	3.20736	10
8	-11	-10	25.7705	4.72518	13
7	-11	-10	-0.1364	2.84610	13
-7	-11	10	1.51989	2.06189	10
-7	11	10	3.96837	1.19563	11
7	11	-10	2.61700	2.44972	10
6	-11	-10	19.0411	3.66307	13
6	11	-10	15.5944	4.01788	10
-6	11	10	18.7857	3.08001	10
5	-11	-10	10.7963	3.33345	13
-5	-11	10	15.8716	3.08038	13
-5	11	10	15.2473	3.26879	10
5	11	-10	19.5036	3.93801	10
4	-11	-10	24.9527	3.78577	13
-4	-11	10	18.3971	3.93813	13
4	11	-10	15.4092	3.67965	10
-4	11	10	23.8790	4.60321	10
-3	-11	10	38.2870	6.08198	13

3	−11	−10	33.8370	5.88543	13
−3	11	10	33.4156	5.27952	10
3	11	−10	40.5061	4.98287	10
−2	−11	10	12.5311	3.35159	13
2	−11	−10	28.1258	4.63837	13
2	11	−10	18.8753	3.31541	10
−2	11	10	29.4948	5.41695	10
−1	−11	10	43.1410	6.15124	13
1	−11	−10	38.8827	5.25206	13
−1	11	10	45.7073	6.53438	10
1	11	−10	45.4243	5.13975	14
1	11	−10	49.5412	5.02955	10
0	−11	−10	30.6548	6.45077	1
0	11	−10	17.1428	3.20074	14
0	11	−10	19.5159	2.46423	11
0	11	10	25.3670	4.83765	10
−1	11	−10	47.8090	5.29537	14
−1	11	−10	47.8006	3.53853	11
−1	11	−10	46.4735	3.80552	11
−2	−11	−10	13.2160	3.48089	10
−2	−11	−10	16.6373	5.50546	1
−2	11	−10	16.8718	3.64045	14
−2	11	−10	16.6602	3.36515	12
−3	−11	−10	23.1598	4.32556	10
−3	11	−10	18.0103	1.89887	6
−3	11	−10	18.7490	3.32957	12
−3	11	−10	18.3380	3.41843	14
3	11	10	21.0837	4.28151	13
−3	11	−10	16.5990	2.06720	9
−4	−11	−10	4.17952	1.95228	10
−4	11	−10	4.69093	1.15092	9
−4	11	−10	5.03976	0.71708	6
4	11	10	3.20655	2.85999	13
−5	−11	−10	0.67198	1.75326	10
−5	11	−10	0.72352	0.60546	9
5	11	10	0.32343	2.54214	13
−5	11	−10	1.05090	1.94694	12
−5	11	−10	1.77176	0.71650	9
−8	−12	10	7.18896	2.05210	10
7	−12	−10	17.5621	3.26979	13
7	12	−10	9.99245	3.64468	10
−7	12	10	18.3472	1.81521	11
−7	12	10	18.7599	2.00592	11
6	−12	−10	12.3004	3.57727	13
−6	12	10	9.51549	2.14350	10
−6	12	10	11.1411	1.99020	11
5	−12	−10	0.47681	3.00790	13
−5	−12	10	1.00494	2.66165	13
5	12	−10	−2.4995	3.10833	10
−5	12	10	−0.4577	2.84030	10

4	−12	−10	26.2256	3.96461	13
−4	−12	10	25.8375	4.75189	13
−4	12	10	24.9930	4.64750	10
4	12	−10	21.2997	3.93825	10
3	−12	−10	16.7508	3.83564	13
−3	−12	10	13.0701	3.69971	13
−3	12	10	14.4841	3.62654	10
3	12	−10	8.09641	2.85586	10
−2	−12	10	3.78078	3.24926	13
2	−12	−10	−0.6840	2.43856	13
2	12	−10	6.10298	2.75440	10
−2	12	10	0.33342	3.06354	10
−1	−12	10	17.1964	3.36168	13
1	−12	−10	11.2581	2.96488	13
1	12	−10	15.9099	2.91297	10
1	12	−10	16.2330	3.01546	14
−1	12	10	9.60355	3.93723	10
0	−12	−10	48.6857	9.26557	1
0	12	10	46.5316	6.44487	10
0	12	−10	32.1847	3.65444	11
0	12	−10	31.8418	5.43498	14
−1	−12	−10	12.6929	4.86003	1
−1	−12	−10	11.0230	3.39825	10
−1	12	−10	7.85058	1.07296	11
−1	12	−10	8.01125	1.03913	11
−1	12	−10	5.13202	2.59127	14
−2	−12	−10	4.10443	3.17025	10
−2	−12	−10	0.11086	3.39261	1
−2	12	−10	−1.9964	2.74408	12
−3	−12	−10	27.7278	4.52932	10
−3	12	−10	23.3108	2.52674	9
−3	12	−10	28.6116	2.13774	6
−4	12	−10	7.97406	2.43635	12
−4	12	−10	5.90619	0.69661	6
−4	12	−10	7.02038	1.13845	9
−4	12	−10	9.08160	2.66179	14
−5	−12	−10	3.83578	1.78057	10
−5	12	−10	6.95422	2.05969	12
8	−13	−10	3.63800	1.95103	13
7	−13	−10	−0.7789	2.34700	13
7	13	−10	2.27516	3.16119	10
6	−13	−10	57.5633	6.44893	13
6	13	−10	68.2220	6.25573	10
−6	13	10	57.1628	4.49542	11
−5	−13	10	12.6953	3.52993	13
5	−13	−10	16.5478	3.82241	13
−5	13	10	18.0818	3.14612	10
5	13	−10	8.01045	3.52439	10
−4	−13	10	14.4285	3.84745	13
4	−13	−10	19.7447	4.11537	13

4	13	−10	12.8094	3.41846	10
−4	13	10	16.6205	3.51009	10
3	−13	−10	19.0480	3.92024	13
−3	−13	10	12.2954	3.69302	13
3	13	−10	14.5343	3.62939	10
−3	13	10	19.2119	3.90498	10
2	−13	−10	6.16207	2.66363	13
−2	−13	10	10.9790	2.98429	13
2	13	−10	11.8243	2.97645	10
−2	13	10	5.94963	3.39982	10
−1	−13	10	6.87512	3.16147	13
1	−13	−10	1.77249	2.32263	13
1	13	−10	8.39149	2.32507	10
1	13	−10	6.22470	2.53377	14
−1	13	10	3.28431	3.39655	10
0	−13	−10	20.7667	6.16030	1
0	13	−10	35.3896	3.21766	11
0	13	10	23.6728	4.83425	10
0	13	−10	33.0232	4.83149	14
−1	−13	−10	37.2562	7.68986	1
1	13	10	31.0306	7.45058	10
−2	−13	−10	24.2779	4.28890	10
−2	13	−10	23.8464	3.79034	14
−2	13	−10	25.4580	3.93019	12
−3	−13	−10	12.4324	3.12586	10
−3	13	−10	6.99392	1.24194	9
−3	13	−10	6.59042	2.89955	14
−3	13	−10	7.59327	0.95553	6
−3	13	−10	8.18228	2.53269	12
−4	−13	−10	1.21178	2.38812	10
−4	13	−10	1.51804	2.26518	14
−4	13	−10	1.62333	0.77905	9
−4	13	−10	4.31531	2.25571	12
−4	13	−10	2.28445	0.43438	6
−4	13	−10	2.29434	0.42526	6
−5	−13	−10	3.38140	1.71971	10
−5	13	−10	4.62420	1.83683	12
7	−14	−10	2.92555	2.63430	13
7	14	−10	11.5275	2.63167	10
6	14	−10	8.79257	3.71304	10
−6	14	10	16.3932	1.96074	11
5	−14	−10	9.46485	3.55959	13
5	14	−10	16.8255	3.99244	10
−5	14	10	12.3316	2.95900	10
4	−14	−10	11.0386	3.45423	13
−4	−14	10	6.42716	3.71151	13
4	14	−10	8.91619	3.23853	10
3	−14	−10	49.2249	5.92430	13
−3	−14	10	46.5895	5.80251	13
−3	14	10	44.5082	5.93557	10

3	14	-10	49.2204	5.50948	10
-2	-14	10	3.23905	3.19466	13
2	-14	-10	0.90672	2.94157	13
2	14	-10	1.72491	2.07859	10
-2	14	10	1.42477	3.20730	10
-1	-14	10	6.35643	3.08885	13
1	-14	-10	4.42641	3.01649	13
-1	14	10	4.16625	3.49849	10
1	14	-10	1.56053	2.45862	14
1	14	-10	7.86655	2.20879	11
0	14	-10	62.1967	6.58373	14
0	14	-10	70.1930	5.36009	11
0	14	-10	70.3756	5.00670	11
-1	-14	-10	46.5271	8.18604	1
1	14	10	45.6659	5.99959	10
-2	14	-10	4.87429	2.86972	14
-2	14	-10	4.68070	2.78021	12
-2	14	-10	0.85400	1.23784	9
-3	14	-10	2.08249	0.60578	6
-3	14	-10	0.97488	2.60250	14
-3	14	-10	1.68460	0.88770	9
-3	14	-10	1.23722	1.68095	12
-4	14	-10	1.74696	0.62928	9
-4	14	-10	5.32597	2.24598	14
-4	14	-10	2.25879	2.09075	12
-4	14	-10	2.77350	0.61283	9
7	-15	-10	10.7977	2.36064	13
7	15	-10	11.2842	3.13858	10
6	-15	-10	13.0072	3.06400	13
-6	15	10	12.9970	1.59217	11
-6	15	10	13.2278	1.68491	11
5	-15	-10	3.19304	2.89959	13
-5	-15	10	5.79332	2.43890	13
-5	15	10	4.16880	1.40988	11
-5	15	10	4.99414	2.10002	10
5	15	-10	4.71133	3.09536	10
-4	-15	10	25.9969	3.86546	13
4	15	-10	25.2679	4.65092	10
-3	-15	10	27.5435	4.67354	13
3	-15	-10	23.5708	4.12279	13
3	15	-10	34.1846	4.59612	10
-3	15	10	23.9249	4.45022	10
2	-15	-10	39.5758	5.40495	13
-2	-15	10	46.0325	6.12042	13
-2	15	10	42.3407	6.17740	10
2	15	-10	47.5223	5.08638	10
-1	-15	10	5.96846	2.59582	13
1	-15	-10	3.25460	2.51266	13
1	15	-10	5.28934	2.77153	14
1	15	-10	5.93933	1.80062	11

−1	15	10	4.24259	3.57548	10
0	15	−10	130.170	10.5723	14
0	15	10	161.045	11.9879	10
0	15	−10	137.666	8.86072	11
0	15	−10	133.458	8.76323	11
1	15	10	37.6497	6.29030	10
−1	15	−10	32.9483	5.37199	14
−2	−15	−10	7.69730	3.06903	10
−2	15	−10	8.11772	2.84418	14
−2	15	−10	7.11680	1.26754	9
−2	15	−10	6.15778	3.76760	12
−2	15	−10	7.88310	0.91279	6
−3	−15	−10	0.01288	1.75118	10
−3	15	−10	0.88940	2.56161	12
−3	15	−10	0.91521	0.48518	6
−3	15	−10	−1.0288	0.86657	9
−3	15	−10	−1.5075	2.38159	14
−4	−15	−10	1.77352	1.58973	10
−4	15	−10	3.43698	1.98306	14
−4	15	−10	4.23287	1.93851	12
7	−16	−10	4.24799	1.88342	13
7	16	−10	2.33159	1.83335	10
6	−16	−10	2.44725	2.24594	13
6	16	−10	4.35140	2.99596	10
−5	−16	10	−1.9753	2.28693	13
5	−16	−10	0.15527	2.96642	13
−5	16	10	−1.7920	1.05923	11
5	16	−10	−0.1507	3.04805	10
−4	−16	10	13.0036	3.08325	13
4	−16	−10	10.2101	3.31389	13
4	16	−10	15.0370	3.40338	10
−4	16	10	12.8262	2.85822	10
3	−16	−10	7.13659	3.08864	13
−3	−16	10	5.17827	2.77236	13
−3	16	10	9.96637	3.13822	10
3	16	−10	5.97293	2.57452	10
−2	−16	10	10.4365	3.58231	13
2	−16	−10	12.1696	3.68135	13
2	16	−10	12.5750	2.63259	10
1	−16	−10	82.6860	7.27561	13
−1	−16	10	75.0134	7.74593	13
−1	16	10	86.6789	8.25332	10
1	16	−10	71.0630	7.13091	14
1	16	−10	71.0060	5.78093	11
0	16	−10	13.5885	3.29682	14
0	16	10	14.8373	4.15441	10
1	16	10	0.80661	3.44781	10
−1	16	−10	1.27497	2.57447	14
−2	−16	−10	20.5202	4.09086	10
2	16	10	21.4682	4.73820	10

−2	16	−10	22.7605	2.26952	9
−2	16	−10	25.5874	1.87278	6
−3	−16	−10	0.96624	2.40269	10
−3	16	−10	5.15680	2.43260	14
−3	16	−10	3.52242	1.95039	12
−3	16	−10	1.81292	0.48219	6
−3	16	−10	1.69731	0.75719	9
−3	16	−10	1.62686	0.45047	6
−4	−16	−10	3.77084	2.10069	10
−4	16	−10	7.20528	1.88140	12
6	−17	−10	20.6200	3.68764	13
6	17	−10	29.4450	4.38440	10
−5	−17	10	3.33564	2.55121	13
5	−17	−10	5.63920	2.80974	13
−5	17	10	7.46517	1.23204	11
5	17	−10	5.26605	2.95899	10
4	−17	−10	20.6287	3.70263	13
−4	17	10	17.2405	3.17293	10
4	17	−10	26.0578	4.34610	10
−3	−17	10	3.29587	2.64670	13
3	−17	−10	7.00320	3.14808	13
−3	17	10	9.27352	2.83431	10
3	17	−10	6.10422	2.53441	10
−2	−17	10	3.83127	2.83669	13
2	−17	−10	6.81111	2.95154	13
2	17	−10	1.84118	1.91904	11
1	−17	−10	57.7750	7.00848	13
−1	−17	10	60.3981	6.42480	13
−1	17	10	66.6996	7.35145	10
1	17	−10	63.0884	4.74841	11
1	17	−10	67.1702	4.67234	11
1	17	−10	59.8190	6.46399	14
0	17	−10	10.7164	3.09096	14
0	17	10	9.10415	3.79784	10
1	17	10	−0.7114	2.46049	10
−1	17	−10	3.05323	2.73461	14
2	17	10	3.33090	2.56756	10
−2	17	−10	9.06844	2.76948	14
−2	17	−10	3.97792	0.61396	6
−2	17	−10	4.47323	1.01866	9
−3	−17	−10	2.12979	1.66787	10
−3	17	−10	2.00209	0.64641	9
−3	17	−10	2.39424	2.23451	14
−3	17	−10	2.30220	0.64007	9
−3	17	−10	2.72228	1.76034	12
6	−18	−10	18.3527	3.10111	13
6	18	−10	15.8229	3.01440	10
5	−18	−10	1.60869	2.27750	13
−5	−18	10	0.46665	2.18230	13
−5	18	10	0.40510	0.63456	11

5	18	−10	−1.1738	2.22738	10
−5	18	10	0.27194	0.56767	11
4	−18	−10	24.1487	3.67720	13
−4	−18	10	22.5320	3.34920	13
−4	18	10	24.6863	2.99637	11
4	18	−10	21.6806	3.77001	10
3	−18	−10	9.13624	3.14231	13
−3	−18	10	6.90090	2.86428	13
3	18	−10	11.6285	2.79447	10
2	−18	−10	13.6442	3.74975	13
−2	−18	10	22.7969	3.43976	13
2	18	−10	23.2464	3.16299	11
−2	18	10	13.8248	3.52194	10
1	−18	−10	6.16380	2.71493	13
−1	−18	10	3.97626	2.37576	13
−1	18	10	5.75848	3.35745	10
1	18	−10	6.06471	2.72191	14
0	18	10	9.94784	3.20849	10
1	18	10	71.2555	7.39651	10
−1	18	−10	72.5696	6.70268	14
−2	18	−10	5.69806	0.69281	6
2	18	10	11.4266	3.45278	10
−2	18	−10	5.97369	1.09269	9
−2	18	−10	6.68348	2.52752	14
−3	−18	−10	5.83203	1.82262	10
−5	−19	10	0.20900	1.90935	13
5	−19	−10	2.99245	2.04171	13
5	19	−10	1.26182	2.02155	10
4	−19	−10	7.32338	2.75557	13
−4	−19	10	5.21490	2.36306	13
−4	19	10	7.75650	1.34212	11
4	19	−10	7.84869	2.20645	10
3	−19	−10	2.80926	2.68145	13
−3	−19	10	2.98114	2.33026	13
−3	19	10	2.62160	1.90589	10
3	19	−10	0.83244	1.75054	11
−2	−19	10	23.5009	3.50862	13
2	19	−10	20.8412	2.90408	11
−2	19	10	28.9247	3.58764	10
2	19	−10	16.4521	2.96699	11
−1	−19	10	1.96161	2.23198	13
1	−19	−10	0.74831	2.94594	13
−1	19	10	2.37880	3.03998	10
1	19	−10	2.11975	2.40345	14
0	19	−10	19.6012	3.26754	14
0	19	10	21.4903	3.69885	10
−1	19	−10	10.1264	1.41392	9
−1	19	−10	11.0339	2.84680	14
1	19	10	11.4630	2.88761	10
−2	19	−10	7.85677	2.33358	14

-2	19	-10	7.71648	1.00463	9
2	19	10	9.29313	3.17304	10
5	-20	-10	11.0114	2.24431	13
5	20	-10	13.1864	3.87184	10
-4	-20	10	1.30127	1.87175	13
4	-20	-10	1.83256	2.10435	13
4	20	-10	1.68117	1.76284	11
4	20	-10	0.94652	1.73464	10
-4	20	10	1.52814	0.75048	11
-4	20	10	1.67562	0.70716	11
-3	-20	10	-0.3919	1.94581	13
3	-20	-10	1.60897	2.25676	13
3	20	-10	0.43822	1.25471	11
-3	20	10	1.36075	1.21039	11
-2	-20	10	11.4812	2.67193	13
2	-20	-10	17.8695	3.14466	13
-2	20	10	11.9727	3.14835	10
1	-20	-10	-2.0483	2.21429	13
-1	-20	10	0.35134	2.32709	13
-1	20	10	-1.8196	2.32726	10
0	20	10	1.45661	2.64939	10
1	20	10	0.36464	2.65387	10
-1	20	-10	1.53720	0.94130	9
2	20	10	46.6970	4.67589	10
-4	-21	10	1.28810	1.99088	13
4	-21	-10	1.34359	1.87886	13
4	21	-10	2.00266	1.16120	11
-3	-21	10	15.0621	2.72978	13
3	-21	-10	11.8822	2.50345	13
3	21	-10	14.0347	1.76964	11
-3	21	10	11.3862	1.50269	11
3	21	-10	15.4447	1.79807	11
2	-21	-10	53.5684	5.18074	13
-2	-21	10	58.3960	5.12568	13
-2	21	10	51.2888	4.83234	10
-1	-21	10	3.97390	2.14049	13
1	-21	-10	4.80826	2.36186	13
-1	21	10	3.08460	2.08403	10
0	21	10	3.43834	1.85354	10
1	21	10	1.22275	2.23300	10
3	-22	-10	18.4605	2.67004	13
-3	-22	10	22.2992	3.16641	13
2	-22	-10	37.4568	4.09806	13
-2	-22	10	50.8087	4.49469	13
-2	22	10	40.9213	3.60020	11
1	-22	-10	17.7800	3.06240	13
-1	-22	10	14.4009	2.46445	13
-1	22	10	16.3231	2.94181	10
0	22	10	6.71069	2.08686	10
-9	0	11	1.31366	1.75434	10

9	0	−11	3.39072	2.72118	13
−8	0	11	18.3517	3.25140	10
8	0	−11	14.3493	3.71569	13
−7	0	11	7.29611	2.98267	10
7	0	−11	8.42185	3.82702	13
6	0	−11	−0.2805	2.59613	13
−6	0	11	−0.1296	2.50358	10
−5	0	11	15.4730	3.62139	10
5	0	−11	11.5924	3.01424	13
−4	0	11	12.9957	3.89752	10
3	0	−11	1.97656	2.64454	10
1	0	−11	42.0784	5.92230	10
0	0	−11	353.620	23.0963	10
0	0	11	349.630	23.4509	13
−1	0	−11	0.43362	2.58828	1
−1	0	−11	−1.7017	1.69961	8
−1	0	−11	−3.4123	2.09867	10
1	0	11	−5.1553	3.33047	13
2	0	11	5.80282	3.58832	13
−2	0	−11	6.91272	2.26782	10
−2	0	−11	6.73422	2.30862	8
−3	0	−11	6.93366	1.93405	8
−3	0	−11	5.83067	2.14517	10
−3	0	−11	8.51394	2.72504	12
−4	0	−11	11.6315	2.02156	8
4	0	11	13.1548	3.90321	13
−4	0	−11	10.5598	2.63431	12
−5	0	−11	4.41113	1.43594	8
−5	0	−11	3.74679	1.94834	12
5	0	11	2.12637	2.66738	13
6	0	11	21.7927	3.41236	13
−9	−1	11	4.68498	2.07986	10
9	−1	−11	7.83217	2.46286	13
9	−1	−11	6.03972	2.36920	13
−9	1	11	7.91722	1.40875	11
−9	1	11	7.28787	1.77057	10
9	1	−11	4.28404	2.71652	13
8	−1	−11	17.7410	3.68439	13
−8	−1	11	10.5693	2.52769	10
8	1	−11	16.6290	3.58724	13
−8	1	11	15.0665	2.96168	10
−7	−1	11	7.64340	2.44898	10
7	1	−11	5.70592	3.18248	13
6	−1	−11	7.27712	3.54153	13
−6	−1	11	2.30157	2.69146	10
6	1	−11	3.77020	3.38130	13
−6	1	11	5.93174	2.92707	10
5	−1	−11	3.06449	3.18446	13
−5	−1	11	1.11734	1.83068	10
−5	1	11	5.17291	3.04934	10

−4	1	11	44.2713	6.23851	10
3	1	−11	41.6145	5.36207	10
2	−1	−11	44.5374	5.65592	10
1	−1	−11	25.4356	4.67058	10
1	1	−11	34.0304	4.72508	10
1	1	−11	28.0328	3.83296	8
0	−1	−11	40.3243	5.02596	10
0	−1	11	44.7463	6.16059	13
0	1	11	37.3800	5.45069	13
0	1	−11	34.8353	4.21518	8
0	1	−11	31.3830	4.61199	10
−1	−1	−11	24.1885	4.19719	10
−1	−1	−11	20.9835	3.24194	8
−1	−1	−11	18.3490	4.38873	1
1	−1	11	25.3410	4.64128	13
−1	1	−11	25.3317	3.85100	10
−1	1	−11	24.3156	3.30785	8
−1	1	−11	26.1779	4.67485	1
2	−1	11	−1.0664	3.27405	13
−2	−1	−11	3.67987	1.83659	8
−2	−1	−11	4.56945	2.18640	10
−2	1	−11	−0.2847	1.60837	8
−2	1	−11	0.04419	2.03617	10
2	1	11	2.20098	2.83570	13
3	−1	11	1.12659	2.79949	13
−3	−1	−11	2.98069	1.84106	8
−3	−1	−11	3.47554	2.13923	10
−3	1	−11	−0.8090	2.34107	12
−3	1	−11	0.94035	1.63022	8
−3	1	−11	−2.4177	2.38568	14
3	1	11	1.26529	2.97478	13
−4	−1	−11	7.94367	2.45589	12
−4	−1	−11	9.64741	2.03658	8
4	−1	11	13.2087	3.81752	13
−4	1	−11	13.8983	1.52051	11
−4	1	−11	13.7413	2.72808	12
−4	1	−11	15.2693	2.28511	8
4	1	11	8.52005	3.35944	13
5	−1	11	−0.4085	2.40528	13
−5	−1	−11	0.80184	1.42890	8
−5	−1	−11	−0.6387	1.79388	12
−5	1	−11	0.89128	1.86182	12
−5	1	−11	1.02617	1.12774	8
5	1	11	2.38260	2.66714	13
6	1	11	10.1868	2.25310	13
−9	−2	11	1.47126	1.70183	10
9	−2	−11	3.02695	2.26766	13
9	−2	−11	1.79703	2.32587	13
−9	2	11	0.60597	0.86175	11
8	−2	−11	17.2356	3.61596	13

−8	−2	11	21.4981	3.91996	10
−8	2	11	17.2669	3.46446	10
8	2	−11	21.4823	4.09433	13
8	2	−11	21.6962	3.93723	13
−7	−2	11	35.7730	5.11487	10
7	−2	−11	31.6300	5.66279	13
7	2	−11	38.3559	5.30798	13
−7	2	11	24.5619	4.63671	10
−6	−2	11	16.4794	3.57398	10
6	−2	−11	9.26781	3.23679	13
−6	2	11	12.8763	3.25038	10
5	−2	−11	20.5457	4.13640	13
−5	−2	11	28.5996	4.69084	10
−5	2	11	15.3191	3.71218	10
4	−2	−11	28.3973	4.68653	13
−4	2	11	34.1138	5.58438	10
4	2	−11	39.2073	5.30714	10
3	2	−11	9.76222	3.17207	10
2	−2	−11	29.8940	5.06322	10
1	−2	−11	18.2249	4.03065	10
1	2	−11	17.1705	3.05218	8
1	2	−11	18.0166	3.63340	10
0	−2	−11	29.4472	4.68841	10
0	−2	−11	29.4135	4.12306	8
0	2	11	27.2094	4.71892	13
−1	−2	−11	39.2746	5.44568	10
1	−2	11	46.4217	5.71426	13
−1	−2	−11	36.1329	4.06222	8
−1	−2	−11	37.5876	5.80208	1
1	2	11	33.5578	6.35465	13
−1	2	−11	37.0135	4.82805	10
−1	2	−11	35.6720	3.83425	8
2	−2	11	9.25882	3.14139	13
−2	−2	−11	9.63479	2.57376	8
−2	2	−11	10.1127	2.15038	8
2	2	11	7.95635	3.76982	13
−2	2	−11	8.97859	2.09730	10
−3	−2	−11	16.6295	3.26566	10
3	−2	11	12.5306	3.39093	13
−3	−2	−11	13.1419	2.46329	8
−3	2	−11	18.8472	2.97595	8
−3	2	−11	21.8739	3.33208	12
3	2	11	9.44950	3.65361	13
4	−2	11	5.39955	2.89901	13
−4	−2	−11	5.46448	1.83562	8
−4	−2	−11	2.26530	2.14911	12
4	2	11	4.73971	3.21655	13
−4	2	−11	8.43316	1.02504	11
−4	2	−11	6.80336	2.49657	12
−4	2	−11	6.73819	1.56034	8

5	−2	11	7.24390	3.11067	13
−5	−2	−11	4.33561	1.91249	12
−5	−2	−11	5.82294	1.47609	8
5	2	11	9.48346	2.90994	13
−5	2	−11	6.71168	1.25948	8
−5	2	−11	7.37528	2.14105	12
9	−3	−11	10.3806	2.69251	13
−9	−3	11	15.3493	2.66720	10
−9	3	11	8.93230	1.35434	11
−8	−3	11	1.49832	2.37995	10
8	−3	−11	5.11341	3.06197	13
−7	−3	11	12.7536	3.54777	10
7	−3	−11	13.7247	4.22074	13
−7	3	11	7.99791	2.97324	10
−6	−3	11	31.0689	5.28164	10
6	−3	−11	44.8961	5.88305	13
−6	3	11	44.9337	5.31229	10
5	−3	−11	71.4444	7.68213	13
−5	−3	11	77.6603	7.63096	10
−5	3	11	82.6939	7.65472	10
4	−3	−11	1.20974	2.97339	13
−4	3	11	1.42660	2.93136	10
4	3	−11	4.89453	2.94467	10
−3	3	11	15.6402	4.04764	10
3	3	−11	8.10770	3.10820	10
2	−3	−11	112.443	9.24013	10
2	3	−11	114.002	9.12769	10
1	−3	−11	68.1068	7.11929	10
1	3	−11	85.6202	6.97332	10
1	3	−11	81.3634	6.30139	8
0	−3	11	14.5294	4.02140	13
0	3	−11	12.4319	2.69314	10
0	3	−11	14.8155	2.58809	8
−1	−3	−11	5.46650	3.27721	1
−1	−3	−11	4.03867	2.49917	10
−1	−3	−11	3.18657	1.92233	8
1	−3	11	3.61002	3.44091	13
−1	3	−11	3.72756	1.72415	8
1	3	11	4.04287	3.03943	13
−1	3	−11	3.92595	1.93354	10
−2	−3	−11	96.0341	7.96242	10
−2	−3	−11	90.4238	8.67152	1
−2	−3	−11	87.4970	7.31487	8
2	−3	11	104.466	8.96095	13
−2	3	−11	98.1764	7.89839	14
−2	3	−11	105.483	7.88575	12
−2	3	−11	95.6130	7.14524	10
2	3	11	89.6211	9.09369	13
−2	3	−11	104.773	7.17135	8
−3	−3	−11	54.2331	5.43274	10

3	−3	11	59.7377	6.39939	13
−3	−3	−11	50.9117	4.98608	8
−3	3	−11	50.6015	5.59715	12
−3	3	−11	54.9133	4.86474	8
−3	3	−11	56.9767	5.85986	14
−4	−3	−11	11.1713	2.20560	8
−4	−3	−11	12.2847	2.39424	10
4	−3	11	12.2995	3.11265	13
−4	−3	−11	13.1003	2.57274	12
−4	3	−11	9.40850	2.67549	12
4	3	11	14.0010	3.69352	13
−4	3	−11	9.89857	1.82698	8
5	−3	11	2.23021	2.20595	13
−5	3	−11	4.59804	1.95056	12
−9	−4	11	0.25323	1.22563	10
9	−4	−11	1.89815	2.32840	13
−9	4	11	1.19768	0.77439	11
8	−4	−11	9.65343	3.19846	13
−8	4	11	9.06818	2.27469	10
−7	−4	11	44.6051	5.04690	10
7	−4	−11	33.4075	5.94247	13
−7	4	11	39.7719	5.09114	10
−6	−4	11	13.2580	3.50074	10
6	−4	−11	13.1886	3.30902	13
−6	4	11	7.60241	2.79654	10
5	−4	−11	77.6283	7.17531	13
−5	−4	11	75.5394	10.5202	1
−5	4	11	78.1533	7.59402	10
−4	−4	11	19.8282	6.90009	1
−4	4	11	7.72198	3.51665	10
4	4	−11	16.1197	3.81799	10
−3	−4	11	7.63041	3.58557	13
3	4	−11	6.58363	2.79359	10
2	−4	−11	42.2680	5.32406	10
2	4	−11	30.1825	4.70881	10
−1	−4	11	99.9184	8.75340	13
1	−4	−11	80.3978	8.52806	10
1	4	−11	96.9393	8.14694	10
1	4	−11	95.4820	7.13232	8
0	−4	−11	2.84815	3.29270	1
0	−4	−11	9.44822	3.39628	10
0	4	−11	10.1341	3.10685	1
−1	−4	−11	46.4222	5.30060	10
1	−4	11	41.4441	6.70344	13
−1	−4	−11	52.1144	5.14773	8
−1	−4	−11	47.1358	7.41744	1
−1	4	−11	39.8995	4.11225	8
1	4	11	49.8622	6.92583	13
−1	4	−11	38.7843	4.59817	10
2	−4	11	103.162	9.53271	13

−2	−4	−11	119.950	8.33297	8
−2	−4	−11	111.328	10.0558	1
−2	−4	−11	120.350	8.96858	10
2	4	11	108.022	9.95420	13
−2	4	−11	100.756	8.56901	14
−2	4	−11	109.551	8.00807	8
−2	4	−11	107.037	8.84112	12
3	−4	11	62.0808	6.34613	13
−3	−4	−11	45.5529	5.28926	10
−3	−4	−11	53.5036	5.01810	8
−3	4	−11	58.0594	5.93014	14
−3	4	−11	38.6067	5.53573	12
−3	4	−11	54.1022	3.88897	11
3	4	11	60.2412	7.24579	13
−3	4	−11	48.3400	4.58367	8
4	−4	11	8.63556	2.76641	13
−4	−4	−11	8.47079	2.30326	12
−4	−4	−11	9.32995	2.17492	8
−4	−4	−11	8.72843	2.08660	10
4	4	11	10.4468	4.31718	13
−4	4	−11	10.7024	2.62753	12
−4	4	−11	7.78168	1.53775	8
−5	−4	−11	3.87539	1.45699	8
5	4	11	3.22950	2.66770	13
−9	−5	11	6.17313	1.97763	10
9	−5	−11	4.81691	2.85972	13
−9	5	11	4.03888	0.59362	11
8	−5	−11	21.6045	3.57870	13
−8	−5	11	13.5347	3.30923	10
−8	5	11	19.1331	3.16507	10
−7	−5	11	70.5197	6.26422	10
7	−5	−11	54.9407	6.82452	13
−7	5	11	58.0873	5.96791	10
−6	−5	11	4.04979	2.85616	10
−6	−5	11	3.09380	4.22493	1
6	−5	−11	8.86836	3.17074	13
−6	5	11	7.99424	2.89873	10
5	−5	−11	37.6291	5.89643	13
−5	−5	11	39.1721	8.26681	1
−5	5	11	41.0765	5.57365	10
5	5	−11	40.4919	5.88620	10
−4	−5	11	14.5249	3.90072	13
4	−5	−11	18.8088	3.60872	13
4	5	−11	15.4666	3.56283	10
−4	5	11	19.7392	4.02788	10
3	−5	−11	100.566	7.40960	13
−3	−5	11	68.9472	7.86577	13
3	5	−11	77.6805	7.73914	10
−3	5	11	96.3305	8.76562	10
−2	−5	11	5.42242	3.53928	13

2	5	−11	5.98642	2.54565	10
1	−5	−11	80.3225	7.99304	10
−1	−5	11	100.617	8.21631	13
1	5	−11	90.3613	7.82429	10
0	−5	11	30.4118	4.87016	13
0	5	−11	29.4836	3.48122	8
0	5	−11	30.3822	4.04877	10
1	−5	11	21.0687	3.80660	13
−1	5	−11	22.2893	3.09818	8
−1	5	−11	22.1460	3.55242	10
−1	5	−11	23.3810	3.72873	12
1	5	11	35.0840	5.58341	13
−1	5	−11	24.5939	3.91004	14
2	−5	11	17.5494	3.56245	13
−2	−5	−11	24.5675	3.64275	8
−2	−5	−11	25.7388	4.26158	10
−2	−5	−11	26.2243	5.51072	1
−2	5	−11	18.3471	3.18201	14
−2	5	−11	18.5802	3.15881	8
−2	5	−11	21.9268	3.64273	12
2	5	11	20.9310	4.00701	13
−2	5	−11	20.5868	2.93278	11
−3	−5	−11	18.4971	2.82213	8
−3	−5	−11	15.6627	3.05787	10
−3	5	−11	14.6941	2.37531	8
−3	5	−11	11.8842	1.72562	11
−3	5	−11	17.2560	3.32508	14
−3	5	−11	14.6573	1.84365	11
3	5	11	28.8754	5.22025	13
−3	5	−11	18.3987	3.34941	12
−4	−5	−11	3.61030	2.06262	12
−4	−5	−11	6.54559	1.65173	8
−4	−5	−11	3.53687	1.90037	10
−4	5	−11	2.93010	2.31639	12
4	5	11	7.45813	3.56012	13
−5	−5	−11	43.4718	3.75893	8
−5	5	−11	40.5818	4.40506	12
5	5	11	46.3514	5.39658	13
−9	−6	11	20.6144	3.04687	10
9	−6	−11	20.1475	3.79460	13
−8	−6	11	3.29682	2.60454	10
−8	6	11	4.64776	2.10414	10
−8	6	11	0.49123	1.16554	11
−7	−6	11	10.5834	3.34213	10
7	−6	−11	13.8640	3.55192	13
−7	6	11	15.0815	2.97456	10
−6	−6	11	53.8056	8.65699	1
−6	−6	11	40.9128	6.03661	10
−6	6	11	42.3145	5.49376	10
5	−6	−11	0.96642	2.50862	13

−5	−6	11	4.18344	4.19072	1
−5	6	11	2.27530	2.70254	10
5	6	−11	3.80054	3.02589	10
4	−6	−11	11.1870	2.82801	13
−4	−6	11	13.6622	3.27737	13
4	6	−11	10.2704	3.23023	10
−4	6	11	15.5188	3.85941	10
−3	−6	11	41.2052	6.43283	13
3	−6	−11	34.7917	4.76326	13
3	6	−11	28.2992	5.10068	10
−3	6	11	36.6539	5.86729	10
−2	−6	11	47.7448	6.81153	13
−2	6	11	33.1270	6.16015	10
1	−6	−11	103.770	8.93691	10
−1	−6	11	101.089	8.96344	13
1	6	−11	96.5438	8.06209	10
1	6	−11	101.047	7.98129	14
0	−6	−11	29.6581	4.99371	10
0	−6	−11	33.5336	6.61592	1
0	−6	11	25.4787	4.12372	13
0	6	−11	23.6484	4.16292	10
0	6	−11	20.3520	3.50179	14
0	6	11	34.1232	6.32177	13
−1	−6	−11	29.9390	5.91611	1
−1	−6	−11	29.7636	4.82460	10
−1	6	−11	30.1493	3.67074	10
1	6	11	27.9768	4.50135	13
−1	6	−11	29.2996	3.33310	8
−1	6	−11	21.3244	3.53455	14
−1	6	−11	23.7488	4.00808	12
−2	−6	−11	145.908	12.6296	1
−2	−6	−11	143.643	10.9498	10
2	−6	11	149.147	12.0792	13
−2	−6	−11	144.105	10.2792	8
−2	6	−11	141.636	10.4629	14
2	6	11	141.622	11.3341	13
−2	6	−11	138.919	9.67963	8
−2	6	−11	148.071	10.7688	12
−2	6	−11	141.977	9.33990	11
−3	−6	−11	11.4157	2.58387	8
−3	−6	−11	11.8578	2.96077	10
3	6	11	11.3775	3.85628	13
−3	6	−11	15.0006	3.62414	14
−3	6	−11	14.7460	2.98452	12
−4	−6	−11	16.5176	2.88956	12
−4	−6	−11	21.6103	2.95266	8
−4	−6	−11	17.5692	3.39591	10
−4	6	−11	24.4107	4.01776	12
−5	−6	−11	11.9768	1.88601	8
−5	6	−11	6.28497	2.19057	12

5	6	11	9.24015	2.88440	13
−5	6	−11	10.3133	1.95014	9
8	−7	−11	25.3466	3.71442	13
−8	−7	11	21.0975	4.06662	10
−8	7	11	22.1186	2.21603	11
7	−7	−11	10.2655	3.37016	13
−7	−7	11	8.79219	3.18504	10
−7	7	11	14.1611	2.72181	10
6	−7	−11	7.59552	3.88443	13
−6	−7	11	16.0594	6.92869	1
−6	7	11	5.33303	2.81713	10
5	−7	−11	4.88141	3.23254	13
−5	−7	11	7.24046	4.67258	1
−5	7	11	6.06843	3.00164	10
5	7	−11	2.99190	3.05376	10
−4	−7	11	7.02506	3.44195	13
4	−7	−11	−0.4555	3.00763	13
−4	7	11	−1.7807	2.77076	10
4	7	−11	7.26857	3.19607	10
3	−7	−11	48.9068	6.23756	13
−3	−7	11	33.7717	6.47681	13
−3	7	11	48.5226	6.29843	10
−2	−7	11	70.1811	6.95986	13
2	7	−11	68.3056	6.46873	10
−2	7	11	68.5738	7.74761	10
−1	−7	11	118.077	9.61425	13
1	7	−11	109.588	8.44694	10
1	7	−11	97.9812	8.37532	14
0	−7	−11	115.440	11.8679	1
0	−7	−11	109.878	9.50147	10
0	−7	11	109.841	9.55124	13
0	7	−11	121.340	8.77234	10
0	7	11	116.487	9.99449	13
0	7	−11	119.076	8.84635	14
−1	−7	−11	94.2389	8.30509	10
−1	−7	−11	86.1113	10.1489	1
1	−7	11	93.9449	8.75949	13
−1	7	−11	98.8077	6.82370	11
−1	7	−11	100.356	7.72885	14
−1	7	−11	102.129	7.84468	12
1	7	11	84.0818	8.53607	13
−2	−7	−11	160.217	11.5637	10
−2	−7	−11	155.703	13.1139	1
−2	7	−11	158.174	9.95479	11
−2	7	−11	152.135	10.9855	12
2	7	11	148.830	11.8070	13
−2	7	−11	147.225	9.69077	11
−2	7	−11	143.065	10.9686	14
−3	−7	−11	−1.4113	2.31222	10
−3	−7	−11	1.98620	2.06417	8

3	7	11	-1.4790	3.77483	13
-3	7	-11	0.99768	2.23381	12
-3	7	-11	3.03122	2.57859	14
-4	-7	-11	8.98805	3.76459	12
4	7	11	7.06966	3.36983	13
-4	7	-11	3.97302	2.33721	12
-5	-7	-11	2.82092	1.52131	8
-5	7	-11	1.72910	1.63835	9
5	7	11	-0.1307	2.37086	13
-5	7	-11	2.69746	1.87691	12
8	-8	-11	25.0907	4.67647	13
-8	-8	11	36.0999	3.90509	10
-8	8	11	28.7710	2.37932	11
7	-8	-11	81.9770	7.84942	13
-7	-8	11	80.8384	7.22788	10
-7	8	11	81.0461	6.86431	10
6	-8	-11	5.07227	3.73677	13
6	8	-11	2.99536	3.05940	10
-6	8	11	1.22434	2.37463	10
5	-8	-11	4.85283	3.33049	13
-5	8	11	1.36161	2.83764	10
5	8	-11	3.44249	2.97321	10
-4	-8	11	9.03876	3.02790	13
4	-8	-11	8.93106	3.42322	13
4	8	-11	11.2737	2.80755	10
-4	8	11	9.68684	3.35617	10
-3	-8	11	42.7481	6.32077	13
3	-8	-11	31.5459	4.88011	13
-3	8	11	31.4500	5.51834	10
3	8	-11	19.7424	3.86952	10
-2	-8	11	24.0139	4.43310	13
2	8	-11	22.9179	3.90697	10
-1	-8	11	263.133	18.4468	13
1	8	-11	270.260	17.7785	14
1	8	-11	274.183	17.9454	10
0	-8	-11	44.2393	7.51054	1
0	-8	11	28.5546	5.19392	13
0	8	-11	28.8452	4.23629	14
0	8	-11	25.1726	3.87788	10
1	-8	11	121.682	10.1884	13
-1	-8	-11	157.613	13.7869	1
-1	-8	-11	148.490	10.7181	10
-1	8	-11	126.226	9.66877	12
-1	8	-11	124.137	10.1358	14
-1	8	-11	124.907	8.78007	11
1	8	11	138.993	10.6781	13
-2	-8	-11	28.8535	5.89993	1
-2	-8	-11	23.6306	4.65407	10
-2	8	-11	28.8179	2.28464	11
-2	8	-11	27.7535	2.28008	11

−2	8	−11	26.6275	4.18748	12
2	8	11	24.0444	4.36070	13
−2	8	−11	33.1105	4.29102	14
−3	−8	−11	13.9701	2.80120	8
−3	−8	−11	12.0652	3.27407	10
−3	8	−11	17.9273	3.39903	14
−3	8	−11	17.7938	3.04597	12
3	8	11	12.2731	3.80841	13
−4	−8	−11	48.5535	4.47175	8
−4	−8	−11	44.9706	4.49692	10
4	8	11	41.2599	5.83496	13
−4	8	−11	37.2258	4.13806	12
5	8	11	8.10050	2.46143	13
−5	8	−11	7.00444	2.01339	12
8	−9	−11	29.5154	4.44532	13
−8	9	11	27.0133	2.07608	11
−8	9	11	28.6982	2.11582	11
7	−9	−11	55.8504	6.45805	13
−7	−9	11	55.6316	6.19989	10
−7	9	11	58.2835	4.90626	10
6	−9	−11	10.2889	3.23104	13
6	9	−11	3.14200	2.24070	10
−6	9	11	6.71531	2.55584	10
−5	9	11	30.0982	4.91022	10
5	9	−11	20.1548	4.03803	10
4	−9	−11	24.7195	4.20289	13
−4	−9	11	14.2800	3.34198	13
−4	9	11	22.7795	4.25280	10
4	9	−11	13.4853	3.58350	10
3	−9	−11	42.5773	5.70775	13
−3	−9	11	49.3136	6.00406	13
−3	9	11	40.5436	6.16521	10
3	9	−11	49.3388	5.64050	10
−2	−9	11	46.8277	5.79866	13
2	−9	−11	29.8304	4.57206	13
−2	9	11	32.2318	5.85905	10
1	−9	−11	3.27334	4.04053	1
−1	−9	11	2.08089	3.30782	13
1	9	−11	8.21084	2.94816	14
1	9	−11	2.71358	2.51340	10
−1	9	11	3.77091	3.83099	10
0	−9	11	42.7911	6.42924	13
0	9	−11	35.4990	4.75513	14
0	9	−11	39.1061	4.35004	10
−1	9	−11	8.66462	1.56131	11
−1	9	−11	6.90092	2.37508	12
−1	9	−11	7.90769	2.60339	14
−2	−9	−11	31.5007	6.27815	1
−2	−9	−11	41.8032	5.34498	10
2	9	11	28.9289	5.28782	13

-2	9	-11	29.5487	4.05794	12
-2	9	-11	32.7532	5.17215	14
-3	-9	-11	31.9186	3.96462	10
3	9	11	30.8741	5.52210	13
-3	9	-11	38.4180	5.13250	14
-3	9	-11	28.7898	4.31502	12
-4	-9	-11	10.5501	1.91689	8
-4	-9	-11	9.82756	2.46102	10
4	9	11	12.8186	3.07632	13
-4	9	-11	8.35973	2.98804	14
-4	9	-11	8.64465	2.05590	12
-4	9	-11	7.81268	0.92223	6
-4	9	-11	8.57184	1.27811	9
5	9	11	0.98591	2.00190	13
-5	9	-11	2.65921	1.75204	12
8	-10	-11	2.87201	2.28745	13
7	-10	-11	17.8888	3.51356	13
-7	10	11	20.7447	2.33554	11
6	10	-11	10.6482	3.46941	10
5	-10	-11	-2.8557	3.61853	13
5	10	-11	0.53469	3.16710	10
-5	10	11	0.94167	2.63844	10
4	-10	-11	10.9309	3.26684	13
-4	-10	11	11.2443	3.63965	13
-4	10	11	14.8283	3.71820	10
4	10	-11	9.87062	3.23549	10
3	-10	-11	39.1795	6.31877	13
-3	-10	11	37.2040	6.44977	13
3	10	-11	40.0257	5.22472	10
-3	10	11	43.9208	6.11806	10
-2	-10	11	145.675	10.7810	13
2	-10	-11	129.969	10.4408	13
2	10	-11	141.167	10.4325	10
-2	10	11	138.416	11.5534	10
1	-10	-11	205.739	18.0043	1
-1	-10	11	206.951	14.7874	13
1	10	-11	217.205	14.3267	10
1	10	-11	214.796	14.5759	14
-1	10	11	204.050	15.8303	10
0	-10	11	46.1706	5.81071	13
0	10	-11	50.3050	6.02995	14
0	10	-11	52.6989	4.28885	11
-1	-10	-11	36.5693	5.72229	10
-1	-10	-11	36.6347	7.28715	1
-1	10	-11	53.6427	5.59283	14
-1	10	-11	54.5531	4.26771	11
-1	10	-11	49.7573	3.77546	11
-1	10	-11	51.5887	4.90050	12
-2	-10	-11	188.023	13.0403	10
-2	10	-11	176.310	12.6090	14

−2	10	−11	164.684	12.2367	12
−3	−10	−11	10.5904	3.04600	10
−3	10	−11	8.35182	2.16034	12
−3	10	−11	11.1055	2.98473	14
3	10	11	10.4132	3.83078	13
−4	−10	−11	3.04324	1.89629	8
−4	−10	−11	2.23432	2.08349	10
−4	10	−11	6.41157	2.33360	14
−4	10	−11	4.97609	1.76653	12
−4	10	−11	3.66569	0.97525	9
−4	10	−11	5.23516	0.63695	6
4	10	11	0.10766	3.06571	13
−5	10	−11	12.4435	2.16201	12
−8	−11	11	35.7413	4.50912	10
8	−11	−11	37.9419	4.31902	13
7	−11	−11	32.2619	4.95580	13
7	11	−11	31.3758	4.33642	10
−7	11	11	32.0539	2.83781	11
6	−11	−11	8.99585	3.30991	13
−6	11	11	8.88101	2.62318	10
6	11	−11	15.4100	3.71690	10
5	−11	−11	13.4367	3.59109	13
5	11	−11	11.1901	2.85895	10
−5	11	11	10.6058	3.12722	10
4	−11	−11	16.8609	4.02952	13
−4	−11	11	8.54697	3.02893	13
4	11	−11	7.00234	3.11653	10
−4	11	11	13.6862	3.63690	10
−3	−11	11	29.1131	5.39455	13
3	−11	−11	37.5785	5.41639	13
3	11	−11	30.7740	4.76917	10
−3	11	11	33.6192	5.70292	10
2	−11	−11	37.2035	6.01399	13
−2	−11	11	40.9575	5.57875	13
2	11	−11	45.2754	4.85463	10
−2	11	11	34.9310	6.07126	10
−1	−11	11	194.523	14.0876	13
1	−11	−11	185.448	18.4254	1
−1	11	11	166.380	14.5919	10
1	11	−11	206.099	13.2977	10
1	11	−11	203.270	13.4546	14
0	−11	11	218.034	15.9881	13
0	11	−11	227.179	15.7952	14
0	11	−11	252.609	14.9361	11
−1	−11	−11	81.9351	10.6310	1
−1	11	−11	89.1032	8.16547	14
−1	11	−11	103.906	6.30842	11
−1	11	−11	100.853	6.27185	11
−2	11	−11	15.0424	2.91205	12
−2	11	−11	13.3673	3.16873	14

-3	11	-11	20.0261	3.35977	12
-3	11	-11	23.3811	3.67940	14
-3	11	-11	24.2898	2.49115	9
-4	-11	-11	3.27315	1.61304	10
-4	11	-11	1.14973	2.03355	14
-4	11	-11	1.33140	1.95517	12
-4	11	-11	1.27485	0.96622	9
8	-12	-11	9.25484	2.23113	13
7	-12	-11	48.7005	5.24221	13
7	12	-11	47.1121	4.98832	10
-7	12	11	47.5325	3.34199	11
-7	12	11	49.9386	3.40408	11
6	-12	-11	8.98228	3.31819	13
-6	12	11	5.42904	2.22948	10
5	-12	-11	33.0019	5.71841	13
-5	12	11	35.1386	4.79042	10
-4	-12	11	4.14640	2.97825	13
4	-12	-11	2.20344	3.79725	13
4	12	-11	3.34649	2.79251	10
-4	12	11	2.31264	2.94568	10
-3	-12	11	14.3575	3.42575	13
3	-12	-11	14.4197	3.27465	13
3	12	-11	7.78134	2.86182	10
-3	12	11	10.5284	3.78970	10
2	-12	-11	18.5621	3.50198	13
-2	12	11	24.7831	4.64342	10
-1	-12	11	79.6059	7.38103	13
1	12	-11	79.8582	7.22413	14
1	12	-11	75.6948	6.40545	10
0	-12	11	34.2363	5.07204	13
0	12	-11	36.0828	3.64316	11
0	12	11	53.7636	6.82498	10
0	12	-11	48.4698	5.58772	14
-1	-12	-11	113.662	8.87593	10
-1	12	-11	98.5760	8.74486	14
-2	-12	-11	4.95721	2.92099	10
-2	12	-11	4.46097	2.74564	12
-2	12	-11	2.95134	2.54818	14
-3	-12	-11	11.8090	3.00675	10
-3	12	-11	12.0004	1.13883	6
-3	12	-11	15.2467	3.04493	14
-3	12	-11	11.0439	1.45936	9
-3	12	-11	12.8147	2.70516	12
-4	-12	-11	7.56825	2.26215	10
-4	12	-11	13.5821	2.58903	14
-4	12	-11	8.82200	2.19412	12
-4	12	-11	6.81840	0.94858	9
7	-13	-11	10.3605	2.52038	13
7	13	-11	4.72937	2.18951	10
6	-13	-11	27.4860	4.79160	13

−6	13	11	26.1746	2.71134	11
5	−13	−11	106.791	8.21990	13
−5	−13	11	73.2087	7.22880	13
−5	13	11	105.927	7.58891	10
5	13	−11	74.4654	7.27688	10
−4	−13	11	26.8880	4.95330	13
4	−13	−11	31.4005	5.45033	13
−4	13	11	26.5223	4.75640	10
4	13	−11	23.2059	4.02660	10
−3	−13	11	11.3584	3.51597	13
3	13	−11	11.9338	3.10402	10
2	−13	−11	17.6852	3.49170	13
−2	−13	11	28.4930	4.91800	13
−2	13	11	16.1400	4.36800	10
−1	−13	11	14.3237	3.27066	13
1	13	−11	16.2070	3.30315	14
1	13	−11	16.2951	2.39307	11
−1	13	11	12.7726	4.36296	10
0	−13	−11	40.9176	8.29827	1
0	−13	11	45.5824	6.56462	13
0	13	−11	64.3776	7.05831	14
0	13	−11	54.8198	4.57934	11
0	13	−11	53.5296	4.17413	11
0	13	11	49.7044	7.35860	10
−1	−13	−11	57.0245	6.72237	10
−1	13	−11	74.3481	6.66466	14
−2	−13	−11	0.58266	2.72468	10
−3	−13	−11	9.50521	2.86397	10
−3	13	−11	3.92042	2.55043	14
−3	13	−11	5.52778	2.25043	12
−3	13	−11	4.10949	0.74969	6
−3	13	−11	4.70585	1.04472	9
−4	−13	−11	12.4161	2.39997	10
−4	13	−11	12.8771	2.25322	12
−4	13	−11	10.8433	2.12998	14
7	−14	−11	9.17677	2.31381	13
7	14	−11	7.03401	2.76073	10
6	−14	−11	17.0177	3.31826	13
−6	14	11	18.2307	2.06421	11
6	14	−11	21.4170	3.66379	10
−5	−14	11	4.96164	2.91178	13
5	−14	−11	1.02122	3.75192	13
−5	14	11	2.27089	2.03608	10
5	14	−11	10.7548	3.20015	10
4	−14	−11	40.6665	5.51464	13
−4	14	11	39.9104	5.25807	10
4	14	−11	38.9329	5.21427	10
3	−14	−11	1.17375	3.59086	13
−3	−14	11	2.87238	3.41276	13
3	14	−11	−0.0226	2.33748	10

−3	14	11	2.48473	2.97163	10
−2	−14	11	28.1588	5.31558	13
2	−14	−11	23.8038	3.92063	13
2	14	−11	25.0924	3.71239	10
−2	14	11	17.9848	4.47123	10
−1	−14	11	48.5545	5.71895	13
1	14	−11	48.3842	4.37074	11
1	14	−11	48.2051	5.79316	14
0	−14	11	53.5155	5.74763	13
0	14	−11	54.5160	3.77019	11
0	14	−11	56.0124	3.84730	11
0	14	−11	55.1693	6.03067	14
−1	−14	−11	14.7797	3.07293	10
1	14	11	11.9519	3.33824	10
−1	14	−11	9.04867	2.96384	14
−2	14	−11	18.9533	2.97397	12
−2	14	−11	20.2966	3.35483	14
−3	−14	−11	0.06769	2.30179	10
−3	14	−11	3.72371	1.88836	12
−3	14	−11	2.17024	0.94366	9
−3	14	−11	2.36180	0.63071	6
−3	14	−11	3.97755	2.25614	14
−4	−14	−11	8.37841	2.11999	10
−4	14	−11	8.69495	1.97164	12
7	−15	−11	2.98693	1.91398	13
7	15	−11	1.86836	2.74808	10
6	−15	−11	1.39868	2.18922	13
−6	15	11	0.74991	0.71534	11
−6	15	11	0.08557	0.78974	11
6	15	−11	0.35804	2.65546	10
−5	−15	11	20.2375	3.44729	13
5	−15	−11	24.6910	4.30671	13
5	15	−11	19.7374	3.47236	10
−5	15	11	17.4506	3.16152	10
−4	−15	11	6.63218	2.71937	13
4	−15	−11	13.0333	3.42989	13
4	15	−11	5.29560	2.70945	10
−4	15	11	11.5549	2.94649	10
3	−15	−11	2.05469	3.10871	13
−3	−15	11	2.73809	2.93177	13
−3	15	11	3.95092	2.94055	10
3	15	−11	−0.2477	2.33786	10
2	−15	−11	16.0352	3.95592	13
−2	−15	11	4.66501	3.44996	13
2	15	−11	6.80380	2.10137	10
−2	15	11	14.2860	3.77048	10
−1	−15	11	116.415	9.63746	13
−1	15	11	113.308	10.2682	10
0	−15	11	19.7309	3.32725	13
0	15	−11	31.2900	4.91287	14

0	15	11	25.6384	4.88640	10
−1	15	−11	44.3490	5.61835	14
−2	−15	−11	0.67741	1.91257	10
−2	15	−11	1.00124	0.54919	6
−2	15	−11	1.53750	0.98836	9
−2	15	−11	0.70016	2.43948	14
−3	−15	−11	12.4512	2.75826	10
−3	15	−11	10.9505	2.43610	12
−3	15	−11	11.4446	1.32385	9
−3	15	−11	12.1342	2.54914	14
6	−16	−11	0.85876	2.40808	13
6	16	−11	−0.3045	2.38046	10
5	−16	−11	9.68908	3.02524	13
−5	−16	11	18.0953	3.38739	13
5	16	−11	20.7518	3.55922	10
−5	16	11	14.0584	2.13075	11
−4	−16	11	−1.6626	2.36735	13
4	−16	−11	−1.2281	2.78377	13
4	16	−11	0.78701	2.45569	10
−4	16	11	0.04897	2.15994	10
−3	−16	11	11.6751	3.16607	13
3	−16	−11	12.5557	3.67047	13
3	16	−11	14.3634	2.73351	10
−3	16	11	10.8119	3.09990	10
2	−16	−11	8.28664	2.92787	13
−2	−16	11	2.71300	2.57478	13
2	16	−11	6.20755	1.87705	11
−2	16	11	9.64692	3.41215	10
−1	−16	11	−1.8939	2.24325	13
−1	16	11	2.19808	3.44469	10
1	16	−11	0.59087	1.16053	11
1	16	−11	1.00578	1.19394	11
1	16	−11	3.61265	2.68204	14
0	−16	11	16.6351	3.01465	13
0	16	−11	12.8866	3.28196	14
1	16	11	70.6141	7.32558	10
−1	16	−11	60.3597	6.21647	14
−2	−16	−11	1.43380	1.83548	10
−2	16	−11	0.21296	2.30590	14
−2	16	−11	−0.0534	0.82368	9
−3	−16	−11	8.65676	2.38255	10
−3	16	−11	3.36999	1.57696	12
−3	16	−11	4.57298	2.04601	14
6	−17	−11	0.17000	1.76509	13
6	17	−11	1.69089	2.17453	10
5	−17	−11	19.3787	3.18821	13
−5	−17	11	25.3900	3.91220	13
5	17	−11	22.2389	3.08079	10
−5	17	11	20.1785	2.12379	11
−4	−17	11	2.93712	2.31212	13

4	−17	−11	4.84743	2.77238	13
−4	17	11	6.17654	2.37479	10
4	17	−11	3.41979	2.32328	10
−3	−17	11	18.8694	3.23670	13
3	−17	−11	15.7919	3.26967	13
−3	17	11	14.9085	3.20829	10
3	17	−11	17.9663	3.04960	10
−2	−17	11	7.89541	3.05657	13
2	−17	−11	14.6059	3.30793	13
2	17	−11	15.3329	2.05082	11
−2	17	11	17.2216	3.84135	10
−1	−17	11	−0.3884	2.34276	13
−1	17	11	−2.0945	2.22577	10
0	−17	11	6.00570	2.71856	13
0	17	−11	7.00975	2.84116	14
0	17	11	7.64013	2.73795	10
1	17	11	14.7022	3.14558	10
−1	17	−11	13.8832	3.04919	14
−2	−17	−11	2.91208	1.69927	10
−2	17	−11	1.68515	0.92448	9
−2	17	−11	1.22312	2.11747	14
2	17	11	5.11085	2.97097	10
−3	17	−11	2.58391	2.15173	14
5	−18	−11	13.2512	2.43219	13
5	18	−11	11.0953	2.34590	10
−5	18	11	9.07879	0.74540	11
−5	18	11	9.32339	0.72345	11
−4	−18	11	8.99896	2.37589	13
4	−18	−11	8.83569	2.72404	13
4	18	−11	8.67953	2.47306	10
−4	18	11	12.6554	1.58836	11
−3	−18	11	10.0917	2.63971	13
3	−18	−11	9.91886	2.88266	13
3	18	−11	7.67227	1.86084	11
−2	−18	11	10.0728	2.71712	13
2	18	−11	10.0759	1.51407	11
−2	18	11	9.71883	2.58306	10
2	18	−11	9.49567	1.61174	11
−1	−18	11	2.01589	2.11064	13
1	18	−11	3.29196	2.33043	14
−1	18	11	1.55951	2.11116	10
0	−18	11	4.29917	2.12922	13
0	18	11	14.0710	3.75668	10
0	18	−11	6.77346	2.64087	14
−1	18	−11	28.2047	4.15281	14
1	18	11	28.2785	4.78957	10
−2	18	−11	3.52575	2.04818	14
5	−19	−11	7.08404	2.32792	13
−4	−19	11	0.09676	2.05556	13
4	−19	−11	0.22481	1.90240	13

4	19	-11	-0.3423	1.49252	11
-4	19	11	0.79287	0.91745	11
-3	-19	11	2.97015	2.08298	13
3	-19	-11	1.26416	2.18771	13
3	19	-11	1.98716	1.45581	11
-3	19	11	1.67608	1.74109	10
2	-19	-11	2.60299	2.54677	13
-2	-19	11	-0.1302	1.95827	13
-2	19	11	1.29713	2.15496	10
-1	-19	11	12.7136	2.43465	13
-1	19	11	10.0963	2.47522	10
0	-19	11	-0.8342	1.62965	13
0	19	-11	0.83006	2.00835	14
0	19	11	0.80004	2.67911	10
1	19	11	7.04164	2.91022	10
-1	19	-11	12.8239	2.38297	14
-4	-20	11	5.30955	2.06041	13
4	-20	-11	4.18063	1.89910	13
-3	-20	11	23.4498	3.40607	13
3	-20	-11	14.4528	2.56413	13
-3	20	11	16.1465	2.05273	11
2	-20	-11	9.97143	2.56122	13
-2	-20	11	13.1938	2.35207	13
-2	20	11	10.9491	2.62689	10
-1	-20	11	3.92362	1.78221	13
-1	20	11	3.60253	2.24577	10
0	-20	11	0.92068	1.76301	13
0	20	11	1.36454	2.12788	10
3	-21	-11	4.15690	2.05920	13
-3	-21	11	2.33964	2.01780	13
-2	-21	11	41.0911	4.06878	13
-1	-21	11	6.12887	1.73319	13
-8	0	12	36.3069	4.26360	10
8	0	-12	30.6170	5.24569	13
7	0	-12	0.05595	2.82413	13
-7	0	12	0.36199	1.82660	10
-6	0	12	4.94081	3.13422	10
-5	0	12	48.5917	6.10010	10
3	0	-12	0.53094	2.64013	10
-2	0	12	80.4282	8.28926	13
2	0	-12	78.5137	7.30984	10
1	0	-12	93.7999	7.89532	10
1	0	-12	92.4939	9.31828	8
-1	0	12	87.9730	8.34149	13
0	0	12	635.778	41.3552	13
0	0	-12	664.547	41.2744	10
0	0	-12	651.041	42.8137	1
0	0	-12	654.320	40.6657	8
-1	0	-12	5.93413	2.08851	8
-1	0	-12	5.33245	2.39001	10

1	0	12	3.50818	3.08255	13
−2	0	−12	−1.8049	1.90556	8
2	0	12	−1.3591	3.72137	13
−2	0	−12	−0.8686	2.32692	10
−3	0	−12	31.1995	4.60283	14
−3	0	−12	22.2475	3.49187	12
3	0	12	22.2291	4.01984	13
−3	0	−12	25.5802	3.33667	8
4	0	12	7.20232	2.85137	13
−4	0	−12	2.19078	1.33597	8
−4	0	−12	4.73706	2.14837	12
−5	0	−12	1.99337	1.47830	12
5	0	12	1.98830	2.02682	13
8	−1	−12	24.1556	4.40402	13
−8	−1	12	14.2997	3.06077	10
−8	1	12	22.8987	3.45893	10
7	−1	−12	26.0923	4.09264	13
−7	1	12	24.8612	3.69084	10
−6	−1	12	19.6979	4.01061	10
6	−1	−12	14.3624	4.06460	13
−6	1	12	19.6551	3.61825	10
−5	−1	12	50.7011	6.20654	10
−5	1	12	50.1567	6.26356	10
−4	1	12	11.9211	3.50504	10
4	1	−12	7.10842	3.15452	10
−3	−1	12	67.8274	6.80731	13
3	−1	−12	65.5508	6.95541	10
3	1	−12	75.4507	7.08350	10
−2	−1	12	113.761	10.2433	13
2	−1	−12	138.446	10.1337	10
2	1	−12	114.635	9.83895	10
−2	1	12	138.589	10.6319	13
−1	−1	12	202.772	15.2931	13
1	−1	−12	228.797	15.0315	10
1	1	−12	199.736	14.0915	8
1	1	−12	193.295	14.5309	10
−1	1	12	224.880	15.3726	13
0	−1	−12	157.753	12.6515	1
0	−1	12	120.264	10.8209	13
0	−1	−12	143.917	10.5318	10
0	−1	−12	148.912	9.92809	8
0	1	12	146.277	11.2147	13
0	1	−12	125.720	11.2533	1
0	1	−12	124.605	10.2117	10
0	1	−12	133.341	9.80903	8
−1	−1	−12	36.1524	4.15017	8
−1	−1	−12	38.4747	5.39429	1
1	−1	12	38.6358	6.83854	13
−1	−1	−12	33.4860	4.51131	10
1	1	12	33.0173	5.70775	13

−1	1	−12	28.6687	4.23655	8
−1	1	−12	29.7581	4.09254	10
−2	−1	−12	9.93374	2.45869	8
−2	1	−12	9.86020	2.44575	8
−2	1	−12	13.9688	2.82362	10
2	1	12	17.6115	3.98336	13
−3	−1	−12	8.56395	2.59511	12
3	−1	12	5.09229	3.03001	13
−3	−1	−12	6.38713	2.00359	8
−3	−1	−12	9.37049	2.76781	14
−3	−1	−12	6.09510	1.76109	10
3	1	12	8.29887	3.24900	13
−3	1	−12	2.64080	2.47766	14
−3	1	−12	4.06100	1.70556	8
−3	1	−12	1.07025	2.35740	12
−4	−1	−12	30.5289	4.05425	12
−4	−1	−12	31.6102	2.73174	11
4	−1	12	40.7995	5.25183	13
−4	−1	−12	31.3419	3.21396	8
−4	1	−12	33.0655	3.05301	8
−4	1	−12	33.7848	4.21995	12
4	1	12	33.0108	5.19922	13
−5	−1	−12	0.75714	1.40835	12
5	−1	12	4.29593	2.05645	13
5	1	12	1.16362	2.04096	13
−5	1	−12	3.27963	1.51273	12
8	−2	−12	12.2762	3.41642	13
−8	−2	12	4.88110	2.51756	10
−8	2	12	10.3730	2.45566	10
−7	−2	12	32.4530	4.22135	10
7	−2	−12	27.9151	5.38045	13
−7	2	12	35.5801	4.15203	10
−6	−2	12	2.40212	2.17968	10
6	−2	−12	0.94915	2.91174	13
−6	2	12	0.91365	2.77350	10
−5	−2	12	4.07310	2.89745	10
−5	2	12	−0.7281	2.67240	10
−4	2	12	34.2053	5.59688	10
4	2	−12	30.8902	5.08625	10
−3	−2	12	39.5813	6.41795	13
3	−2	−12	55.8485	6.00793	10
3	2	−12	44.1418	5.74707	10
−2	−2	12	29.7084	5.16224	13
2	2	−12	31.7262	4.81179	10
1	−2	−12	62.7461	6.66853	10
−1	−2	12	71.3252	7.83180	13
1	2	−12	71.5949	6.54230	10
−1	2	12	64.7634	7.11166	13
1	2	−12	71.8448	5.99329	8
0	−2	−12	10.3396	2.44600	8

0	−2	−12	12.4677	3.18942	10
0	−2	−12	12.5111	4.56729	1
0	−2	12	6.13847	3.26358	13
0	2	12	8.91172	3.50036	13
0	2	−12	10.9757	3.36859	1
0	2	−12	7.95531	2.63904	10
0	2	−12	9.24710	2.39121	8
−1	−2	−12	5.69193	3.26303	1
−1	−2	−12	6.78499	2.69443	10
−1	−2	−12	8.48810	2.31054	8
1	−2	12	2.52001	3.02849	13
1	2	12	9.37519	4.05181	13
−1	2	−12	3.04051	1.96330	8
−1	2	−12	2.47299	1.97414	10
2	−2	12	6.37950	3.29134	13
−2	−2	−12	5.41437	2.42567	10
−2	−2	−12	1.67712	2.07411	8
2	2	12	9.75345	4.20254	13
−2	2	−12	6.62149	2.07582	8
−2	2	−12	0.12640	2.84802	14
−2	2	−12	6.67048	2.74664	12
−2	2	−12	8.86316	2.10831	10
−3	−2	−12	0.32193	1.74066	8
−3	−2	−12	−1.3426	1.55809	10
3	−2	12	0.13866	2.60525	13
−3	−2	−12	1.51716	2.21292	12
−3	2	−12	−0.8263	2.30069	14
3	2	12	−0.1698	3.66426	13
−3	2	−12	−0.1144	1.44478	8
−3	2	−12	0.43723	2.23950	12
−4	−2	−12	6.14990	2.12614	12
4	−2	12	0.89974	2.36437	13
−4	−2	−12	4.16124	1.61834	8
4	2	12	4.46237	2.88095	13
−4	2	−12	2.17687	1.12443	8
−4	2	−12	5.72626	2.16457	12
5	−2	12	19.1630	3.48369	13
5	2	12	14.9673	3.00280	13
−5	2	−12	17.5560	2.65673	12
8	−3	−12	2.98440	2.60771	13
−8	−3	12	6.84523	2.06588	10
−8	3	12	6.24004	2.09045	10
7	−3	−12	37.1963	5.77074	13
−7	−3	12	45.0812	5.62332	10
−7	3	12	45.9989	5.27887	10
6	−3	−12	7.73488	3.09348	13
−6	3	12	4.84868	2.88309	10
5	−3	−12	23.8772	3.83353	13
−5	3	12	26.5343	4.94802	10
−4	3	12	15.7419	4.07496	10

4	3	−12	22.2848	3.94432	10
−3	−3	12	2.81757	3.46200	13
3	3	−12	0.40147	2.62202	10
−2	−3	12	42.1367	6.47116	13
2	3	−12	48.2755	5.49719	10
1	−3	−12	60.5255	6.08411	10
−1	−3	12	38.2550	5.87288	13
1	3	−12	45.1813	5.35361	10
−1	3	12	57.9090	6.93629	13
0	−3	−12	169.438	14.8921	1
0	−3	−12	161.826	13.3842	10
0	−3	12	190.937	13.9110	13
0	3	12	156.801	13.2697	13
0	3	−12	206.377	12.4870	8
0	3	−12	198.221	12.8989	10
1	−3	12	69.0056	8.00997	13
−1	−3	−12	76.7440	6.90726	10
−1	−3	−12	77.3303	7.91326	1
1	3	12	72.2145	8.39935	13
−1	3	−12	65.5731	5.90048	10
−1	3	−12	66.6729	5.78543	8
−2	−3	−12	16.8115	3.10068	10
2	−3	12	13.0378	4.09504	13
−2	−3	−12	13.4797	2.59311	8
−2	3	−12	6.59076	2.90321	14
−2	3	−12	9.24976	2.22136	8
2	3	12	17.6965	4.63570	13
−2	3	−12	9.91604	2.96456	12
−3	−3	−12	9.80885	2.21070	10
3	−3	12	7.78393	2.94712	13
−3	−3	−12	10.7954	2.25749	8
3	3	12	8.30458	4.09183	13
−3	3	−12	7.19504	1.35433	11
−3	3	−12	8.65929	1.19246	11
−3	3	−12	9.01267	2.04711	8
−3	3	−12	7.50803	2.61539	12
−3	3	−12	8.15880	2.74186	14
4	−3	12	5.41401	2.58810	13
−4	−3	−12	3.95699	1.73473	8
−4	−3	−12	2.48446	2.00368	12
−4	3	−12	7.11868	1.37641	8
−4	3	−12	7.66045	2.35202	12
4	3	12	7.17659	3.04968	13
5	−3	12	1.29828	1.78459	13
−5	3	−12	2.99466	1.63324	12
5	3	12	1.98956	2.05419	13
−8	−4	12	11.4256	2.81969	10
8	−4	−12	7.62581	3.25348	13
−8	4	12	8.38961	2.09053	10
7	−4	−12	12.1809	3.48276	13

−7	4	12	5.41816	2.77462	10
6	−4	−12	2.43675	2.79975	13
−6	−4	12	3.81007	2.93136	10
−6	4	12	−0.0332	2.39410	10
5	−4	−12	8.36660	3.07171	13
5	4	−12	0.68988	2.19006	10
−5	4	12	5.22963	3.10307	10
−4	4	12	31.9795	5.69562	10
4	4	−12	42.4733	5.56844	10
−3	−4	12	40.1676	6.50444	13
−3	4	12	29.9728	5.88540	10
3	4	−12	40.4930	5.27618	10
−2	−4	12	215.804	14.7090	13
2	−4	−12	174.212	14.5245	10
2	4	−12	222.945	14.6222	10
1	−4	−12	52.8595	6.45747	10
−1	−4	12	74.2500	7.85143	13
−1	4	12	47.6565	7.10117	13
1	4	−12	73.2131	6.24934	10
0	−4	12	393.735	26.4521	13
0	−4	−12	416.469	26.6179	10
0	−4	−12	409.693	28.7273	1
0	4	12	391.884	26.6766	13
0	4	−12	405.135	26.0653	10
0	4	−12	399.842	25.5586	8
1	−4	12	19.9017	4.33384	13
−1	4	−12	16.1314	3.09579	12
−1	4	−12	15.6188	2.98811	10
−1	4	−12	18.5458	2.68333	8
−1	4	−12	21.0184	3.37576	14
2	−4	12	12.8522	4.00994	13
−2	−4	−12	8.61227	2.44341	8
−2	−4	−12	8.71801	2.99893	10
−2	4	−12	13.7838	2.96286	12
2	4	12	14.2665	3.96920	13
−2	4	−12	16.0786	2.22531	11
−2	4	−12	15.8218	2.62663	8
−2	4	−12	15.0251	3.20058	14
−3	−4	−12	14.6053	2.65066	10
−3	−4	−12	16.5050	2.51521	8
3	−4	12	11.7289	3.09373	13
−3	4	−12	13.8816	2.97534	14
3	4	12	18.9762	3.88716	13
−3	4	−12	13.8145	2.15183	8
−3	4	−12	9.71309	2.80596	12
−4	−4	−12	8.43816	2.14100	12
−4	−4	−12	9.31804	1.98767	8
−4	4	−12	13.4897	2.57289	12
4	4	12	6.04031	3.06430	13
5	−4	12	1.83234	1.73525	13

5	4	12	2.51602	2.02110	13
−5	4	−12	2.94951	1.61697	12
−8	−5	12	13.7792	3.07983	10
8	−5	−12	19.5421	3.17105	13
−8	5	12	18.7598	2.60044	10
−7	−5	12	17.1982	3.63837	10
7	−5	−12	12.4099	3.52742	13
−7	5	12	19.7368	3.75969	10
6	−5	−12	0.95617	2.79218	13
−6	−5	12	0.95923	2.69051	10
−6	5	12	1.65885	2.51026	10
5	−5	−12	28.2722	5.15597	13
−5	5	12	23.6339	4.37514	10
5	5	−12	26.2633	5.17369	10
4	−5	−12	60.3303	7.26692	13
−4	−5	12	74.5347	7.43608	13
−4	5	12	73.5138	7.85190	10
4	5	−12	90.9735	7.63515	10
−3	−5	12	30.3958	5.30988	13
3	5	−12	28.7470	4.84289	10
−3	5	12	33.7364	5.91044	10
−2	−5	12	115.356	10.4132	13
2	−5	−12	130.321	10.0997	10
2	5	−12	120.679	9.54875	10
2	5	−12	117.619	9.01606	14
1	−5	−12	188.562	12.6810	10
−1	−5	12	158.344	12.8526	13
1	5	−12	159.606	11.8496	10
1	5	−12	149.883	11.8269	14
0	−5	12	20.7031	3.99174	13
0	5	−12	21.4381	3.39358	14
0	5	−12	21.5237	3.45028	12
0	5	−12	19.4324	3.46936	10
−1	−5	−12	22.6241	4.30858	10
1	−5	12	10.5574	3.88225	13
−1	5	−12	11.2460	2.25982	10
−1	5	−12	12.7041	2.99134	14
−1	5	−12	12.3770	2.69782	12
−1	5	−12	11.0319	2.59822	8
−2	−5	−12	18.7044	3.71925	10
−2	−5	−12	16.8255	2.96616	8
2	−5	12	21.9324	3.90351	13
−2	5	−12	25.8297	3.46803	8
−2	5	−12	25.1715	4.16115	12
−2	5	−12	24.2169	2.89232	11
−2	5	−12	25.9982	3.65957	14
2	5	12	23.8570	4.44122	13
−3	−5	−12	25.1923	3.72306	10
−3	−5	−12	26.1296	3.58141	8
3	−5	12	26.5250	4.21694	13

−3	5	−12	26.2633	4.08408	12
3	5	12	26.3654	4.27941	13
−3	5	−12	27.2662	4.57144	14
−4	−5	−12	10.6007	2.16868	12
4	−5	12	10.4722	2.63749	13
−4	−5	−12	10.0337	3.10412	10
−4	−5	−12	12.1248	1.77180	8
−4	5	−12	11.4922	2.51699	12
4	5	12	8.93773	3.02215	13
−5	5	−12	4.31887	1.62405	12
5	5	12	2.36164	2.42580	13
−8	−6	12	14.0443	2.70788	10
8	−6	−12	13.5860	2.90937	13
−8	6	12	13.1311	1.89636	11
7	−6	−12	17.2107	3.53403	13
−7	−6	12	15.2562	3.49191	10
−7	6	12	18.2794	3.49512	10
−6	−6	12	8.86051	2.87817	10
6	6	−12	6.95572	2.95479	10
5	−6	−12	37.8212	6.65403	13
5	6	−12	27.8750	4.53318	10
−5	6	12	40.5031	5.71693	10
−4	−6	12	77.2341	7.97383	13
4	−6	−12	82.9309	7.71506	13
4	6	−12	75.6529	7.06079	10
−4	6	12	84.1621	8.20331	10
−3	−6	12	20.5670	4.40884	13
3	6	−12	21.5403	3.87752	10
−3	6	12	21.6797	4.46222	10
−2	−6	12	33.2293	6.31934	13
2	6	−12	29.8225	4.55481	10
2	6	−12	28.0146	4.88805	14
−1	−6	12	42.1368	5.82938	13
1	6	−12	51.8229	5.02068	14
1	6	−12	35.9959	4.71783	10
0	−6	−12	13.9260	4.66596	1
0	−6	−12	11.2578	3.40233	10
0	6	12	12.1539	3.42919	13
1	−6	12	14.6851	3.51459	13
−1	−6	−12	26.6821	5.29147	1
−1	−6	−12	24.4763	4.51323	10
1	6	12	21.6494	4.75218	13
−1	6	−12	16.4409	2.88386	12
−1	6	−12	14.0616	3.00523	14
−1	6	−12	17.0138	2.28575	11
−2	−6	−12	4.01197	2.06347	8
−2	−6	−12	2.78546	2.58362	10
2	−6	12	5.30738	2.79846	13
−2	6	−12	3.96115	1.15572	11
−2	6	−12	3.37378	2.37598	12

2	6	12	2.80802	3.28391	13
−2	6	−12	1.98948	2.47885	14
3	−6	12	−2.3753	2.85669	13
−3	−6	−12	3.62937	2.23056	10
−3	−6	−12	1.31096	1.43700	8
3	6	12	0.27667	3.06841	13
−3	6	−12	3.52220	2.55595	14
−3	6	−12	0.10081	2.22850	12
−4	−6	−12	5.05845	1.69895	12
−4	−6	−12	5.87665	1.75239	10
−4	−6	−12	6.15967	1.78468	8
4	6	12	5.69811	2.73811	13
−4	6	−12	2.46896	2.00014	12
8	−7	−12	8.57594	2.99148	13
−8	−7	12	11.5448	2.73663	10
−8	7	12	9.38566	1.37105	11
7	−7	−12	11.0492	3.25611	13
−7	7	12	11.7393	2.63206	10
6	−7	−12	15.6091	4.15738	13
−6	7	12	10.3680	2.95051	10
6	7	−12	11.4134	3.54617	10
5	−7	−12	37.9676	6.49383	13
−5	7	12	24.6338	4.46353	10
5	7	−12	23.2007	4.36470	10
−4	−7	12	23.8822	4.22975	13
4	7	−12	21.2022	3.85681	10
−3	−7	12	76.1343	8.03972	13
3	7	−12	73.2842	7.00480	10
−3	7	12	68.4137	7.86686	10
−2	−7	12	17.4687	4.39864	13
2	7	−12	17.6159	3.37985	10
1	−7	−12	15.6914	5.77897	1
−1	−7	12	5.65643	3.12813	13
1	−7	−12	11.8004	3.53480	10
1	7	−12	8.27691	2.51113	10
1	7	−12	10.1788	3.08631	14
0	−7	−12	36.5955	5.54307	10
0	−7	12	43.9624	5.99811	13
0	−7	−12	47.4058	7.55821	1
0	7	−12	44.1202	4.32025	10
0	7	−12	37.0720	4.84228	14
−1	−7	−12	81.4591	9.20866	1
1	−7	12	66.9025	7.39501	13
−1	7	−12	81.0035	7.08267	12
−1	7	−12	73.4404	6.91623	14
−1	7	−12	77.2441	5.73031	11
2	−7	12	−1.1612	2.45637	13
−2	−7	−12	5.13946	2.89307	10
−2	7	−12	0.20843	0.82246	11
−2	7	−12	0.26953	0.78564	11

−2	7	−12	−0.2891	2.35503	14
−2	7	−12	−2.2069	2.17013	12
−3	−7	−12	8.13871	2.69890	10
−3	−7	−12	10.5349	2.46139	8
3	7	12	8.21499	4.11585	13
−3	7	−12	14.7294	2.91936	12
−3	7	−12	15.9920	3.14190	14
−4	−7	−12	0.81751	1.60187	8
−4	−7	−12	0.44896	1.69757	10
−4	−7	−12	−0.6901	1.60504	12
4	7	12	2.23253	2.51975	13
−4	7	−12	0.27061	1.95600	12
8	−8	−12	14.2577	3.04268	13
−8	−8	12	8.37269	2.55273	10
−8	8	12	13.2852	1.41266	11
−7	−8	12	9.02384	3.02509	10
−7	8	12	8.63375	2.19566	10
6	−8	−12	15.1273	3.42595	13
−6	8	12	13.0460	3.02489	10
6	8	−12	14.3333	3.07151	10
5	−8	−12	8.82026	3.74751	13
−5	8	12	2.83188	2.98227	10
5	8	−12	0.26280	2.84205	10
−4	−8	12	39.5803	5.96200	13
4	−8	−12	25.5477	4.80306	13
4	8	−12	33.3354	5.21574	10
−4	8	12	29.5260	5.54314	10
−3	−8	12	4.67516	3.53495	13
−3	8	12	8.19423	3.64133	10
3	8	−12	5.27104	2.79260	10
−2	−8	12	18.4503	3.86493	13
−2	8	12	16.7914	4.33515	10
2	8	−12	20.2110	3.92007	10
−1	−8	12	7.43758	3.10379	13
1	−8	−12	6.54424	3.33796	10
1	−8	−12	6.34777	4.87205	1
1	8	−12	7.52828	2.35314	10
1	8	−12	9.31919	2.55559	14
0	−8	−12	0.79956	3.63443	1
0	−8	12	3.24792	2.84181	13
0	−8	−12	2.51736	2.86887	10
0	8	−12	1.99904	2.30849	14
0	8	−12	3.20180	2.16442	10
1	−8	12	11.9593	3.29373	13
−1	−8	−12	8.75318	4.15237	1
−1	−8	−12	12.0569	3.44923	10
1	8	12	12.2181	4.07610	13
−1	8	−12	16.3100	2.90778	12
−1	8	−12	10.3363	2.87990	14
−1	8	−12	12.3282	1.92726	11

−2	−8	−12	4.07810	2.87495	10
2	−8	12	8.76094	2.96314	13
2	8	12	5.18299	3.29878	13
−3	−8	−12	10.9755	2.77569	10
−3	−8	−12	12.1638	2.44986	8
−3	8	−12	10.6355	2.87121	14
3	8	12	10.8384	3.28210	13
−3	8	−12	8.27894	2.50022	12
−4	−8	−12	8.53394	1.81385	10
−4	8	−12	9.36952	2.23235	12
4	8	12	8.96148	2.61328	13
8	−9	−12	3.97599	2.15076	13
−7	−9	12	49.6804	4.77113	10
7	−9	−12	40.3739	5.31788	13
−7	9	12	37.2803	3.90624	10
7	9	−12	46.1970	4.76369	10
6	−9	−12	19.6659	3.55998	13
6	9	−12	16.8096	3.69572	10
−6	9	12	14.6349	2.96487	10
5	−9	−12	22.9158	3.86358	13
−5	9	12	24.0126	4.27437	10
5	9	−12	26.3175	5.03031	10
4	−9	−12	17.1638	3.94180	13
−4	9	12	19.2927	4.44070	10
3	−9	−12	18.7593	3.29618	13
−3	−9	12	9.87249	3.34931	13
3	9	−12	10.4656	3.26081	10
−2	−9	12	87.9172	8.21975	13
−2	9	12	68.9610	7.95332	10
2	9	−12	68.9904	6.63674	10
−1	−9	12	11.9175	3.41639	13
1	−9	−12	10.4519	4.78424	1
1	9	−12	15.9698	2.99088	14
1	9	−12	12.6254	3.07740	10
0	−9	−12	36.4328	5.36954	10
0	−9	−12	35.5024	7.20989	1
0	9	−12	24.3040	3.66291	14
0	9	−12	22.7647	3.25031	11
−1	−9	−12	28.0207	4.73365	10
1	−9	12	17.8018	3.49076	13
−1	9	−12	11.6568	2.76789	12
−1	9	−12	17.6514	2.30995	11
−1	9	−12	18.8548	3.37095	14
−1	9	−12	22.0209	2.86126	11
−2	−9	−12	29.5655	4.62976	10
2	9	12	34.1717	5.13347	13
−2	9	−12	31.5703	4.01381	14
−2	9	−12	27.5177	4.12528	12
−3	−9	−12	11.0898	2.78203	10
−3	−9	−12	9.30189	2.70456	8

-3	9	-12	16.9557	2.97051	14
3	9	12	9.72402	3.40731	13
-3	9	-12	14.4984	2.68269	12
-4	-9	-12	1.79208	1.52107	10
4	9	12	2.29055	2.50708	13
-4	9	-12	1.17495	1.80705	12
7	-10	-12	2.13794	2.46655	13
-7	10	12	1.63796	1.04418	11
6	-10	-12	35.2786	5.11368	13
6	10	-12	17.0230	3.88794	10
-6	10	12	26.6171	3.69642	10
5	-10	-12	66.6645	7.41785	13
5	10	-12	68.3415	6.81573	10
-5	10	12	54.8878	6.32243	10
4	-10	-12	4.11947	2.93560	13
-4	-10	12	-1.8702	3.21053	13
4	10	-12	0.03024	2.06223	10
-4	10	12	6.78705	3.53519	10
3	-10	-12	7.73071	2.91445	13
-3	-10	12	9.69343	3.37815	13
-3	10	12	8.92567	3.66823	10
-2	-10	12	97.2392	9.28025	13
-2	10	12	111.424	9.59871	10
2	10	-12	92.6529	8.31972	10
-1	-10	12	102.052	8.89641	13
1	-10	-12	94.4310	11.3965	1
-1	10	12	93.9662	9.63492	10
1	10	-12	106.285	7.93169	10
1	10	-12	115.567	8.55792	14
0	-10	-12	32.0762	5.21444	10
0	-10	12	23.3278	3.99085	13
0	-10	-12	28.0578	6.11776	1
0	10	-12	27.5970	3.91891	14
0	10	-12	24.9503	3.05096	11
1	-10	12	-0.9601	2.95753	13
-1	-10	-12	2.64779	3.03347	10
-1	10	-12	-2.2350	2.32800	14
-1	10	-12	-0.4897	0.73412	11
-2	-10	-12	9.03894	2.39092	10
-2	10	-12	3.67017	2.21508	12
-2	10	-12	-1.3655	2.58254	14
-3	-10	-12	5.64535	2.00821	10
-3	10	-12	4.42758	2.30512	12
-3	10	-12	6.81684	2.55078	14
-4	-10	-12	0.22777	1.48401	10
-4	10	-12	2.33987	1.75151	12
7	-11	-12	27.4027	4.16975	13
-7	11	12	21.8757	2.04913	11
6	-11	-12	3.44775	2.73022	13
-6	11	12	4.05963	1.99786	10

6	11	-12	10.2428	3.19108	10
5	-11	-12	58.0579	6.92721	13
-5	11	12	54.9613	5.86582	10
-4	-11	12	5.66588	2.80804	13
4	-11	-12	6.20110	3.36302	13
-4	11	12	6.18594	3.32831	10
4	11	-12	6.28416	2.94759	10
-3	-11	12	16.0584	3.85870	13
3	-11	-12	28.0580	4.88515	13
-3	11	12	26.1289	4.67367	10
3	11	-12	18.0708	3.62634	10
-2	-11	12	2.50925	3.69984	13
-2	11	12	19.7055	4.47744	10
-1	-11	12	164.200	12.4243	13
1	11	-12	166.158	12.1531	14
-1	11	12	183.743	13.6191	10
1	11	-12	157.766	11.3598	10
0	-11	12	7.04183	3.51769	13
0	-11	-12	11.3291	2.79189	10
0	11	-12	6.10019	2.86412	14
0	11	-12	9.87117	1.58970	11
1	-11	12	13.5610	3.12789	13
-1	-11	-12	18.3148	3.44212	10
-1	11	-12	11.3294	3.06602	14
-2	-11	-12	11.1352	2.61801	10
-2	11	-12	11.7808	2.90138	14
-2	11	-12	12.6323	2.63784	12
-3	-11	-12	4.03284	1.77844	10
-3	11	-12	1.94664	2.22709	14
-3	11	-12	1.60125	1.10605	9
-3	11	-12	0.08797	1.88667	12
-4	-11	-12	1.50895	1.92793	10
-4	11	-12	4.92506	1.70948	12
7	-12	-12	17.5290	2.82128	13
-7	12	12	15.3896	1.59439	11
7	12	-12	12.2149	2.53809	10
-7	12	12	18.4298	1.60234	11
6	-12	-12	26.4370	4.59379	13
-6	12	12	22.9705	3.07454	10
-5	12	12	23.2469	4.14671	10
-4	-12	12	6.59880	3.35270	13
4	-12	-12	6.17216	2.99404	13
-4	12	12	6.21265	3.26233	10
4	12	-12	5.28709	2.75746	10
-3	-12	12	50.4149	6.13759	13
3	-12	-12	50.3893	5.68833	13
-3	12	12	43.4109	6.13277	10
3	12	-12	39.6442	5.03072	10
-2	-12	12	52.7449	5.98834	13
-2	12	12	33.9000	6.39115	10

2	12	−12	51.3469	4.90028	10
−1	−12	12	2.64735	2.75417	13
1	12	−12	4.32074	2.21728	11
1	12	−12	4.00801	2.71320	14
0	−12	−12	41.4684	5.63800	10
0	−12	12	38.9423	6.25261	13
0	12	−12	38.1467	3.13978	11
0	12	−12	36.9666	3.21218	11
0	12	12	27.8467	5.50468	10
1	−12	12	40.9529	5.02479	13
−1	−12	−12	31.1207	5.04418	10
−2	−12	−12	27.8210	3.72470	10
−2	12	−12	29.9651	4.61647	14
−2	12	−12	31.0470	3.82609	12
−3	−12	−12	0.04961	1.48596	10
−3	12	−12	0.78181	0.82529	9
−3	12	−12	−0.3409	1.63920	12
−3	12	−12	1.44678	2.11141	14
7	−13	−12	5.17265	2.10849	13
6	−13	−12	30.4683	4.82826	13
6	13	−12	19.1877	3.59085	10
−6	13	12	20.4617	2.30110	11
5	−13	−12	11.6091	3.11863	13
5	13	−12	14.4164	3.29012	10
−5	13	12	12.1413	3.13469	10
4	−13	−12	11.7273	3.13036	13
−4	−13	12	15.4494	3.21607	13
4	13	−12	13.4511	3.13217	10
−4	13	12	10.5342	2.75298	10
−3	−13	12	24.3517	3.82396	13
3	−13	−12	18.8635	3.62459	13
3	13	−12	27.9448	4.09478	10
−3	13	12	17.7165	4.13964	10
−2	−13	12	95.1281	8.59518	13
2	13	−12	92.1294	7.26526	10
1	13	−12	36.9797	5.08349	14
1	13	−12	25.6560	3.33791	11
0	−13	12	51.1464	5.72020	13
0	13	−12	50.8463	5.76486	14
0	13	12	53.1724	6.85302	10
−1	−13	−12	64.2437	6.69331	10
1	−13	12	68.3636	7.25164	13
−1	13	−12	69.7408	6.58479	14
−2	−13	−12	1.64280	2.65517	10
−2	13	−12	4.36684	2.50720	14
−3	−13	−12	2.78487	2.07834	10
−3	13	−12	4.20494	2.01125	14
−3	13	−12	1.65845	1.62054	12
6	−14	−12	4.97574	2.27506	13
6	14	−12	5.77563	2.09309	10

-6	14	12	4.42270	1.07703	11
5	-14	-12	29.8776	4.70631	13
-5	14	12	29.3107	4.04891	10
5	14	-12	38.8733	4.77789	10
4	-14	-12	23.0062	3.68224	13
-4	-14	12	23.1769	3.89989	13
-4	14	12	20.8432	3.50945	10
4	14	-12	22.8661	3.93054	10
3	-14	-12	26.8620	4.83725	13
-3	-14	12	35.1515	5.16652	13
-3	14	12	32.1321	5.07865	10
3	14	-12	37.8062	4.70481	10
-2	-14	12	50.9294	6.24622	13
2	14	-12	47.0741	4.65020	10
2	14	-12	49.3538	4.56011	11
-1	-14	12	6.66555	2.75851	13
1	14	-12	5.22737	2.65371	14
1	14	-12	7.83947	1.63040	11
-1	14	12	3.94961	3.33970	10
0	-14	12	3.89155	2.96450	13
0	14	-12	6.53675	2.93989	14
-1	-14	-12	55.0658	5.29727	10
-1	14	-12	50.1126	5.62690	14
-2	14	-12	18.5337	2.59975	14
-2	14	-12	21.9319	2.65216	9
-3	-14	-12	24.4700	3.34736	10
-3	14	-12	20.0785	3.39994	14
6	-15	-12	7.79098	2.20215	13
-6	15	12	7.91653	1.19253	11
6	15	-12	12.5564	2.72155	10
-6	15	12	7.61924	1.07433	11
5	-15	-12	15.1579	2.79418	13
-5	15	12	15.2079	2.69777	10
5	15	-12	9.46979	2.81589	10
-4	-15	12	10.4642	2.69209	13
4	-15	-12	17.5924	3.07919	13
-4	15	12	18.5229	3.60122	10
4	15	-12	11.8060	2.38924	10
-3	-15	12	34.5118	4.91723	13
3	15	-12	22.5806	3.60578	10
-3	15	12	25.3473	4.66168	10
-2	-15	12	8.93043	3.11372	13
2	15	-12	16.9862	2.30196	11
-2	15	12	12.8172	4.09618	10
-1	-15	12	9.58499	3.01245	13
1	15	-12	9.77193	2.97325	14
1	15	-12	6.62667	1.21038	11
1	15	-12	4.41786	1.24637	11
-1	15	12	0.81931	2.35541	10
0	-15	12	5.12040	2.38666	13

0	15	−12	4.69390	2.68144	14
0	15	12	4.29131	3.54657	10
−1	−15	−12	19.8202	3.24732	10
1	15	12	19.3125	3.80368	10
−1	15	−12	19.6562	3.10432	14
−2	−15	−12	43.7210	4.33153	10
−2	15	−12	47.1507	4.68564	14
6	−16	−12	8.20830	2.07520	13
5	−16	−12	3.90317	2.06390	13
−5	16	12	3.34656	1.02974	11
5	16	−12	3.65608	2.20465	10
−4	−16	12	8.10811	2.37793	13
4	−16	−12	3.98968	2.36419	13
−4	16	12	5.11514	2.59138	10
4	16	−12	8.41478	2.29355	10
−3	−16	12	18.1781	3.22076	13
3	−16	−12	26.7968	4.11582	13
3	16	−12	14.4059	2.77764	11
3	16	−12	19.2671	3.21097	10
−2	−16	12	2.94041	2.36725	13
−2	16	12	1.24441	2.92128	10
−1	−16	12	−1.0172	2.13613	13
−1	16	12	−0.4539	2.07056	10
1	16	−12	0.14125	2.30441	14
0	−16	12	6.64741	2.75494	13
0	16	12	6.54384	3.42223	10
0	16	−12	9.49368	2.73771	14
−1	16	−12	14.0434	2.68461	14
1	16	12	8.97321	2.69413	10
−2	16	−12	21.9388	3.42054	14
5	−17	−12	2.47117	1.83558	13
5	17	−12	3.33556	2.13112	10
−5	17	12	3.77614	0.78318	11
4	−17	−12	23.9394	3.64560	13
−4	17	12	26.0091	3.31852	10
−3	−17	12	16.8051	2.74662	13
3	−17	−12	8.05245	2.38225	13
−3	17	12	12.9051	2.81085	10
3	17	−12	14.7100	1.92394	11
−2	−17	12	15.8690	2.89326	13
2	17	−12	15.6429	1.86667	11
−2	17	12	11.3296	2.61735	10
2	17	−12	16.2998	2.00488	11
−1	−17	12	7.62831	2.37108	13
−1	17	12	6.48137	2.94751	10
1	17	−12	8.96420	2.75846	14
0	−17	12	12.5898	2.50931	13
0	17	−12	13.0051	2.71476	14
0	17	12	17.4673	3.31405	10
1	17	12	35.3206	4.73151	10

−1	17	−12	35.2135	4.09437	14
−4	−18	12	54.8228	5.05757	13
4	−18	−12	57.4191	5.11536	13
−4	18	12	55.8519	3.89876	11
4	18	−12	49.3759	4.22060	11
−3	−18	12	44.8696	4.41115	13
3	−18	−12	31.4583	3.93235	13
−3	18	12	33.7404	4.00209	10
3	18	−12	43.7507	3.30417	11
−2	−18	12	15.7944	2.65349	13
−2	18	12	13.1843	2.89673	10
−1	−18	12	−0.0637	2.23787	13
−1	18	12	−0.2926	2.42438	10
0	−18	12	9.29491	2.34406	13
0	18	−12	9.04723	2.15879	14
0	18	12	11.0700	2.42082	10
−1	18	−12	20.3963	3.18274	14
3	−19	−12	76.8946	5.54462	13
−3	−19	12	67.6016	5.68508	13
−2	−19	12	33.9580	3.78479	13
−2	19	12	30.2274	3.72565	10
−1	−19	12	6.71780	1.82439	13
−1	19	12	3.85520	1.73302	10
0	19	12	4.54097	2.52731	10
−8	0	13	0.42166	1.32475	10
−7	0	13	34.4828	4.68541	10
−6	0	13	24.4993	3.93323	10
−5	0	13	3.20973	2.93708	10
4	0	−13	8.12457	3.27590	10
3	0	−13	2.55682	2.84019	10
−2	0	13	12.3013	3.42147	13
2	0	−13	14.4436	3.41156	10
−1	0	13	43.5019	6.33691	13
1	0	−13	48.3844	5.58360	10
1	0	−13	46.9126	5.57311	8
0	0	13	−2.1934	3.76188	13
0	0	−13	0.56865	2.68919	1
0	0	−13	−0.5091	2.23788	10
0	0	−13	1.04394	2.04433	8
−1	0	−13	3.51669	2.15827	8
−1	0	−13	3.74658	2.30161	10
1	0	13	−1.0280	3.42348	13
−2	0	−13	9.35410	2.22498	8
−2	0	−13	8.62994	2.31535	10
−2	0	−13	10.6477	2.86474	14
−2	0	−13	11.5622	2.67132	12
2	0	13	7.18138	3.44548	13
−3	0	−13	0.32690	1.99854	12
3	0	13	0.49813	2.70315	13
−3	0	−13	0.27314	1.48614	8

-3	0	-13	-2.3430	1.98089	14
4	0	13	3.70503	2.24065	13
-4	0	-13	4.43492	1.80013	12
-8	-1	13	7.55618	2.31724	10
-8	1	13	6.77797	2.12574	10
-7	-1	13	11.9392	3.20983	10
-6	-1	13	28.0846	4.76813	10
-6	1	13	18.2502	3.83637	10
-5	-1	13	28.0058	4.90813	10
-5	1	13	16.6543	3.86578	10
-4	1	13	75.9982	9.01654	10
4	1	-13	100.270	8.40642	10
3	-1	-13	12.7597	3.54190	10
3	1	-13	17.2815	3.68427	10
-2	-1	13	29.7336	4.86980	13
2	-1	-13	29.0769	4.73815	10
-2	1	13	25.4467	4.06685	13
2	1	-13	28.1706	4.79161	10
-1	-1	13	54.1898	7.65758	13
1	-1	-13	69.8065	6.47708	10
1	1	-13	59.7444	6.29884	10
-1	1	13	78.4413	8.08455	13
1	1	-13	54.0833	5.61956	8
0	-1	-13	101.622	8.34073	10
0	-1	-13	103.326	9.76780	1
0	-1	13	99.1248	10.0345	13
0	-1	-13	103.006	7.88911	8
0	1	13	107.376	9.24698	13
0	1	-13	102.094	7.79057	8
0	1	-13	94.3517	8.11942	10
-1	-1	-13	25.6011	4.08899	10
-1	-1	-13	24.0111	3.79048	8
1	-1	13	21.1502	4.37100	13
-1	1	-13	21.4318	4.14370	10
1	1	13	26.1312	4.58453	13
-1	1	-13	22.7626	3.60008	8
-2	-1	-13	1.41371	1.81645	8
-2	-1	-13	0.71170	1.67282	10
2	-1	13	0.74593	3.02803	13
-2	1	-13	-0.6271	2.40324	14
-2	1	-13	0.91806	1.57146	12
-2	1	-13	-1.8726	1.70904	10
2	1	13	2.67717	3.20021	13
-2	1	-13	-0.4698	1.61497	8
-3	-1	-13	4.66206	1.71869	8
-3	-1	-13	3.90281	2.08237	12
-3	1	-13	5.31244	2.27069	12
-3	1	-13	4.31968	1.02187	11
3	1	13	12.0516	3.25608	13
-3	1	-13	8.89545	2.35880	14

-3	1	-13	5.13807	1.06328	11
-3	1	-13	5.10903	1.58273	8
4	-1	13	5.74021	2.24134	13
-4	-1	-13	4.77877	1.73155	12
4	1	13	0.78665	2.18200	13
-4	1	-13	4.36120	1.79424	12
-8	-2	13	16.2286	2.60891	10
-8	2	13	16.4135	2.84138	10
-7	-2	13	7.87228	2.97345	10
-7	2	13	4.31298	2.70137	10
-6	2	13	19.5159	3.82106	10
-5	-2	13	14.7706	3.72526	10
-5	2	13	14.6861	3.26401	10
5	2	-13	18.4659	4.29459	10
-4	2	13	136.857	10.7759	10
4	2	-13	127.557	10.6353	10
-3	-2	13	10.7469	3.43118	13
3	-2	-13	15.2615	3.64788	10
3	2	-13	18.9148	3.76708	10
-2	-2	13	15.6752	3.65683	13
2	-2	-13	9.62314	3.15218	10
2	2	-13	15.9362	3.41952	10
1	-2	-13	16.7324	3.59025	10
-1	-2	13	16.4164	4.45306	13
1	2	-13	18.4587	3.48236	10
-1	2	13	20.4512	4.38485	13
0	-2	-13	11.2478	3.20729	10
0	-2	13	22.6980	4.78914	13
0	-2	-13	16.6545	4.52612	1
0	-2	-13	14.2674	2.85754	8
0	2	-13	19.6227	3.75337	10
0	2	-13	17.2185	2.86215	8
1	-2	13	16.5850	3.95957	13
-1	2	-13	8.80322	3.03211	14
-1	2	-13	18.0120	2.69685	8
-1	2	-13	13.5498	2.85189	12
-1	2	-13	12.1543	2.57395	10
-2	-2	-13	3.24232	1.49840	8
-2	-2	-13	4.49526	2.03881	10
2	-2	13	4.13377	3.11303	13
2	2	13	1.11876	3.14831	13
-2	2	-13	2.54215	1.86223	14
-2	2	-13	2.81509	1.74083	8
-2	2	-13	2.03989	2.26967	12
3	-2	13	5.69220	2.87649	13
-3	-2	-13	2.82688	2.09911	12
-3	-2	-13	2.89106	1.66865	8
-3	2	-13	2.86030	2.23993	12
-3	2	-13	3.17232	1.41395	8
3	2	13	2.28842	2.91197	13

−3	2	−13	3.05274	0.61061	11
−3	2	−13	3.16639	2.17684	14
−3	2	−13	4.53413	0.60161	11
4	−2	13	2.06369	2.11772	13
−4	−2	−13	0.48216	1.45614	12
4	2	13	−0.8435	2.56374	13
−4	2	−13	0.40361	1.17152	12
−8	−3	13	24.7045	3.62495	10
−8	3	13	25.1543	3.40062	10
−7	−3	13	28.6064	3.77399	10
−7	3	13	21.8692	3.99886	10
−6	−3	13	10.7544	3.33921	10
−6	3	13	15.7992	3.47178	10
5	3	−13	57.0439	6.39254	10
−5	3	13	49.6872	6.32369	10
−4	3	13	57.8484	6.92590	10
4	3	−13	51.0875	5.75402	10
−3	−3	13	6.26403	3.68045	13
3	−3	−13	0.95648	2.81651	10
3	3	−13	2.57623	2.76768	10
2	−3	−13	7.78038	3.16324	10
−2	−3	13	9.73711	3.40787	13
2	3	−13	10.3826	3.11496	10
1	−3	−13	13.9765	4.63891	1
−1	−3	13	22.6388	4.55272	13
1	−3	−13	14.4054	3.59700	10
−1	3	13	11.9361	4.07932	13
0	−3	13	45.2971	6.81056	13
0	−3	−13	47.8159	5.69712	10
0	−3	−13	42.2680	7.19797	1
0	3	−13	48.7575	5.16617	10
−1	−3	−13	4.64622	2.62320	10
−1	−3	−13	5.60568	2.26608	8
1	−3	13	9.41729	3.35364	13
−1	3	−13	10.9506	2.29267	8
−1	3	−13	7.41811	2.41357	10
−1	3	−13	12.1679	2.68998	12
−1	3	−13	11.0074	2.97939	14
1	3	13	2.45864	4.17857	13
−2	−3	−13	17.7680	3.30755	10
−2	−3	−13	14.1934	2.56545	8
2	−3	13	19.0514	3.66491	13
−2	3	−13	14.3987	2.83222	12
−2	3	−13	15.5346	2.91667	14
−2	3	−13	16.0593	2.08398	11
2	3	13	12.4400	3.76945	13
−2	3	−13	16.6136	2.72633	8
−3	−3	−13	10.8392	2.02586	10
−3	−3	−13	10.4583	2.09616	8
3	−3	13	7.34097	2.91365	13

−3	−3	−13	12.7762	2.54033	12
−3	3	−13	10.0567	2.55632	12
−3	3	−13	13.3506	2.71756	14
3	3	13	12.7103	3.36120	13
−4	−3	−13	0.06672	1.40228	12
4	−3	13	1.58811	1.96986	13
−4	3	−13	1.30021	1.74518	12
4	3	13	0.83209	2.57251	13
−8	−4	13	6.59888	1.90204	10
8	−4	−13	9.78295	2.24827	13
−8	4	13	8.52500	1.80935	10
7	−4	−13	69.5251	6.44063	13
−7	−4	13	48.8277	5.26793	10
−7	4	13	58.3362	5.25674	10
−6	−4	13	1.99206	2.69751	10
−6	4	13	5.73109	2.79936	10
5	4	−13	28.5739	5.18463	10
−5	4	13	31.3072	5.14512	10
−4	4	13	0.84163	2.53730	10
−3	−4	13	27.8358	4.73274	13
3	4	−13	23.6782	4.69384	10
−3	4	13	20.6499	5.29998	10
2	−4	−13	6.35271	2.95889	10
−2	−4	13	1.15943	3.85972	13
2	4	−13	2.52947	2.61142	10
2	4	−13	5.23403	2.49376	14
1	−4	−13	136.437	11.4449	10
−1	−4	13	161.182	11.8551	13
1	−4	−13	144.872	13.5390	1
−1	4	13	134.270	11.3249	13
1	4	−13	164.889	11.0759	14
1	4	−13	162.415	11.2607	10
0	−4	13	83.6391	9.25144	13
0	−4	−13	97.4303	9.54720	1
0	−4	−13	78.1722	7.80514	10
0	4	13	92.8551	8.90414	13
0	4	−13	86.7604	7.14980	10
0	4	−13	82.9019	7.13021	12
0	4	−13	78.6897	7.58944	14
1	−4	13	9.34248	3.46428	13
−1	−4	−13	19.4196	3.38816	10
−1	−4	−13	12.2308	2.58151	8
−1	4	−13	10.9462	2.48063	10
−1	4	−13	15.3530	2.91413	12
−1	4	−13	13.5527	3.53342	14
1	4	13	14.6953	3.84350	13
2	−4	13	11.1162	3.17944	13
−2	−4	−13	9.70010	2.37248	8
−2	−4	−13	10.6680	2.57068	10
−2	4	−13	10.8553	2.75556	14

-2	4	-13	8.67588	1.50813	11
2	4	13	7.38343	3.47405	13
-2	4	-13	8.85662	2.59496	12
-3	-4	-13	-1.3198	1.37436	10
-3	-4	-13	0.92616	1.96200	12
-3	-4	-13	1.61517	1.68407	8
3	-4	13	3.96143	2.58380	13
3	4	13	1.96901	2.90516	13
-3	4	-13	6.72540	2.43383	14
-4	-4	-13	0.40985	1.38392	12
4	-4	13	1.01703	1.85969	13
-4	-4	-13	1.95612	0.78956	11
-4	4	-13	-0.0144	1.59633	12
4	4	13	0.13516	2.03947	13
-8	-5	13	5.67815	2.24232	10
8	-5	-13	8.14116	2.21972	13
-7	-5	13	8.99713	2.43472	10
7	-5	-13	14.4718	2.89569	13
-7	5	13	16.5697	2.73261	10
-6	-5	13	11.7620	2.82125	10
6	-5	-13	-1.4098	2.77750	13
6	5	-13	8.63578	2.78332	10
-6	5	13	1.06875	2.60952	10
-5	5	13	22.1322	4.05205	10
5	5	-13	12.9564	3.05137	10
4	5	-13	25.9122	5.12819	10
-4	5	13	26.9759	4.99487	10
-3	-5	13	52.3855	6.36921	13
3	5	-13	50.7878	6.05857	10
-2	-5	13	20.8585	4.08355	13
2	-5	-13	12.4054	3.00486	10
2	5	-13	15.1714	3.40555	10
2	5	-13	21.4572	3.55394	14
-1	-5	13	109.211	9.63744	13
1	5	-13	113.606	8.77657	14
1	5	-13	113.331	8.86152	10
0	-5	13	81.7400	9.03050	13
0	5	-13	85.9340	6.90718	10
0	5	-13	86.1262	7.49467	14
0	5	-13	82.8896	7.00545	12
1	-5	13	27.6851	4.31689	13
-1	-5	-13	23.7168	4.55712	10
-1	-5	-13	23.4426	3.82047	8
-1	5	-13	31.6857	4.43649	14
-1	5	-13	29.8585	4.15906	12
1	5	13	27.7607	4.97442	13
2	-5	13	11.4434	3.19545	13
-2	-5	-13	15.4152	2.66704	8
2	5	13	17.9722	3.70850	13
-2	5	-13	8.19859	1.27761	11

−2	5	−13	6.34048	2.66673	12
−2	5	−13	8.66244	1.37675	11
−2	5	−13	9.15535	2.77032	14
−3	−5	−13	6.13381	2.13287	10
−3	−5	−13	6.49128	2.12257	12
−3	−5	−13	6.85782	1.95444	8
3	−5	13	7.53249	2.63754	13
−3	5	−13	8.31493	2.32705	12
−3	5	−13	14.3434	2.74948	14
3	5	13	7.91057	2.94217	13
4	−5	13	1.39637	2.17031	13
4	5	13	0.10829	2.00905	13
−4	5	−13	0.46586	1.60554	12
8	−6	−13	14.6540	4.65793	13
7	−6	−13	26.3622	4.33224	13
−7	−6	13	33.0416	3.88782	10
−7	6	13	27.8496	3.75630	10
6	−6	−13	8.10187	2.99966	13
6	6	−13	15.3573	3.48336	10
−6	6	13	8.22055	2.88569	10
−5	6	13	25.1314	4.45911	10
−4	−6	13	78.4328	7.58078	13
−4	6	13	94.5183	8.77373	10
4	6	−13	81.4752	7.35313	10
−3	−6	13	38.2137	5.88562	13
3	6	−13	37.2598	5.28722	10
−3	6	13	29.8884	5.52377	10
−2	−6	13	83.4408	8.14175	13
2	−6	−13	76.5762	7.73475	10
2	6	−13	83.7560	7.01720	14
2	6	−13	85.3677	7.38559	10
1	−6	−13	16.0322	3.83314	10
1	−6	−13	18.5193	4.96029	1
−1	−6	13	7.05131	3.73192	13
1	6	−13	9.14010	2.98793	14
1	6	−13	13.2486	3.11714	10
0	−6	13	32.6089	5.91553	13
0	6	−13	36.5163	4.06227	10
0	6	−13	33.1891	4.89641	14
1	−6	13	5.51230	3.20575	13
−1	6	−13	1.48461	1.84402	11
−1	6	−13	8.03895	2.80122	14
1	6	13	2.87825	3.83597	13
−1	6	−13	1.06784	2.27923	12
−2	−6	−13	4.98802	2.59506	10
−2	−6	−13	7.51626	2.46121	8
2	−6	13	9.22608	3.18369	13
−2	6	−13	7.46122	2.03584	12
2	6	13	3.94192	3.19609	13
−2	6	−13	7.24984	2.84053	14

-3	-6	-13	9.12071	2.09770	8
-3	-6	-13	12.1923	2.27718	12
3	-6	13	9.67831	2.62311	13
-3	-6	-13	12.2947	2.38736	10
3	6	13	9.38677	2.91458	13
-3	6	-13	12.9678	2.60495	14
-3	6	-13	11.3198	1.99715	12
-4	6	-13	12.3217	2.09640	12
4	6	13	7.65900	2.28288	13
7	-7	-13	22.6170	3.61692	13
-7	-7	13	19.7135	3.96011	10
-7	7	13	20.6425	3.17085	10
6	-7	-13	12.0161	3.02205	13
-6	7	13	10.0546	2.46318	10
6	7	-13	11.2135	2.71693	10
5	-7	-13	37.7803	5.89306	13
5	7	-13	27.3044	4.94937	10
-5	7	13	25.4906	4.96850	10
-4	-7	13	74.8621	7.33488	13
4	7	-13	79.2487	6.91376	10
-4	7	13	80.1382	7.51463	10
-3	-7	13	46.5093	6.10684	13
3	7	-13	42.5732	5.64080	10
-3	7	13	51.4020	6.95391	10
2	-7	-13	50.9907	8.12458	10
2	7	-13	57.3917	5.85322	10
-2	7	13	51.0906	6.57151	10
2	7	-13	60.6661	8.67648	14
-1	-7	13	108.505	9.73527	13
1	7	-13	109.126	8.49785	10
1	7	-13	114.564	9.23209	14
0	-7	13	174.537	12.6591	13
0	-7	-13	147.691	12.3805	10
0	7	-13	180.139	12.0700	14
0	7	-13	173.963	11.5329	10
1	-7	13	2.45062	2.76535	13
-1	-7	-13	-0.8207	2.49913	10
-1	7	-13	3.31828	1.19363	11
-1	7	-13	2.82474	2.07448	12
1	7	13	2.14603	2.70877	13
-1	7	-13	1.25519	2.29574	14
-2	-7	-13	37.9685	4.23707	8
2	-7	13	33.5621	4.97469	13
-2	-7	-13	39.8428	4.58969	10
-2	7	-13	32.3457	4.92765	14
2	7	13	46.0447	6.05003	13
-2	7	-13	35.7097	4.27806	12
-3	-7	-13	27.0842	3.38517	8
3	-7	13	25.1871	4.10830	13
-3	-7	-13	28.7434	3.41343	12

−3	−7	−13	27.0531	3.27261	10
3	7	13	28.4372	5.04085	13
−3	7	−13	30.7045	3.74955	12
−3	7	−13	31.9657	4.14549	14
−7	−8	13	15.1482	2.87281	10
7	8	−13	14.8568	2.86840	10
6	−8	−13	6.32957	2.68650	13
6	8	−13	12.4317	3.17414	10
−6	8	13	4.44156	2.37126	10
5	−8	−13	19.8341	4.13569	13
−5	8	13	16.3108	3.21535	10
−4	−8	13	14.8723	3.35138	13
−4	8	13	5.86968	3.59242	10
4	8	−13	15.5553	3.71629	10
−3	−8	13	36.0126	5.80500	13
−3	8	13	41.2107	6.30374	10
3	8	−13	38.5150	5.15698	10
−2	−8	13	−2.0317	3.10595	13
−2	8	13	8.63219	3.85362	10
2	8	−13	1.46257	2.46882	10
1	−8	−13	154.358	11.8241	10
−1	−8	13	150.836	11.9864	13
1	8	−13	152.786	10.9836	10
1	8	−13	160.148	11.1921	14
0	−8	13	34.4552	5.81078	13
0	8	−13	37.9353	5.06420	14
0	8	−13	35.7985	3.68597	11
−1	−8	−13	4.17926	2.81165	10
1	−8	13	2.67895	2.62657	13
−1	8	−13	0.35759	2.02217	12
−1	8	−13	0.96402	1.07633	11
−1	8	−13	0.41577	1.05090	11
−1	8	−13	−1.5149	2.38790	14
−2	−8	−13	6.55321	2.53603	10
2	−8	13	2.23913	2.44904	13
2	8	13	1.88841	3.12966	13
−2	8	−13	2.09110	2.09041	12
−2	8	−13	4.02118	2.50008	14
−3	−8	−13	12.8942	2.56331	10
3	−8	13	12.0869	2.47685	13
−3	−8	−13	10.6865	2.01173	12
3	8	13	12.7908	3.08573	13
−3	8	−13	13.5234	2.41279	12
−3	8	−13	16.5904	2.57114	14
7	−9	−13	9.11734	2.41790	13
7	9	−13	6.08835	2.55258	10
−7	9	13	7.70138	1.83328	10
6	−9	−13	9.02481	2.89419	13
6	9	−13	12.0090	2.72876	10
−6	9	13	8.76111	2.57276	10

5	−9	−13	50.8999	5.91831	13
5	9	−13	40.8810	5.64217	10
−5	9	13	44.2882	5.09122	10
−4	−9	13	74.1244	6.73648	13
4	9	−13	72.9488	6.85652	10
−4	9	13	64.9204	7.43887	10
−3	−9	13	49.9322	6.34794	13
−3	9	13	63.1699	7.13182	10
3	9	−13	46.1175	5.68556	10
−2	−9	13	11.5430	3.57832	13
2	9	−13	14.0064	3.03271	10
−2	9	13	19.5384	4.24477	10
−1	−9	13	18.2734	4.06996	13
1	9	−13	18.2202	3.20950	10
0	−9	13	1.40125	3.00153	13
0	−9	−13	2.33744	2.89154	10
0	9	−13	1.15359	2.55046	14
0	9	−13	3.18439	1.45855	11
1	−9	13	0.72147	2.65126	13
−1	−9	−13	−0.5870	2.01218	10
−1	9	−13	0.01592	2.60825	14
2	−9	13	0.03994	2.30191	13
−2	−9	−13	0.30011	2.38120	10
−2	9	−13	2.07651	2.39954	14
−2	9	−13	0.54868	2.01135	12
−3	−9	−13	1.59961	1.71167	10
−3	−9	−13	2.54308	1.46600	12
−3	9	−13	3.18510	1.76391	12
−3	9	−13	5.16837	1.98915	14
7	−10	−13	16.1655	3.04362	13
−7	10	13	17.4291	1.87914	11
7	10	−13	26.4321	4.13693	10
6	−10	−13	34.4980	4.41217	13
6	10	−13	22.0341	3.60973	10
−6	10	13	32.0068	3.71973	10
5	−10	−13	41.4632	5.12891	13
−5	10	13	39.6518	5.11018	10
−4	−10	13	37.5777	5.16020	13
4	−10	−13	37.5265	4.94186	13
4	10	−13	35.7477	4.98323	10
−4	10	13	40.8274	4.95707	10
−3	−10	13	45.8585	6.09441	13
−3	10	13	35.5703	6.17562	10
3	10	−13	47.0483	5.12856	10
−2	−10	13	5.79672	3.88478	13
−2	10	13	19.9124	4.38446	10
2	10	−13	9.35915	2.68175	10
1	−10	−13	13.6713	3.00343	10
−1	10	13	14.3767	3.49364	10
0	−10	13	73.9696	7.83427	13

0	−10	−13	77.3887	6.73004	10
0	10	−13	75.1908	5.37201	11
0	10	−13	70.6335	6.81096	14
−1	−10	−13	9.07348	3.08803	10
1	−10	13	17.8164	3.27874	13
−1	10	−13	14.8533	3.04673	14
2	−10	13	7.20384	2.41915	13
−2	−10	−13	1.93527	2.38735	10
−2	10	−13	9.38043	2.46787	14
−2	10	−13	8.15568	2.09846	12
−3	−10	−13	7.77409	2.15546	12
−3	−10	−13	5.94695	1.52752	10
−3	10	−13	8.21733	2.11987	14
−3	10	−13	7.73305	1.85904	12
7	−11	−13	39.1326	4.21624	13
6	−11	−13	7.97023	2.54473	13
−6	11	13	8.72593	2.11120	10
6	11	−13	6.64081	2.69083	10
5	−11	−13	12.7912	3.00328	13
−5	11	13	10.8531	2.89264	10
4	−11	−13	11.9088	3.14212	13
−4	−11	13	12.4564	3.59593	13
−4	11	13	11.0158	3.68941	10
4	11	−13	11.7437	2.77250	10
−3	−11	13	47.7398	6.48532	13
−3	11	13	29.0278	4.68734	10
3	11	−13	40.0815	4.63326	10
−2	−11	13	30.9595	4.70210	13
−2	11	13	24.6773	4.46947	10
2	11	−13	31.4296	3.80648	10
−1	−11	13	41.0661	5.73142	13
1	11	−13	42.5991	4.21486	11
1	11	−13	45.6207	5.57807	14
−1	11	13	57.1893	5.85722	10
0	−11	13	60.8386	6.59021	13
0	−11	−13	59.5726	5.84696	10
0	11	−13	50.6707	6.24159	14
0	11	−13	57.7397	4.16755	11
0	11	−13	54.9395	4.17271	11
−1	−11	−13	40.3420	5.15344	10
1	−11	13	52.6045	5.49136	13
−1	11	−13	39.5735	4.93495	14
−2	−11	−13	3.15105	2.34945	10
2	−11	13	5.64534	2.63362	13
−2	11	−13	4.59950	2.25425	14
−2	11	−13	5.33546	1.87718	12
−3	−11	−13	4.05405	1.69159	10
−3	11	−13	2.66120	2.00756	12
−3	11	−13	2.11965	1.73188	14
6	−12	−13	8.60859	2.36556	13

−6	12	13	6.44206	1.85617	10
6	12	−13	9.74949	2.19774	10
5	−12	−13	22.6884	4.24273	13
−5	12	13	27.8114	3.88041	10
4	−12	−13	3.36811	2.44704	13
−4	12	13	6.31650	2.37201	10
−3	12	13	25.9266	4.33116	10
3	12	−13	22.8154	3.92848	10
−2	−12	13	14.6725	3.53499	13
−2	12	13	22.4344	4.50996	10
2	12	−13	22.2406	3.46809	10
1	12	−13	98.4626	7.01604	11
1	12	−13	96.6249	8.05516	14
0	−12	−13	28.8692	3.94637	10
0	−12	13	24.7973	4.70384	13
0	12	−13	24.5322	3.49138	14
−1	−12	−13	18.0216	3.17959	10
1	−12	13	19.9317	3.54060	13
−1	12	−13	15.9671	2.94176	14
−2	−12	−13	12.8767	2.69020	10
−2	12	−13	10.2171	2.43251	14
6	−13	−13	14.4534	2.50833	13
−6	13	13	14.3579	1.68297	11
6	13	−13	8.51995	2.59940	10
5	−13	−13	22.8324	3.80107	13
−5	13	13	22.5691	3.25916	10
5	13	−13	16.4273	3.15496	10
4	−13	−13	24.2714	3.70046	13
−4	−13	13	27.3934	4.27817	13
4	13	−13	23.9911	3.92183	10
−4	13	13	27.3895	4.44341	10
−3	−13	13	11.6322	2.95388	13
3	13	−13	10.6990	2.54189	10
−2	−13	13	28.8157	5.18212	13
−2	13	13	29.6060	5.03152	10
2	13	−13	19.7240	2.70004	11
−1	−13	13	7.56375	2.84891	13
1	13	−13	9.99967	2.70394	14
−1	13	13	12.3379	3.71496	10
1	13	−13	9.93160	1.49959	11
0	−13	13	30.3748	4.53951	13
−1	−13	−13	3.55626	1.96580	10
1	−13	13	2.95123	2.54933	13
−1	13	−13	4.00741	1.70641	14
−2	−13	−13	5.24233	1.78219	10
−2	13	−13	7.32096	2.11316	14
6	−14	−13	2.74679	2.33207	13
5	−14	−13	18.7007	3.35060	13
5	14	−13	14.1196	2.62627	10
−5	14	13	19.8890	2.95548	10

4	−14	−13	11.4379	2.73516	13
−4	−14	13	7.47437	2.28297	13
4	14	−13	8.06886	2.34172	10
−4	14	13	10.2180	2.75323	10
−3	−14	13	5.11517	2.50461	13
−3	14	13	3.20463	2.89486	10
−2	−14	13	1.84756	2.47608	13
2	14	−13	−0.2076	1.72923	11
−2	14	13	1.65234	2.89259	10
−1	−14	13	12.4495	2.95768	13
−1	14	13	13.9925	3.24500	10
1	14	−13	11.2120	2.67337	14
0	−14	13	9.12402	2.51143	13
0	14	−13	10.6630	2.51629	14
0	14	13	6.48777	2.47688	10
1	−14	13	24.3928	4.08865	13
−1	−14	−13	18.6354	3.03233	10
−1	14	−13	22.1056	3.68598	14
−2	14	−13	0.88700	1.31566	14
5	−15	−13	16.7839	2.47751	13
5	15	−13	16.0293	2.93576	10
4	−15	−13	1.64790	1.78092	13
−4	−15	13	−0.0458	1.73361	13
−4	15	13	0.66256	1.82298	10
4	15	−13	3.24889	2.10494	10
−3	−15	13	21.7893	3.12151	13
3	15	−13	18.7478	2.33270	11
−3	15	13	15.8568	3.11484	10
−2	−15	13	20.6406	3.17024	13
−2	15	13	14.0694	3.47905	10
2	15	−13	22.0976	2.38456	11
−1	−15	13	1.71677	2.16676	13
1	15	−13	1.95793	2.03914	14
−1	15	13	−0.3847	1.96676	10
0	−15	13	12.2659	2.47161	13
0	15	−13	15.2914	2.57547	14
0	15	13	11.4036	2.67133	10
1	−15	13	19.5224	3.56512	13
−1	15	−13	17.4515	2.57881	14
4	−16	−13	1.84425	1.59257	13
−4	−16	13	1.97353	2.01414	13
−4	16	13	0.68131	1.53285	10
−3	−16	13	7.67507	2.21824	13
−3	16	13	11.4311	2.73117	10
3	16	−13	7.16787	1.65087	11
−2	−16	13	3.49942	2.11029	13
−2	16	13	7.15196	2.19845	10
−1	−16	13	9.37922	2.36689	13
−1	16	13	12.7071	2.66913	10
1	16	−13	10.7071	2.22774	14

0	−16	13	4.35915	1.90578	13
0	16	13	3.46332	2.67594	10
0	16	−13	5.29510	1.89926	14
−4	−17	13	2.01525	1.59155	13
−3	−17	13	7.77118	2.00723	13
3	17	−13	8.98400	1.15317	11
3	17	−13	9.95874	1.44182	11
−3	17	13	7.88289	2.16629	10
−2	−17	13	4.22044	1.92224	13
−2	17	13	3.08327	2.33635	10
−1	−17	13	6.06767	2.28952	13
−1	17	13	2.23096	1.72153	10
0	−17	13	10.4680	2.36614	13
0	17	−13	10.8473	2.03787	14
−7	0	14	18.3602	3.66420	10
−6	0	14	2.89366	2.76500	10
−5	0	14	3.05097	2.13399	10
4	0	−14	15.1702	3.83951	10
3	0	−14	4.69948	3.09927	10
2	0	−14	5.95082	2.31282	10
−2	0	14	3.45190	3.04254	13
1	0	−14	59.1619	6.24449	10
−1	0	14	59.9062	7.67601	13
0	0	−14	203.948	14.1869	10
0	0	−14	207.340	13.8060	8
0	0	14	203.114	14.6908	13
−1	0	−14	1.75822	2.76010	14
−1	0	−14	4.83353	2.42943	12
1	0	14	−3.6765	3.20900	13
−1	0	−14	0.71510	2.07342	10
−1	0	−14	0.01775	2.01637	8
2	0	14	−0.3526	2.86567	13
−2	0	−14	−2.3059	2.16506	14
−2	0	−14	−2.0780	1.42332	12
−2	0	−14	0.76528	1.32720	11
−2	0	−14	1.31211	1.22197	8
3	0	14	0.13217	2.13452	13
−3	0	−14	0.82270	1.69141	12
−7	1	14	15.4989	3.00068	10
−6	−1	14	0.14288	1.88789	10
−6	1	14	−0.2305	1.86120	10
−5	−1	14	16.3900	3.23787	10
5	1	−14	14.5863	2.97771	10
4	−1	−14	4.51187	3.41423	10
4	1	−14	4.38922	3.17890	10
3	−1	−14	30.4703	5.20085	10
3	1	−14	35.2013	5.17087	10
−2	−1	14	78.1951	7.43676	13
2	−1	−14	61.3810	6.92905	10
2	1	−14	78.9173	7.16368	10

1	−1	−14	166.591	12.0731	10
−1	−1	14	170.524	12.7280	13
1	1	−14	169.882	12.3122	10
−1	1	14	169.342	12.5143	13
0	−1	−14	128.969	9.27479	8
0	−1	14	131.047	10.3898	13
0	−1	−14	128.463	9.68054	10
0	1	−14	121.635	9.52909	10
−1	−1	−14	27.9427	3.86218	8
−1	−1	−14	21.5396	3.79115	10
1	−1	14	31.2338	4.63056	13
−1	1	−14	30.0208	3.71077	10
−1	1	−14	33.1380	3.83494	8
−1	1	−14	30.9536	4.01378	14
−1	1	−14	32.0483	4.30844	12
1	1	14	26.9019	4.49664	13
−2	−1	−14	−0.3565	1.61727	10
−2	−1	−14	0.86063	1.22442	8
−2	−1	−14	−0.6482	1.94599	12
−2	−1	−14	−0.8443	2.20164	14
2	−1	14	−1.0384	2.73790	13
−2	1	−14	0.05644	1.49222	12
−2	1	−14	−1.1980	1.48760	8
−2	1	−14	5.66757	2.27480	14
−2	1	−14	−1.8099	1.10358	11
2	1	14	−1.7432	2.77433	13
3	−1	14	14.0882	2.68401	13
−3	−1	−14	13.5700	1.05196	11
−3	−1	−14	11.6217	1.03250	11
−3	−1	−14	18.1245	2.90393	12
3	1	14	14.4199	3.09258	13
−7	−2	14	66.3610	5.93535	10
−7	2	14	59.0099	5.65292	10
−6	−2	14	17.2065	3.35169	10
−6	2	14	19.8086	3.44019	10
5	2	−14	12.3943	3.41755	10
−5	2	14	18.9443	3.37823	10
4	2	−14	33.3890	5.39860	10
−4	2	14	42.5975	5.80206	10
3	−2	−14	13.0752	3.60523	10
3	2	−14	12.4814	3.35265	10
2	−2	−14	19.4190	3.32869	10
−2	−2	14	19.0964	3.85669	13
2	2	−14	17.9046	3.69401	10
1	−2	−14	32.2595	4.63344	10
−1	−2	14	32.2853	5.87114	13
−1	2	14	26.2953	4.16332	13
1	2	−14	22.4878	4.34688	10
0	−2	−14	39.9496	4.35279	8
0	−2	14	38.0607	5.90276	13

0	−2	−14	41.5345	4.76698	10
0	2	−14	32.3622	4.93664	14
0	2	−14	38.5318	4.38028	12
0	2	−14	31.2685	4.32360	10
0	2	14	36.3841	5.65637	13
−1	−2	−14	23.9417	3.72878	8
−1	−2	−14	24.6963	3.15613	10
1	−2	14	13.7072	4.01293	13
−2	−2	−14	4.80534	2.18839	12
−2	−2	−14	6.46667	1.93393	8
−2	−2	−14	6.03106	2.48899	14
2	−2	14	7.56344	3.05536	13
−2	−2	−14	3.92753	1.64279	10
−2	2	−14	7.60587	2.27996	12
2	2	14	0.59571	2.80358	13
−2	2	−14	5.70400	1.34593	11
−2	2	−14	6.18517	2.37268	14
−3	−2	−14	11.3201	2.10780	12
3	−2	14	10.0897	2.85808	13
−3	−2	−14	10.5024	1.20400	11
−3	−2	−14	11.8642	1.49972	11
3	2	14	7.74467	2.41540	13
−3	2	−14	8.48854	2.06456	12
−3	2	−14	10.6976	2.07032	14
−7	−3	14	15.7354	2.79675	10
−6	−3	14	16.6539	3.08256	10
−6	3	14	17.7935	3.09249	10
5	3	−14	1.44467	3.04335	10
−5	3	14	5.52031	3.17812	10
−4	3	14	3.28843	3.31906	10
4	3	−14	1.36277	2.77669	10
3	−3	−14	1.23532	2.14330	10
−3	−3	14	−0.5328	3.46900	13
3	3	−14	−1.9246	2.69818	10
2	−3	−14	40.4955	5.17357	10
−2	−3	14	29.7951	5.55273	13
2	3	−14	28.2161	4.63269	10
2	3	−14	28.0945	3.79401	14
−1	−3	14	61.5718	7.22164	13
1	−3	−14	74.9345	6.09100	10
−1	3	14	73.5913	6.88832	13
1	3	−14	51.1624	5.96582	10
1	3	−14	51.2470	6.31103	14
0	−3	14	22.9709	4.15874	13
0	3	−14	25.0897	3.63414	10
0	3	−14	26.8938	3.78928	12
0	3	−14	23.0147	3.45432	14
−1	−3	−14	6.21932	1.80948	8
−1	−3	−14	5.58915	2.55376	10
1	−3	14	11.9522	3.56842	13

−1	3	−14	14.9227	2.64904	12
−1	3	−14	11.0906	2.02260	11
−1	3	−14	10.2085	2.51086	10
−1	3	−14	10.9949	2.90297	14
−2	−3	−14	5.70411	1.93315	8
−2	−3	−14	6.77116	1.72029	12
−2	−3	−14	6.09545	1.75551	10
2	−3	14	3.34824	2.66531	13
−2	3	−14	5.54445	2.29166	14
2	3	14	6.51392	2.84724	13
−2	3	−14	3.54604	2.19157	12
−2	3	−14	4.59103	0.97226	11
−2	3	−14	5.00242	0.99877	11
−3	−3	−14	4.05169	1.70813	12
3	−3	14	3.49420	2.13284	13
−3	3	−14	1.95679	1.76801	14
3	3	14	3.01716	2.16159	13
−3	3	−14	3.92268	1.79412	12
−7	−4	14	1.04434	1.57756	10
−7	4	14	0.36526	1.97896	10
−6	−4	14	6.29296	2.37504	10
−6	4	14	1.49560	1.99024	10
6	4	−14	3.85088	2.22783	10
−5	4	14	7.53318	3.33304	10
5	4	−14	12.0597	2.97005	10
−4	4	14	2.68427	3.27062	10
4	4	−14	11.5474	3.29274	10
3	−4	−14	60.8208	6.74011	10
2	−4	−14	7.13723	3.12170	10
−2	−4	14	5.71962	3.42987	13
2	4	−14	4.68459	2.83798	10
2	4	−14	6.48190	2.67728	14
−1	−4	14	33.6158	6.38107	13
1	4	−14	38.8902	4.85162	10
1	4	−14	47.6449	5.43126	14
0	−4	14	195.638	13.7146	13
0	−4	−14	149.397	12.5742	10
0	4	14	154.135	12.7161	13
0	4	−14	197.204	12.3436	10
0	4	−14	197.979	12.2905	12
0	4	−14	184.026	12.2490	14
1	−4	14	6.28413	3.16031	13
−1	4	−14	5.27341	2.45220	14
−1	4	−14	4.54592	1.56960	11
−1	4	−14	4.73889	1.77755	12
1	4	14	11.9563	3.22883	13
−2	−4	−14	15.0897	2.44278	8
2	−4	14	15.6486	3.11864	13
−2	−4	−14	14.3355	2.62287	10
−2	−4	−14	21.3349	3.26243	12

−2	4	−14	25.1326	3.89862	14
2	4	14	15.4270	3.13128	13
−2	4	−14	19.1931	2.79504	12
3	−4	14	1.55160	2.40278	13
−3	−4	−14	1.50535	1.55133	12
3	4	14	0.30427	1.99515	13
−3	4	−14	4.96268	1.76934	14
−3	4	−14	4.13705	1.74925	12
−7	−5	14	5.56733	1.90059	10
−7	5	14	3.77688	1.98277	10
−6	5	14	8.56238	2.89195	10
6	5	−14	10.9260	2.51019	10
−5	5	14	39.0501	5.41921	10
4	5	−14	24.9026	3.82556	10
−4	5	14	21.2721	4.19427	10
−2	−5	14	7.46435	4.04267	13
2	−5	−14	0.44074	2.84883	10
2	5	−14	4.71294	2.27239	10
2	5	−14	10.5060	3.11791	14
1	−5	−14	77.3341	6.13704	10
−1	−5	14	49.6150	7.07155	13
1	5	−14	61.4817	5.83052	10
1	5	−14	58.6890	6.04239	14
0	−5	14	91.1147	8.20538	13
0	−5	−14	79.2428	6.88302	10
0	5	−14	85.3630	6.68385	10
0	5	14	76.5917	8.54469	13
0	5	−14	89.3134	7.02354	14
−1	−5	−14	2.17800	1.69761	10
1	−5	14	1.89431	2.76078	13
−1	−5	−14	2.75256	2.09324	8
−1	5	−14	5.78201	1.54795	11
−1	5	−14	2.23905	2.28686	14
−1	5	−14	7.73118	2.22700	12
1	5	14	2.13118	2.71550	13
−2	−5	−14	25.2427	3.06119	10
−2	−5	−14	24.5592	3.25235	8
2	−5	14	25.9956	4.48897	13
2	5	14	23.5094	3.83674	13
−2	5	−14	21.2294	2.96151	12
−2	5	−14	20.4622	3.08274	14
−3	−5	−14	4.90126	1.64811	12
3	−5	14	8.18676	2.17017	13
−3	5	−14	9.74075	1.93640	12
−3	5	−14	8.95277	1.92326	14
3	5	14	3.97026	2.07505	13
−7	6	14	9.78731	2.15554	10
6	6	−14	12.0785	3.07163	10
−6	6	14	15.8943	2.90375	10
5	6	−14	89.3764	8.05297	10

−5	6	14	120.978	8.62371	10
−4	6	14	4.23419	3.12850	10
4	6	−14	2.85748	2.78995	10
−3	−6	14	62.7458	6.62149	13
−3	6	14	65.9711	7.25675	10
3	6	−14	57.9047	5.64634	10
2	−6	−14	18.6762	3.63795	10
−2	−6	14	21.0725	3.95487	13
2	6	−14	20.1957	3.15694	14
2	6	−14	16.6634	3.24087	10
1	−6	−14	16.5967	3.41308	10
−1	−6	14	23.4436	4.41748	13
1	6	−14	23.8253	3.54883	10
1	6	−14	23.4554	3.33138	14
0	6	−14	6.14147	2.54420	14
0	6	−14	5.79109	2.18737	10
1	−6	14	2.63913	2.76907	13
−1	−6	−14	6.04452	2.54037	10
−1	6	−14	5.69314	2.09043	12
−1	6	−14	5.45380	1.84724	14
1	6	14	4.91262	3.06827	13
−1	6	−14	5.08831	1.18785	11
−1	6	−14	6.64772	1.33567	11
−2	−6	−14	−0.8503	1.59539	10
2	−6	14	−0.4974	2.79721	13
−2	6	−14	2.74321	2.13130	14
−2	6	−14	0.72924	1.82891	12
2	6	14	−0.7210	2.23973	13
3	−6	14	9.04643	2.24673	13
−3	−6	−14	12.3150	1.94480	12
−3	6	−14	13.5751	2.25896	14
−3	6	−14	12.4765	2.05037	12
−7	7	14	2.24717	3.05189	10
6	7	−14	3.58453	1.91473	10
−6	7	14	6.31327	1.96738	10
5	7	−14	19.3306	3.57357	10
−4	7	14	0.18786	3.29679	10
4	7	−14	1.43527	2.23275	10
−3	−7	14	10.1019	3.15828	13
3	7	−14	10.4685	3.00630	10
−2	−7	14	58.1466	6.66524	13
2	−7	−14	60.8888	6.28609	10
2	7	−14	58.7427	5.72534	10
1	−7	−14	17.8822	3.33074	10
−1	−7	14	22.3104	4.28448	13
1	7	−14	14.2290	2.93024	14
1	7	−14	11.3321	2.56465	10
0	−7	14	10.2553	3.76769	13
0	7	−14	17.7303	2.76364	14
0	7	−14	24.7558	2.63098	11

−1	−7	−14	19.9745	3.64314	10
1	−7	14	18.7066	3.63395	13
−1	7	−14	18.3365	2.67749	14
−1	7	−14	18.7141	3.17772	12
2	−7	14	3.67204	2.75319	13
−2	−7	−14	0.38177	1.72343	10
−2	7	−14	2.06902	2.02600	14
−2	7	−14	2.96411	1.72286	12
6	8	−14	4.53083	1.88362	10
−6	8	14	4.38922	2.20701	10
5	8	−14	−0.5287	2.63597	10
−5	8	14	4.87597	2.72325	10
−4	−8	14	18.5314	5.24273	13
4	8	−14	17.2057	3.20591	10
−4	8	14	15.8879	3.88148	10
−3	−8	14	2.96853	2.86337	13
−3	8	14	1.64399	2.36052	10
3	8	−14	7.18426	2.12221	10
2	−8	−14	4.87983	2.97161	10
−2	−8	14	1.77216	3.03182	13
2	8	−14	1.07506	2.23749	10
−2	8	14	5.25979	2.68750	10
−1	−8	14	−2.0374	3.38404	13
1	−8	−14	2.57955	2.96410	10
1	8	−14	−4.3749	2.55656	14
1	8	−14	−2.1374	2.20607	10
0	−8	14	50.6624	6.21602	13
0	8	−14	52.9152	4.31861	11
0	8	−14	56.0136	5.61222	14
−1	−8	−14	3.62562	2.37487	10
1	−8	14	4.87570	2.76584	13
−1	8	−14	4.14995	1.74330	14
−2	−8	−14	9.61562	1.84377	10
2	−8	14	10.9234	2.56300	13
−2	8	−14	13.1606	2.15319	12
−2	8	−14	14.1543	2.43172	14
−6	9	14	11.6276	2.39549	10
6	9	−14	8.66836	2.63306	10
−5	9	14	6.64486	2.66774	10
−4	−9	14	2.42461	2.74986	13
4	9	−14	4.47528	2.55026	10
−4	9	14	6.16633	2.49279	10
−3	−9	14	45.6323	5.47364	13
−3	9	14	24.8929	4.88766	10
3	9	−14	35.8504	4.54561	10
2	9	−14	31.1355	4.00533	10
−2	9	14	34.7658	5.80286	10
1	−9	−14	3.38064	2.03453	10
−1	−9	14	3.33680	2.94504	13
1	9	−14	−0.5357	1.69271	11

1	9	−14	3.70865	2.56869	14
0	−9	14	11.0236	3.17940	13
0	−9	−14	6.91051	2.70589	10
0	9	−14	6.59724	2.54604	14
−1	−9	−14	1.62672	1.63346	10
1	−9	14	1.89773	2.44811	13
−1	9	−14	−0.0004	2.09992	14
−2	−9	−14	1.81678	1.76061	10
2	−9	14	1.15871	1.93148	13
−2	9	−14	0.95304	1.44424	12
−2	9	−14	1.27454	1.78912	14
6	10	−14	5.49785	1.79559	10
−6	10	14	1.74899	1.64526	10
−5	10	14	24.0848	3.28250	10
5	10	−14	29.0992	4.13158	10
−4	−10	14	5.25855	2.33185	13
−4	10	14	4.55671	3.10650	10
4	10	−14	4.22725	2.44654	10
−3	−10	14	83.2083	7.68088	13
3	10	−14	70.9341	6.40568	10
−3	10	14	82.1298	7.73931	10
−2	−10	14	45.4007	5.71643	13
2	10	−14	45.0230	4.30550	10
−1	−10	14	13.7484	3.27052	13
1	−10	−14	5.74762	2.84552	10
1	10	−14	11.3685	2.77762	14
1	10	−14	13.1802	2.11681	11
0	−10	14	70.5341	7.11291	13
0	−10	−14	83.3314	6.91349	10
0	10	−14	81.7850	6.82315	14
1	−10	14	8.56574	2.57272	13
−1	−10	−14	3.91606	1.72708	10
−1	10	−14	4.99789	2.19862	14
−2	−10	−14	3.97664	1.46040	10
2	−10	14	7.12308	2.05553	13
−2	10	−14	9.99670	2.08997	14
6	11	−14	0.43831	2.22579	10
5	11	−14	13.2393	2.36850	10
−4	−11	14	1.95414	2.02523	13
4	11	−14	2.47973	2.20509	10
−4	11	14	1.90419	2.58656	10
−3	−11	14	1.12063	2.50389	13
3	11	−14	2.06124	2.02807	10
−3	11	14	8.76122	3.44662	10
−2	−11	14	18.6654	3.42731	13
2	11	−14	15.6821	2.93830	10
−2	11	14	26.6502	5.03369	10
1	−11	−14	44.6126	4.58504	10
−1	−11	14	44.3166	5.41979	13
1	11	−14	40.3820	3.70836	11

−1	11	14	46.4931	5.18499	10
1	11	−14	40.2993	5.39093	14
0	−11	14	31.0244	4.79263	13
0	−11	−14	41.0343	4.52718	10
0	11	−14	33.5514	4.36504	14
1	−11	14	24.7298	3.88384	13
−1	−11	−14	29.6683	3.14141	10
−1	11	−14	19.4778	2.91656	14
5	12	−14	12.1505	2.18631	10
−5	12	14	13.9595	2.69221	10
−4	−12	14	9.74453	2.24962	13
4	12	−14	8.75814	2.37282	10
−4	12	14	6.13925	2.62350	10
−3	−12	14	5.46068	2.37903	13
3	12	−14	4.57527	2.25517	10
−2	−12	14	12.2949	3.02296	13
2	12	−14	14.7609	1.94773	11
−2	12	14	7.70556	3.38808	10
−1	−12	14	9.46846	2.65743	13
1	12	−14	7.98821	1.12999	11
−1	12	14	10.1963	2.73931	10
1	12	−14	7.57407	1.10531	11
1	12	−14	9.98995	2.30581	14
0	−12	−14	4.30030	2.33734	10
0	−12	14	5.44306	2.24986	13
0	12	−14	4.12044	2.06337	14
1	−12	14	4.99377	2.29329	13
−1	−12	−14	4.52308	2.04201	10
−1	12	−14	7.23048	2.09977	14
−5	13	14	−0.4967	2.34847	10
5	13	−14	−0.1918	1.74180	10
−4	−13	14	8.02448	1.99674	13
4	13	−14	7.47492	2.26500	10
−4	13	14	10.6402	2.56693	10
−3	−13	14	5.46943	2.64512	13
3	13	−14	4.02434	1.77359	10
−2	−13	14	14.0077	2.78976	13
−2	13	14	9.47992	2.63647	10
2	13	−14	10.4791	1.50824	11
−1	−13	14	0.78133	2.14414	13
1	13	−14	0.94648	2.27438	14
−1	13	14	1.24849	2.77272	10
0	−13	14	9.17037	2.35099	13
0	13	−14	9.28319	2.24097	14
−1	13	−14	10.2626	2.08897	14
−4	−14	14	20.9735	3.32658	13
−4	14	14	15.7156	2.94002	10
−3	−14	14	20.7271	3.43152	13
−3	14	14	15.5653	2.92676	10
3	14	−14	19.2533	2.50029	11

−2	−14	14	9.70008	2.29428	13
−2	14	14	6.77161	2.23050	10
2	14	−14	5.23569	0.94127	11
2	14	−14	4.90017	0.88763	11
−1	−14	14	6.34757	2.22699	13
−1	14	14	3.13171	2.59641	10
1	14	−14	6.23875	1.84927	14
0	−14	14	5.42896	1.94729	13
0	14	−14	6.20995	1.88846	14
−3	−15	14	34.9779	3.77122	13
−3	15	14	30.4404	3.93438	10
3	15	−14	32.8935	2.71547	11
−2	−15	14	8.25214	2.13355	13
−1	−15	14	10.9431	2.17881	13
−1	15	14	2.44418	2.73701	10
−6	0	15	−0.1436	1.61340	10
−5	0	15	11.7136	3.25333	10
5	0	−15	13.5012	3.25708	10
4	0	−15	−0.6137	2.63221	10
3	0	−15	7.42979	3.19772	10
2	0	−15	9.43026	2.59789	10
−1	0	15	−1.6054	2.71458	13
1	0	−15	1.15910	1.70402	10
0	0	15	2.54271	2.87499	13
0	0	−15	6.18559	2.19285	10
0	0	−15	2.79324	2.32831	14
0	0	−15	3.22533	2.04163	12
1	0	15	12.4878	3.33477	13
−1	0	−15	12.7281	2.34623	12
−1	0	−15	11.6778	2.09141	10
−2	0	−15	19.9747	1.76317	11
−2	0	−15	20.8010	2.92748	12
2	0	15	22.8293	3.67988	13
−2	0	−15	21.5477	1.72590	11
−6	−1	15	28.6512	4.10587	10
−6	1	15	23.1111	4.01677	10
5	1	−15	7.94146	2.87664	10
−5	1	15	1.29107	2.00814	10
4	−1	−15	0.60486	2.62217	10
4	1	−15	5.21235	2.78035	10
3	−1	−15	27.5301	4.84874	10
−2	−1	15	50.1222	6.51655	13
2	1	−15	40.6694	5.12691	14
2	1	−15	44.2555	5.34409	10
1	−1	−15	19.3587	3.83440	10
−1	−1	15	15.0426	3.97101	13
1	1	−15	11.9778	2.93312	10
−1	1	15	21.8306	3.52093	13
1	1	−15	12.5003	2.94229	14
0	−1	−15	24.4858	3.62403	10

0	−1	15	23.1481	3.84312	13
0	−1	−15	24.5765	3.40219	14
0	1	−15	25.2380	3.38960	10
0	1	−15	26.1549	3.49972	12
0	1	15	19.4422	3.59414	13
0	1	−15	26.8912	4.22128	14
−1	−1	−15	22.0283	2.85882	10
1	−1	15	27.8935	4.43746	13
−1	−1	−15	25.1558	3.41028	12
−1	1	−15	27.2026	3.75051	14
−1	1	−15	22.3669	2.81999	11
1	1	15	30.0330	4.37081	13
−1	1	−15	27.1542	3.35166	12
2	−1	15	10.5332	2.40130	13
−2	−1	−15	12.1775	1.65387	11
−2	−1	−15	11.8625	2.09830	12
−2	1	−15	12.8646	2.15409	14
−2	1	−15	14.0206	2.16614	12
2	1	15	12.5453	2.50993	13
−6	−2	15	55.6046	5.28178	10
−6	2	15	62.5831	5.78803	10
5	2	−15	14.0504	2.71433	10
−5	2	15	14.2490	3.34260	10
4	−2	−15	7.83064	2.33112	10
4	2	−15	3.82839	2.78459	10
3	−2	−15	25.3383	3.74529	10
3	2	−15	18.4156	3.39075	10
2	−2	−15	36.6645	4.29543	10
−2	−2	15	30.5020	5.22199	13
2	2	−15	36.8111	4.68346	14
−1	−2	15	19.6957	3.87540	13
1	2	−15	26.4334	3.88338	10
1	2	−15	29.6975	4.33843	14
0	−2	−15	12.3871	2.75435	10
0	−2	15	7.40892	3.21594	13
0	2	−15	11.5930	2.24974	12
0	2	−15	10.3187	2.54671	10
0	2	15	7.83001	2.91781	13
0	2	−15	11.4241	2.66084	14
1	−2	15	7.66148	2.81767	13
−1	−2	−15	3.98069	1.53157	12
−1	−2	−15	4.33060	1.77549	10
−1	−2	−15	4.66671	2.22446	14
−1	2	−15	9.81835	2.09950	12
1	2	15	3.08600	2.43101	13
−2	−2	−15	7.99179	1.28805	11
−2	−2	−15	7.75659	1.88607	12
2	−2	15	6.68962	2.23415	13
−2	2	−15	5.95646	1.79632	12
2	2	15	7.76011	2.24060	13

−2	2	−15	9.20389	1.99360	14
−6	−3	15	14.0267	2.80948	10
6	3	−15	13.5157	2.88345	10
−6	3	15	20.3515	3.60523	10
5	3	−15	24.0374	3.51450	10
−5	3	15	25.7540	4.34228	10
4	−3	−15	1.45151	1.98961	10
−4	3	15	1.19763	2.02986	10
4	3	−15	4.23088	2.75849	10
3	−3	−15	27.6115	4.72437	10
3	3	−15	27.3029	4.44243	10
2	−3	−15	9.19892	3.09146	10
−2	−3	15	4.76213	3.52738	13
2	3	−15	6.14438	2.43927	14
2	3	−15	4.05186	2.65442	10
−1	−3	15	4.28650	3.13506	13
1	−3	−15	1.85421	1.77992	10
1	3	−15	5.50512	2.33347	10
1	3	−15	4.11261	2.34760	14
0	−3	15	12.3415	3.43774	13
0	−3	−15	15.4534	2.96234	10
0	3	−15	14.0894	2.69698	14
0	3	15	12.0476	3.48003	13
0	3	−15	14.8005	2.45488	12
0	3	−15	13.2804	2.53148	10
−1	−3	−15	7.45507	1.71664	8
−1	−3	−15	10.1795	2.10656	10
−1	−3	−15	10.0075	2.11161	12
1	−3	15	10.6766	2.93495	13
−1	3	−15	11.3870	2.15842	12
−1	3	−15	11.3012	1.49826	11
1	3	15	7.61730	2.93995	13
−1	3	−15	9.63673	2.28305	14
2	−3	15	1.52179	1.99174	13
−2	−3	−15	0.41486	1.47498	12
−2	3	−15	1.19993	1.71365	14
2	3	15	0.05353	1.93013	13
−2	3	−15	0.07098	1.52633	12
−6	4	15	5.12950	2.39828	10
6	4	−15	10.4782	2.63043	10
−5	4	15	0.44057	2.48102	10
5	4	−15	−0.3046	2.38931	10
4	4	−15	1.59038	1.85960	10
−4	4	15	0.54677	2.75094	10
3	−4	−15	0.64401	1.98383	10
3	4	−15	10.2161	2.33544	10
−2	−4	15	−1.5278	3.33615	13
2	4	−15	1.31058	2.16957	14
2	4	−15	1.93674	2.31585	10
1	−4	−15	1.80973	2.50927	10

−1	−4	15	3.96500	3.70341	13
1	4	−15	3.12777	2.27525	14
1	4	−15	4.46413	2.07881	10
0	−4	15	19.5313	3.84296	13
0	4	−15	24.6179	3.83543	14
0	4	−15	25.0446	3.14288	11
0	4	−15	20.7441	2.90559	10
1	−4	15	11.6385	2.88621	13
−1	−4	−15	9.25536	2.09413	10
1	4	15	7.44379	2.75409	13
−1	4	−15	11.4029	2.40463	12
−1	4	−15	11.3411	2.39170	14
−1	4	−15	10.3147	1.29571	11
−1	4	−15	10.4641	1.21289	11
−2	−4	−15	2.51734	1.55763	12
2	−4	15	4.55747	2.08951	13
−2	4	−15	4.90654	1.70065	12
−2	4	−15	7.63039	1.85518	14
6	5	−15	19.4378	3.47683	10
5	5	−15	4.42064	1.95645	10
−5	5	15	0.61635	2.49576	10
4	5	−15	17.8614	3.43930	10
3	−5	−15	54.1055	5.42297	10
−3	−5	15	56.3962	5.83856	13
3	5	−15	59.1069	5.30695	10
−2	−5	15	28.5521	4.97114	13
2	5	−15	30.1414	4.02848	10
2	5	−15	23.6883	4.04437	14
−1	−5	15	0.94152	3.08959	13
1	−5	−15	4.73229	1.96006	10
1	5	−15	0.75620	1.79567	10
1	5	−15	4.97640	2.26947	14
0	−5	−15	6.72421	1.87431	10
0	−5	15	1.76948	2.84122	13
0	5	−15	2.66263	2.14869	14
0	5	−15	3.35044	1.71351	11
−1	−5	−15	14.4937	2.64149	10
1	−5	15	11.7761	2.86172	13
−1	5	−15	12.9792	2.42032	14
−1	5	−15	13.8257	2.40664	12
2	−5	15	6.26209	2.05995	13
−2	5	−15	6.46965	1.94812	14
6	6	−15	34.7938	3.52508	10
−6	6	15	29.1689	3.35292	10
−5	6	15	15.7461	2.93324	10
5	6	−15	12.9507	2.91262	10
4	6	−15	5.44149	2.69426	10
−4	6	15	11.3782	2.66606	10
−3	−6	15	24.8787	3.97089	13
3	−6	−15	33.8760	4.74717	10

3	6	−15	22.5680	4.10252	10
−3	6	15	35.1579	5.24441	10
−2	−6	15	26.9835	4.77197	13
2	−6	−15	31.5167	3.85957	10
2	6	−15	29.7209	4.59866	14
2	6	−15	29.2411	3.87763	10
−1	−6	15	8.53254	3.11646	13
1	6	−15	8.68592	2.45292	10
1	6	−15	16.9462	2.80388	14
0	−6	15	12.2063	3.16795	13
0	−6	−15	19.0549	3.28537	10
0	6	−15	11.6944	1.59550	11
0	6	−15	11.5227	2.52927	14
−1	−6	−15	7.91036	2.02310	10
1	−6	15	8.53108	2.53673	13
−1	6	−15	7.17695	2.12730	14
−1	6	−15	8.76162	1.81722	12
−6	7	15	3.73843	1.93620	10
−5	7	15	21.6750	3.75151	10
5	7	−15	23.1183	3.70891	10
−4	7	15	3.60664	2.84838	10
4	7	−15	10.2504	2.66478	10
−3	−7	15	4.73575	2.55010	13
−3	7	15	10.0879	3.42413	10
3	7	−15	5.53773	2.55695	10
2	−7	−15	26.6280	3.75145	10
−2	−7	15	34.2084	5.45659	13
2	7	−15	23.6592	4.01961	10
−1	−7	15	1.78611	2.62509	13
1	−7	−15	1.91813	1.71472	10
1	7	−15	4.38207	2.26164	14
1	7	−15	1.13148	1.48005	10
1	7	−15	1.61402	1.51981	11
0	−7	15	0.25726	2.58661	13
0	−7	−15	4.59404	2.20005	10
0	7	−15	2.52113	1.09734	11
0	7	−15	1.08593	1.04081	11
0	7	−15	1.12204	2.09184	14
1	−7	15	3.32442	2.62997	13
−1	−7	−15	3.05831	1.77255	10
−1	7	−15	4.25736	1.94480	14
5	8	−15	6.49810	2.38142	10
−5	8	15	9.91875	2.53058	10
−4	8	15	15.6799	3.09699	10
4	8	−15	9.93248	2.68474	10
−3	−8	15	4.40461	2.32093	13
−3	8	15	3.77375	2.24999	10
3	8	−15	3.17738	2.07994	10
−2	−8	15	9.10859	2.71472	13
2	−8	−15	5.09920	2.58823	10

2	8	-15	5.43731	1.85574	10
-1	-8	15	14.4915	3.13088	13
1	8	-15	16.5988	1.98668	11
1	8	-15	17.0841	2.75176	14
0	-8	15	2.13021	2.81510	13
0	-8	-15	1.51322	1.47122	10
0	8	-15	1.40499	1.45521	14
-1	-8	-15	9.74883	1.73678	10
1	-8	15	6.69986	2.70682	13
-1	8	-15	8.17662	2.02359	14
-5	9	15	6.83739	1.83214	10
4	9	-15	14.4216	2.41084	10
-3	-9	15	44.8242	4.79141	13
3	9	-15	43.3958	4.19493	10
-3	9	15	40.3347	4.73440	10
-2	-9	15	17.4143	3.11736	13
2	-9	-15	13.9216	2.97659	10
2	9	-15	17.3073	2.72674	10
-2	9	15	16.0049	3.61943	10
-1	-9	15	7.32130	2.53143	13
1	-9	-15	3.61680	1.76208	10
1	9	-15	8.49817	1.37896	11
1	9	-15	7.88641	2.15909	14
0	-9	15	5.40262	2.80458	13
0	-9	-15	4.17544	2.08762	10
0	9	-15	4.94650	1.94752	14
1	-9	15	29.5155	3.89809	13
-1	9	-15	33.1493	3.47852	14
-5	10	15	4.86246	1.99778	10
5	10	-15	3.77668	2.00499	10
-4	10	15	7.38294	2.50595	10
4	10	-15	4.62323	1.96965	10
-3	-10	15	27.1835	3.88672	13
3	10	-15	29.5841	3.46071	10
-3	10	15	29.4649	3.86823	10
-2	-10	15	7.10046	2.49025	13
-2	10	15	5.64451	2.96349	10
2	10	-15	9.29549	1.57316	11
-1	-10	15	8.03401	2.49133	13
1	-10	-15	9.62860	2.03954	10
1	10	-15	8.61027	1.13080	11
1	10	-15	9.68317	2.04504	14
1	10	-15	8.84923	1.08235	11
0	-10	-15	6.83284	2.09588	10
0	-10	15	5.34476	2.14191	13
0	10	-15	7.66324	1.95834	14
-4	11	15	5.95176	1.79031	10
4	11	-15	4.77606	1.76584	10
-3	-11	15	11.1499	2.62953	13
-3	11	15	10.0312	2.89647	10

3	11	−15	10.6507	1.92986	10
−2	−11	15	8.33968	2.37429	13
−2	11	15	12.7046	3.06950	10
2	11	−15	8.11999	1.48547	11
1	−11	−15	2.27033	3.20946	10
−1	−11	15	2.95384	2.07830	13
1	11	−15	3.52591	2.06713	14
0	−11	15	12.4390	2.35097	13
0	11	−15	17.5499	2.86868	14
−3	−12	15	32.7069	3.78677	13
−3	12	15	35.1143	3.78456	10
−2	−12	15	10.2800	2.53281	13
2	12	−15	5.96807	0.94297	11
−2	12	15	9.50987	2.34340	10
2	12	−15	7.28747	0.95801	11
−1	−12	15	9.29404	2.58496	13
5	0	−16	1.59328	2.19921	10
4	0	−16	6.54049	1.95827	10
3	0	−16	5.58416	2.49814	10
2	0	−16	1.47596	2.24477	14
2	0	−16	1.14735	1.57147	10
1	0	−16	18.7637	2.94170	10
1	0	−16	16.4430	2.54265	14
0	0	−16	63.6963	5.05793	14
0	0	−16	59.9963	4.74583	10
0	0	−16	58.6146	4.74423	12
0	0	16	62.4552	5.65741	13
5	1	−16	0.76407	1.42232	10
4	−1	−16	6.19704	2.45613	10
4	1	−16	7.05241	2.03328	10
3	−1	−16	3.03458	1.75092	10
3	1	−16	6.15430	1.89559	10
2	−1	−16	6.20164	2.05681	14
2	−1	−16	5.41200	1.94403	10
2	1	−16	5.75337	2.39423	10
2	1	−16	12.2693	2.18291	14
1	−1	−16	5.75202	2.05394	14
1	−1	−16	7.19991	2.19981	10
−1	−1	16	9.75601	2.77036	13
1	1	−16	7.20243	2.04879	14
1	1	−16	5.83033	1.97171	10
0	−1	−16	30.1876	3.08461	10
0	−1	16	27.3391	4.41015	13
0	−1	−16	39.2602	3.68456	14
0	−1	−16	35.3872	3.23269	12
0	1	−16	25.4367	3.55838	14
0	1	16	35.5325	4.21410	13
0	1	−16	29.1477	3.86615	12
5	2	−16	1.64198	2.10328	10
4	−2	−16	8.47301	2.10480	10

4	2	−16	7.59323	2.05220	10
3	−2	−16	3.51381	2.34012	10
3	2	−16	2.11966	1.62490	10
2	−2	−16	34.8249	3.78275	14
2	−2	−16	32.1571	3.53378	10
2	2	−16	30.0085	4.04583	14
2	2	−16	24.2192	3.15454	10
1	−2	−16	60.6107	4.93182	14
1	−2	−16	54.6236	4.95472	10
1	−2	−16	55.6999	4.47158	12
−1	−2	16	56.1022	5.23192	13
1	2	−16	51.7850	4.58154	10
1	2	−16	52.1929	4.86231	14
0	−2	−16	11.1325	1.71386	14
0	−2	−16	9.98370	2.15115	10
0	−2	16	6.16972	2.24394	13
0	−2	−16	11.5169	1.87435	12
0	2	−16	6.60487	1.42095	11
0	2	−16	5.22504	1.86708	14
5	3	−16	2.04668	2.11366	10
4	−3	−16	4.61826	1.74821	10
4	3	−16	1.09521	2.22872	10
3	−3	−16	1.42648	1.67736	10
3	3	−16	0.15025	2.11591	10
2	−3	−16	8.04782	2.45639	10
2	3	−16	7.28093	1.79251	10
2	3	−16	8.04205	2.28209	14
−1	−3	16	44.0143	4.70431	13
1	−3	−16	42.3272	3.97119	10
1	3	−16	42.6418	4.04135	10
1	3	−16	44.1574	4.27923	14
0	−3	−16	3.27054	1.68155	10
0	−3	16	6.22322	2.58630	13
0	−3	−16	2.66330	1.51390	12
0	3	−16	7.61066	1.17475	11
0	3	−16	7.31858	1.91798	14
5	4	−16	2.20471	1.58690	10
4	−4	−16	21.3584	2.94223	10
−4	4	16	20.1139	3.06461	10
4	4	−16	19.5162	2.85226	10
3	−4	−16	−1.0327	2.15211	10
3	4	−16	0.16652	2.00102	10
−2	−4	16	2.99747	2.06370	13
2	−4	−16	2.84441	1.72489	10
2	4	−16	5.44902	1.95016	10
2	4	−16	3.07398	2.41964	14
−1	−4	16	7.77637	2.37585	13
1	−4	−16	12.8366	2.46896	10
1	4	−16	7.66520	2.05625	14
1	4	−16	7.18545	1.84104	10

```
   0  -4 -16 20.0104 2.74450  12
   0  -4  16 26.4205 3.72708  13
   0  -4 -16 16.1529 2.77840  10
   0   4 -16 26.6107 3.28568  14
   0   4 -16 25.6881 2.21536  11
  -4   5  16 4.12444 1.81034  10
   4   5 -16 3.56024 2.09011  10
   3  -5 -16 2.45209 1.67560  10
   3   5 -16 6.13062 2.14965  10
   2  -5 -16 2.27309 1.56891  10
  -2  -5  16 2.38887 2.06400  13
   2   5 -16 2.38330 1.62060  10
  -1  -5  16 3.38129 2.09451  13
   1  -5 -16 3.38295 1.54781  10
   1   5 -16 2.09058 1.77445  14
   1   5 -16 4.18948 1.35844  11
   0  -5  16 24.0638 4.11976  13
   0  -5 -16 18.2703 2.73247  10
   0  -5 -16 18.0129 2.52331  12
   0   5 -16 19.7712 3.00018  14
   4   6 -16 5.11529 1.62382  10
   3  -6 -16 4.91632 1.73442  10
   3   6 -16 2.63773 1.80148  10
  -2  -6  16 7.49295 2.17364  13
   2  -6 -16 5.39803 1.82978  10
   2   6 -16 5.38764 1.59856  10
  -1  -6  16 16.2277 2.91006  13
   1  -6 -16 11.3951 2.31886  10
   1   6 -16 15.2399 1.78093  11
   1   6 -16 14.9005 2.24940  14
   4   7 -16 -0.5349 1.69594  10
   3   7 -16 6.43781 1.80158  10
  -2  -7  16 25.2581 3.57043  13
   2  -7 -16 22.2691 3.52906  10
   1  -7 -16 11.4403 2.46872  10
  -1  -7  16 14.3759 2.49799  13
   1   7 -16 16.0540 1.78565  11
   1   7 -16 14.6313 2.17427  14
  -2  -8  16 19.8997 3.28882  13
  -1  -8  16 5.05938 2.63573  13
   0   0   0    0.00    0.00   0
;
_shelx_hkl_checksum               12380
_shelx_SHELXL_version_number      '2014/7'
_olex2_submission_special_instructions 'No special instructions were
received'
```
